# *k*-mer manifold approximation and projection for visualizing DNA sequences

**DOI:** 10.1101/gr.279458.124

**Published:** 2025-05

**Authors:** Chengbo Fu, Einari A. Niskanen, Gong-Hong Wei, Zhirong Yang, Marta Sanvicente-García, Marc Güell, Lu Cheng

**Affiliations:** 1 Department of Computer Science, School of Science, Aalto University, 02150 Espoo, Finland;; 2 Institute of Biomedicine, University of Eastern Finland, 70211 Kuopio, Finland;; 3 Fudan University Shanghai Cancer Center & MOE Key Laboratory of Metabolism and Molecular Medicine and Department of Biochemistry and Molecular Biology of School of Basic Medical Sciences, Shanghai Medical College of Fudan University, 200032 Shanghai, China;; 4 Disease Networks Research Unit, Faculty of Biochemistry and Molecular Medicine, Biocenter Oulu, University of Oulu, 90220 Oulu, Finland;; 5 Department of Computer Science, Norwegian University of Science and Technology, 7491 Trondheim, Norway;; 6 Jinhua Institute of Zhejiang University, 321032 Zhengjiang, China;; 7 Department of Medicine and Life Sciences, Universitat Pompeu Fabra, 08003 Barcelona, Spain;; 8 Institució Catalana de Recerca i Estudis Avançats, ICREA, 08003 Barcelona, Spain

## Abstract

Identifying and illustrating patterns in DNA sequences are crucial tasks in various biological data analyses. In this task, patterns are often represented by sets of *k*-mers, the fundamental building blocks of DNA sequences. To visually unveil these patterns, one could project each *k*-mer onto a point in two-dimensional (2D) space. However, this projection poses challenges owing to the high-dimensional nature of *k*-mers and their unique mathematical properties. Here, we establish a mathematical system to address the peculiarities of the *k*-mer manifold. Leveraging this *k*-mer manifold theory, we develop a statistical method named KMAP for detecting *k*-mer patterns and visualizing them in 2D space. We applied KMAP to three distinct data sets to showcase its utility. KMAP achieves a comparable performance to the classical method MEME, with ∼90% similarity in motif discovery from HT-SELEX data. In the analysis of H3K27ac ChIP-seq data from Ewing sarcoma (EWS), we find that BACH1, OTX2, and KNCH2 might affect EWS prognosis by binding to promoter and enhancer regions across the genome. We also observe potential colocalization of BACH1, OTX2, and the motif CCCAGGCTGGAGTGC in ∼70 bp windows in the enhancer regions. Furthermore, we find that FLI1 binds to the enhancer regions after ETV6 degradation, indicating competitive binding between ETV6 and FLI1. Moreover, KMAP identifies four prevalent patterns in gene editing data of the AAVS1 locus, aligning with findings reported in the literature. These applications underscore that KMAP can be a valuable tool across various biological contexts.

DNA sequence serve as the primary carrier of genetic information. In diverse research contexts, investigators aim to uncover patterns within DNA sequences, a pursuit central to various applications. Among the most prominent of these is the study of transcription factor (TF) DNA-binding specificity. Researchers employ methods like SELEX-seq (Kinzler and Vogelstein 1990; Tuerk and Gold 1990; Jolma et al. 2013) and ChIP-seq (Johnson et al. 2007; Wei et al. 2010) to determine the binding specificities of TFs. SELEX-seq, an in vitro technique, typically examines a single TF at a time, whereas H3K27ac ChIP-seq, which targets active regulatory regions, an in vivo approach, allows for the investigation of the binding of multiple TFs. The DNA sequences obtained from these methodologies provide crucial biological insights into TF activities, such as their DNA-binding specificities. Furthermore, DNA sequencing is also utilized to study the effect of gene editing protocols (Sanvicente-García et al. 2023), in which researchers are interested in discovering the editing patterns. Therefore, the DNA sequence encapsulate a wide array of biological information, varying according to the specific application in focus.

*k*-mers are foundational in bioinformatics, especially for motif discovery, as they enable the efficient identification of conserved regions and regulatory elements across genomes. The Jellyfish algorithm, introduced by Marçais and Kingsford (2011), is a widely used *k*-mer counting tool that emphasizes the utility of *k*-mers in detecting frequent motifs and conserved sequences. Zielezinski et al. (2017) demonstrated the effectiveness of alignment-free, *k*-mer-based methods for uncovering conserved genomic motifs and analyzing sequence similarity, whereas Fan et al. (2015) explored *k*-mers in comparative genomics, showing how they can approximate phylogenetic relationships and reveal conserved regions. Although these approaches focus on *k*-mer similarities, they do not address the structure of the *k*-mer manifold, which could offer deeper insights into relationships among *k*-mers and further enhance motif discovery.

To extract the biological information from DNA sequences, researchers usually convert these sequences into *k*-mers (Marçais and Kingsford 2011). The *k*-mer distribution mirrors the underlying biological information, allowing for the identification of potential patterns. For instance, in SELEX-seq data, *k*-mers relevant to the DNA-binding specificity of a certain TF may cluster together (Yuan et al. 2019). Similarly, in gene editing data sets, multiple clusters might represent editing patterns. Therefore, it would be very convenient for researchers to explore the *k*-mer distribution by projecting each *k*-mer to a point in the two-dimensional (2D) Euclidean space. However, this task presents two main challenges. First, the *k*-mer space is extremely large. The total number of possible *k*-mers is 4^*k*^, which grows exponentially as *k* increases. It brings a huge computation load to project so many points to the 2D space when *k* is large (Marçais and Kingsford 2011). Human eyes struggle to discern real patterns among a dense collection of data points in 2D space, in which meaningful signals may be overwhelmed by noisy points. Second, the discrete nature of *k*-mers has introduced special topological properties to the *k*-mer space, which forms the *k*-mer manifold. There are intrinsic conflicts between the *k*-mer manifold and the Euclidean space, whereas current dimensionality reduction methods are designed for the continuous Euclidean space.

Classical motif discovery algorithms such as MEME (Bailey and Elkan 1994), HOMER (Heinz et al. 2010), STREME (Bailey 2021) address the issue of *k*-mer explosion by filtering *k*-mers that are relevant to a motif pattern. These selected *k*-mers are used to construct a position weight matrix (PWM) representing the motif. However, this approach typically focuses on individual motifs one at a time, failing to capture the full spectrum of patterns in the *k*-mer distribution. For example, the relative strengths between different patterns are not directly observable. Displaying all patterns within a single figure would be significantly more informative, offering a comprehensive view of the underlying biological information.

Because of the special properties of the *k*-mer manifold, researchers rarely get satisfactory results in *k*-mer visualization. Kruppa et al. (2017) proposed visualizing the *k*-mer distribution as bubbles anchored on a pyramid, but this method lacked intuitiveness. Yi et al. (2021) randomly scattered *k*-mers in a 2D space, yet this approach failed to reveal underlying motif patterns. Classical dimensionality reduction methods designed for Euclidean space, such as principal component analysis (PCA) (Hotelling 1933) and multidimensional scaling (MDS) (Torgerson 1952), are linear dimensionality reduction methods, which cannot cope with peculiarities of the *k*-mer manifold. Other methods such as t-SNE (Van der Maaten and Hinton 2008) and UMAP (McInnes et al. 2018) could handle data from a nonlinear perspective, but they face the issue of gradient explosion, especially when the space is discrete and there are a large number of duplicate points in the input. Yuan et al. (2019) alleviate this problem by learning a high-dimensional continuous embedding (*d* = 300) using a supervised neural network that matches a bag of *k*-mers with a single TF label, which learns the embeddings of TFs instead of *k*-mers. Their approach, however, is not suitable for the *k*-mer manifold visualization task here, which is inherently an unsupervised task. Deterministic unsupervised neural networks like autoencoder (Kramer 1992) suffer from the identical mapping problem that identical *k*-mers would be projected to the same point, which leads to the loss of the density information. Probabilistic unsupervised neural networks, such as variational autoencoders (VAEs) (Khemakhem et al. 2020), offer a promising alternative. VAEs have been used for motif discovery from ATAC-seq data (Kshirsagar et al. 2022), although their potential for *k*-mer manifold visualization remains untapped.

In this study, we aim to project each *k*-mer onto a point in 2D space to provide an intuitive visualization of the *k*-mer distribution. The key to achieving this objective is to explore the unique properties of the *k*-mer manifold. As far as we are aware, there is not a proper theoretical formalization of the *k*-mer manifold in the field of bioinformatics. To fill this gap, we have built up the mathematical theories for describing the *k*-mer manifold. Leveraging this theoretical framework, we examined the probability distribution of *k*-mers, introduced the concept of Hamming ball, and developed a motif discovery algorithm, such that we could sample relevant *k*-mers to depict the full *k*-mer manifold. After that, we performed transformations to the *k*-mer distances based on the *k*-mer manifold theory to mitigate the inherent discrepancies between the *k*-mer manifold and the 2D Euclidean space. Finally, we developed the KMAP visualization algorithm to project *k*-mers to 2D space by extending the UMAP framework. This study aims to demonstrate KMAP's utility in various biological data sets, enhancing motif discovery and sequence data exploration.

## Results

### *k*-mer manifold theory and KMAP workflow

**Figure 1.**
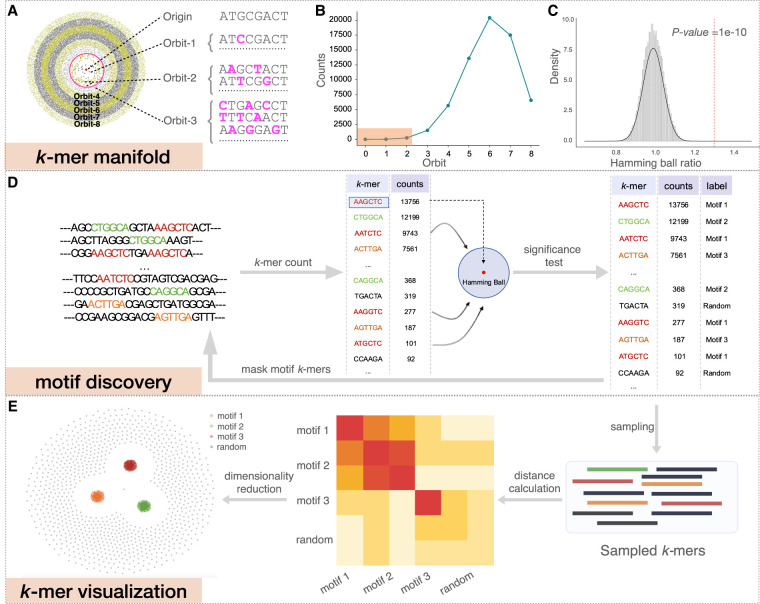
KMAP workflow. (*A*) Schematic illustration of the *k*-mer manifold for *k* = 8. Each point represents a unique *k*-mer. Orbit-*i* consists of *k*-mers with *i* mutations to the origin; namely, the Hamming distance from the *k*-mer to the origin is *i*. *k*-mers in the *i*th orbit are uniformly scattered in the *i*th ring, where each ring has an equal width. *k*-mers within the red circle forms the Hamming ball centered on the origin with a radius *r*^(*k*)^ = 2. (*B*) *k*-mer counts of each orbit. The rectangle highlights the *k*-mer counts of orbits in the Hamming ball. (*C*) Null distribution of Hamming ball ratio. The histogram is generated by taking all Hamming ball ratios from a random DNA sequence of 100,000 bp. The experiment is repeated 10 times, and a Gaussian distribution is fitted to the obtained ratios with the mean fixed to one. The fitted Gaussian distribution is used as the null distribution, in which the vertical dashed line indicates the significant ratio corresponding to a *P*-value of 1 × 10^−10^. (*D*) The motif discovery workflow. We first count the *k*-mers and then test the Hamming ball centered on the top *k*-mer; after that, we mask all motif *k*-mers from the input DNA sequence and repeat the process iteratively until no motif can be found. (*E*) *k*-mer visualization algorithm; 2500 motif *k*-mers and 2500 random *k*-mers are sampled for the visualization. The Hamming distance matrix of the sampled *k*-mers is smoothed and further utilized for dimensionality reduction.

We have built up the mathematical theories for studying the *k*-mer manifold, in which the most important conclusions are illustrated in Figure 1, A through C. Let us denote the *i*th *k*-mer by $s_{i}^{\left(k\right)}=s_{i1}^{\left(k\right)}s_{i2}^{\left(k\right)}\cdots s_{ij}^{\left(k\right)}\cdots s_{ik}^{\left(k\right)}$. Here $s_{ij}^{\left(k\right)}\in \{A,C,G,T\;\}$, and *k* is the length of the *k*-mer. There are 4^*k*^ unique *k*-mers in the *k*-mer space. By selecting a *k*-mer as the origin of the *k*-mer space, we could partition all *k*-mers into *k* + 1 orbits (Fig. 1A), where *k*-mers of the *i*th orbit (*i* = 0, 1, 2, …, *k*) have *i* mutations compared with the origin. In other words, the Hamming distance between each *k*-mer in the *i*th orbit, and the origin is *i*. The *k*-mer manifold Ω^(*k*)^ is jointly defined by the origin, the metric (Hamming distance), and all *k*-mers in the *k*-mer space. In Supplemental Note S1, we derived the count of *k*-mers in each orbit of Ω^(*k*)^:

$$|A_{i}^{\left(k\right)}|=C_{k}^{i}\;*\;{\left(4-1\right)}^{i}.$$

We have proved that this function is an unimode function of the orbit index *i* (Fig. 1B), with the mode located near $\frac{3}{4}k$. As illustrated by Figure 1A, orbits 5, 6, and 7 have the densest points, at which $\frac{3}{4}k=\frac{3}{4}8=6$. We define *k*-mers of orbits within a radius of *r*^(*k*)^ as a *Hamming ball* (Fig. 1A, red circle) to represent *k*-mers that are similar to the origin. As shown in Figure 1B, the count of *k*-mers within the Hamming ball is relatively small (∼0.4%) for *k* = 8 and *r*^(*k*)^ = 2. *r*^(*k*)^ is a predefined value for different *k* (see Supplemental Note S1, Section 5, Supplemental Table S1). Figure 1C provides the empirical distribution of the ratio between the empirical probability and the theoretical probability for all Hamming balls from a random DNA sequence, which we term as the *Hamming ball ratio*. The empirical probability of a Hamming ball is given by the proportion of *k*-mers within the Hamming ball out of all *k*-mers in the input DNA sequence, whereas the theoretical probability is given by (see Supplemental Note S1, Section 5)

$$\begin{array}{c} \;p_{\mathrm{unif}}(B^{\left(k\right)})=\frac{\left|B^{\left(k\right)}\right|}{\left|\Omega^{\left(k\right)}\right|}=\frac{\left|hb(\Omega^{\left(k\right)},o^{\left(k\right)},r^{\left(k\right)})\right|}{\left|\Omega^{\left(k\right)}\right|}\\ =\frac{\sum_{i=0}^{r^{\left(k\right)}}\left|A_{i}^{\left(k\right)}\right|}{4^{k}}\;. \end{array}$$

We fit a Gaussian distribution to the Hamming ball ratios over all *k*-mers with the mean fixed to one and use it as the null distribution. Based on practical experiences, we set the *P*-value threshold to 1 × 10^−10^ for a Hamming ball ratio to be considered as significant. Detailed description of the *k*-mer manifold theory is given in Supplemental Note S1, in which we also discuss reverse complements of *k*-mers and how to treat them in practice.

The motif discovery algorithm (Fig. 1D) is based on the *k*-mer manifold theory, particularly, the null distribution of the Hamming ball ratio. We have proved that the *k*-mer manifold is isotropic; namely, we could generate the whole *k*-mer space by centering on any *k*-mer (as origin) in the manifold. Therefore, a motif can be represented by a Hamming ball, in which the origin is termed as the *consensus sequence* of the motif. From real sequencing data, we can count *k*-mers and calculate the actual probability of a Hamming ball centered on a high-count *k*-mer, which is tested against the null distribution. Hamming balls with a *P*-value less than the significance threshold are kept as motifs. It is difficult to visualize the *k*-mer space as it is extremely large; for example, only 0.4% *k*-mers land in the Hamming ball, and majority *k*-mers are random *k*-mers in Figure 1A. Here we term *k*-mers within a motif Hamming ball as *motif k*-mer*s* and other *k*-mers as *random k*-mer*s*. We perform sampling of *k*-mers for visualization after motif discovery. Half of the sampled *k*-mers are motif *k*-mers, whereas the other half are random *k*-mers. *k*-mers from different motifs are pooled and sampled with the weights given by their counts. Random *k*-mers are sampled in a similar manner to reflect the background noise. A detailed description of the motif discovery algorithm is provided in Supplemental Note S2.

We visualize the sampled *k*-mers *X* = (***x***_1_, ***x***_2_, … , ***x***_*N*_) by projecting them to 2D space (Fig. 1E). First, we calculate the Hamming distance matrix between the *k*-mers. Then, we compute the smoothed distance matrix by pulling neighboring *k*-mers and repulsing distant *k*-mers. Neighboring *k*-mers are pulled closer by the following distance transformation:

(1) $$d_{0}(x_{i},x_{j})=\frac{1}{\left|N_{i}||N_{j}\right|}\underset{s_{m}\in N_{i}}{\sum }\underset{s_{n}\in N_{j}}{\sum }d_{H}(s_{m},s_{n}),$$

where ***s***_*m*_ and ***s***_*n*_ are one of the 20 nearest neighbors of ***x***_*i*_ and ***x***_*j*_, respectively. Here *N*_*i*_ and *N*_*j*_ denote the 20 nearest neighbors of ***x***_*i*_ and ***x***_*j*_. *d*_*H*_(***s***_*m*_, ***s***_*n*_) denotes the Hamming distance between ***s***_*m*_ and ***s***_*n*_.

Distant *k*-mers are repulsed away by the following transformation:

(2) $$f(x)=\frac{16}{1+e^{-\gamma (x-x_{0})}},\;$$

where *x* is the transformed distance (Equation 1), γ = 0.2*k* − 0.2 controls the curvature of the transformation, $x_{0}=\frac{k}{2}$ is the change point parameter, and *k* is the length of the input *k*-mer. *x*_0_ is the rough boundary between Hamming ball and the outer orbits. According to Remark 1.1 in Supplemental Note S1, the expected distance between two random *k*-mers is $\frac{3}{4}k$, namely, a *k*-mer in the Hamming ball and a *k*-mer in the outer orbits. Hence, we choose $x_{0}=\frac{k}{2}$ as the rough boundary.

**Figure 2.**
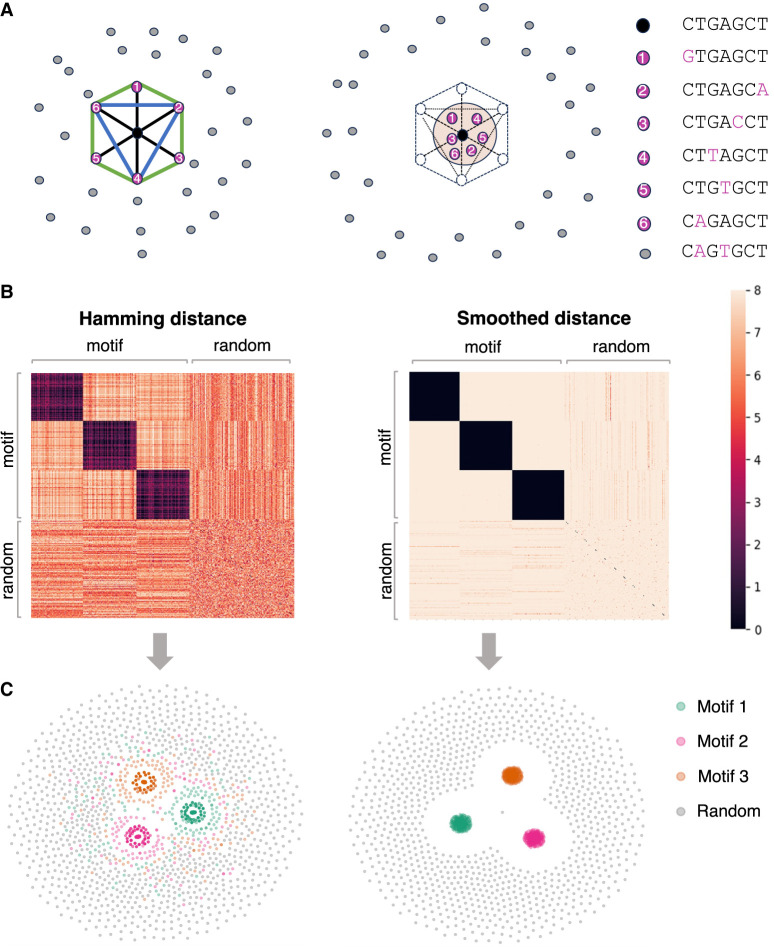
Peculiarities of *k*-mer manifold. (*A*) Six motif *k*-mers (purple dots) with one mutation from the origin (black dot) placed as a hexagon on a circle of radius one, and random *k*-mers (gray dots) are placed outside. The Hamming distance between each pair of motif *k*-mers is two. The Euclidean distance between the dots are two, $\sqrt{3}$, and one for the diagonal (black line), semidiagonal (blue line), and adjacent *k*-mers (green line). The *right* panel shows the schematic effects of Hamming distance transformations (Equations 1, 2), in which motif *k*-mers are pulled closer and random *k*-mers are repulsed further. (*B*) Toy example. The *left* panel shows the Hamming distance matrix of a *k*-mer (*k* = 8) data set with three motifs, as highlighted by the black blocks. Within a motif, the Hamming distance ranges from zero to four. The *right* panel shows the transformed Hamming distance matrix. After transformation, the distances between the motif *k*-mers are reduced, whereas the distances between the motif and random *k*-mers become larger. (*C*) KMAP visualizations based on the original Hamming distance matrix (*left*) and the transformed Hamming distance matrix (*right*).

These transformations try to mitigate the intrinsic conflicts between Hamming distance and Euclidean distance. As shown in Figure 2A, all six *k*-mers have exactly one mutation from the consensus sequence, so we arrange them on a circle of radius one. However, the Hamming distance between any pair of *k*-mers is two. There is no way to arrange the *k*-mers on the circle that satisfies these constraints. The most intuitive solution is to place them as a hexagon on the circle. Although the Euclidean distances between diagonal *k*-mers (black edges) are two, the Euclidean distances between adjacent *k*-mers (green edges) and semidiagonal *k*-mers (blue edges) are one and $\sqrt{3}$, which are less than the designed Hamming distance of two. As a result, directly utilizing the Hamming distances leads to inferior results. Figure 2A (right panel) shows the effect of distance smoothing, which pulls neighboring *k*-mers and repulses distant *k*-mers, as well as introduces randomness to the distances. We demonstrate the Hamming distance matrix and its transformation on a toy example with three motifs in Figure 2B. KMAP visualizations based on the original and transformed Hamming distance matrices are provided in Figure 2C. It can be seen that the transformation pulls motif *k*-mers closer and repulses random *k*-mers further, which improves the visualization effect.

The KMAP visualization algorithm projects the high-dimensional *k*-mers to 2D space, given the transformed Hamming distance matrix. We denote the *i*th *k*-mer as ***x***_*i*_ and its 2D embedding as ***w***_*i*_ = (*w*_*i*0_, *w*_*i*1_), where *i* = 1, 2, …, *N*. We use the same cross entropy loss function as UMAP for dimensionality reduction:

$$L=\underset{i}{\sum }\underset{j}{\sum }p_{ij}\log\frac{\;p_{ij}}{q_{ij}}+(1-p_{ij})\log\frac{\left(1-p_{ij}\right)}{\left(1-q_{ij}\right)},$$

where *p*_*ij*_ is the similarity probability of the high-dimensional data, given by

$$p_{ij}=\exp\left(\frac{-d(x_{i},x_{j})}{2\sigma^{2}}\right),$$

and *q*_*ij*_ is the similarity probability of the low-dimensional embeddings, given by

$$q_{ij}=\frac{1}{1+{\left|\left|w_{i}-w_{j}\right|\right|}^{2}}.$$

It is obvious that the loss function reaches its minimum when *p*_*ij*_ = *q*_*ij*_ for all *i* ≠ *j*. By optimizing the loss function, we try to find embeddings that generate *q*_*ij*_ as close as *p*_*ij*_, such that the low-dimensional embeddings could represent the high-dimensional manifold. We use the gradient descent algorithm for the optimization. The gradient is given by

$$\frac{\partial L}{\partial w_{i}}=4\underset{j}{\sum }(\;p_{ij}-q_{ij})\frac{1}{||w_{i}-w_{j}|{|}^{2}}(w_{i}-w_{j}).$$

We notice that the term ||***w***_*i*_ − ***w***_*j*_||^2^ can easily go to zero in the optimization, which causes gradient exploding. We add the following diffusion terms to *w*_*j*_ to avoid this problem:

$$w_{j}^{\prime }=\{\begin{array}{cc} w_{j}+\epsilon \; & \mathrm{if}\;|\left|w_{i}-w_{j}\right||\leq 0.1\;\\ w_{j}\; & \mathrm{otherwise}\; \end{array},$$

where $\epsilon =(\epsilon_{0},\epsilon_{1})$ and $\epsilon_{0},\epsilon_{1}∼N(0,0.01^{2})$ are two independent Gaussian samples. Detailed description of the KMAP visualization algorithm is provided in Supplemental Note S3.

### HT-SELEX data analysis

**Figure 3.**
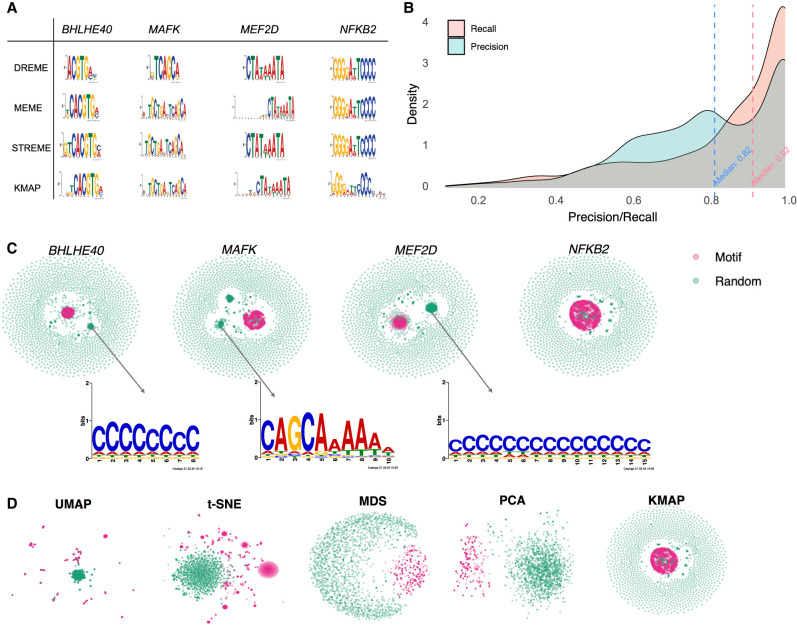
Benchmark on HT-SELEX data. (*A*) Motif logos of four example TFs given by DREME, MEME, STREME, and KMAP. (*B*) Distribution of precision and recall scores between MEME and KMAP. The scores are calculated for 1273 samples (SELEX rounds 3–6), with the corresponding medians highlighted by the dashed vertical lines. (*C*) KMAP visualizations of example TFs. Motif and random *k*-mers are highlighted in red and green, respectively. The logos illustrate exemplary secondary motifs of TFs. (*D*) *k*-mer visualizations of NFKB2 based on UMAP, t-SNE, MDS, and PCA. KMAP visualization of NFKB2 is highlighted in the rectangle above.

We demonstrate KMAP's performance in motif discovery on a public high-throughput SELEX (HT-SELEX) data set (Jolma et al. 2013), which contains 461 TFs. We analyze 1273 samples of SELEX rounds 3–6 for these TFs, which have stronger motif signals compared with rounds 1–2. Figure 3A shows motif logos for four example TFs: BHLHE40, MAFK, MEF2D, and NFKB2 given by KMAP and by classical methods such as MEME, STREME, DREME. It can be seen that motifs given by different methods are similar. We further compared the results of KMAP and MEME, in which only the top motif is compared. For each sample, we calculated the precision and recall scores from the consensus sequences given by KMAP and MEME using the following formulas:

$$\mathrm{precision}=\frac{\left|\mathrm{overlap}(s_{K},s_{M})\right|}{\left|s_{K}\right|},$$

$$\mathrm{recall}=\frac{\left|\mathrm{overlap}(s_{K},s_{M})\right|}{\left|s_{M}\right|},$$

where | · | takes the length of a given sequence, and ***s***_*K*_ and ***s***_*M*_ denote the consensus sequences provided by KMAP and MEME, respectively. Figure 3B shows the distribution of the precision and recall scores over all samples. The precision and recall medians between KMAP and MEME are 82% and 92%, which suggests a high consistency between KMAP and MEME. Note that the precision is generally smaller than the recall, which suggests that KMAP generates longer motifs than MEME.

We show the KMAP 2D embeddings of *k*-mers for the example TFs in Figure 3C, in which secondary motifs could be observed for BHLHE40, MAFK, and MEF2D. These secondary motifs are distinct to the major motifs. Figure 3D illustrates the dimensionality reduction results of UMAP, t-SNE, MDS, PCA, and KMAP, which are generated using the same set of *k*-mers of NFKB2. It can be seen that motif *k*-mers (red dots) do not form a clear cluster in the PCA and MDS embeddings. Because of gradient exploding, t-SNE and UMAP stop after a few iterations. We can see that motif *k*-mers are scattered around and random *k*-mers form a cluster, which is likely owing to initialization. KMAP provides the most intuitive representation of the *k*-mer manifold, in which Hamming balls form clusters in the center, and random *k*-mers are placed in the peripheral space. KMAP motif logos and 2D visualization plots for all 1273 TFs are provided in Supplemental Data S1. On average, KMAP's runtime for motif discovery on this HT-SELEX data set is shorter than that of MEME (Supplemental Fig. S1).

### Ewing sarcoma data analysis

**Figure 4.**
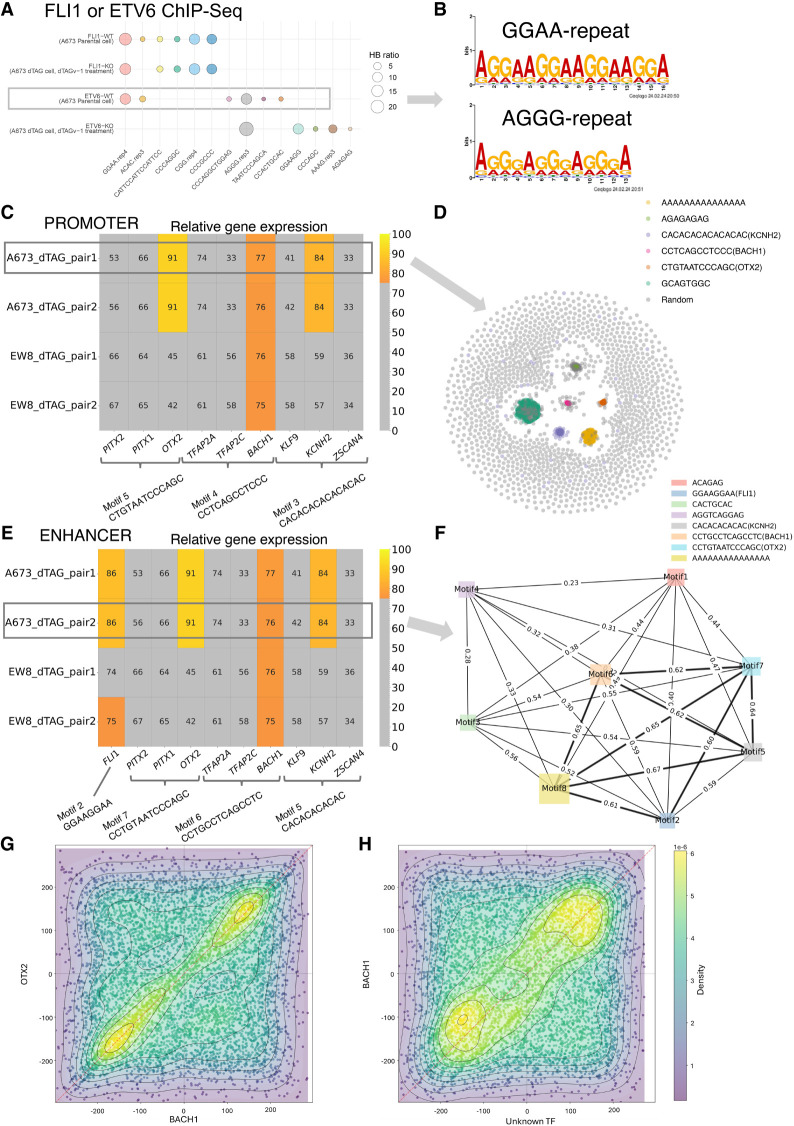
Ewing sarcoma (EWS) data analysis. (*A*) Motifs identified by KMAP from FLI1 or ETV6 ChIP-seq data. (WT) A673 parental cells, (KO) dTAG^V^-1 treated ETV6-dTAG cells derived from A673 parental cells. In the motifs, “rep4” means repeat larger or equal to four times, and a similar rule applies to “rep3.” The circle size indicates the Hamming ball ratio. (*B*) Motif logos of GGAA and AGGG repeats generated from motif *k*-mers, based on ChIP-seq peaks of the ETV6-WT sample. (*C*) Expression levels of potential promoter region–associated TFs. Each row is a sample pair, namely, dTAG cells treated with dTAG^V^-1 (ETV6 degraded) and DMSO (ETV6 intact), in which newly gained promoter regions (ETV6 degraded vs. intact) are used as the input for KMAP. The columns are the potential TFs of the identified motifs. Note that FIMO returns multiple TFs for a single input consensus sequence. For each pair, gene expressions in the corresponding ETV6 degraded sample (dTAG^V^-1 treated) are ranked, and the ranks are converted to relative expression 0%–100%, in which top 25% genes are colored, and the rest of the genes are gray. The number in each cell shows the relative expression. The motif consensus sequences are obtained from the “A673_dTAG_pair1” pair highlighted by the rectangle. (*D*) KMAP visualization of *k*-mers in the promoter region, based on newly gained ChIP-seq peaks (ETV6 degraded vs. intact) from the “A673_dTAG_pair1” pair. (*E*) Expression levels of potential TFs in enhancer regions. The motif consensus sequences are extracted from the “A673_dTAG_pair2” pair. (*F*) Co-occurrence network of identified motifs from the “A673_dTAG_pair2” pair. The edge weight indicates the proportion of ChIP-seq peaks that contain both TFs out of all peaks that contain at least one TF. The node size indicates the Hamming ball ratio. (*G*) Motif positions of BACH1 and OTX2 on enhancer regions (400–600 bp) from “EW8_dTAG_pair2.” Each dot represents a pair of BACH1 and OTX2 occurrences within a single enhancer region. The center of each enhancer region is set as the origin, and motif positions are shown relative to this origin. (*H*) Motif positions of BACH1 and CCCAGGCTGGAGTGC on enhancer regions (400–600 bp) from “EW8_dTAG_pair2.”

We analyzed ChIP-seq data from an Ewing sarcoma (EWS) study (Lu et al. 2023). Lu et al. (2023) have found that ETV6 promotes the development of EWS by competitively binding to the binding sites of FLI1, which is also confirmed in another study (Gao et al. 2023). Lu et al. (2023) prepared ETV6-dTAG A673 and EW8 cells, from which ETV6 could be rapidly degraded by adding a small molecule called dTAG^V^-1, whereas ETV6 was intact if DMSO was added. From ETV6 or FLI1 ChIP-seq data of the parental A673 cells (WT), KMAP identified GGAA-repeats as the strongest motif for FLI1 and ETV6 (Fig. 4A). From ChIP-seq data of A673 ETV6-dTAG cells with dTAG^V^-1 treated, in which ETV6 was degraded, KMAP found the GGAA-repeat motif disappeared in ETV6 ChIP-seq data (Fig. 4A) but remained in FLI1 ChIP-seq data. The GGAA-repeat motif is found in 95.5% of input reads and shows a preference for central positioning (Supplemental Fig. S2A), suggesting it is likely a genuine motif. Figure 4B shows the motif logos generated from the Hamming ball of GGAA-repeats (16 bp) and AGGG-repeat (13 bp) motifs identified in the ETV6-WT sample. Both motifs occur at high frequencies (92% and 99.2%, respectively) in the input reads and show a preference for central positioning (Supplemental Fig. S2B). These findings have confirmed the original conclusion that ETV6 and FLI1 competitively bind to GGAA-repeats on the genome. It is worth mentioning that ETV6 also binds to AGGG-repeats, which is the second strongest motif in WT. The “GGAAGG” motif with a single “GGAA” repeat can be observed in the ETV6-KO sample, which suggests the existence of a small amount of ETV6 in the ETV6-KO sample. Because ETV6 is both an oncogenic gene and a repressor, we are interested in its target genes, which might inhibit the development of EWS.

Lu et al. (2023) provided the H3K27ac ChIP-seq data of A673 and EW8 ETV6-dTAG cells, which marked the promoter and enhancer regions of ETV6 target genes. There are eight samples divided into four pairs (Supplemental Table S1), each of which contains a control sample with intact ETV6 (DMSO treated) and a ETV6 degraded sample (dTAG^V^-1 treated). Each pair corresponds to a specific combination of the cell line (A673 or EW8) and the replicate; for example, “A673-dTAG-pair1” refers to A673 dTAG cells with dTAG^V^-1 (ETV6 degraded, replicate 1) and DMSO (ETV6 intact, replicate 1). For each pair, we extracted the H3K27ac ChIP-seq peaks that were gained upon ETV6 degradation. This was done by subtracting peaks of the ETV6 intact sample from that of the ETV6 degraded sample, namely, dTAG^V^-1 versus DMSO. The newly gained peaks were further classified into promoter and enhancer regions. KMAP identified eight unique motifs from the promoter regions. We next fed the consensus sequences of the eight unique motifs to FIMO (Grant et al. 2011) to retrieve the corresponding TFs, in which one consensus sequence might match several TFs. In total, FIMO identified three motifs with available annotations in at least one of the pairs. Assuming the potential TFs were highly expressed, we extracted the gene expressions of the matched TFs in the ETV6 degraded samples (dTAG^V^-1 treated). We ranked the expression levels of all genes and converted these ranks to relative expression values on a 0%–100% scale, with rank 1 set to 100% and the lowest rank set to 0%. Figure 4C shows the potential TFs binding to the promoter region, where BACH1 are identified as a motif in almost all pairs and have a high expression. OTX2 and KCNH2 are likely only associated with the A673 dTAG cells. Figure 4D shows the *k*-mer manifold of the newly gained ChIP-seq peaks (ETV6 degraded vs. ETV6 intact) from the A673-dTAG-pair1 pair, in which six motifs are observed.

Similar analysis of the enhancer regions (Fig. 4E) has identified eight motifs, four out of which have matched TFs after motif identification using FIMO. It can be seen that *FLI1* and *BACH1* have a high expression in almost all pairs, whereas *OTX2* and *KCNH2* are only associated with the A673 cells. Figure 4F illustrates the TF co-occurrence network generated from newly gained ChIP-seq peaks (ETV6 degraded vs. intact) from the “A673_dTAG_pair2” pair. This result shows that motifs 2, 5, 6, 7, and 8 (FLI1, KCNH2, BACH1, OTX2, A-repeats) have a high frequency of co-occurrence, whereas motifs 1, 3, and 4 occurs less frequently with other motifs.

We further examined the co-occurrence of identified TF motifs within the enhancer regions. Averaged across the four pairs, a larger proportion of enhancer regions contain motifs for FLI1 (75.8%), BACH1 (75.2%), OTX2 (66.2%), and the motif CCCAGGCTGGAGTGC (71.2%) compared with KCNH2 (36.6%), as shown in Supplemental Table S2. This higher prevalence suggests greater biological relevance for these motifs. Next, we analyzed their relative position distributions within enhancers, shown in Supplemental Figure S3, which indicates that these motifs are uniformly distributed across the enhancer regions. Further inspection of their precise locations revealed that BACH1, OTX2, and the motif CCCAGGCTGGAGTGC tend to colocalize within a ∼70 bp window in all four pairs (Fig. 4G,H; Supplemental Figs. S4, S5), with the distance between BACH1 and OTX2 being slightly smaller than that between BACH1 and CCCAGGCTGGAGTGC. No consistent patterns were observed in the distances between FLI1 and the other motifs.

These findings suggest that (1) ETV6 prevents the binding of BACH1, OTX2, and KCNH2 to the promoter and enhancer regions, which may have implications for EWS prognosis; (2) ETV6 and FLI1 competitively bind to the enhancer regions more than the promoter regions; and (3) BACH1, OTX2, and an unidentified TF with motif CCCAGGCTGGAGTGC potentially colocalize in ∼70 bp windows in the enhancer regions.

### Gene editing data analysis

**Figure 5.**
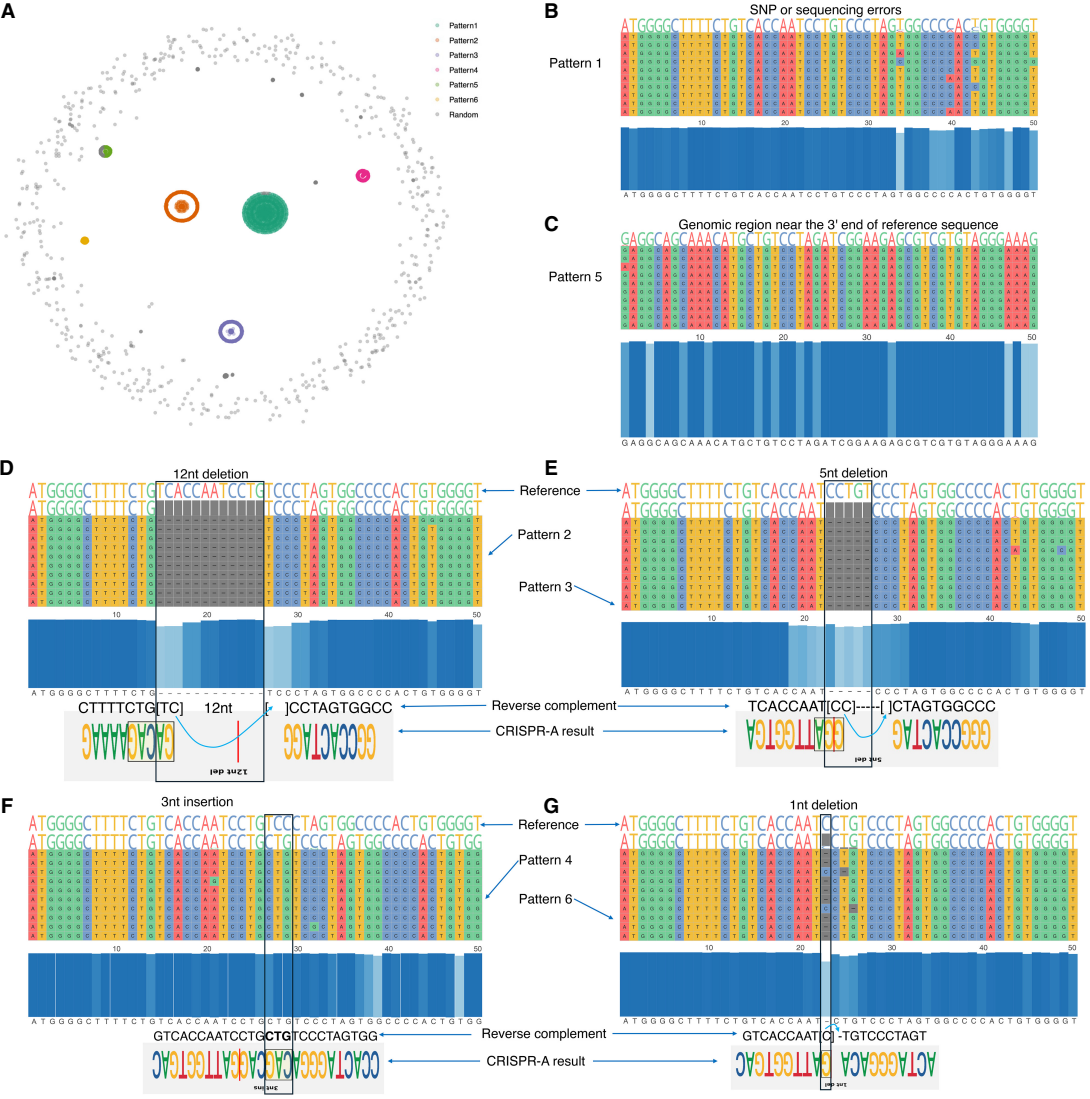
Gene editing patterns. (*A*) KMAP visualization of aligned DNA sequences. The six clusters are given by DBSCAN based on the 2D embeddings. (*B*–*G*) Sequence alignment of each pattern. Each row represents a DNA sequence. Ten sequences from each cluster are shown in the alignment to illustrate the corresponding pattern. The blue panel in the center shows the conservative nucleotide at each position. The top four gene editing patterns given by CRISPR-A are provided at the *bottom* panel for the last four patterns (*D*–*G*), above which are their reverse complements. Note that although the deletions in our result show 1–3 nt shifts compared with CRISPR-A's result, they are equivalent as the shifted nucleotides can be placed on either side of the deletion in the alignment. Pattern 1 is treated as SNPs or sequencing errors by CRISPR-A. Pattern 5 corresponds to a different genomic region.

We use KMAP to detect editing patterns from gene editing data of the adeno-associated virus site 1 (AAVS1) locus. Sanvicente-García et al. (2023) used a previously described (Mali et al. 2013; Doench et al. 2016) guide RNA with a 20 nt protospacer that targeted the genomic location (Chr 19: 55,115,752–55,115,771; GRCh38/hg38) on the human genome. The *Streptococcus* pyogenes Cas9 (SpCas9) protein will make a double-stranded break (DSB) at 3 nucleotides (nt) upstream of the PAM, after that the broken DNAs are ligated by non-homologous end-joining (NHEJ), which leads to different DNA editing results (mainly random small indels). Another cellular repair mechanism involved in the repair of DSB is microhomology-mediated end-joining (MMEJ), resulting in longer deletions led by homologous patterns in both sides of the cleavage. It is interesting to explore if there exist any patterns in the gene editing results, because patterns that lead to a certain repair resolution allow a higher rate of success to achieve certain outcomes, like efficient knockouts with out-of-frame deletions. From the gene editing results (FASTQ files) of the aforementioned experiment, we first removed DNA reads that were identical to the reference sequence, which represented unedited DNA. Next, we generated a multiple sequence alignment from the remaining reads using MUSCLE (Edgar 2004). After that, we calculated the pairwise distance matrix between the aligned reads and performed unsupervised sampling in KMAP (Supplemental Note S2, Algorithm 2). Given the Hamming distance matrix of the sampled reads (*n* = 2696), we directly performed dimensionality reduction using KMAP (Fig. 5A). Based on the 2D embeddings, we identified six clusters/patterns using DBSCAN (Ester et al. 1996). The patterns in our results nicely agree with that of Sanvicente-García et al.’s (2023) CRISPR-A platform. Pattern 1 (Fig. 5B) represents sequencing errors owing to poly nucleotide and high GC content in the protospacer and its flanking regions. Pattern 5 (Fig. 5C) represent reads from another genomic region near the 3′ end of the reference sequence that is distant to the protospacer. The remaining four patterns (Fig. 5D–G) have a one-to-one correspondence with Sanvicente-García et al.’s (2023) results, which represent a 12 nt MMEJ deletion, a 5 nt MMEJ deletion, a 3 nt CAG insertion adjacent to the cleavage site, and a 1 nt deletion at the cut site, respectively. Out of these four patterns, patterns 2 and 3 are the most abundant. Patterns 2 and 3, which show 12 nt and 5 nt deletions, respectively, likely result from MMEJ. MMEJ utilizes microhomologous sequences flanking a DSB to create deletions of variable lengths, depending on the available sequences. In contrast, pattern 4, which shows a 3 nt CAG insertion, and pattern 6, a 1 nt deletion at the cut site, are indicative of NHEJ. As an error-prone repair pathway, NHEJ introduces diversity in editing through random insertions or deletions during DNA end-processing before DNA repair.

We performed a similar analysis by inputting KMAP's pairwise distance matrix into UMAP, followed by clustering with DBSCAN. This resulted in five clusters/patterns. However, the visualization was suboptimal, and the five clusters lacked consistency among reads, differing significantly from true gene editing patterns, as shown in Supplemental Figure S6.

## Discussion

*k*-mer is an important object in various DNA sequence studies. A set of *k*-mers, or a Hamming ball in this context, can provide a description of the binding preferences of a given TF. If we turn an input sequence data into a set of *k*-mers, the *k*-mers form clusters that potentially have biological interpretations. An intuitive idea is to project *k*-mers to 2D space to visualize them.

*k*-mers are high-dimensional objects living in the *k*-mer manifold with special properties. We dive deep into the math to build up the theories for the *k*-mer manifold. The *k*-mer manifold is isotropic. By centering on any *k*-mer, the *k*-mer manifold can be partitioned into *k* + 1 orbits, in which majority *k*-mers are in orbits with indices close to $\frac{3}{4}k$. Although the Hamming ball contains a relatively large number of *k*-mers when *k* is large, they only constitute a tiny fraction of the whole *k*-mer manifold. We derived the null distribution of the Hamming ball ratio and used it for motif discovery. The reverse-complement operation adds an additional layer of symmetry to the *k*-mer manifold. We spend lots of efforts discussing how a Hamming ball is partitioned into four parts owing to the reverse-complement operation in Supplemental Note S1, which greatly helps the real data treatment. Additional discussions are provided in Supplemental Note S4, section 1, regarding the choice of the Hamming ball radius, nonuniform *k*-mer background distribution, and secondary motifs.

There exist intrinsic conflicts between the *k*-mer manifold and the Euclidean space. The metric of the *k*-mer manifold is the Hamming distance, which possesses counter-intuitive properties as shown in Figure 2A. We performed distance smoothing to harmonize the conflicts between Hamming distance and Euclidean distance. The idea is that the neighbors of a motif *k*-mer are likely still neighbors of another motif *k*-mer, but their distances to a random *k*-mer are still near $\frac{3}{4}k$, which allows us to further scale the distance to repulse random *k*-mers. The discreteness of the Hamming distance often induces zero to the denominator of a term in the gradient, which causes the gradient exploding problem and pretermination of the optimization. Adding a small diffusion term to identical embeddings can effectively solve this problem.

Beyond the distance smoothing and diffusion term, there is another difference between KMAP and UMAP. In UMAP, a point-specific scaling parameter is used to pull distant points such that the scaled distances to other points follow the standard Gaussian distribution. This treatment has a side-effect in the *k*-mer manifold, in which distant *k*-mers (random *k*-mers) are pulled closer and form a cluster. In KMAP, we do not perform point-specific scaling, which preserves the global structure of the *k*-mer manifold. Therefore, random *k*-mers are scattered in the peripheral space. Because of the huge volume (4^*k*^) of the *k*-mer manifold, we had to sample a subset of *k*-mers for the visualization. The bottleneck is the calculation of the distance matrix, which contains *n*^2^ elements for *n* input *k*-mers. Supplemental Note S4, section 2, provides additional discussions about the visualization algorithm, including how to choose the change point parameter, initializations of the 2D embeddings, and t-SNE's and UMAP's suboptimal performance on *k*-mer data.

The *k*-mer manifold theory has enabled us to study the *k*-mer distribution in different biological contexts. We have shown that KMAP provided similar results as MEME in the HT-SELEX data. In the EWS data, we found that BACH1-, OTX2-, and KCNH2-motif-containing promoters and enhancers are masked by ETV6. This suggest that BACH1, OTX2, and KCNH2 could be involved in alleviating EWS progression, which could be tested by activating the expression of BACH1, OTX2, and KCNH2. Activating of BACH1-, OTX2-, and the CCCAGGCTGGAGTGC-motif-containing enhancers could also result from increase in EWS-FLI activity at the enhancer regions upon ETV6 degradation. Further investigation is warranted to understand why these three motifs colocalize within ∼70 bp windows in the enhancers. Indel distribution visualization is very important for the gene editing community. The selection of gRNAs that induce low-complexity distributions that are absent in in-frame indels is highly desirable to maximize knock out implementation. In the gene editing data, KMAP has identified the major editing patterns. KMAP could be used for other tasks such as analyzing RNA–protein interactions (CLIP-seq), ATAC-seq, etc. As demonstrated in Supplemental Note S4, section 3, we present more applications of KMAP on several ATAC-seq data sets, composite motif analysis, and CTCF ChIP-seq data.

There are open questions from the method perspective. We use a heuristic to determine the motif length *k*. If a sequence and its subsequences are identified as motif sequences for three consecutive lengths *k* − 1, *k*, and *k* + 1, we use *k* as the final motif length. However, it is unclear how a Hamming ball changes when the motif length changes from *k* to *k* + 1. New theories are needed to depict the transformation of the *k*-mer manifold from *k* to *k* + 1. Given the success of such a theory, we could investigate the possibilities of projecting the *k* and *k* + 1 *k*-mer manifolds to 2D and 3D jointly, such that we could visualize the transformation process. This will help to address the motif length determination problem. Another direction worth further investigation is how to characterize the *k*-mer manifolds for two different conditions, for example, WT and KO, and how to compare the *k*-mer manifolds and get the differentially expressed Hamming balls. The challenge in motif length estimation means we should not use KMAP to differentiate motifs with subtle changes in the flanking regions, for example, the strong and weak GR motifs of Schöne et al. (2016).

One limitation of KMAP is that genomes do not conform to the uniform *k*-mer manifold hypothesis, which can result in false-positive motifs, particularly repetitive motifs. Although adopting a nonuniform *k*-mer manifold model could help address this issue, it presents both theoretical and computational challenges. A nonuniform manifold invalidates the unimodal property of *k*-mer counts across orbits, requiring computationally intensive numerical calculations. To assist users in interpreting results, KMAP provides auxiliary information, including the *P*-value of the Hamming ball ratio and the proportion of motif-containing reads, which can aid in assessing motif significance. Based on practical experience, repetitive motifs are more likely to be false positives than are nonrepetitive motifs. Additionally, users can examine the relative positional distribution of motifs within input reads, as central positioning is often indicative of true binding sites in ChIP-seq data. For further insights into TF interactions, users may also analyze colocalization patterns of motif pairs within input reads.

In conclusion, KMAP is a powerful sequence data exploration tool that offers several advantages for motif analysis. Its *k*-mer manifold theory enhances our understanding of *k*-mer count distributions across different orbits and guides the selection of an appropriate Hamming ball radius to identify similar *k*-mers. Unlike PWMs, KMAP identifies sequence-level motifs that are easier to interpret and locate on a reference sequence. For instance, locating a consensus sequence with up to two mutations on the reference sequence is more straightforward than evaluating PWM-derived *P*-values. Additionally, KMAP provides an intuitive 2D visualization of the *k*-mer manifold, displaying the relative strengths of identified motifs and enabling efficient detection of sequence patterns across diverse data sets.

## Methods

### KMAP analysis workflow

The following workflow was used both for HT-SELEX and EWS data analysis. For KMAP motif discovery, we set the following default parameters: *k* = 5, 6, …, 16; the ratio threshold of significance was 1 × 10^−10^; and the reverse-complement mode was on. Results of different *k*-mer lengths were merged to generate the final motifs (Hamming ball), each of which could have a different length. KMAP motif discovery also provided the co-occurrence matrix of the motifs. For visualization, 5000 data points were sampled (supervised sampling). For KMAP dimensionality reduction, we used 20 neighbors for distance smoothing, set the learning rate to 0.01, and used 2500 iterations for the optimization. The outcome of KMAP dimensionality reduction was the 2D embeddings of input *k*-mers.

The motif discovery algorithm begins by counting *k*-mers for a user-defined range of *k*-mer lengths. For each *k*-mer length, the algorithm tests the Hamming ball ratios of the 10 most frequent *k*-mers in the input FASTA file. Among these, the *k*-mer with the lowest *P*-value is selected, and its Hamming ball is then masked from the input file to prevent redundant discoveries. This process is iterated up to 10 times or until no more *k*-mers pass the significance test. The detailed steps of this algorithm are outlined in Algorithm 1 in Supplemental Note S2.

In the final step, a list of candidate consensus sequences for various *k*-mer lengths is generated. To determine the final motifs, sequences that appear in the candidate list for three consecutive *k*-mer lengths, in which each shorter sequence is a substring of the next, are selected. For instance, if the candidate list includes AATCGTAGGA (10-mer), AATCGTAGGAT (11-mer), and AATCGTAGGATG (12-mer), then AATCGTAGGAT would be chosen as the final motif. This consensus sequence merging algorithm is explained in detail in Algorithm 3 in Supplemental Note S2.

*k*-mers in the motif (Hamming ball) were used for PWM calculation and logo generation, by “sites2meme” and “ceqlogo” commands in MEME suite (version = 5.0.5) (Bailey et al. 2015), as well as LogoMaker (version = 0.8) (Tareen and Kinney 2020).

### HT-SELEX data analysis

The data was downloaded from the European Nucleotide Archive (ENA; https://www.ebi.ac.uk/ena/browser/home) under accession number ERP001824. We selected 1273 samples of SELEX cycles 3 to 6 for the benchmark comparison of KMAP and MEME. We limited the motif length to range from five to 16 and only picked the top motif in the comparison both for KMAP and MEME. Single-nucleotide repetitive motifs such as “AAAAA” or “CCCCC” were removed from KMAP output. Exact match is used to identify the overlap between KMAP and MEME consensus sequences.

The following samples are used for the illustration of Figure 1A: BHLHE40 (cycle 4, 137,919 sequences), MAFK (cycle 6, 662,800 sequences), MEF2D (cycle 4, 580,243 sequences), and NFKB2 (cycle 5, 132,941 sequences). Default parameters were used in DREME (Bailey 2011) and STREME (Bailey 2021) analyses. The motif length was set to range from five to 16, and the strongest motif is selected for illustration.

The scikit-learn package (version = 1.3.0) (Pedregosa et al. 2011) was used for PCA, MDS, t-SNE, and UMAP analysis, and default parameters were used. *k*-mers were converted to one-hot vectors to be used as the input of PCA. The Hamming distance matrix of input *k*-mers was used as the input for MDS, t-SNE, and UMAP.

### EWS data analysis

All data were downloaded from the NCBI Gene Expression Omnibus (GEO; https://www.ncbi.nlm.nih.gov/geo/) under accession number GSE181554. A detailed list of files and the meta information, as well as their names shown in the figures, are provided in Supplemental Table S1.

For ChIP-seq/cut-tag analysis, Sequence Read Archive (SRA; https://www.ncbi.nlm.nih.gov/sra) files were converted to FASTQ files using the “fastq-dump” command in SRAtools (version 3.0.5). Reads were aligned to the human genome GRCh37 (hg19) using Bowtie 2 (version = 2.5.1) (Quinlan and Hall 2010) using the “—very sensitive” preset collection of parameters. SAMtools (version = 1.6) (Danecek et al. 2021) was used to convert BAM files into SAM files. Compared with hg19, GRCh38 (hg38) provides a more complete representation of complex genomic regions such as repetitive elements, segmental duplications, and centromeres. However, our ChIP-seq/cut-tag analysis primarily involves uniquely mapped reads, which are largely unaffected by these improvements. Therefore, realignment to GRCh38 would result in negligible differences. ChIP-seq peaks were called using MACS2 (version = 2.2.7.1) (Zhang et al. 2008) with default parameters by comparing with the corresponding input DNA control. The derived peaks (BED files) were converted to a FASTA file using BEDTools (version = 2.31.0) (Quinlan and Hall 2010).

The differential peaks (H3K27ac) were derived by subtracting peaks of the ETV6 intact sample from that of ETV6 degraded sample, using the “subtract” command of BEDTools. Promoter regions were identified as (−2000 bp, +200 bp) of transcription start sites, whereas other regions were treated as enhancer regions. Peaks were classified into these two categories using the “annotatePeak” function from the ChIPseeker R package (Yu et al. 2015), which used the annotation file of hg19 human genome from the “TxDb.Hsapiens.UCSC.hg19.knownGene” package (https://bioconductor.org/packages/release/data/annotation/html/TxDb.Hsapiens.UCSC.hg19.knownGene.html). The corresponding DNA sequences on the genome of these peaks were fed to KMAP analysis. We deleted motifs that were full of A's in the KMAP results, which were presumed to be noise.

Given the consensus sequences of KMAP motifs, FIMO in MEME suite was used to identify the TFs of the motifs from JASPAR database (Castro-Mondragon et al. 2022), with default *P*-value threshold of 1 × 10^−^^4^. The expression levels of all genes (TPM column from the quantified gene expression file in the original publication; GEO accession number GSM5505965) were ranked for each sample to generate the relative expression (0%–100%) for all relevant TFs. Rank 1 is assigned a value of 100%, and the lowest rank is assigned a value of 0%. This transformation linearly converts each gene's rank into a percentage scale, with higher ranks corresponding to higher percentages. For each sample, a gene is treated as a high-expressing gene if its relative expression is >75%.

To derive the co-occurrence matrix of different motifs, we scan for all motif consensus sequences on each input read and then record the co-occurrence of all identified motif pairs. We get the co-occurrence count matrix after scanning all reads. For each pair of motifs, we normalized its co-occurrence count by dividing the number of reads that contain either motif. The normalized co-occurrence matrix is fed to NetworkX (version = 2.2) (Hagberg et al. 2008) to generate the co-occurrence network.

Note that we use the gene symbol *KCNH2* for *ERG1* (FIMO's output in EWS data) and the gene symbol *BHLHE40* for *BHLHB2* (SELEX data), according to the nomenclatures given by the HUGO gene nomenclature committee (https://www.genenames.org/).

### Gene editing data analysis

The FASTQ file, Protospacer sequence, and reference sequence were obtained from the CRISPR-A website (https://synbio.upf.edu/crispr-a/documentation.html#fourth, access date 7.2.2024): reference sequence, GCTCCAGGAAATGGGGGTGTGTCACCAGATAAGGAATCTGCCTAACAGGAGGTGGGGGTTAGACCCAATATCAGGAGACTAGGAAGGAGGAGGCCTAAGGATGGGGCTTTTCTGTCACCAATCCTGTCCCTAGTGGCCCCACTGTGGGGTGGAGGGGACAGATAAAAGTACCCAGAACCAGAGCCACATTAACCGGCCCTGGGAATATAAGGTGGTCCCAGCTCGGGGACACAGGATCCCTGGAGGCAGCAAACATGCTGTCCT, and protospacer, GGGGCCACTAGGGACAGGAT.

Filtered reads were aligned using MUSCLE (version 5) (Edgar 2004) with the “super5” command. Multiple sequence alignment was visualized using ggmsa (version = 1.4.0) package (Zhou et al. 2022). DBSCAN (Ester et al. 1996) in scikit-learn (version = 1.3.0) (Pedregosa et al. 2011) was used to identify clusters from 2D embeddings, with parameters *eps = 3* and *min_sample = 200*.

Because there are only about 20,000 reads and the read lengths are too long to be treated as a *k*-mer (*k* = 250), we performed unsupervised sampling (Supplemental Note S2, Algorithm 2) on the data. We calculated the pairwise Hamming distance matrix between the aligned reads, from which we derived the smoothed distance matrix by taking the average of 600 nearest neighbors. We then fitted a Gaussian distribution to smoothed distances, which was given by *N*(46.5, 14.9^2^). We kept distances that were less than μ − 2σ = 15.56, which presumably correspond to motif *k*-mers/sequences. Sequences involved in the filtered distances were the output of the unsupervised sampling. Hamming distance matrix of the sampled DNA sequences was directly used in the dimensionality reduction.

### Software availability

The KMAP software and analysis scripts for reproducing the work are uploaded as Supplemental Code. The KMAP package is freely available at GitHub (https://github.com/chengl7-lab/kmap). Analysis scripts are deposited at GitHub (https://github.com/Dionysos-o/kmap/tree/main/kmap_paper). KMAP motif logos and 2D visualization plots are available at Zenodo (https://doi.org/10.5281/zenodo.14046858) and as Supplemental Data S1.

## Supplemental Material

Supplement 1

Supplement 2

Supplement 3

Supplement 4

Supplement 5

Supplement 6

Supplement 7

Supplement 8
