# Supplementary material for: k-mer manifold approximation and projection for visualizing DNA sequences
Source: Genome Res. 2025 May;35(5):1234–46. doi: 10.1101/gr.279458.124 (PMC12047656; doi:10.1101/gr.279458.124)
Supplement: Supplement 1 [file Supplemental_Figures.pdf]

A

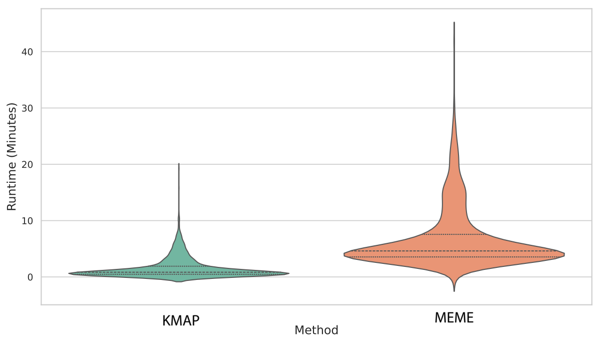

B

Input file size	Time
1MB	1min
10MB	5min
100MB	38min
1000MB	5h22min

Supplemental Figure S1: Runtime benchmark of KMAP vs MEME. (A) Runtime of KMAP and MEME on 1273 HT-SELEX datasets. Note that only the motif discovery portion of KMAP is measured here. (B) Typical running time of KMAP for various input file sizes on a dual Intel Xeon E5-2680 v3 2.50 GHz CPU, using a single core and 10 GB of memory.

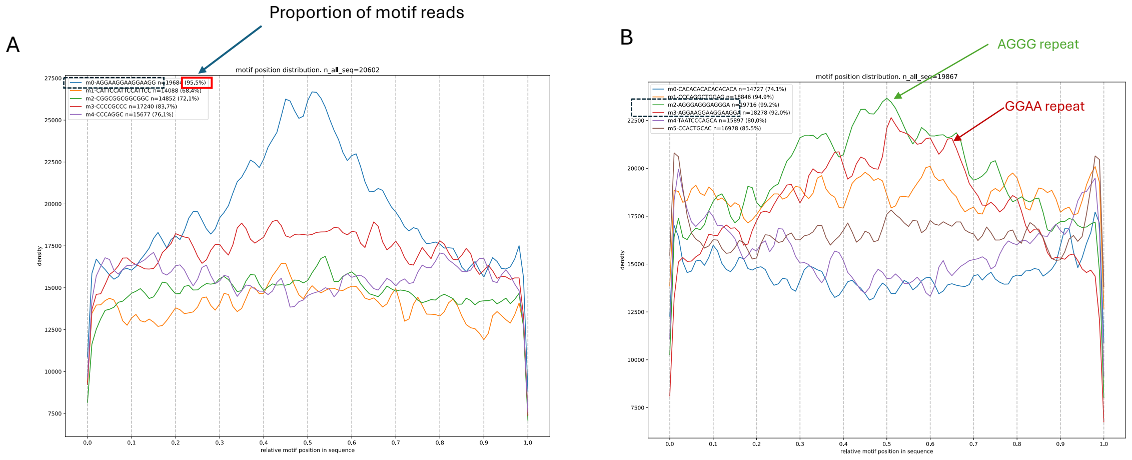

FLI1 ChIP-seq data
(A673 dTAG cell, dTAGv−1 treatment, ETV6 KO)

ETV6 ChIP-seq data
(A673 Parental cell, ETV6 WT)

Supplemental Figure S2: Relative position distributions of motifs.
(A) FLI1 ChIP-seq data: The GGAA-repeat motif shows a preference for central positions. Percentages indicate the proportion of input reads containing the given motif.
(B) ETV6 ChIP-seq data: Both the GGAA-repeats and AGGG-repeats display a preference for central positioning.

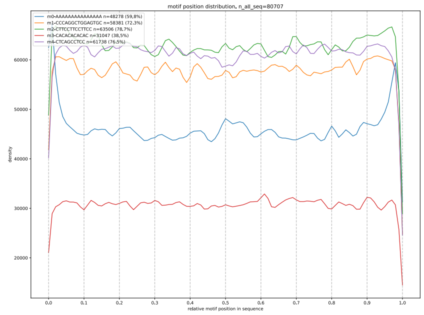

A673_dTAG_pair1

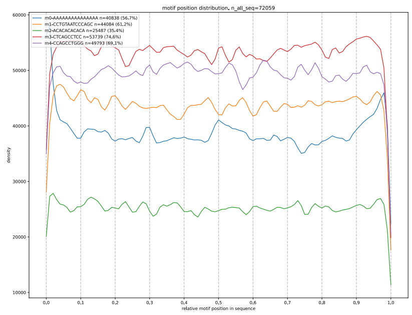

A673_dTAG_pair2

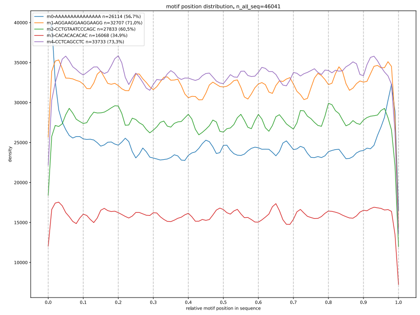

EW8_dTAG_pair1

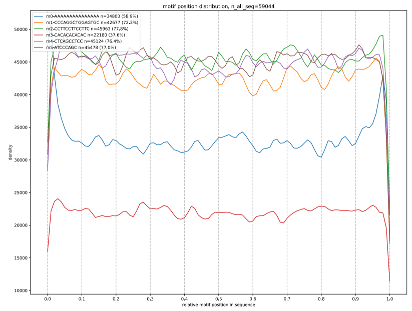

EW8_dTAG_pair2

Supplemental Figure S3: Relative position distribution of motifs on enhancer regions derived from four pairs.

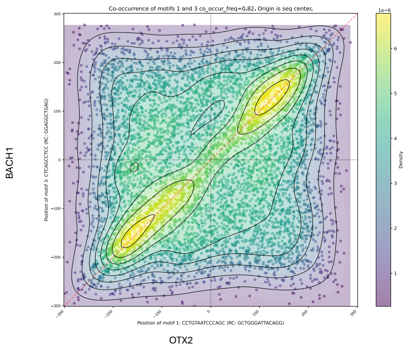

A673_dTAG_pair2

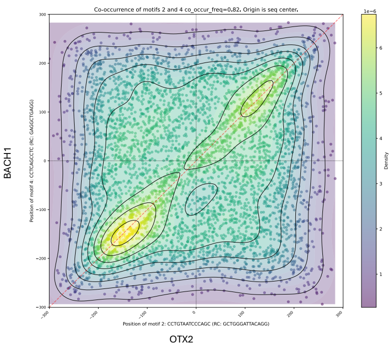

EW8_dTAG_pair1

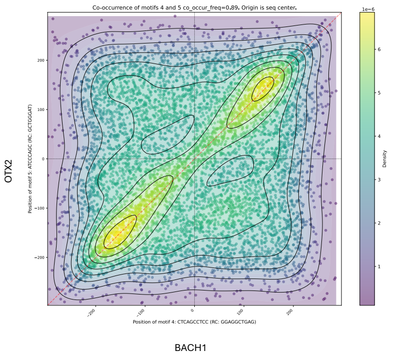

EW8_dTAG_pair2

Supplemental Figure S4: Motif positions of OTX2 and BACH1 on enhancer regions (400–600 bp). Each dot represents a pair of OTX2 and BACH1 occurrences within a single enhancer region. The center of each enhancer region is set as the origin, and motif positions are shown relative to this origin. The OTX2 motif was not found in the enhancers of “A673_dTAG_pair1.”

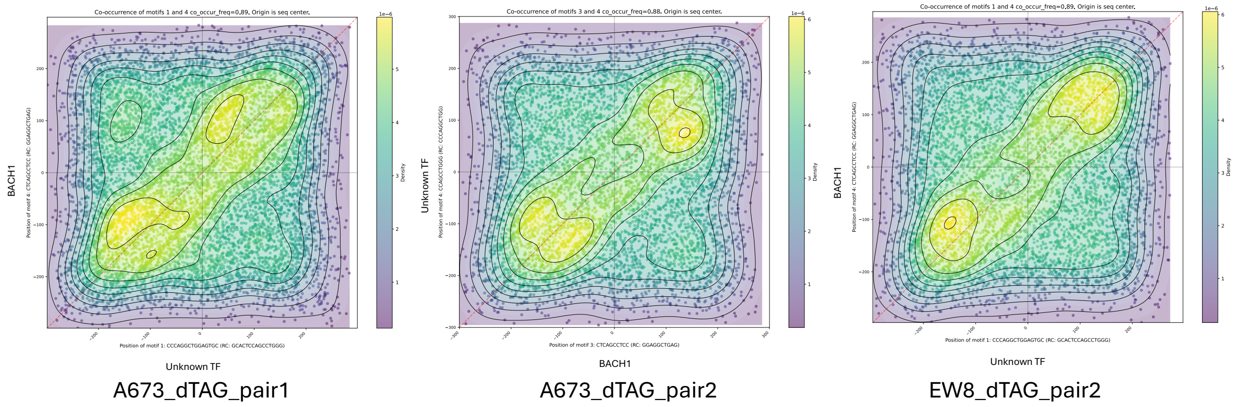

Supplemental Figure S5: Motif positions of CCCAGGCTGGAGTGC and BACH1 on enhancer regions (400–600 bp). Each dot represents a pair of occurrences of the CCCAGGCTGGAGTGC motif and BACH1 within a single enhancer region. The center of each enhancer region is set as the origin, and motif positions are shown relative to this origin. The CCCAGGCTGGAGTGC motif was not found in the enhancers of "EW8_dTAG_pair1."

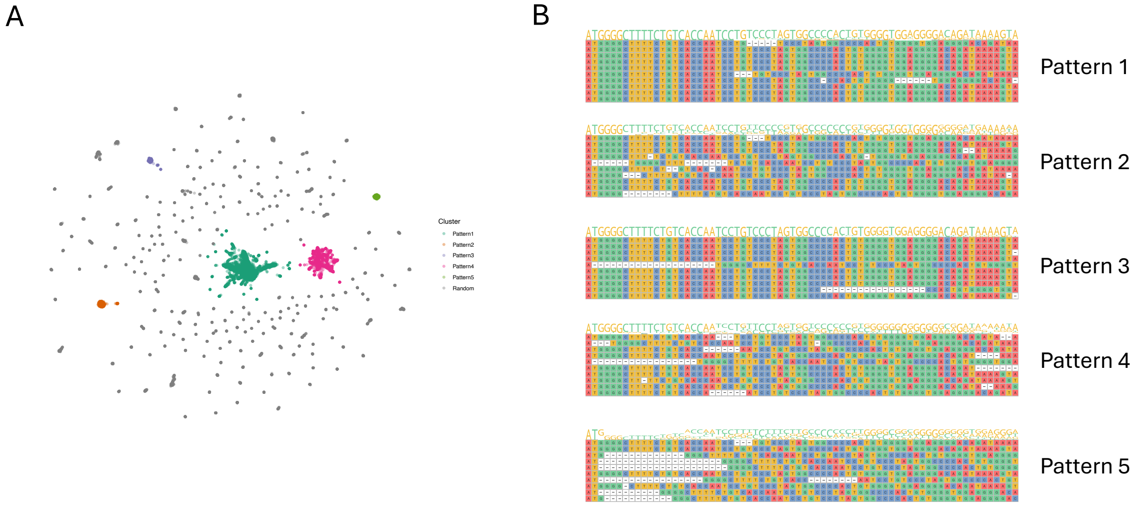

Supplemental Figure S6: Gene editing data analysis with UMAP. (A) UMAP plot of gene editing data showing patterns detected by DBSCAN. (B) Aligned reads for each detected pattern.
