# Supplementary material for: k-mer manifold approximation and projection for visualizing DNA sequences
Source: Genome Res. 2025 May;35(5):1234–46. doi: 10.1101/gr.279458.124 (PMC12047656; doi:10.1101/gr.279458.124)
Supplement: Supplement 2 [file Supplemental_Note_S1.pdf]

k-mer manifold theory

(Supplementary Note 1)

Chengbo Fu, Lu Cheng

February 7, 2025

Contents

1 Introduction **1**

2 k-mer space **2**

3 k-mer manifold **3**

4 Uniform distribution hypothesis **4**

5 Hamming ball **7**

6 Properties of k-mer manifold **10**

6.1 Hamming distance properties 11

6.2 Hash key partition of k-mer manifold 12

1 Introduction

DNA sequences are the main carrier of genetic information. It is of vital importance to set up a mathematical system to study DNA sequences. This document tries to establish a proper notation system to characterize k-mer space and its properties.

Consider a DNA sequence $\boldsymbol{z} = z_1 z_2 \cdots z_i \cdots z_N$ $(z_i \in \{A, C, G, T\})$, we can convert it to a list of k-mers $X = (\boldsymbol{x}_1, \boldsymbol{x}_2, \cdots, \boldsymbol{x}_i, \cdots, \boldsymbol{x}_{N-k+1})$ by a sliding window of length k from position 1 to $N-k+1$, where the ith k-mer is given by $\boldsymbol{x}_i = z_i z_{i+1} \cdots z_{i+k-1}$. We are interested in a random DNA sequence $\boldsymbol{z}$ of infinite length N. There should be 4^k different k-mers in X, since each site can take 4 possibilities. Due to the randomness, the amount of each k-mer should be the same for all unique k-mers in X.

Here we only consider the unique k-mers of length k and denote its domain as $S^{(k)}$, where $|S^{(k)}| = 4^k$. For a random DNA sequence, the distribution over the unique k-mers $S^{(k)}$ should be uniform. As k increases, the size of the k-mer domain $|S^{(k)}|$ grows exponentially as 4^k. Therefore, the maximum length of k-mer is set to 31 ($4^{31} = 2^{62}$) in practice. By converting to a list of k-mers, a given DNA sequence can be approximately characterized the derived unique k-mers and their counts. The same DNA sequence can be characterized in k-mer spaces of different lengths, e.g. k and $k+1$. The k-mer distributions in these two spaces are related. However, the relationship is so complex that we cannot provide analytic solutions.

We are interested in studying the structure of k-mer space with a uniform distribution over k-mers. We can take any k-mer as the **origin** of the k-mer space. For any other other k-mer, we calculate the Hamming distance between the two k-mers, which is the total number of sites with different nucleotides. The origin and Hamming distance jointly determine the structure of the k-mer space, which we call the **k-mer manifold**. The k-mer manifold is symmetric in the sense we can generate the whole k-mer space by centering on any k-mer (as the origin).

Since the Hamming distance is discrete, the k-mer manifold can be partitioned into $k+1$ orbits, where k-mers in a orbit have the same Hamming distance to the the origin. By introducing a radius, we define a **Hamming Ball** centered on the origin as the set of k-mers with a Hamming distance

less than or equal to the given radius. The Hamming Ball represents all k-mers that is similar to the origin. In the context of motif discovery, the origin refers to the consensus sequence.

In practice, the DNA is double stranded, which contains both the positive strand and negative strand. The sequence on the positive strand is the reverse complement of the sequence on the negative strand, and *vice versa*. It is common that the data contain sequences of both strands. Consequently, two different k-mers (reverse complements) can represent the same DNA fragment. Here we compute an unique hash key (integer) for each k-mer, which is used to compare different k-mers. To save storage space in practice, we only keep the k-mer with a smaller hash key for each pair of k-mers (a k-mer and its reverse complement). As a result, the k-mer space can be partitioned into two hemispheres based on the comparison of the hash keys. k-mers with smaller or equal hash keys than their reverse complements are assigned to the positive hemisphere while others fall into the negative hemisphere.

Since the manifold orbit partition and hash key partition are based on different principles, special treatments are taken to harmonize the restrictions imposed by the two different partitions.

The theories built in this document serves as the foundation for the motif discovery algorithm and k-mer visualization algorithm in Supplementary Note 2 and 3.

2 k-mer space

This section provides the notations of the k-mer space. For a given length k, a k-mer is k consecutive nucleotides. There are 4^k unique k-mers in total. The **k-mer space** is define as $S^{(k)} = \{\boldsymbol{s}_1^{(k)}, \boldsymbol{s}_2^{(k)}, \cdots, \boldsymbol{s}_i^{(k)}, \cdots \boldsymbol{s}_{N^{(k)}}^{(k)}\}$, where $N^{(k)} = |S| = 4^k$ is the total number of k-mers in the k-mer space. The ith k-mer is defined as $\boldsymbol{s}_i^{(k)} = s_{i1}^{(k)} s_{i2}^{(k)} \cdots s_{ij}^{(k)} \cdots s_{ik}^{(k)}$, where $s_{ij}^{(k)} \in \{A, C, G, T\}$. Note that $\boldsymbol{s}_i^{(k)} \neq \boldsymbol{s}_j^{(k)}$ for any $i \neq j$.

We define the k-mer hash function $h(\cdot)$ of a k-mer $\boldsymbol{s}_i^{(k)}$ by

$$h(\boldsymbol{s}_i^{(k)}) = \sum_{j=1}^{k} g(s_{ij}^{(k)}) 4^{k-j}, \tag{1}$$

where $g(\cdot)$ is given by

$$g(s) = \begin{cases} 0 & if\ s = A \\ 1 & if\ s = C \\ 2 & if\ s = G \\ 3 & if\ s = T \end{cases} \tag{2}$$

The hash of a k-mer is an integer between 0 and $4^k - 1$. For example, let $k = 3$ and the hash of ACT is give by

$$\begin{aligned} h(ACT) &= g(A)4^{3-1} + g(C)4^{3-2} + g(T)4^{3-3} \\ &= 0 \times 4^2 + 1 \times 4^1 + 3 \times 4^0 \\ &= 0 + 4 + 3 \\ &= 7 \end{aligned}$$

It can be seen that $0 < 7 < 4^3 - 1 = 63$.

We define the complement function by

$$f(s) = \begin{cases} T & if\ s = A \\ G & if\ s = C \\ C & if\ s = G \\ A & if\ s = T \end{cases} \tag{3}$$

The reverse complement function for $\boldsymbol{s}_i^{(k)}$ is given by

$$\begin{aligned} rc(\boldsymbol{s}_i^{(k)}) &= rc(s_{i1}^{(k)} s_{i2}^{(k)} \cdots s_{ij}^{(k)} \cdots s_{ik}^{(k)}) \\ &= f(s_{ik}^{(k)}) \cdots f(s_{ij}^{(k)}) \cdots f(s_{i2}^{(k)}) f(s_{i1}^{(k)}) \end{aligned}$$

As an illustration, the reverse complement of the k-mer ACT is given by

$$rc(ACT) = f(T)f(C)f(A) = AGT$$

For simplicity, we allow reverse complement function to operate on a list of k-mers. Given a k-mer list $X = (\boldsymbol{x}_1^{(k)}, \boldsymbol{x}_2^{(k)}, \cdots, \boldsymbol{x}_i^{(k)}, \cdots \boldsymbol{x}_N^{(k)})$, where $\boldsymbol{x}_i^{(k)} \in S^{(k)}$. The reverse complement of the k-mer list X is also a k-mer list given by

$$rc(X) = (rc(\boldsymbol{x}_1^{(k)}), rc(\boldsymbol{x}_2^{(k)}), \cdots, rc(\boldsymbol{x}_i^{(k)}), \cdots, rc(\boldsymbol{x}_N^{(k)})) \tag{4}$$

3 k-mer manifold

To study the topology of the k-mer space, we introduce the origin and metric to set up the k-mer manifold on the k-mer space. We could choose any k-mer $\boldsymbol{s}_i^{(k)} \in S^{(k)}$ as the origin denoted by $\boldsymbol{o}^{(k)}$. We use the Hamming distance as the metric. The hamming distance between $\boldsymbol{s}_i^{(k)}$ and $\boldsymbol{s}_j^{(k)}$ is defined as

$$d(\boldsymbol{s}_i^{(k)}, \boldsymbol{s}_j^{(k)}) = \sum_{p=1}^{k} \mathbb{1}_{s_{ip}^{(k)} \neq s_{jp}^{(k)}} \tag{5}$$

, where $\mathbb{1}$ is the indicator function. The indicator function returns 1 if the condition is true, otherwise returns 0.

It is obvious that Hamming distance is a valid metric function $d : S^{(k)} \times S^{(k)} \rightarrow \mathcal{N}$ that satisfies the following properties.

- The distance from a k-mer to itself is 0, i.e. $d(\boldsymbol{s}_i^{(k)}, \boldsymbol{s}_i^{(k)}) = 0$
- (Positivity) The distance between two distinct k-mers is always positive, $d(\boldsymbol{s}_i^{(k)}, \boldsymbol{s}_j^{(k)}) > 0$
- (Symmetry) The distance from $\boldsymbol{s}_i^{(k)}$ to $\boldsymbol{s}_j^{(k)}$ is always the same as the distance from $\boldsymbol{s}_j^{(k)}$ to $\boldsymbol{s}_i^{(k)}$, i.e. $d(\boldsymbol{s}_i^{(k)}, \boldsymbol{s}_j^{(k)}) = d(\boldsymbol{s}_j^{(k)}, \boldsymbol{s}_i^{(k)})$
- The triangle inequality holds $d(\boldsymbol{s}_i^{(k)}, \boldsymbol{s}_j^{(k)}) \leq d(\boldsymbol{s}_i^{(k)}, \boldsymbol{s}_l^{(k)}) + d(\boldsymbol{s}_j^{(k)}, \boldsymbol{s}_l^{(k)})$, where $i \neq j \neq l$. Here we consider a position p that $\boldsymbol{s}_i^{(k)}$ differs with $\boldsymbol{s}_j^{(k)}$, i.e. $s_{ip}^{(k)} \neq s_{jp}^{(k)}$, it must be true that either $s_{ip}^{(k)}$ or $s_{jp}^{(k)}$ is not equal to $s_{lp}^{(k)}$, which hints the sum of $d(s_{ip}^{(k)}, s_{lp}^{(k)})$ and $d(s_{jp}^{(k)}, s_{lp}^{(k)})$ is greater than or equal to $d(s_{ip}^{(k)}, s_{jp}^{(k)}) = 1$.

Now we compute the Hamming distance for each k-mer in the k-mer space to the origin $d(\boldsymbol{s}_i^{(k)}, \boldsymbol{o}^{(k)})$, which takes discrete values from $\{0, 1, 2, \cdots, k\}$. As a result, the k-mers can be assigned to $k+1$ orbits, denoted by $\mathcal{A}_0^{(k)}, \mathcal{A}_1^{(k)}, \cdots, \mathcal{A}_k^{(k)}$. The ith orbit is given by

$$\mathcal{A}_i^{(k)} = \{\boldsymbol{s}^{(k)} \mid \forall d(\boldsymbol{s}^{(k)}, \boldsymbol{o}^{(k)}) = i \;\&\; \boldsymbol{s}^{(k)} \in S\}, \tag{6}$$

The Hamming distance from any k-mer in pth orbit $\mathcal{A}_p^{(k)}$ to the origin $\boldsymbol{o}^{(k)}$ is the same, i.e.

$$d(\boldsymbol{s}_i^{(k)}, \boldsymbol{o}^{(k)}) = d(\boldsymbol{s}_j^{(k)}, \boldsymbol{o}^{(k)}) = p \quad \forall \boldsymbol{s}_i^{(k)}, \boldsymbol{s}_j^{(k)} \in \mathcal{A}_p^{(k)} \;\&\; i \neq j \tag{7}$$

The ***k*-mer manifold** is defined as a union of the orbits

$$\Omega^{(k)} = \cup_{i=0}^{k} \mathcal{A}_i^{(k)}, \tag{8}$$

where $\mathcal{A}_i^{(k)} = \{\boldsymbol{s}^{(k)} \mid \forall d(\boldsymbol{s}^{(k)}, \boldsymbol{o}^{(k)}) = i \;\&\; \boldsymbol{s}^{(k)} \in S\}$ and $\mathcal{A}_i^{(k)} \cap \mathcal{A}_j^{(k)} = \emptyset \; \forall i \neq j$.

The k-mer manifold has a isotropic property given by the following remark. The k-mer space is always the same no matter which k-mer is selected as the origin of the k-mer manifold.

Remark 0.1. *For any origin $\boldsymbol{o}^{(k)}$, the k-mer manifold $\Omega^{(k)}$ corresponds to the same k-mer space $S^{(k)}$.*

Assume we have two k-mer manifolds $\Omega_A^{(k)}, \Omega_B^{(k)}$centered on origins $\boldsymbol{o}_A^{(k)}$ and $\boldsymbol{o}_B^{(k)}$. For any k-mer $\boldsymbol{s}_i^{(k)} \in \Omega_A^{(k)}$, the Hamming distance to the $\boldsymbol{o}_B^{(k)}$ is given by $d(\boldsymbol{s}_i^{(k)}, \boldsymbol{o}_B^{(k)}) \in \{0, 1, 2, \cdots, k\}$. Therefore, $\boldsymbol{s}_i^{(k)}$ belongs to one orbit of manifold B, i.e. $\boldsymbol{s}_i^{(k)} \in \Omega_B^{(k)}$. Similarly, for any k-mer $\boldsymbol{s}_j^{(k)} \in \Omega_B^{(k)}$, we have $\boldsymbol{s}_j^{(k)} \in \Omega_A^{(k)}$. Therefore, there exists a bijective map between $\Omega_A^{(k)}$ and $\Omega_B^{(k)}$ and both manifolds corresponds to the same k-mer space $S^{(k)}$.

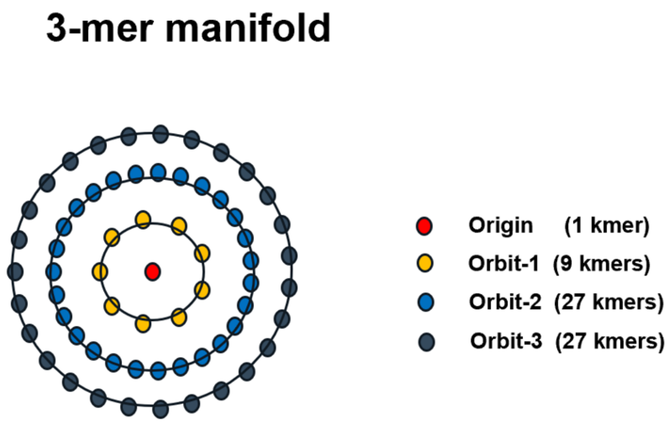

Figure 1: Orbits of k-mer manifold for $k = 3$. The number of k-mers on each orbit is shown in the legend.

4 Uniform distribution hypothesis

We are interested in studying the k-mer manifold of a random DNA sequence. Due to the randomness, the distribution over the unique k-mers should be uniform, i.e. each k-mer in the k-mer space has the same probability.

Since we have defined the k-mer manifold by introducing an origin and Hamming distance, we are interested in deriving the probability mass function for each orbit given the uniform k-mer distribution hypothesis. Here we assume the occurrence of each k-mer is exactly 1 in the random DNA sequence. The count of orbit i is given by

$$|\mathcal{A}_i^{(k)}| = C_k^i * (4-1)^i, \tag{9}$$

where $C_k^i = \binom{k}{i} = \frac{k!}{i!(k-i)!}$ is the binomial coefficient. As shown in Fig. 1, there are 4 orbits for $k = 3$, where the origin ($\mathcal{A}_0^{(k)}$) can also be considered as an orbit.

Here we show the derivation of the counts from a special case where $k = 3$ and AGT is set as the origin. It is obvious to see that the number of k-mers in orbit 0 is 1, which equals to $|\mathcal{A}_i^{(k)}| = C_3^0 * (4-1)^0 = 1$. To jump from the origin to orbit 1, we introduce a mutation to the pth position ($p \in \{1, 2, 3\}$) of the origin, i.e. change $o_p^{(k)}$ to a different nucleotide. There are $C_3^1 C_3^1$ possibilities, where the first 3 refers to the position and the second 3 refers to the possible mutated nucleotides. There are $C_3^1 * (4-1)^1 = 9$ k-mers in orbit 1:

- (1st position mutation): GGT, CGT, TGT
- (2nd position mutation): AAT, ACT, ATT
- (3rd position mutation): AGA, AGG, AGC

Similarly, we could introduce mutations at pth and qth positions ($p, q \in \{1, 2, 3\}$, $p \neq q$) on the origin to generate k-mers of orbit 2. First we need to select 2 positions, where there are C_3^2 possibilities. Then we need to introduce mutations to both positions, where there are 3^2 possibilities. So the total number of k-mers in orbit 2 is given by $C_3^2 \cdot 3^2 = 27$.

The total number of k-mers in orbit 3 is given by $C_3^3 \cdot 3^3 = 27$. Summing k-mers of all orbits, we

have

$$\sum_{i=0}^{3} |\mathcal{A}_i^{(3)}| = C_3^0 * (4-1)^0 + C_3^1 * (4-1)^1 + C_3^2 * (4-1)^2 + C_3^3 * (4-1)^3$$
$$= 1 + 9 + 27 + 27$$
$$= 64 = 4^3$$

We could generalize the above counting process to a larger k, where the sum of k-mer counts of different orbits is given by

$$\sum_{i=0}^{k} |\mathcal{A}_i^{(k)}| = C_k^0 + C_k^1 * (4-1) + C_k^2 * (4-1)(4-1) + ... + C_k^i * (4-1)^i + ... + C_k^k * (4-1)^k$$
$$= (3+1)^k$$

It is obvious to notice that the formula above is the binomial expansion of $(3+1)^k$, where each term in the expansion corresponds to the k-mer count of an orbit. Fig. 2 shows the k-mer count distribution over different orbits for $k = 5, 6, \cdots, 20$.

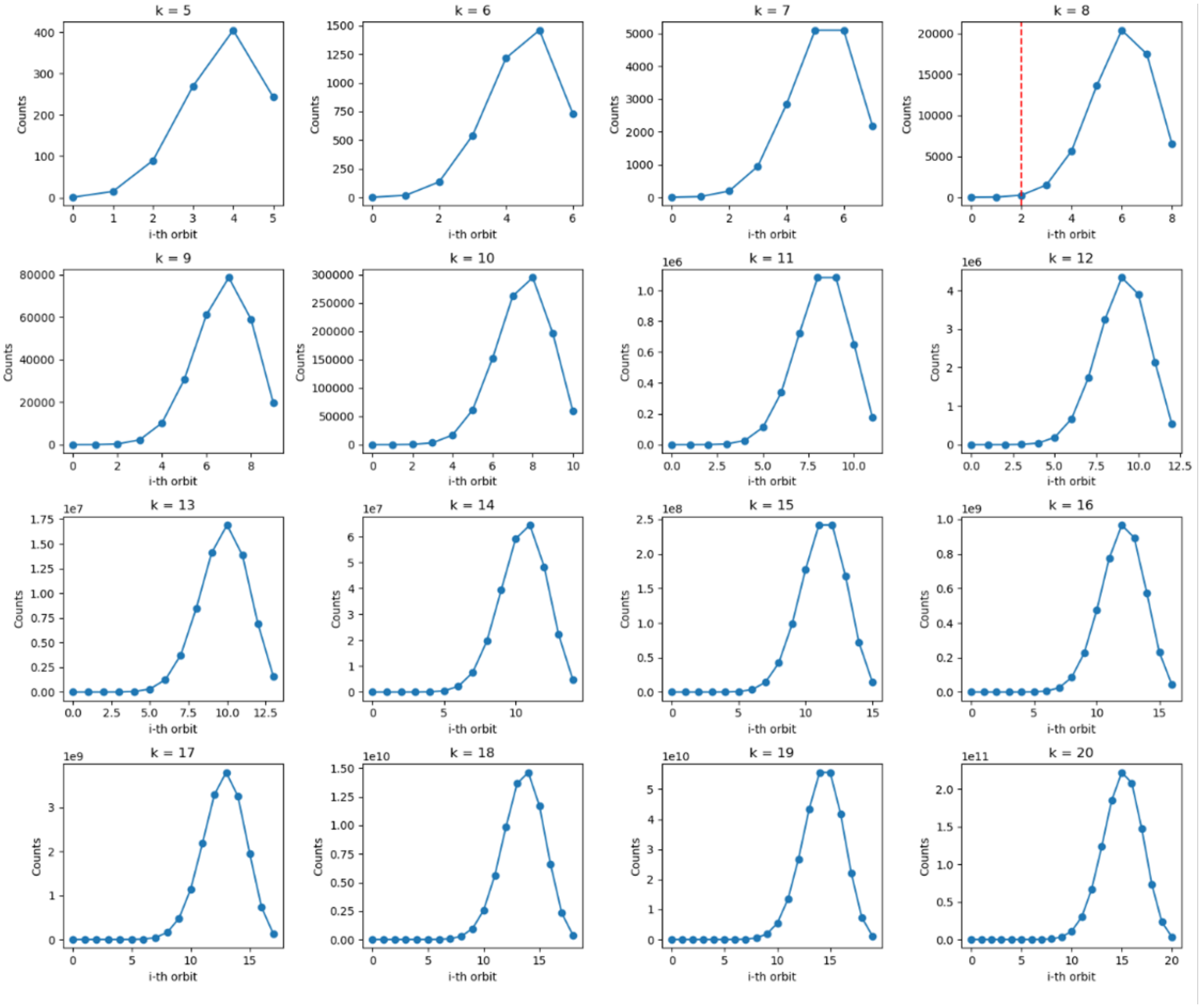

Figure 2: k-mer counts on different orbits in k-mer manifold for $k = \{5 \cdots 20\}$

The probability mass function (pmf) of ith orbit is given by

$$p(\mathcal{A}_i^{(k)}) = \frac{|\mathcal{A}_i^{(k)}|}{|\Omega^{(k)}|} = \frac{C_k^i * (4-1)^i}{4^k} \tag{10}$$

Fig. 2 shows that the pmf of the orbits is unimode for $k = 5, 6, \cdots, 20$. It can be proved that this unimode property holds for $k > 4, k \in \mathcal{N}$, as illustrated by the following theorem.

Theorem 1. *The k-mer count of orbit i* $|\mathcal{A}_i^{(k)}| = C_k^i * (4-1)^i$ *is a unimode function w.r.t i for* $k > 4, k \in \mathcal{N}$.

Proof. Note $|\mathcal{A}_i^{(k)}| = C_k^i * (4-1)^i = 3^i \binom{k}{i}$ and consider the following definitions of Gamma function and Digamma function.

$$\left(\begin{array}{c} k \\ i \end{array} \right) = \frac{\Gamma(k+1)}{\Gamma(i+1)\Gamma(k-i+1)}$$

$$\psi(i) = \frac{\Gamma(i)'}{\Gamma(i)}$$

We have

$$\frac{d}{di}\Gamma(i+1) = \Gamma(i+1)\psi(i+1)$$
$$\frac{d}{di}\Gamma(k-i+1) = -\Gamma(k-i+1)\psi(k-i+1)$$

The gradient of $|\mathcal{A}_i^{(k)}|$ w.r.t. i is given by

$$\frac{d}{di}\left(3^i \left(\begin{array}{c} k \\ i \end{array} \right)\right) = 3^i \ln(3)\frac{\Gamma(k+1)}{\Gamma(i+1)\Gamma(k-i+1)} + 3^i(\frac{\Gamma(k+1)}{\Gamma(i+1)\Gamma(k-i+1)})'$$
$$= 3^i \ln(3)\frac{\Gamma(k+1)}{\Gamma(i+1)\Gamma(k-i+1)}$$
$$+ 3^i(\frac{0 \cdot \Gamma(i+1)\Gamma(k-i+1) - \Gamma(k+1)\{\Gamma(i+1)\psi(i+1)\Gamma(k-i+1) - \Gamma(k-i+1)\psi(k-i+1)\Gamma(i+1)\}}{(\Gamma(i+1)\Gamma(k-i+1))^2})$$
$$= 3^i \ln(3)\frac{\Gamma(k+1)}{\Gamma(i+1)\Gamma(k-i+1)} - 3^i\frac{\Gamma(k+1)\cdot\psi(i+1)}{\Gamma(i+1)\Gamma(k-i+1)} + 3^i\frac{\Gamma(k+1)\cdot\psi(k-i+1)}{\Gamma(i+1)\Gamma(k-i+1)}$$
$$= 3^i(-\psi(i+1) + \psi(k-i+1) + \ln(3))\frac{\Gamma(k+1)}{\Gamma(i+1)\Gamma(k-i+1)}$$

To show $|\mathcal{A}_i^{(k)}|$ is a unimode function w.r.t. i, we want to prove its gradient is positive on the left of its zero point and negative on the right of it zero point. Since 3^i and $\frac{\Gamma(k+1)}{\Gamma(i+1)\Gamma(k-i+1)}$ are always positive, we only need to consider middle term $g(i) = -\psi(i+1) + \psi(k-i+1) + \ln(3)$.

Here we use a Harmonic series[1] to approximate the $\psi(x)$ by

$$\psi(x) = H_{x-1} - \gamma = \sum_{j=1}^{x}\frac{1}{j} - \gamma, \tag{11}$$

, where γ is the Euler constant $\gamma \approx 0.57721$.

We have

$$\begin{aligned} g(i) =& -\psi(i+1) + \psi(k-i+1) + \ln(3) \\ =& -H_i + \gamma + H_{k-i} - \gamma + ln(3) \\ =& H_{k-i} - H_i + ln(3) \end{aligned}$$

When k is a constant, both $-H_i$ and H_{k-i} are monotonic decreasing functions w.r.t. i. When $i = k$, $g(i)$ gets its minimum value $g_{min} = g(k) = -H_k + ln(3) < 0$. When $i = 0$, $g(i)$ gets its

[1] https://en.wikipedia.org/wiki/Digamma_function

maximum value $g(i)_{max} = g(0) = H(k) + ln(3) > 0$. According to intermediate value theorem and the monotonic property, $g(i)$ has and only has one zero point $i_0 \in [0, k]$, where $|\mathcal{A}_i^{(k)}|$ reaches its maximum value. In the interval $[0, i_0)$, $g(i)$ is positive. In the interval $(i_0, k]$, $g(i)$ is negative. Therefore, $|\mathcal{A}_i^{(k)}|$ is a unimode function w.r.t. i. □

According to Theorem 1, we approximate the unimode pmf using a Gaussian distribution. It can be seen from Fig. 2 that majority of the k-mers are located in the outer orbits that are distant to the origin. Central orbits that contains similar k-mers to the origin have low pmf. Under the uniform distribution hypothesis, majority (random) k-mers should locate at the outer orbits that corresponds to the central areas of the Gaussian distribution.

We approximate the mode of $|\mathcal{A}_i^{(k)}|$ using the mean of the Gaussian distribution, which is the Hamming distance between a random k-mer and the origin. As the origin can be any k-mer, we could consider it as a random k-mer and calculate the expected Hamming distance between two random k-mers. Under the uniform k-mer distribution hypothesis, the expected Hamming distance between two random k-mers given by the following remark:

Remark 1.1. *The expected Hamming distance between two random k-mers* $\boldsymbol{x}^{(k)}$ *and* $\boldsymbol{y}^{(k)}$ *is* $\frac{3}{4}k$, *where* $\boldsymbol{y}^{(k)}$ *could fixed to the origin.*

Proof. Consider the ith ($i \in \{1, 2, \cdots, k\}$) position of the k-mers, there are two cases for calculating Hamming distance. In case 1, we have $x_i = y_i = A, C, G, T$, which has 4 possibilities. In case 2, we have $x_i \neq y_i$, which has 12 possibilites. The Hamming distance for case 1 and 2 are 0 and 1, respectively. So the expected Hamming distance for the ith position is given by

$$0 \times \frac{4}{4+12} + 1 \times \frac{12}{4+12} = \frac{3}{4}$$

Since all k positions can be treated in the same way, the expected Hamming distance between $\boldsymbol{x}^{(k)}$ and $\boldsymbol{y}^{(k)}$ is $\frac{3}{4}k$.

Due to the symmetry of the k-mer manifold, we could fixed $\boldsymbol{y}^{(k)}$ to the origin and still get the same conclusion. □

Fig. 3 shows the k-mer manifold for $k = 8$, which consists of 9 orbits. It can be seen that orbit-5,6,7 have the most dense points. According to Remark 1.1, the expected Hamming distance of a random k-mer to the origin is $\frac{3}{4}k = \frac{3}{4}8 = 6$, which confirms our observation.

5 Hamming ball

Given the k-mer manifold, we would like to group similar k-mers together using a Hamming Ball $\mathcal{B}^{(k)}$, which contains all k-mers with a Hamming distance less than or equal to a predefined radius $r^{(k)}$ (Fig. 3). The hamming ball function defined on the manifold $\Omega^{(k)}$ is given by

$$hb(\Omega^{(k)}, \boldsymbol{o}^{(k)}, r^{(k)}) = \{\boldsymbol{s}^{(k)} \mid \forall d(\boldsymbol{s}^{(k)}, \boldsymbol{o}^{(k)}) \leq r^{(k)} \ \& \ \boldsymbol{s}^{(k)} \in \Omega^{(k)}\} \tag{12}$$

Under the null hypothesis (uniform distribution), the probability of a Hamming ball is given by

$$\begin{aligned} p_{unif}(\mathcal{B}^{(k)}) &= \frac{|\mathcal{B}^{(k)}|}{|\Omega^{(k)}|} = \frac{|hb(\Omega^{(k)}, \boldsymbol{o}^{(k)}, r^{(k)})|}{|\Omega^{(k)}|} \\ &= \frac{\sum_{i=0}^{r^{(k)}} |\mathcal{A}_i^{(k)}|}{4^k} \end{aligned} \tag{13}$$

We noticed that the pmf demonstrated an increasing-peak-decreasing trend for all $k \in \{5, 6, \cdots, 20\}$ in Fig. 2. Here we choose $r^{(k)} = 2$ for $k = 8$ as the reference cutoff for a **standard Hamming ball** $\mathcal{B}_0^{(k)}$, k-mers within which are considered similar enough to be included in a motif. As shown in Fig. 3, the central part (highlighted in circle) are the Hamming ball. It can be seen that the k-mer manifold is extremely large. The point densities show that the outer orbits (rings, orbit-5,6,7) contain majority of the k-mers (points). Points in the standard Hamming ball only constitute a small proportion of k-mers in the k-mer manifold.

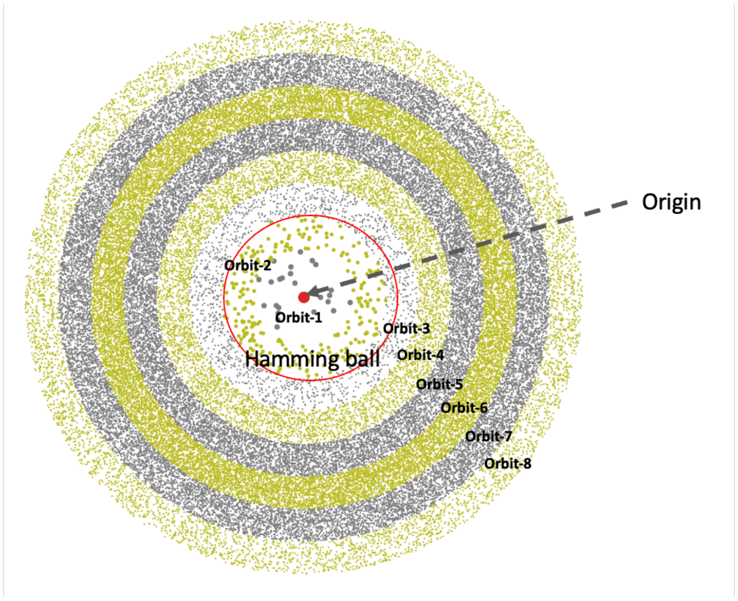

Figure 3: Schematic representation of k-mer manifold in 2d for $k = 8$. Each point represents a k-mer. We use $r^{(8)} = 2$ for the standard Hamming ball, as indicated by the red circle. We assign each k-mer of the ith orbit to a random point in the $i + 1$th ring. Each ring has an equal width.

Let us denote the probability of the standard Hamming ball $\mathcal{B}_0^{(k)}$ under the null hypothesis as b_0 (Eq. 13). To harmonize different k, we would like to set the Hamming ball radius r such that the probabilities of the Hamming ball under the null hypothesis are similar across different k, i.e.

$$r^{(k)} = \underset{r \in \{0,1,\cdots,k\}}{\operatorname{argmin}} |p_{unif}(hb(\Omega^{(k)}, \boldsymbol{o}^{(k)}, r)) - b_0| \tag{14}$$

Based on this criterion, we derive the radius for different k-mer length k by using $r^{(8)} = 2$ as the reference, as shown in Table 1. Meanwhile we provide the probabilities of the Hamming ball given the specified radius $r^{(k)}$ under the uniform k-mer distribution hypothesis.

k	$r^{(k)}$	$p_{unif}(\mathcal{B}^{(k)})$
3	0	0.015625
4	0	0.00390625
5	0	0.0009765625
6	1	0.004882813
7	1	0.001403809
**8**	**2**	**0.004241943**
9	2	0.001346588
10	3	0.00350666
11	3	0.001188517
12	4	0.00278157
13	5	0.005649343
14	5	0.002154179
15	6	0.004193015
16	6	0.001644466
17	7	0.003100779
18	8	0.005421779
19	8	0.002288429
20	9	0.003942142
21	10	0.00642271
22	10	0.002870987
23	11	0.004646849
24	11	0.002094047
25	12	0.003370448
26	13	0.005211411
27	13	0.002449967
28	14	0.003781377
29	15	0.005645352
30	15	0.002749534
31	16	0.004106948

Table 1: Hamming ball radius and probabilities for different k-mer lengths.

In the real data, we calculate the empirical probability for a given Hamming Ball $\mathcal{B}^{(k)}$ by

$$p_{empr}(\mathcal{B}^{(k)}) = \frac{\sum_{\boldsymbol{s}^{(k)} \in \mathcal{B}^{(k)}} c_{\boldsymbol{s}^{(k)}}}{\sum_{\boldsymbol{s}^{(k)} \in \Omega^{(k)}} c_{\boldsymbol{s}^{(k)}}}, \tag{15}$$

where $c_{\boldsymbol{s}^{(k)}}$ denotes the count of $\boldsymbol{s}^{(k)}$ in the empirical data. Given a random DNA sequence, there exist variations between $p_{empr}(\mathcal{B}^{(k)})$ and $p_{unif}(\mathcal{B}^{(k)})$. We denote the ratio $\gamma = p_{empr}(\mathcal{B}^{(k)})/p_{unif}(\mathcal{B}^{(k)})$ as the **Hamming ball ratio**. The expected mean of this ratio is 1, while its standard deviation has to be estimated from the real data. We first generate a random DNA sequence of length 100,000 bp, where the nucleotide at each position is uniformly drawn from {A,C,G,T}. Given the random DNA sequence, we calculate the Hamming ball ratio for each k-mer and derive the null distribution of Hamming ball ratio. Fig. 4 shows the Hamming ball ratio distribution for $k = 5, 6, \cdots, 16$ by repeating the above process on 10 times. It can be seen that that densities are Gaussian-like, with the mean near 1. For each density line, we fix the mean to 1 and calculate the standard deviation (std). We use a Gaussian distribution to represent these density lines, with the mean fixed to 1 and std taken as average over 10 replicates.

The empirical Hamming ball ratio distribution (the fitted Gaussian distribution) is used as the null distribution to test the significance of a random Hamming ball observed in the real data. If the ratio is larger than a predefined cutoff, it means that the given Hamming Ball is over-represented in the data and could be considered as a motif. We fix the mean of the null distribution to 1 and calculate the threshold corresponding right tail p-value 10^{-10}, as shown in Table 2. These thresholds are used for the motif discovery algorithm in Supplementary Note 2.

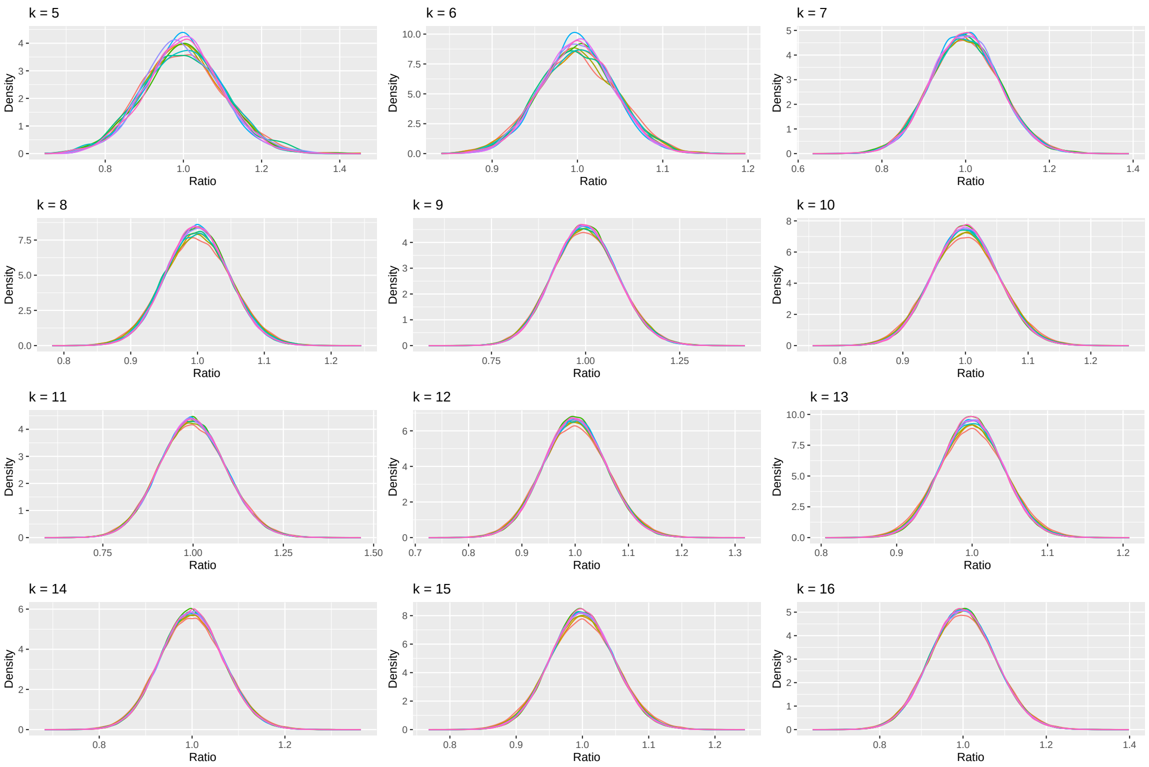

Figure 4: Empirical distribution for Hamming ball ratio on random data for $k = \{5 \cdots 16\}$. Each density line is obtained from a 100,000 bp random DNA sequence. This process is repeated 10 times, thus there are 10 lines.

k	Mean	Std	ratio(p=1e-10)
5	1	0.10140962	1.645101
6	1	0.04641315	1.29525
7	1	0.08646003	1.550002
8	1	0.04864974	1.309478
9	1	0.08605892	1.54745
10	1	0.0534363	1.339927
11	1	0.09138465	1.581329
12	1	0.05997705	1.381534
13	1	0.04208481	1.267716
14	1	0.06800379	1.432595
15	1	0.04876416	1.310205
16	1	0.07773227	1.494481

Table 2: Estimated parameters of Hamming ball ratio distribution. The mean is fixed to 1. The std is the average of std obtained from 10 replicative experiments. The third column are thresholds corresponding to p-value of 1e-10, i.e. 10^{-10}.

6 Properties of k-mer manifold

This section tries to illustrate several special properties of the k-mer manifold that is counter-intuitive to the commonly used Euclidean manifold.

6.1 Hamming distance properties

We have shown that the Hamming distance is a proper metric in Sec. 3. However, the Hamming distance is different to the commonly used Euclidean distance.

First, we show k-mer manifold is isotropic, i.e. the manifold looks the same from any given origin. For simplicity, we use a 3-bit string to illustrate this idea, where each bit can be either 0 or 1. As shown in Fig. 5, the manifold after rotation looks the same by centering on three different 0-1 strings: 000, 110, 111.

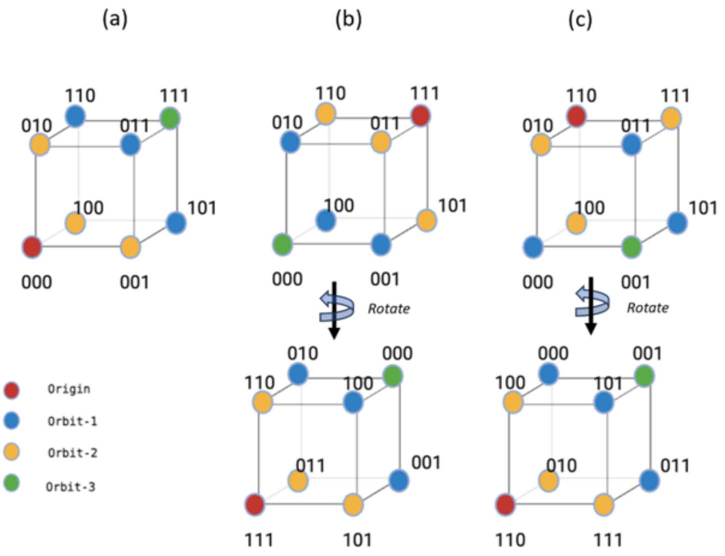

Figure 5: Isotropy of Hamming distance space. Each vertex is a 3-bit 0-1 string. The Hamming distance between vertices with an edge is 1. The three panels use (a) 000 (b) 110 (c) 111 as the origin, respectively. Orbits 0, 1, 2, 3 are colored in red, yellow, blue and green, respectively. The top panels of (b) and (c) shows the cubic from the same view point of (a). The lower panels of (b) and (c) are rotated cubics of the top panel. It can be seen the three configurations demonstrate the same manifold structure.

Second, the triangle inequality of Hamming distance is different to that of Euclidean distance. As shown in Fig. 6, there are three k-mers A**C**CCTGC, AGCC**A**GC, AG**A**CTGC in orbit 1 of k-mer manifold cerntering on AGCCTGC ($k = 7$). The Hamming distances of all three k-mers to the origin is 1, as indicated by the bold mutations. In the Euclidean space, all points with a fixed distance to the origin forms a circle. The distances are almost always less than 2 when considering the Euclidean distance between any two points on the circle, e.g. the Euclidean distances between s_a and s_b is less than 2. However, the Hamming distance between these three k-mers are 2, which contradicts the Euclidean distances. Since the Hamming distance between any two random points in orbit 1 are almost always 2, there is no straightforward way to display the k-mer manifold in the Euclidean space.

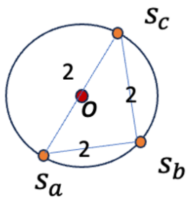

o : AGCCTGC
s_a: ACCCTGC
s_b: AGCCAGC
s_c: AGACTGC

Hamming distance

	o: AGCCTGC	s_a: ACCCTGC	s_b: AGCCAGC	s_c: AGACTGC
o: AGCCTGC	0	1	1	1
s_a: ACCCTGC	1	0	2	2
s_b: AGCCAGC	1	2	0	2
s_c: AGACTGC	1	2	2	0

Figure 6: Triangle inequality of Hamming distance. s_a, s_b, s_c are of Hamming distance 1 to the origin. However, the Hamming distances between themselves are 2, as highlighted by the rectangle in the distance matrix. It is impossible to arrange s_a, s_b, s_c in a circle in the Euclidean space by keeping the same distance constraints.

6.2 Hash key partition of k-mer manifold

It worth noting the symmetries of the k-mers given the reverse complement function. This property has introduced an additional challenge to process real data. Before entering the technical treatments, let us introduce Lemma 1 and Theorem 2 for proper treatments of the real data.

Lemma 1. *The hamming distance between $\boldsymbol{s}_1$ and $\boldsymbol{s}_2$ of length k is equal to the distance between $rc(\boldsymbol{s}_1)$ and $rc(\boldsymbol{s}_2)$*

$$d(\boldsymbol{s}_1, \boldsymbol{s}_2) = d(rc(\boldsymbol{s}_1), rc(\boldsymbol{s}_2))$$

Proof. For a given position p, if s_{1p} and s_{2p} are the same, then their complements $f(s_{1p})$ and $f(s_{2p})$ are also the same. If s_{1p} and s_{2p} are different, their complements $f(s_{1p})$ and $f(s_{2p})$ are also different. Therefore, $\mathbb{1}_{s_{1p} \neq s_{2p}} = \mathbb{1}_{f(s_{1p}) \neq f(s_{2p})}$. The reverse function only changes the position from p to $k+1-p$, but does not affect the Hamming distance calculation. The Hamming distance between the reverse complements is the same as the original k-mers. □

Theorem 2. *The hamming distance between $\boldsymbol{s}_1$ and $rc(\boldsymbol{s}_2)$ is equal to the distance between $rc(\boldsymbol{s}_1)$ and $\boldsymbol{s}_2$*

$$d(\boldsymbol{s}_1, rc(\boldsymbol{s}_2)) = d(rc(\boldsymbol{s}_1), \boldsymbol{s}_2)$$

Proof. By Lemma 1, $d(\boldsymbol{s}_1, rc(\boldsymbol{s}_2)) = d(rc(\boldsymbol{s}_1), rc(rc(\boldsymbol{s}_2))) = d(rc(\boldsymbol{s}_1), \boldsymbol{s}_2)$ □

Theorem 3. *The reverse complement function and the hamming ball function are commutative,*

$$rc(hb(\Omega^{(k)}, \boldsymbol{o}^{(k)}, r^{(k)})) = hb(\Omega^{(k)}, rc(\boldsymbol{o}^{(k)}), r^{(k)})$$

Proof. First we prove that $rc(hb(\Omega^{(k)}, \boldsymbol{o}^{(k)}, r^{(k)})) \subseteq hb(\Omega^{(k)}, rc(\boldsymbol{o}^{(k)}), r^{(k)})$.

For any element $\boldsymbol{s}^{(k)} \in rc(hb(\Omega^{(k)}, \boldsymbol{o}^{(k)}, r^{(k)}))$, we know that $rc(\boldsymbol{s}^{(k)}) \in hb(\Omega^{(k)}, \boldsymbol{o}^{(k)}, r^{(k)})$ according to the definition of the reverse complement function (Eq. 4). By the definition of the Hamming ball function (Eq. 12), we have $d(rc(\boldsymbol{s}^{(k)}), \boldsymbol{o}^{(k)}) \leq r^{(k)}$ & $rc(\boldsymbol{s}^{(k)}) \in \Omega^{(k)}$. By Therorem 2, we can get $d(\boldsymbol{s}^{(k)}, rc(\boldsymbol{o}^{(k)})) = d(rc(\boldsymbol{s}^{(k)}), \boldsymbol{o}^{(k)}) \leq r^{(k)}$. Of course, we have $\boldsymbol{s}^{(k)} \in \Omega^{(k)}$. Since $hb(\Omega^{(k)}, rc(\boldsymbol{o}^{(k)}), r^{(k)}) = \{\boldsymbol{s}^{(k)} \mid \forall d(\boldsymbol{s}^{(k)}, rc(\boldsymbol{o}^{(k)})) \leq r^{(k)}$ & $\boldsymbol{s}^{(k)} \in \Omega^{(k)}\}$, we know that $\boldsymbol{s}^{(k)} \in hb(\Omega^{(k)}, rc(\boldsymbol{o}^{(k)}), r^{(k)})$. As $\boldsymbol{s}^{(k)}$ can be any k-mer in $rc(hb(\Omega^{(k)}, \boldsymbol{o}^{(k)}, r^{(k)}))$, we have $rc(hb(\Omega^{(k)}, \boldsymbol{o}^{(k)}, r^{(k)})) \subseteq hb(\Omega^{(k)}, rc(\boldsymbol{o}^{(k)}), r^{(k)})$.

Then we prove $hb(\Omega^{(k)}, rc(\boldsymbol{o}^{(k)}), r^{(k)}) \subseteq rc(hb(\Omega^{(k)}, \boldsymbol{o}^{(k)}, r^{(k)}))$.

For any element $s^{(k)} \in hb(\Omega^{(k)}, rc(\boldsymbol{o}^{(k)}), r^{(k)})$, the relation $d(\boldsymbol{s}^{(k)}, rc(\boldsymbol{o}^{(k)})) \leq r^{(k)}$ & $\boldsymbol{s}^{(k)} \in \Omega^{(k)}$ holds. By Theorem 2, we can get $d(rc(\boldsymbol{s}^{(k)}), \boldsymbol{o}^{(k)}) = d(\boldsymbol{s}^{(k)}, rc(\boldsymbol{o}^{(k)})) \leq r^{(k)}$ and $rc(\boldsymbol{s}^{(k)}) \in \Omega^{(k)}$. Therefore, $rc(\boldsymbol{s}^{(k)}) \in hb(\Omega^{(k)}, \boldsymbol{o}^{(k)}, r^{(k)})$. So $\boldsymbol{s}^{(k)} = rc(rc(\boldsymbol{s}^{(k)})) \in rc(hb(\Omega^{(k)}, \boldsymbol{o}^{(k)}, r^{(k)}))$. Hence, $hb(\Omega^{(k)}, rc(\boldsymbol{o}^{(k)}), r^{(k)}) \subseteq rc(hb(\Omega^{(k)}, \boldsymbol{o}^{(k)}, r^{(k)}))$.

Based on the previous two conclusions, we get

$$rc(hb(\Omega^{(k)}, \boldsymbol{o}^{(k)}, r^{(k)})) = hb(\Omega^{(k)}, rc(\boldsymbol{o}^{(k)}), r^{(k)}).$$

□

$\boldsymbol{o}^{(k)}, \boldsymbol{o}^{(k-1)}, \boldsymbol{o}^{(k-2)}$ In real data processing, we usually store the k-mers as hash keys. Based on the hash key, we can partition the k-mer manifold into two hemispheres, which is an independent property of the orbits. We define the positive and negative hemispheres of $\Omega^{(k)}$ by $\Omega^{+(k)}$ and $\Omega^{-(k)}$ as follows.

$$\Omega^{+(k)} = \{\boldsymbol{s}^{(k)} \mid h(\boldsymbol{s}^{(k)}) \leq h(rc(\boldsymbol{s}^{(k)})),\ \forall \boldsymbol{s}^{(k)} \in \Omega^{(k)}\} \tag{16}$$

$$\Omega^{-(k)} = \{\boldsymbol{s}^{(k)} \mid h(\boldsymbol{s}^{(k)}) > h(rc(\boldsymbol{s}^{(k)})),\ \forall \boldsymbol{s}^{(k)} \in \Omega^{(k)}\} \tag{17}$$

Depending on the hash values of a k-mer, its reverse complement's hash key is either larger or smaller. In the case that the reverse complement of a k-mer is itself, the k-mer is called a palindrome, e.g. AGTACT. The hash key of a palindrome is the same as its reverse complement.

Let us start from an ideal situation that we only have one representation of each k-mer, i.e. either the k-mer or its reverse complement can be observed in the data. By ignoring the palindromes without losing generality (Fig. 7), a Hamming ball in the k-mer manifold can be separated into two parts, with one part living in the positive hemisphere and the other living in the negative hemisphere. For any k-mer in the positive part, we can map it to the negative hemisphere by taking its reverse complement (Fig. 7). Similarly, we could map the negative part to the positive hemisphere (Fig. 8). According to Theorem 3, we can find the union of the mapped parts (by reverse complement function) forms a new Hamming ball that centers on the reverse complement of the origin (Fig. 9).

Note that positive and negative hemisphere definition is only applicable to each k-mer and its reverse complement. The hash value of random k-mer in the positive hemisphere is not necessary smaller than that of a random k-mer in the negative hemisphere. As shown in Fig. 9, the hash value of GCAGCA (hash: 2340) living in the positive hemisphere is less than its reverse complement TGCTGC (hash: 3705). However, it is larger than k-mers AGCTGT (hash: 635) and GCACCT (hash: 2327) in the negative hemisphere. Therefore, the hash values of k-mers in the positive hemisphere are not necessarily less than that of k-mers in the negative hemisphere.

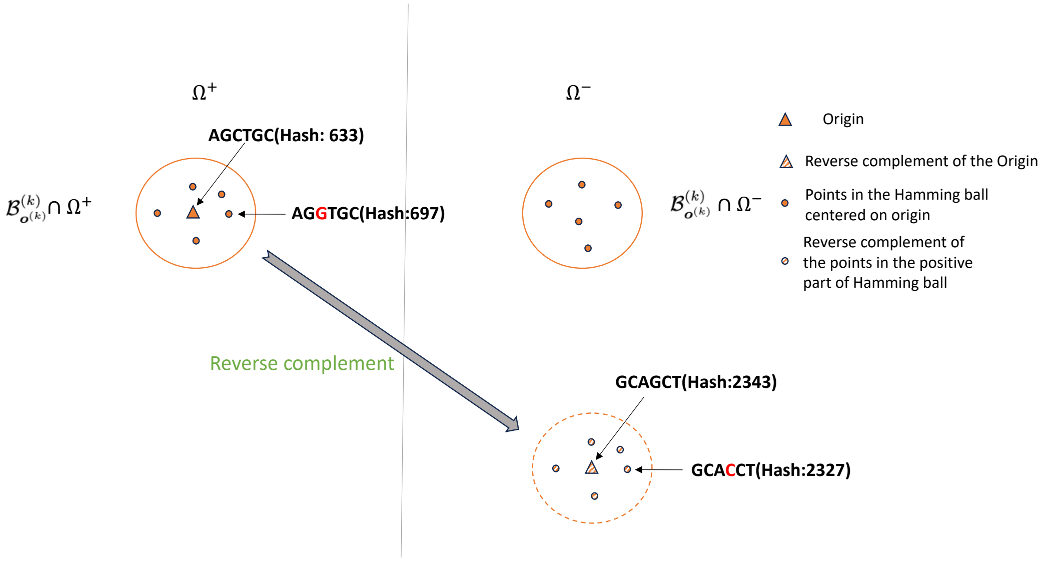

Figure 7: Reverse complements of the positive part of the Hamming ball $\mathcal{B}^{(k)}_{\boldsymbol{o}^{(k)}}$ ($k = 6$). The left part is the positive hemisphere Ω^+ and the right part is the negative hemisphere Ω^-. The Hamming ball is partitioned into two parts (solid circles). By taking the reverse complement, the positive part of the Hamming ball is mapped to the negative hemisphere (dashed circle). Two example k-mers and their hash values are provided. For each pair of k-mers, the k-mer in the positive hemisphere has a smaller hash.

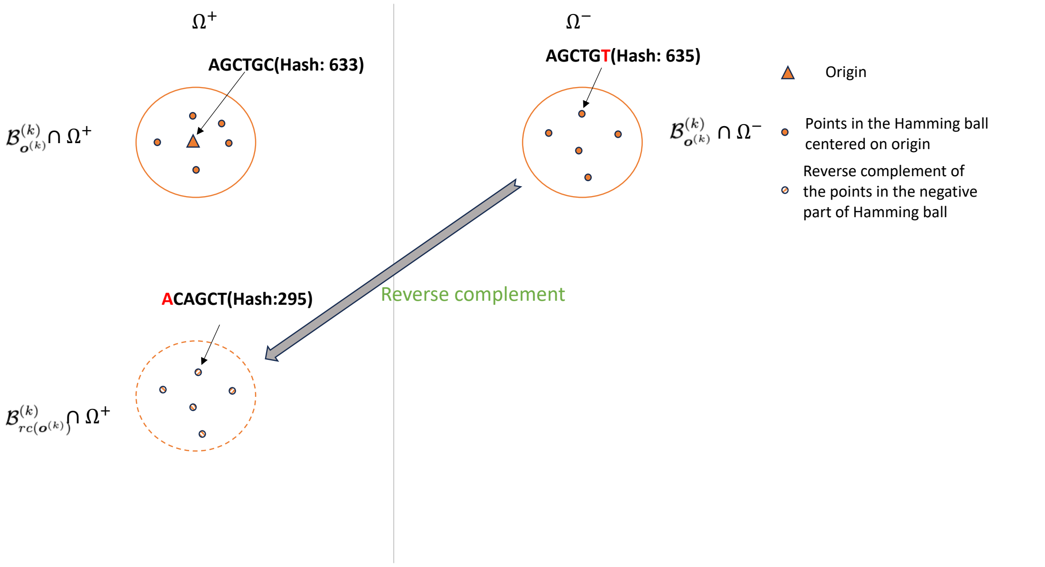

Figure 8: Reverse complements of the negative part of the Hamming ball $\mathcal{B}^{(k)}_{\boldsymbol{o}^{(k)}}$ ($k = 6$). It can be seen the Hamming distance of the example kmer $AGCTGT$ to the origin is 1, while its reverse complement $ACAGCT$'s Hamming distance to the origin is 5.

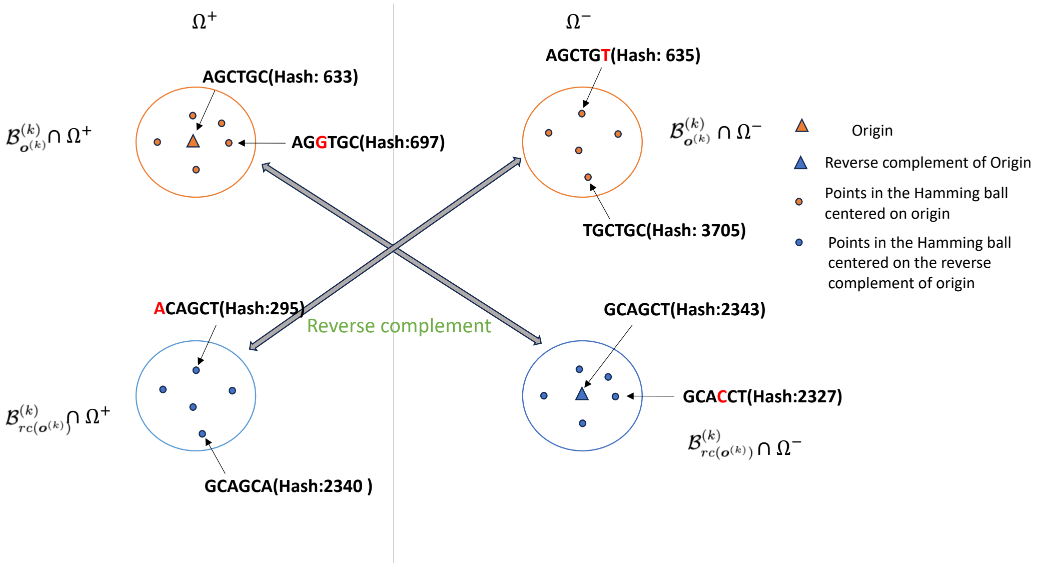

Figure 9: Reverse complement of a Hamming ball. It can be seen that the reverse complement of a Hamming ball (orange circles) is also Hamming ball (blue circles), which centers on the reverse complement of the origin (blue triangle). It can be seen that the example k-mers in the blue circles have maximally 1 mutation compared with the blue triangle (origin of the reverse complement of the Hamming ball).

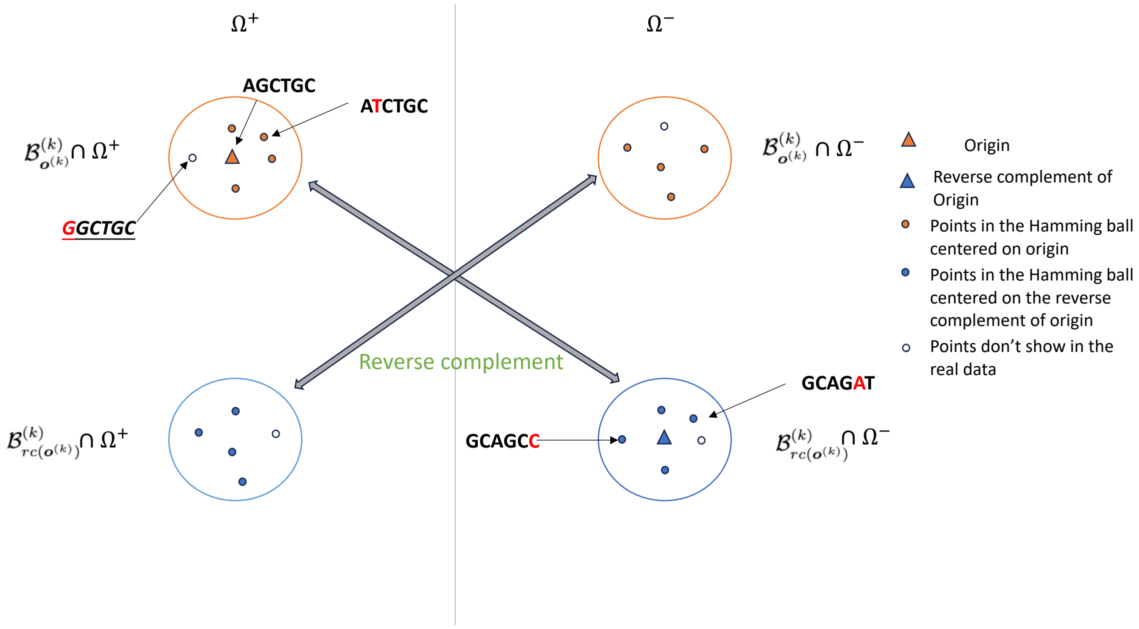

Figure 10: Scheme illustration of a Hamming ball in the real data. The real data may contain k-mers both in a Hamming ball and its reverse complement, i.e. all four large circles should be considered as one Hamming ball. Solid points are k-mers that can be observed in the real data, while unfilled points represent k-mers not observed, e.g. the underlined example k-mer GGCTGC.

The real data may contain k-mers in the four parts of Fig. 10. This means we need to treat all k-mers in the fours parts as the same Hamming ball. For some k-mers, we may have both the k-mer and its reverse complement, e.g. ATCTGC in Fig. 10. For other k-mers, we do not have its reverse complement, e.g. GCAGCC. In practice, we map all k-mers to the positive hemisphere and merge the counts of each k-mer pair (Fig. 11). For palindromes, the counts are not summed since the reverse complement of a palindrome is the same as itself. For a k-mer pair, only the k-mer in the positive hemisphere is kept for referencing in the processed data. When considering if a k-mer belongs to a Hamming ball, we need to calculate its Hamming distances both to the origin and the reverse complement of the origin, where the minimum of the two is taken as the final Hamming distance.

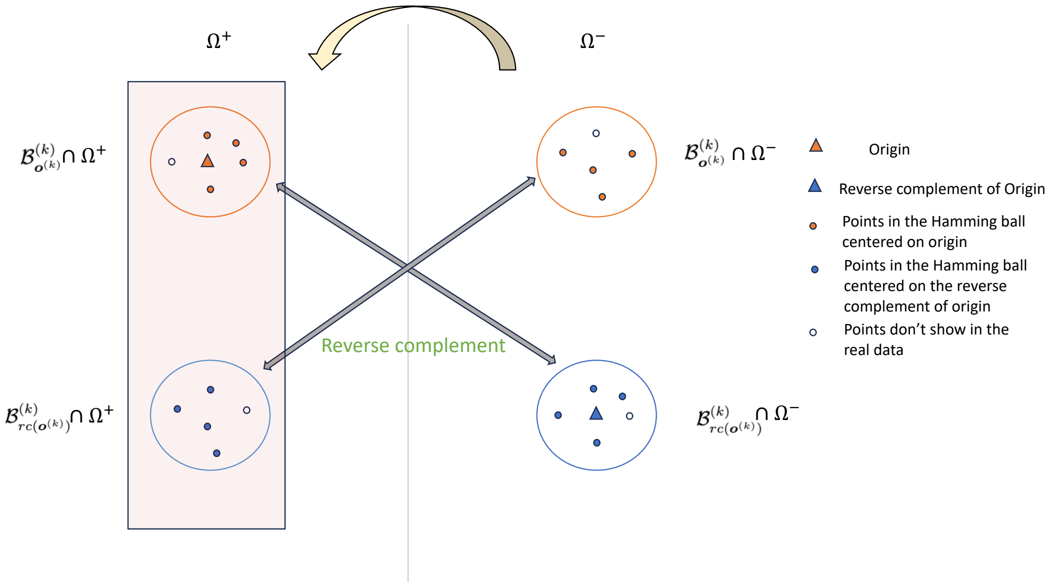

Figure 11: Real data processing. All k-mers in the negative hemisphere are reverse complemented to their corresponding mates in the positive hemisphere (highlighted by the rectangle) with the counts merged in the real data processing.
