# Supplementary material for: k-mer manifold approximation and projection for visualizing DNA sequences
Source: Genome Res. 2025 May;35(5):1234–46. doi: 10.1101/gr.279458.124 (PMC12047656; doi:10.1101/gr.279458.124)
Supplement: Supplement 3 [file Supplemental_Note_S2.pdf]

Motif discovery algorithm

(Supplementary Note 2)

Chengbo Fu, Lu Cheng

February 7, 2025

Contents

1 Introduction 1

2 Method 2

3 Practical treatments 4

1 Introduction

In modern biology, we are interested in studying various types of proteins, such as transcription factors (TF), RNA binding proteins (RBP). These proteins have some binding preference to certain DNA/RNA sequences, which have a shared pattern. This shared pattern is usually called motif in the literature.

There are different wet lab experiments to obtain the DNA/RNA sequences of a given TF or RBP. In SELEX-seq, TF is first mixed with random DNA sequences. Then TFs are fished out and the DNA not bound by TF are washed away. As a result, DNA sequences with high binding affinity to the TF are kept for the final sequencing. ChIP-seq is another technology based on a similar idea. The genome of live cells if first fragmented and TF is fished out by antibodies. Then, DNA sequences bound by TF are purified and squenced, from which we could estimate the binding specificities of the TF.

Traditionally researchers use position weight matrix (PWM) to describe the binding specificities of the TF, i.e. the motif. In this document, we use a set of k-mers (Hamming ball, defined in Supplementary Note 1) as the motif to characterize the binding preference of a TF. The process of estimating the motif from the DNA/RNA sequences from SELEX-seq, ChIP-seq, etc is called motif discovery.

In general, the input of motif discovery is a set of DNA/RNA sequences, which can have the same or different lengths. For simplicity, we convert the input sequences to a list of k-mers by sliding a window of length k along the input sequences. Based on the uniform k-mer distribution hypothesis in Supplementary Note 1, we derived the null distribution of the Hamming ball ratio and quantified the significance level for a Hamming ball ratio. In this way, we could filter out significant Hamming balls (motifs) from the input data.

Due to the large number of k-mers, it is impossible to visualize all k-mers in one figure. Sampling of the k-mers is needed to highlight the significant motifs of the input data, as well as background noise. Based on the detected motifs, we use supervised sampling to sample a set of k-mers ($N = 5000$) for downstream k-mer visualization.

For a small number of input k-mers ($< 10^5$ k-mers), there is no need to do motif discovery, we could perform unsupervised sampling and visualize the k-mers and quantify the clusters from the visualization.

In the supervised sampling case, the output the motif discovery algorithm includes (1) the consensus sequences, the proportion of k-mers in their Hamming balls and the Hamming ball ratios (2) sampled k-mers (3) motif labels of k-mers. For the unsupervised sampling case, the output is a list of k-mers, which does not contain any motif label information.

2 Method

We denote the input DNA sequences by $X = (\boldsymbol{x}_1, \boldsymbol{x}_2, \cdots, \boldsymbol{x}_M)$, where the M sequences can have different lengths and contain missing values like "N". Next we count the unique k-mers $S^{(k_i)} = (\boldsymbol{s}_1^{(k_i)}, \boldsymbol{s}_2^{(k_i)}, \cdots, \boldsymbol{s}_{M^{(k_i)}}^{(k_i)})$ from the input DNA sequences X for k-mer length k_i, whose counts are given by $\boldsymbol{c}^{(k_i)} = (c_1^{(k_i)}, c_2^{(k_i)}, \cdots, c_{M^{(k_i)}}^{(k_i)})$. Note that a k-mer is omitted in the counting if it contains a missing value "N". We then select top k-mers from $S^{(k_i)}$ that have the highest counts as the candidate consensus sequences. These candidate consensus sequences are used as origins of the k-mer manifold. The radius $r^{(k_i)}$ and a consensus sequence $\boldsymbol{o}^{(k_i)}$ jointly defines a Hamming ball $\mathcal{B}_{\boldsymbol{o}^{(k_i)}}^{(k_i)}$, which can be generated by the Hamming ball function $hb(\boldsymbol{o}^{(k_i)}, r^{(k_i)}, S^{(k_i)})$. We define the full list of Hamming ball radius for different k-mer lengths by $R = (r^{(5)}, r^{(6)}, \cdots, r^{(k_i)}, \cdots, r^{(31)})$, where the maximum k-mer length is set to 31.

Based on the uniform k-mer distribution hypothesis (Supplementary Note 1), the theoretical probability of a random Hamming ball $\mathcal{B}_{\boldsymbol{o}^{(k_i)}}^{(k_i)}$ is given by $p_{unif}(\mathcal{B}_{\boldsymbol{o}^{(k_i)}}^{(k_i)})$, which is a constant. The empirical probability of a Hamming Ball $\mathcal{B}_{\boldsymbol{o}^{(k_i)}}^{(k_i)}$ is given by

$$p_{empr}(\mathcal{B}_{\boldsymbol{o}^{(k_i)}}^{(k_i)}) = \frac{\sum_{\boldsymbol{s}_j^{(k_i)} \in hb(\boldsymbol{o}^{(k_i)}, r^{(k_i)}, S^{(k_i)})} c_j^{(k_i)}}{\sum_{j=1}^{M^{(k_i)}} c_j^{(k_i)}}$$

There exist some variation between the empirical probability of a random Hamming Ball and the theoretical uniform probability $b_0^{(k_i)}$. We define the **Hamming ball ratio** as

$$\gamma(\mathcal{B}_{\boldsymbol{o}^{(k_i)}}^{(k_i)}) = \frac{p_{empr}(\mathcal{B}_{\boldsymbol{o}^{(k_i)}}^{(k_i)})}{p_{unif}(\mathcal{B}_{\boldsymbol{o}^{(k_i)}}^{(k_i)})} \tag{1}$$

Given a random DNA sequence, we could calculate the Hamming ball ratios for all Hamming balls by centering on each unique k-mer, which yield the empirical distribution of the Hamming ball ratio. As this distribution is generated from random DNA sequence, it can be used as the null distribution of the Hamming ball ratio. Our null hypothesis of a DNA sequence dataset is that the DNA sequences are random. Therefore, the Hamming ball ratios should follow the null distribution. Given a Hamming ball ratio, we could compute the p-value for it and test its significance.

We generated a random DNA sequence of 100,000bp by uniformly sampling from $\{A, C, G, T\}$ with replacement. Next calculated the Hamming ball ratio for each unique k-mer ($k = 8$) in the sequence, which is used as the origin of a Hamming ball ($r^{(8)} = 2$). Figure 1 shows the Hamming ball ratio distribution. Since the density is uni-modal, we fit a Gaussian distribution with the mean fixed to 1. We also derived the estimated Gaussian distribution for $k = 5, 6, \cdots, 16$. To gain a robust estimation, we repeated the process on 10 random DNA sequences and yielded 10 Gaussian distributions for each k-mer length. For each k-mer length, the mean of standard deviations of 10 Gaussian distribution is used as the standard deviation of the final Gaussian distribution, where the mean is fixed to 1. The final Gaussian distribution is used as the null distribution for the Hamming ball ratio. Through practical experiences, we set the significance level to 10^{-10} (right tail p-value), which corresponds to the Hamming ball ratio cutoff γ_0. Hamming balls with a ratio greater than γ_0 are unlikely events under the null hypothesis, thus can be considered as motifs.

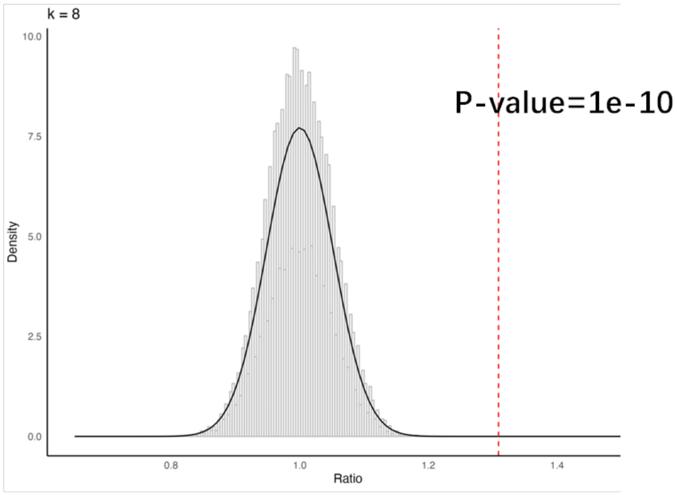

Figure 1: Hamming ball ratio distribution. It can be seen that the distribution is Gaussian-like and the mean is close to 1. The density line over the red histogram is the fitted Gaussian distribution with the mean fixed to 1. The black dash line is the significance threshold γ_0 (p-value $= 10^{-10}$).

To identify motifs from the sequencing data, we first pick out top 5 k-mers with the highest counts. Then we choose the k-mer with the largest Hamming ball and calculate its Hamming ball ratio. If the ratio is greater than the predefined significance threshold γ_0, the Hamming ball is treated as a motif, otherwise not. If the Hamming ball is identified as a motif, we will record it and mask all motif k-mers from the input DNA sequences. The process is iterated 10 times, which means that we identify 10 motifs at the maximum. The motif discovery algorithm is detailed in Alg. 1.

Algorithm 1 Motif discovery algorithm

Input: Input DNA sequences X, k-mer length k, Hamming ball radius $r^{(k)}$, Hamming ball ratio cutoff γ_0, Hamming ball ratio distribution $N(\mu_k, \sigma_k^2)$

Output: A list of consensus sequences O and their corresponding Hamming ball p-values P

$O \leftarrow \emptyset$

$M^{(k)} \leftarrow$ total number of k-mers in X

for $t = 1 \cdots 10$ **do**

  Derive unique k-mers $S^{(k)}$ and their counts $\boldsymbol{c}^{(k)}$ from the input data X, where k-mers containing 'N' are omitted.

  Get the top 5 k-mers $Z = (\boldsymbol{z}_1, \boldsymbol{z}_2, \boldsymbol{z}_3, \boldsymbol{z}_4, \boldsymbol{z}_5)$, $\boldsymbol{z}_i \in S^{(k)}$ with the highest counts in $\boldsymbol{c}^{(k)}$

  Count the number of k-mers in each Hamming ball with $\boldsymbol{z}_i$ as the origin, i.e. $n_i = \sum_j c_j^{(k)}\ \forall \boldsymbol{s}_j^{(k)} \in hb(\boldsymbol{z}_i, r^{(k)}, S^{(k)})$, for $i = 1 \cdots 5$

  Choose the largest Hamming ball with $\boldsymbol{z}_i$ as the consensus sequence s.t. n_i is the largest for $i = 1 \cdots 5$

  Calculate the Hamming ball ratio γ_i (Eq. 1), where the empirical probability of the Hamming ball is given by $n_i / M^{(k)}$

  if $\gamma_i > \gamma_0$ **then**

    append $\boldsymbol{z}_i$ to the consensus sequence list O

    append the p-value $N(\gamma_i | \mu_k, \sigma_k^2)$ to the p-value list P

    Mask k-mers of the Hamming ball $hb(\boldsymbol{z}_i, r^{(k)}, S^{(k)})$ in the input DNA sequences, i.e. the corresponding locations of the k-mer are replaced by "N"

    update X

  else

    break

  end if

end for

return O, P

As the k-mer space is huge, we have to perform sampling for downstream k-mer visualization. Based on the motif discovery results, we perform **supervised sampling** as follows. In total 5000 k-mers are sampled for visualization. Here we want to keep a proportion (50%) of motif k-mers (k-mers of identified motif Hamming balls) and a proportion (50%) of random k-mers (non-motif k-mers) to depict the whole k-mer manifold. All k-mers are assigned a motif class label or a random class label. The number of k-mers for each class is proportional to the size of each class, i.e. sum of k-mer counts in that class. Given the number of k-mers for each class, weighted uniform sampling with replacement is used to sample k-mers, where the weights are given by the k-mer counts. We then pool sampled k-mers of each class together for final visualization.

For certain scenarios (e.g. gene editing), the input DNA sequence data is relative small ($n < 50000$). **Unsupervised sampling** can be used for such scenarios. First, multiple sequence alignment is performed on the input DNA sequences (~250bp), such that all sequences are of equal length after alignment. Then, we are interested in picking out sequences/k-mers with high similarity, e.g. belonging to the same gene editing pattern. According to the k-mer manifold theory, similar k-mers should fall into the inner orbits, while random k-mers are scatter on the outer orbits. If there is a motif (Hamming ball) in the k-mer manifold, k-mers belonging to this motif should have a smaller distance to its neighbours than a random k-mer. This distance should follow a unimode distribution (Theorem 1 in Supplementary Note 1). Therefore, motif k-mers generally have a small distance to its neighbours. This property is utilized in the unsupervised sampling algorithm, as detailed in Alg. 2. The sampled k-mers are likely motif k-mers and also contain a small amount of random k-mers.

Algorithm 2 Unsupervised sampling algorithm

Input: Aligned input DNA sequences X (equal length)
Output: A list of sampled sequences S

```
S ← ∅
N ← |X|
Calculate the N × N pairwise distance matrix D for input DNA sequences X
Get the k = 600 nearest neighbours of each sequence using the k-nearest neighbors algorithm
  (kNN) based on the distance matrix D
Calculate the average distance of each sequence to its k neighbours, denoted by d̄ = (d̄_1, d̄_2, ··· , d̄_N)
Fit a Gaussian distribution N(μ, σ^2) to the average distances d̄
for i = 1 ··· N do
    if 0 ≤ d̄_i < μ − 2σ then
        Append x_i to S
    end if
end for
return S
```

The output of supervised sampling consists of a list of k-mers, their class labels (motifs and random) and consensus sequences of the motifs. The output of the unsupervised sampling is simply a list of k-mers/aligned DNA sequences.

3 Practical treatments

We have provided the algorithms for the ideal cases of motif discovery. However, there are many other factors to consider in the real motif discovery applications.

One significant factor to consider is the reverse complement. Since the sequencing data is generated for both DNA strands, one DNA sequence and its reverse complement actually represent the same DNA fragment. This cause problems in the motif identification. For example, a k-mer may be located at orbit 1, but its reverse complement may be located at the outer most orbit. Therefore, we map all k-mers to the positive k-mer space (Sec. 6.2 in Supplementary Note 1), such that both a k-mer and its reverse complement are counted together in Alg. 1. Note that palindromes are only counted once. When considering if a k-mer belongs to a Hamming ball, we always consider both the k-mer and its reverse complement, i.e. the k-mer also belongs to a Hamming ball if the Hamming distance between its reverse complement and the consensus sequence is smaller than the radius.

In the implementation, we allow users to input the consensus sequences of motifs based on their prior knowledge. Hamming balls of user motifs are masked from the input DNA sequences in the beginning of Alg. 1.

In motif discovery, we do not know the actual length of the motifs. Here we use a heuristic to merge motifs of different k to decide the final motifs. If a motif is strong enough, it should appear in different k-mer lengths. Here we require it to appear in three consecutive k-mer lengths. We first run the motif discovery algorithm for a series of k-mer lengths, e.g. $k = \{5, 6, \cdots 16\}$, which generate several motifs for each k. For a consensus sequence $\boldsymbol{o}^{(k)}$ to be considered as the final motif, we check if (1) it is a substring of any consensus sequence $\boldsymbol{o}^{(k+1)}$ and (2) any consensus sequence $\boldsymbol{o}^{(k-1)}$ is a substring of $\boldsymbol{o}^{(k)}$. If both conditions hold, we include $\boldsymbol{o}^{(k)}$ in the final motif list and remove all consensus sequences that are substrings of $\boldsymbol{o}^{(k+1)}$. Otherwise we remove $\boldsymbol{o}^{(k)}$ from the candidate motif list. By iterating this process from the maximum k-mer length to the minimum k-mer length, we identify all the motifs. Figure 2 shows the motif merging process. Detailed steps are provided in Algorithm 3

Algorithm 3 Motif merging algorithm

Input: Consensus sequence list $O^{(k_{min})}, \cdots, O^{(k)}, \cdots, O^{(k_{max})}$, where $O^{(k)} = (\boldsymbol{o}_1^{(k)}, \boldsymbol{o}_2^{(k)}, \cdots, \boldsymbol{o}_{N^{(k)}}^{(k)})$

Output: Final consensus sequence list S

$S \leftarrow \emptyset$
for $k = k_{max} \cdots k_{min}$ **do**
  for $i = 1 \cdots N^{(k)}$ **do**
    flag1 $\leftarrow$ False
    flag2 $\leftarrow$ False
    for $j = 1 \cdots N^{(k-1)}$ **do**
      if any $k-2$ substring of $\boldsymbol{o}_j^{(k-1)}$ is also a substring of $\boldsymbol{o}_i^{(k)}$ **then**
        $\boldsymbol{o}^{(k-1)} \leftarrow \boldsymbol{o}_j^{(k-1)}$
        flag1 $\leftarrow$ True
        break
      end if
    end for
    for $j = 1 \cdots N^{(k-2)}$ **do**
      if any $k-3$ substring of $\boldsymbol{o}_j^{(k-2)}$ is also a substring of $\boldsymbol{o}_i^{(k)}$ **then**
        flag2 $\leftarrow$ True
        break
      end if
    end for
    if flag1 = True and flag2=True **then**
      Append $\boldsymbol{o}^{(k-1)}$ to S
      Remove all consensus sequences if any of its $m-1$ substring is also a substring of $\boldsymbol{o}_i^{(k)}$ from $O^{(k_{min})}, \cdots, O^{(k)}, \cdots, O^{(k_{max})}$, where m denote the length of the given consensus sequence.
    end if
  end for
end for
return S

After motif merging, we may get several final motifs, which can have different lengths. The next step is to sample k-mers from different motifs to visualize the k-mers, where the Hamming distance matrix of the sampled k-mers is needed. A nature question is how to sample k-mers for these motifs and how to calculate the Hamming distance matrix for k-mers with different lengths. We always use the largest motif length as the k-mer length for visualization and only sample k-mers of this length. Next we explain how to assign a longer k-mer to a short motif, i.e. if the longer k-mer belongs to the Hamming ball of the shorter motif. We compare the longer k-mer with the short motif consensus sequence from the first position to the last position of the shorter motif, which yields the Hamming distance between the longer k-mer and the shorter motif. Here we neglect the remaining unmatched positions of the longer k-mer. We also derive the reverse complement of the longer k-mer and calculate

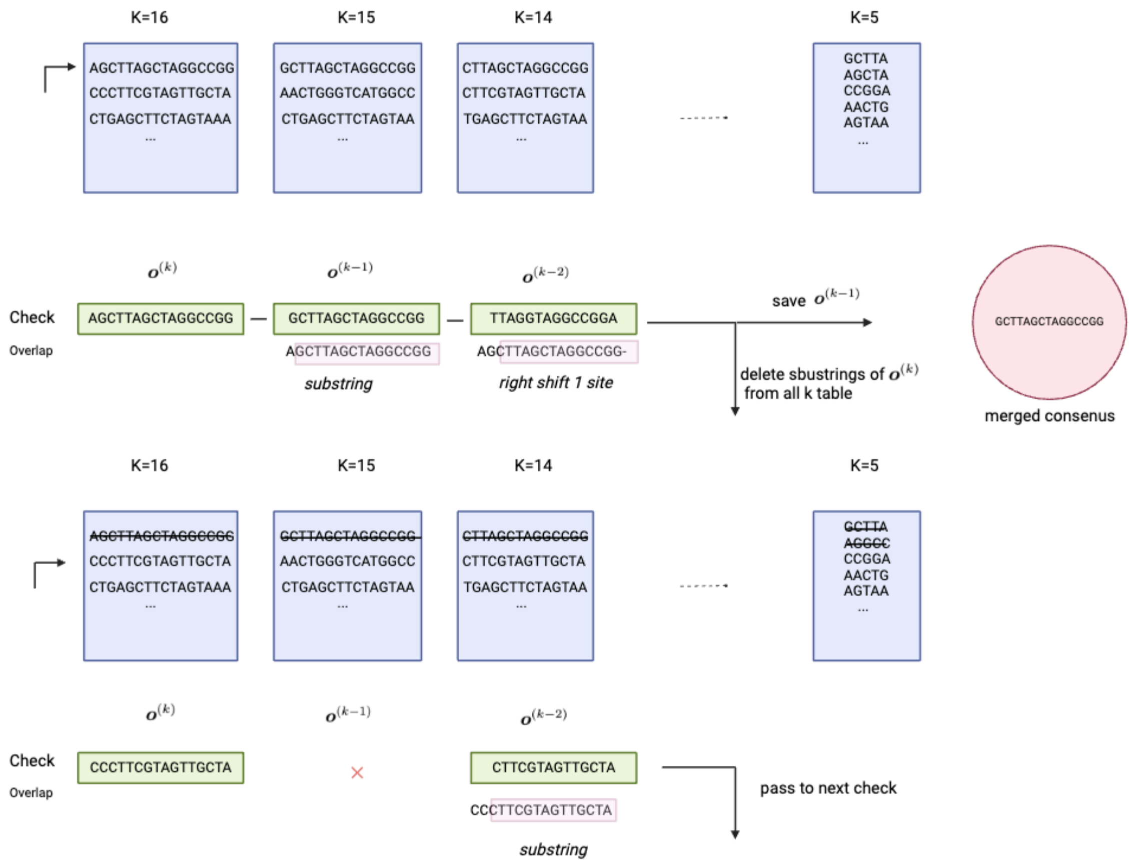

Figure 2: Motif merging process. We iterate over all candidate motif consensus sequences from the largest k to the smallest k. In the first pass, we have identified a motif sequence, which has shorter candidate motif sequences (can shift up to 1 nt) as its substrings. Then, all candidate motif consensus sequences that are substrings of the identified motif sequence are removed. After that, we start scanning motif sequence from the next candidate motif sequence. In the second pass, the candidate motif sequence is not selected since no k-1 candidate motif sequence is a substring of it.

its Hamming distance to the shorter motif in the same way. The smaller Hamming distance (the k-mer and the reverse complement to the motif) is used as the final Hamming distance. For example, we have a longer k-mer $CCAGACAC$ and a shorter motif consensus sequence $CCAGT$. The Hamming distance of $CCAGA$ and $CCAGT$ is 1 since we only compare the first 5 bases. For the reverse complement of $CCAGACAC$, which is $GTGTCTGG$, the Hamming distance is 5, given by comparing the first 5 bases $GTGTC$ and $CCAGT$. The Hamming distance between $CCAGACAC$ and the consensus sequence $CCAGT$ is 1, which is the smaller value of 1 and 5. If a longer k-mer is in the Hamming ball of the shorter motif, we will keep the mate with smaller distance to the motif, e.g. $CCAGACAC$ is kept instead of $GTGTCTGG$ in case $GTGTCTGG$ is the input k-mer. Then we replace the extra bases with "N", e.g. $CCAGACAC$ becomes $CCAGANNN$. In this way, we could sample k-mers for all motifs and calculate their Hamming distance matrix, where the Hamming distance between $\{A, C, G, T\}$ and "N" is 1.
