# Supplementary material for: k-mer manifold approximation and projection for visualizing DNA sequences
Source: Genome Res. 2025 May;35(5):1234–46. doi: 10.1101/gr.279458.124 (PMC12047656; doi:10.1101/gr.279458.124)
Supplement: Supplement 4 [file Supplemental_Note_S3.pdf]

k-mer manifold Approximation and Projection (KMAP) algorithm

(Supplementary Note 3)

Chengbo Fu, Lu Cheng

February 7, 2025

Contents

1 Introduction 1

2 Input and output 2

3 Loss function 2

4 Distance calculation 3

5 Inference algorithm 4

1 Introduction

Given a set of k-mers, we would like to visualize them in a 2 dimensional (2D) space to illustrate its structure. As we know, k-mers live in the k-mer manifold (Supplementary Note 1), which is a high dimensional non-euclidean space. Projecting k-mers in this high dimensional space requires a proper understanding of the k-mer manifold.

A popular method for project high-dimensional data to low-dimensional space is UMAP (Uniform Manifold Approximation and Projection), which is used in various scenarios. However, UMAP has difficulties in visualizing k-mers. The fundamental hypothesis of UMAP is that the high-dimensional data lives in a uniform space and the un-uniformality comes from the metric. For each data point, UMAP tries to scale the distances from their neighbours such that closer points are pushed away and distant points are pulled closer. However, this hypothesis contradicts with our goal in the sense that we would like to distinguish motif k-mers with random k-mers. The discreteness of the Hamming distance between k-mers, as well as redundancy of identical k-mers, poses challenges in the UMAP optimization.

Here we present the KMAP algorithm (k-mer Manifold Approximation and Projection) for visualizing k-mers in 2D, which is an extension of the UMAP algorithm. KMAP has made the following improvements to address the aforementioned challenges: (1) a global distance metric to depict the whole k-mer manifold (2) distance smoothing to pull similar points closer and handle distance discreteness (3) logistic transformation of distance to repulse distant k-mers (4) a diffusion term to solve the gradient exploding problem.

The output of KMAP is a 2D visualization of k-mers, where each point represent a k-mer. The inner part of the visualization are motif k-mers, while the outer part are random k-mers. Figure 1 shows the visualization results of both KMAP and UMAP. It can be seen that random k-mers are grouped into one cluster in UMAP. UMAP fails to converge after a few rounds of optimization due to gradient exploding.

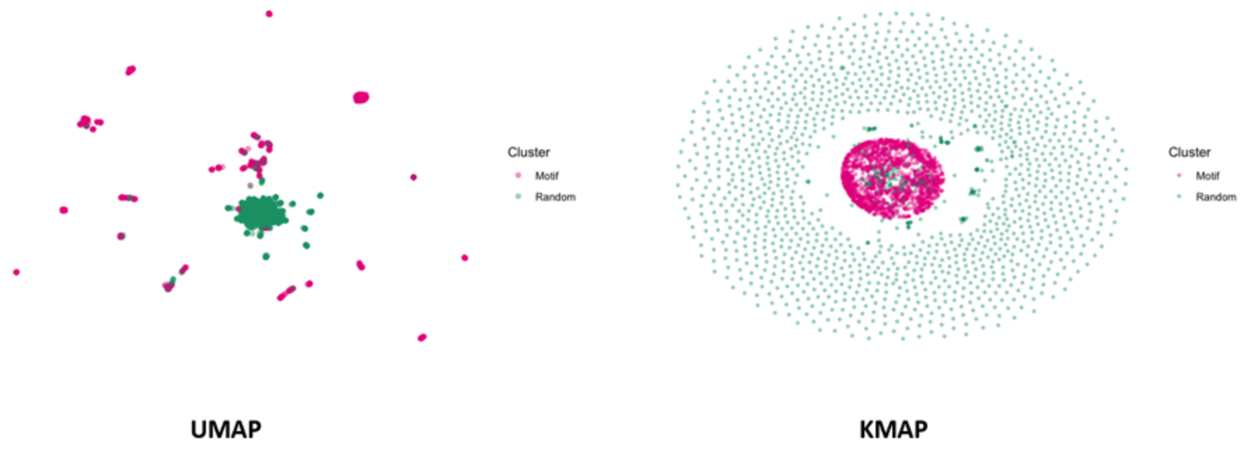

Figure 1: Visualization results of UMAP and KMAP on HT-SELEX data of transcription factor NFKB2. Each dot represents a k-mer. The motif k-mers are scattered around in UMAP due to gradient exploding. KMAP captures the main motif, as well as a minor motif right below the main motif.

2 Input and output

The input of KMAP is a list of k-mers denoted by $X = (\boldsymbol{x}_1, \boldsymbol{x}_2, \cdots, \boldsymbol{x}_N)$, where $\boldsymbol{x}_i$ could equal to $\boldsymbol{x}_j$ for $i \neq j$. In the supervised sampling case, we also have the labels of the input k-mers, but they are only used for coloring purposes in the final visualization.

The idea of KMAP is to project k-mers living in the k-mer manifold to low dimensional embeddings, which depicts important information of the k-mer manifold. KMAP projects each k-mer $\boldsymbol{x}_i$ to a 2D embedding $\boldsymbol{w}_i = (w_{i0}, w_{i1})$. We denote the low dimensional embeddings as $W = (\boldsymbol{w}_1, \boldsymbol{w}_2, \cdots, \boldsymbol{w}_N)$, which is the output of KMAP.

3 Loss function

In the projection, we would like to keep the similarities between all k-mer pairs in the low dimensional space. If $\boldsymbol{x}_i$ and $\boldsymbol{x}_j$ are similar, we would like their projections $\boldsymbol{w}_i$ and $\boldsymbol{w}_j$ to be close in the low dimensional space. On the contrary, we would like $\boldsymbol{w}_i$ and $\boldsymbol{w}_j$ to be distant when $\boldsymbol{x}_i$ and $\boldsymbol{x}_j$ are dissimilar. To quantify the similarity of $\boldsymbol{x}_i$ and $\boldsymbol{x}_j$, we could use the following high dimensional similarity probability

$$p_{ij} = exp(\frac{-d(\boldsymbol{x}_i, \boldsymbol{x}_j)}{2\sigma^2}), \tag{1}$$

where the distance function $d(\cdot)$ and scaling parameter σ is set to 0.5.

The low dimensional similarity probability is given by

$$q_{ij} = \frac{1}{1 + ||\boldsymbol{w}_i - \boldsymbol{w}_j||^2} \tag{2}$$

Given the similarity matrices P and Q, we could evaluate the goodness of projecting X to W by the following loss function:

$$L_{\text{KMAP}} = \sum_i \sum_j p_{ij} \log \frac{p_{ij}}{q_{ij}} + (1 - p_{ij}) \log \frac{(1 - p_{ij})}{(1 - q_{ij})}, \tag{3}$$

where $i, j \in \{1, 2, \cdots, N\}$ and $i \neq j$. This loss function is the same as UMAP, which is the cross entropy between P and Q. The loss function gets its minimum value when $p_{ij} = q_{ij}$. The first term pulls similar k-mers together, while the second term pushes dissimilar k-mers away.

4 Distance calculation

Due to the intrinsic difference between the k-mer manifold and the Euclidean space (Sec. 6.1 in Supplmentary Note 1), it is not possible to pe rfectly map a k-mer to the 2D Euclidean space. The major problem is that the distances between k-mers within a Hamming ball are generally larger than that of a circle in the Euclidean space. Here we use a distance smoothing based on 20 nearest neighbours to mitigate this problem.

For each k-mer $\boldsymbol{x}_i$, we use N_i to denote its 20 nearest neighbours based on Hamming distance. The smoothed distance between $\boldsymbol{x}_i$ and $\boldsymbol{x}_j$ is given by

$$d_0(\boldsymbol{x}_i, \boldsymbol{x}_j) = \frac{1}{|N_i||N_j|} \sum_{\boldsymbol{s}_m \in N_i} \sum_{\boldsymbol{s}_n \in N_j} d_H(\boldsymbol{s}_m, \boldsymbol{s}_n), \tag{4}$$

where $\boldsymbol{s}_m$ and $\boldsymbol{s}_n$ are one of the 20 nearest neighbours of $\boldsymbol{x}_i$ and $\boldsymbol{x}_j$, respectively. $d_H(\cdot)$ denotes the Hamming distance function.

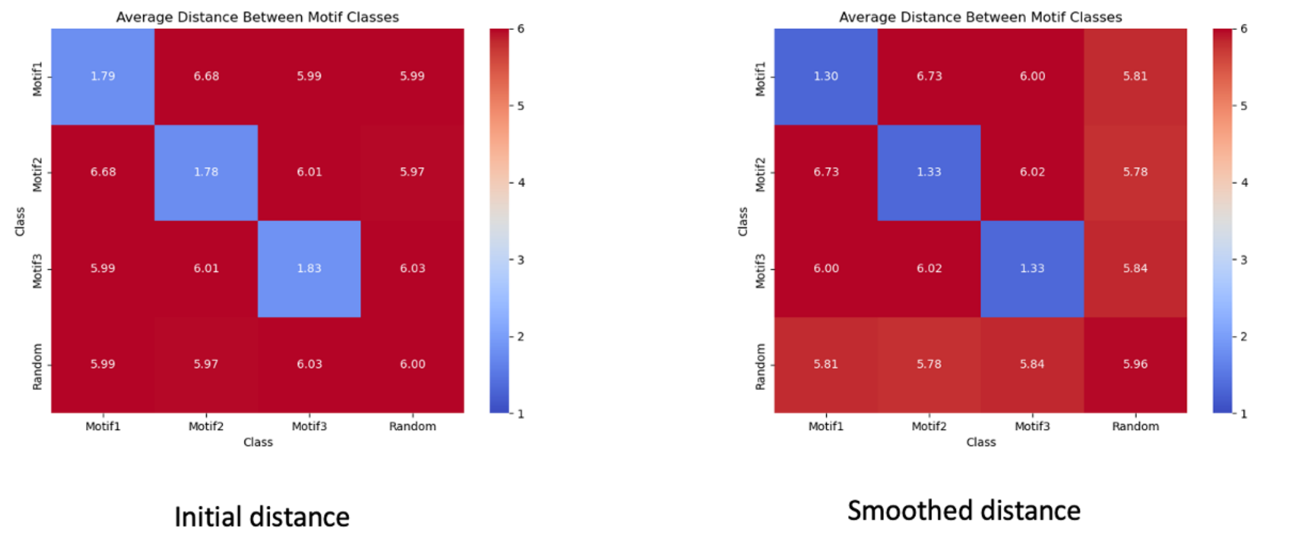

Figure 2: Smoothed k-mer distance on a simulated data ($k = 8$). The simulated data contains 3 motifs (500 k-mers each) and 1000 random k-mers. Each element in the heatmap shows the average distance across all pairs between different classes. It can be seen that the average distances within motif classes has drop from $\sim$1.8 to $\sim$1.3 after smoothing. It can also be seen that the average distance between two random k-mers is ~ 6, which is close to its theoretical expectation $\frac{3}{4}k = 6$ (Remark 1.1 in Supplementary Note 1).

Fig. 2 shows that the smoothed distances in a simulated data. Compared with the original Hamming distance, the average distances between k-mers within the Hamming ball (motif) has decreased, which better reflect the similarity in the Euclidean space. The smoothing function (Eq. 4) pulls motif k-mers closer.

After that, we perform a logistic transformation of the smoothed distance to enlarge the gap between a random Hamming ball and its outer orbits by the following function

$$f(x) = \frac{16}{1 + e^{-\gamma(x - x_0)}} = \frac{16}{1 + e^{-(0.2k - 0.2)(x - k/2)}}, \tag{5}$$

where k is the length of input k-mer. The transformed distance ranges between 0 and 16. $\gamma = 0.2k - 0.2$ controls the curvature of the transformation. $x_0 = k/2$ is the change point parameter. x_0 is the rough

boundary between Hamming ball and the outer orbits. According to Remark 1.1 in Supplementary Note 1, the expected distance between two random k-mers is $\frac{3}{4}k$, i.e. a k-mer in the Hamming ball and a k-mer in the outer orbits. Hence, we choose $x_0 = \frac{k}{2}$ as the rough boundary. The logistic transformation (Eq. 5) has the effect of repulsing motif k-mers from random k-mers.

In the end, we get the final distance

$$d(\boldsymbol{x}_i, \boldsymbol{x}_j) = f(d_0(\boldsymbol{x}_i, \boldsymbol{x}_j)) \quad \forall i, j \in \{1, 2, \cdots N\} \tag{6}$$

5 Inference algorithm

Here we want to optimize the values of W such that the loss function L_{KMAP} (Eq. 3) reaches its minimum. We use gradient descent algorithm to do this. The gradient is given by the following derivations. First we isolate all terms that depend on W from the loss function, i.e. q_{ij}.

$$\begin{aligned} L &= \sum_i \sum_j p_{ij} \log \frac{p_{ij}}{q_{ij}} + (1 - p_{ij}) \log \frac{(1 - p_{i;})}{(1 - q_{ij})} \\ &= \sum_i \sum_j -p_{ij} \log q_{ij} - (1 - p_{ij}) \log (1 - q_{ij}) + const, \end{aligned} \tag{7}$$

where $i \neq j$ and *const* is a constant that depend on p_{ij}. Note that p_{ij} is not a function of any $\boldsymbol{w}_i$, so it can be treated as a constant here.

Next we derive the gradient of q_{ij} w.r.t. $\boldsymbol{w}_i$.

$$\begin{aligned} \frac{\partial q_{ij}}{\partial \boldsymbol{w}_i} &= \frac{\partial (\frac{1}{1+||\boldsymbol{w}_i - \boldsymbol{w}_j||^2})}{\partial \boldsymbol{w}_i} \\ &= \frac{1}{(1+||\boldsymbol{w}_i - \boldsymbol{w}_j||^2)^2} \cdot -1 \cdot \frac{\partial (||\boldsymbol{w}_i - \boldsymbol{w}_j||^2)}{\partial \boldsymbol{w}_i} \\ &= \frac{-1}{(1+||\boldsymbol{w}_i - \boldsymbol{w}_j||^2)^2} \cdot \frac{\partial ((\boldsymbol{w}_i - \boldsymbol{w}_j)^T \cdot (\boldsymbol{w}_i - \boldsymbol{w}_j))}{\partial \boldsymbol{w}_i} \\ &= \frac{-1}{(1+||\boldsymbol{w}_i - \boldsymbol{w}_j||^2)^2} \cdot \left[(\frac{\partial ((\boldsymbol{w}_i - \boldsymbol{w}_j))}{\partial \boldsymbol{w}_i}) \cdot (\boldsymbol{w}_i - \boldsymbol{w}_j) + \frac{\partial ((\boldsymbol{w}_i - \boldsymbol{w}_j))}{\partial \boldsymbol{w}_i} \cdot (\boldsymbol{w}_i - \boldsymbol{w}_j) \right]^1 \\ &= \frac{-1}{(1+||\boldsymbol{w}_i - \boldsymbol{w}_j||^2)^2} \cdot \left[\boldsymbol{I}^T \cdot (\boldsymbol{w}_i - \boldsymbol{w}_j) + \boldsymbol{I}^T \cdot (\boldsymbol{w}_i - \boldsymbol{w}_j) \right] \\ &= \frac{-2(\boldsymbol{w}_i - \boldsymbol{w}_j)}{(1+||\boldsymbol{w}_i - \boldsymbol{w}_j||^2)^2} \\ &= -2(\boldsymbol{w}_i - \boldsymbol{w}_j) q_{ij}^2 \end{aligned} \tag{8}$$

Given the gradient of $\frac{\partial q_{ij}}{\partial \boldsymbol{w}_i}$, the gradient of the loss function L w.r.t. $\boldsymbol{w}_i$ is given by

[1] The rule of derivative of the dot Product $\frac{\partial(\boldsymbol{u}^{\boldsymbol{T}}\boldsymbol{v})}{\partial \boldsymbol{w}} = \frac{\partial(\boldsymbol{u})}{\partial \boldsymbol{w}} \cdot \boldsymbol{v} + \frac{\partial(\boldsymbol{v})}{\partial \boldsymbol{w}} \cdot \boldsymbol{u}$, where $\boldsymbol{u}$ and $\boldsymbol{v}$ are $N \times 1$ vectors.

$$
\begin{aligned}
\frac{\partial L}{\partial \boldsymbol{w}_i} &= \sum_i \sum_j -p_{ij} \cdot \frac{1}{q_{ij}} \cdot \frac{\partial q_{ij}}{\partial \boldsymbol{w}_i} - \frac{(1-p_{ij})}{1-q_{ij}} \cdot -1 \cdot \frac{\partial q_{ij}}{\partial \boldsymbol{w}_i} \\
&= 2\sum_j \left[\frac{-p_{ij}}{q_{ij}} + \frac{(1-p_{ij})}{(1-q_{ij})} \right] \cdot \frac{\partial q_{ij}}{\partial \boldsymbol{w}_i} \qquad \text{(only keep terms relevant w.r.t. } \boldsymbol{w}_i \text{)} \\
&= 2\sum_j \left[\frac{-p_{ij} + p_{ij}q_{ij} + q_{ij} - q_{ij}p_{ij}}{q_{ij}\,(1-q_{ij})} \right] \cdot \frac{\partial q_{ij}}{\partial \boldsymbol{w}_i} \\
&= 2\sum_j \frac{q_{ij} - p_{ij}}{q_{ij}\,(1-q_{ij})} \cdot \frac{\partial q_{ij}}{\partial \boldsymbol{w}_i} \\
&= 2\sum_j \frac{q_{ij} - p_{ij}}{q_{ij}\,(1-q_{ij})} \cdot -2(\boldsymbol{w}_i - \boldsymbol{w}_j) q_{ij}^2 \qquad \text{(Eq. 8)} \\
&= 2\sum_j \frac{2q_{ij}(p_{ij} - q_{ij})}{(1-q_{ij})} \cdot (\boldsymbol{w}_i - \boldsymbol{w}_j) \\
&= 4\sum_j (p_{ij} - q_{ij}) \frac{q_{ij}}{(1-q_{ij})} (\boldsymbol{w}_i - \boldsymbol{w}_j) \\
&= 4\sum_j (p_{ij} - q_{ij}) \frac{1}{\|\boldsymbol{w}_i - \boldsymbol{w}_j\|^2} (\boldsymbol{w}_i - \boldsymbol{w}_j)
\end{aligned}
\tag{9}
$$

Now have the gradient of the loss function. We could use the gradient descent algorithm to optimize $\boldsymbol{w}_i$. However, we note that the denominator of the middle term $\frac{1}{\|\boldsymbol{w}_i - \boldsymbol{w}_j\|^2}$ in Eq. 9 can be zero in the optimization, which causes gradient exploding. Therefore, we added a diffusion term (Gaussian noise) to $\boldsymbol{w}_i$ and $\boldsymbol{w}_j$ in the optimization when they are close.

$$
\boldsymbol{w_j} = \begin{cases} \boldsymbol{w_j} + \boldsymbol{\epsilon} & \text{if } \|\boldsymbol{w_i} - \boldsymbol{w_j}\| \le 0.1 \\ \boldsymbol{w_j} & \text{otherwise} \end{cases} \tag{10}
$$

where $\boldsymbol{\epsilon} = (\epsilon_0, \epsilon_1)$ and $\epsilon_0, \epsilon_1 \sim N(0, 0.01^2)$ are two independent Gaussian samples.

The full KMAP visualization algorithm is provided in Alg. 1, which is a typical gradient descent algorithm. After obtaining the low dimensional embeddings W, we could produce the 2D plot and color k-mers according to provided labels or perform further clustering to color the k-mers.

Algorithm 1 KMAP Visualization algorithm

Input: k-mer list $X = (\boldsymbol{x}_1, \boldsymbol{x}_2, \cdots, \boldsymbol{x}_N)$, learning rate $\eta = 0.01$, iterations $T = 2500$
Output: 2D embeddings $W = (\boldsymbol{w}_1, \boldsymbol{w}_2, \cdots, \boldsymbol{w}_N)$

Calculate the smoothed distance matrix D from X according to Eq. 6
Calculate the high dimensional similarity probability matrix P from D according to Eq. 1
Initialize W by sampling $\boldsymbol{w}_i$ from standard normal distribution, i.e. $w_{i0}, w_{i1} \sim N(0, 1^2)$ for $i = \{1, 2, \cdots, N\}$
for $t = 1, 2, \cdots, T$ **do**
  Calculate the low dimensional similarity probability matrix Q from W according to Eq. 2
  Calculate the current loss L_{curr} given P and Q according to Eq. 3
  for $i = 1, 2, \cdots, N$ **do**
    for $j = 1, 2, \cdots, N$ **do**
      if $i \neq j$ and $\boldsymbol{w}_j = \boldsymbol{w}_i$ **then**
        Update $\boldsymbol{w}_j$ by adding the diffusion terms according to Eq. 10
      end if
    end for
    Calculate the loss function gradient $\frac{\partial L}{\partial \boldsymbol{w}_i}$ w.r.t. $\boldsymbol{w}_i$ given P, Q, W according to Eq. 9
    Update $\boldsymbol{w}_i$ by $\boldsymbol{w}_i = \boldsymbol{w}_i - \eta \frac{\partial L}{\partial \boldsymbol{w}_i}$
  end for
  Calculate the low dimensional similarity probability matrix Q from updated W according to Eq. 2
  Calculate the new loss L_{new} given P and Q according to Eq. 3
  if $|L_{new} - L_{curr}| < 10^{-8}|L_{curr}|$ **then**
    break
  end if
  $L_{curr} \leftarrow L_{new}$
end for
return W
