# Supplementary material for: k-mer manifold approximation and projection for visualizing DNA sequences
Source: Genome Res. 2025 May;35(5):1234–46. doi: 10.1101/gr.279458.124 (PMC12047656; doi:10.1101/gr.279458.124)
Supplement: Supplement 5 [file Supplemental_Note_S4.pdf]

Supplemental Note 4

Additional discussions of KMAP

Chengbo Fu, Lu Cheng

This document tries to clarify certain aspects of KMAP, which are summarised from the discussions with reviewers in the review process of the manuscript. We organise the document into three sections: motif discovery algorithm, visulization algorithm and additional applications.

Table of Contents

1. *Motif discovery algorithm* **1**

1.1 Radius of the Hamming ball **1**

1.2 Non-uniform *k*-mer background distribution **3**

1.3 Secondary motifs **5**

2. *Visualization algorithm* **6**

2.1 Setting parameter x_0 in Eq. 2 .... **6**

2.2 Initialization of 2D embeddings **7**

2.3 Unusual patterns in t-SNE and UMAP **8**

3. *Additional applications* **9**

3.1 KMAP vs MEME on ATAC-seq data **9**

3.2 Composite motif analysis **12**

3.3 CTCF ChIP-seq data analysis **13**

1. Motif discovery algorithm

1.1 Radius of the Hamming ball

One of the concern is that if we have used a hard threshold (e.g. r=2) to define the radius of the Hamming ball.

The radius of a Hamming ball depends on the *k*-mer length. For *k*-mers of length 8, we select a radius of 2. As shown by the *k*-mer count distribution across different orbits in Fig. 1B (main text), the Hamming ball at this radius includes only a small fraction (0.424%) of all *k*-mers in a uniformly distributed *k*-mer space. By choosing a Hamming

ball that contains a small subset of *k*-mers, we reduce the likelihood that these *k*-mers are generated by random noise, making them more likely to be closely related to the consensus sequence.

In Supplemental Note 1, Section 4 ("Uniform Distribution Hypothesis"), we demonstrate that the *k*-mer count in orbit *i* follows a unimodal distribution as *i* increases from 0 to k. Based on this principle, we adjust the radius for various *k*-mer lengths to ensure that the Hamming ball consistently encompasses a similar proportion of *k*-mers as observed with k = 8 and r = 2. The derived orbit counts for *k*-mers of lengths ranging from 5 to 20, as well as the corresponding Hamming ball radius, are provided in Supplemental Note 1, Section 4.

For clarity, we present *k*-mer count distributions for different *k*-mer lengths in Figure 1 below.

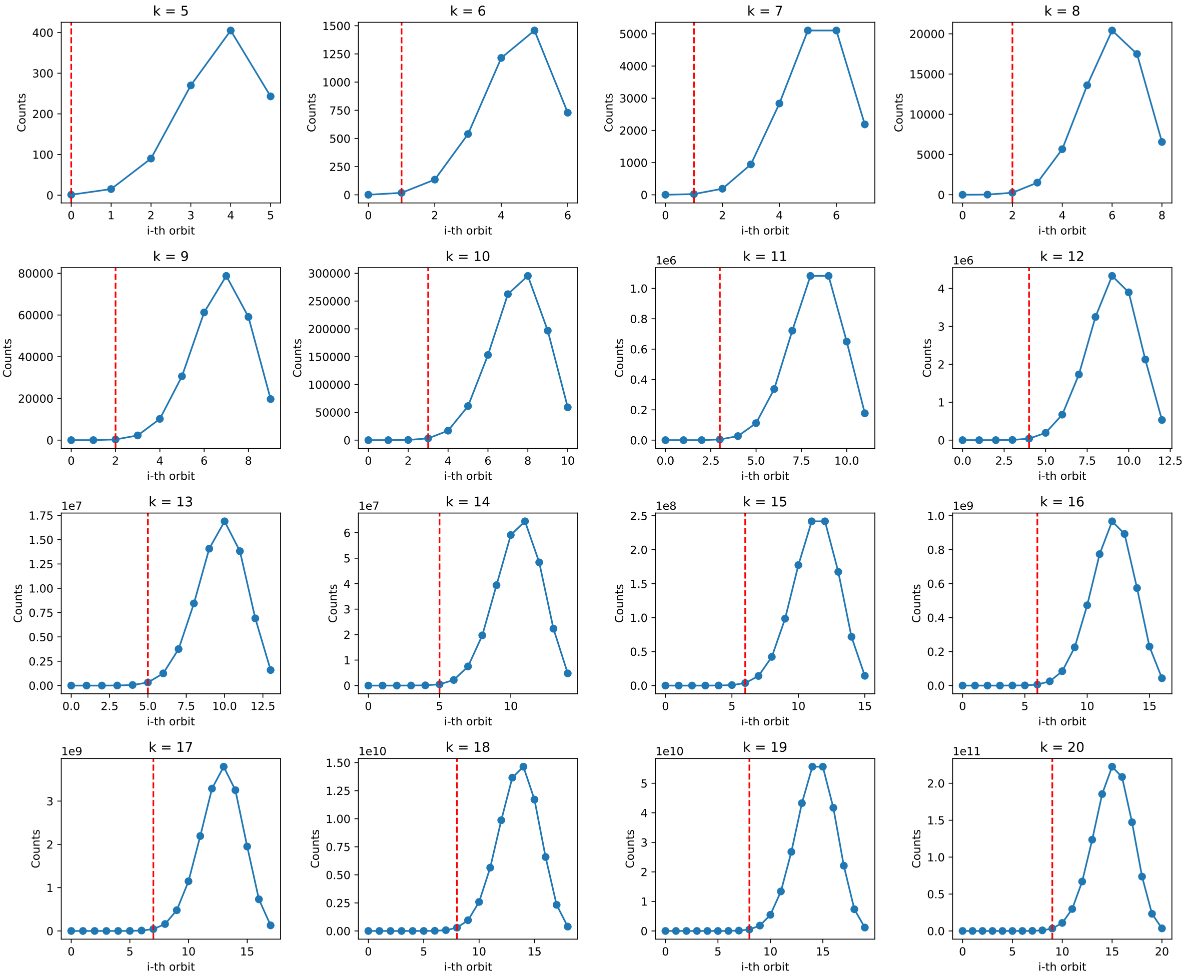

Figure 1: *K*-mer counts across different orbits under the uniform distribution hypothesis. Red dashed vertical line indicates the Hamming ball radius.

As shown, the radius r increases with k, while the proportion of *k*-mers within the Hamming ball remains relatively consistent across various *k*-mer lengths.

1.2 Non-uniform *k*-mer background distribution

One concern is that the human genome is not uniform. The GC content might challenge the uniform *k*-mer distribution assumption.

Our *k*-mer manifold theory is based on the assumption of a uniform *k*-mer space, which provides desirable theoretical properties, such as isotropy, allowing us to derive a consistent *k*-mer count distribution across orbits for all *k*-mers. However, if we consider differing nucleotide probabilities, such as higher GC content, we face several challenges:

- **Q1:** Does the unimodal assumption for *k*-mer count distribution across different orbits still hold?
- **Q2:** Without isotropy, the *k*-mer count distribution for each *k*-mer will vary. Can we feasibly derive this distribution?
- **Q3:** What is the expected distance between random *k*-mers, and how should we smooth distances between motif *k*-mers?

Our conclusions regarding these questions are as follows:

- **For Q1:** The *k*-mer count distribution loses its unimodal shape under non-uniform nucleotide distributions.
- **For Q2:** Deriving the *k*-mer count distribution for each consensus sequence requires a significant computational load for two reasons: (1) for each consensus sequence, we must enumerate combinatorial terms (equal to the binomial coefficient), and (2) this process must be repeated for all *k*-mers, treating each as a consensus sequence or origin.
- **For Q3:** We can calculate the expected distance from random *k*-mers to a consensus sequence under non-uniform conditions.

Our main obstacle in generalizing this theory is the complexity of deriving *k*-mer count distributions for each consensus sequence, which becomes computationally intensive. In Figure 2A, we show *k*-mer count distributions centered at “GCGCGCGC” and “ATATATAT” using a simulated sequence with a GC content of 60% (p(G)=p(C)=0.3, p(A)=p(T)=0.2). Interestingly, the counts within the Hamming ball (orbits 0, 1, and 2) remain close to the theoretical distribution under a uniform *k*-mer assumption, suggesting that the current model may serve as a reasonable approximation for non-uniform backgrounds (zero order Markov chain). However, given that the human genome likely exhibits higher Markov orders, this could explain the occurrence of repetitive motifs like “AAAAAAA” or “CCCCCCC.”

In a more extreme case with an 80% GC content (Figure 2B), the *k*-mer count distribution centered at “ATATATAT” is no longer unimodal, reflecting the impact of non-uniform assumption.

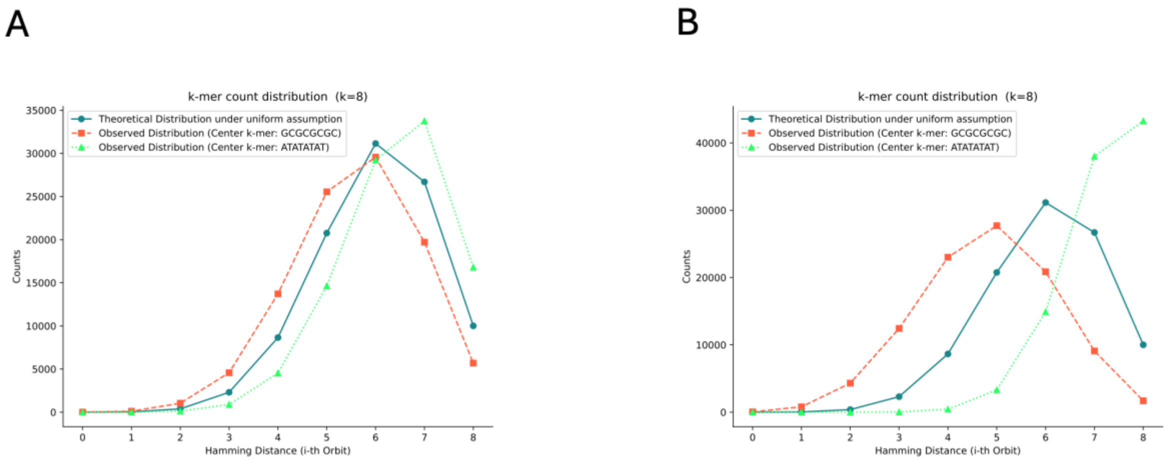

Figure 2: *k*-mer counts across different orbits centered at GCGCGCGC and ATATATAT, generated from a 100,000 bp simulated sequence with p(G)=p(C)=0.3 and p(A)=p(T)=0.2 (A, left panel) and p(G)=p(C)=0.4 and p(A)=p(T)=0.1 (B, right panel).

To mitigate this issue, we include various diagnostic outputs in the software package to assist users in exploring the data and evaluating results. For example, KMAP provides the proportion of reads containing a detected motif, which, if high, suggests the motif is more likely to be biologically relevant. KMAP also generates the positional distribution of motif occurrences within reads; for instance, motifs appearing centrally in ChIP-seq data are more likely to be true motifs. In the FLI1 ChIP-seq example (A673 dTAG cell, dTAGv−1 treatment, ETV6 knockout), 95.5% of reads contain the GGAA motif, which generally appears near the center, as shown in Figure 3A. This central occurrence suggests that the GGAA repeat is likely a true motif, while other motifs may require further investigation. Similarly, in the ETV6 ChIP-seq example (A673 parental cell, WT), 92% and 99.2% of reads contain the GGAA-repeat and AGGG-repeat motifs, respectively, both showing a central positional preference (Figure 3B). This suggests that these repetitive motifs are also likely genuine.

Based on our experience with different datasets (SELEX-seq, ChIP-seq, ATAC-seq), KMAP performs comparably to MEME.

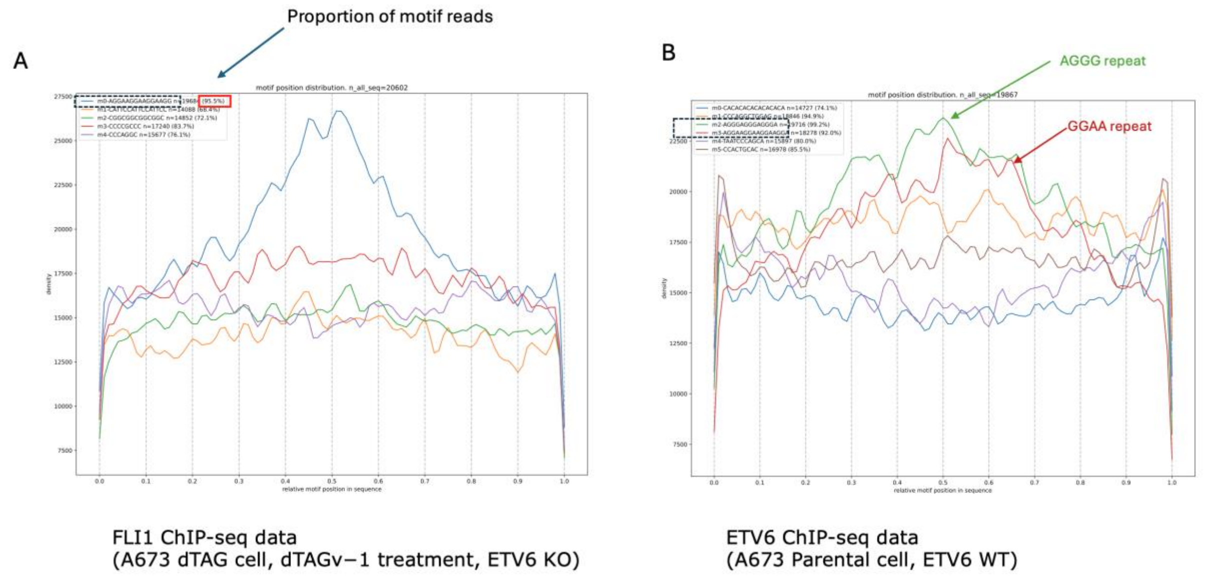

Figure 3: Relative position distributions of motifs.
(A) FLI1 ChIP-seq data: The GGAA-repeat motif shows a preference for central positions. Percentages indicate the proportion of input reads containing the given motif.
(B) ETV6 ChIP-seq data: Both the GGAA-repeats and AGGG-repeats display a preference for central positioning.

1.3 Secondary motifs

One concern is that how can we know if the secondary motif in the HT-SELEX data or other types of data (e.g. ChIP-seq) is technical artifact or biologically meaningful.

If a cluster forms, it indicates that these *k*-mers are statistically overrepresented based on the uniform *k*-mer manifold hypothesis. As mentioned in our previous response, a secondary motif is more likely to be genuine if it is not repetitive. For repetitive motifs, diagnostic information—such as the proportion of input sequences containing the motif and the positional distribution of the motif within these sequences—can help determine whether it is a real motif or a false positive.

As a data exploration tool, KMAP provides auxiliary information to help users make biological interpretations. KMAP can rank motifs by the p-values of their Hamming ball ratios, which may assist in distinguishing between major and secondary motifs. Assuming both the major and secondary motifs are non-repetitive, one approach is to check if these secondary motifs (or their reverse complements) partially match the major motif, indicating they may simply be shifts of the primary motif. If they are distinct from the major motif, their likelihood of being novel motifs increases. Furthermore, if secondary motifs are consistently located at the center or at a fixed distance from the major motif, their chances of being biologically relevant motifs also increase.

For example, in Figure 4, which shows KMAP results for FOXA1 ChIP-seq data, motifs 2 and 3 are repetitive and appear in a relatively low proportion of reads (38.2% and 30.2%, respectively), making them less likely to be true motifs. In contrast, motifs 0 and 1 are found in a higher proportion of reads, increasing their likelihood of being real motifs. Motif 0 is non-repetitive and has a preference for central positions, making it highly likely to be a true motif in this ChIP-seq data. Although motif 1 (a CA-repeat) is repetitive, it shows a mild preference for central locations, suggesting a moderate chance of biological relevance.

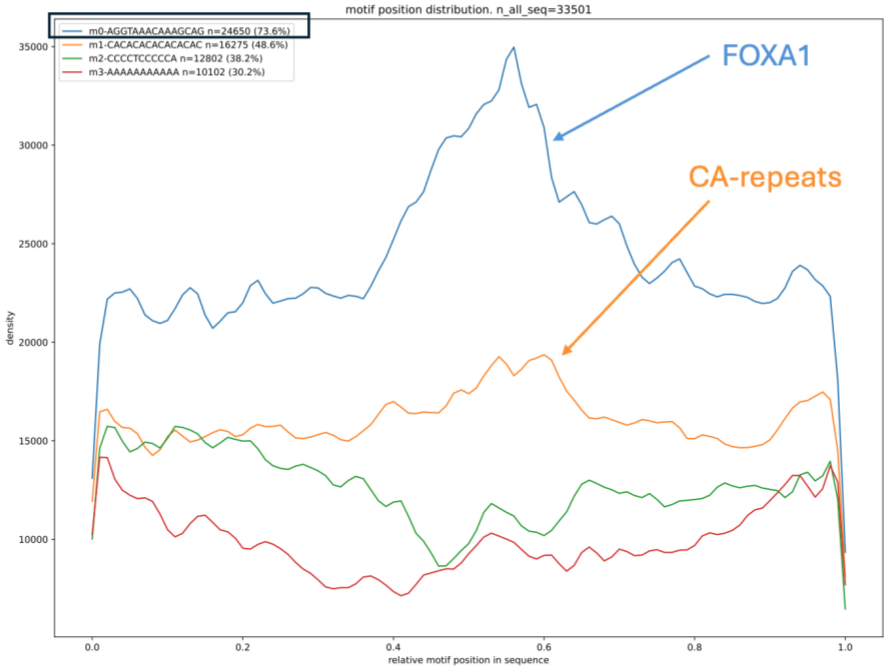

Figure 4: KMAP results (motif position distribution) for FOXA1 ChIP-seq data.

2. Visualization algorithm

2.1 Setting parameter x_0 in Eq. 2

One question is how to select the parameter x0 in Eq. 2.

Theoretically, x0 should be chosen as a value between the Hamming ball radius r and 3/4k, which is the expected distance of random *k*-mers from the consensus sequence (or origin). For *k*-mers within a distance of x0, they will be pulled toward the center; *k*-mers with a distance greater than x0 will be repelled. Our selection of x0 is guided by the following considerations:

1. Since the smoothed distance within a motif cluster is generally less than or equal to the Hamming ball radius r, x0 should be set larger than r to ensure that *k*-mers within a motif cluster together.
2. x0 should be less than 3/4k to avoid attracting too many random *k*-mers into the clusters.
3. x0 should also increase with *k*-mer length, as both the Hamming ball radius and 3/4k increase with k.

Based on these considerations, we empirically select x0 = k/2, which has proven effective across various datasets. To demonstrate the impact of x0, we vary it from r/2 to (3k/4 + k)/2 using the NFKB2 dataset and present the visualizations in Figure 5. As shown, x0 = k/2 provides a balanced and effective clustering.

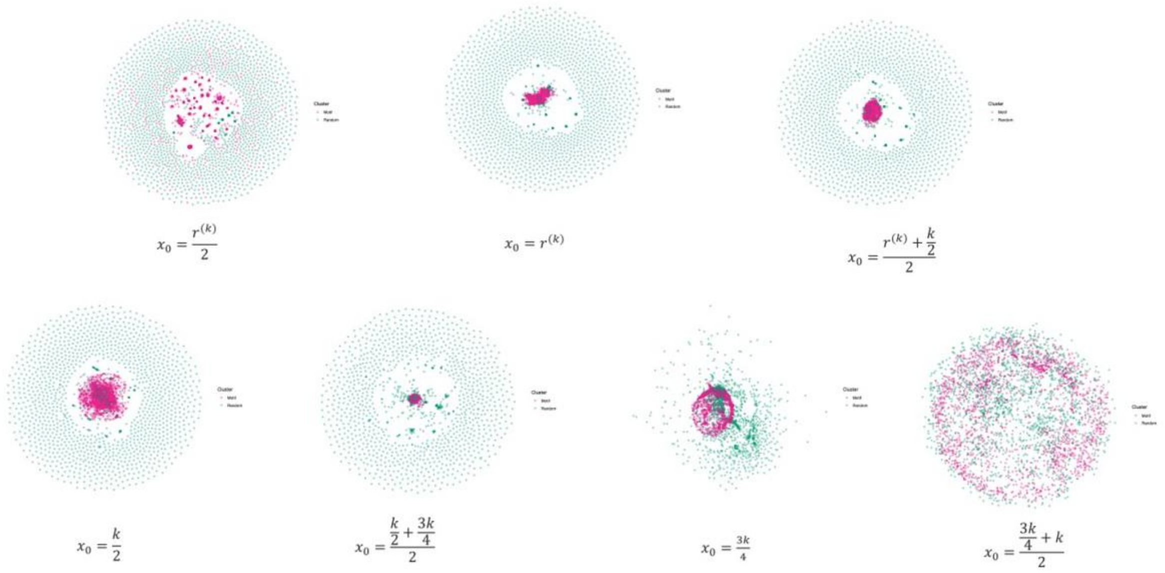

Figure 5: KMAP visualization results of NFKB2 dataset across different values of x0.

2.2 Initialization of 2D embeddings

One questions is how we initialize the 2d embeddings in KMAP. Will different initializations affect the final visualizations?

The initial 2D coordinates of the *k*-mers are randomly drawn from a Gaussian distribution N(0, 1^2). Despite different initial coordinates, the final visualizations remain very consistent. In Figure 6, we show visualization results for the NFKB2 data across 10 replicates, each using different initial coordinates. As seen, the final visualizations are very similar across replicates, indicating that KMAP is robust to initialization.

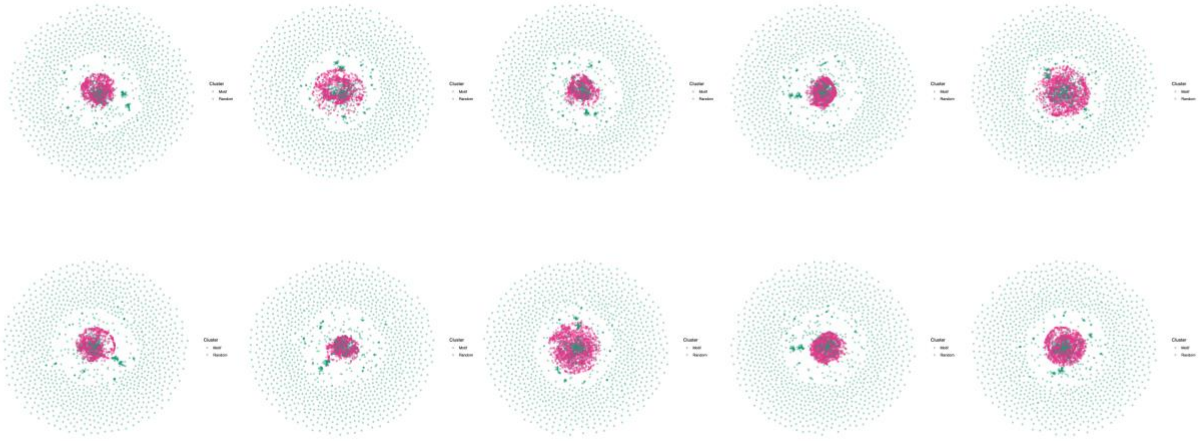

Figure 6: KMAP visualization results using different initial coordinates.

2.3 Unusual patterns in t-SNE and UMAP

One reviwer noticed the usually patterns in t-SNE and UMAP plot in Fig. 3D (main text), where random *k*-mers form a cluster while motif *k*-mers are splitted into small clusters. The question is if we have used the same distance matrix for all visulization matrix in Fig. 3D in the main text.

The input matrix for all visualizations, including UMAP, t-SNE, PCA, and MDS, is the Hamming distance matrix. In Figure 7 below, we display both the original Hamming distance matrix (left) and the KMAP smoothed distance matrix (right) for the 2500 motif *k*-mers and 2500 random *k*-mers from the NFKB2 SELEX data used in Figure 3C (main text). The KMAP smoothing process draws similar *k*-mers closer together, which can be observed in the smoothed matrix.

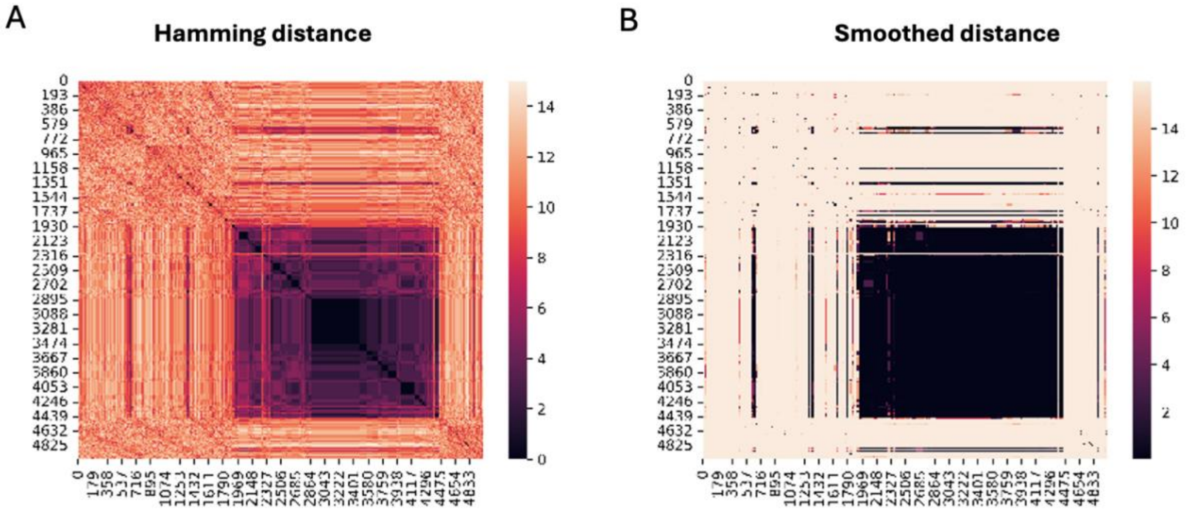

Figure 7: Hamming distance matrix (left) and KMAP smoothed distance matrix (right) for 2500 motif + 2500 random *k*-mers from NFKB2 SELEX data.

Additionally, in Figure 8, we show the visualization results of t-SNE and UMAP using the KMAP smoothed distance matrix as input.

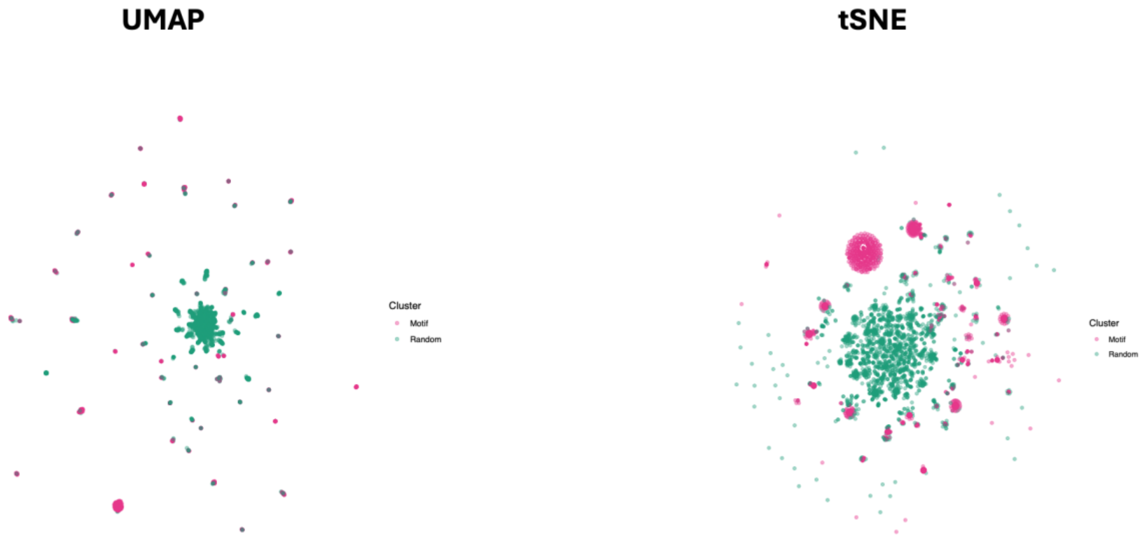

Figure 8: UMAP (left) and t-SNE (right) visualizations using the KMAP smoothed distance matrix.

As can be seen, the visualization quality is still suboptimal. This is likely due to the discrete nature of the distance matrix, which can cause gradient explosions during the optimization process, as discussed in the "*K*-mer Manifold Theory and KMAP Workflow" section in the manuscript.

3. Additional applications

3.1 KMAP vs MEME on ATAC-seq data

In HT-SELEX, we only compared the top motif, but it is unclear if KMAP generate similar results as MEME if we try to find multiple motifs.

To assess the performance of KMAP for identifying multiple motifs, we applied KMAP and MEME to H3K27ac ChIP-seq data, two ATAC-seq datasets, and an additional ChIP-seq dataset, as referenced below:

- REF1 (1 ATAC-seq + 1 ChIP-seq datasets): ENCODE Project Consortium. An integrated encyclopedia of DNA elements in the human genome. Nature 2012 Sep 6;489(7414):57-74. PMID: 22955616
- REF2 (1 ATAC-seq dataset): Davie K, Jacobs J, Atkins M, Potier D et al. Discovery of transcription factors and regulatory regions driving in vivo tumor development

by ATAC-seq and FAIRE-seq open chromatin profiling. PLoS Genet 2015 Feb;11(2)
. PMID: 25679813

For each dataset, we identified the top 5 motifs using both KMAP and MEME. In Figure 9, we show the motifs detected by KMAP and MEME from the differential enhancer regions of "EW8_dTAG_pair2". Both methods identified very similar motifs, though MEME missed the FLI1 motif, which appears as GGAA repeats.

Figures 10–12 present the top five motifs identified by KMAP and MEME in three additional datasets. The similarity in motif detection between the two methods across these ATAC-seq and ChIP-seq datasets suggests comparable motif identification performance for both tools.

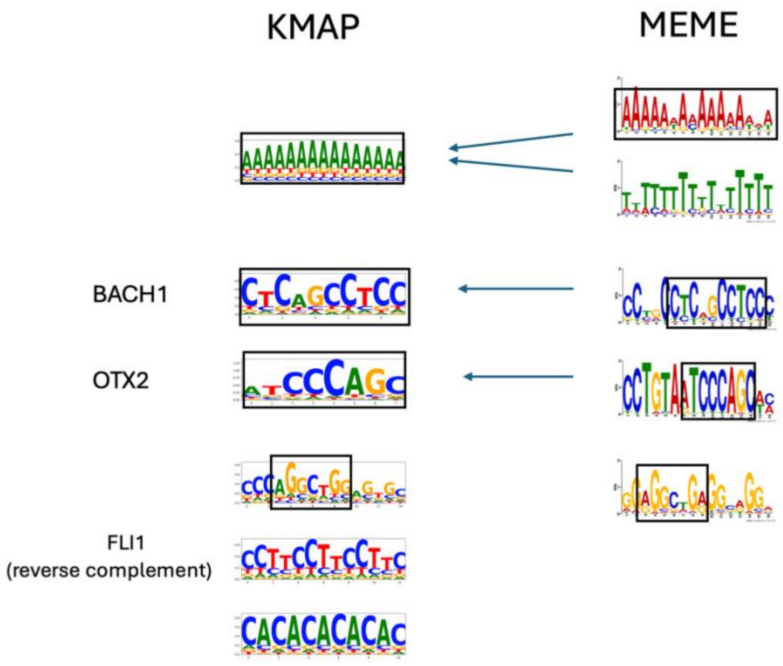

Figure 9: Top motifs detected by KMAP and MEME in H3K27ac ChIP-seq data (differential enhancer regions of "EW8_dTAG_pair2").

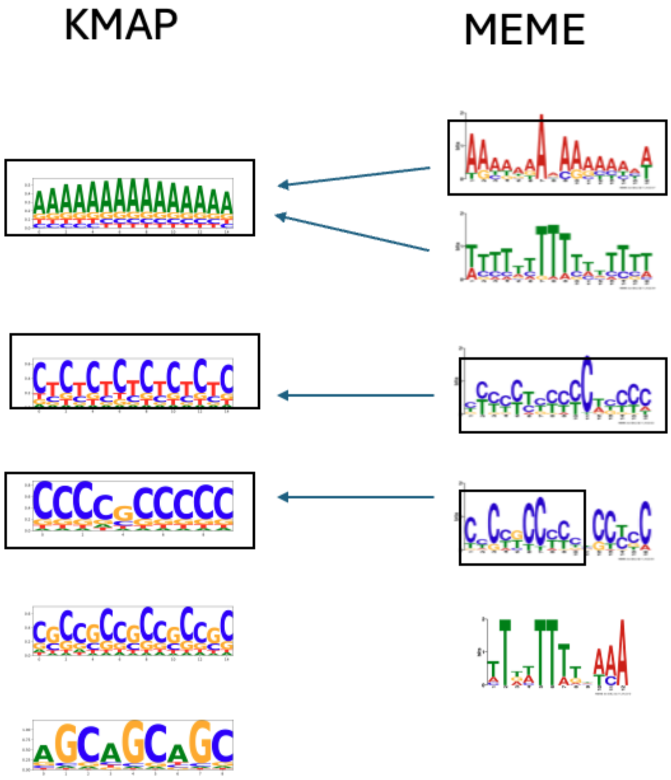

Figure 10: Top 5 motifs identified by KMAP and MEME in ATAC-seq dataset 1 (GSM6637886), generated from the psoas muscle tissue of a 16-year-old female (Homo sapiens) by the ENCODE Project Consortium [REF1].

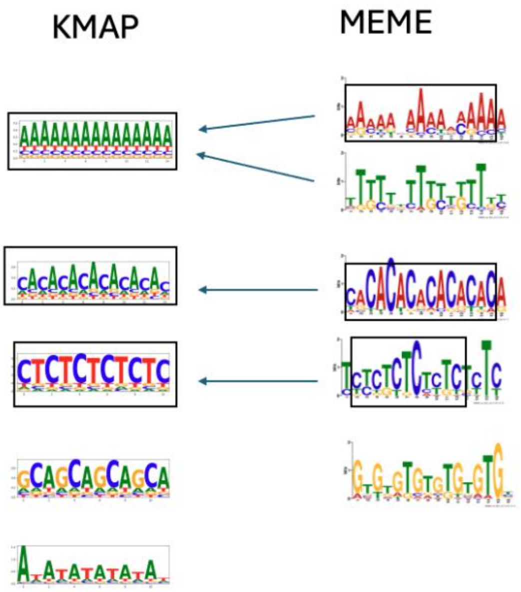

Figure 11: Top 5 motifs identified by KMAP and MEME in ATAC-seq dataset 2 (GSM1426259), generated from the Eye-Antennal disc of *Drosophila melanogaster* by Davie et al. [REF2].

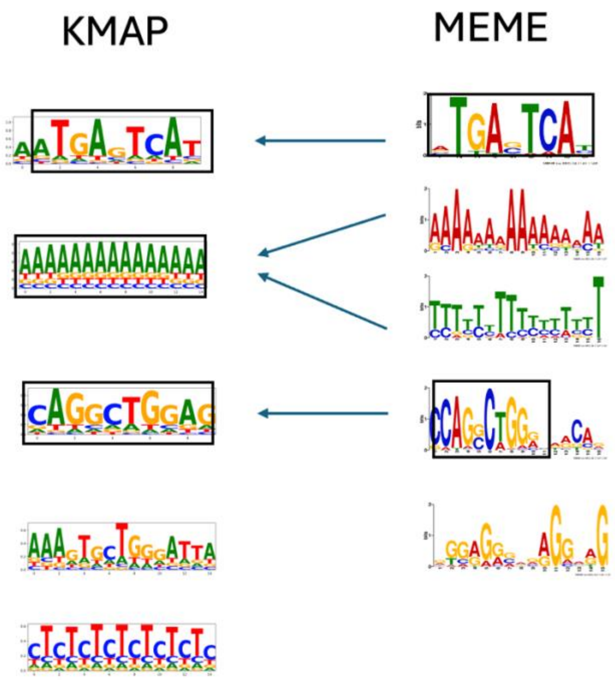

Figure 12: Top 5 motifs identified by KMAP and MEME in an AP1 ChIP-seq dataset (ENCFF624TOT), generated from umbilical vein endothelial cells of a newborn (Homo sapiens) by the ENCODE Project Consortium [REF1].

3.2 Composite motif analysis

One reviewer asked if KMAP could be used to identify composite motifs.

We applied KMAP to a dataset with known composite motifs (Fig. 2 of Jolma et al., *DNA-dependent formation of transcription factor pairs alters their binding specificity*, *Nature* 527, 384–388, 2015. https://doi.org/10.1038/nature15518). In the original study, the authors identified a composite motif of GCM1 and ELK1. As shown in Figure 13, KMAP successfully identified this composite motif, reproducing the original finding. This demonstrates that KMAP can be used to detect composite motifs.

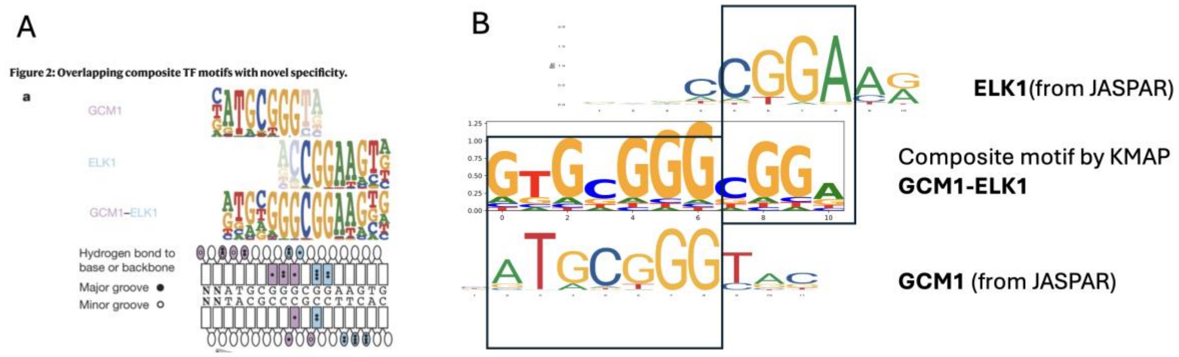

Figure 13: Composition motif identification with KMAP. (A) Composite motif from the original paper (Fig. 2 of Jolma et al.). (B) KMAP results. The top motif identified by KMAP is shown in the center panel and represents a composite motif of GCM1 and ELK1. JASPAR motifs of GCM1 and ELK1 are shown in the top and bottom panels for reference.

3.3 CTCF ChIP-seq data analysis

One reviwer asked us to do an additonal case study on CTCF.

We have downloaded the following CTCF ChIP-seq data from ENCODE and performed KMAP analysis: https://www.encodeproject.org/experiments/ENCSR877MSN/.

Figure 14 shows the motifs detected by KMAP. Motif 4 (AGCCACCA) corresponds to the first part of the CTCF motif from JASPAR, while Motif 2 (CCAGAAGAGGGCA) aligns with the second part of the CTCF motif. Both motifs appear centrally within the sequences and at high frequencies, with Motif 4 (AGCCACCA, 87.1%) slightly preceding Motif 2 (CCAGAAGAGGGCA, 93.3%). These results indicate that KMAP's findings are consistent with established knowledge about CTCF binding motifs.

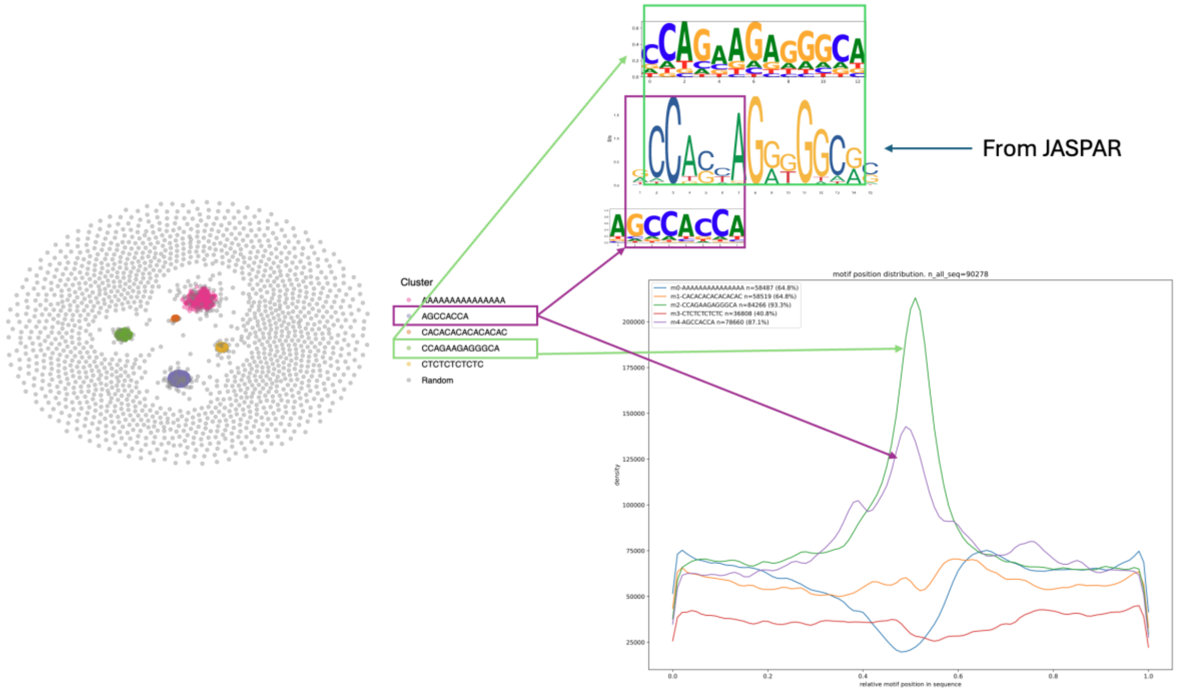

Figure 14: KMAP results on CTCF ChIP-seq data from ENCODE. (Top) 2D visualizations and KMAP motif logos, with the central motif taken from JASPAR for reference. (Bottom) Motif position distributions.
