# Supplementary figures and images for: k-mer manifold approximation and projection for visualizing DNA sequences (part 1 of 2)
Source: Genome Res. 2025 May;35(5):1234–46. doi: 10.1101/gr.279458.124 (PMC12047656; doi:10.1101/gr.279458.124)

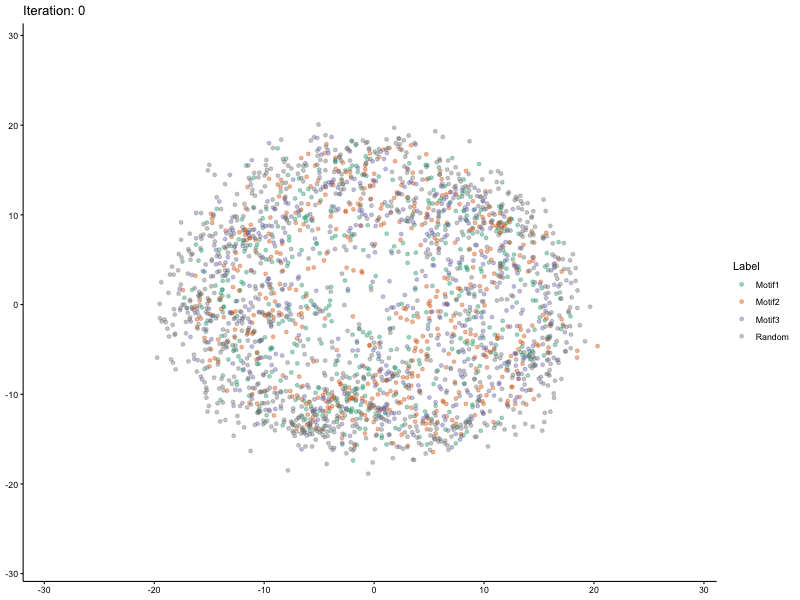

Supplement: Supplement 7 [file Supplemental_code.zip › Supplemental code/kmap_pacakge/kmap_cartoon.gif]

KMAP LD Plot - Alx1\_TAAAGC20NCG\_Z\_3

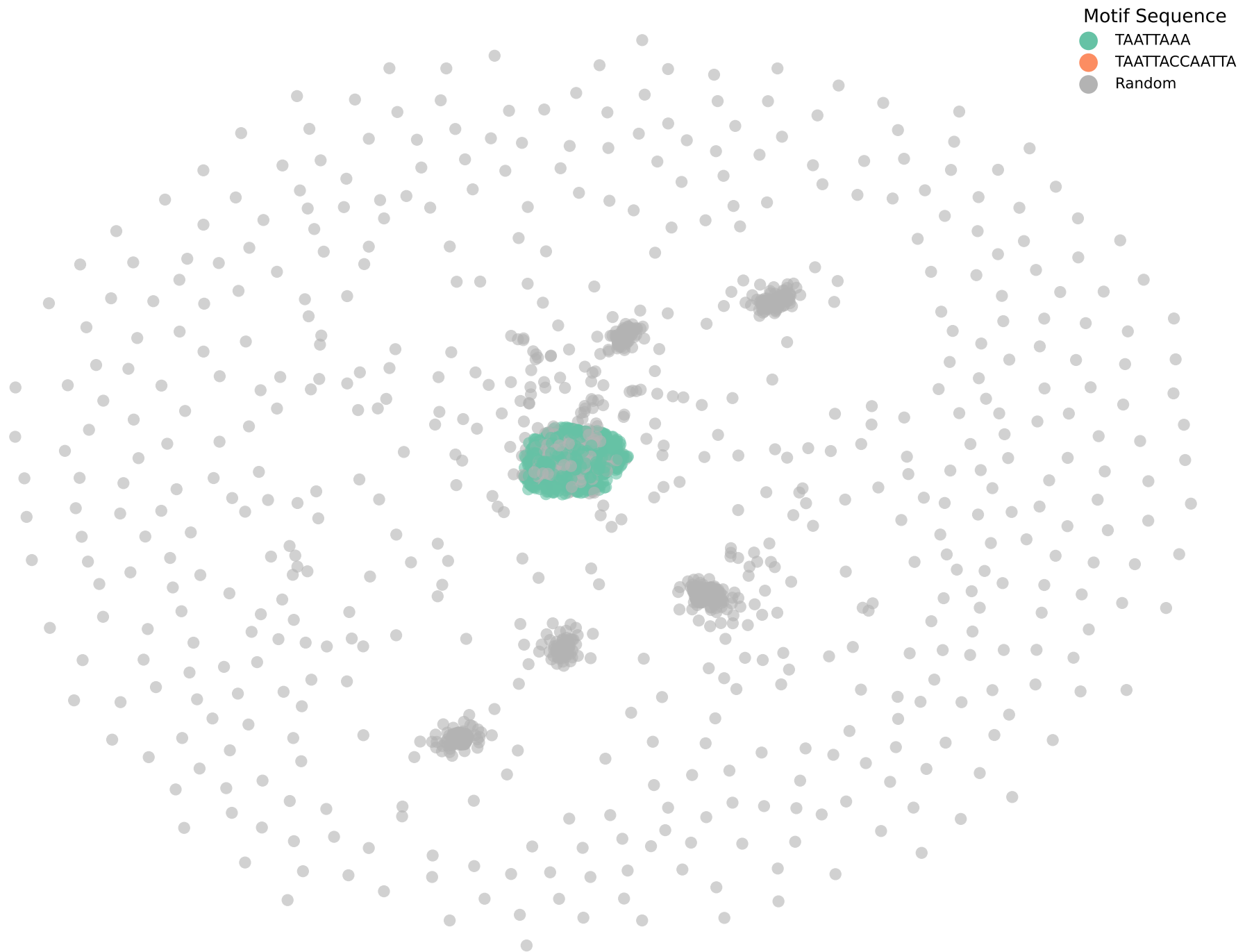

Supplement: Supplement 8 [file Supplemental_Data_1.zip › Supplemental_Data_1/Alx1_TAAAGC20NCG_Z_3/Alx1_TAAAGC20NCG_Z_3_KMAP.pdf]

MDS Plot - Alx1\_TAAAGC20NCG\_Z\_3

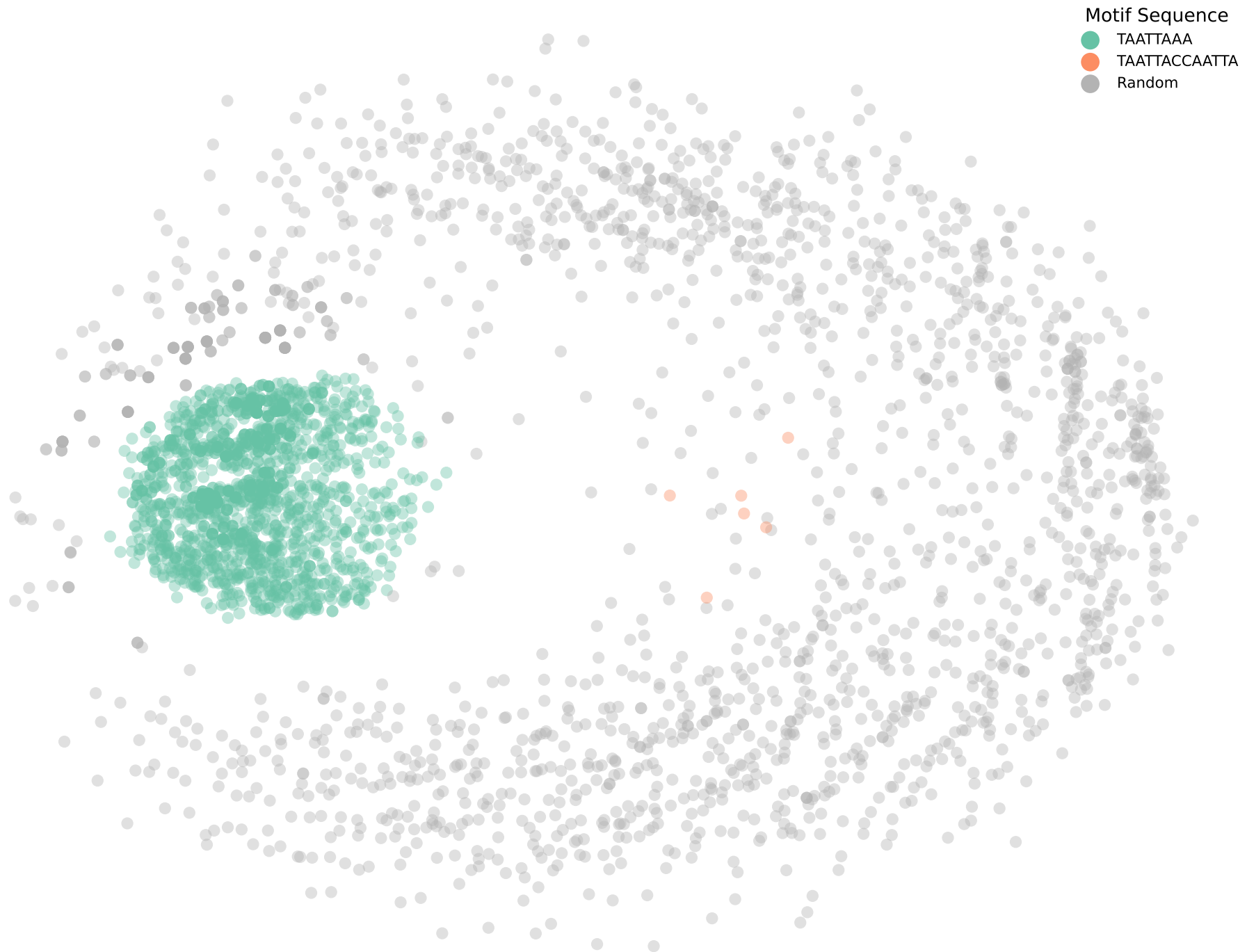

Supplement: Supplement 8 [file Supplemental_Data_1.zip › Supplemental_Data_1/Alx1_TAAAGC20NCG_Z_3/Alx1_TAAAGC20NCG_Z_3_MDS.pdf]

PCA Plot - A1x1\_TAAAGC20NCG\_Z\_3

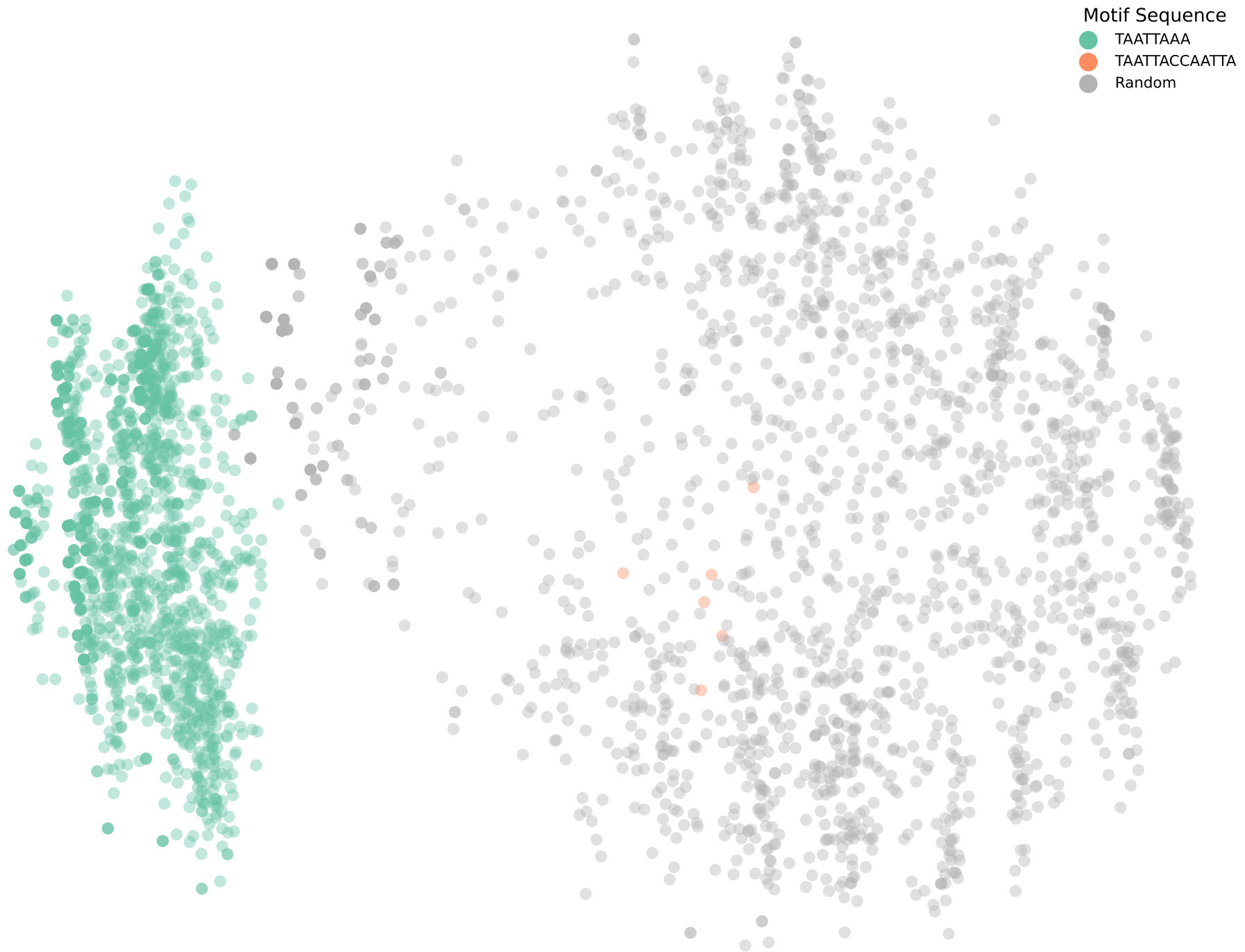

Supplement: Supplement 8 [file Supplemental_Data_1.zip › Supplemental_Data_1/Alx1_TAAAGC20NCG_Z_3/Alx1_TAAAGC20NCG_Z_3_PCA.pdf]

tSNE Plot - Alx1\_TAAAGC20NCG\_Z\_3

Motif Sequence

- TAATTAAA
- TAATTACCAATTA
- Random

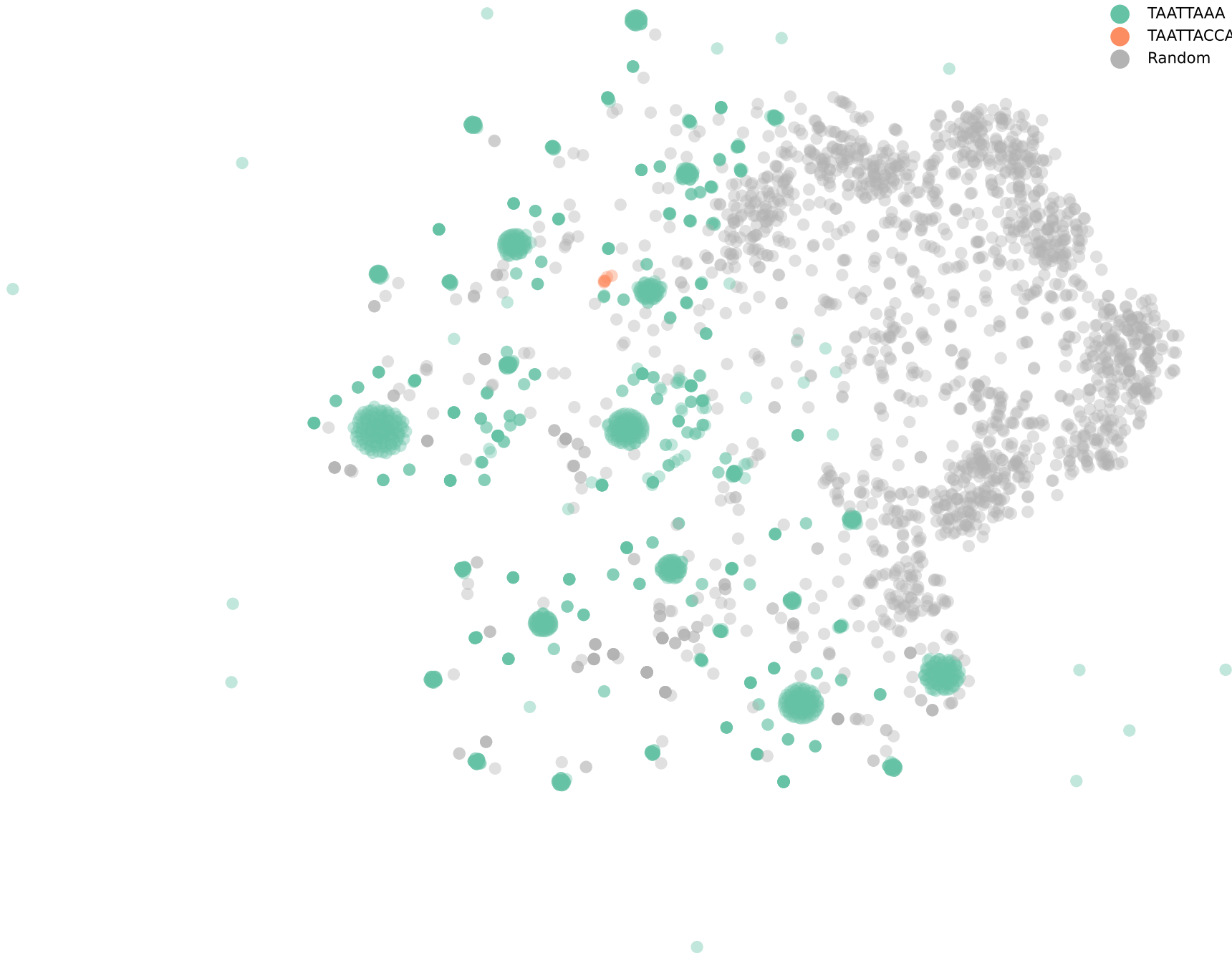

Supplement: Supplement 8 [file Supplemental_Data_1.zip › Supplemental_Data_1/Alx1_TAAAGC20NCG_Z_3/Alx1_TAAAGC20NCG_Z_3_tSNE.pdf]

UMAP Plot - Alx1\_TAAAGC20NCG\_Z\_3

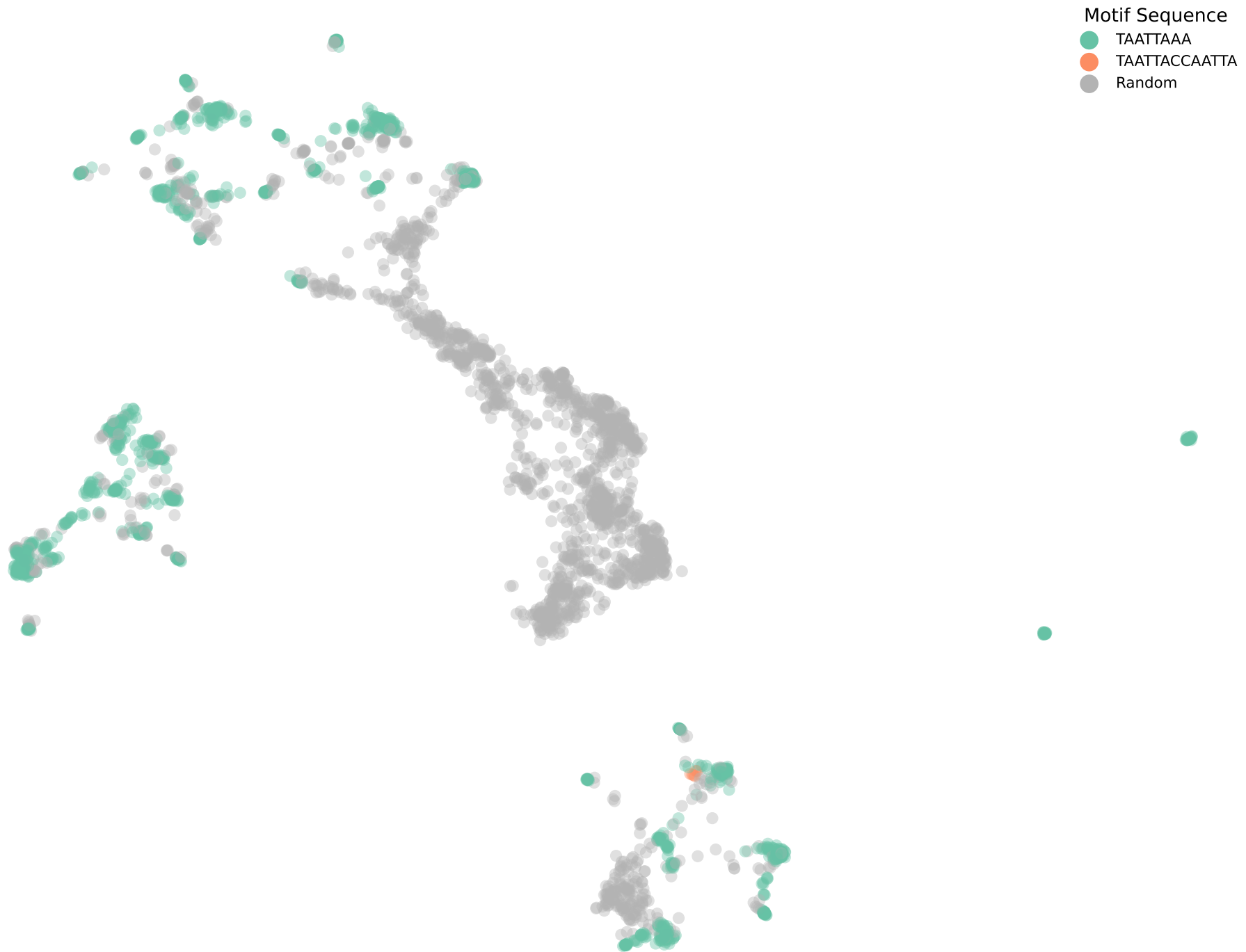

Supplement: Supplement 8 [file Supplemental_Data_1.zip › Supplemental_Data_1/Alx1_TAAAGC20NCG_Z_3/Alx1_TAAAGC20NCG_Z_3_UMAP.pdf]

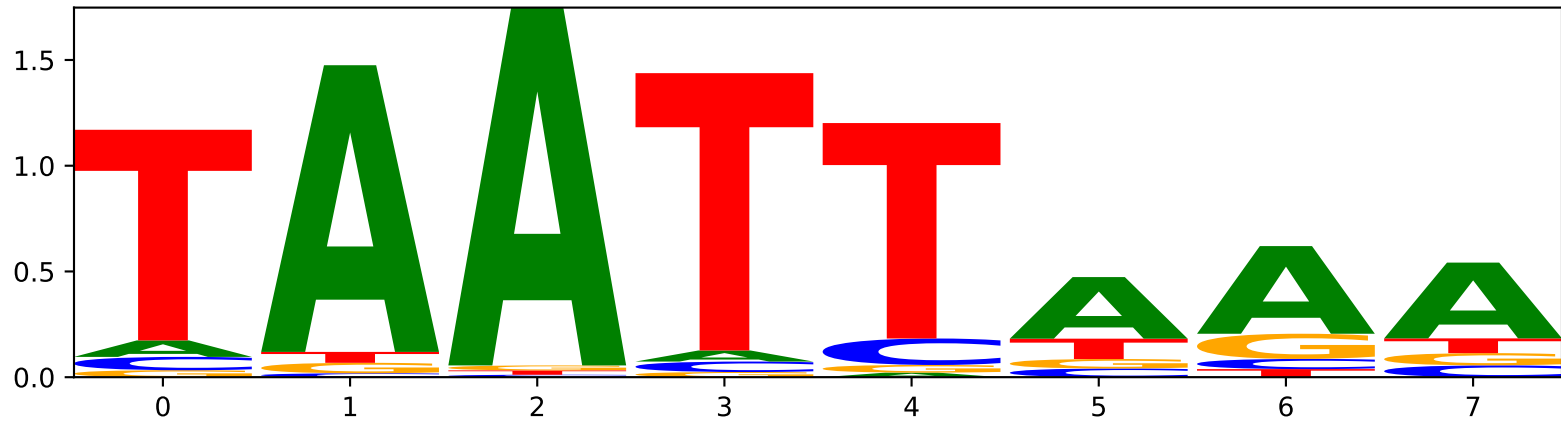

Supplement: Supplement 8 [file Supplemental_Data_1.zip › Supplemental_Data_1/Alx1_TAAAGC20NCG_Z_3/kmap_logo.pdf]

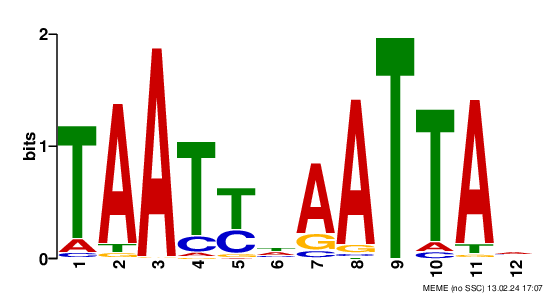

Supplement: Supplement 8 [file Supplemental_Data_1.zip › Supplemental_Data_1/Alx1_TAAAGC20NCG_Z_3/meme_logo.png]

KMAP LD Plot - Alx1\_TAAAGC20NCG\_Z\_4

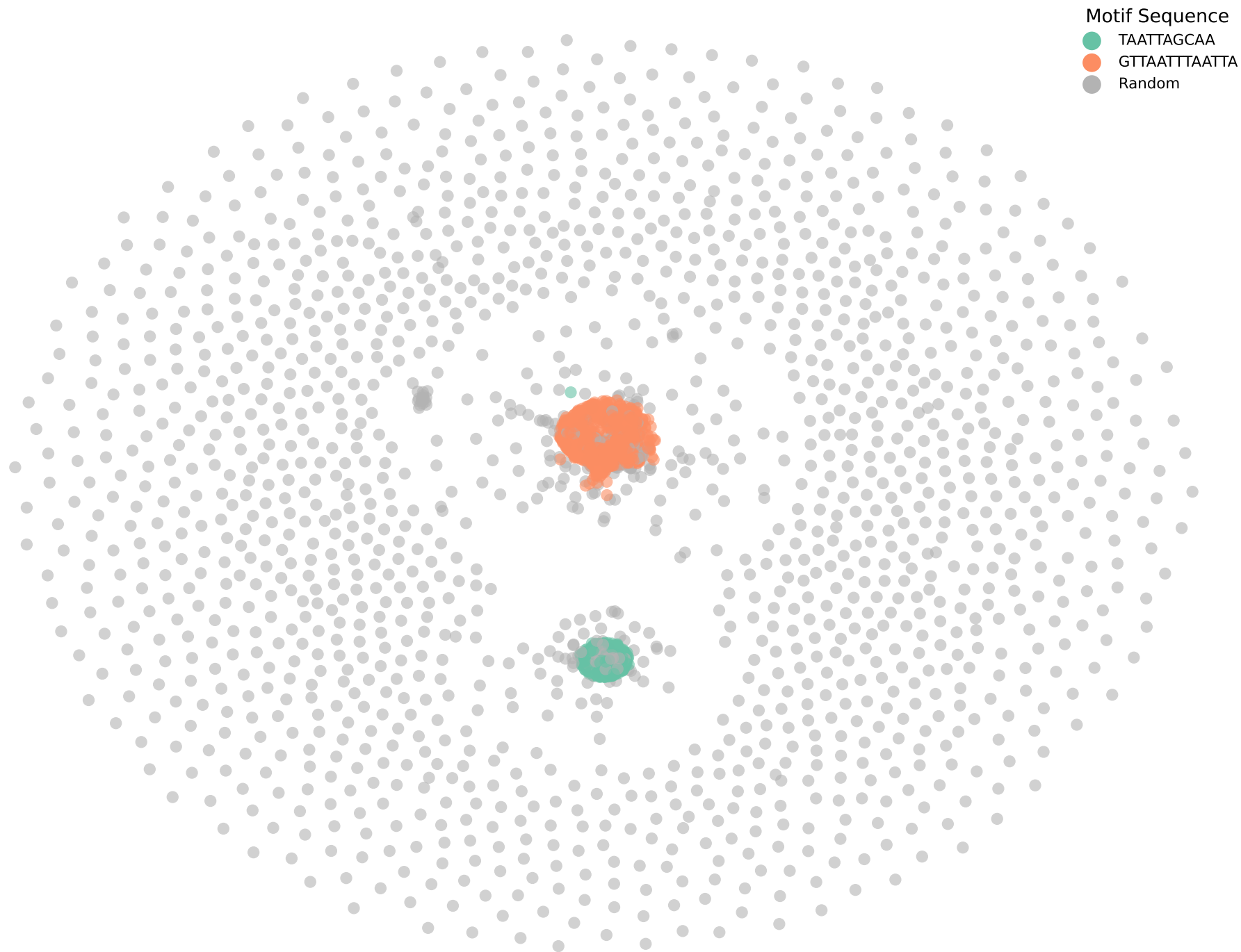

Supplement: Supplement 8 [file Supplemental_Data_1.zip › Supplemental_Data_1/Alx1_TAAAGC20NCG_Z_4/Alx1_TAAAGC20NCG_Z_4_KMAP.pdf]

MDS Plot - Alx1\_TAAAGC20NCG\_Z\_4

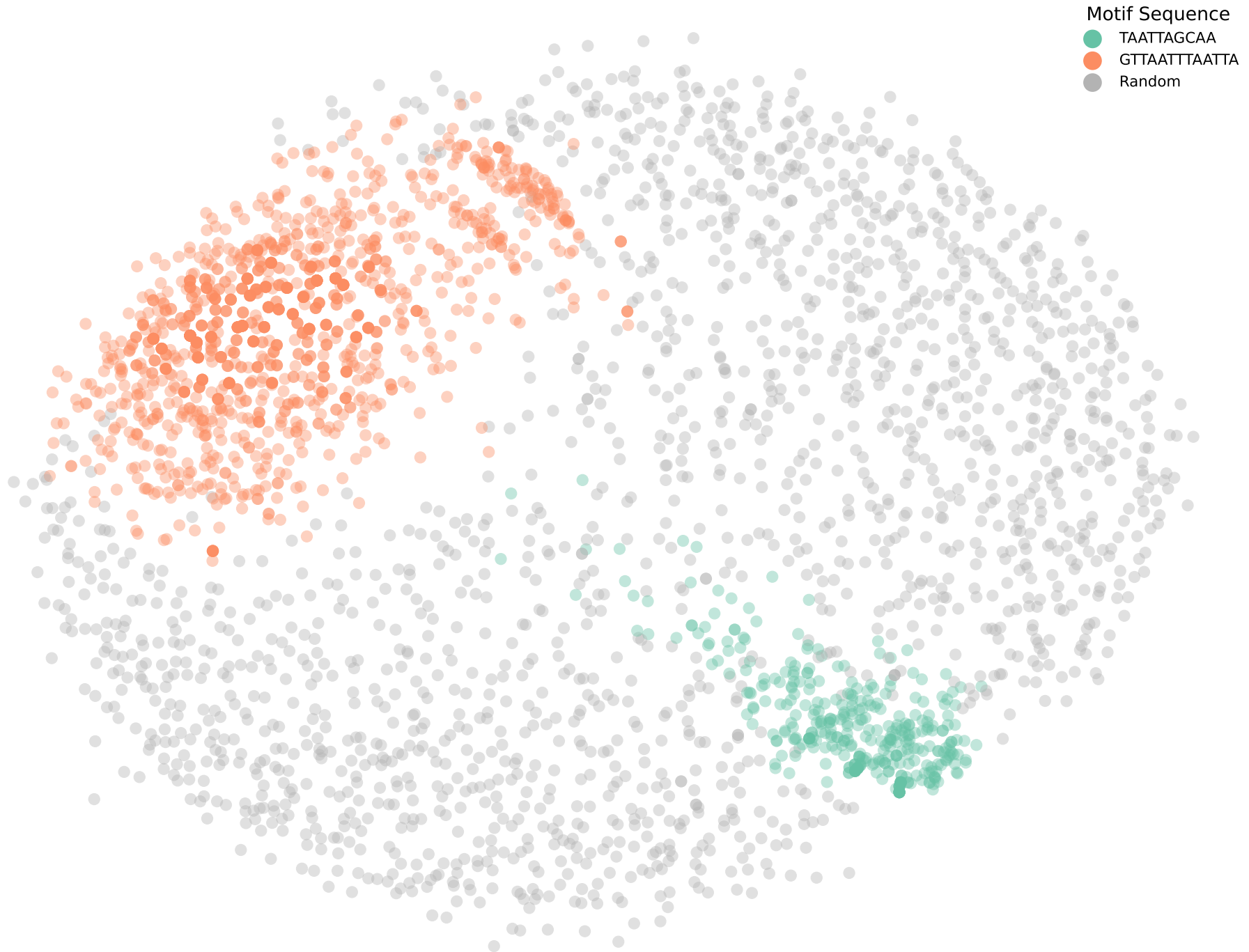

Supplement: Supplement 8 [file Supplemental_Data_1.zip › Supplemental_Data_1/Alx1_TAAAGC20NCG_Z_4/Alx1_TAAAGC20NCG_Z_4_MDS.pdf]

PCA Plot - Alx1\_TAAAGC20NCG\_Z\_4

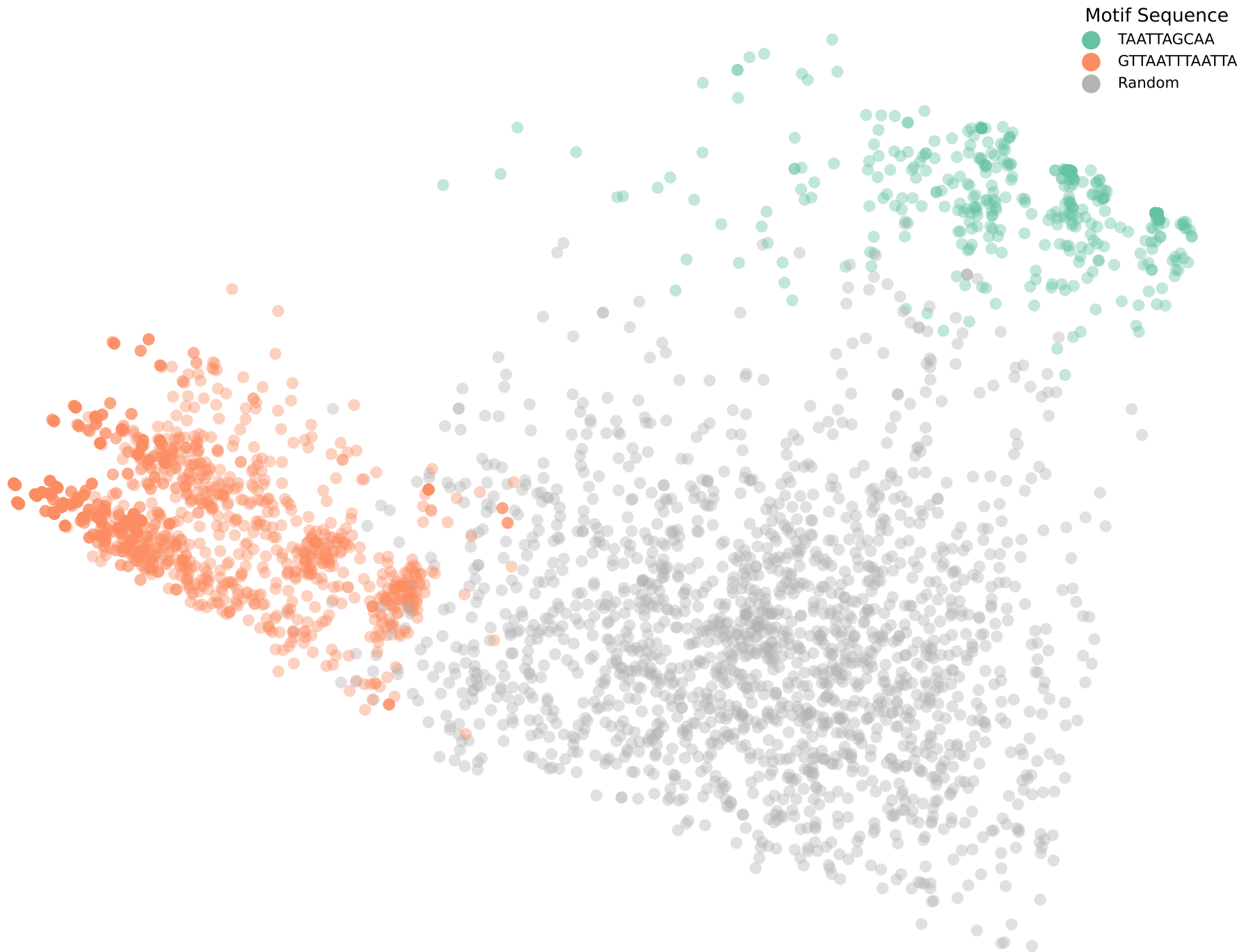

Supplement: Supplement 8 [file Supplemental_Data_1.zip › Supplemental_Data_1/Alx1_TAAAGC20NCG_Z_4/Alx1_TAAAGC20NCG_Z_4_PCA.pdf]

tSNE Plot - Alx1\_TAAAGC20NCG\_Z\_4

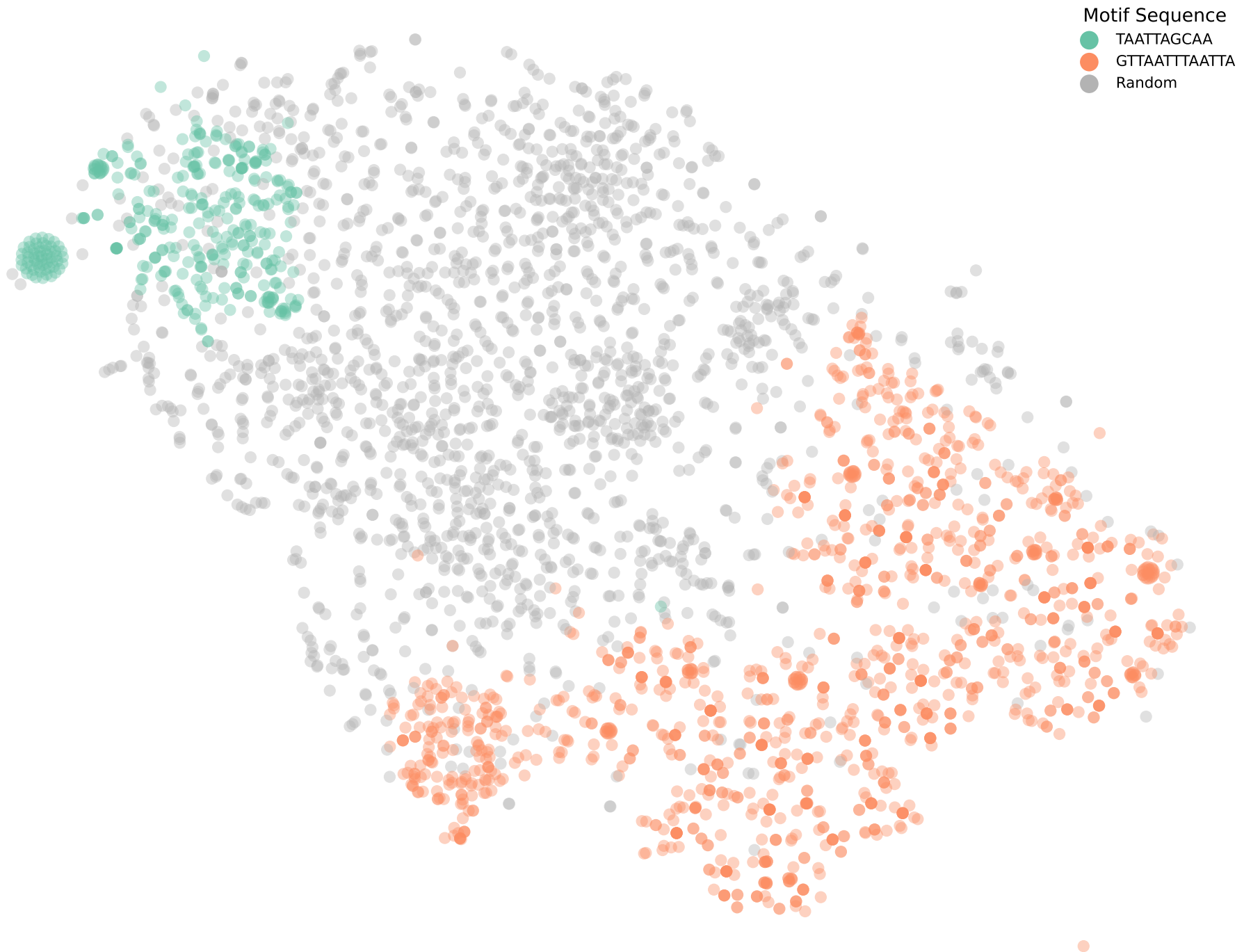

Supplement: Supplement 8 [file Supplemental_Data_1.zip › Supplemental_Data_1/Alx1_TAAAGC20NCG_Z_4/Alx1_TAAAGC20NCG_Z_4_tSNE.pdf]

UMAP Plot - Alx1\_TAAAGC20NCG\_Z\_4

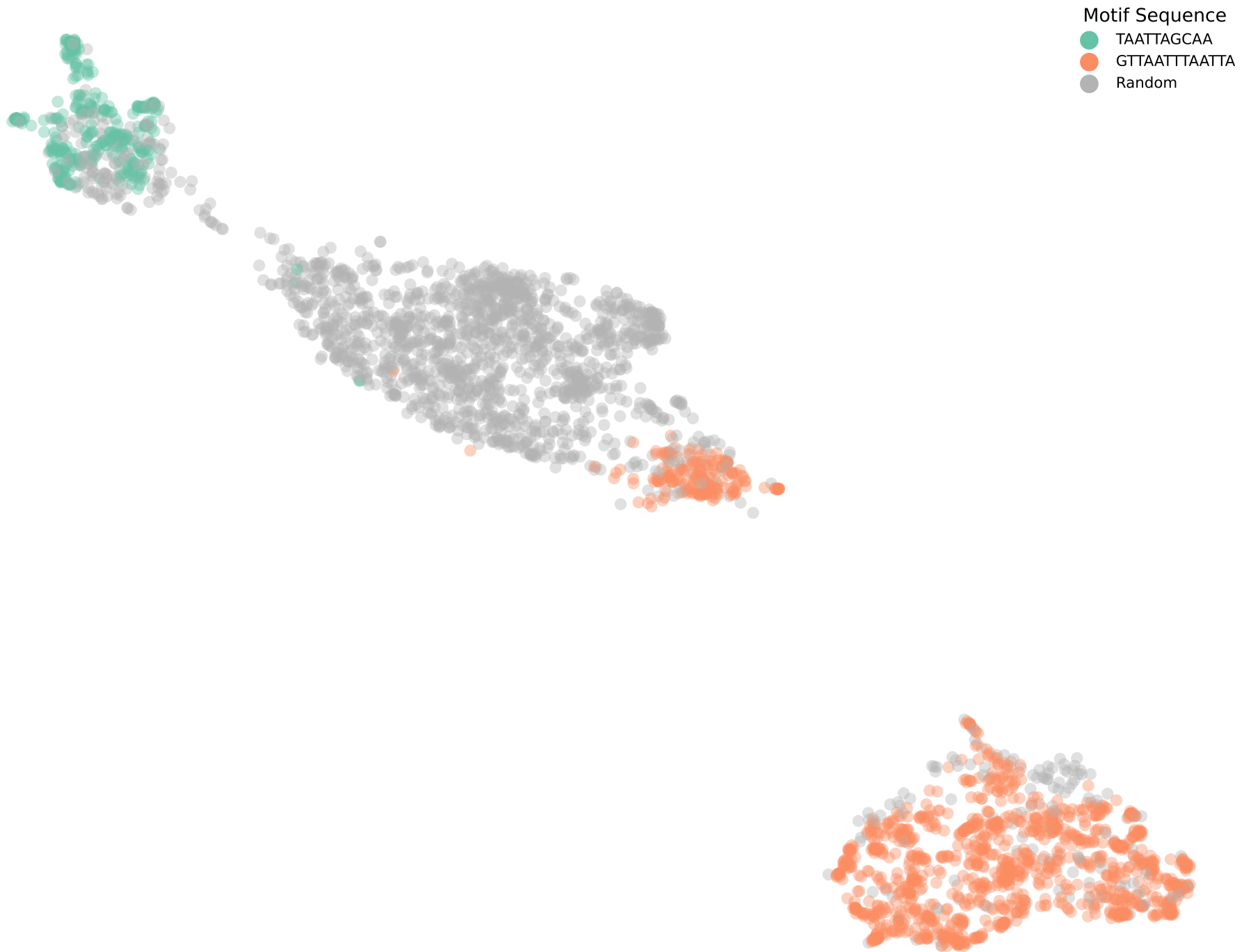

Supplement: Supplement 8 [file Supplemental_Data_1.zip › Supplemental_Data_1/Alx1_TAAAGC20NCG_Z_4/Alx1_TAAAGC20NCG_Z_4_UMAP.pdf]

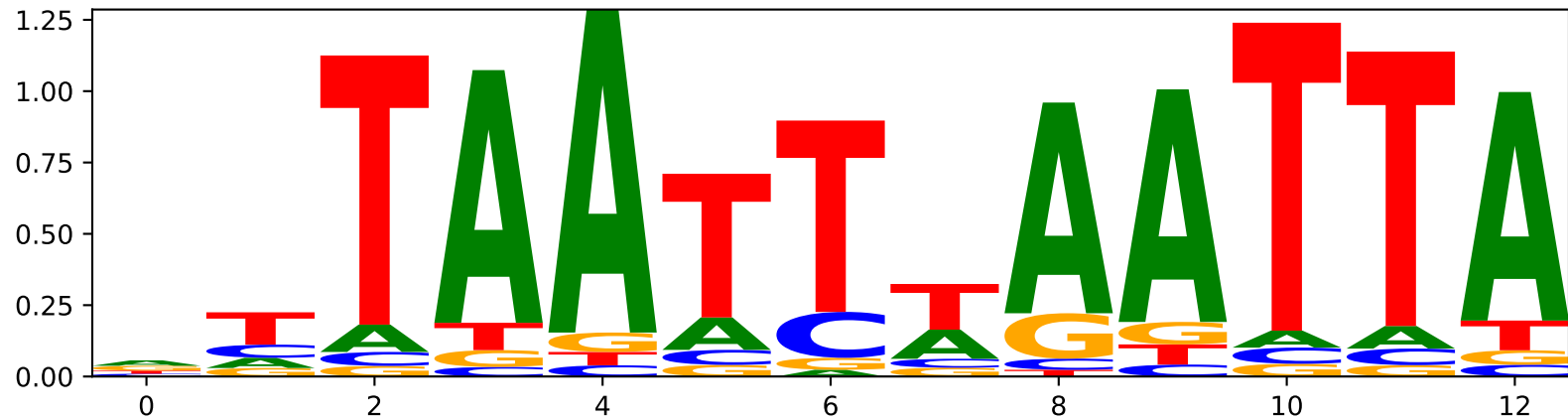

Supplement: Supplement 8 [file Supplemental_Data_1.zip › Supplemental_Data_1/Alx1_TAAAGC20NCG_Z_4/kmap_logo.pdf]

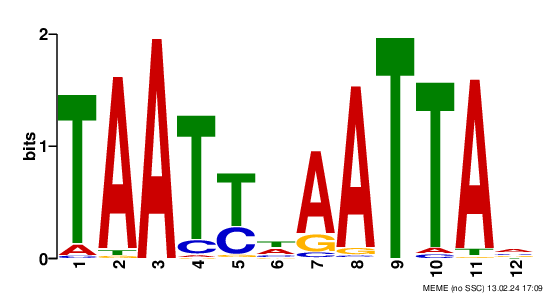

Supplement: Supplement 8 [file Supplemental_Data_1.zip › Supplemental_Data_1/Alx1_TAAAGC20NCG_Z_4/meme_logo.png]

KMAP LD Plot - ALX3\_TGCAAG20NGA\_AE\_3

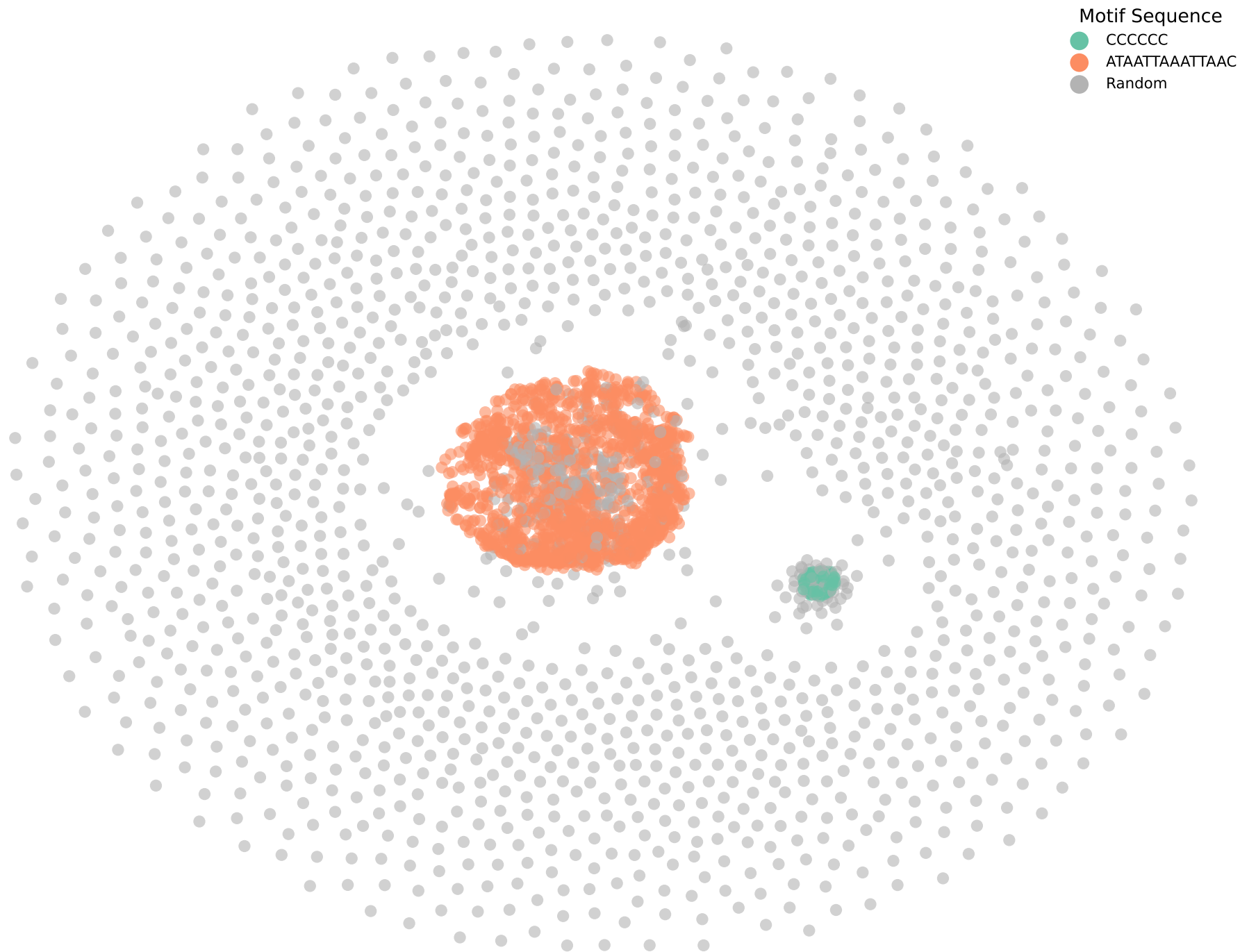

Supplement: Supplement 8 [file Supplemental_Data_1.zip › Supplemental_Data_1/ALX3_TGCAAG20NGA_AE_3/ALX3_TGCAAG20NGA_AE_3_KMAP.pdf]

MDS Plot - ALX3\_TGCAAG20NGA\_AE\_3

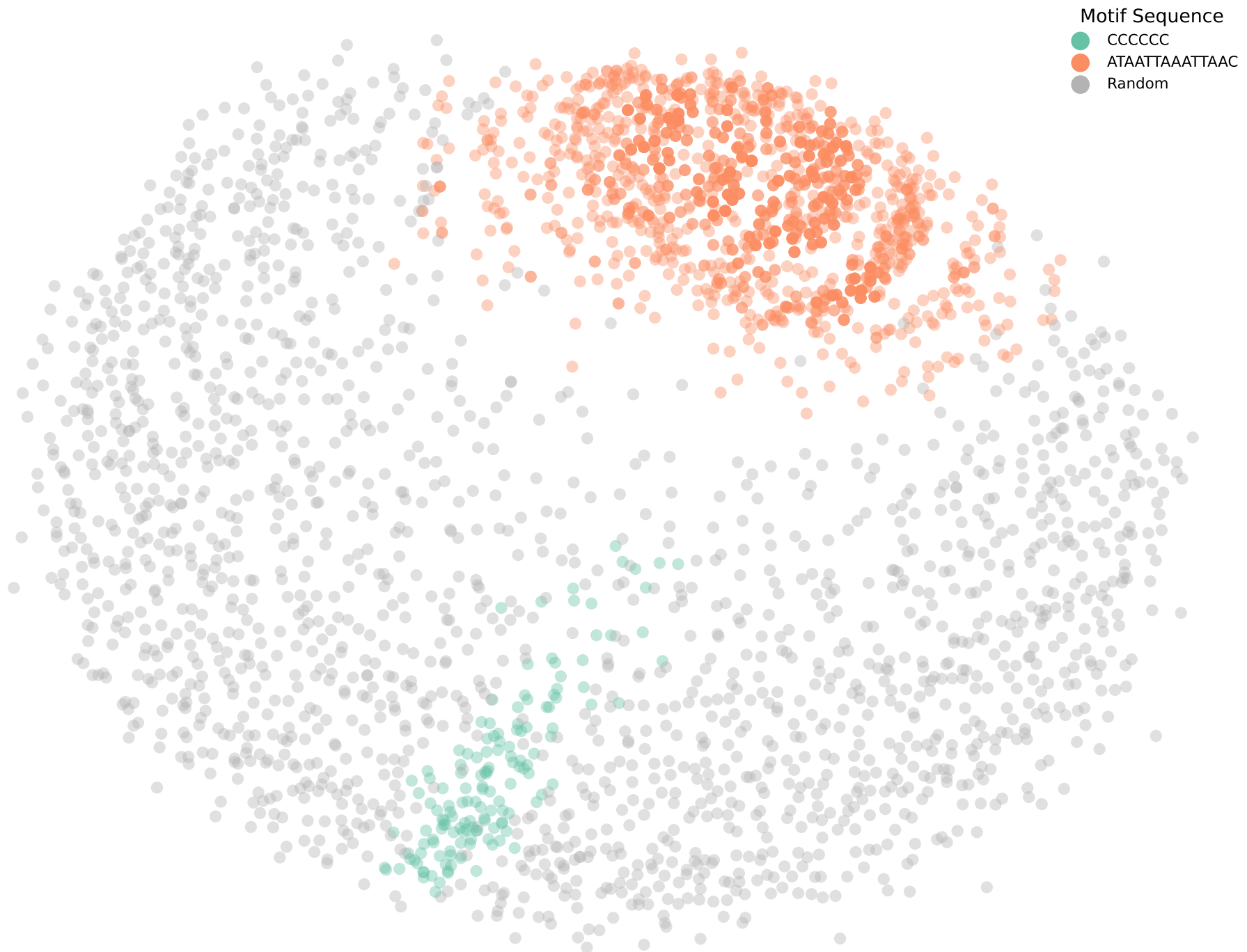

Supplement: Supplement 8 [file Supplemental_Data_1.zip › Supplemental_Data_1/ALX3_TGCAAG20NGA_AE_3/ALX3_TGCAAG20NGA_AE_3_MDS.pdf]

PCA Plot - ALX3\_TGCAAG20NGA\_AE\_3

Motif Sequence

- CCCCC
- ATAATTAAATTAAC
- Random

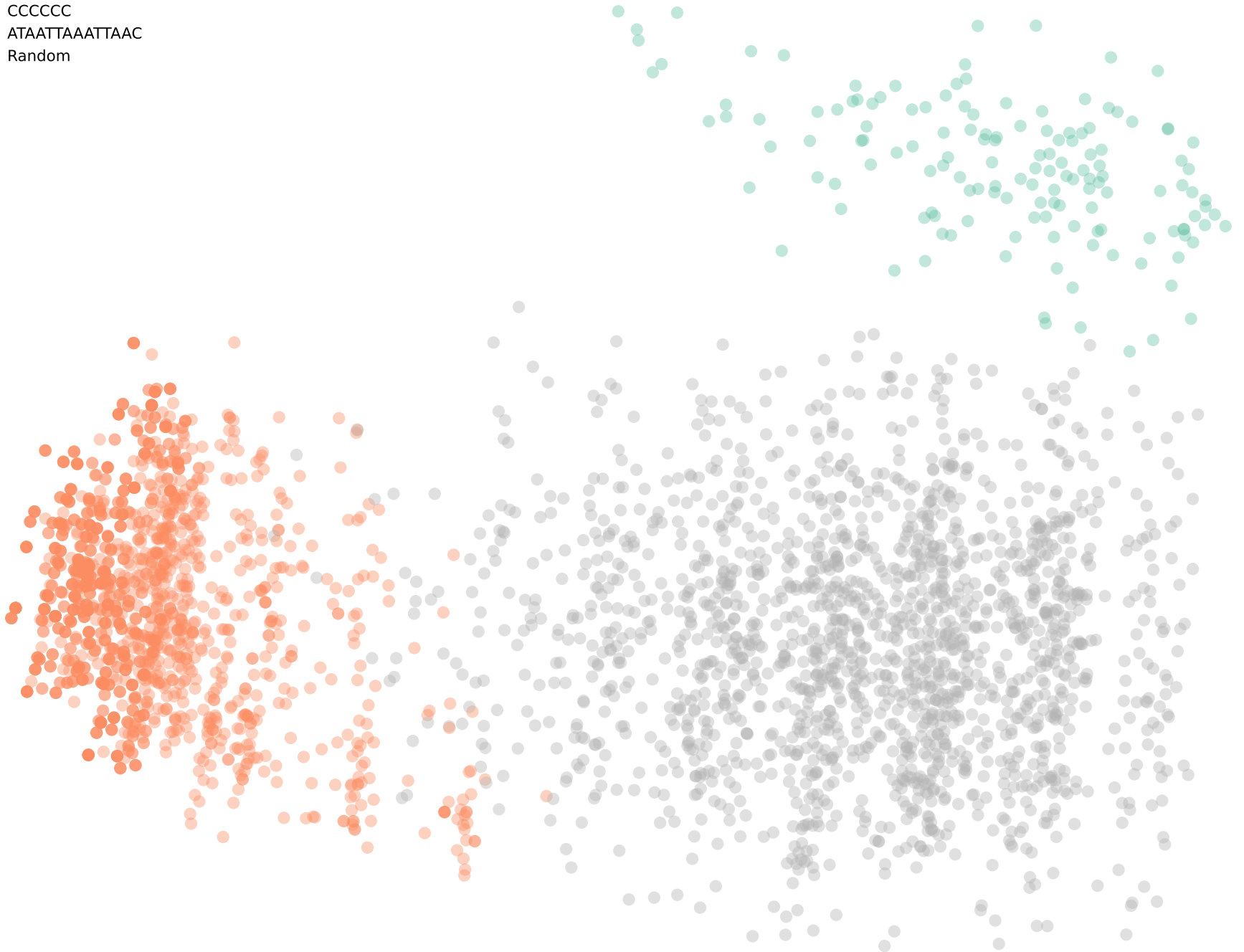

Supplement: Supplement 8 [file Supplemental_Data_1.zip › Supplemental_Data_1/ALX3_TGCAAG20NGA_AE_3/ALX3_TGCAAG20NGA_AE_3_PCA.pdf]

tSNE Plot - ALX3\_TGCAAG20NGA\_AE\_3

Motif Sequence

- CCCCC
- ATAATTAAATTAAC
- Random

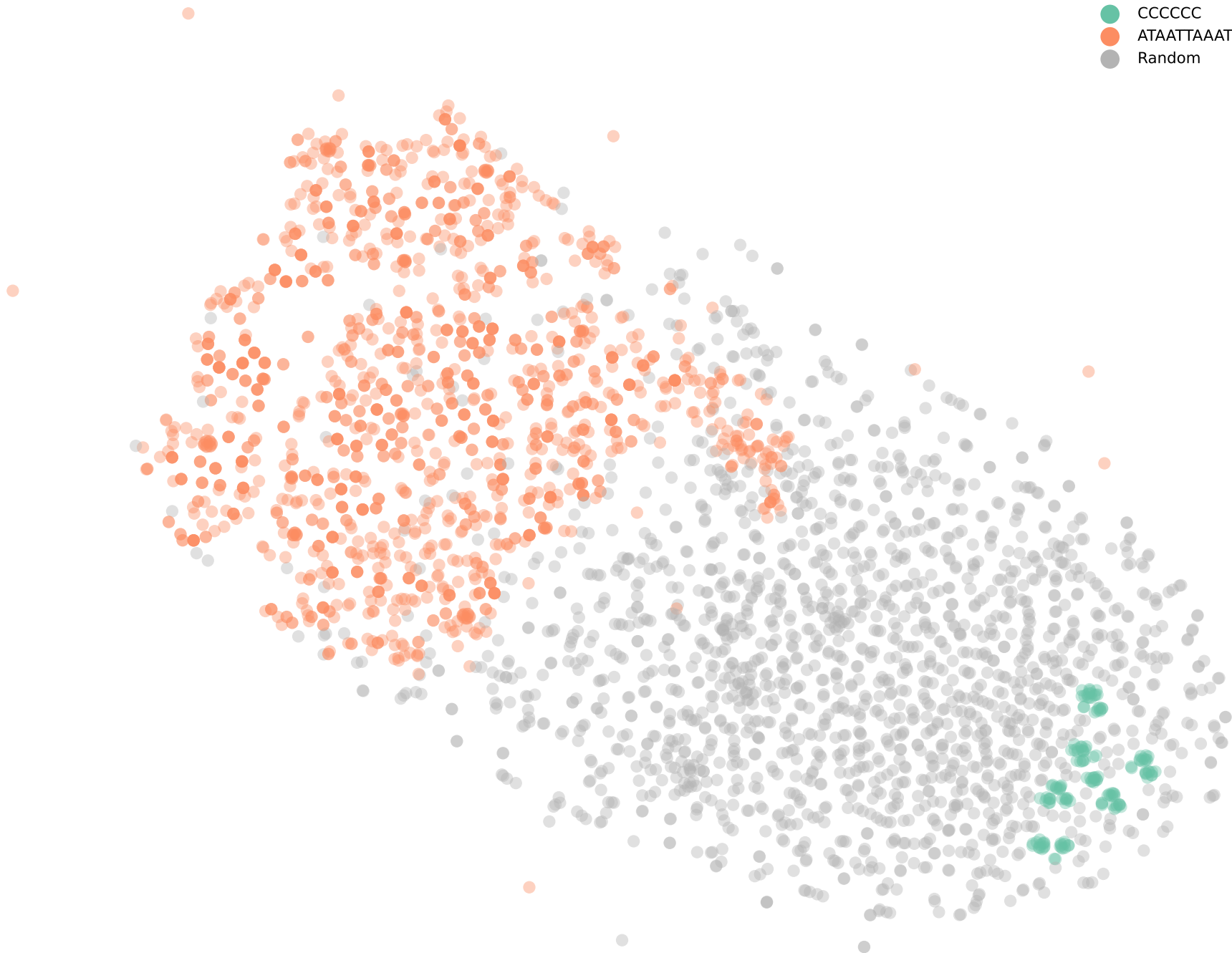

Supplement: Supplement 8 [file Supplemental_Data_1.zip › Supplemental_Data_1/ALX3_TGCAAG20NGA_AE_3/ALX3_TGCAAG20NGA_AE_3_tSNE.pdf]

UMAP Plot - ALX3\_TGCAAG20NGA\_AE\_3

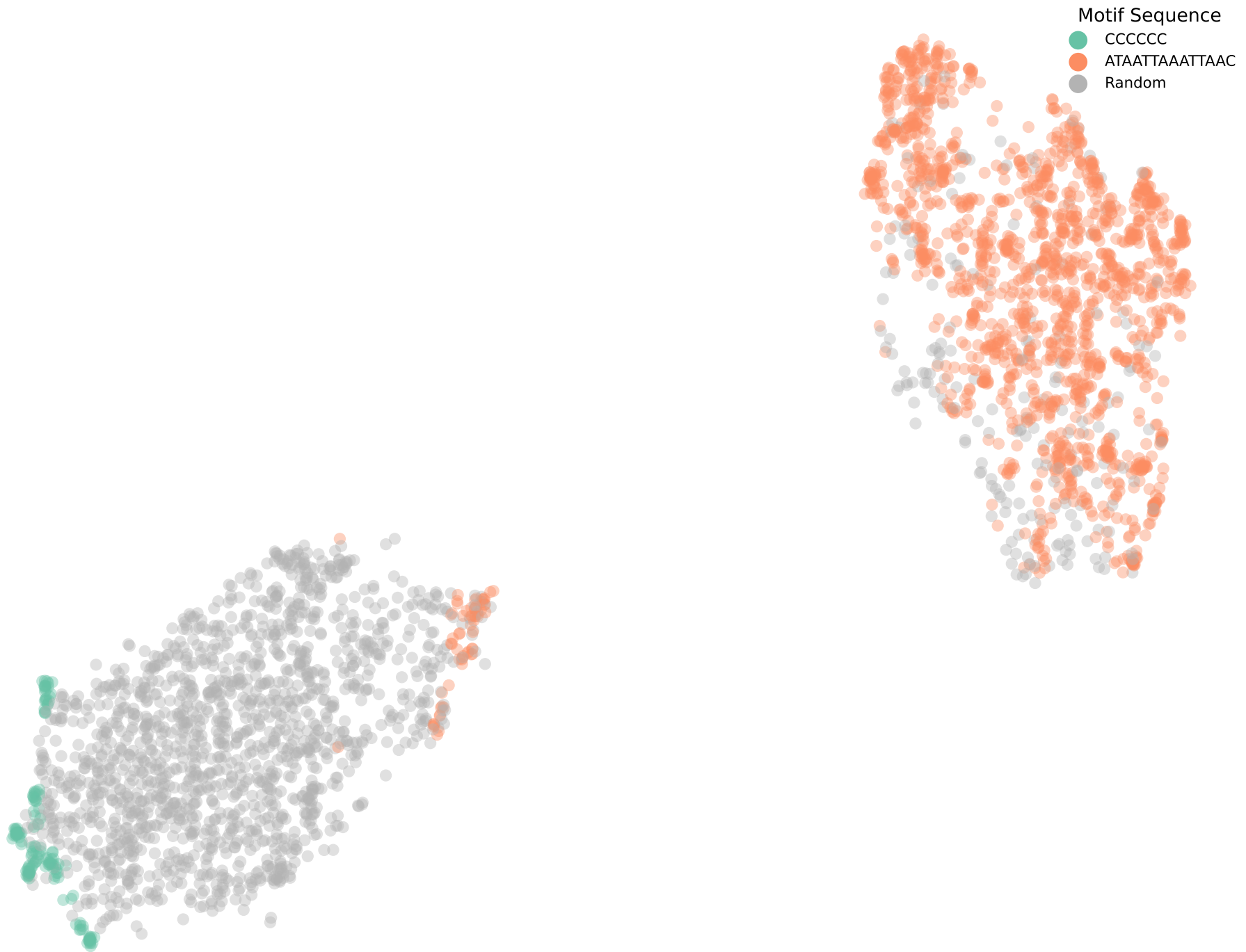

Supplement: Supplement 8 [file Supplemental_Data_1.zip › Supplemental_Data_1/ALX3_TGCAAG20NGA_AE_3/ALX3_TGCAAG20NGA_AE_3_UMAP.pdf]

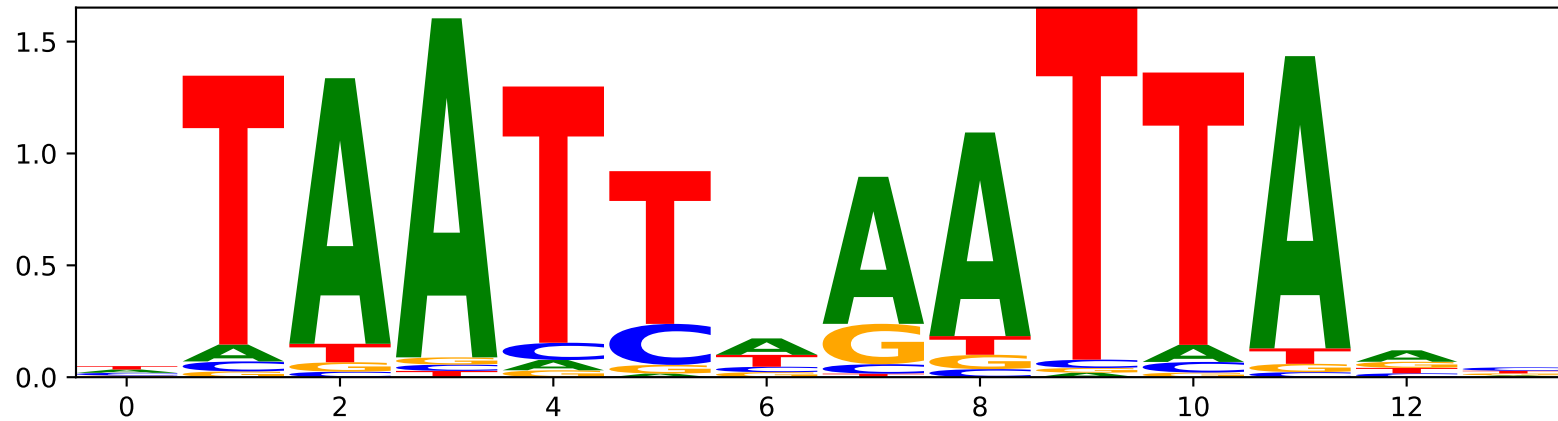

Supplement: Supplement 8 [file Supplemental_Data_1.zip › Supplemental_Data_1/ALX3_TGCAAG20NGA_AE_3/kmap_logo.pdf]

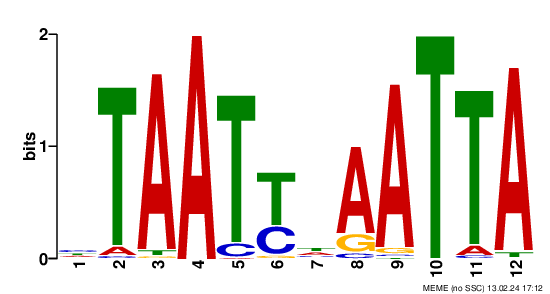

Supplement: Supplement 8 [file Supplemental_Data_1.zip › Supplemental_Data_1/ALX3_TGCAAG20NGA_AE_3/meme_logo.png]

KMAP LD Plot - ALX3\_TGCAAG20NGA\_AE\_4

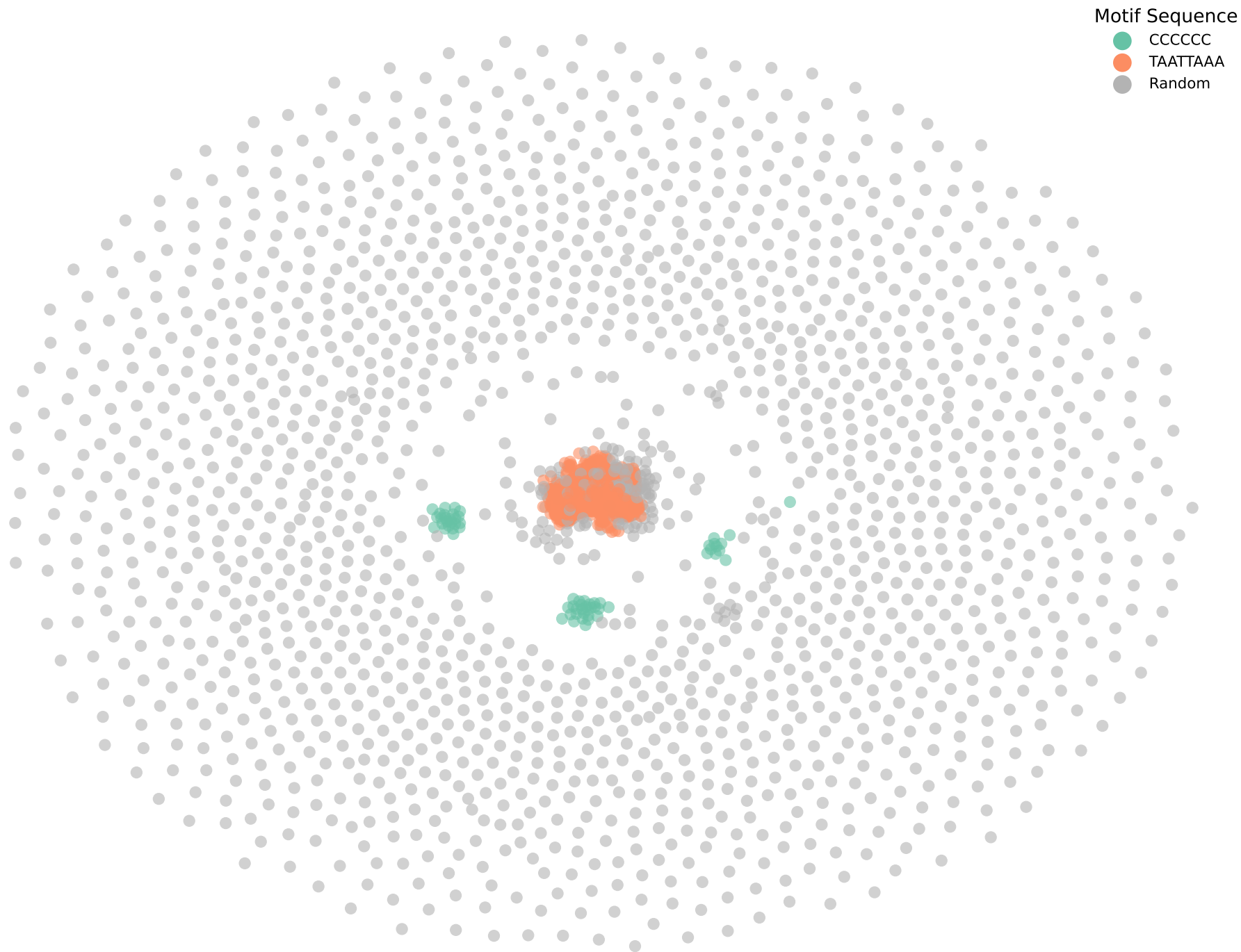

Supplement: Supplement 8 [file Supplemental_Data_1.zip › Supplemental_Data_1/ALX3_TGCAAG20NGA_AE_4/ALX3_TGCAAG20NGA_AE_4_KMAP.pdf]

MDS Plot - ALX3\_TGCAAG20NGA\_AE\_4

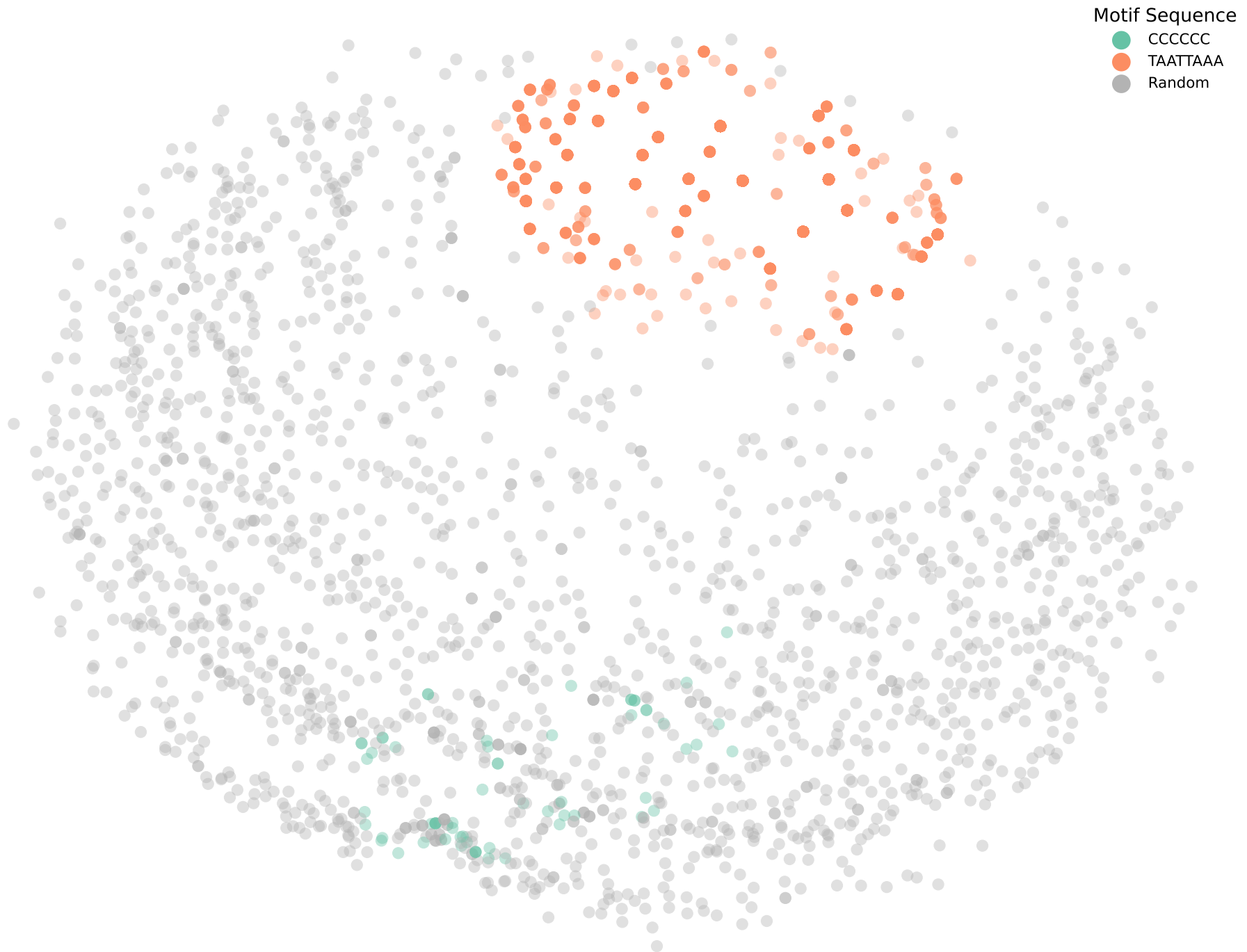

Supplement: Supplement 8 [file Supplemental_Data_1.zip › Supplemental_Data_1/ALX3_TGCAAG20NGA_AE_4/ALX3_TGCAAG20NGA_AE_4_MDS.pdf]

PCA Plot - ALX3\_TGCAAG20NGA\_AE\_4

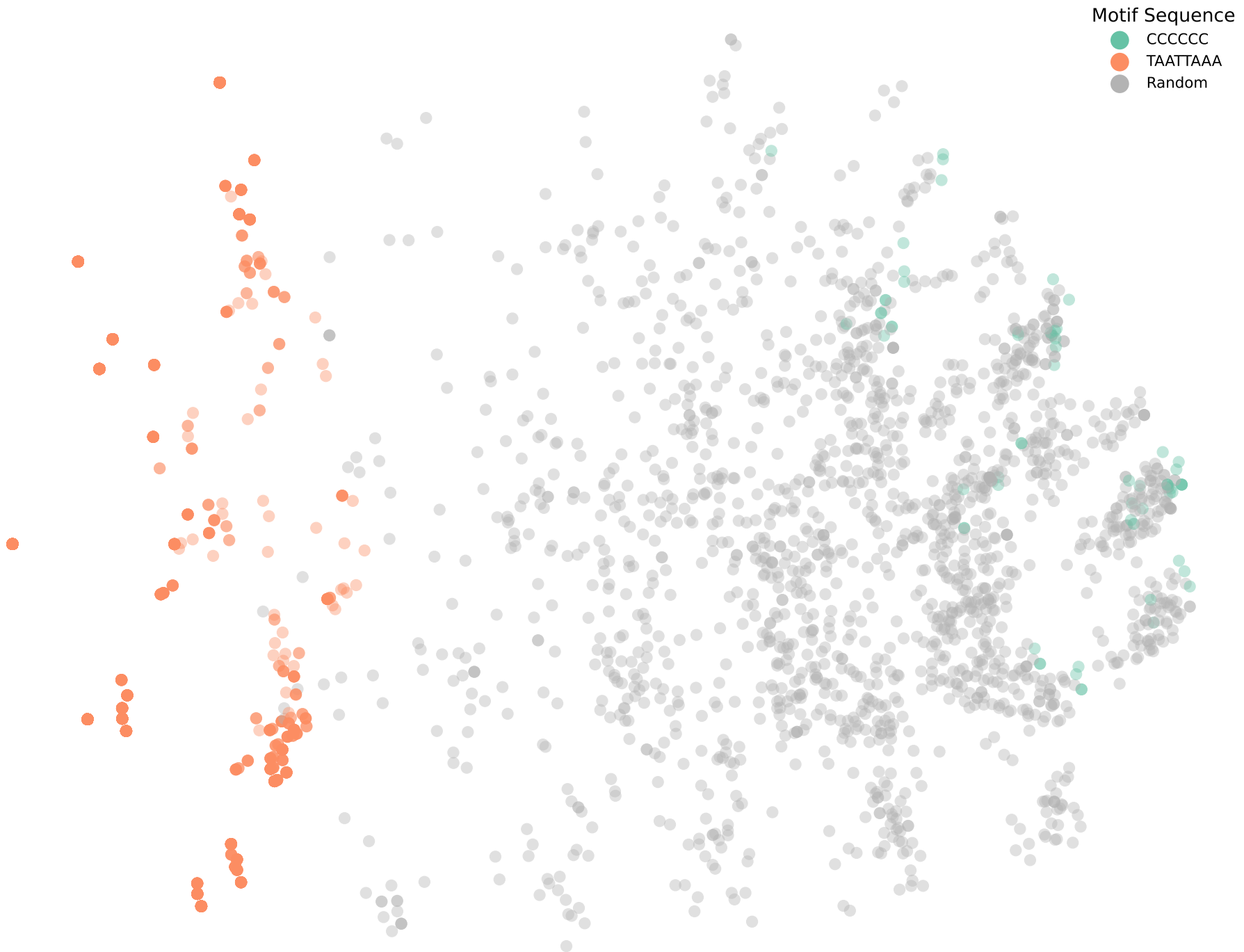

Supplement: Supplement 8 [file Supplemental_Data_1.zip › Supplemental_Data_1/ALX3_TGCAAG20NGA_AE_4/ALX3_TGCAAG20NGA_AE_4_PCA.pdf]

tSNE Plot - ALX3\_TGCAAG20NGA\_AE\_4

Motif Sequence

- CCCCC
- TAATTAAA
- Random

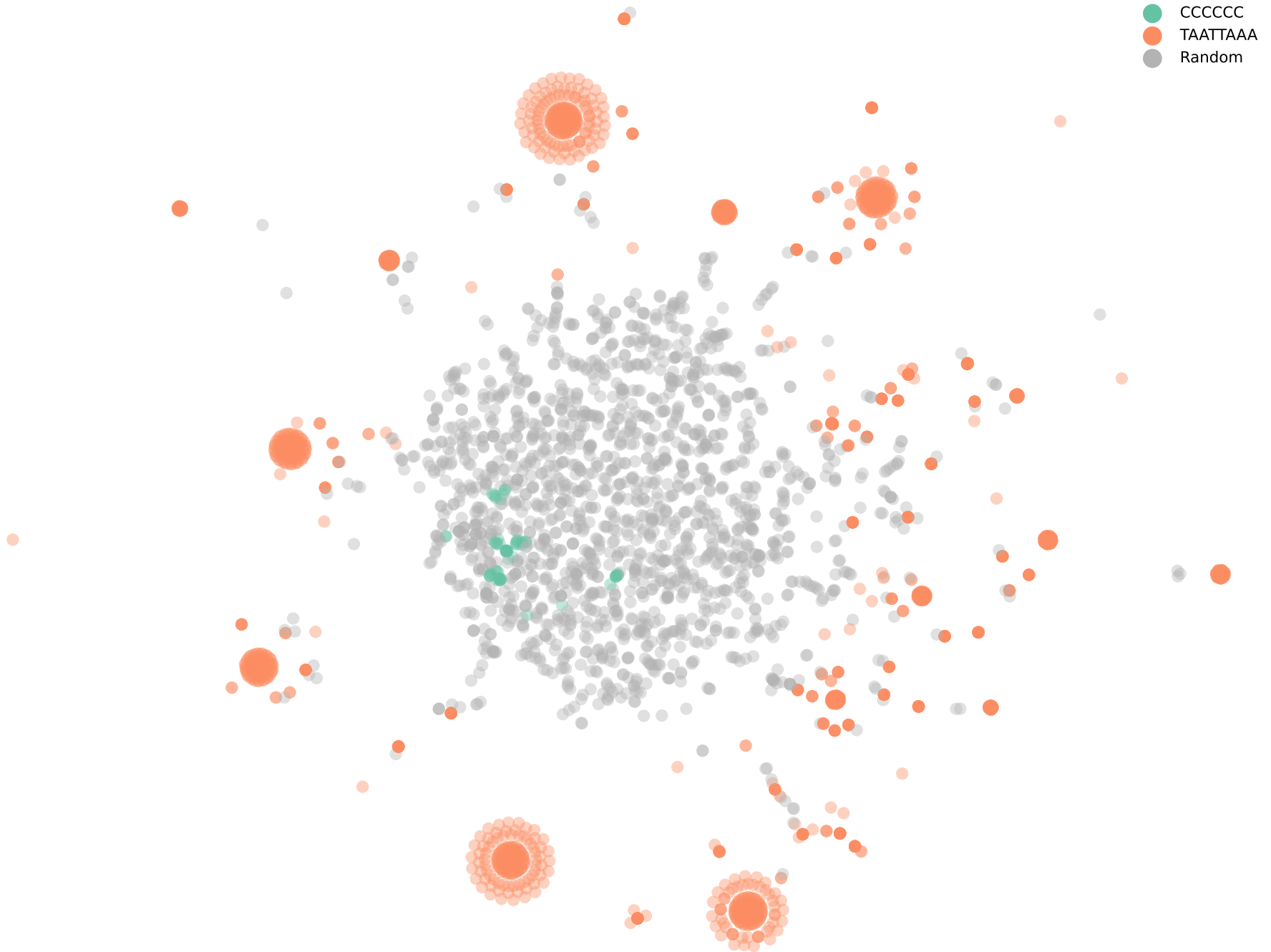

Supplement: Supplement 8 [file Supplemental_Data_1.zip › Supplemental_Data_1/ALX3_TGCAAG20NGA_AE_4/ALX3_TGCAAG20NGA_AE_4_tSNE.pdf]

UMAP Plot - ALX3\_TGCAAG20NGA\_AE\_4

Motif Sequence

- CCCCCC
- TAATTAAA
- Random

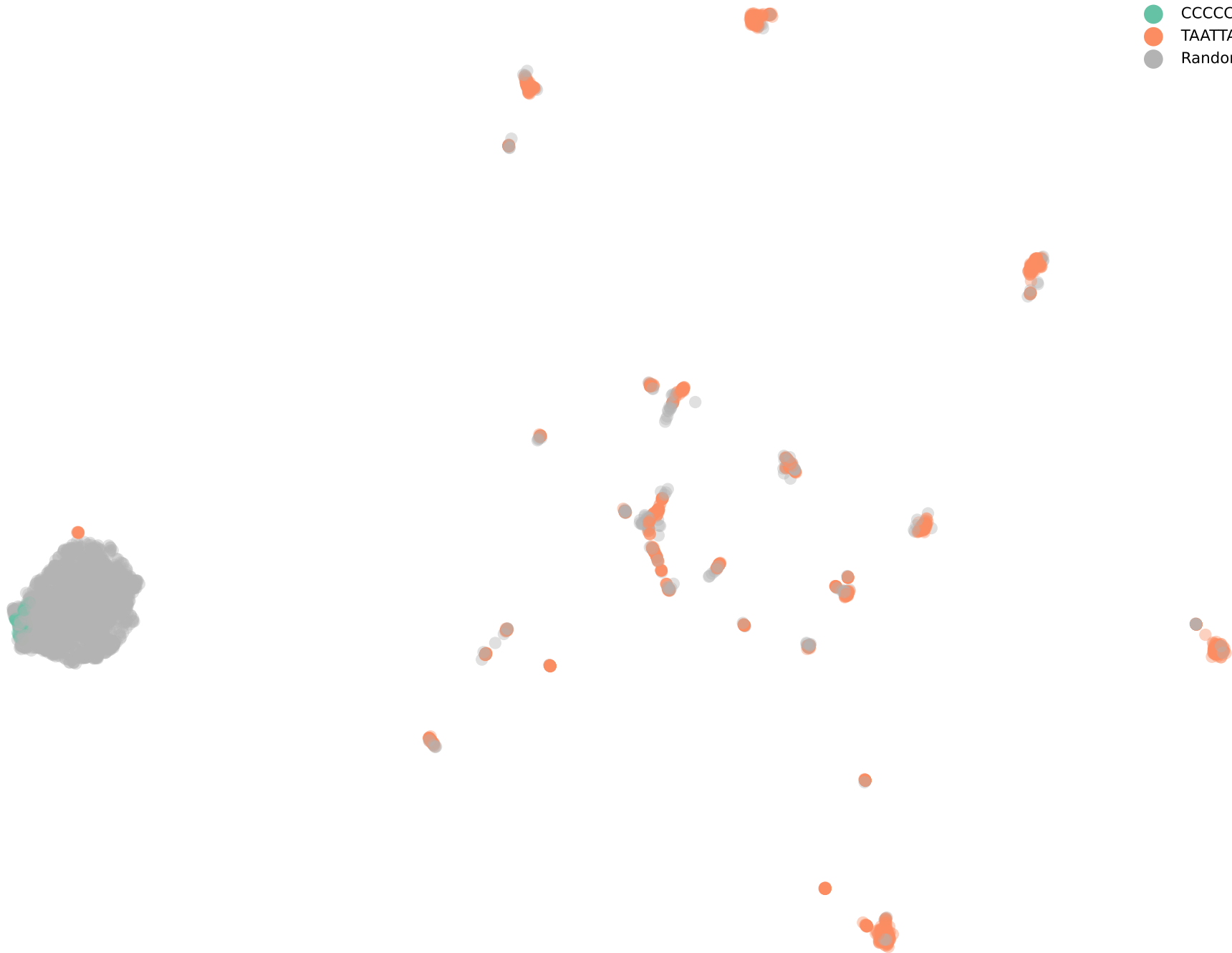

Supplement: Supplement 8 [file Supplemental_Data_1.zip › Supplemental_Data_1/ALX3_TGCAAG20NGA_AE_4/ALX3_TGCAAG20NGA_AE_4_UMAP.pdf]

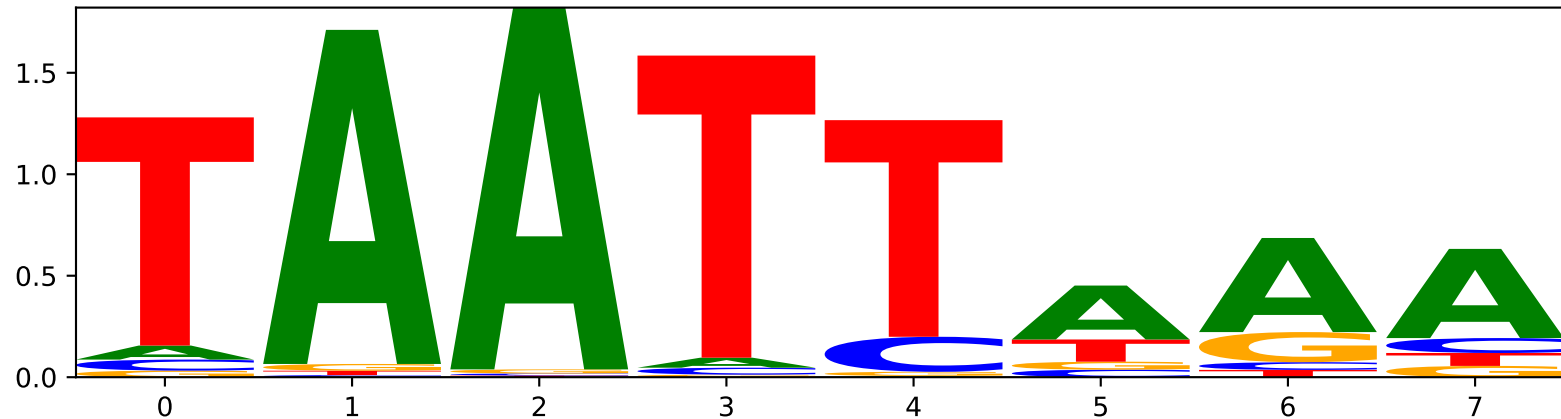

Supplement: Supplement 8 [file Supplemental_Data_1.zip › Supplemental_Data_1/ALX3_TGCAAG20NGA_AE_4/kmap_logo.pdf]

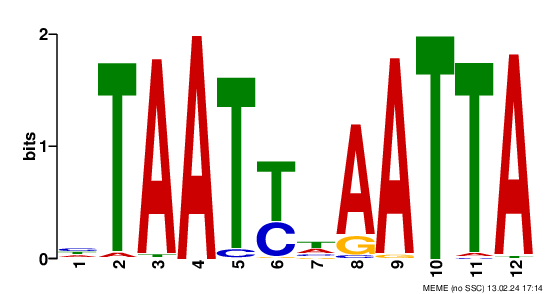

Supplement: Supplement 8 [file Supplemental_Data_1.zip › Supplemental_Data_1/ALX3_TGCAAG20NGA_AE_4/meme_logo.png]

KMAP LD Plot - ALX3\_TGTAAA20NAAG\_Z\_3

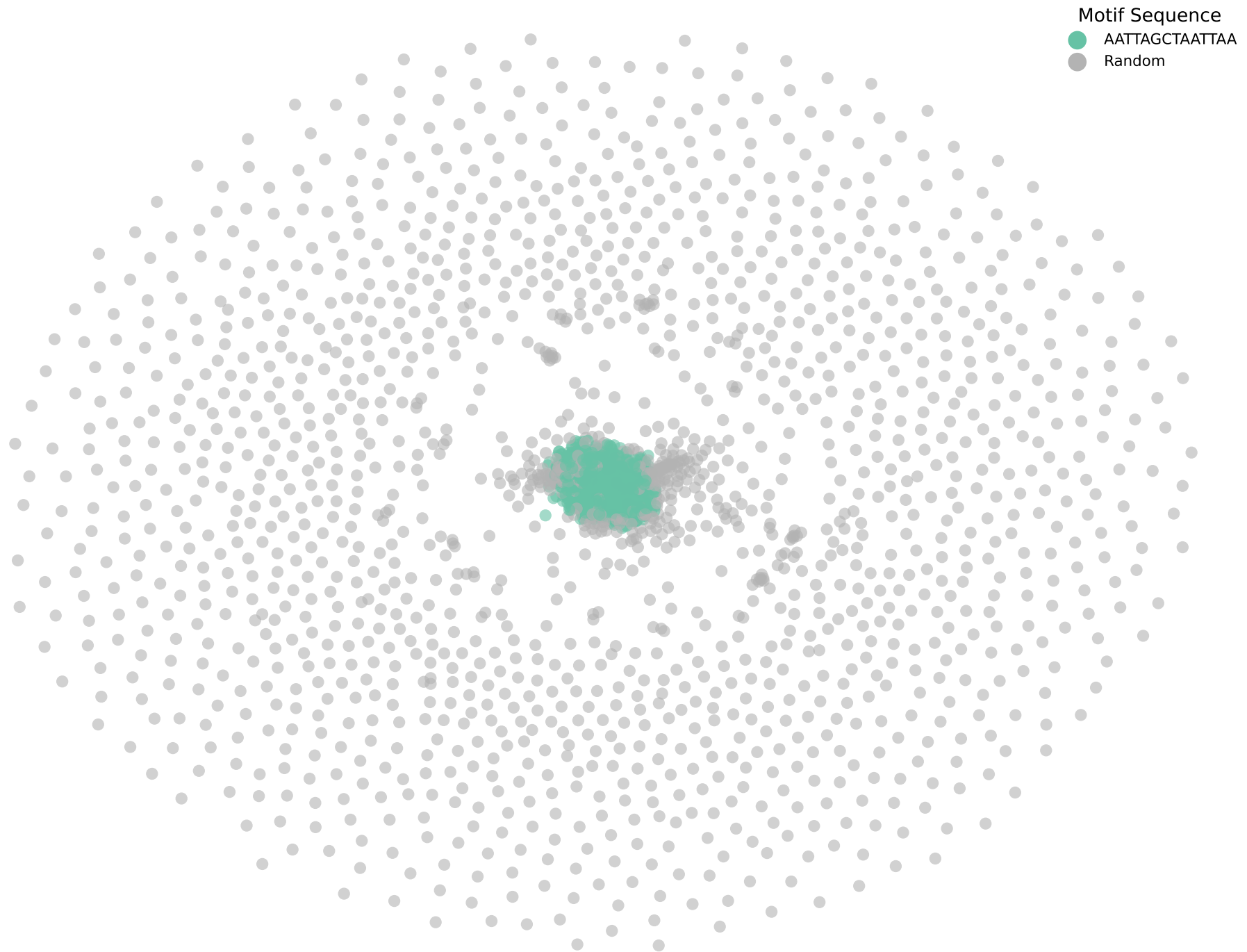

Supplement: Supplement 8 [file Supplemental_Data_1.zip › Supplemental_Data_1/ALX3_TGTAAA20NAAG_Z_3/ALX3_TGTAAA20NAAG_Z_3_KMAP.pdf]

MDS Plot - ALX3\_TGTAAA20NAAG\_Z\_3

Motif Sequence

- AATTAGCTAATTAA
- Random

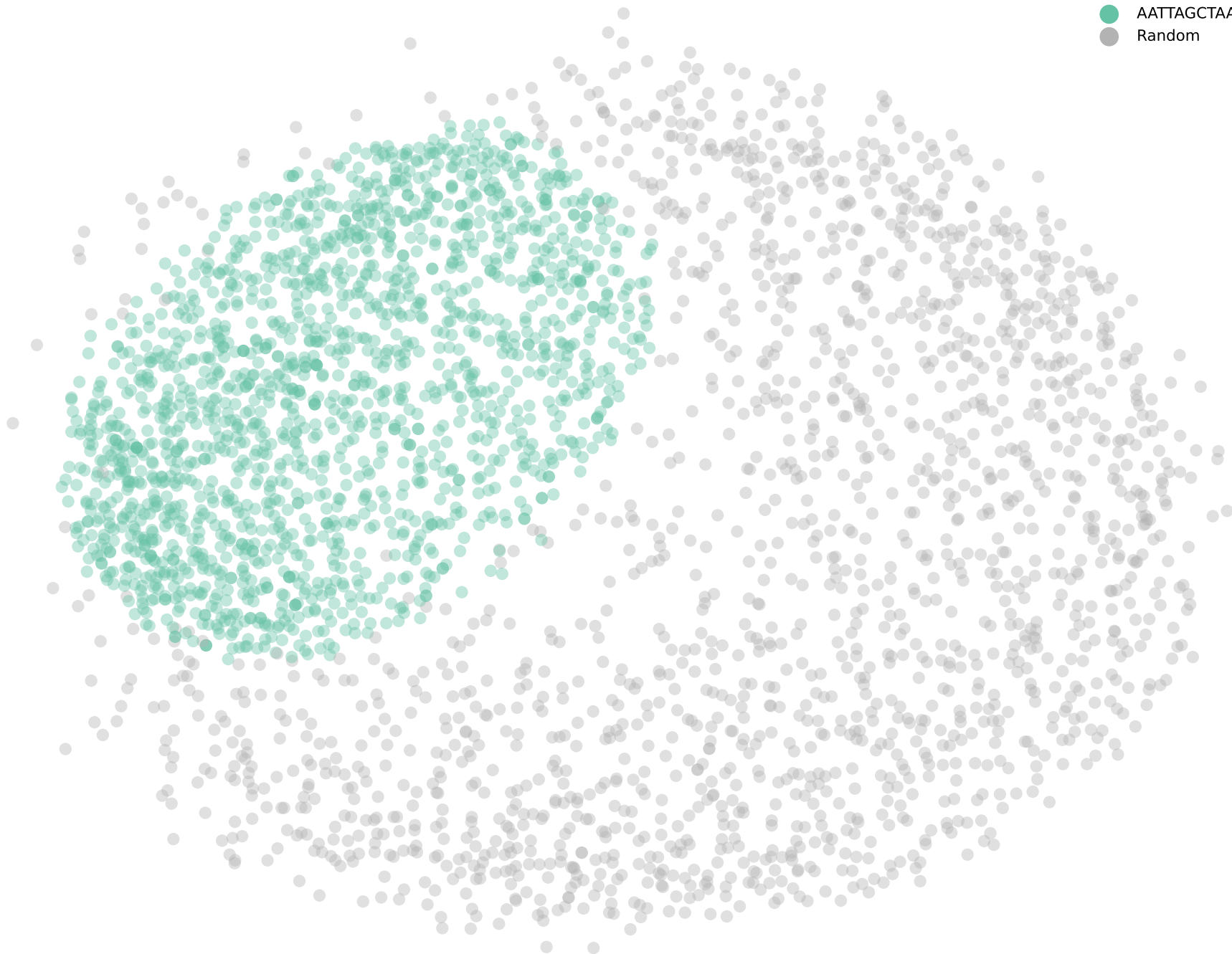

Supplement: Supplement 8 [file Supplemental_Data_1.zip › Supplemental_Data_1/ALX3_TGTAAA20NAAG_Z_3/ALX3_TGTAAA20NAAG_Z_3_MDS.pdf]

PCA Plot - ALX3\_TGTAAA20NAAG\_Z\_3

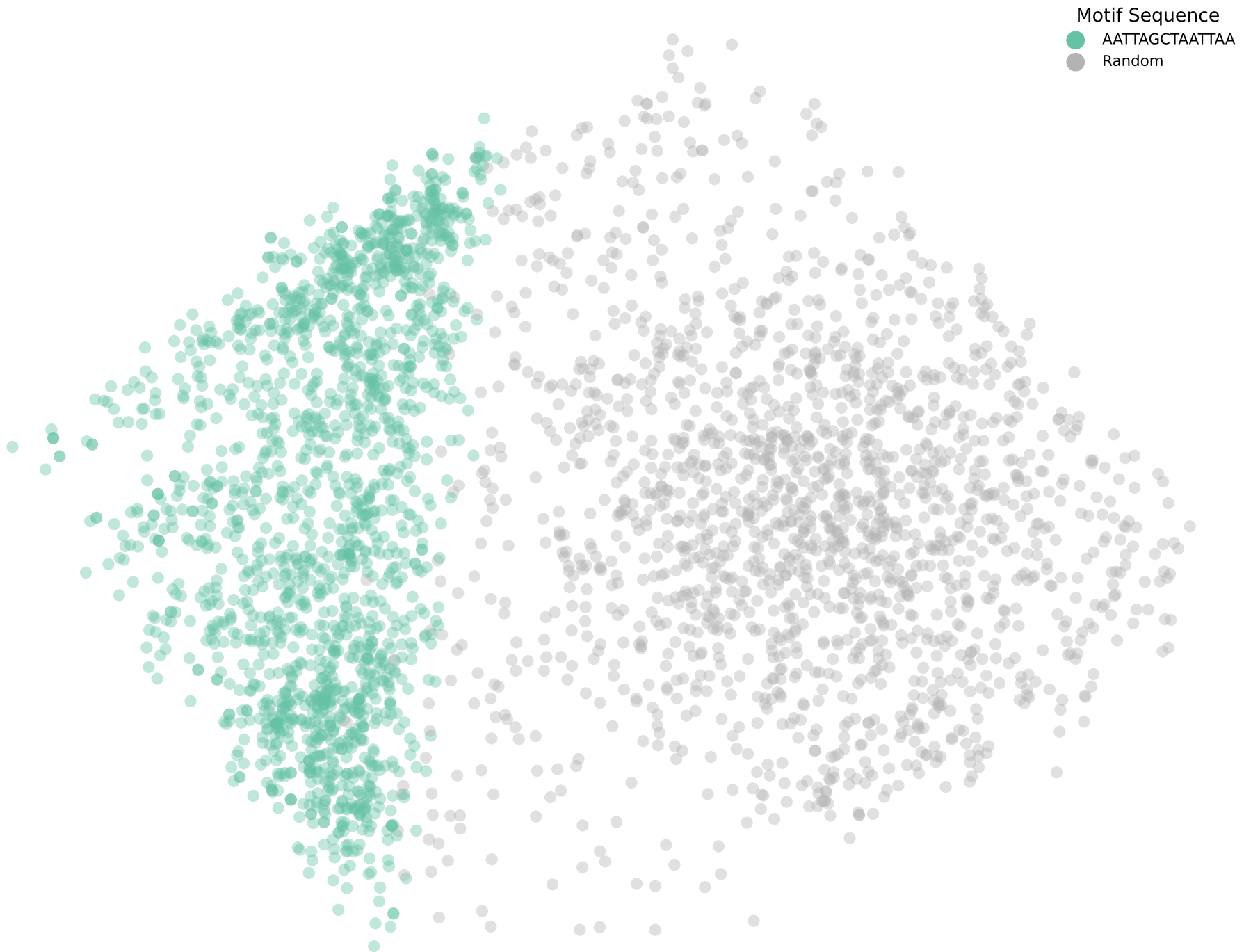

Supplement: Supplement 8 [file Supplemental_Data_1.zip › Supplemental_Data_1/ALX3_TGTAAA20NAAG_Z_3/ALX3_TGTAAA20NAAG_Z_3_PCA.pdf]

tSNE Plot - ALX3\_TGTAAA20NAAG\_Z\_3

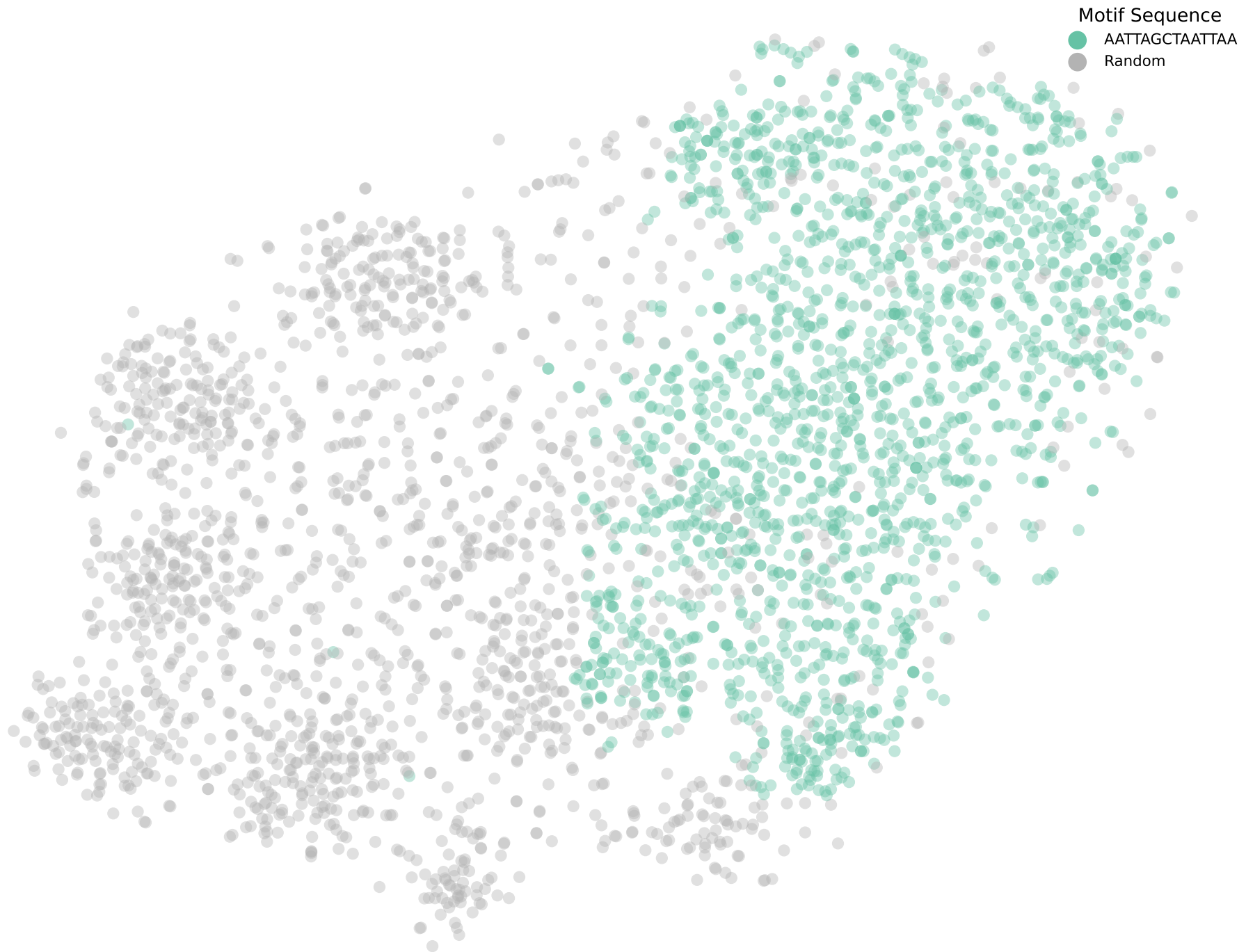

Supplement: Supplement 8 [file Supplemental_Data_1.zip › Supplemental_Data_1/ALX3_TGTAAA20NAAG_Z_3/ALX3_TGTAAA20NAAG_Z_3_tSNE.pdf]

UMAP Plot - ALX3\_TGTAAA20NAAG\_Z\_3

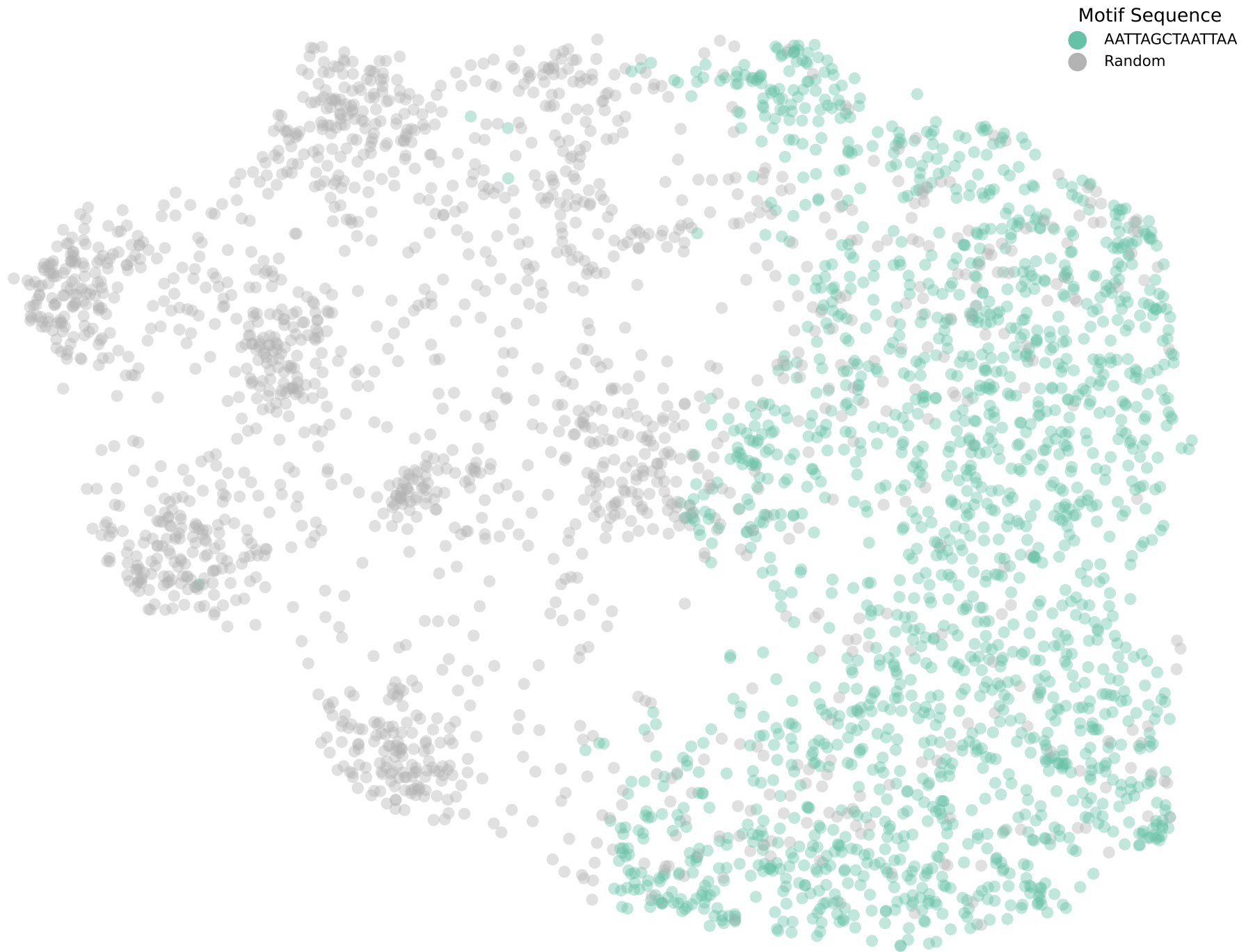

Supplement: Supplement 8 [file Supplemental_Data_1.zip › Supplemental_Data_1/ALX3_TGTAAA20NAAG_Z_3/ALX3_TGTAAA20NAAG_Z_3_UMAP.pdf]

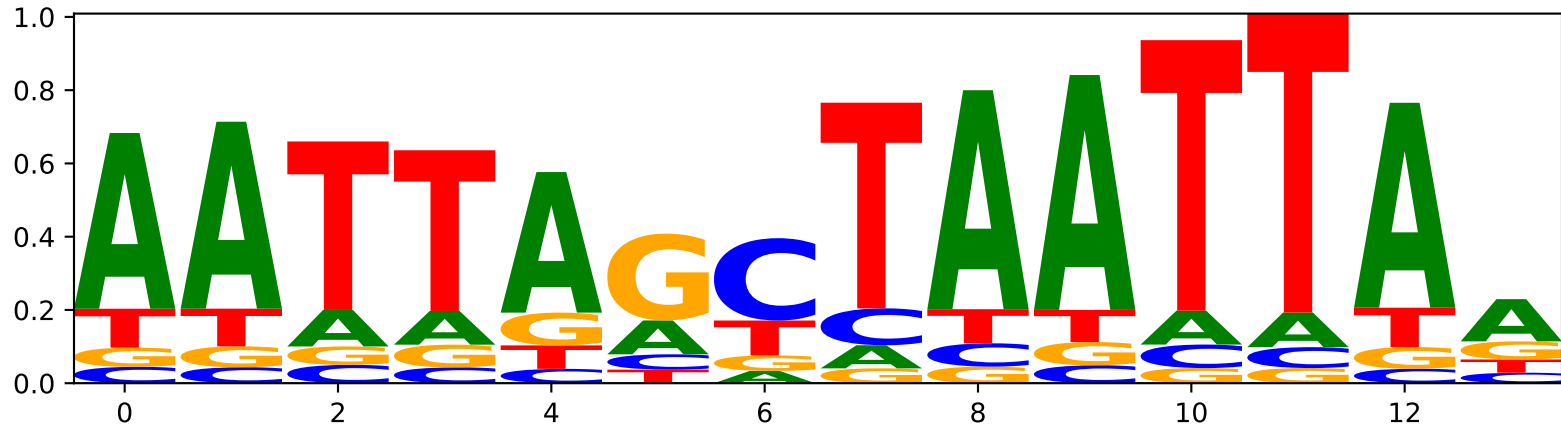

Supplement: Supplement 8 [file Supplemental_Data_1.zip › Supplemental_Data_1/ALX3_TGTAAA20NAAG_Z_3/kmap_logo.pdf]

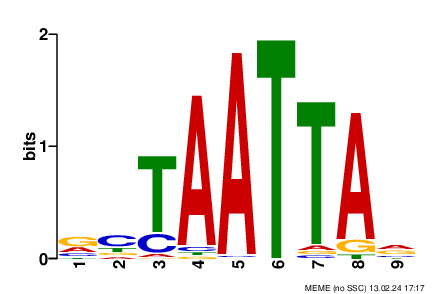

Supplement: Supplement 8 [file Supplemental_Data_1.zip › Supplemental_Data_1/ALX3_TGTAAA20NAAG_Z_3/meme_logo.png]

KMAP LD Plot - ALX3\_TGTAAA20NAAG\_Z\_4

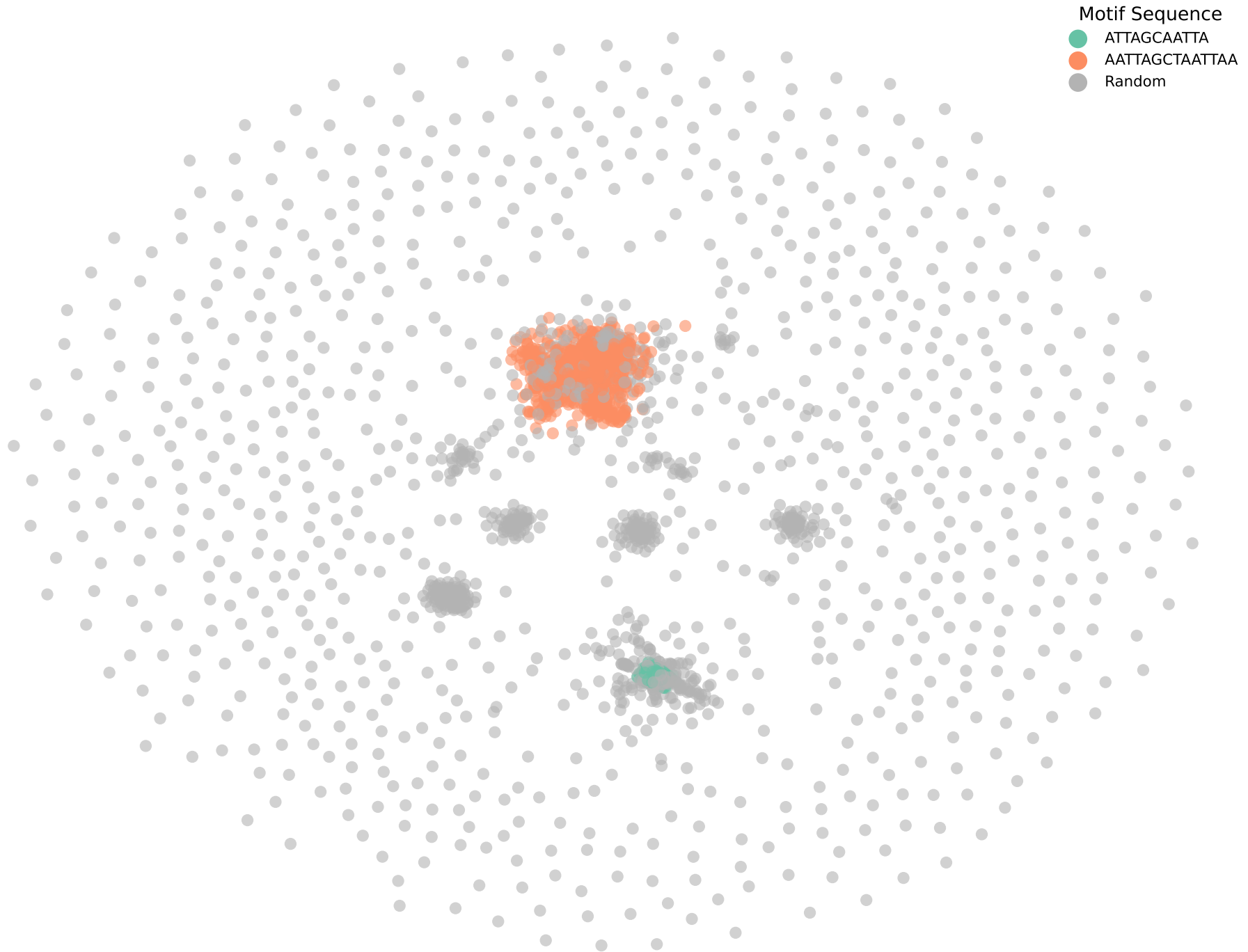

Supplement: Supplement 8 [file Supplemental_Data_1.zip › Supplemental_Data_1/ALX3_TGTAAA20NAAG_Z_4/ALX3_TGTAAA20NAAG_Z_4_KMAP.pdf]

MDS Plot - ALX3\_TGTAAA20NAAG\_Z\_4

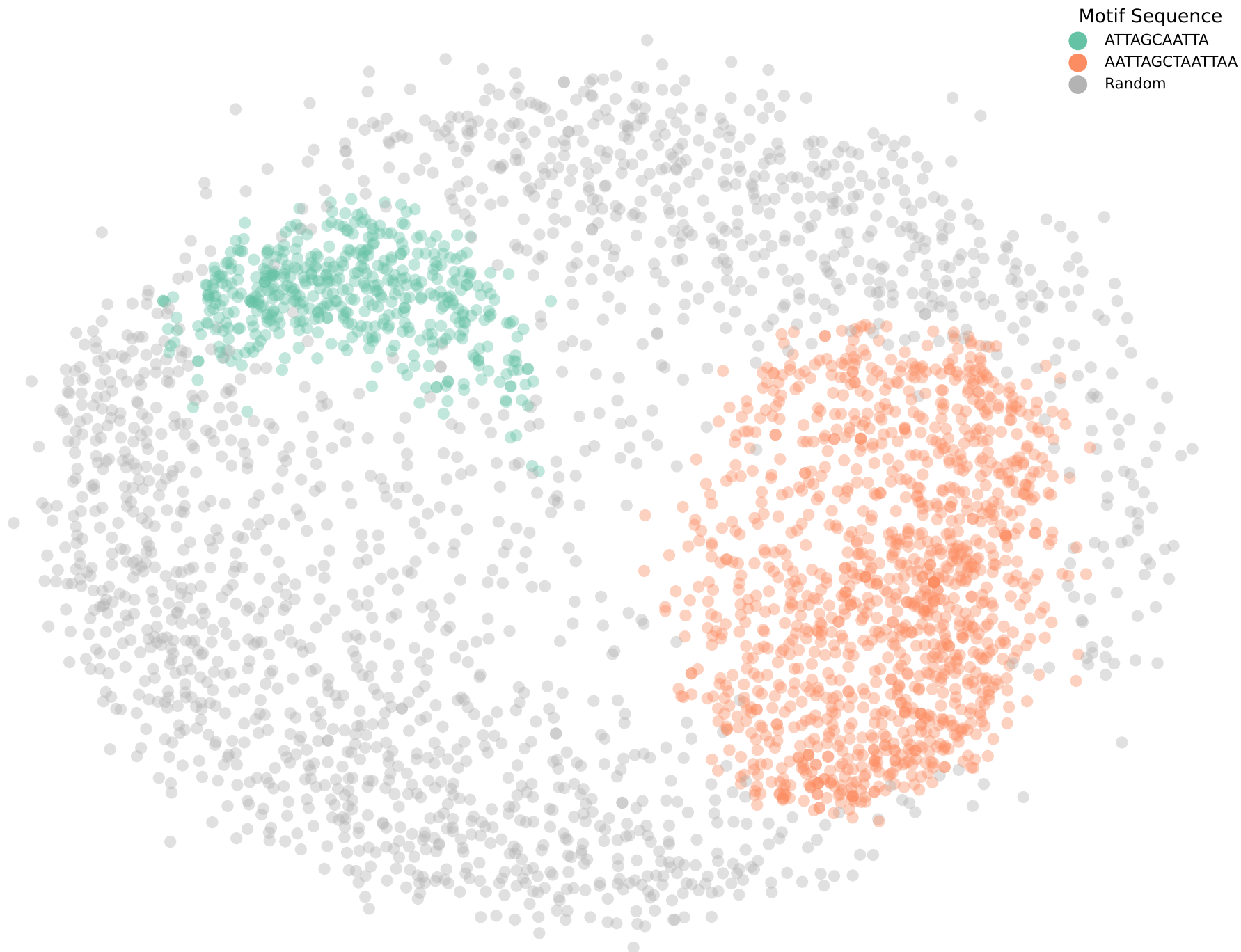

Supplement: Supplement 8 [file Supplemental_Data_1.zip › Supplemental_Data_1/ALX3_TGTAAA20NAAG_Z_4/ALX3_TGTAAA20NAAG_Z_4_MDS.pdf]

PCA Plot - ALX3\_TGTAAA20NAAG\_Z\_4

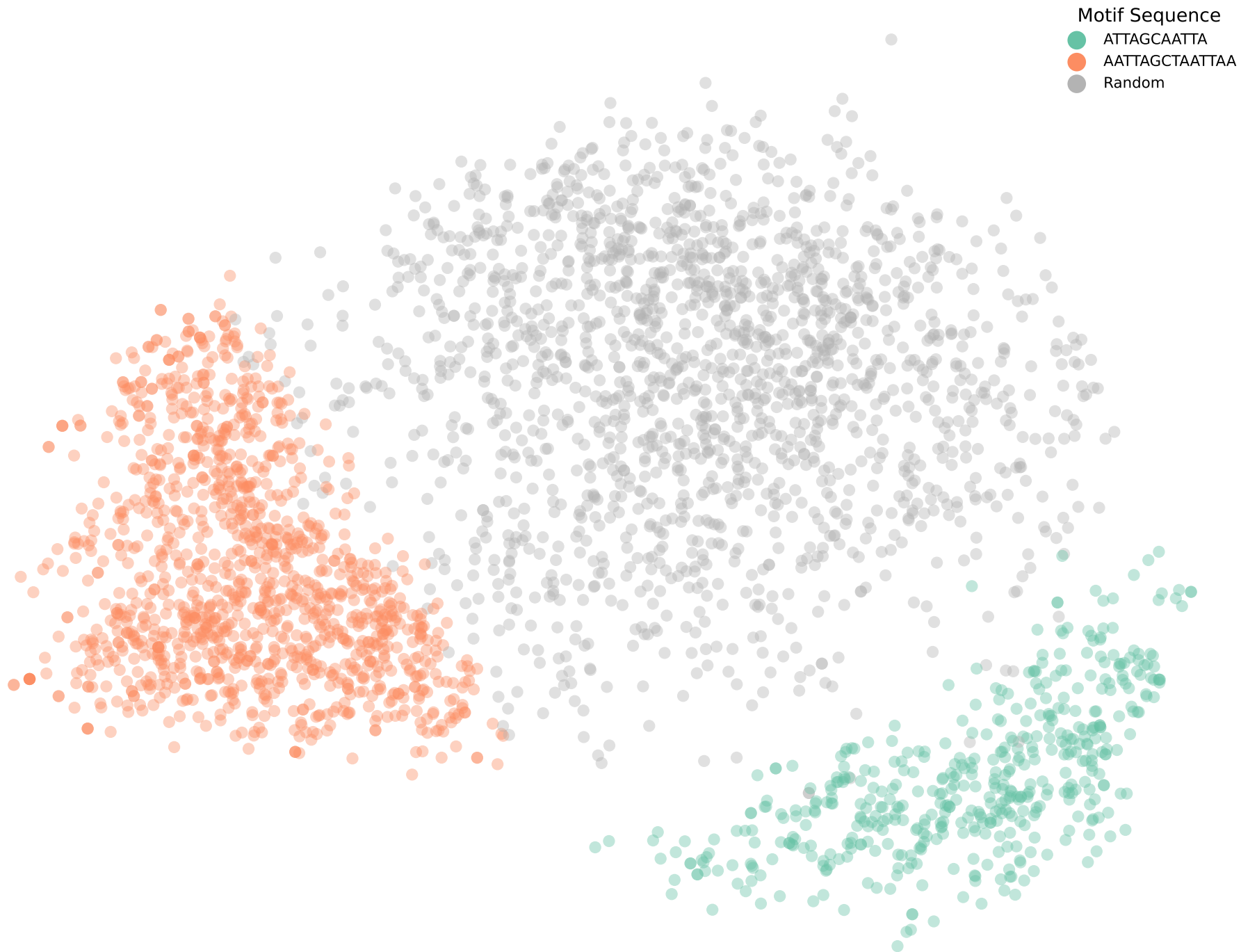

Supplement: Supplement 8 [file Supplemental_Data_1.zip › Supplemental_Data_1/ALX3_TGTAAA20NAAG_Z_4/ALX3_TGTAAA20NAAG_Z_4_PCA.pdf]

tSNE Plot - ALX3\_TGTAAA20NAAG\_Z\_4

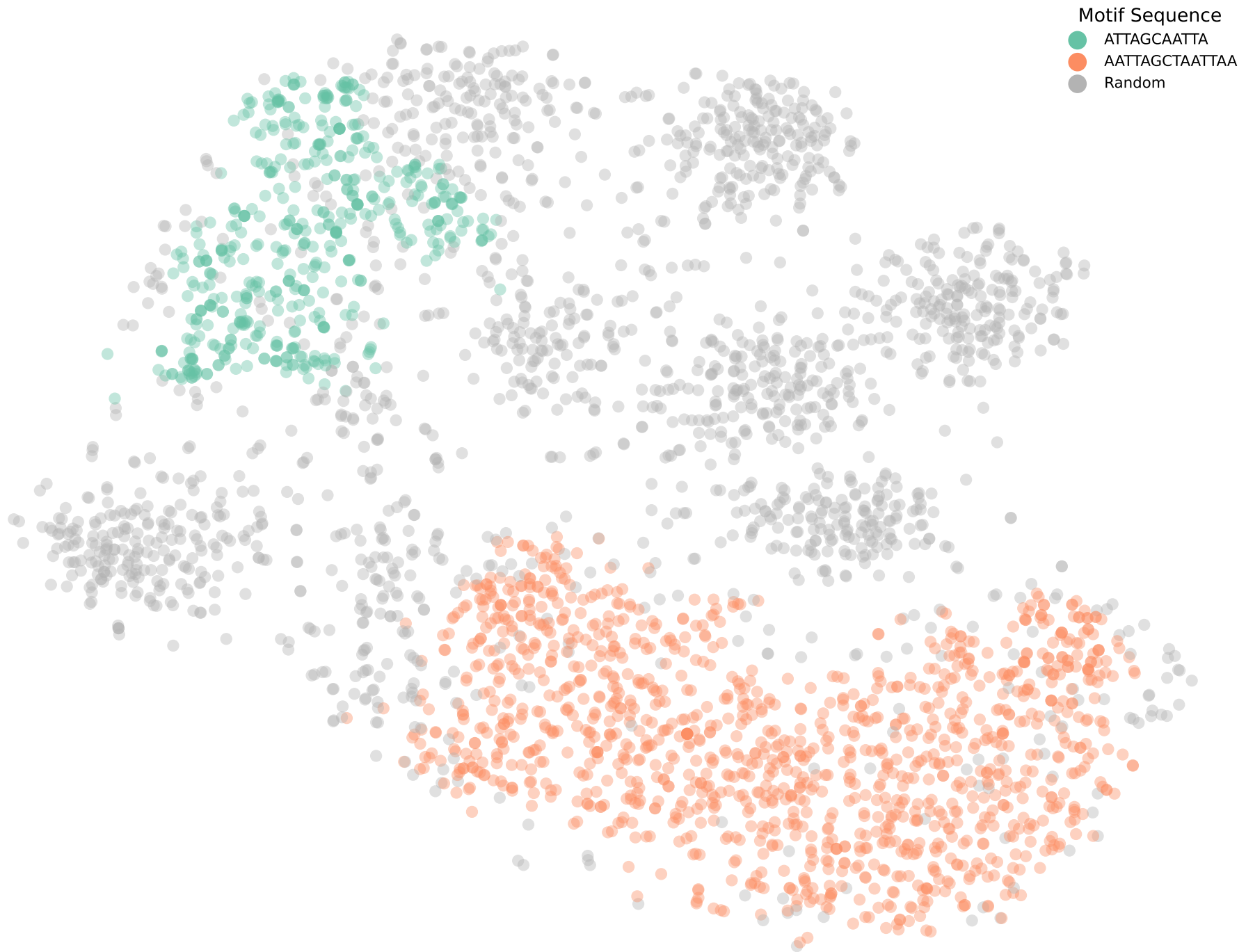

Supplement: Supplement 8 [file Supplemental_Data_1.zip › Supplemental_Data_1/ALX3_TGTAAA20NAAG_Z_4/ALX3_TGTAAA20NAAG_Z_4_tSNE.pdf]

UMAP Plot - ALX3\_TGTAAA20NAAG\_Z\_4

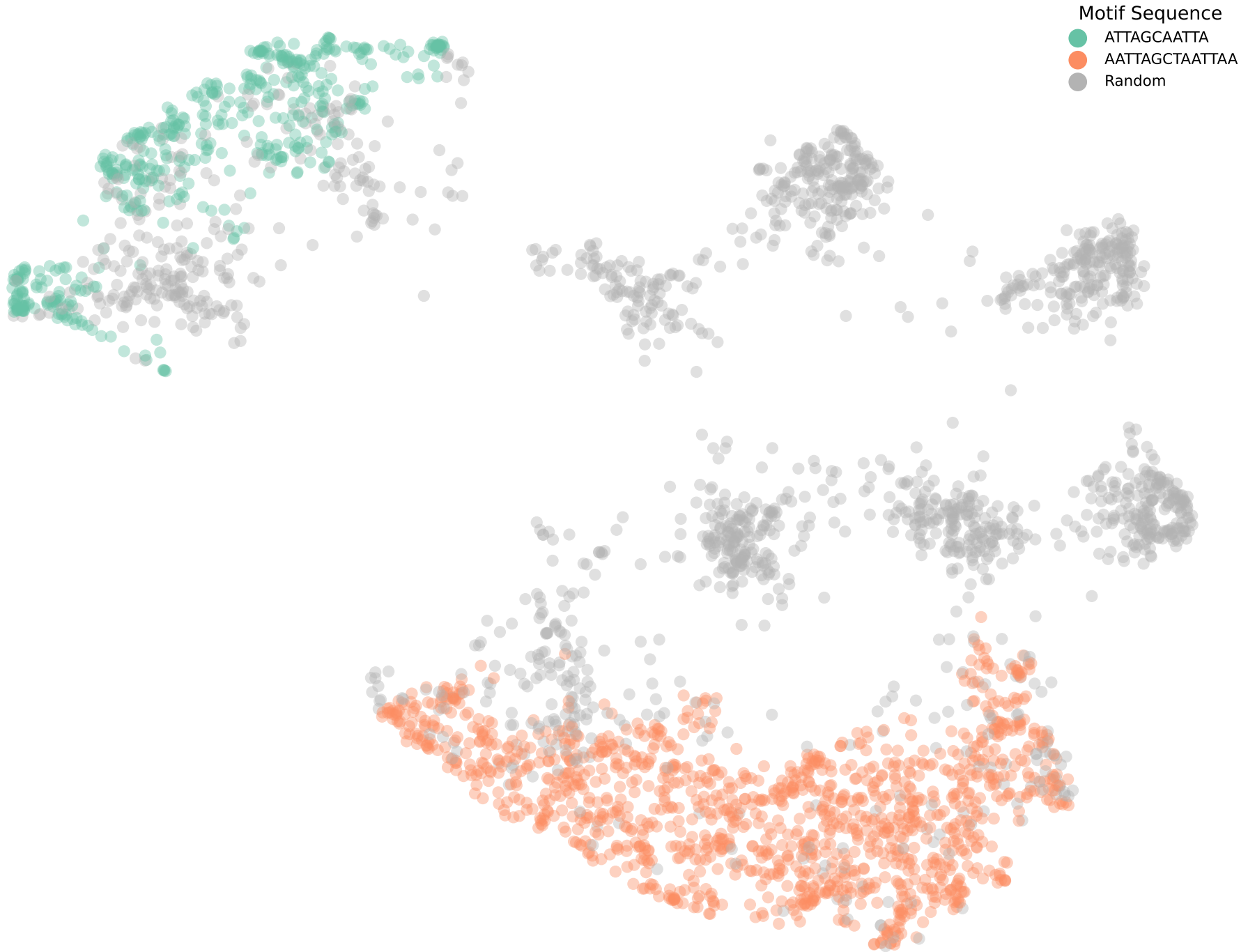

Supplement: Supplement 8 [file Supplemental_Data_1.zip › Supplemental_Data_1/ALX3_TGTAAA20NAAG_Z_4/ALX3_TGTAAA20NAAG_Z_4_UMAP.pdf]

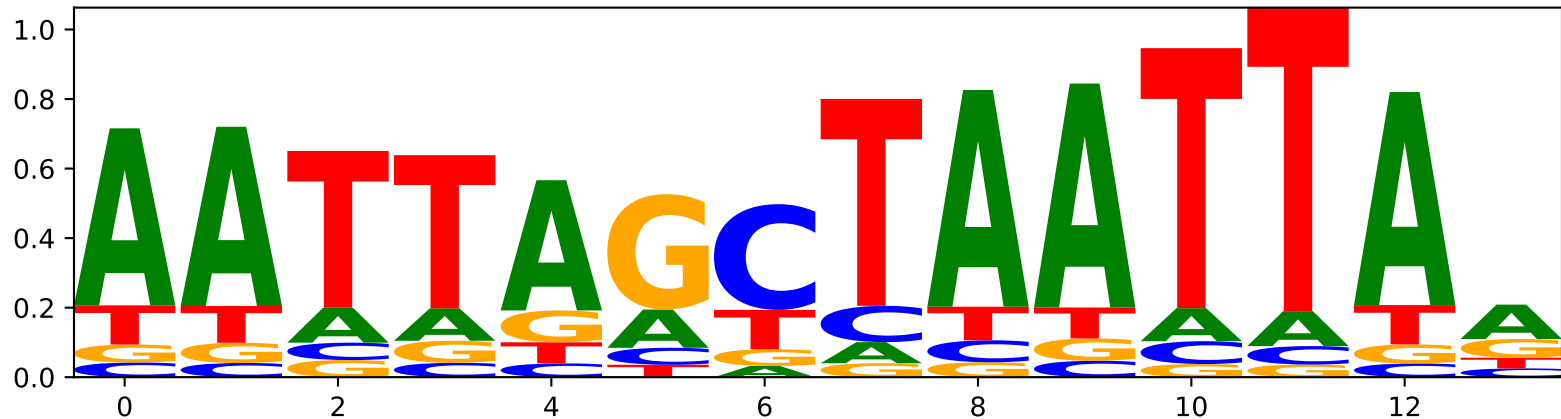

Supplement: Supplement 8 [file Supplemental_Data_1.zip › Supplemental_Data_1/ALX3_TGTAAA20NAAG_Z_4/kmap_logo.pdf]

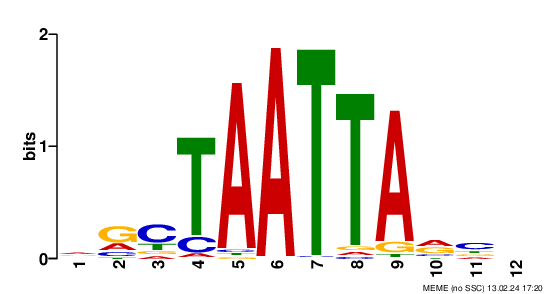

Supplement: Supplement 8 [file Supplemental_Data_1.zip › Supplemental_Data_1/ALX3_TGTAAA20NAAG_Z_4/meme_logo.png]

KMAP LD Plot - Alx4\_TGGTAG20NCG\_P\_3

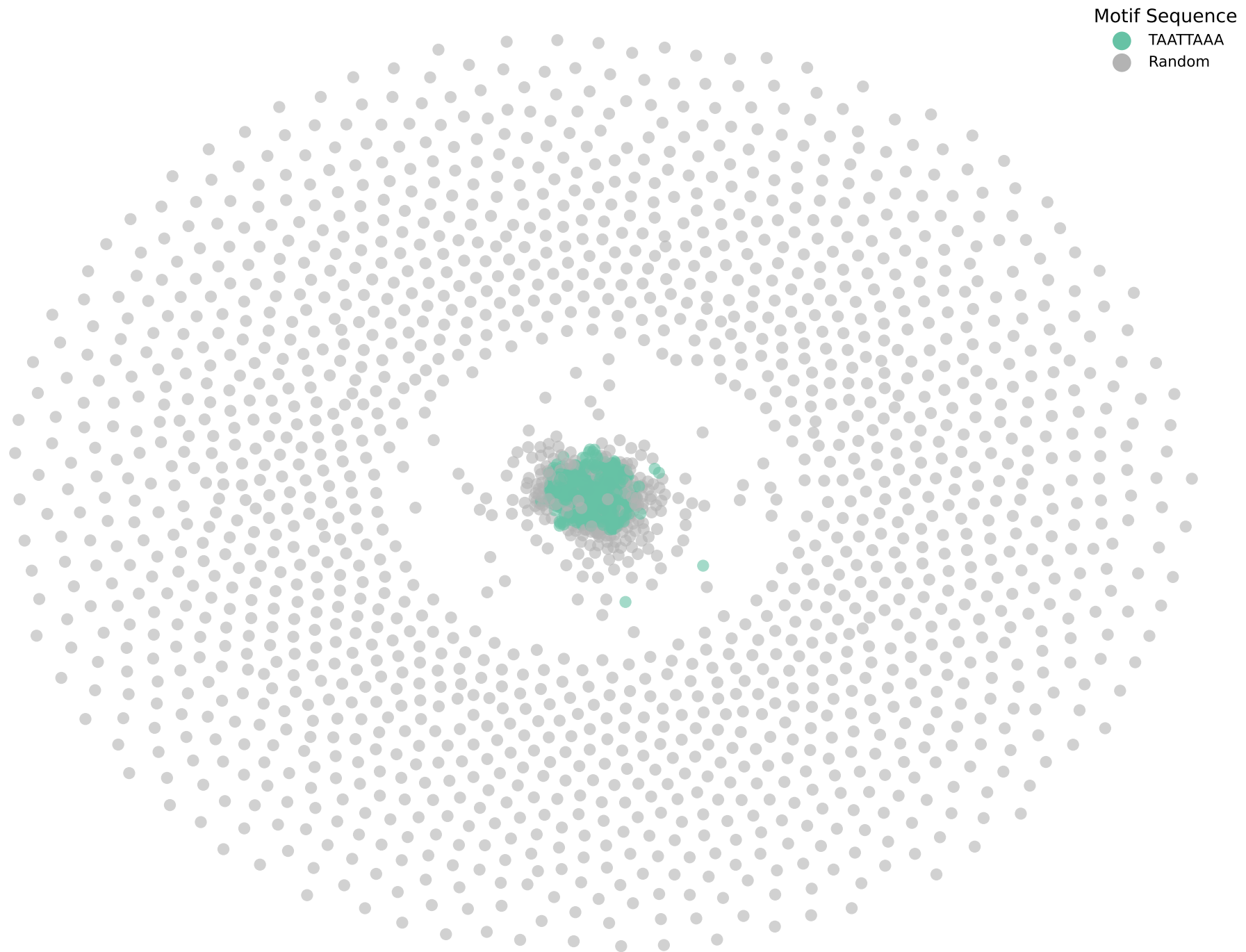

Supplement: Supplement 8 [file Supplemental_Data_1.zip › Supplemental_Data_1/Alx4_TGGTAG20NCG_P_3/Alx4_TGGTAG20NCG_P_3_KMAP.pdf]

MDS Plot - Alx4\_TGGTAG20NCG\_P\_3

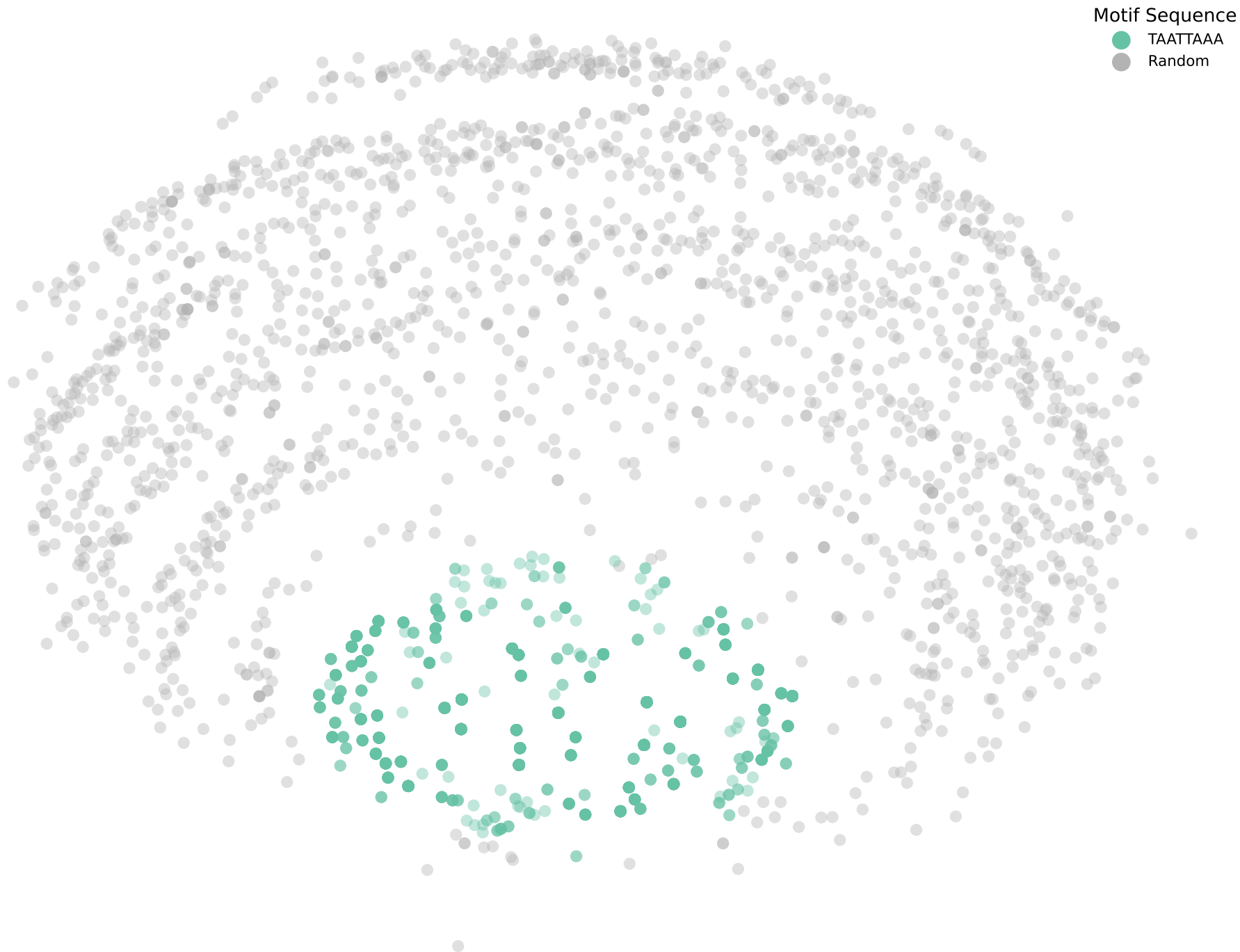

Supplement: Supplement 8 [file Supplemental_Data_1.zip › Supplemental_Data_1/Alx4_TGGTAG20NCG_P_3/Alx4_TGGTAG20NCG_P_3_MDS.pdf]

PCA Plot - Alx4\_TGGTAG20NCG\_P\_3

Motif Sequence

TAATTAAA

Random

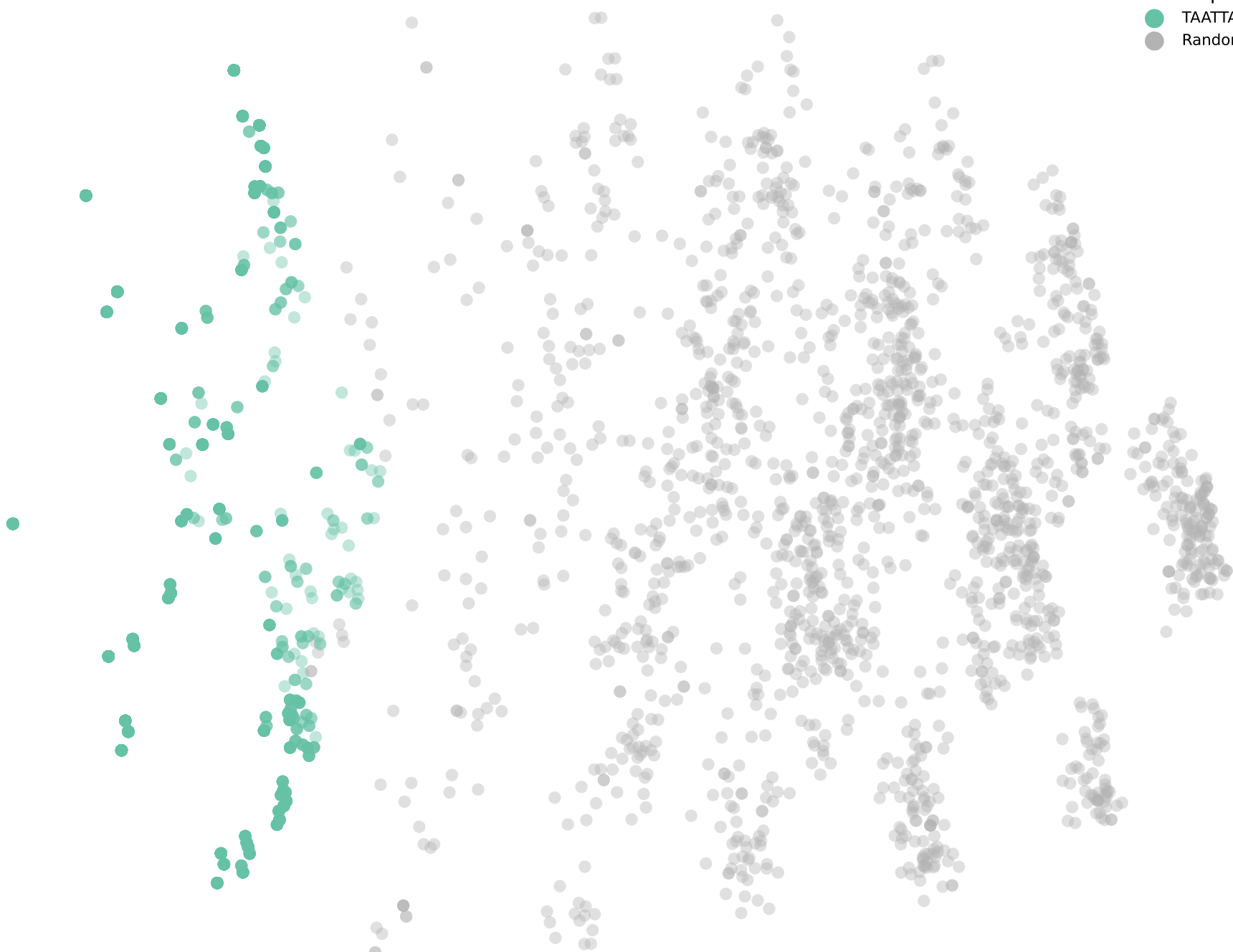

Supplement: Supplement 8 [file Supplemental_Data_1.zip › Supplemental_Data_1/Alx4_TGGTAG20NCG_P_3/Alx4_TGGTAG20NCG_P_3_PCA.pdf]

tSNE Plot - Alx4\_TGGTAG20NCG\_P\_3

Motif Sequence

TAATTAAA

Random

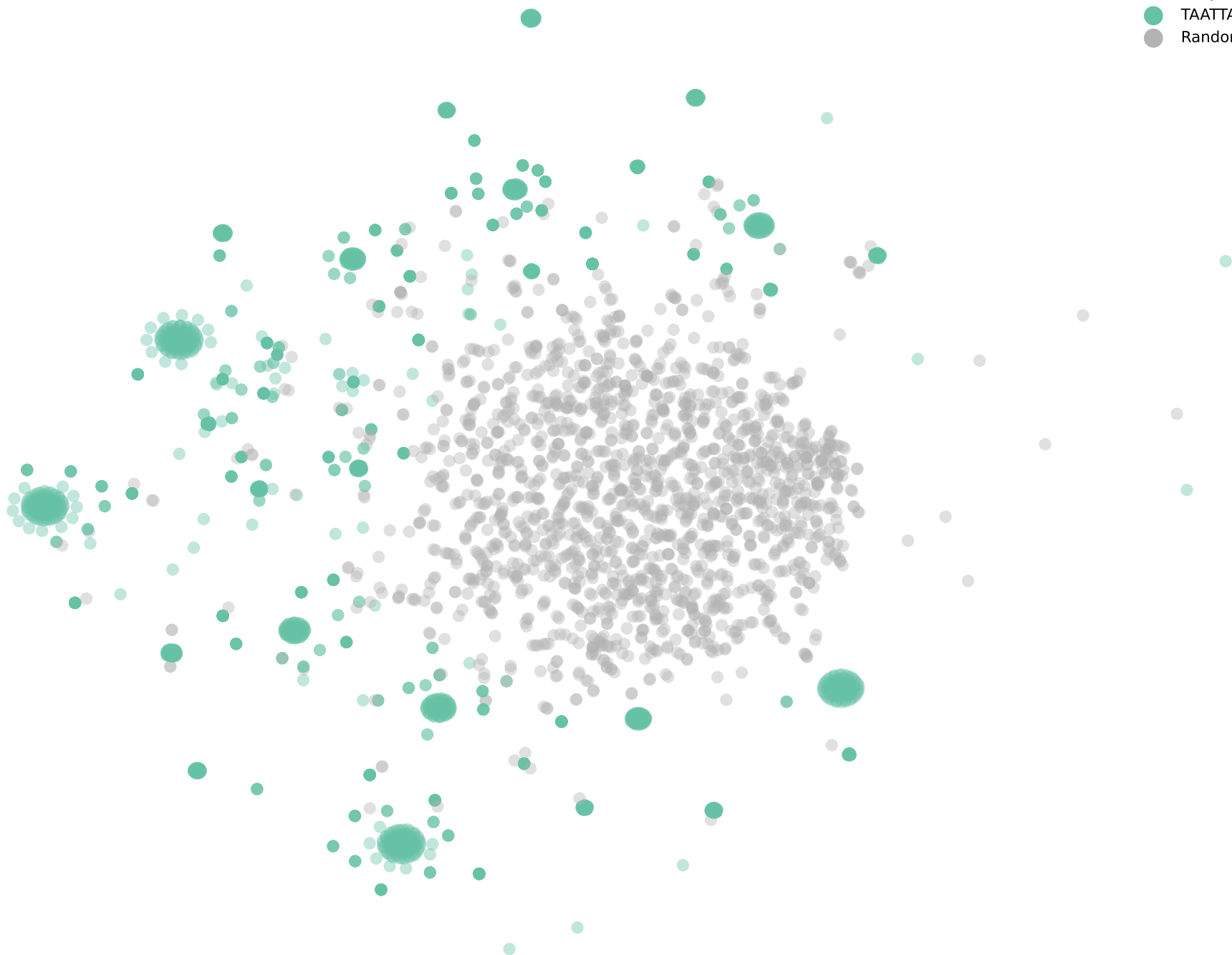

Supplement: Supplement 8 [file Supplemental_Data_1.zip › Supplemental_Data_1/Alx4_TGGTAG20NCG_P_3/Alx4_TGGTAG20NCG_P_3_tSNE.pdf]

UMAP Plot - Alx4\_TGGTAG20NCG\_P\_3

Motif Sequence

- TAATTAAA
- Random

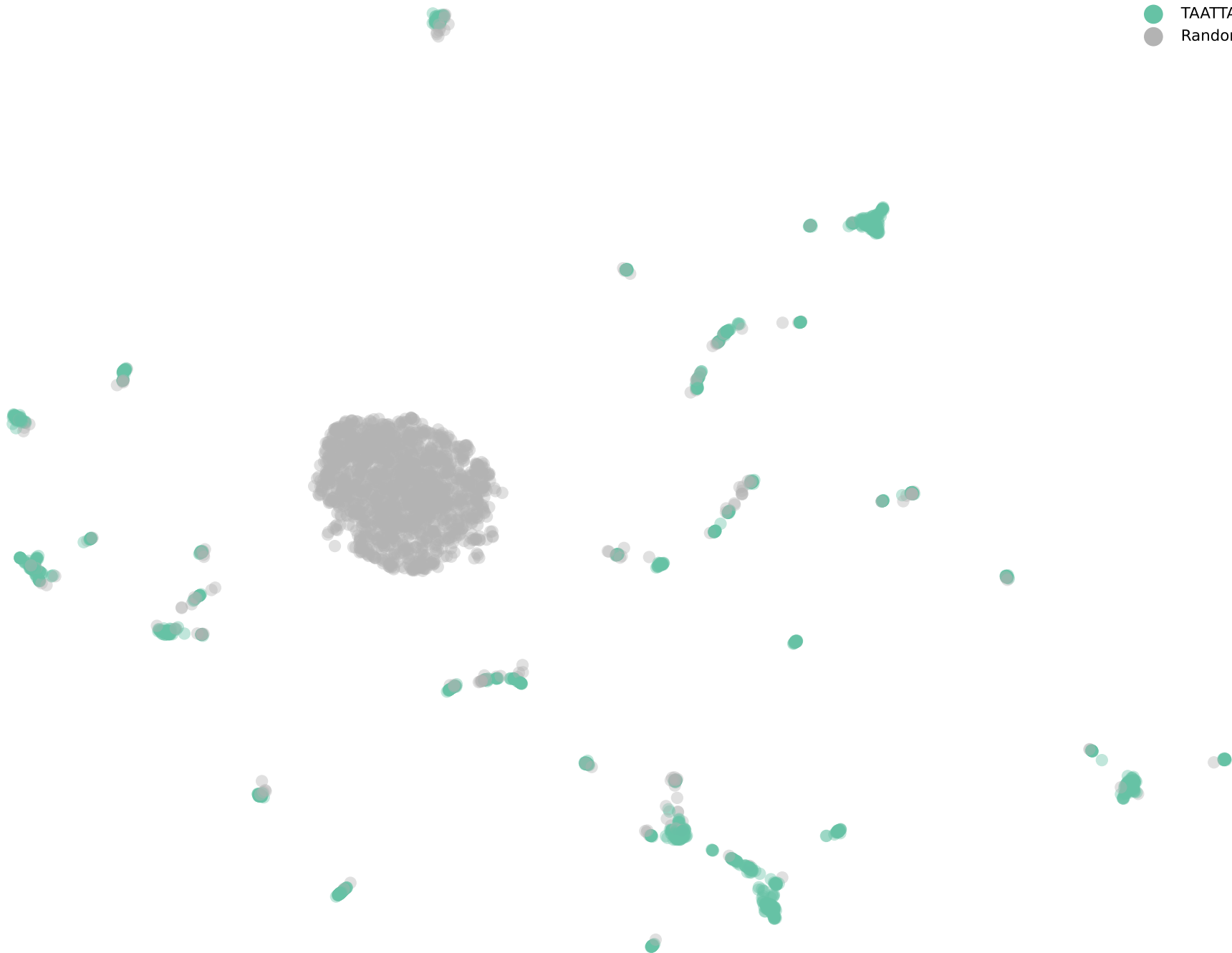

Supplement: Supplement 8 [file Supplemental_Data_1.zip › Supplemental_Data_1/Alx4_TGGTAG20NCG_P_3/Alx4_TGGTAG20NCG_P_3_UMAP.pdf]

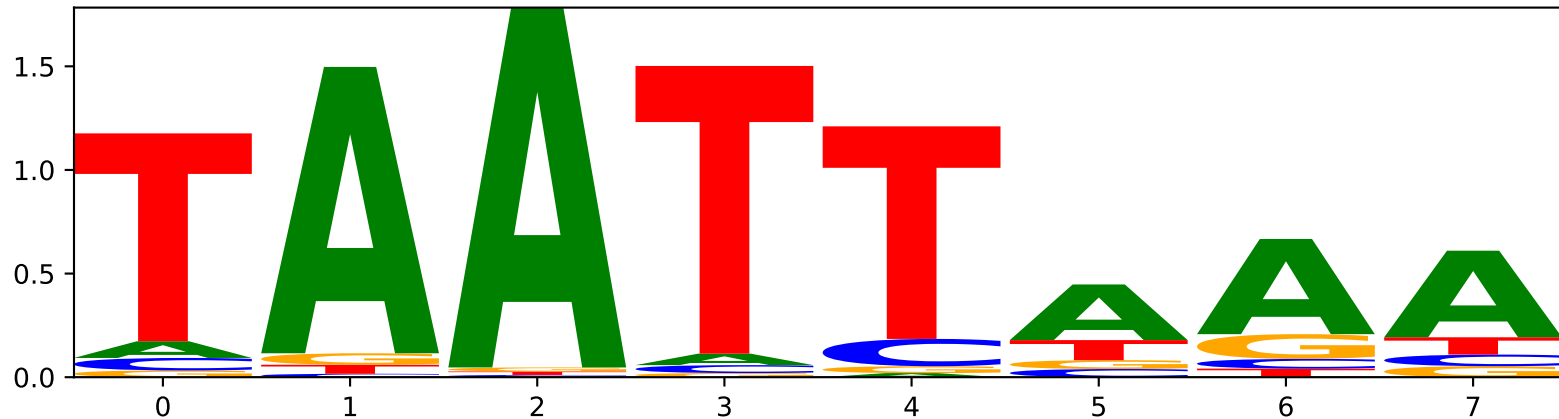

Supplement: Supplement 8 [file Supplemental_Data_1.zip › Supplemental_Data_1/Alx4_TGGTAG20NCG_P_3/kmap_logo.pdf]

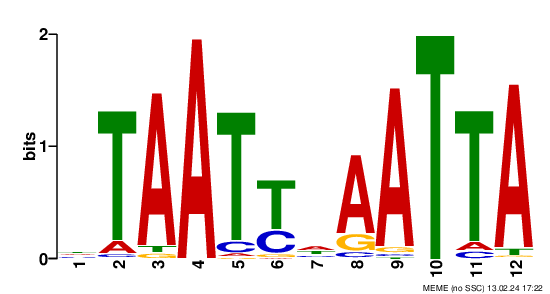

Supplement: Supplement 8 [file Supplemental_Data_1.zip › Supplemental_Data_1/Alx4_TGGTAG20NCG_P_3/meme_logo.png]

KMAP LD Plot - A1x4\_TGGTAG20NCG\_P\_4

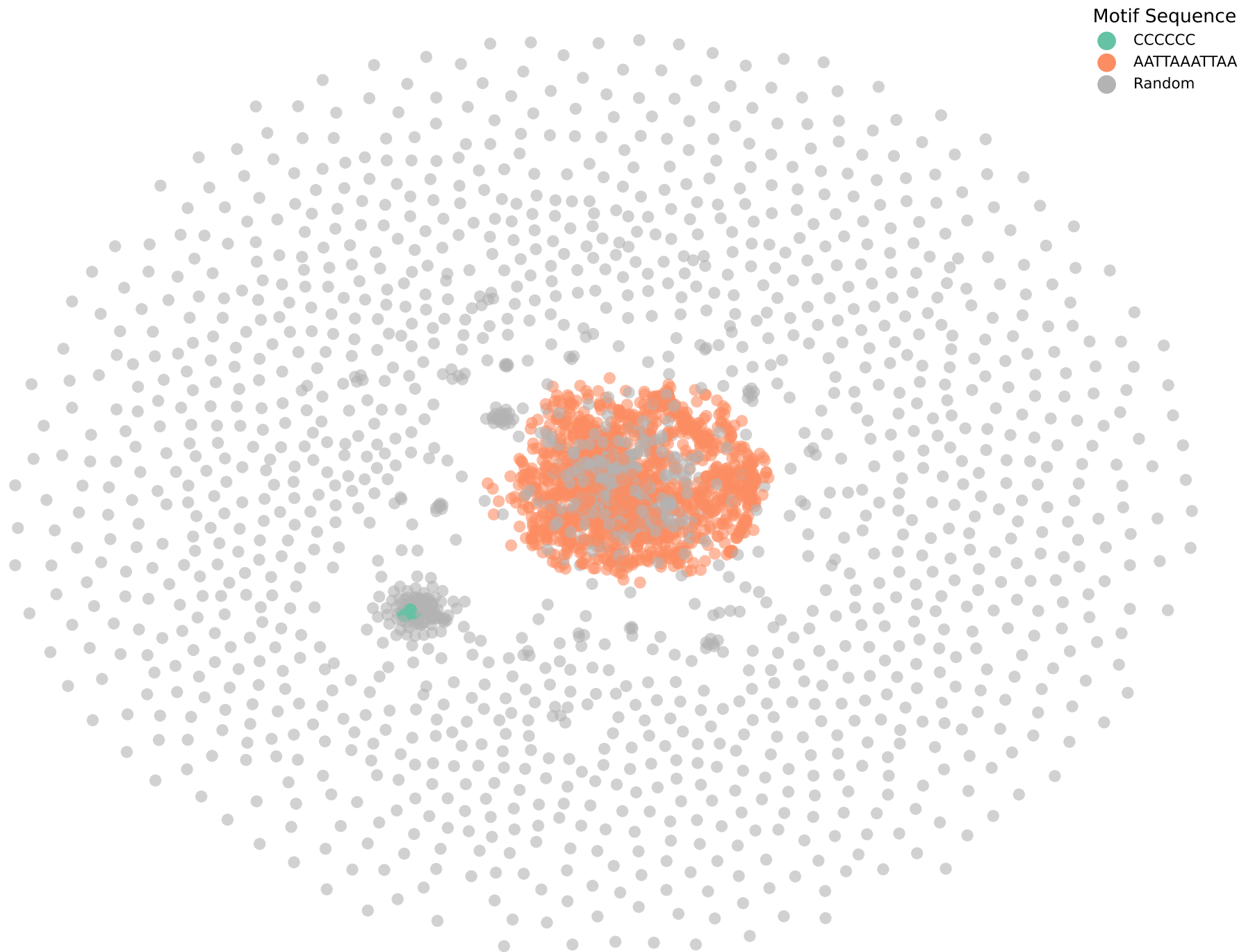

Supplement: Supplement 8 [file Supplemental_Data_1.zip › Supplemental_Data_1/Alx4_TGGTAG20NCG_P_4/Alx4_TGGTAG20NCG_P_4_KMAP.pdf]

MDS Plot - Alx4\_TGGTAG20NCG\_P\_4

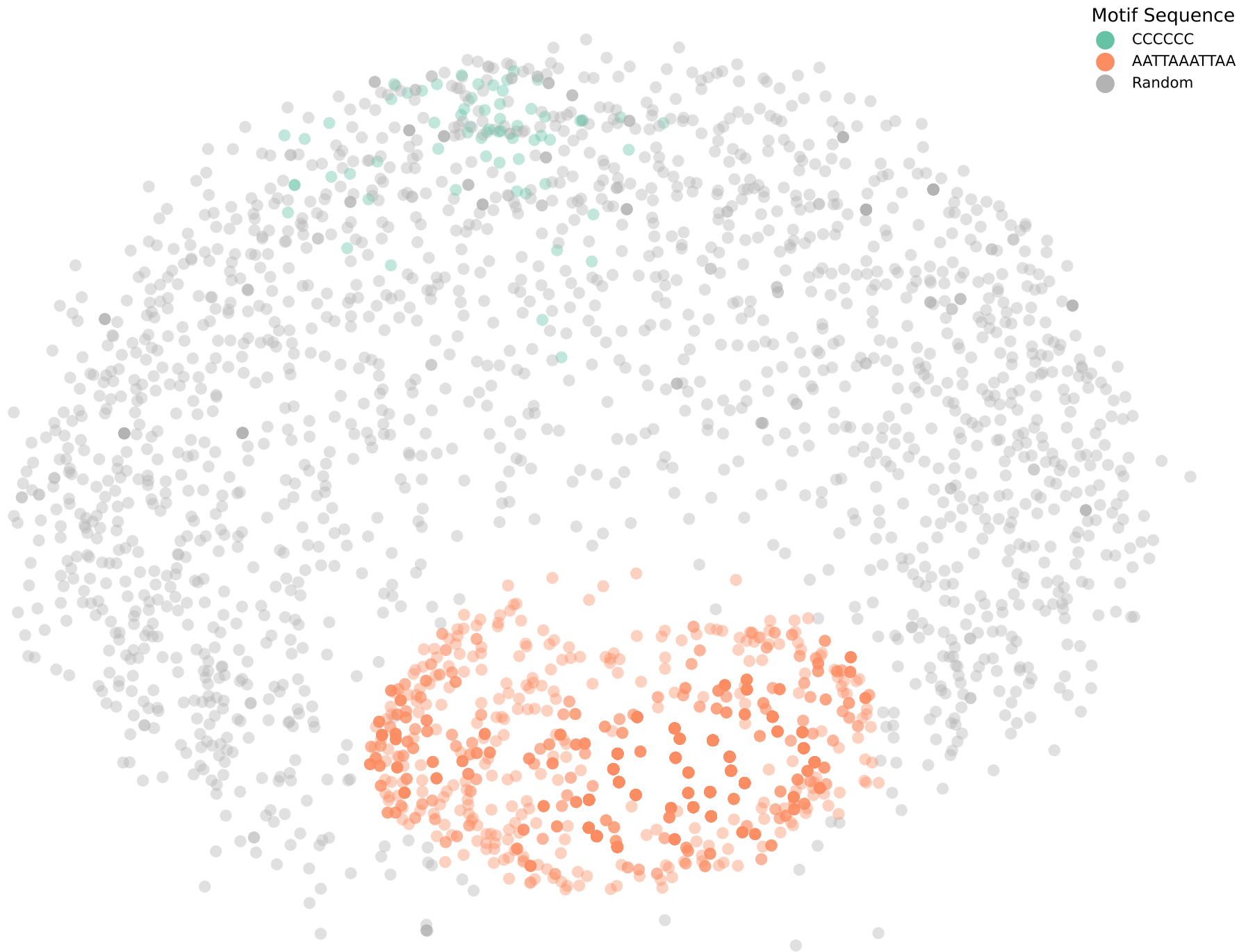

Supplement: Supplement 8 [file Supplemental_Data_1.zip › Supplemental_Data_1/Alx4_TGGTAG20NCG_P_4/Alx4_TGGTAG20NCG_P_4_MDS.pdf]

PCA Plot - Alx4\_TGGTAG20NCG\_P\_4

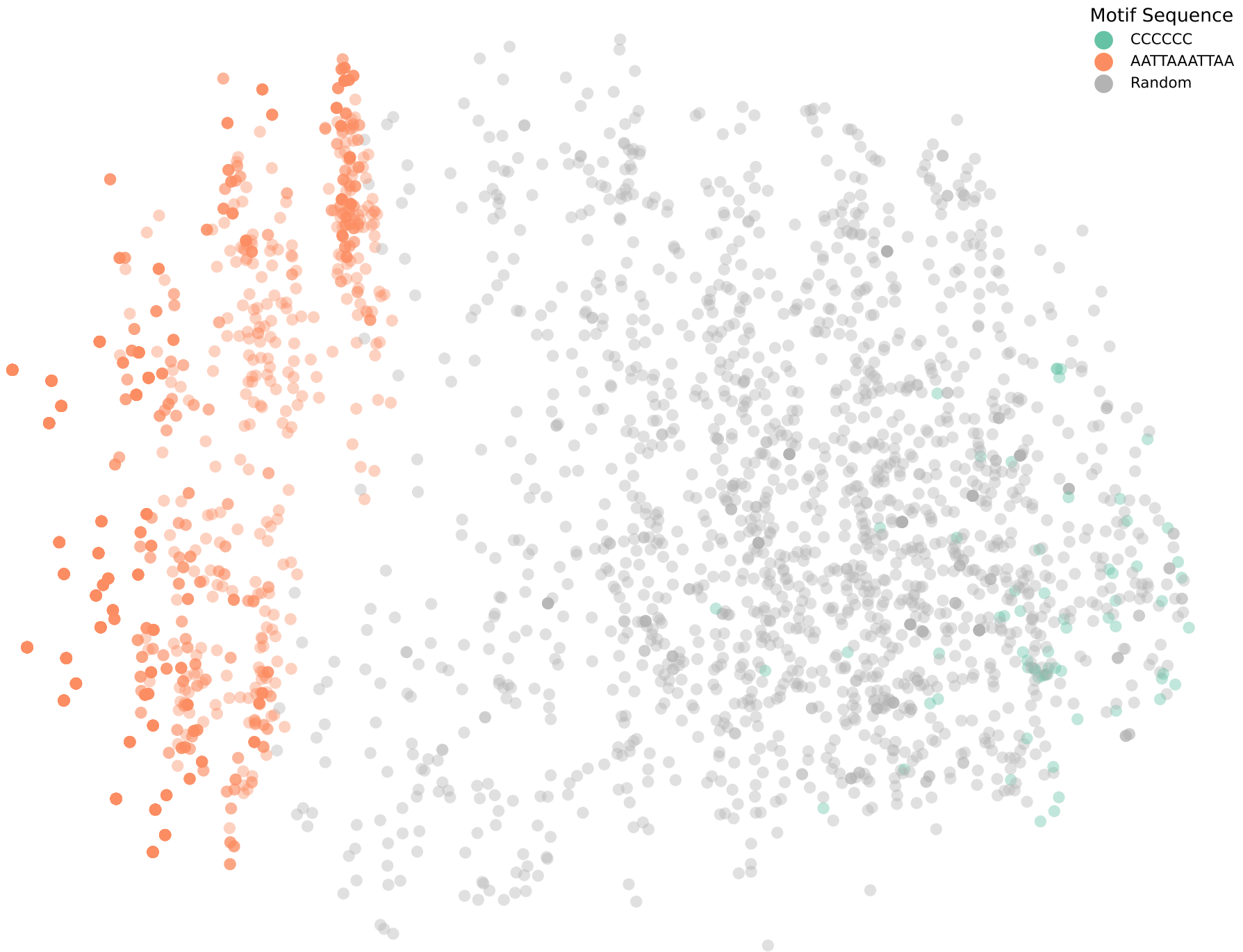

Supplement: Supplement 8 [file Supplemental_Data_1.zip › Supplemental_Data_1/Alx4_TGGTAG20NCG_P_4/Alx4_TGGTAG20NCG_P_4_PCA.pdf]

tSNE Plot - Alx4\_TGGTAG20NCG\_P\_4

Motif Sequence

- CCCCC
- AATTAAATTAA
- Random

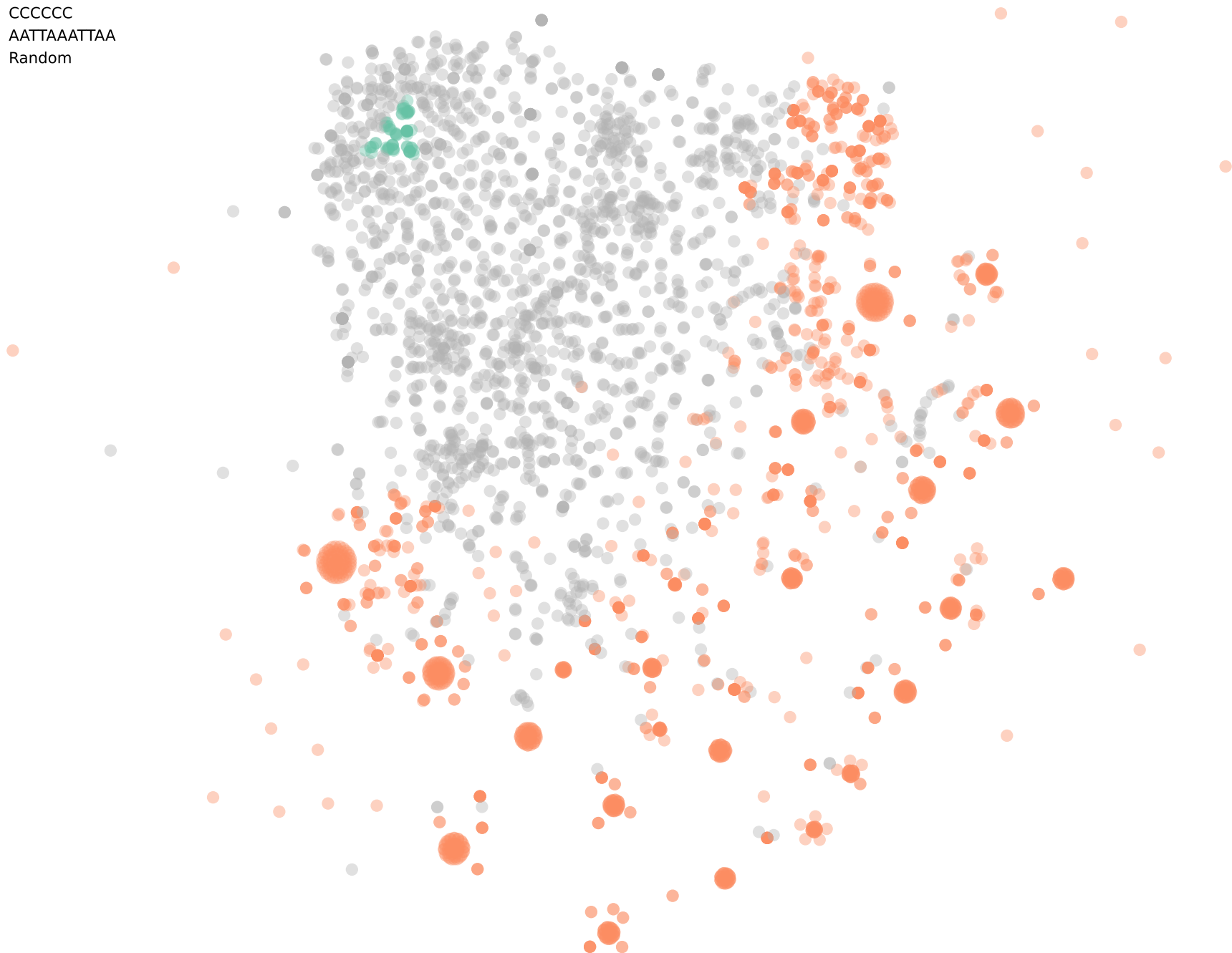

Supplement: Supplement 8 [file Supplemental_Data_1.zip › Supplemental_Data_1/Alx4_TGGTAG20NCG_P_4/Alx4_TGGTAG20NCG_P_4_tSNE.pdf]

UMAP Plot - Alx4\_TGGTAG20NCG\_P\_4

Motif Sequence

- CCCCC
- AATTAAATTAA
- Random

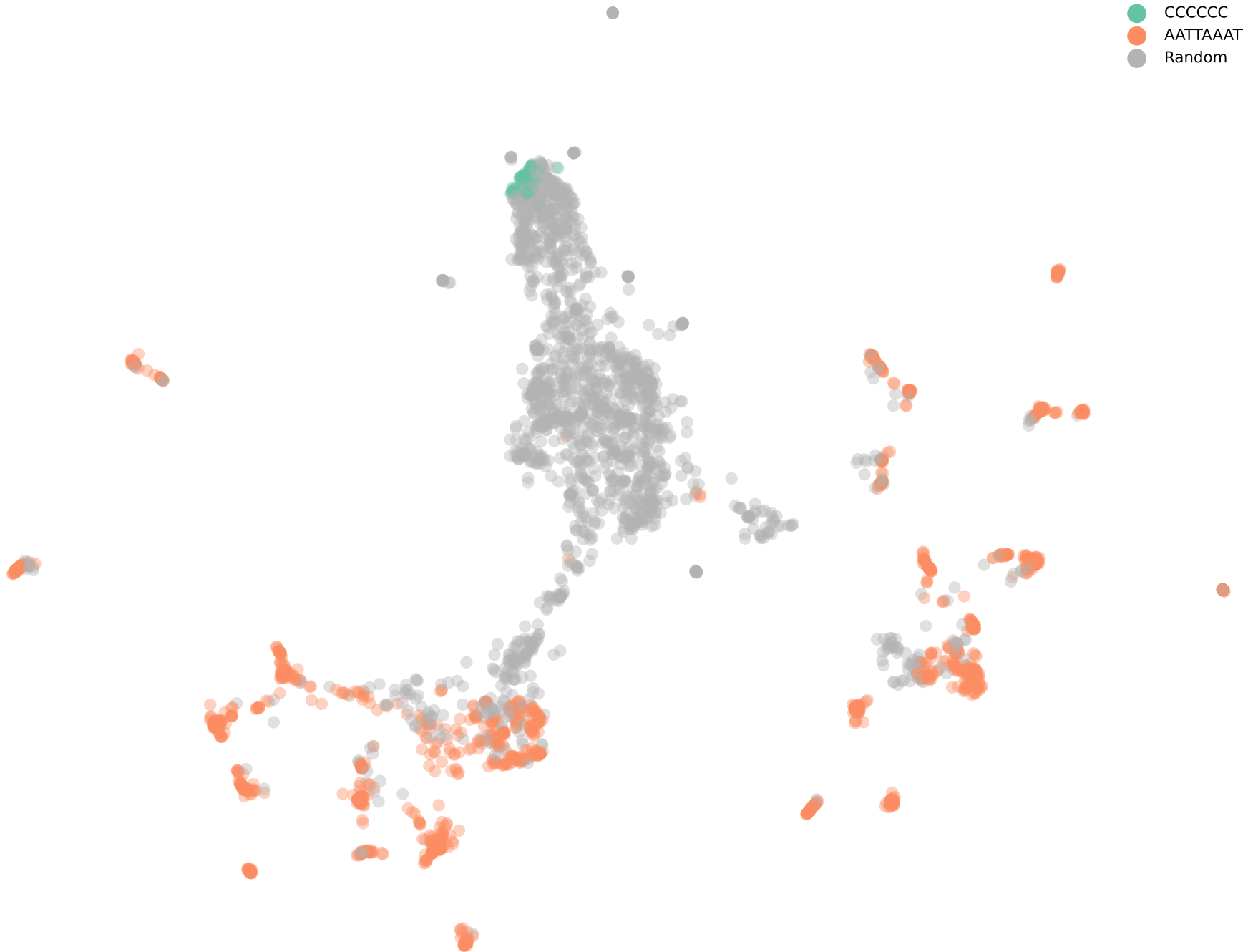

Supplement: Supplement 8 [file Supplemental_Data_1.zip › Supplemental_Data_1/Alx4_TGGTAG20NCG_P_4/Alx4_TGGTAG20NCG_P_4_UMAP.pdf]

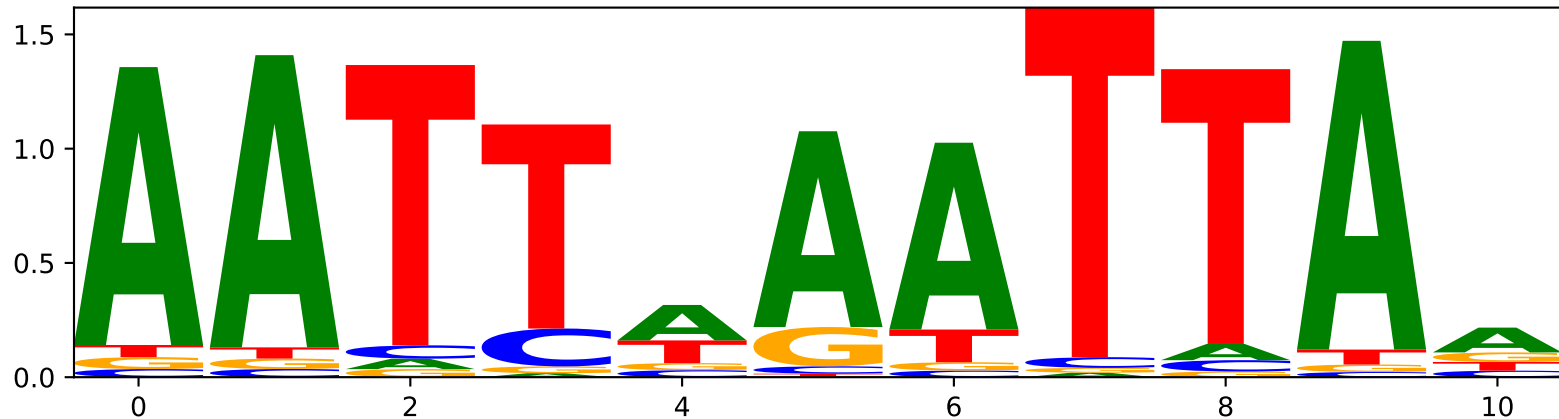

Supplement: Supplement 8 [file Supplemental_Data_1.zip › Supplemental_Data_1/Alx4_TGGTAG20NCG_P_4/kmap_logo.pdf]

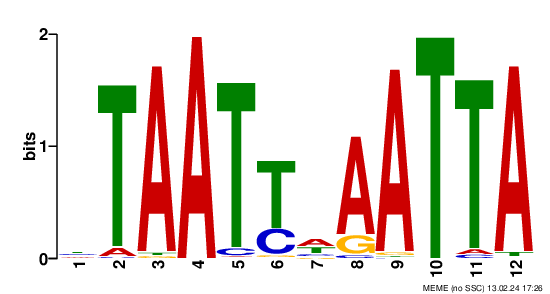

Supplement: Supplement 8 [file Supplemental_Data_1.zip › Supplemental_Data_1/Alx4_TGGTAG20NCG_P_4/meme_logo.png]

KMAP LD Plot - Alx4\_TGGTAG20NCG\_P\_5

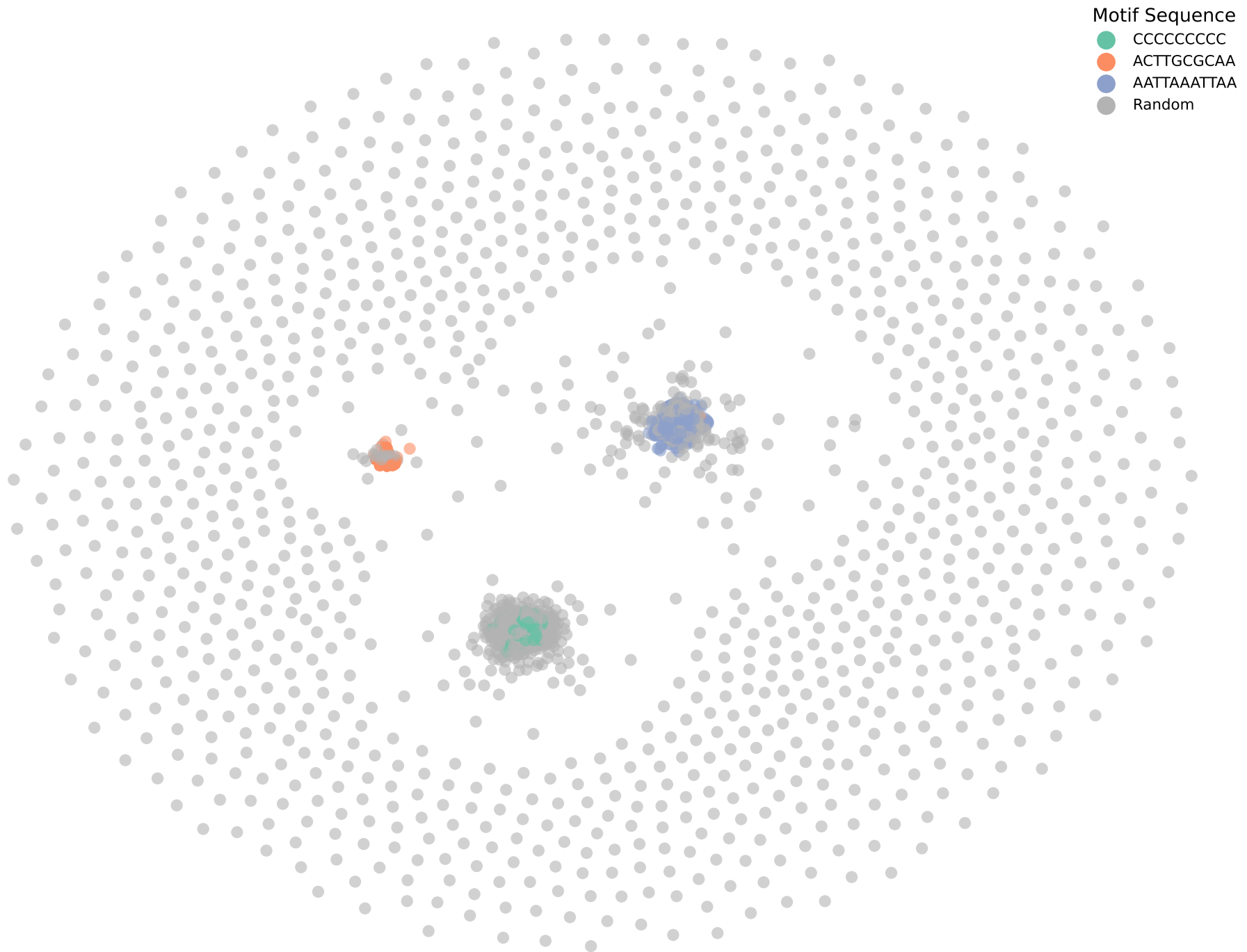

Supplement: Supplement 8 [file Supplemental_Data_1.zip › Supplemental_Data_1/Alx4_TGGTAG20NCG_P_5/Alx4_TGGTAG20NCG_P_5_KMAP.pdf]

MDS Plot - Alx4\_TGGTAG20NCG\_P\_5

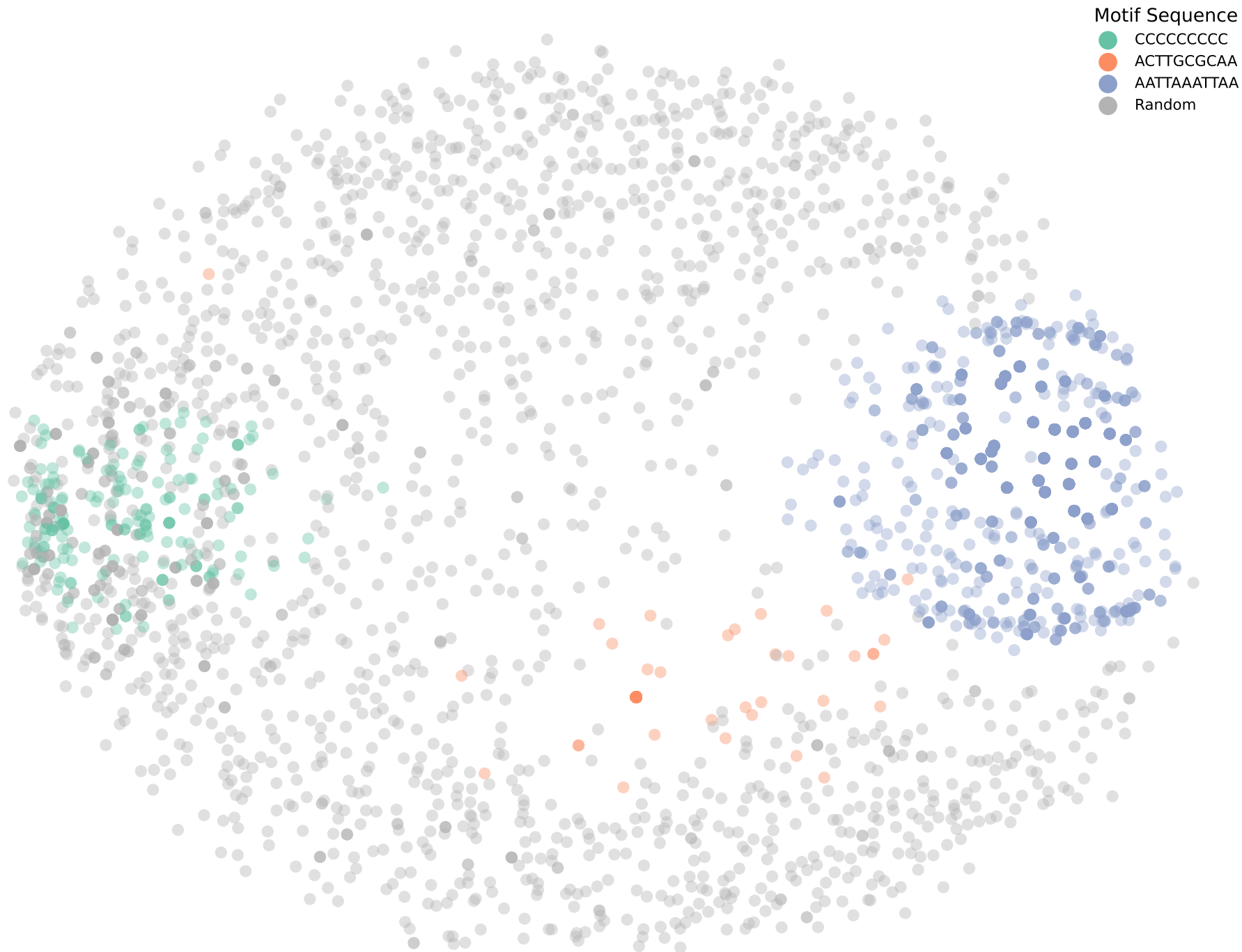

Supplement: Supplement 8 [file Supplemental_Data_1.zip › Supplemental_Data_1/Alx4_TGGTAG20NCG_P_5/Alx4_TGGTAG20NCG_P_5_MDS.pdf]

PCA Plot - Alx4\_TGGTAG20NCG\_P\_5

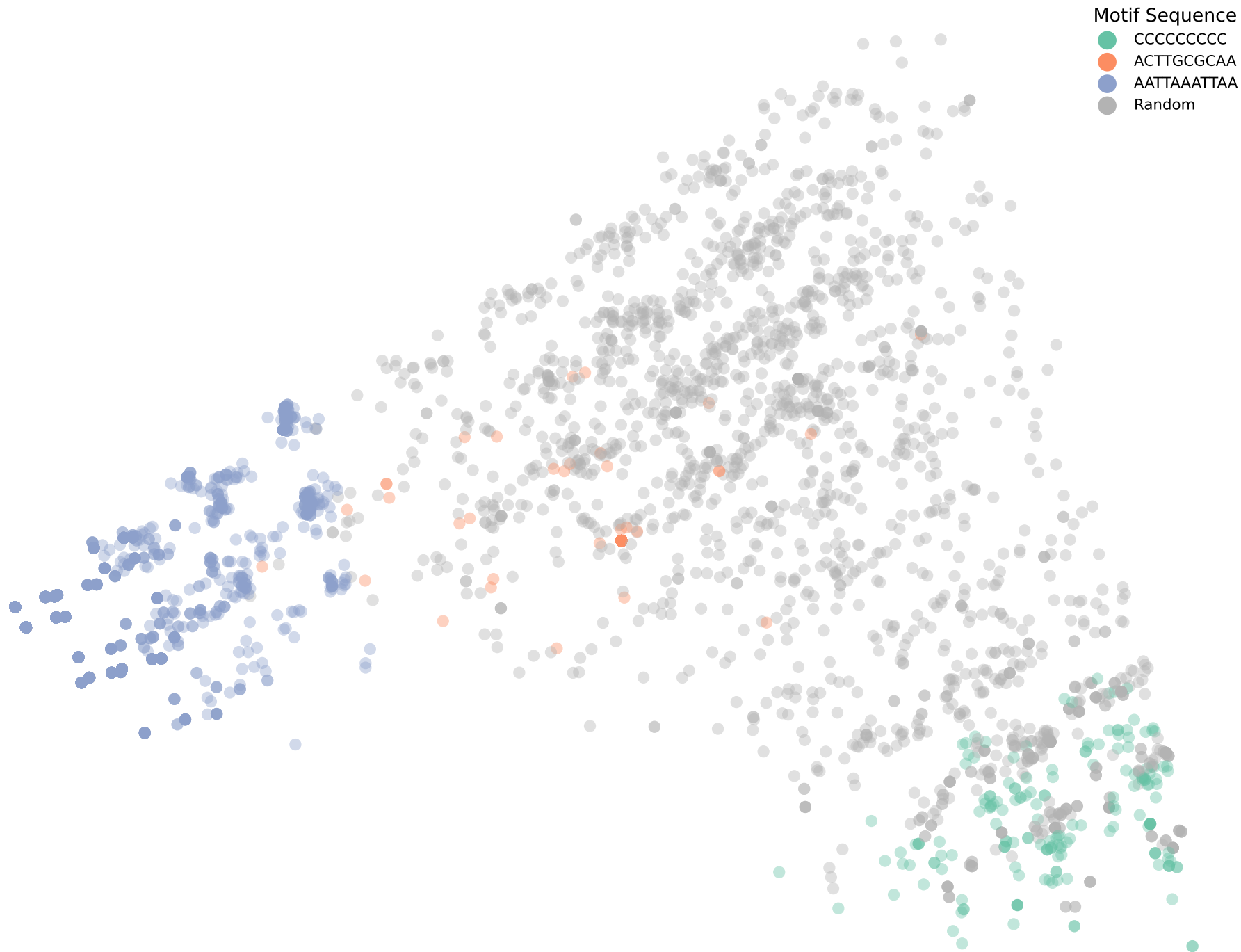

Supplement: Supplement 8 [file Supplemental_Data_1.zip › Supplemental_Data_1/Alx4_TGGTAG20NCG_P_5/Alx4_TGGTAG20NCG_P_5_PCA.pdf]

tSNE Plot - Alx4\_TGGTAG20NCG\_P\_5

Motif Sequence

- CCCCCCCC
- ACTTGCGCAA
- AATTAAATTAA
- Random

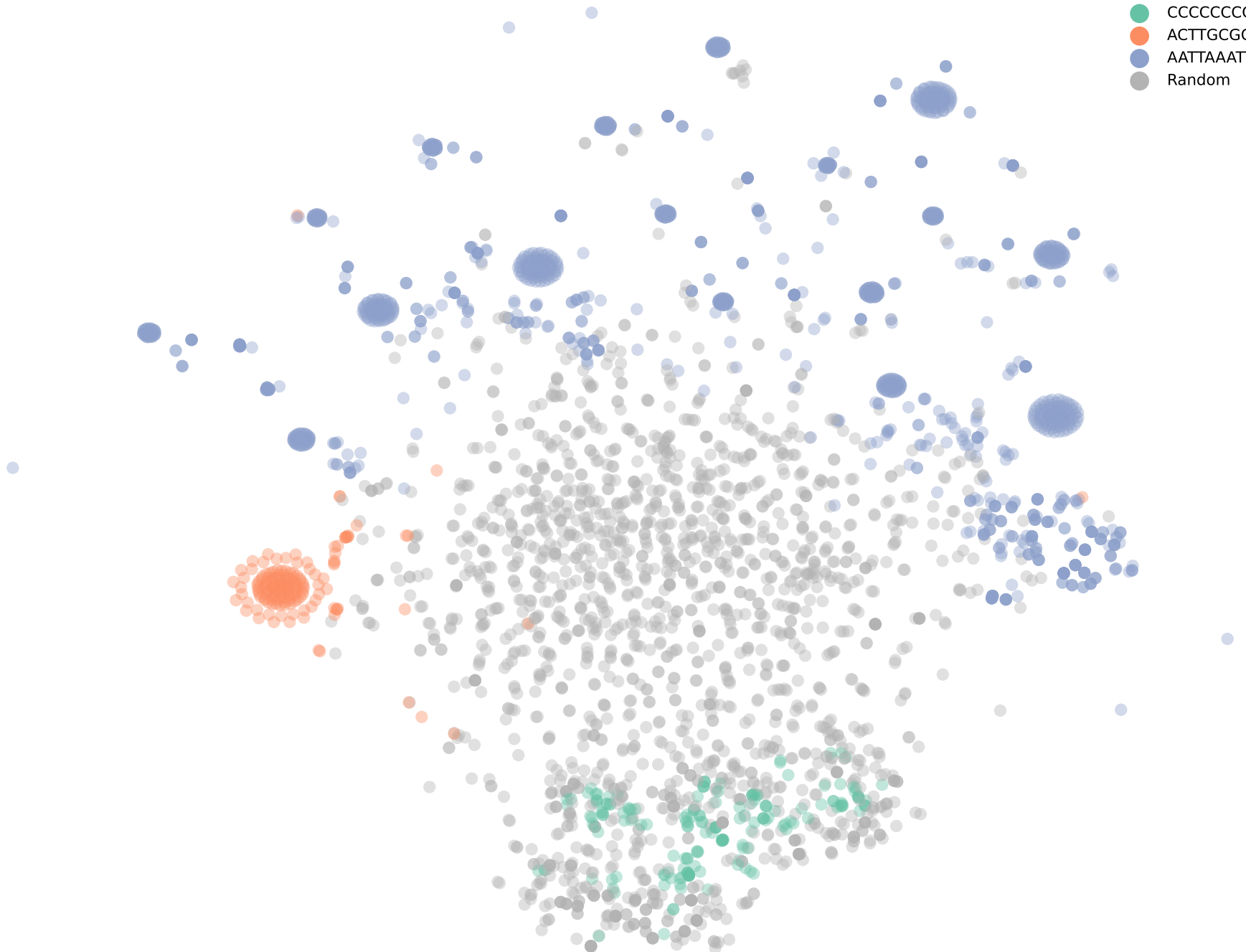

Supplement: Supplement 8 [file Supplemental_Data_1.zip › Supplemental_Data_1/Alx4_TGGTAG20NCG_P_5/Alx4_TGGTAG20NCG_P_5_tSNE.pdf]

UMAP Plot - Alx4\_TGGTAG20NCG\_P\_5

Motif Sequence

- CCCCCCCCC
- ACTTGCGCAA
- AATTAAATTAA
- Random

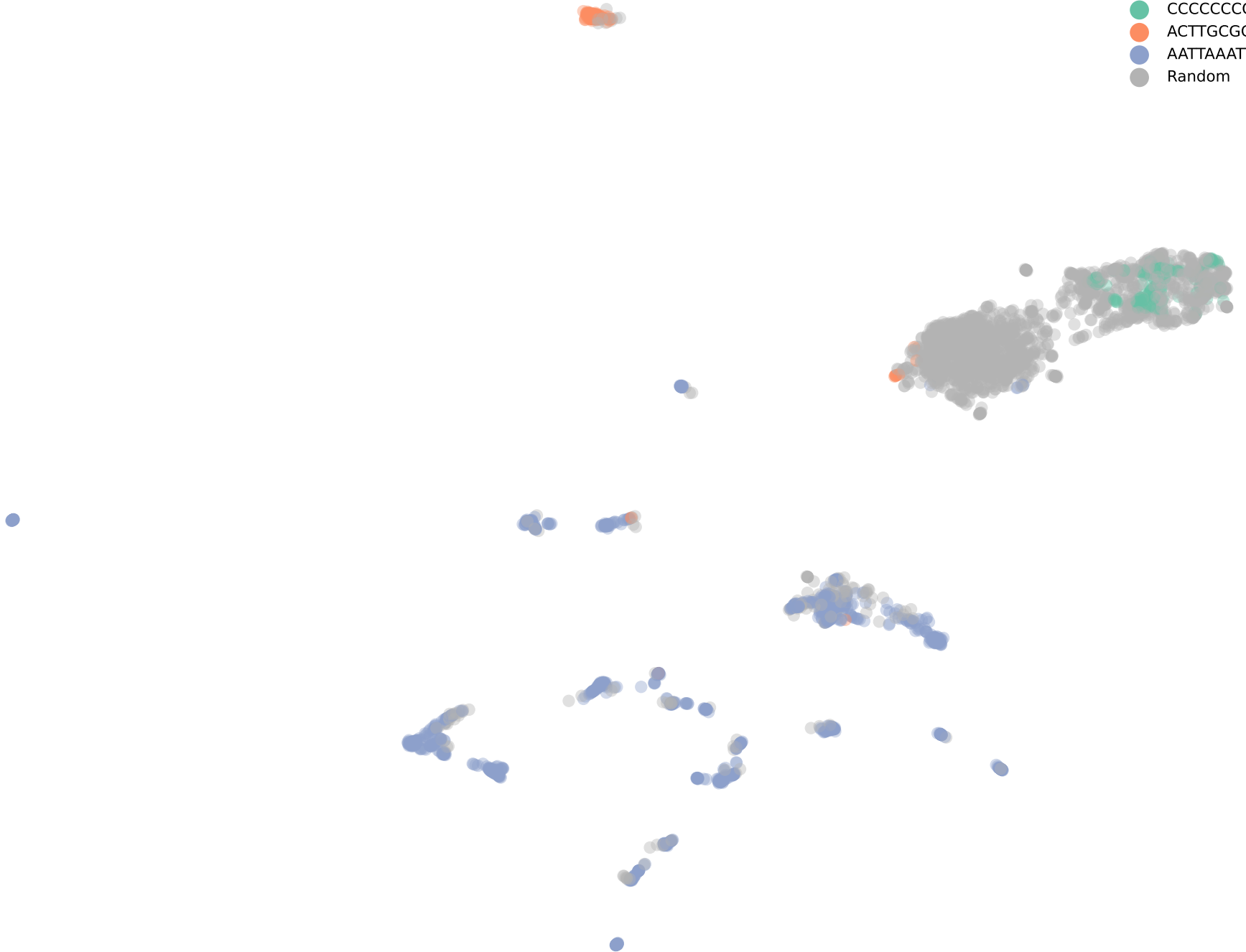

Supplement: Supplement 8 [file Supplemental_Data_1.zip › Supplemental_Data_1/Alx4_TGGTAG20NCG_P_5/Alx4_TGGTAG20NCG_P_5_UMAP.pdf]

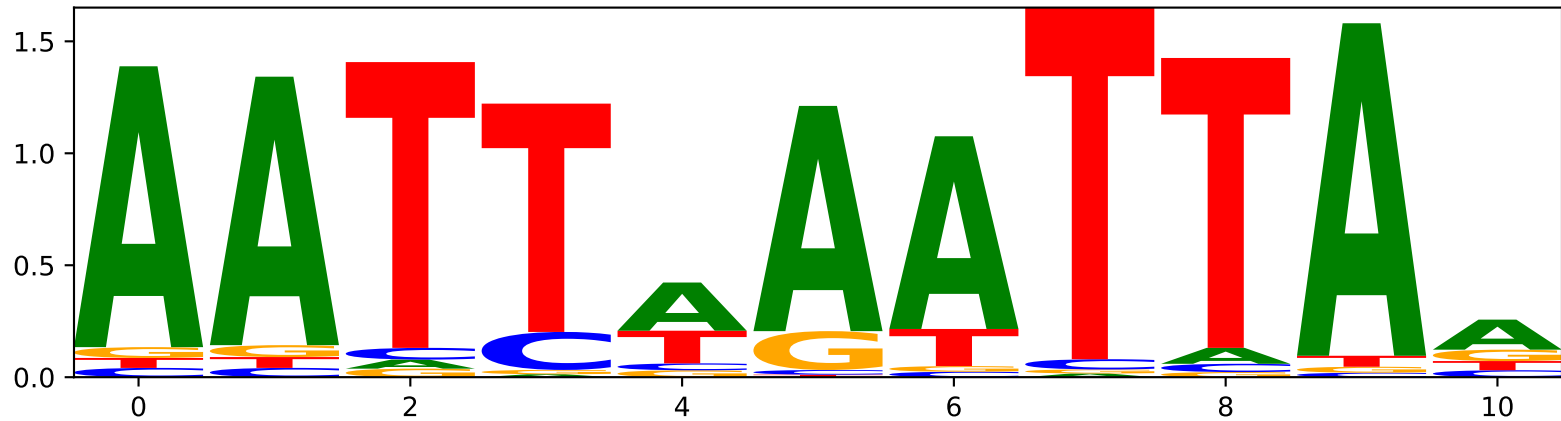

Supplement: Supplement 8 [file Supplemental_Data_1.zip › Supplemental_Data_1/Alx4_TGGTAG20NCG_P_5/kmap_logo.pdf]

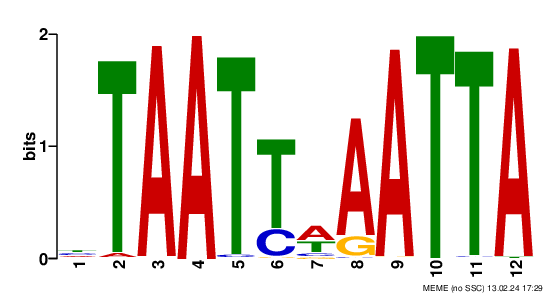

Supplement: Supplement 8 [file Supplemental_Data_1.zip › Supplemental_Data_1/Alx4_TGGTAG20NCG_P_5/meme_logo.png]

KMAP LD Plot - ALX4\_TGTGTC20NGA\_W\_3

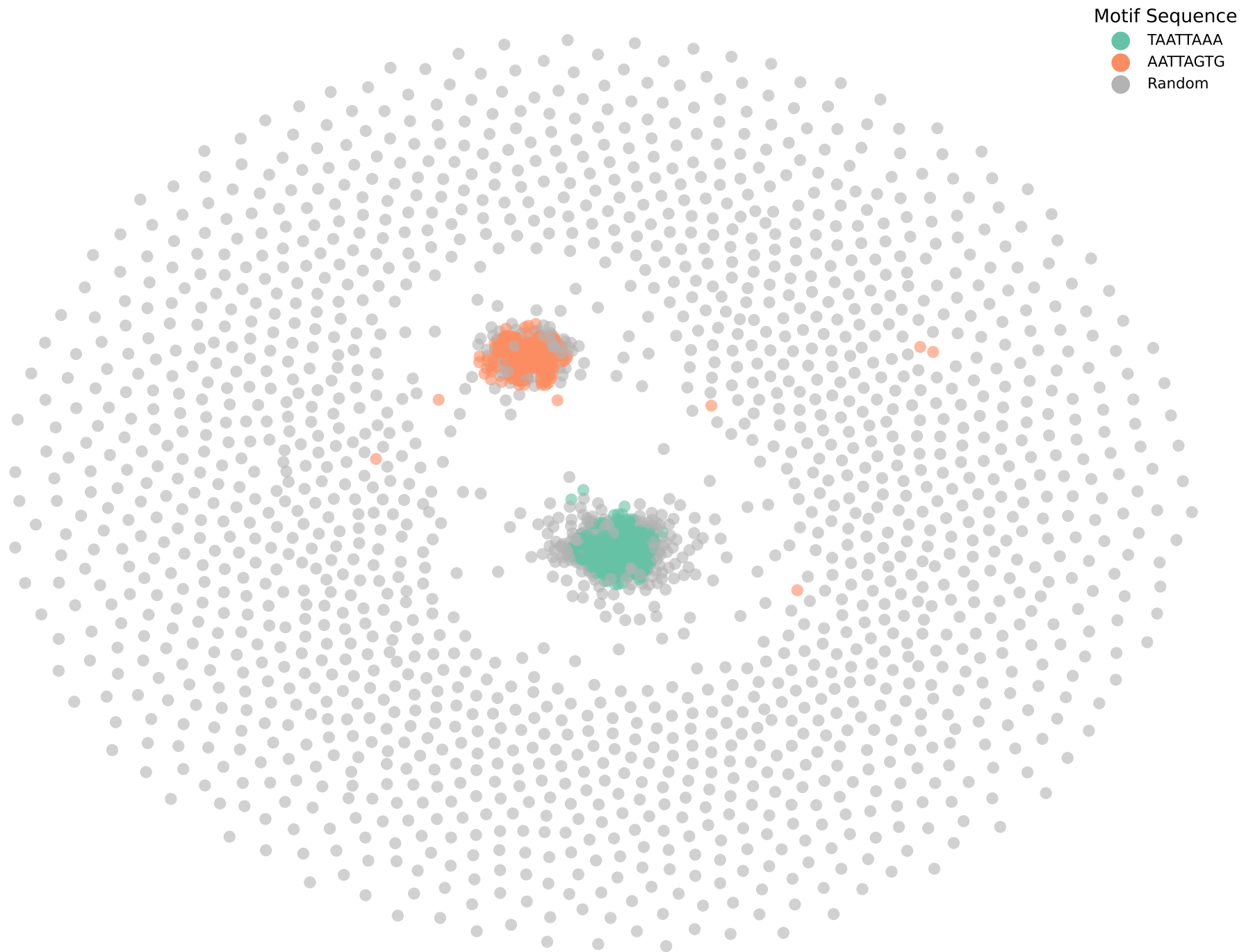

Supplement: Supplement 8 [file Supplemental_Data_1.zip › Supplemental_Data_1/ALX4_TGTGTC20NGA_W_3/ALX4_TGTGTC20NGA_W_3_KMAP.pdf]

MDS Plot - ALX4\_TGTGTC20NGA\_W\_3

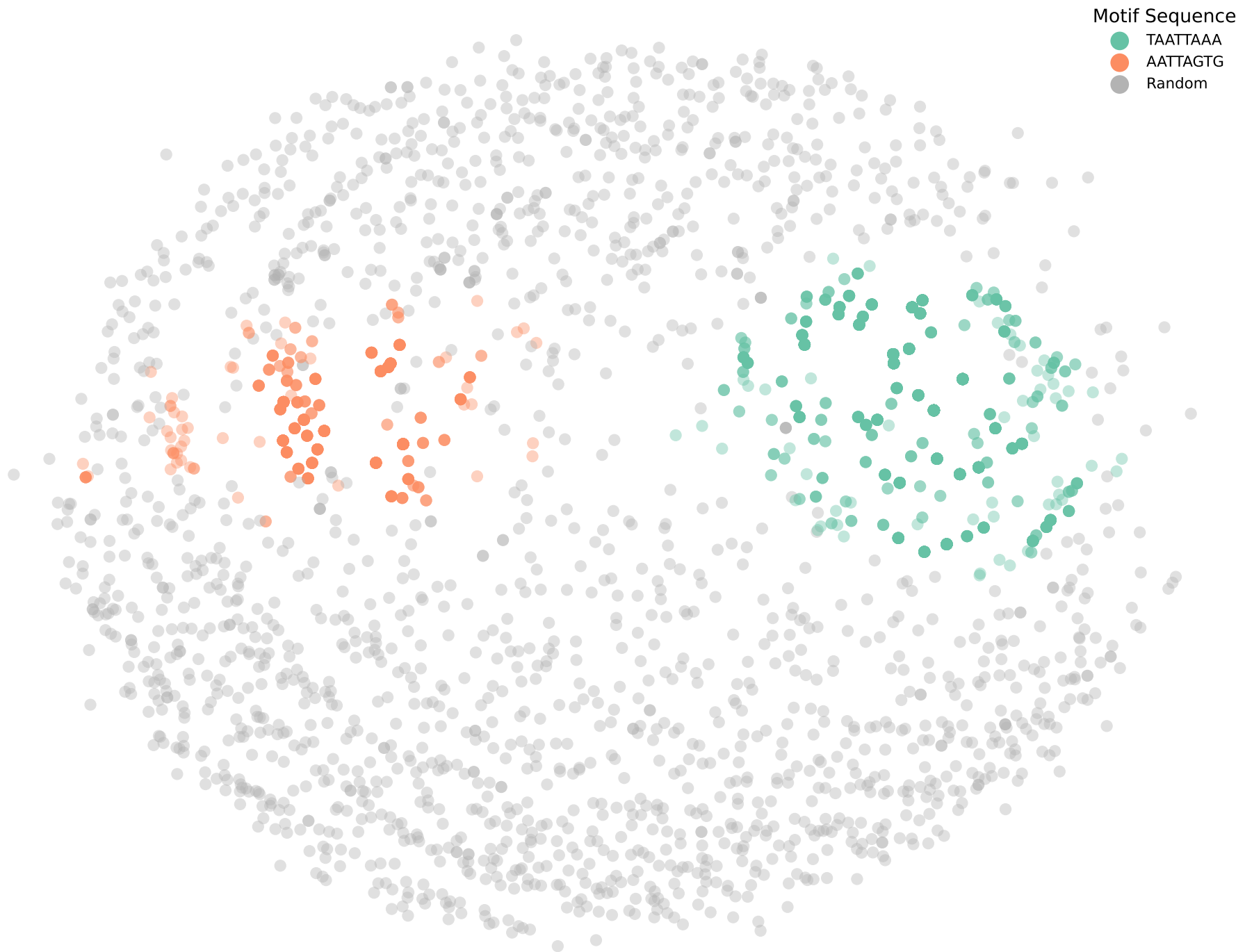

Supplement: Supplement 8 [file Supplemental_Data_1.zip › Supplemental_Data_1/ALX4_TGTGTC20NGA_W_3/ALX4_TGTGTC20NGA_W_3_MDS.pdf]

PCA Plot - ALX4\_TGTGTC20NGA\_W\_3

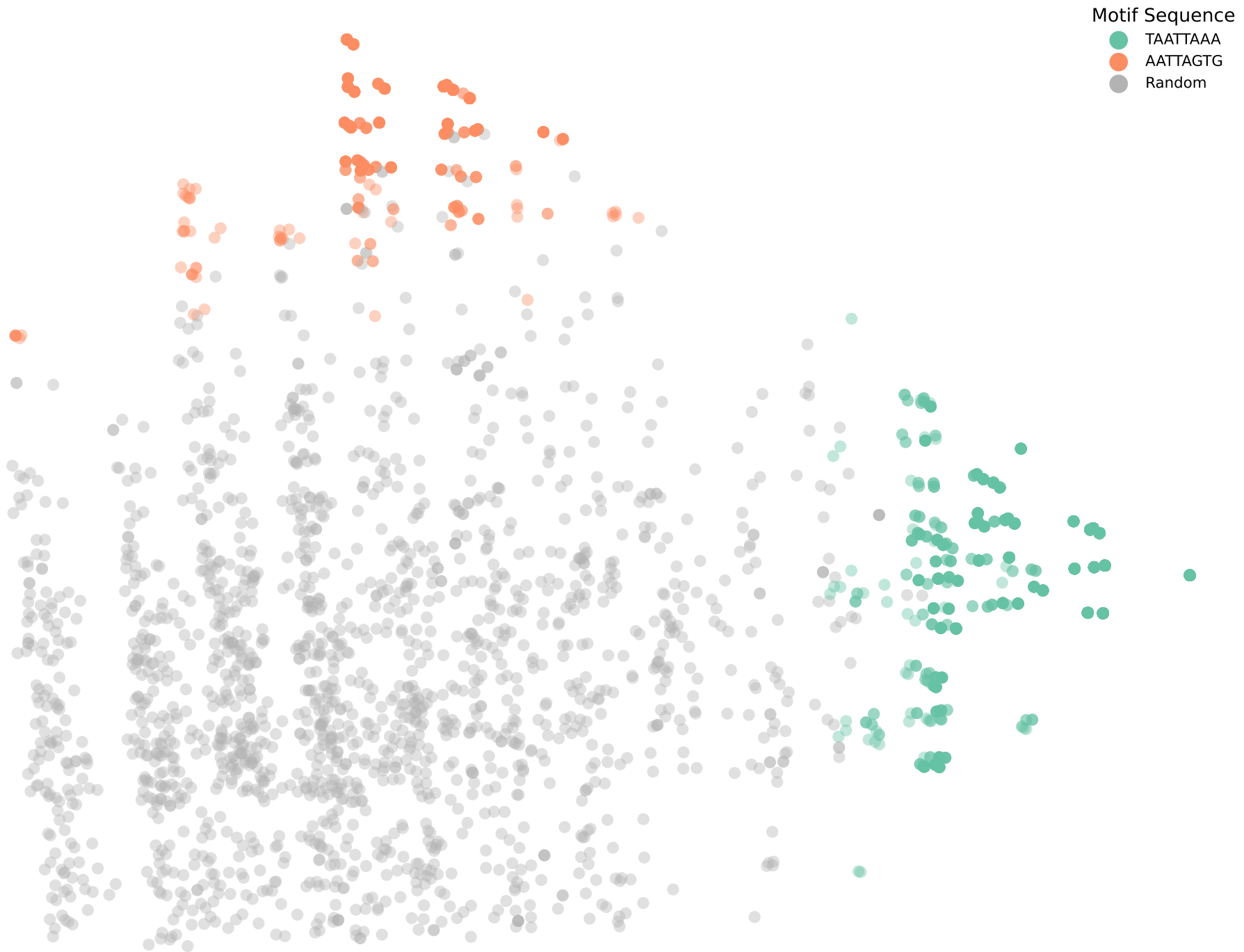

Supplement: Supplement 8 [file Supplemental_Data_1.zip › Supplemental_Data_1/ALX4_TGTGTC20NGA_W_3/ALX4_TGTGTC20NGA_W_3_PCA.pdf]

tSNE Plot - ALX4\_TGTGTC20NGA\_W\_3

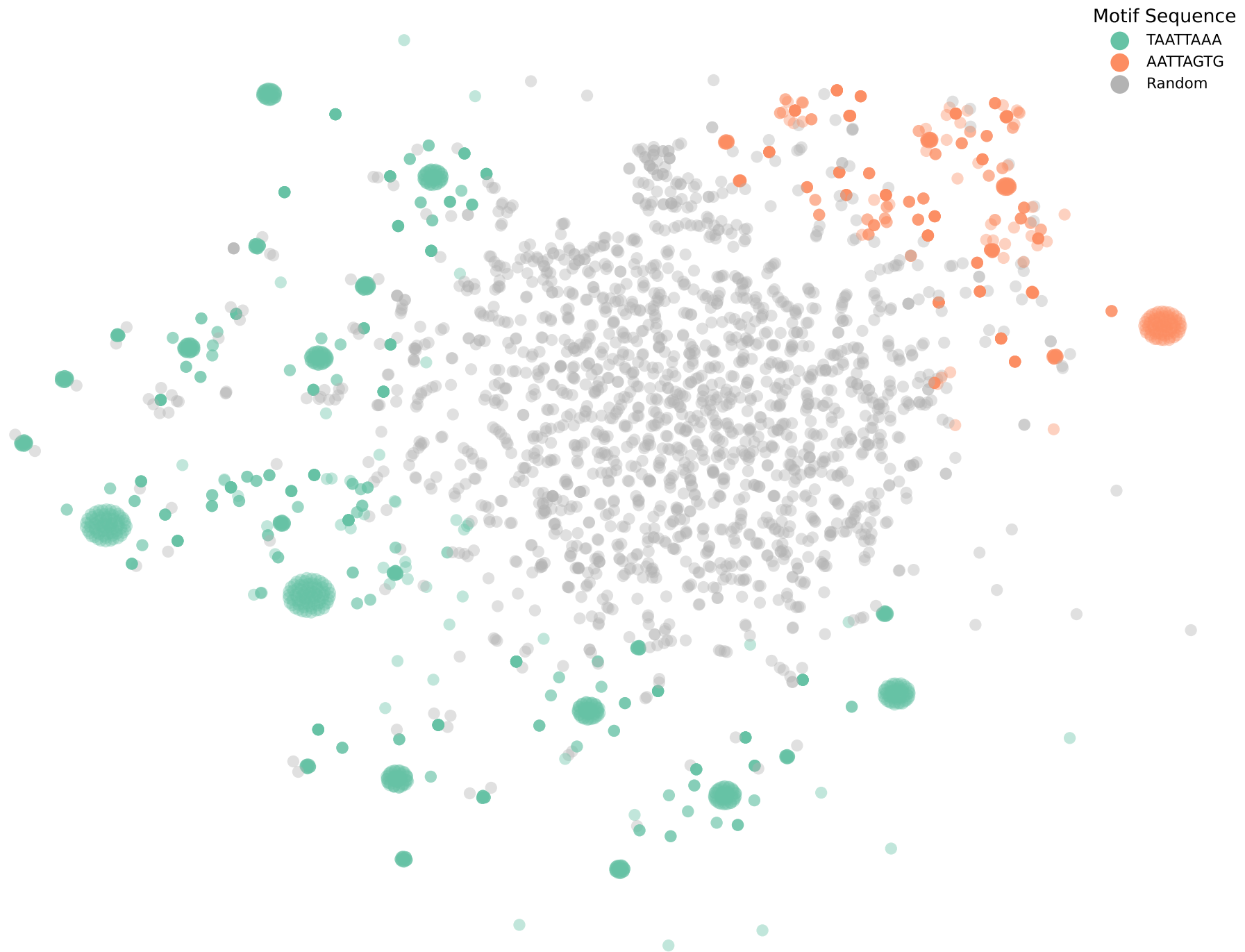

Supplement: Supplement 8 [file Supplemental_Data_1.zip › Supplemental_Data_1/ALX4_TGTGTC20NGA_W_3/ALX4_TGTGTC20NGA_W_3_tSNE.pdf]

UMAP Plot - ALX4\_TGTGTC20NGA\_W\_3

Motif Sequence

- TAATTAAA
- AATTAGTG
- Random

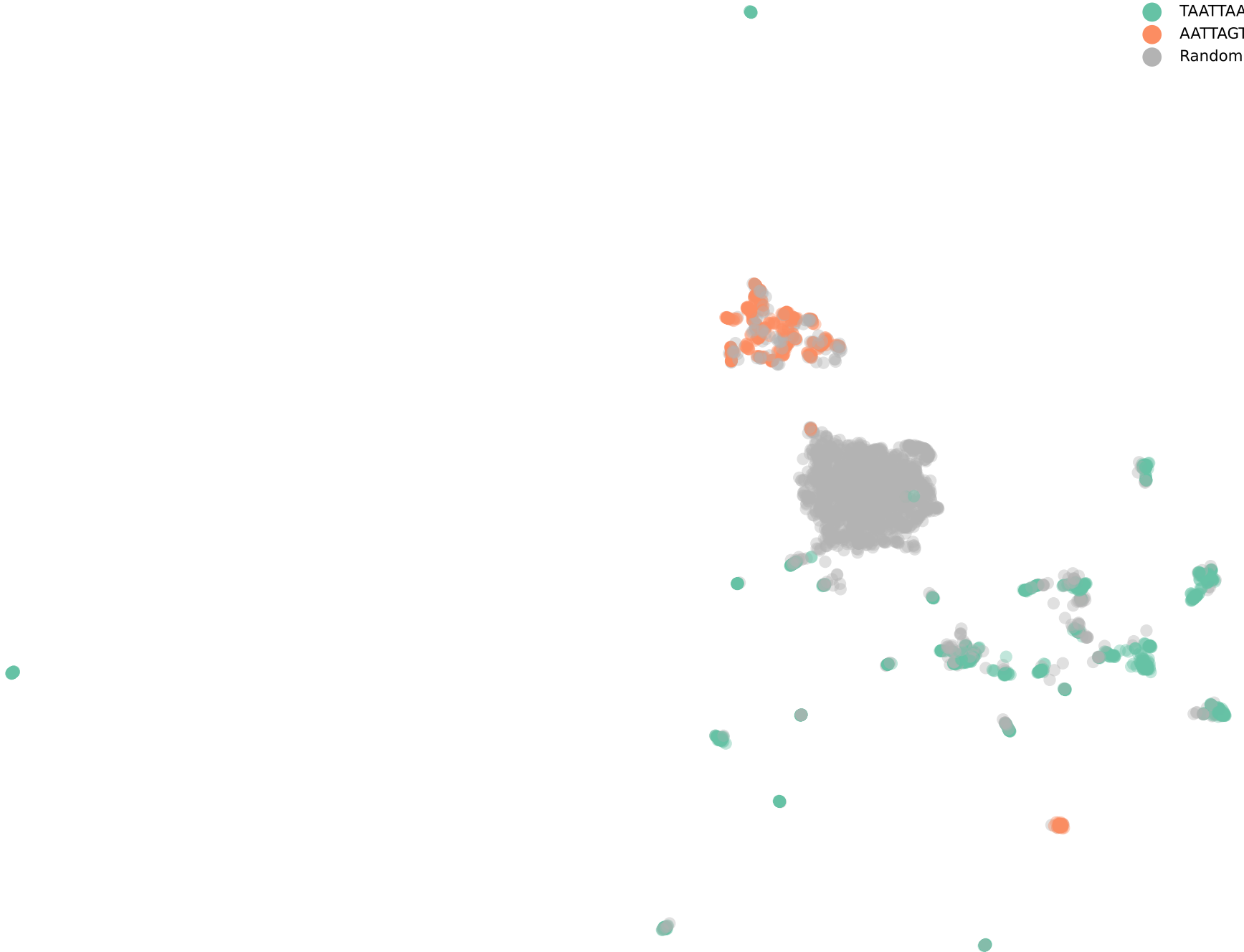

Supplement: Supplement 8 [file Supplemental_Data_1.zip › Supplemental_Data_1/ALX4_TGTGTC20NGA_W_3/ALX4_TGTGTC20NGA_W_3_UMAP.pdf]

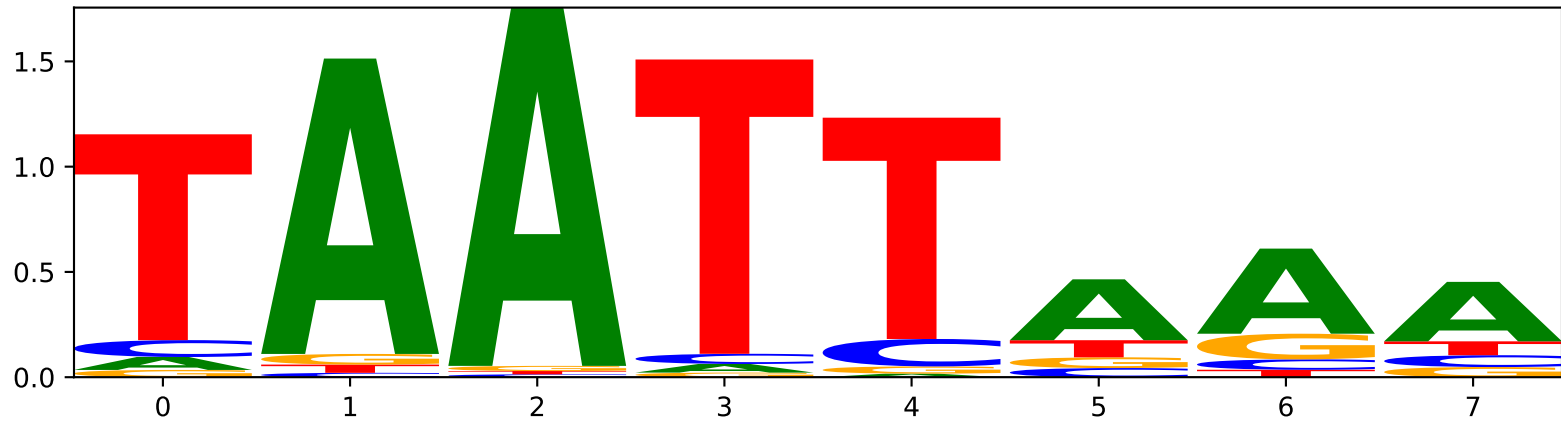

Supplement: Supplement 8 [file Supplemental_Data_1.zip › Supplemental_Data_1/ALX4_TGTGTC20NGA_W_3/kmap_logo.pdf]

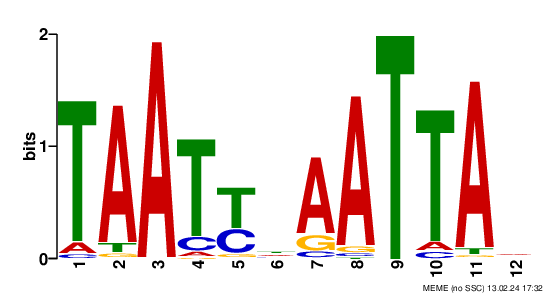

Supplement: Supplement 8 [file Supplemental_Data_1.zip › Supplemental_Data_1/ALX4_TGTGTC20NGA_W_3/meme_logo.png]

KMAP LD Plot - ALX4\_TGTGTC20NGA\_W\_4

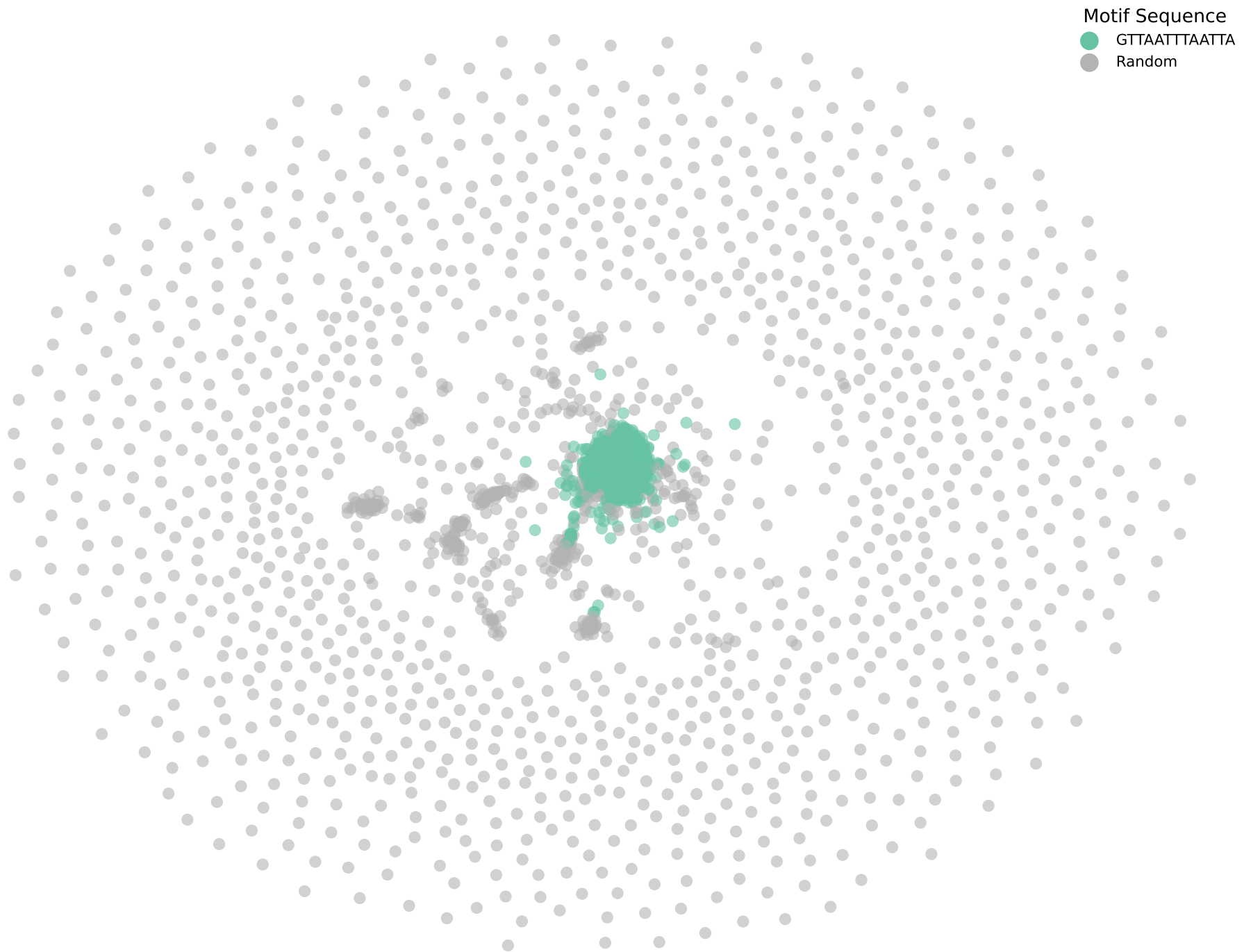

Supplement: Supplement 8 [file Supplemental_Data_1.zip › Supplemental_Data_1/ALX4_TGTGTC20NGA_W_4/ALX4_TGTGTC20NGA_W_4_KMAP.pdf]

MDS Plot - ALX4\_TGTGTC20NGA\_W\_4

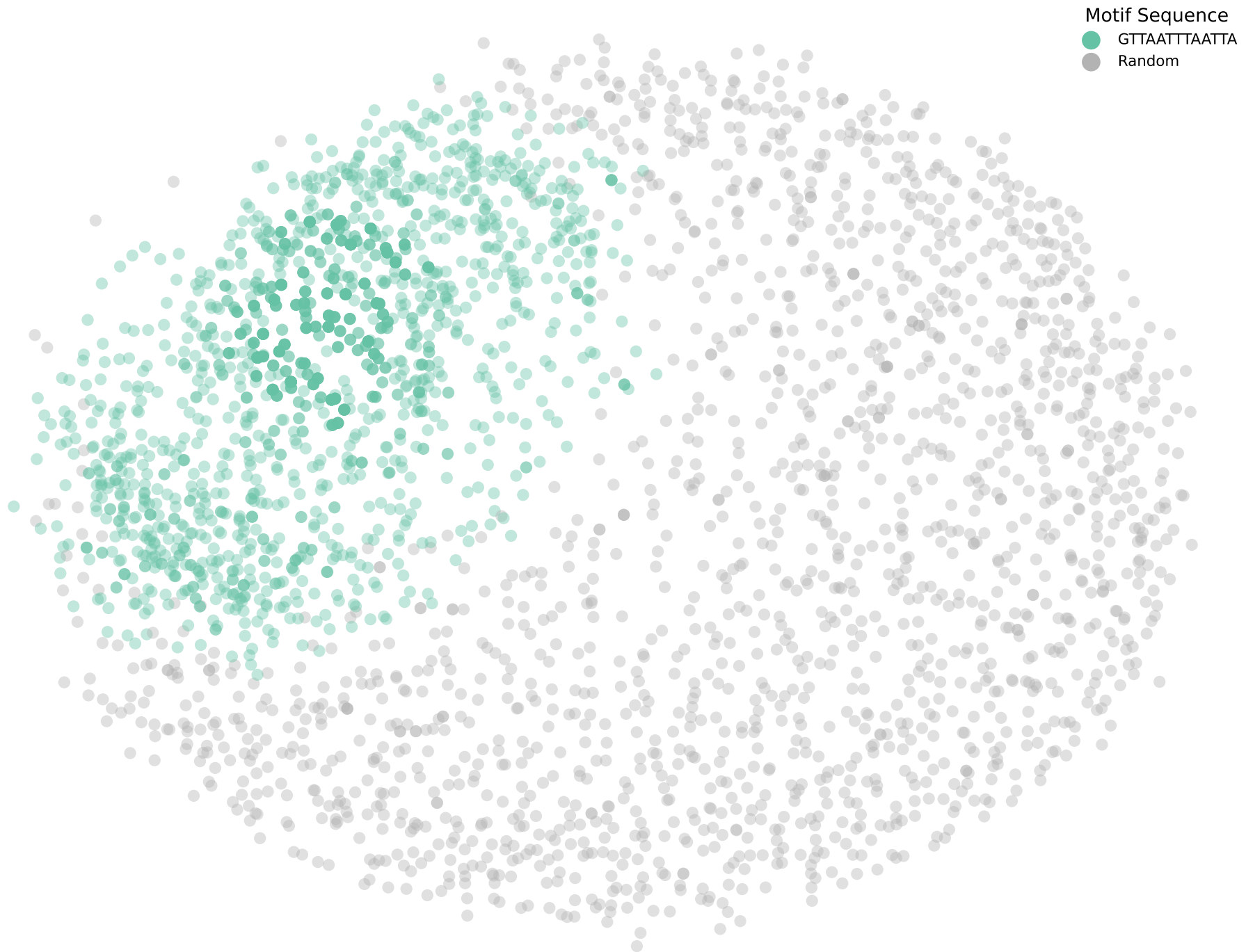

Supplement: Supplement 8 [file Supplemental_Data_1.zip › Supplemental_Data_1/ALX4_TGTGTC20NGA_W_4/ALX4_TGTGTC20NGA_W_4_MDS.pdf]

PCA Plot - ALX4\_TGTGTC20NGA\_W\_4

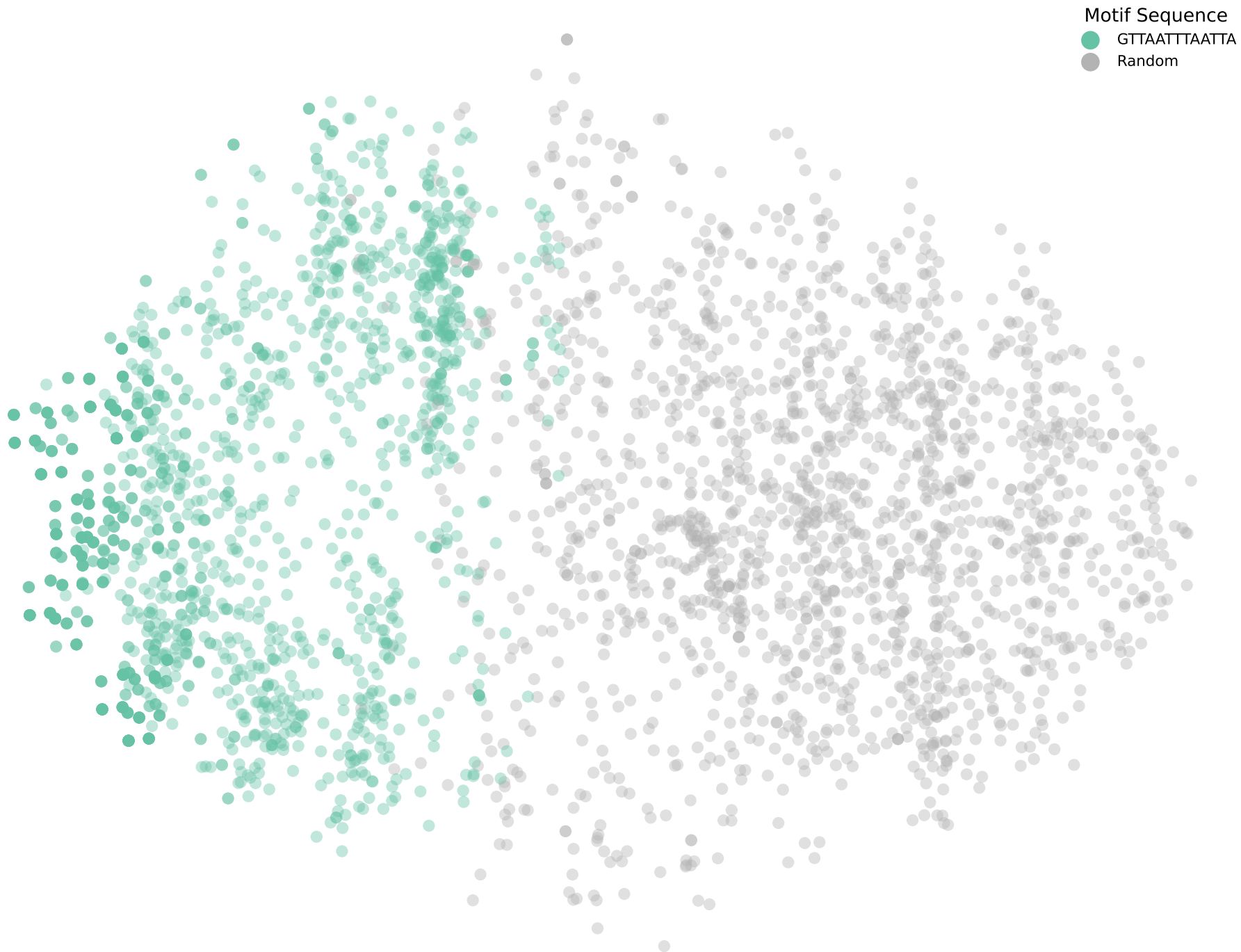

Supplement: Supplement 8 [file Supplemental_Data_1.zip › Supplemental_Data_1/ALX4_TGTGTC20NGA_W_4/ALX4_TGTGTC20NGA_W_4_PCA.pdf]

tSNE Plot - ALX4\_TGTGTC20NGA\_W\_4

Motif Sequence

● GTTAATTTAATTA

● Random

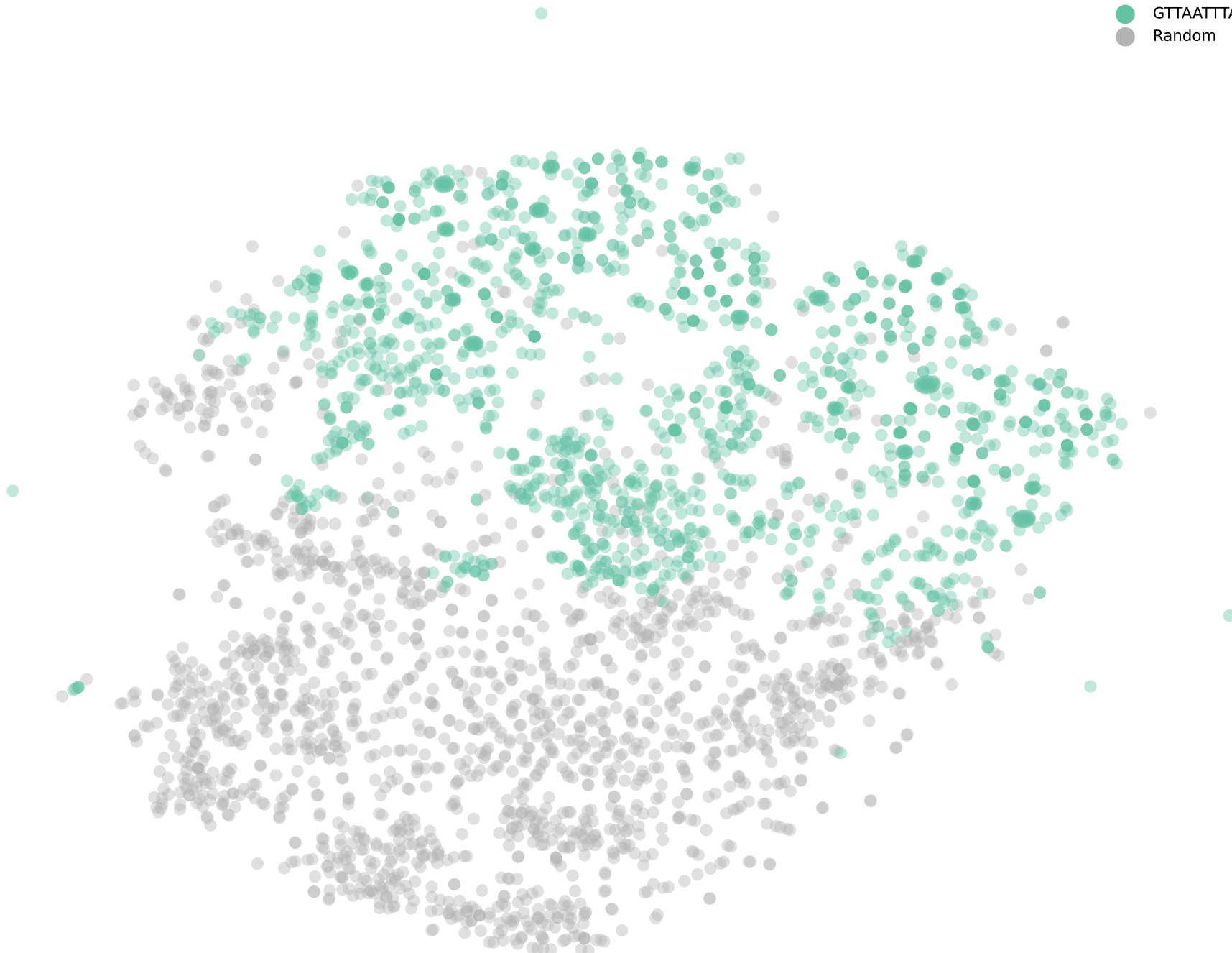

Supplement: Supplement 8 [file Supplemental_Data_1.zip › Supplemental_Data_1/ALX4_TGTGTC20NGA_W_4/ALX4_TGTGTC20NGA_W_4_tSNE.pdf]

UMAP Plot - ALX4\_TGTGTC20NGA\_W\_4

Motif Sequence

- GTTAATTTAATTA
- Random

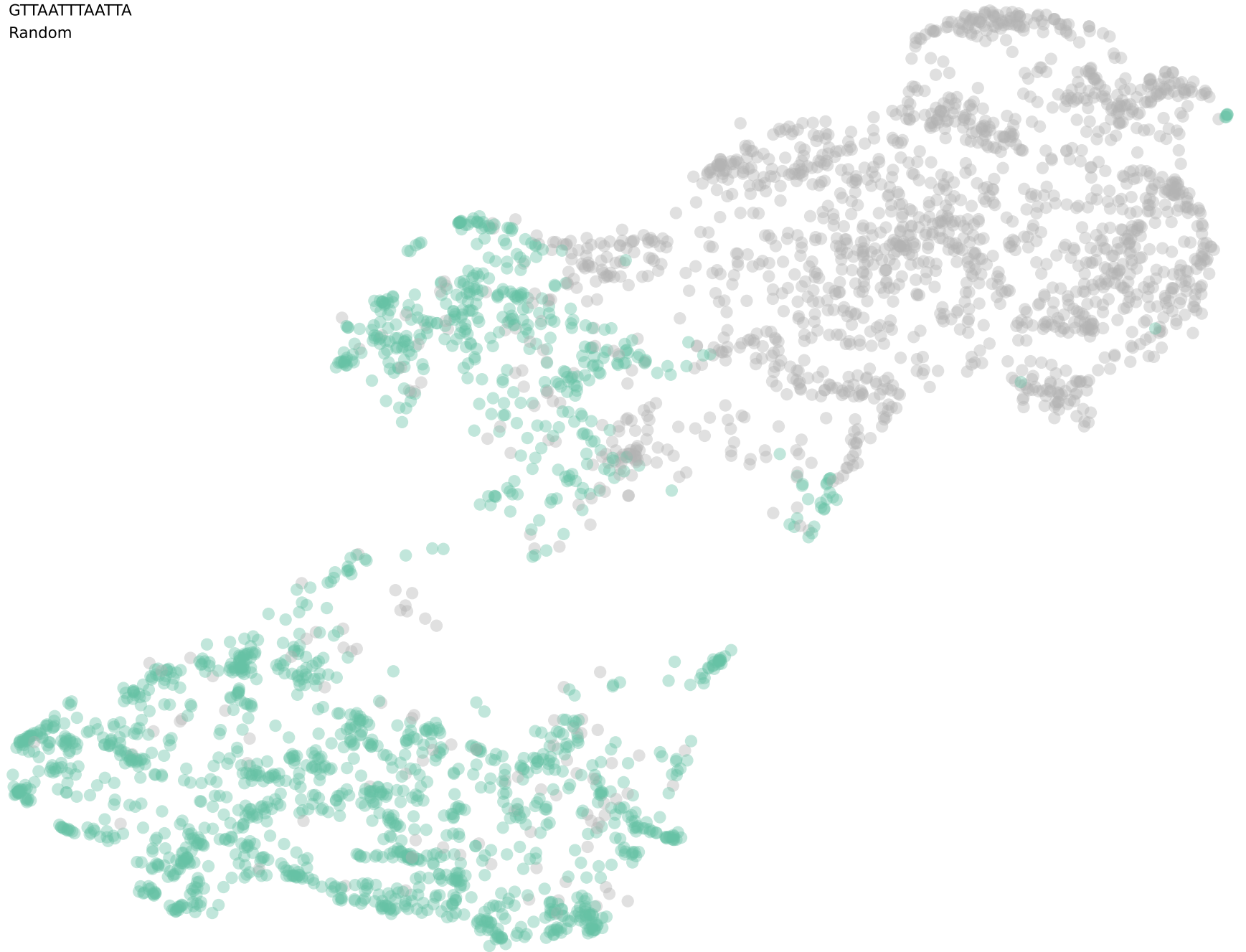

Supplement: Supplement 8 [file Supplemental_Data_1.zip › Supplemental_Data_1/ALX4_TGTGTC20NGA_W_4/ALX4_TGTGTC20NGA_W_4_UMAP.pdf]

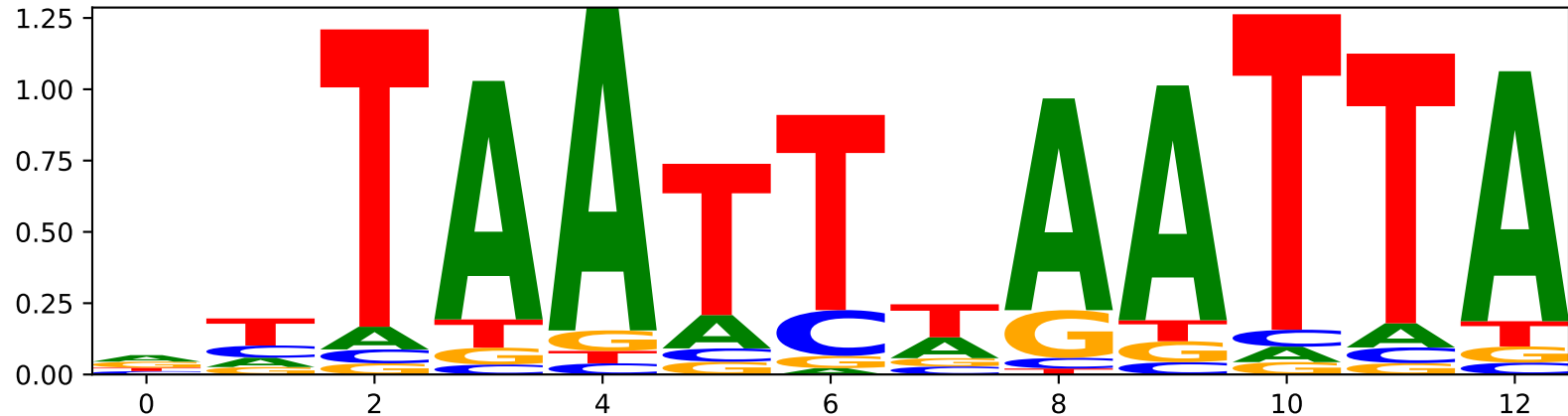

Supplement: Supplement 8 [file Supplemental_Data_1.zip › Supplemental_Data_1/ALX4_TGTGTC20NGA_W_4/kmap_logo.pdf]

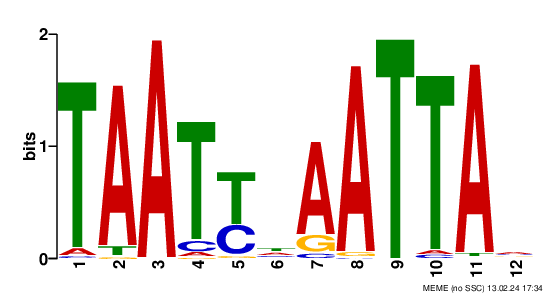

Supplement: Supplement 8 [file Supplemental_Data_1.zip › Supplemental_Data_1/ALX4_TGTGTC20NGA_W_4/meme_logo.png]

KMAP LD Plot - ARNTL\_TCAAAA20NCG\_W\_3

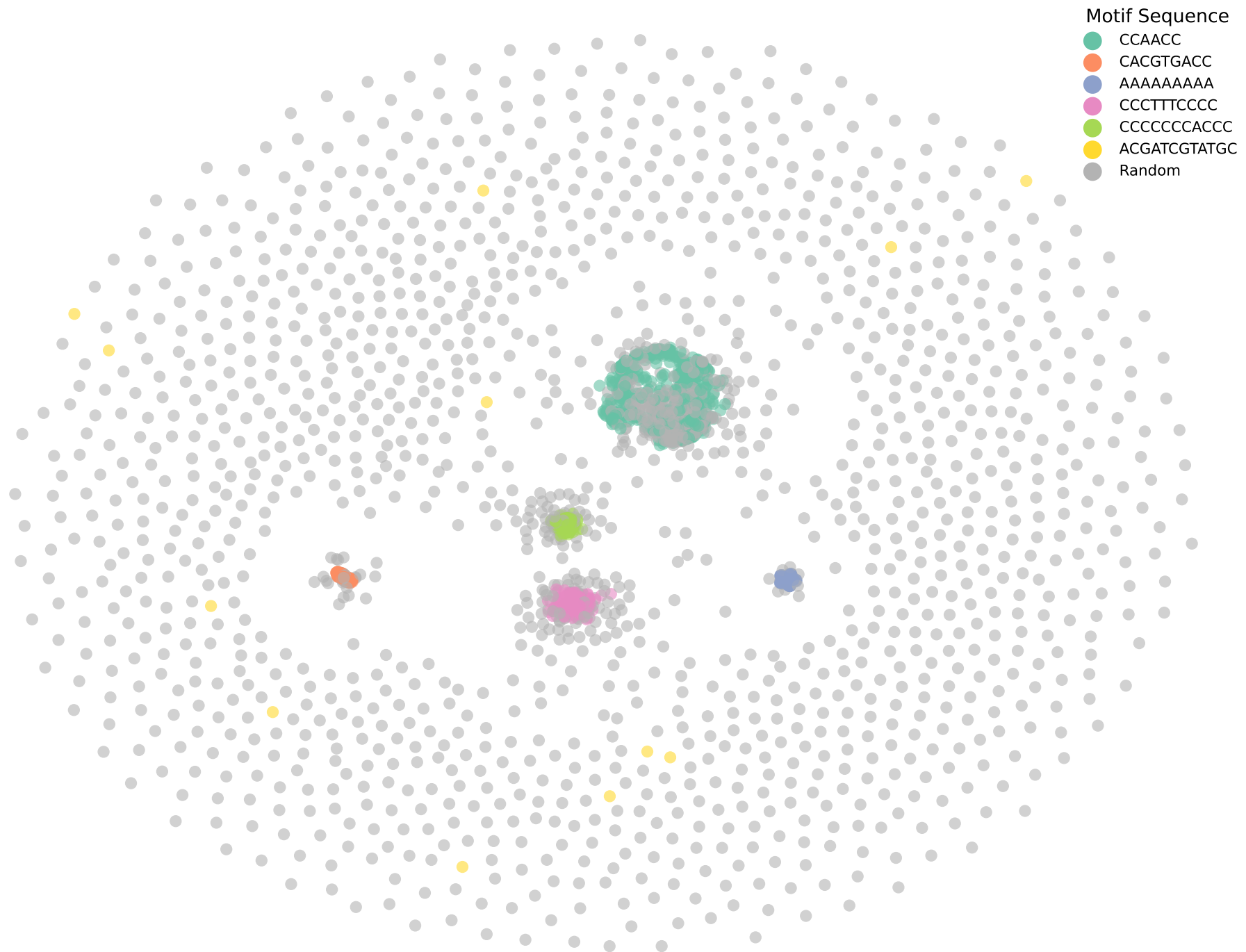

Supplement: Supplement 8 [file Supplemental_Data_1.zip › Supplemental_Data_1/ARNTL_TCAAAA20NCG_W_3/ARNTL_TCAAAA20NCG_W_3_KMAP.pdf]

MDS Plot - ARNTL\_TCAAAA20NCG\_W\_3

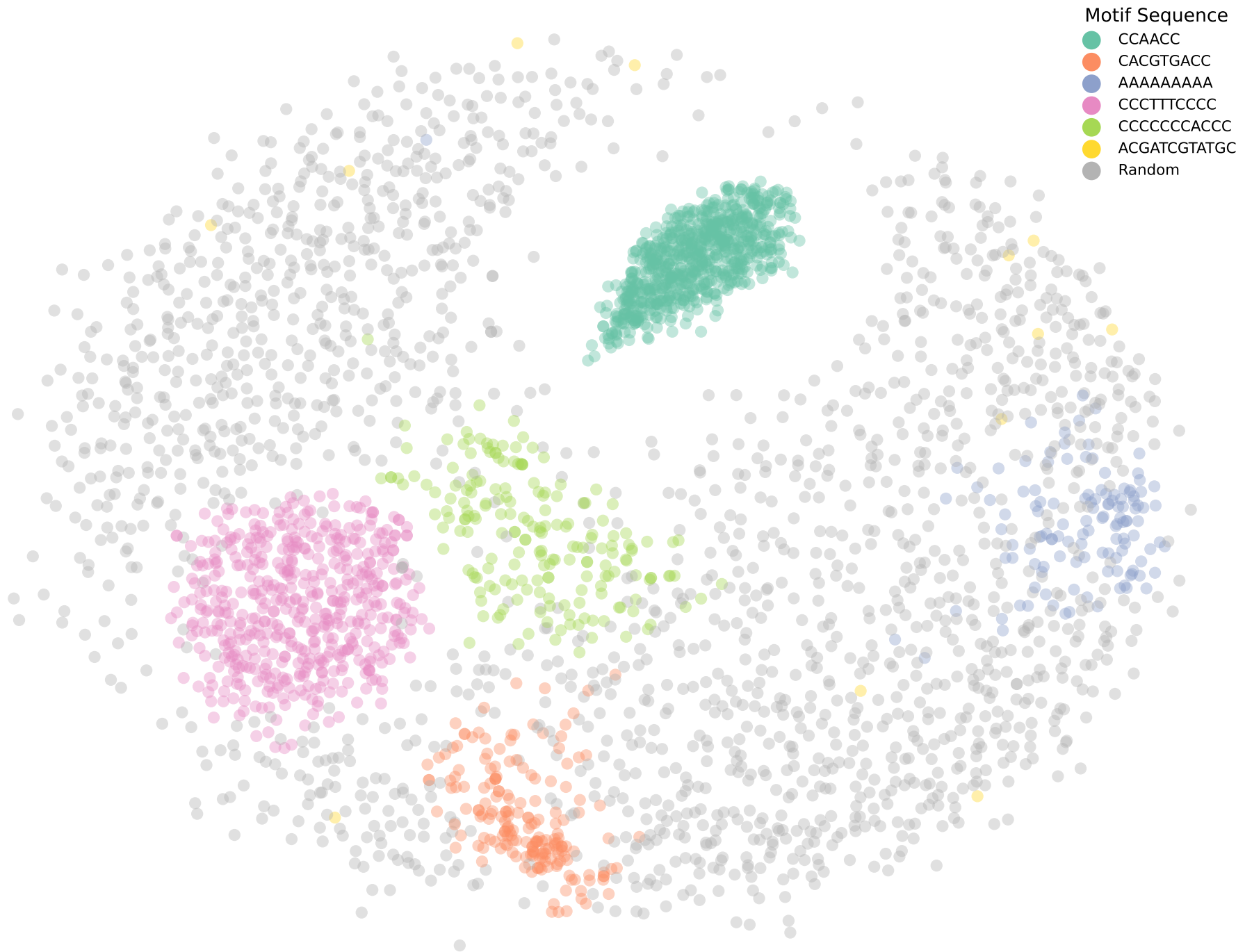

Supplement: Supplement 8 [file Supplemental_Data_1.zip › Supplemental_Data_1/ARNTL_TCAAAA20NCG_W_3/ARNTL_TCAAAA20NCG_W_3_MDS.pdf]

PCA Plot - ARNTL\_TCAAAA20NCG\_W\_3

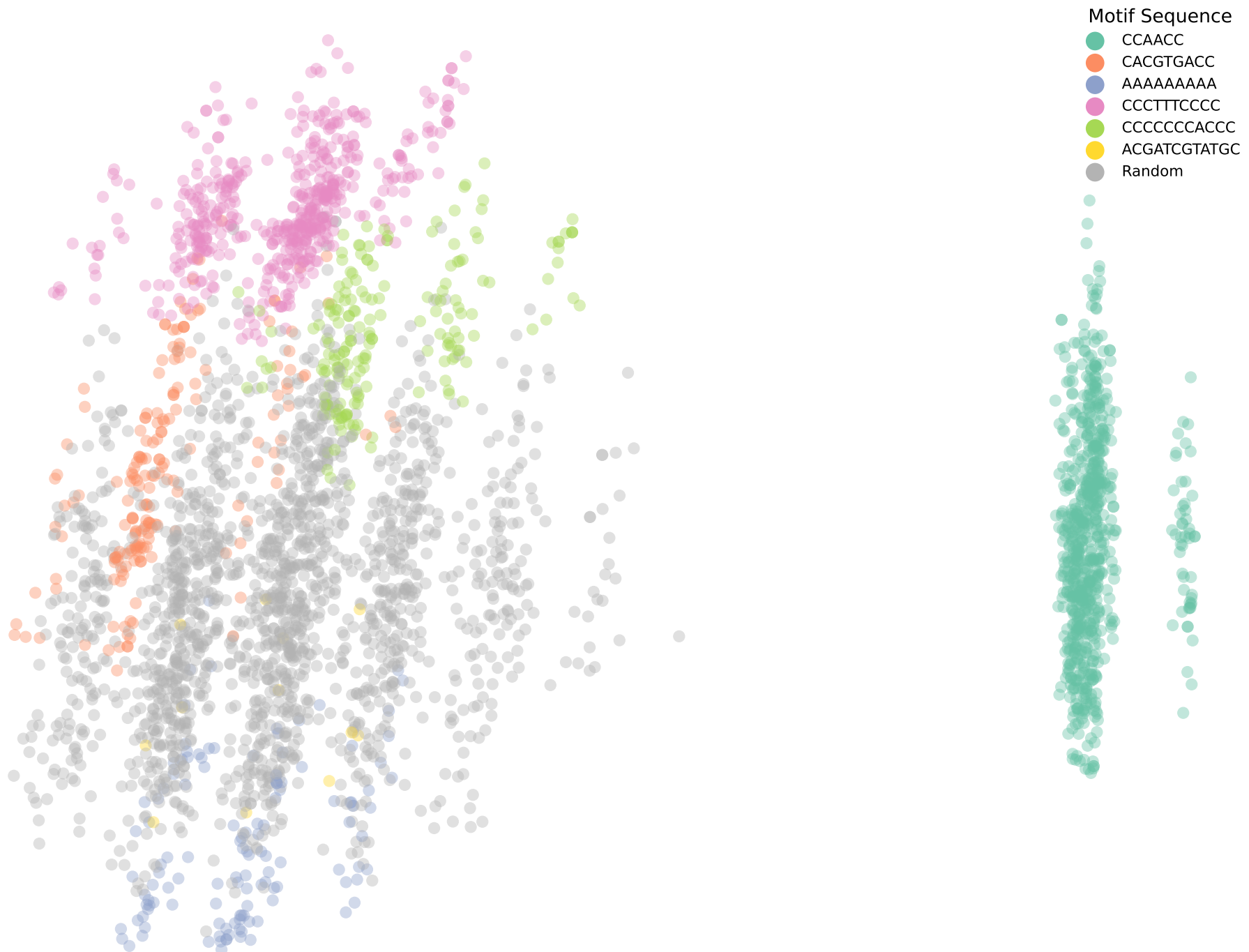

Supplement: Supplement 8 [file Supplemental_Data_1.zip › Supplemental_Data_1/ARNTL_TCAAAA20NCG_W_3/ARNTL_TCAAAA20NCG_W_3_PCA.pdf]

tSNE Plot - ARNTL\_TCAAAA20NCG\_W\_3

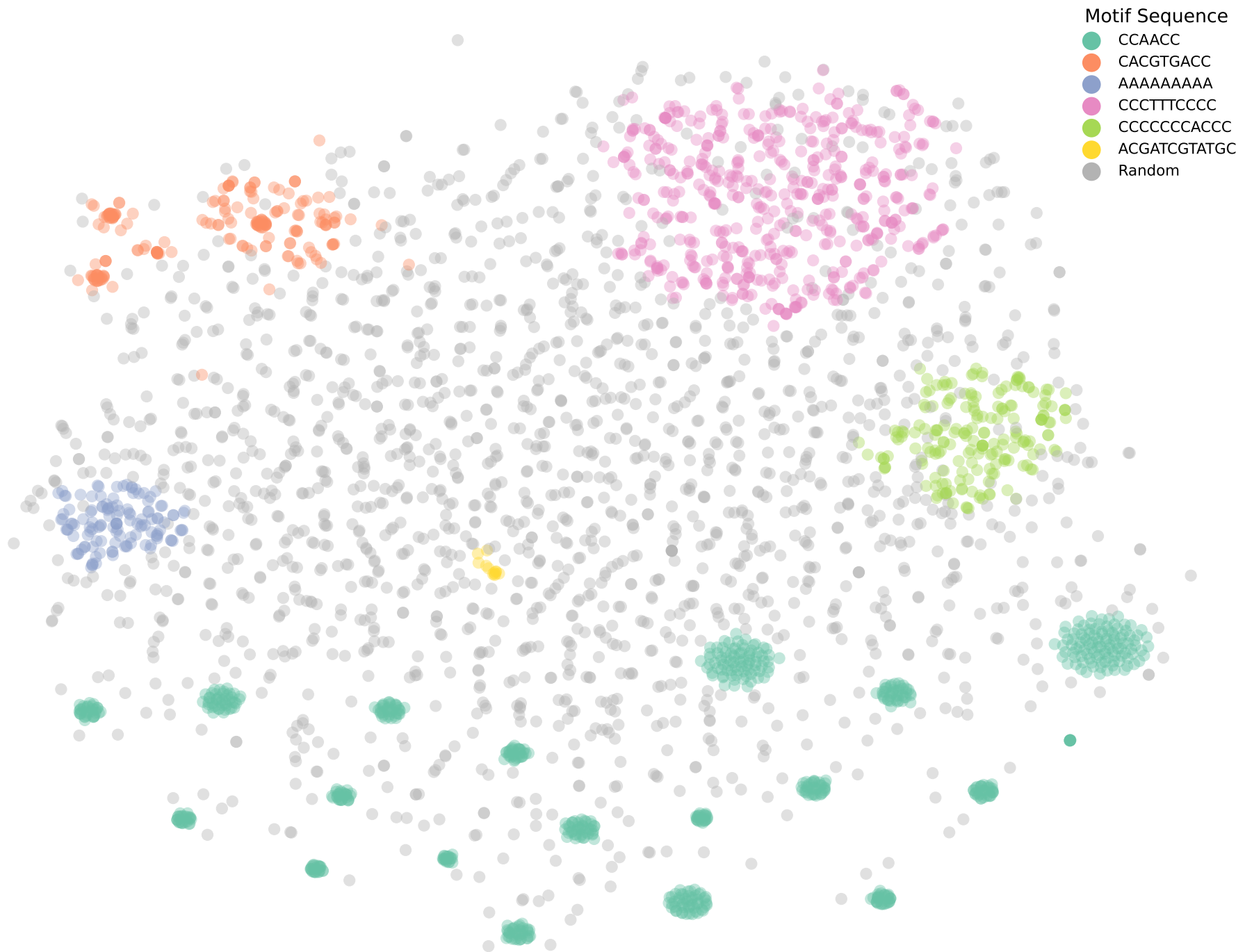

Supplement: Supplement 8 [file Supplemental_Data_1.zip › Supplemental_Data_1/ARNTL_TCAAAA20NCG_W_3/ARNTL_TCAAAA20NCG_W_3_tSNE.pdf]

UMAP Plot - ARNTL\_TCAAAA20NCG\_W\_3

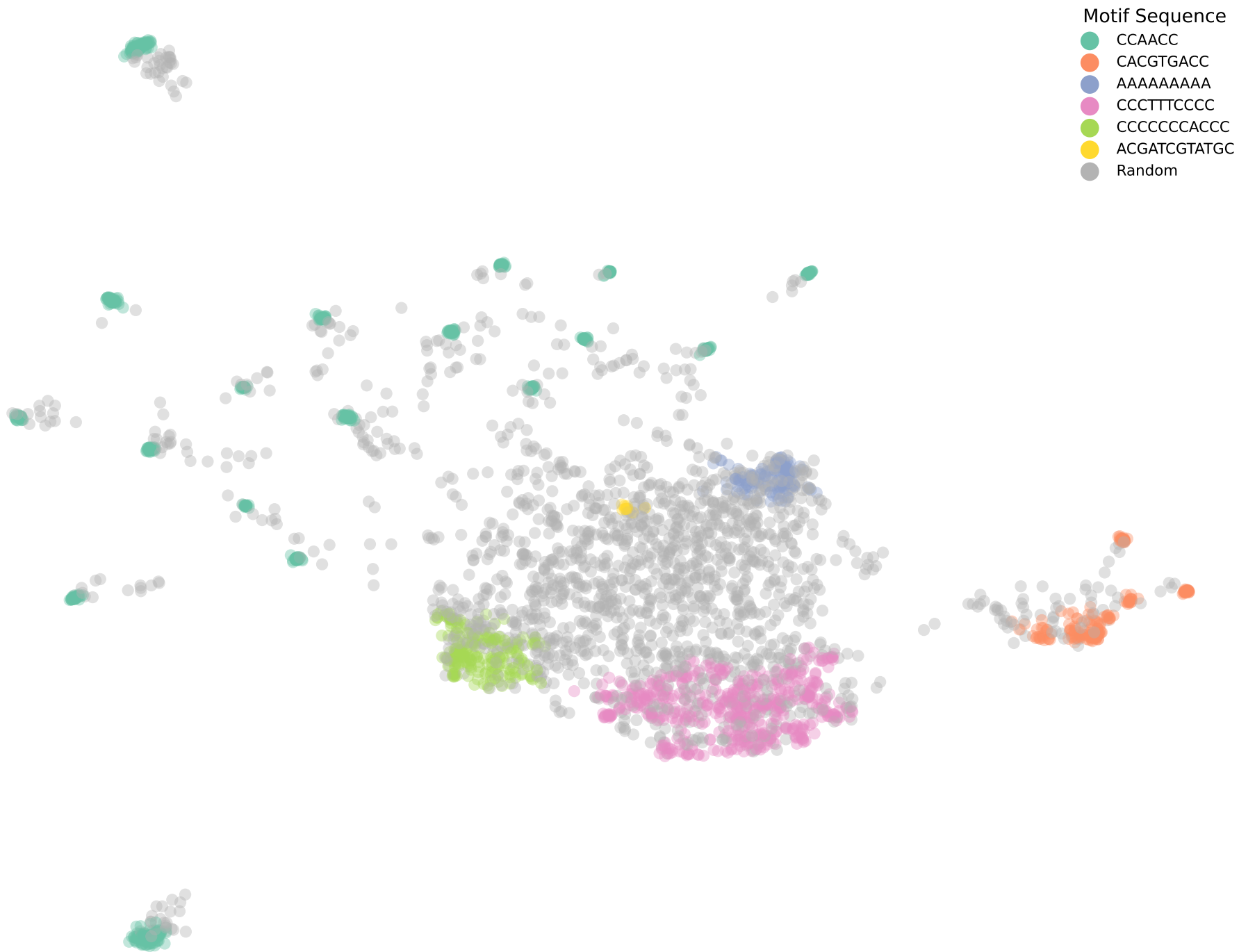

Supplement: Supplement 8 [file Supplemental_Data_1.zip › Supplemental_Data_1/ARNTL_TCAAAA20NCG_W_3/ARNTL_TCAAAA20NCG_W_3_UMAP.pdf]

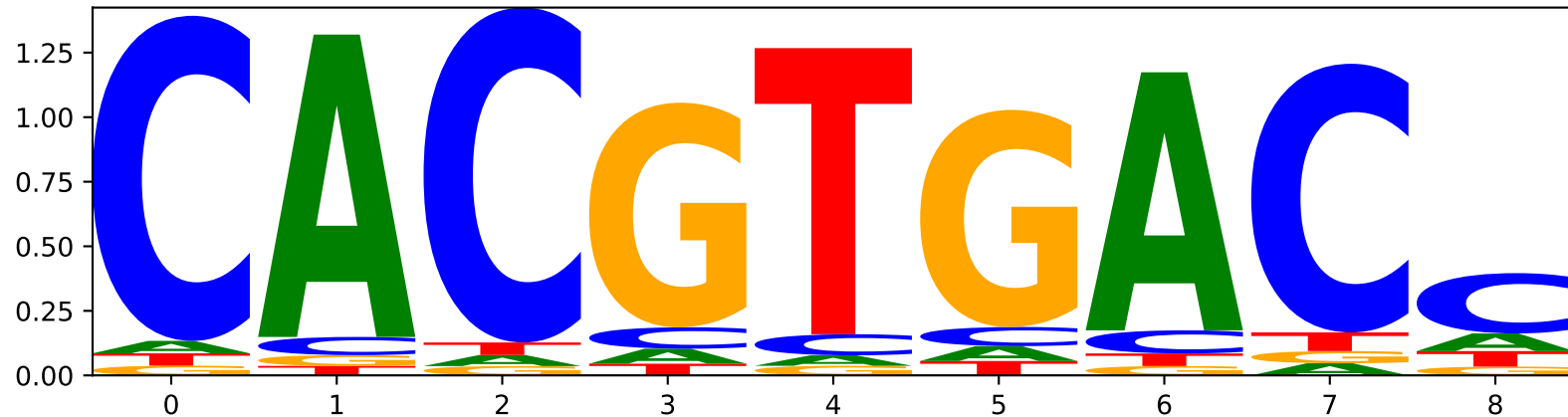

Supplement: Supplement 8 [file Supplemental_Data_1.zip › Supplemental_Data_1/ARNTL_TCAAAA20NCG_W_3/kmap_logo.pdf]

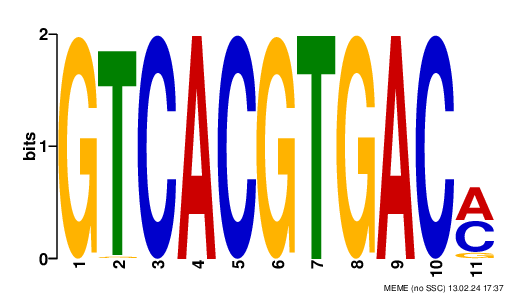

Supplement: Supplement 8 [file Supplemental_Data_1.zip › Supplemental_Data_1/ARNTL_TCAAAA20NCG_W_3/meme_logo.png]

# KMAP LD Plot - ARNTL\_TCAAAA20NCG\_W\_4

Motif Sequence

CACCAC

Random

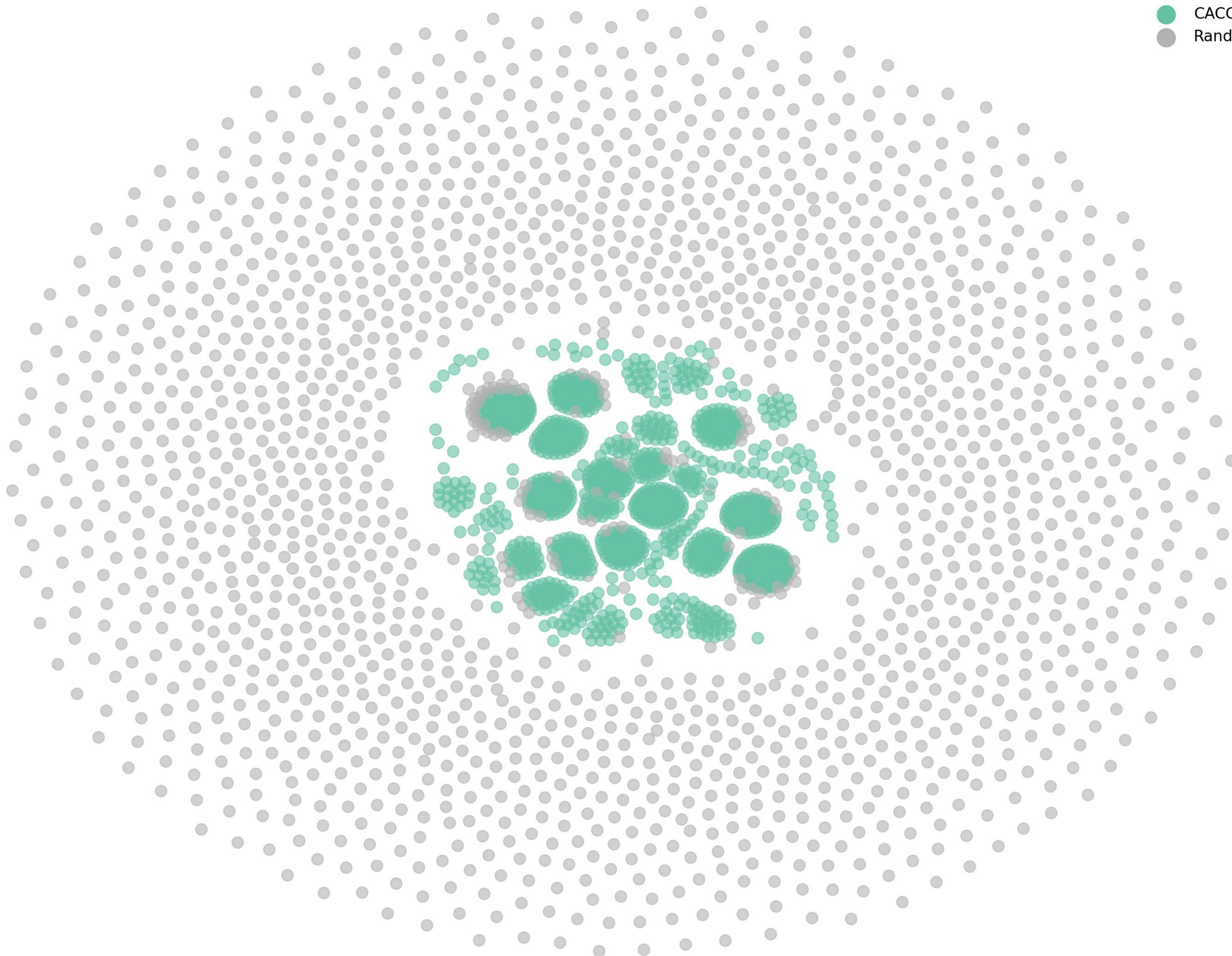

Supplement: Supplement 8 [file Supplemental_Data_1.zip › Supplemental_Data_1/ARNTL_TCAAAA20NCG_W_4/ARNTL_TCAAAA20NCG_W_4_KMAP.pdf]

MDS Plot - ARNTL\_TCAAAA20NCG\_W\_4

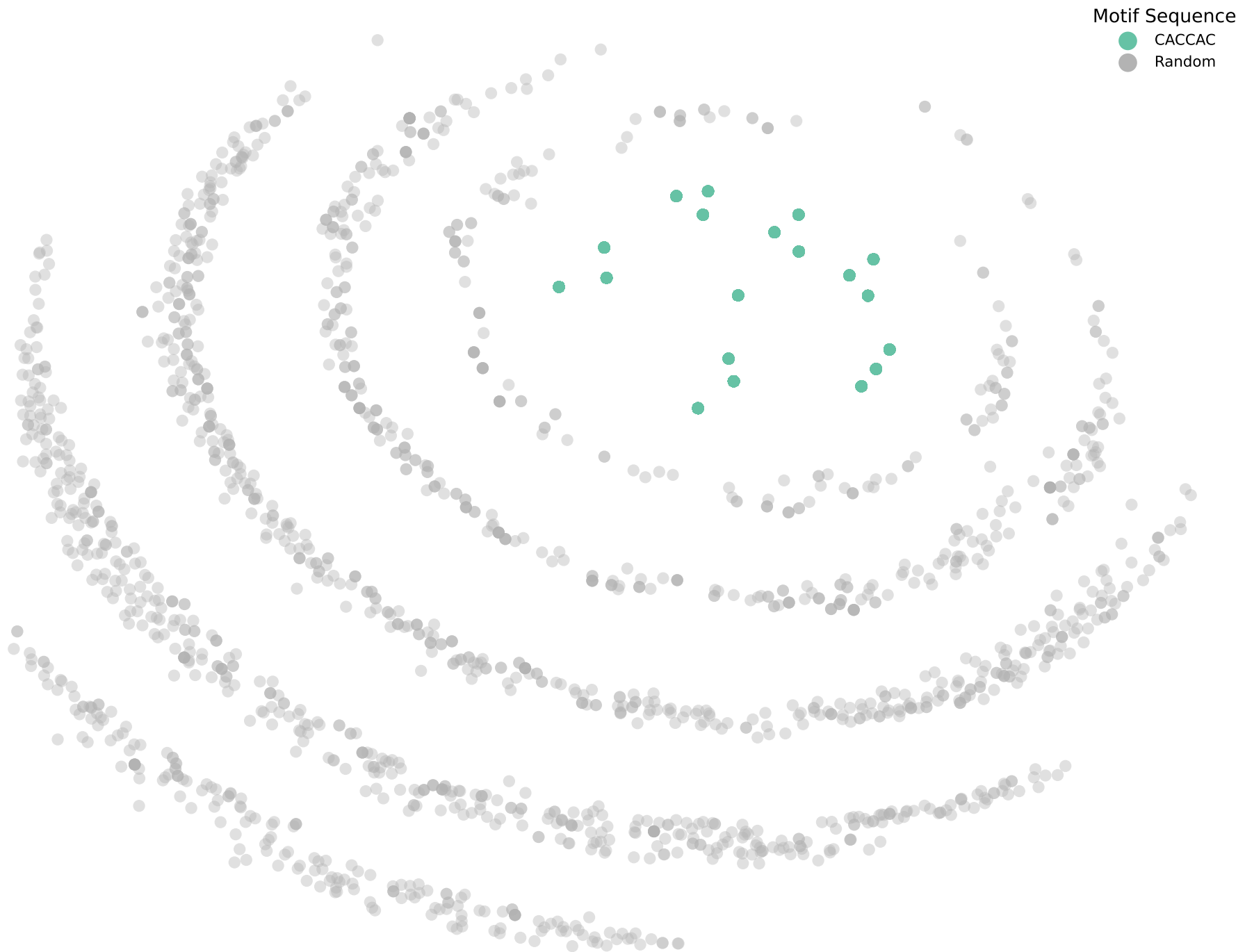

Supplement: Supplement 8 [file Supplemental_Data_1.zip › Supplemental_Data_1/ARNTL_TCAAAA20NCG_W_4/ARNTL_TCAAAA20NCG_W_4_MDS.pdf]

PCA Plot - ARNTL\_TCAAAA20NCG\_W\_4

Motif Sequence

CACCAC

Random

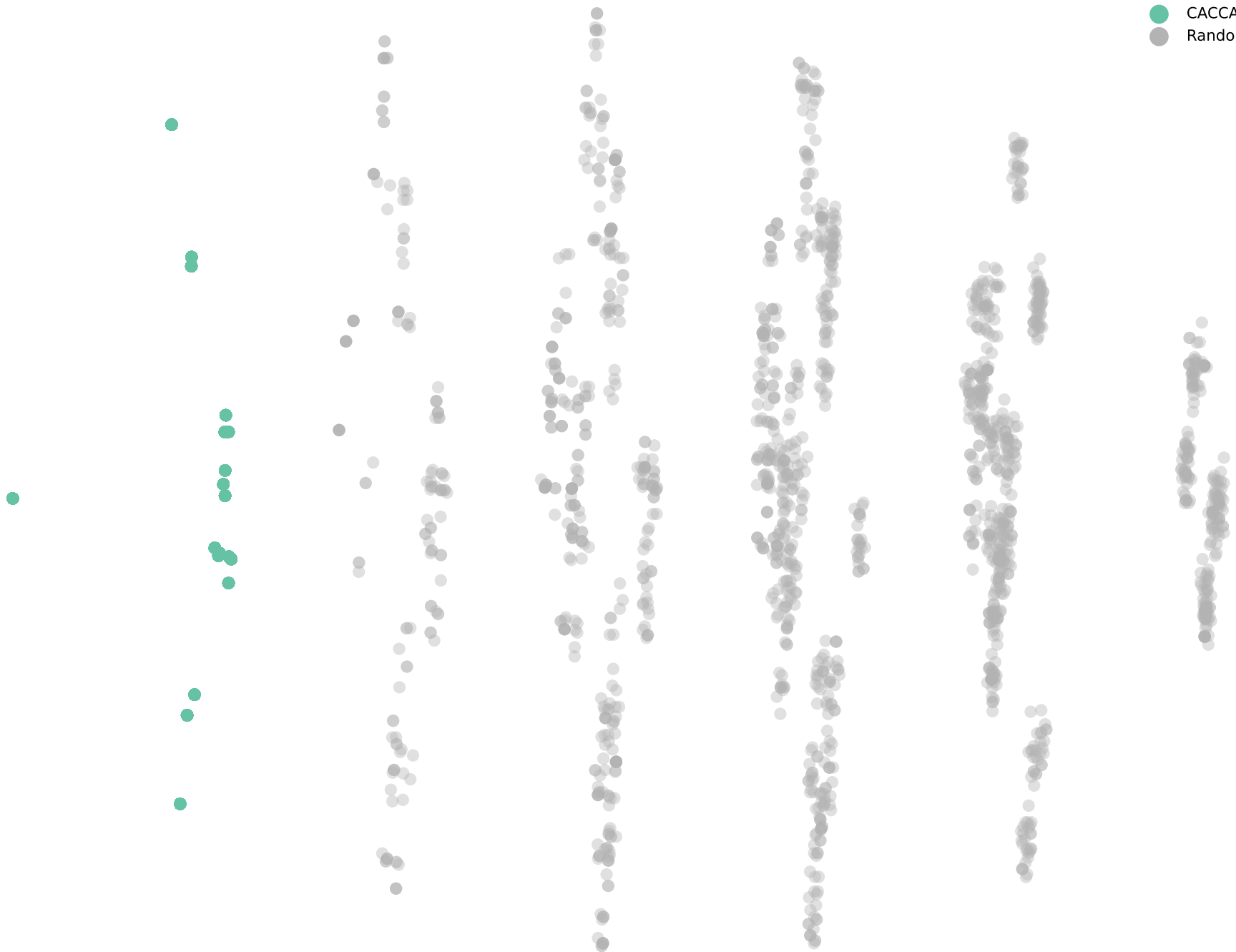

Supplement: Supplement 8 [file Supplemental_Data_1.zip › Supplemental_Data_1/ARNTL_TCAAAA20NCG_W_4/ARNTL_TCAAAA20NCG_W_4_PCA.pdf]

tSNE Plot - ARNTL\_TCAAAA20NCG\_W\_4

Motif Sequence

CACCAC

Random

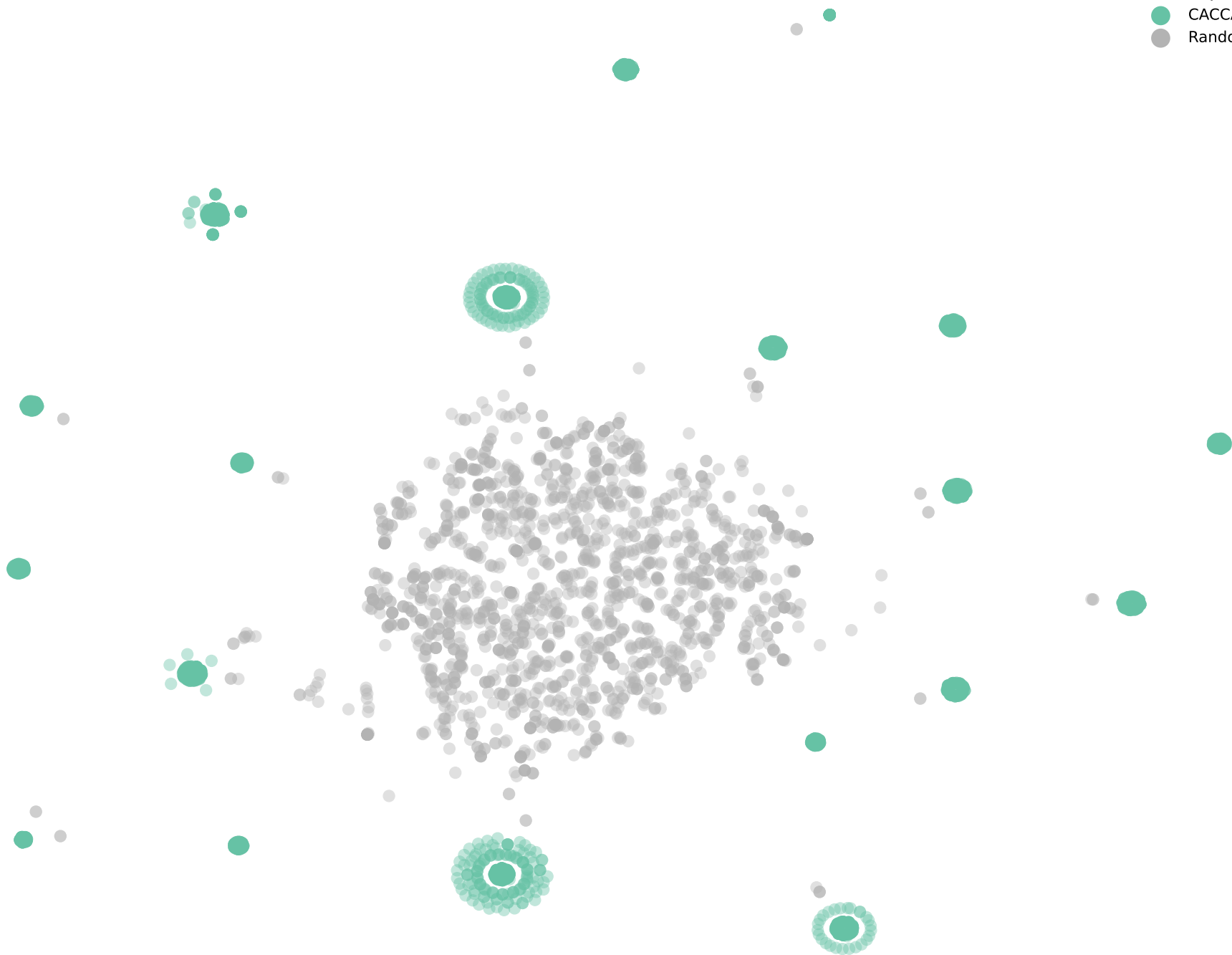

Supplement: Supplement 8 [file Supplemental_Data_1.zip › Supplemental_Data_1/ARNTL_TCAAAA20NCG_W_4/ARNTL_TCAAAA20NCG_W_4_tSNE.pdf]

UMAP Plot - ARNTL\_TCAAAA20NCG\_W\_4

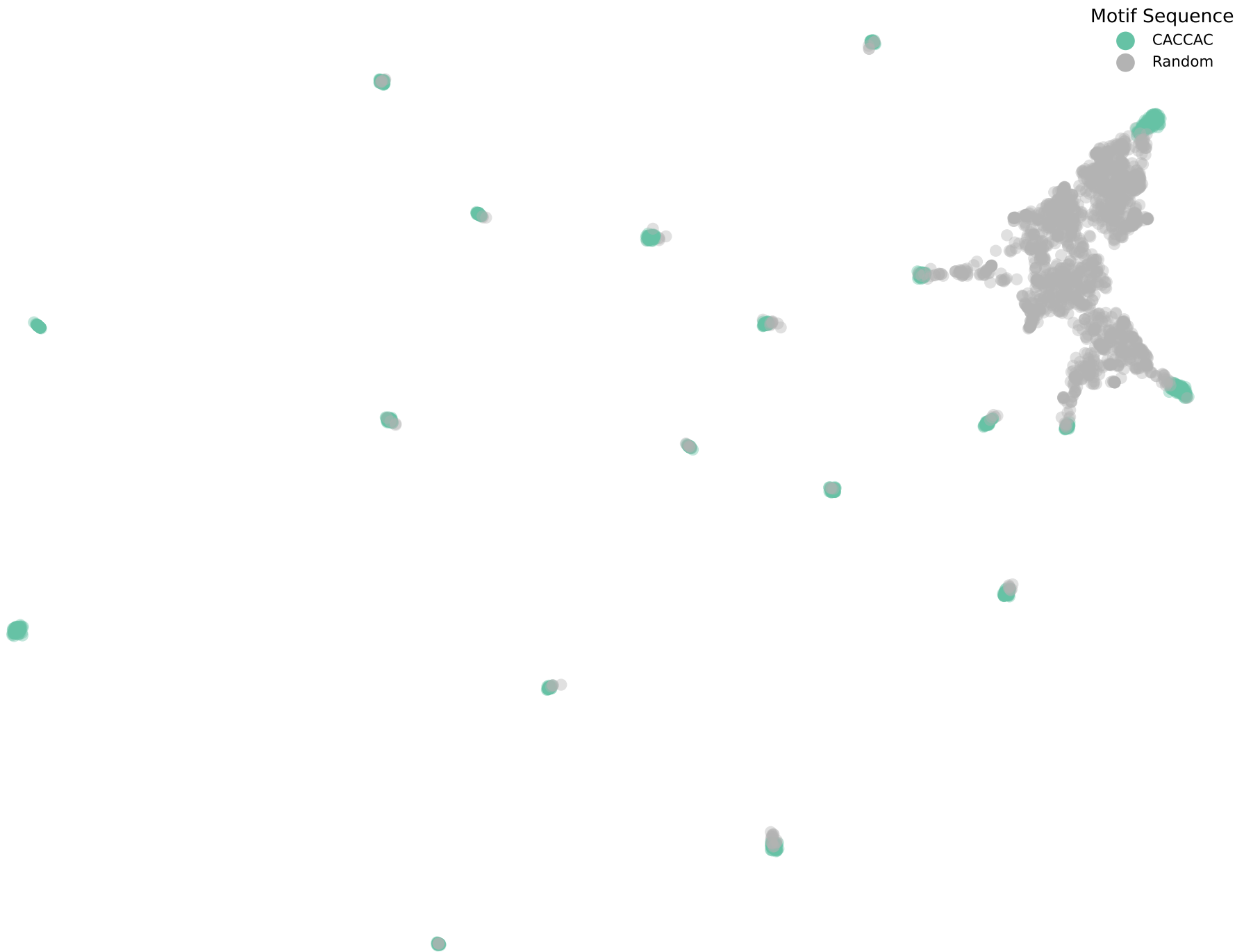

Supplement: Supplement 8 [file Supplemental_Data_1.zip › Supplemental_Data_1/ARNTL_TCAAAA20NCG_W_4/ARNTL_TCAAAA20NCG_W_4_UMAP.pdf]

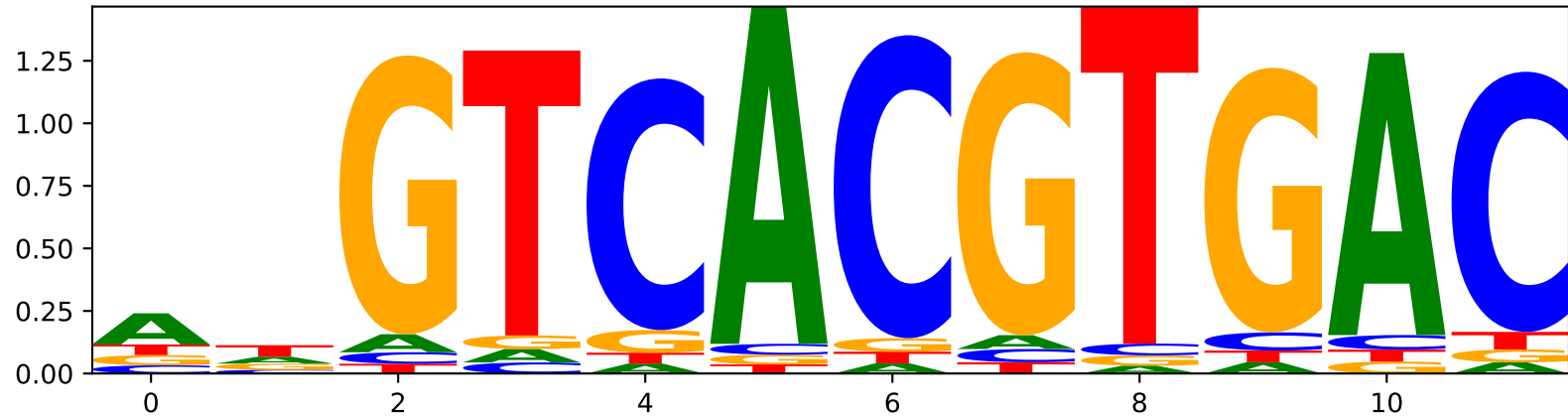

Supplement: Supplement 8 [file Supplemental_Data_1.zip › Supplemental_Data_1/ARNTL_TCAAAA20NCG_W_4/kmap_logo.pdf]

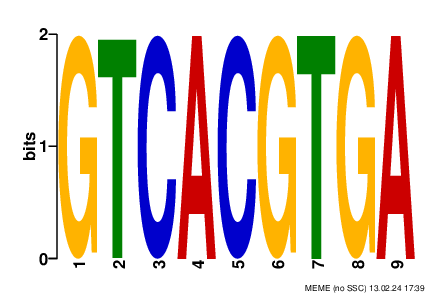

Supplement: Supplement 8 [file Supplemental_Data_1.zip › Supplemental_Data_1/ARNTL_TCAAAA20NCG_W_4/meme_logo.png]

KMAP LD Plot - Arx\_TCGCAT20NACT\_AC\_3

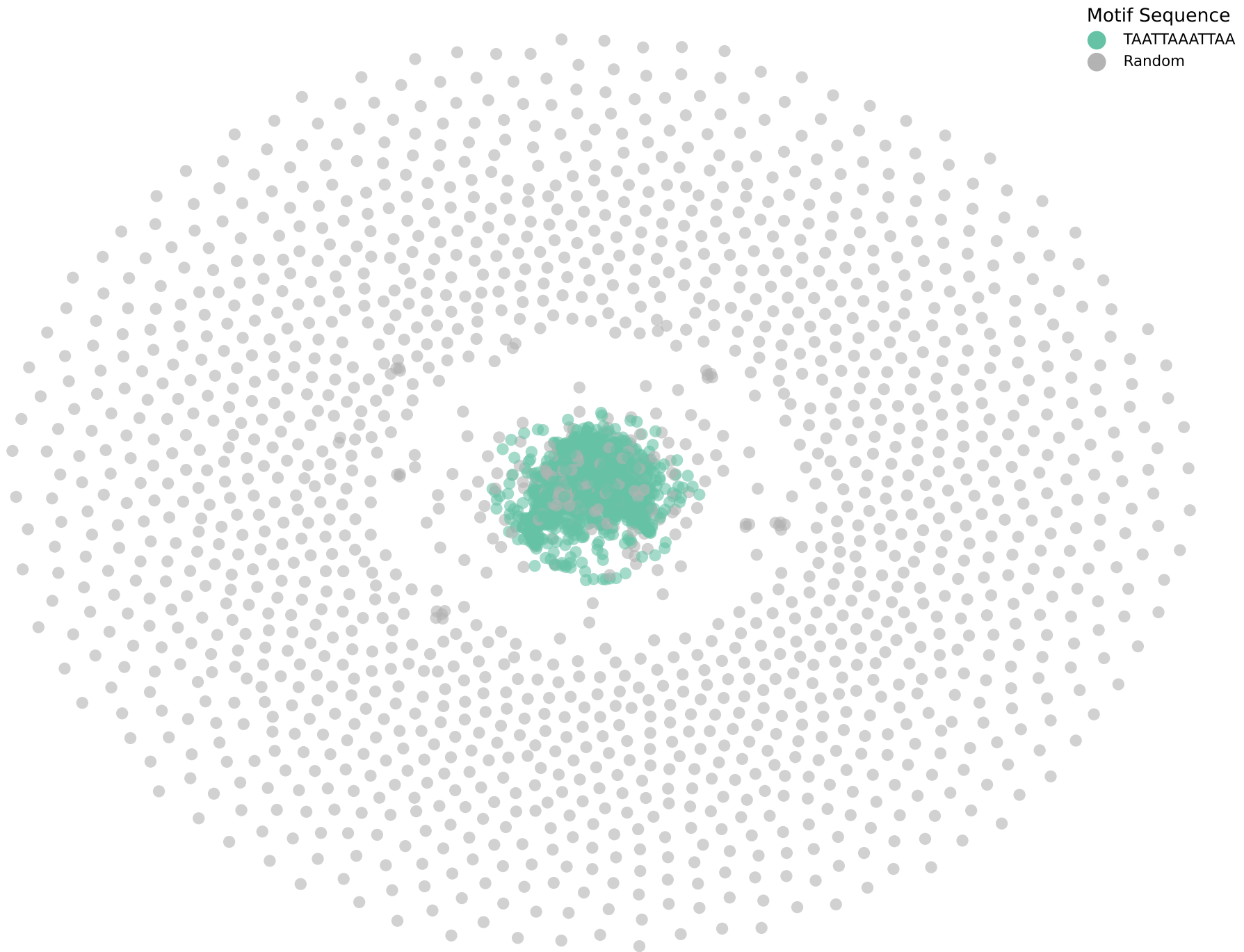

Supplement: Supplement 8 [file Supplemental_Data_1.zip › Supplemental_Data_1/Arx_TCGCAT20NACT_AC_3/Arx_TCGCAT20NACT_AC_3_KMAP.pdf]

MDS Plot - Arx\_TCGCAT20NACT\_AC\_3

Motif Sequence

- TAATTAAATTAA
- Random

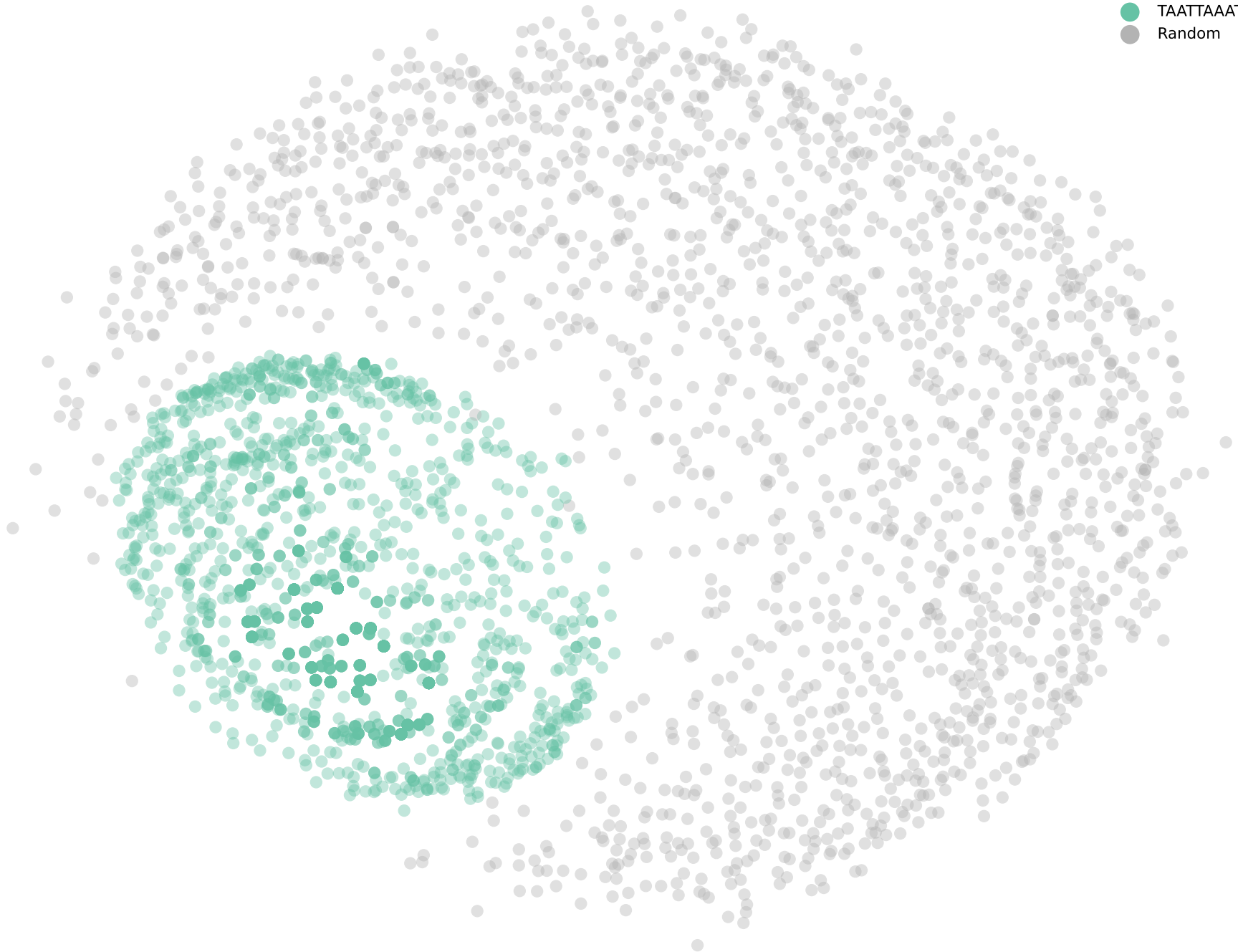

Supplement: Supplement 8 [file Supplemental_Data_1.zip › Supplemental_Data_1/Arx_TCGCAT20NACT_AC_3/Arx_TCGCAT20NACT_AC_3_MDS.pdf]

PCA Plot - Arx\_TCGCAT20NACT\_AC\_3

Motif Sequence

TAATTAAATTAA

Random

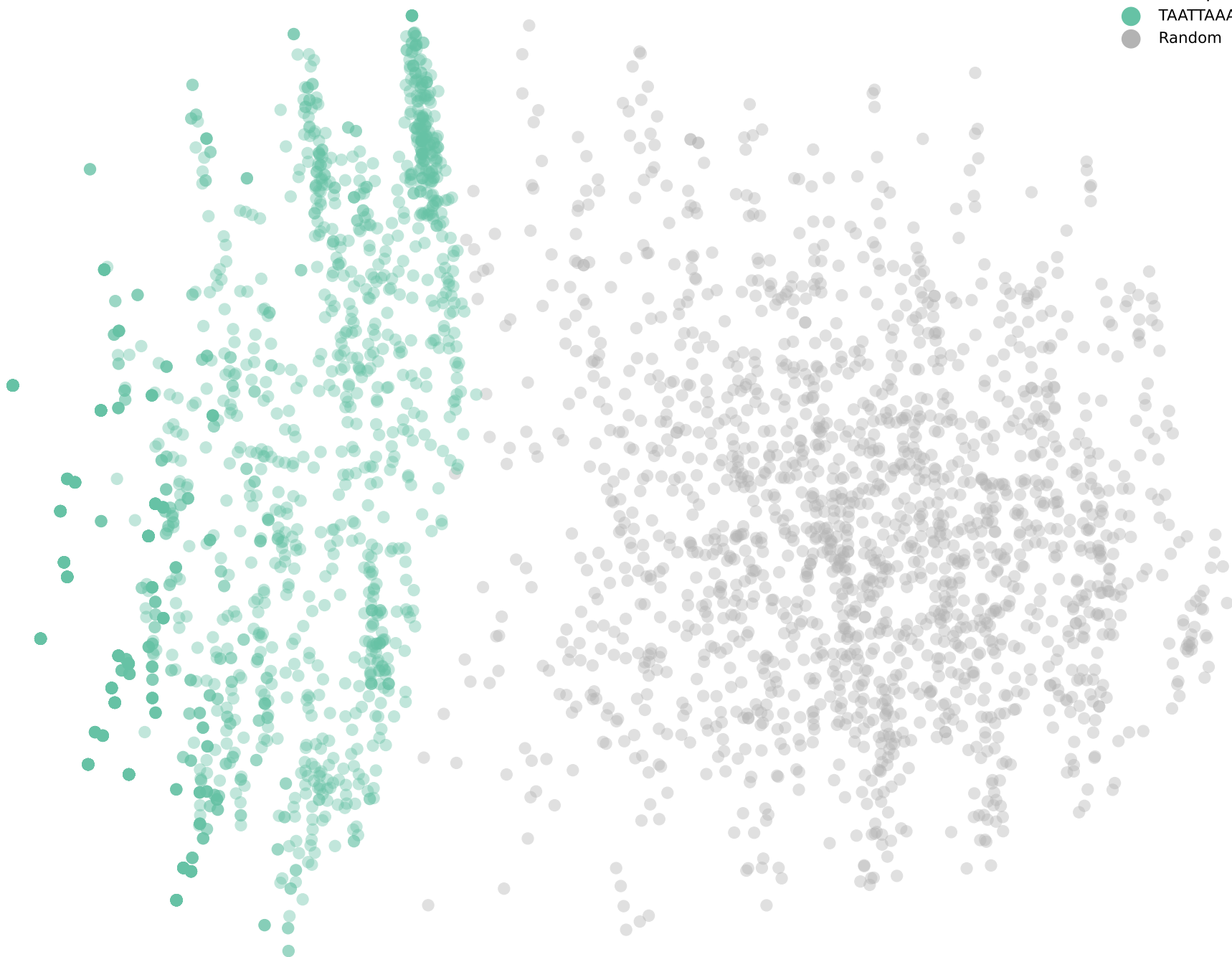

Supplement: Supplement 8 [file Supplemental_Data_1.zip › Supplemental_Data_1/Arx_TCGCAT20NACT_AC_3/Arx_TCGCAT20NACT_AC_3_PCA.pdf]

tSNE Plot - Arx\_TCGCAT20NACT\_AC\_3

Motif Sequence

- TAATTAAATTAA
- Random

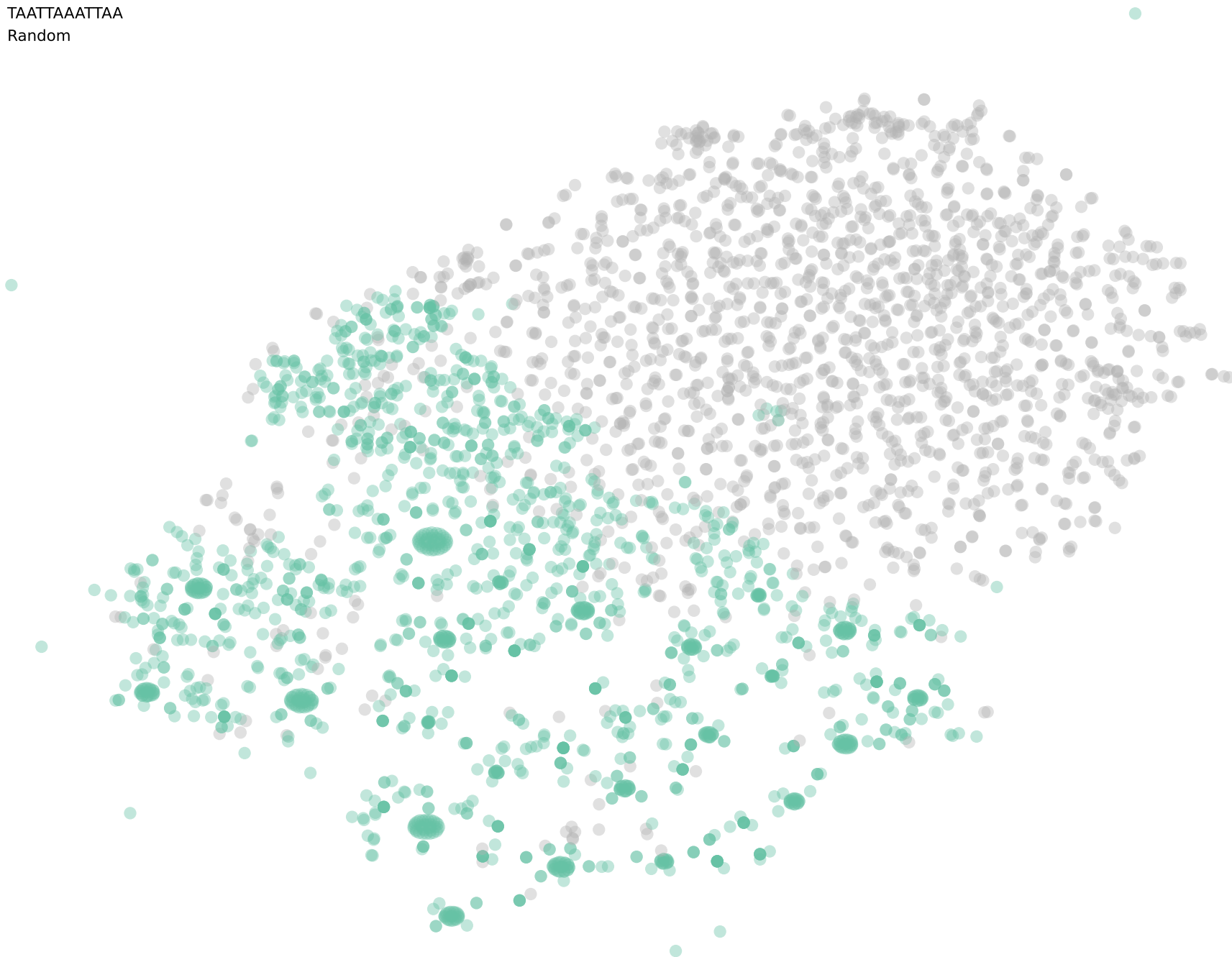

Supplement: Supplement 8 [file Supplemental_Data_1.zip › Supplemental_Data_1/Arx_TCGCAT20NACT_AC_3/Arx_TCGCAT20NACT_AC_3_tSNE.pdf]

UMAP Plot - Arx\_TCGCAT20NACT\_AC\_3

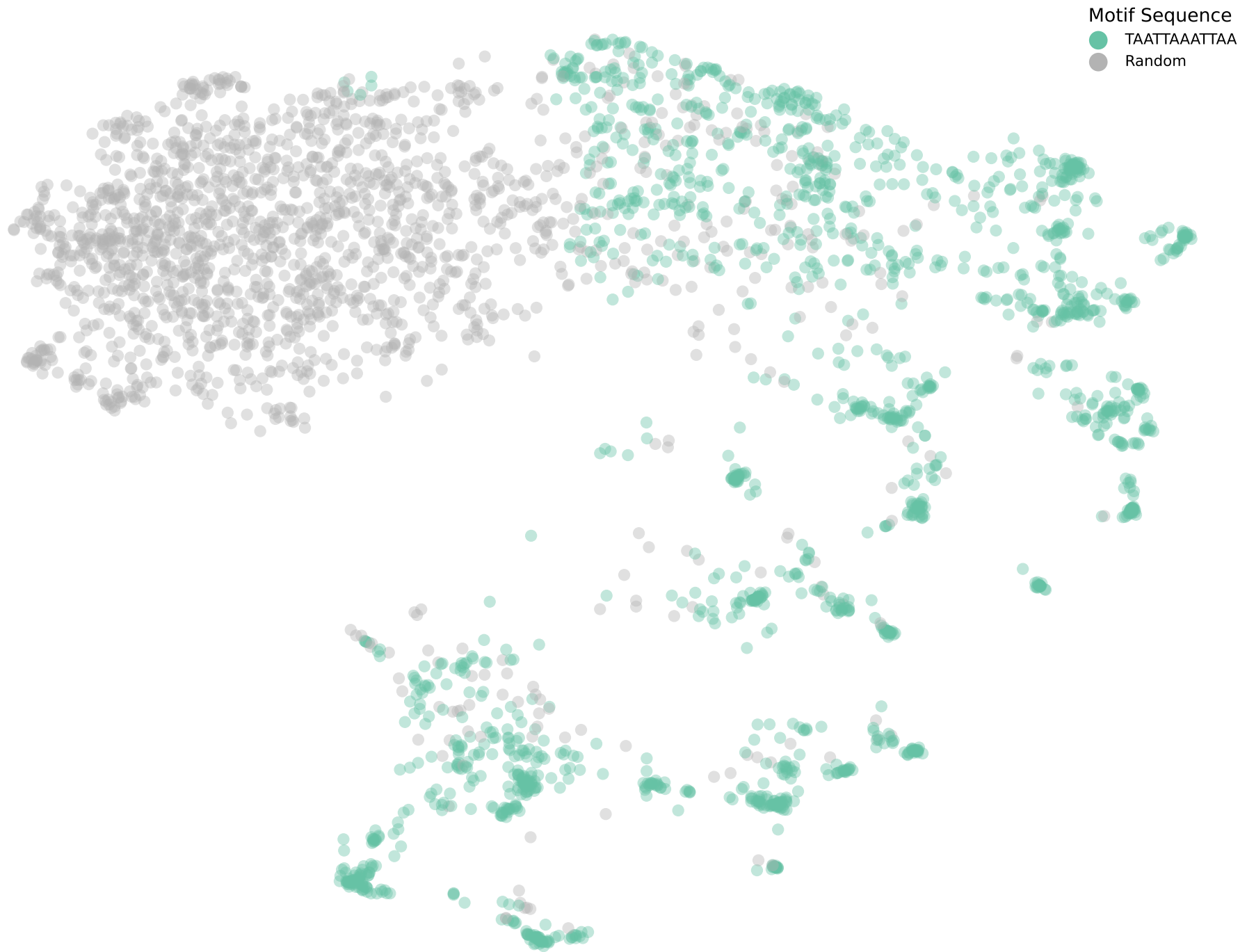

Supplement: Supplement 8 [file Supplemental_Data_1.zip › Supplemental_Data_1/Arx_TCGCAT20NACT_AC_3/Arx_TCGCAT20NACT_AC_3_UMAP.pdf]

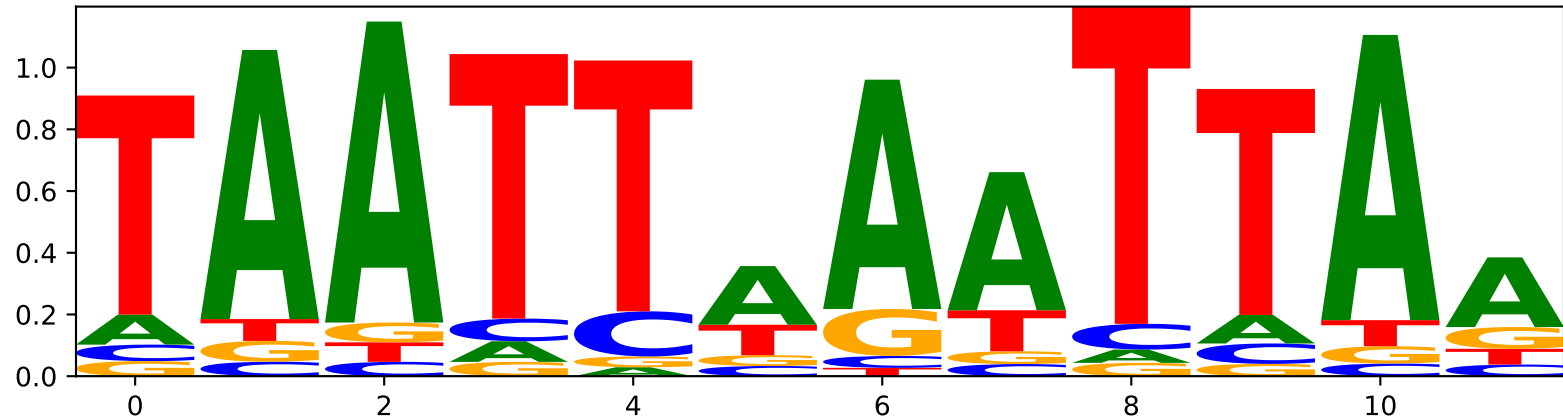

Supplement: Supplement 8 [file Supplemental_Data_1.zip › Supplemental_Data_1/Arx_TCGCAT20NACT_AC_3/kmap_logo.pdf]

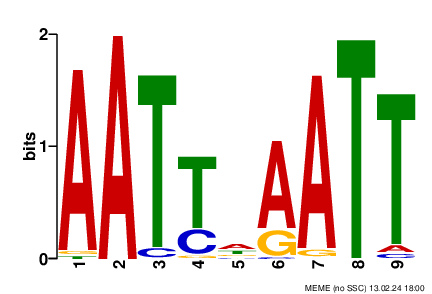

Supplement: Supplement 8 [file Supplemental_Data_1.zip › Supplemental_Data_1/Arx_TCGCAT20NACT_AC_3/meme_logo.png]

KMAP LD Plot - Arx\_TCGCAT20NACT\_AC\_4

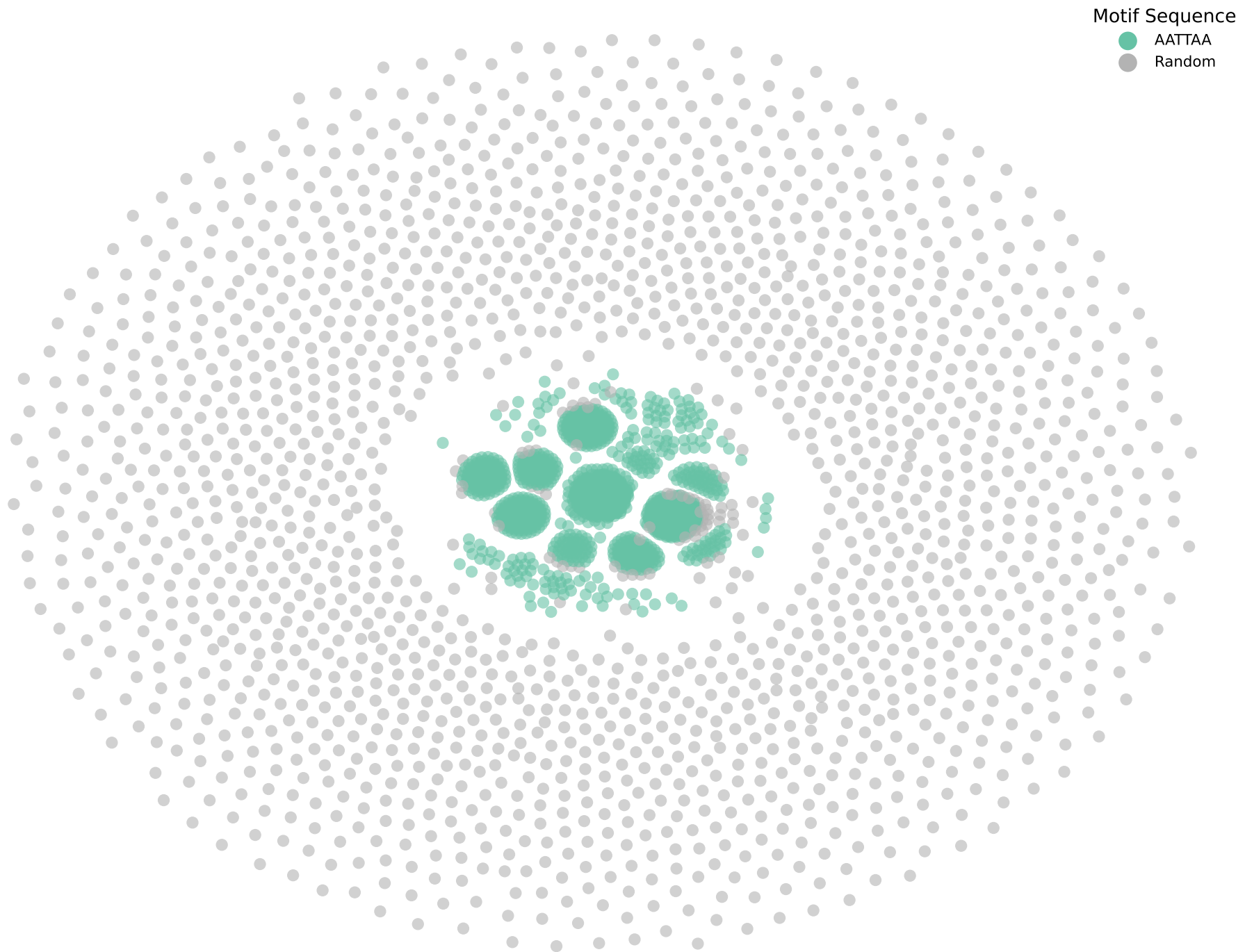

Supplement: Supplement 8 [file Supplemental_Data_1.zip › Supplemental_Data_1/Arx_TCGCAT20NACT_AC_4/Arx_TCGCAT20NACT_AC_4_KMAP.pdf]
