# Supplementary figures and images for: k-mer manifold approximation and projection for visualizing DNA sequences (part 2 of 2)
Source: Genome Res. 2025 May;35(5):1234–46. doi: 10.1101/gr.279458.124 (PMC12047656; doi:10.1101/gr.279458.124)

MDS Plot - Arx\_TCGCAT20NACT\_AC\_4

Motif Sequence

AATTAA

Random

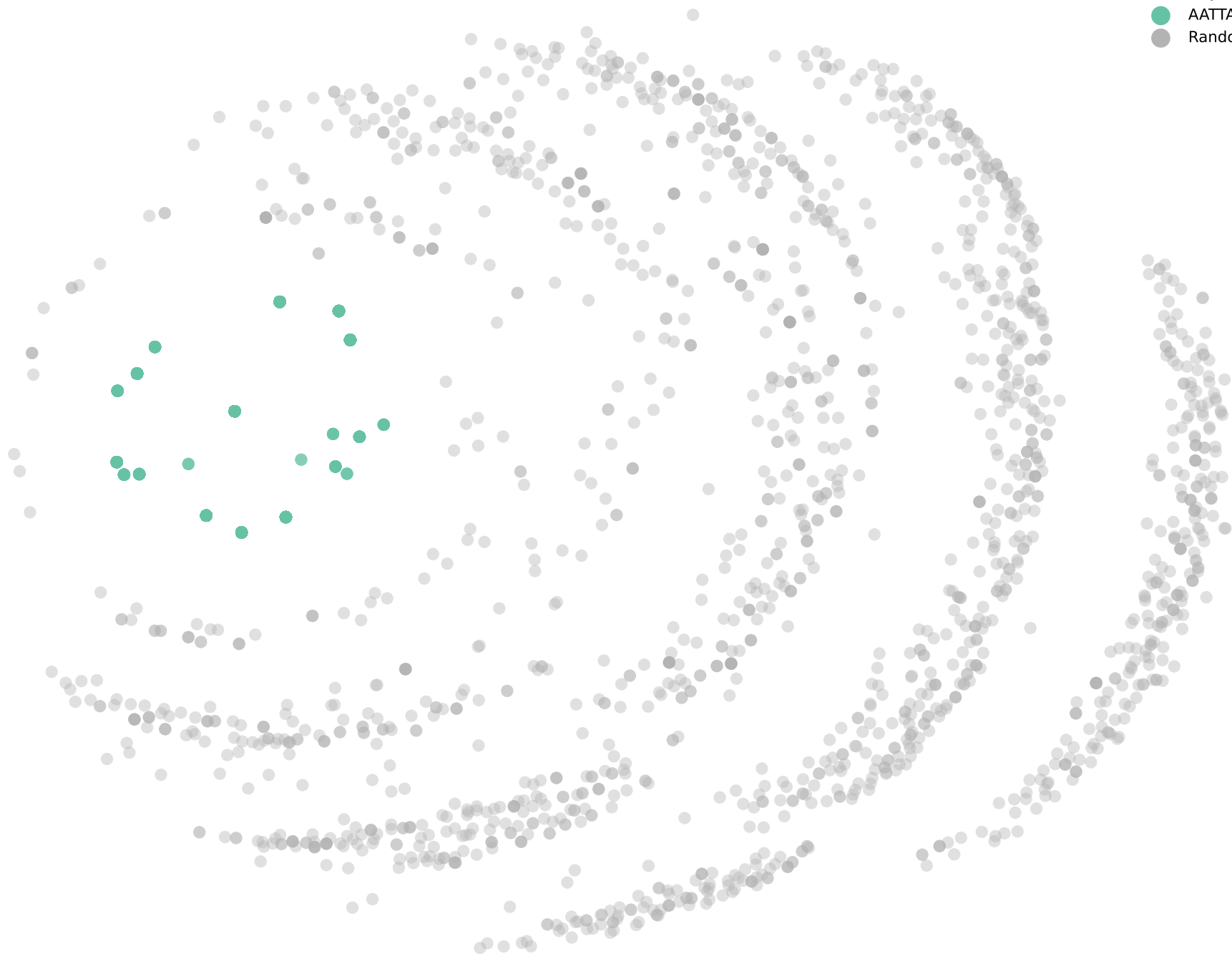

Supplement: Supplement 8 [file Supplemental_Data_1.zip › Supplemental_Data_1/Arx_TCGCAT20NACT_AC_4/Arx_TCGCAT20NACT_AC_4_MDS.pdf]

PCA Plot - Arx\_TCGCAT20NACT\_AC\_4

Motif Sequence

AATTAA

Random

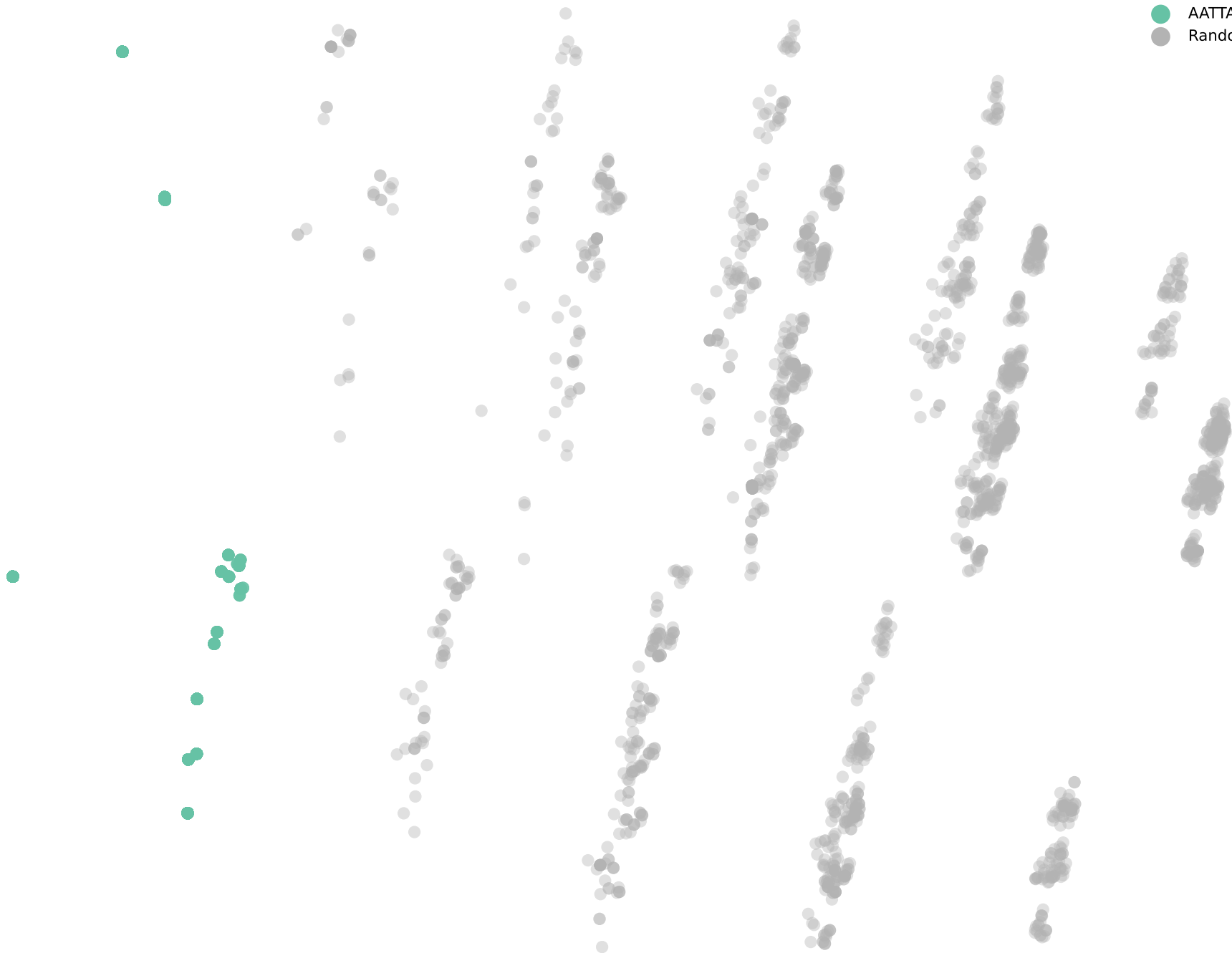

Supplement: Supplement 8 [file Supplemental_Data_1.zip › Supplemental_Data_1/Arx_TCGCAT20NACT_AC_4/Arx_TCGCAT20NACT_AC_4_PCA.pdf]

tSNE Plot - Arx\_TCGCAT20NACT\_AC\_4

Motif Sequence

AATTAA

Random

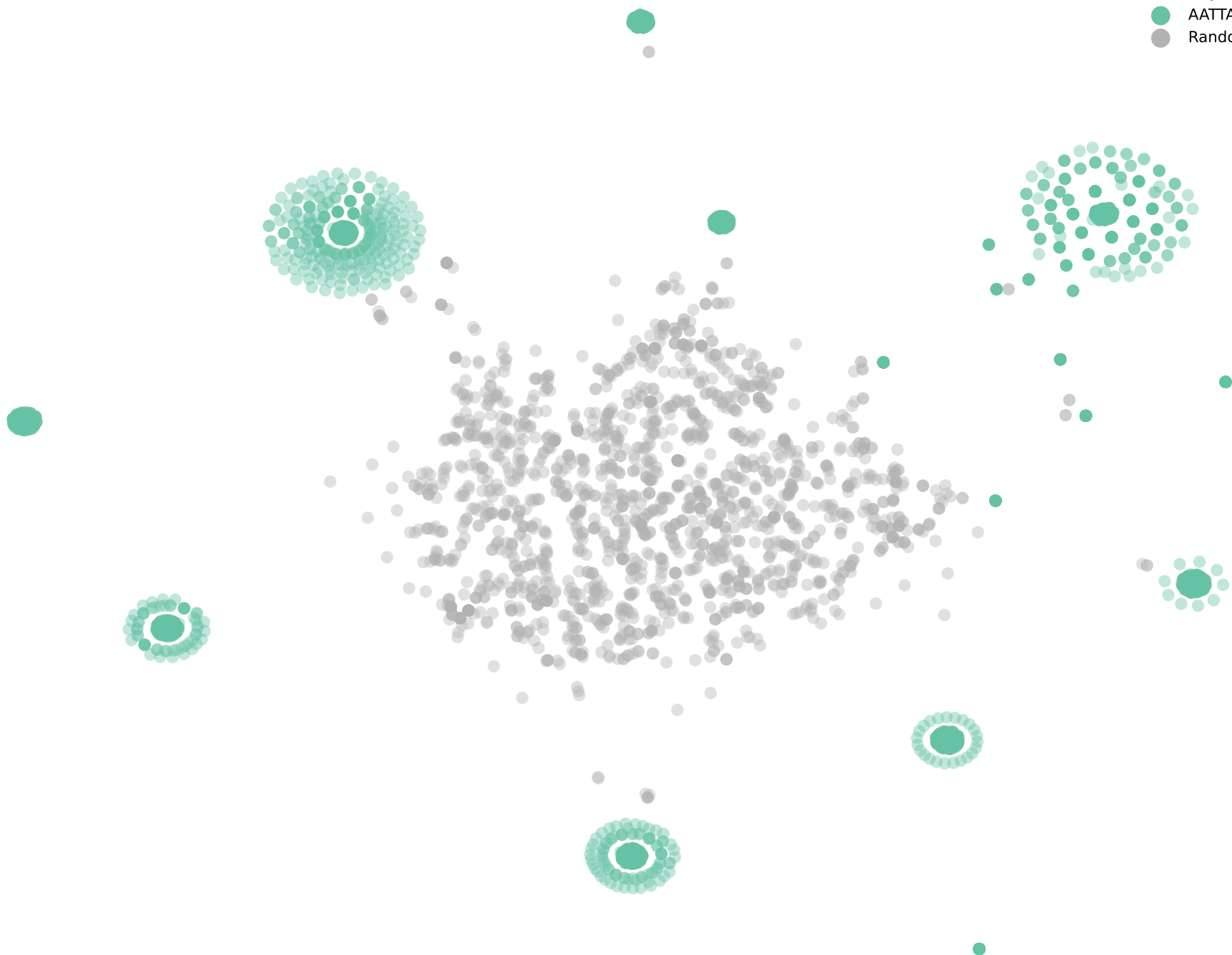

Supplement: Supplement 8 [file Supplemental_Data_1.zip › Supplemental_Data_1/Arx_TCGCAT20NACT_AC_4/Arx_TCGCAT20NACT_AC_4_tSNE.pdf]

UMAP Plot - Arx\_TCGCAT20NACT\_AC\_4

Motif Sequence

AATTAA

Random

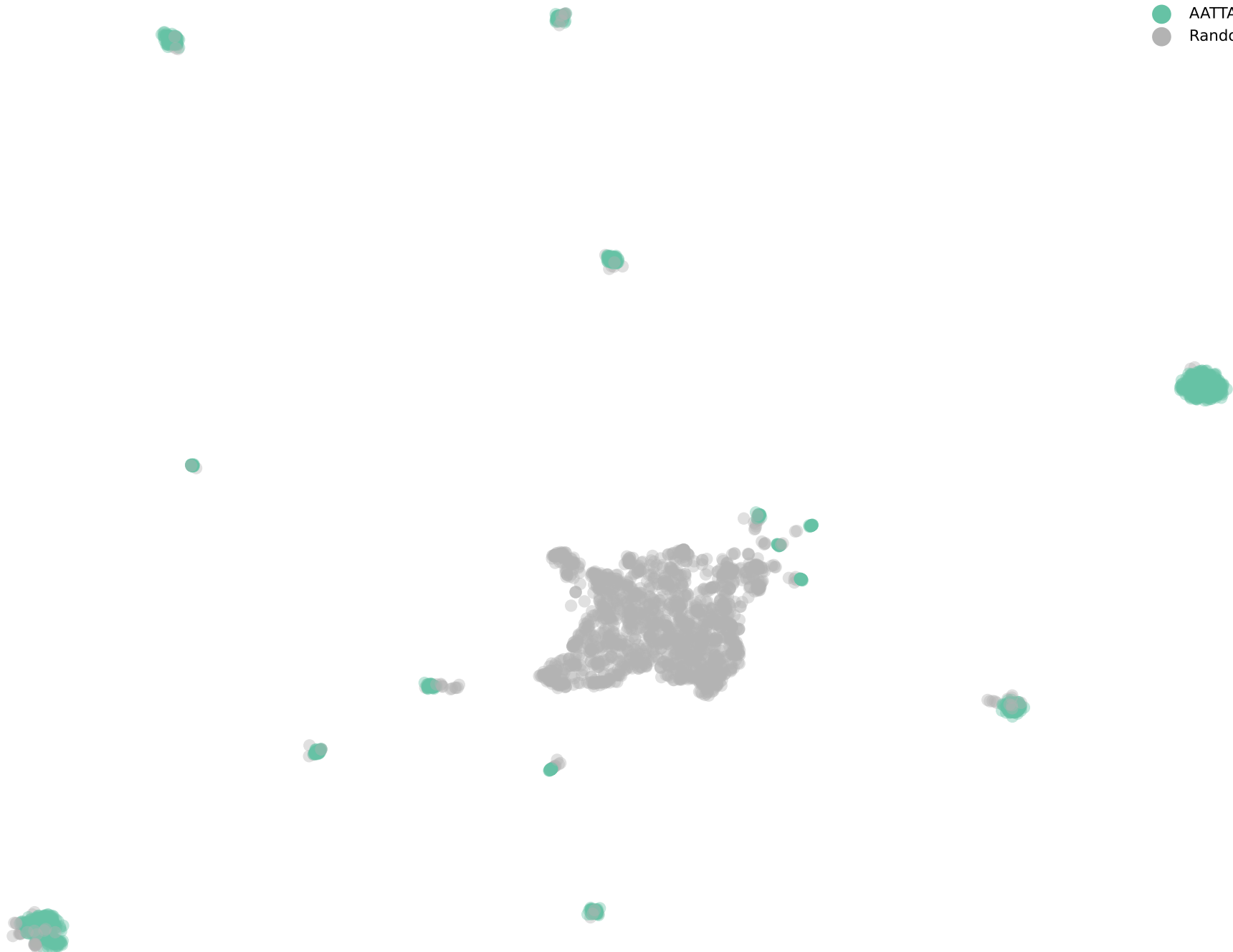

Supplement: Supplement 8 [file Supplemental_Data_1.zip › Supplemental_Data_1/Arx_TCGCAT20NACT_AC_4/Arx_TCGCAT20NACT_AC_4_UMAP.pdf]

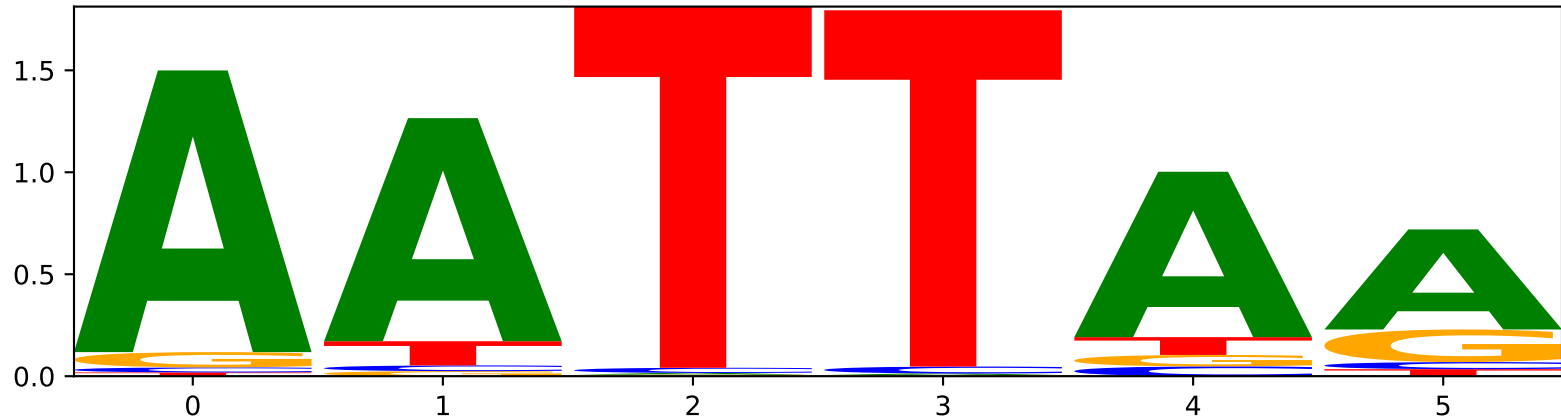

Supplement: Supplement 8 [file Supplemental_Data_1.zip › Supplemental_Data_1/Arx_TCGCAT20NACT_AC_4/kmap_logo.pdf]

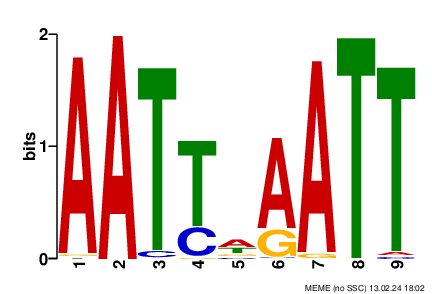

Supplement: Supplement 8 [file Supplemental_Data_1.zip › Supplemental_Data_1/Arx_TCGCAT20NACT_AC_4/meme_logo.png]

KMAP LD Plot - ARX\_TGCGTT20NTGC\_Z\_3

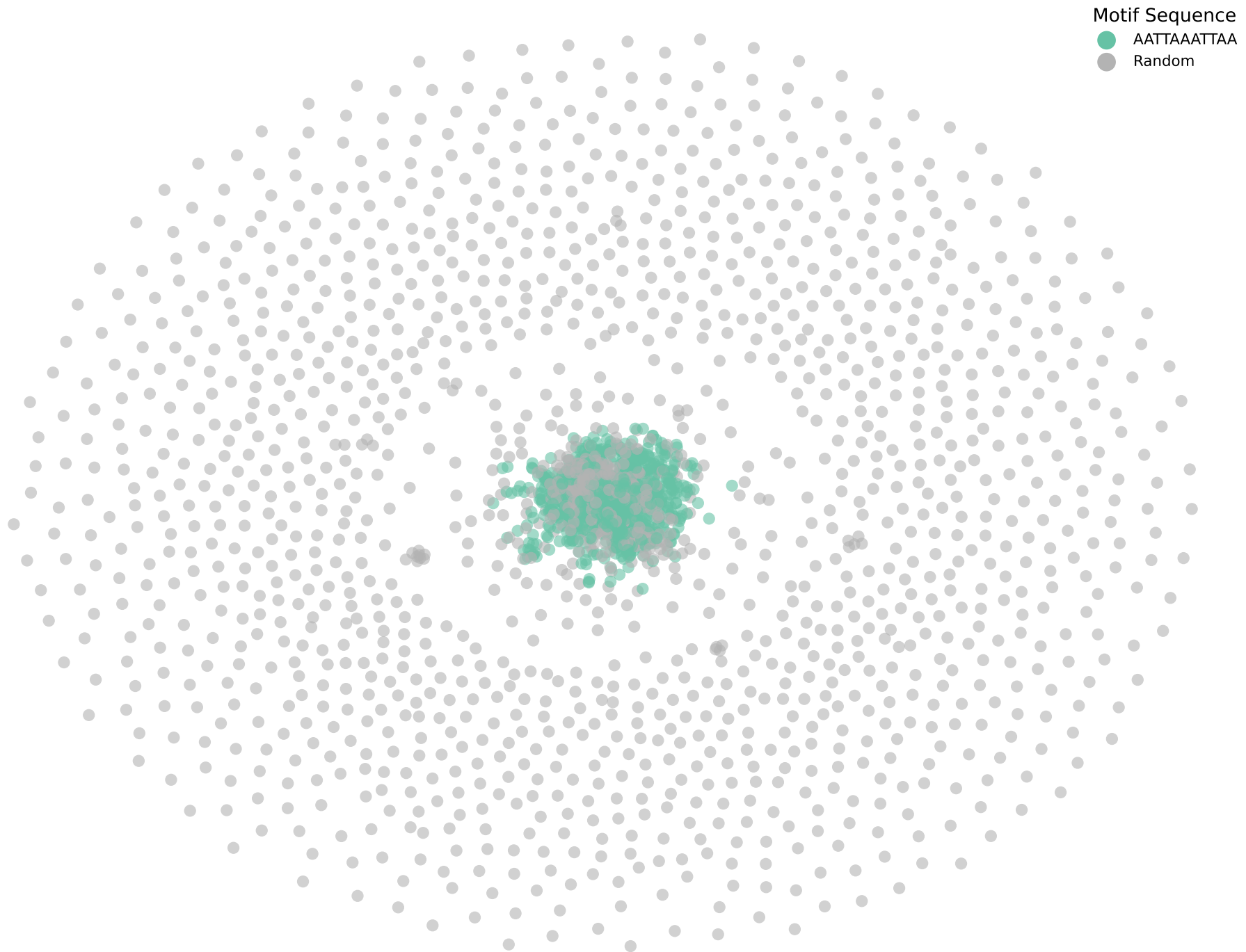

Supplement: Supplement 8 [file Supplemental_Data_1.zip › Supplemental_Data_1/ARX_TGCGTT20NTGC_Z_3/ARX_TGCGTT20NTGC_Z_3_KMAP.pdf]

MDS Plot - ARX\_TGCGTT20NTGC\_Z\_3

Motif Sequence

AATTAAATTAA

Random

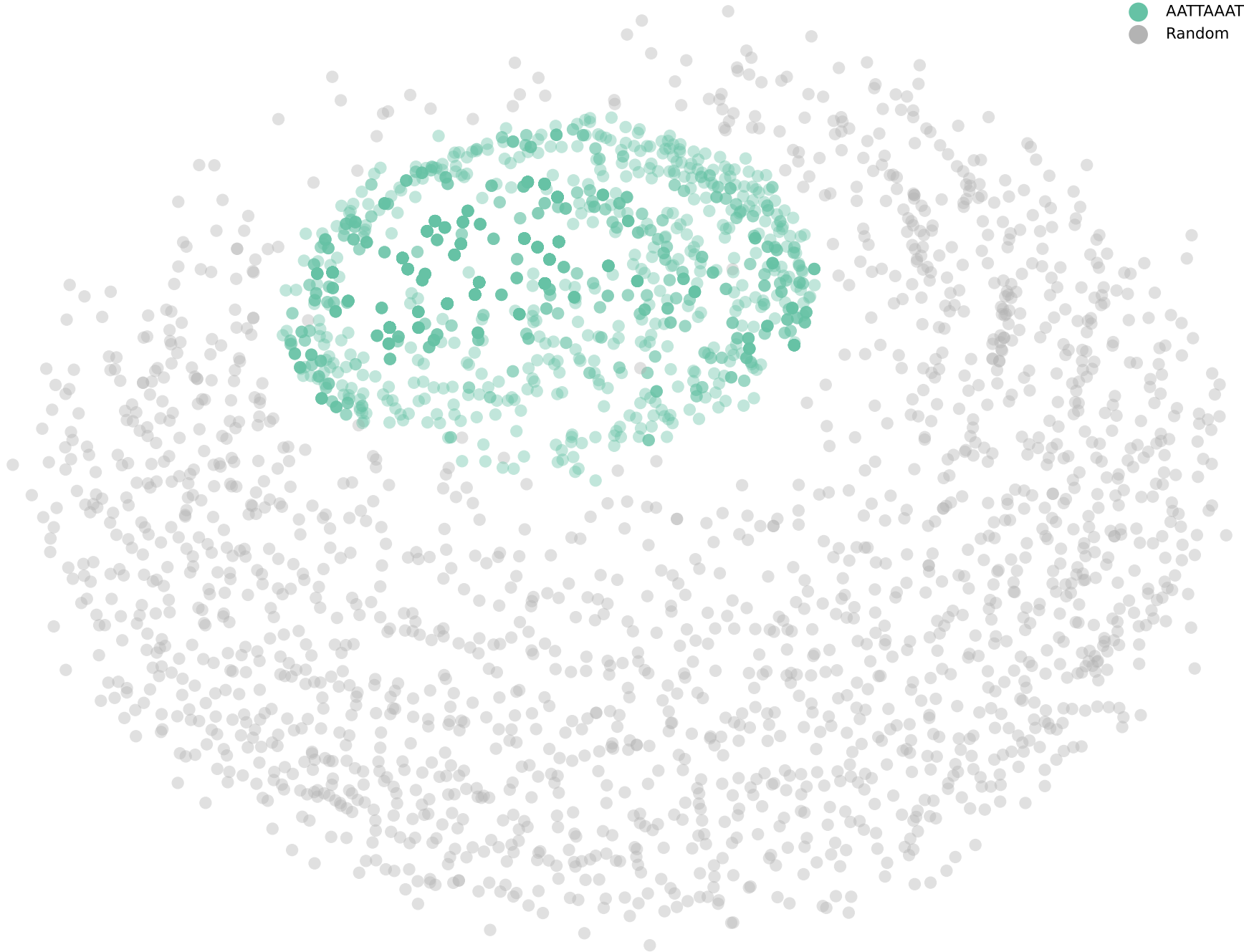

Supplement: Supplement 8 [file Supplemental_Data_1.zip › Supplemental_Data_1/ARX_TGCGTT20NTGC_Z_3/ARX_TGCGTT20NTGC_Z_3_MDS.pdf]

PCA Plot - ARX\_TGCGTT20NTGC\_Z\_3

Motif Sequence

- AATTAAATTAA
- Random

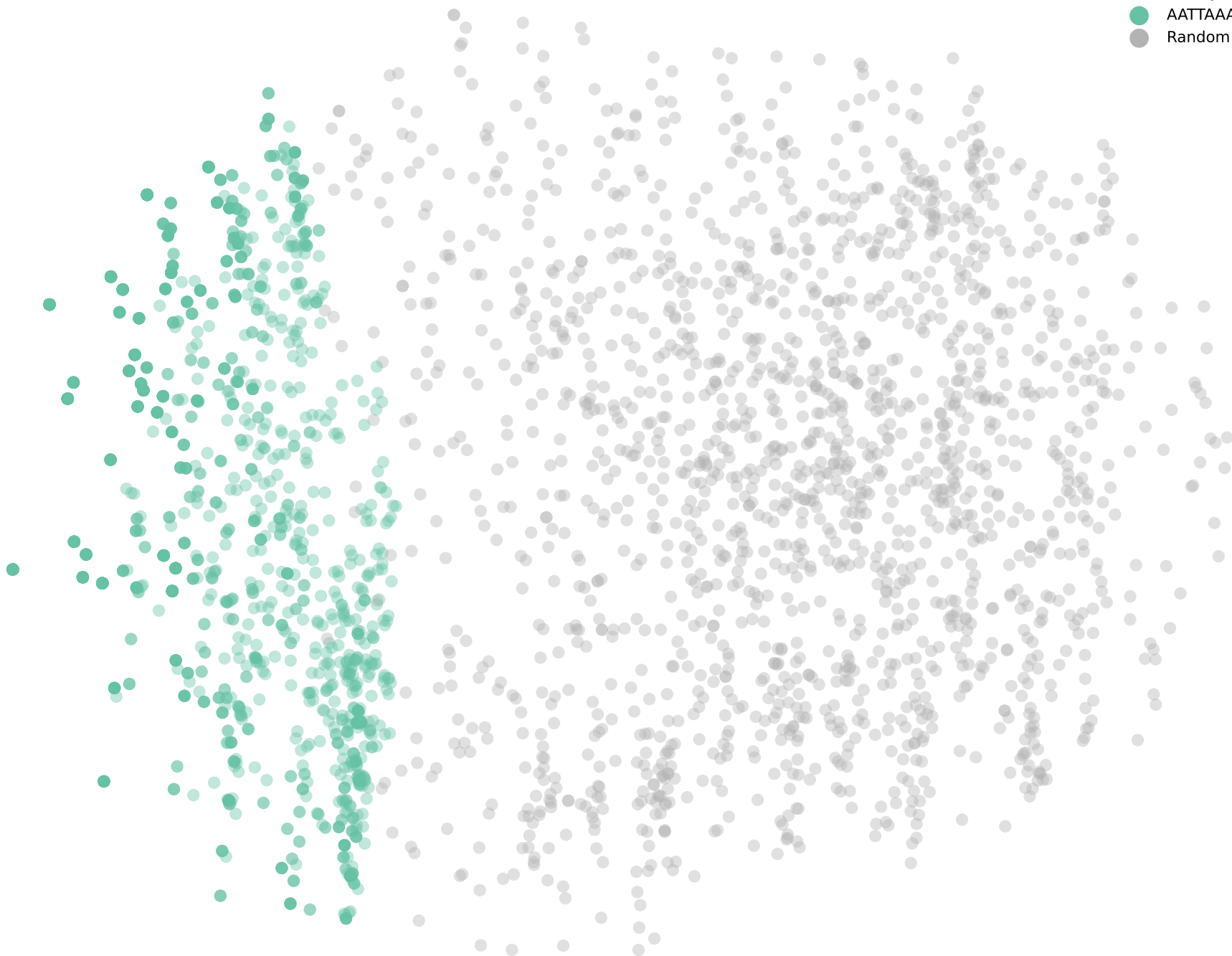

Supplement: Supplement 8 [file Supplemental_Data_1.zip › Supplemental_Data_1/ARX_TGCGTT20NTGC_Z_3/ARX_TGCGTT20NTGC_Z_3_PCA.pdf]

tSNE Plot - ARX\_TGCGTT20NTGC\_Z\_3

Motif Sequence

- AATTAAATTAA
- Random

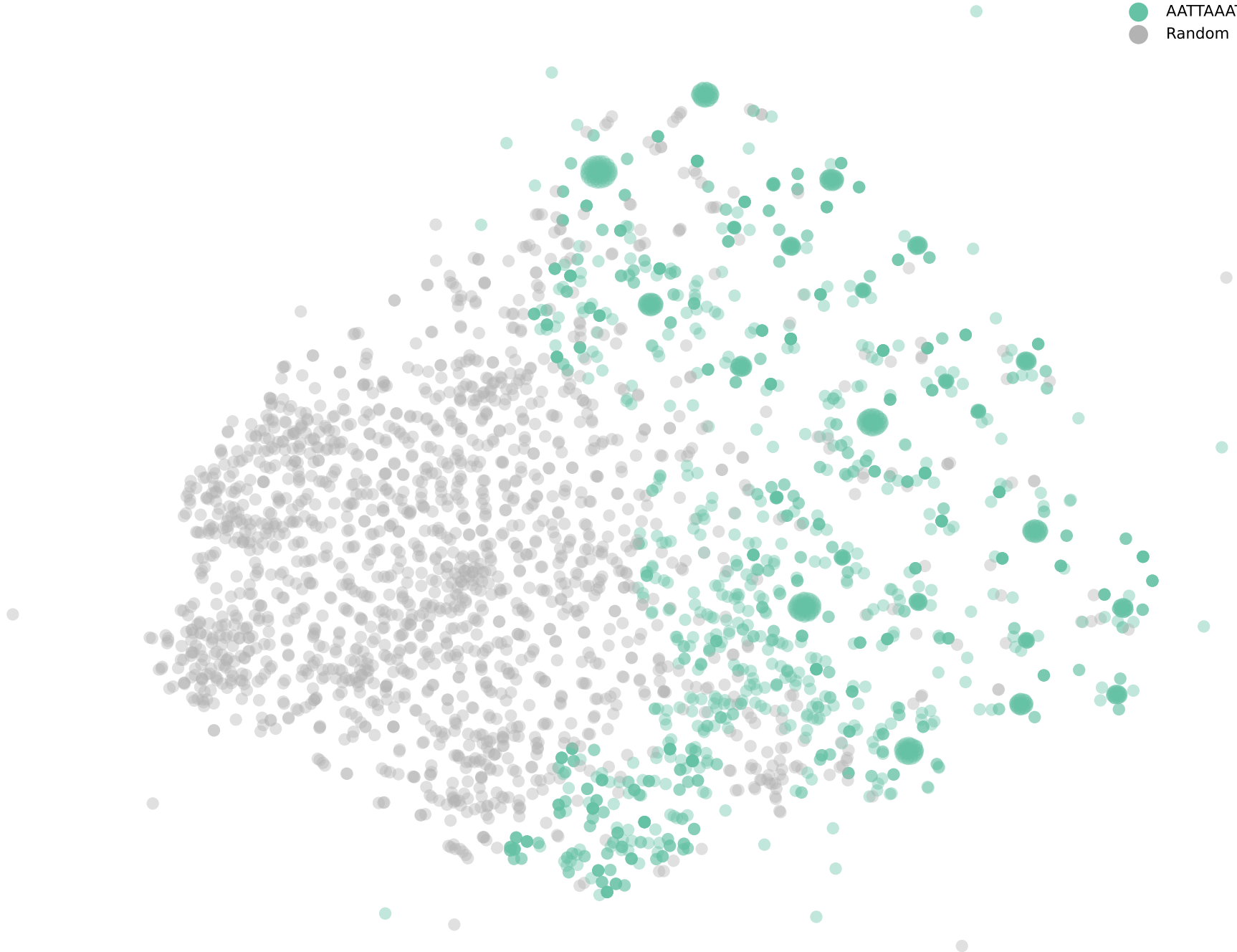

Supplement: Supplement 8 [file Supplemental_Data_1.zip › Supplemental_Data_1/ARX_TGCGTT20NTGC_Z_3/ARX_TGCGTT20NTGC_Z_3_tSNE.pdf]

UMAP Plot - ARX\_TGCGTT20NTGC\_Z\_3

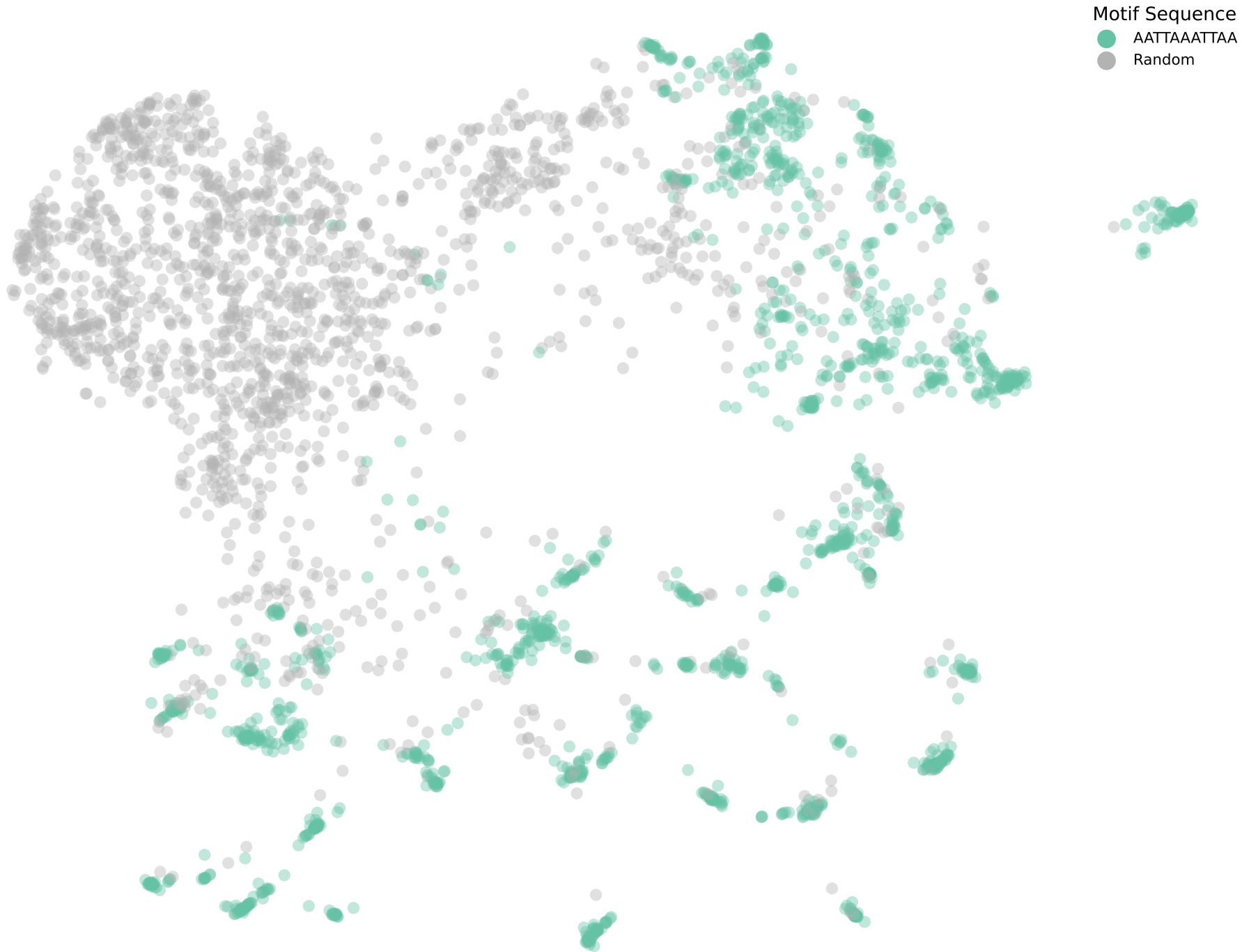

Supplement: Supplement 8 [file Supplemental_Data_1.zip › Supplemental_Data_1/ARX_TGCGTT20NTGC_Z_3/ARX_TGCGTT20NTGC_Z_3_UMAP.pdf]

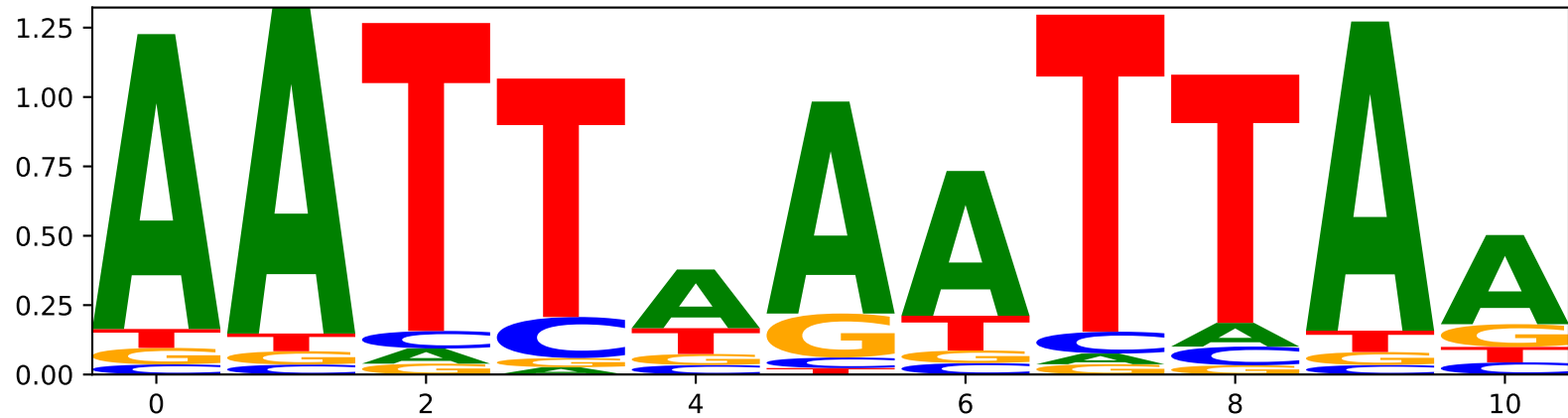

Supplement: Supplement 8 [file Supplemental_Data_1.zip › Supplemental_Data_1/ARX_TGCGTT20NTGC_Z_3/kmap_logo.pdf]

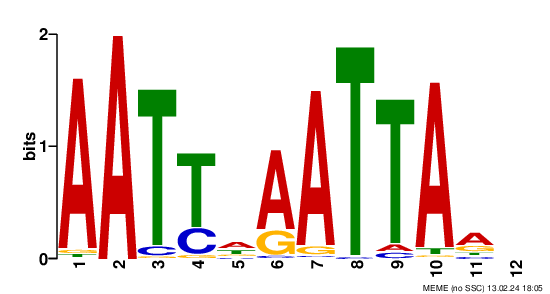

Supplement: Supplement 8 [file Supplemental_Data_1.zip › Supplemental_Data_1/ARX_TGCGTT20NTGC_Z_3/meme_logo.png]

KMAP LD Plot - ARX\_TGCGTT20NTGC\_Z\_4

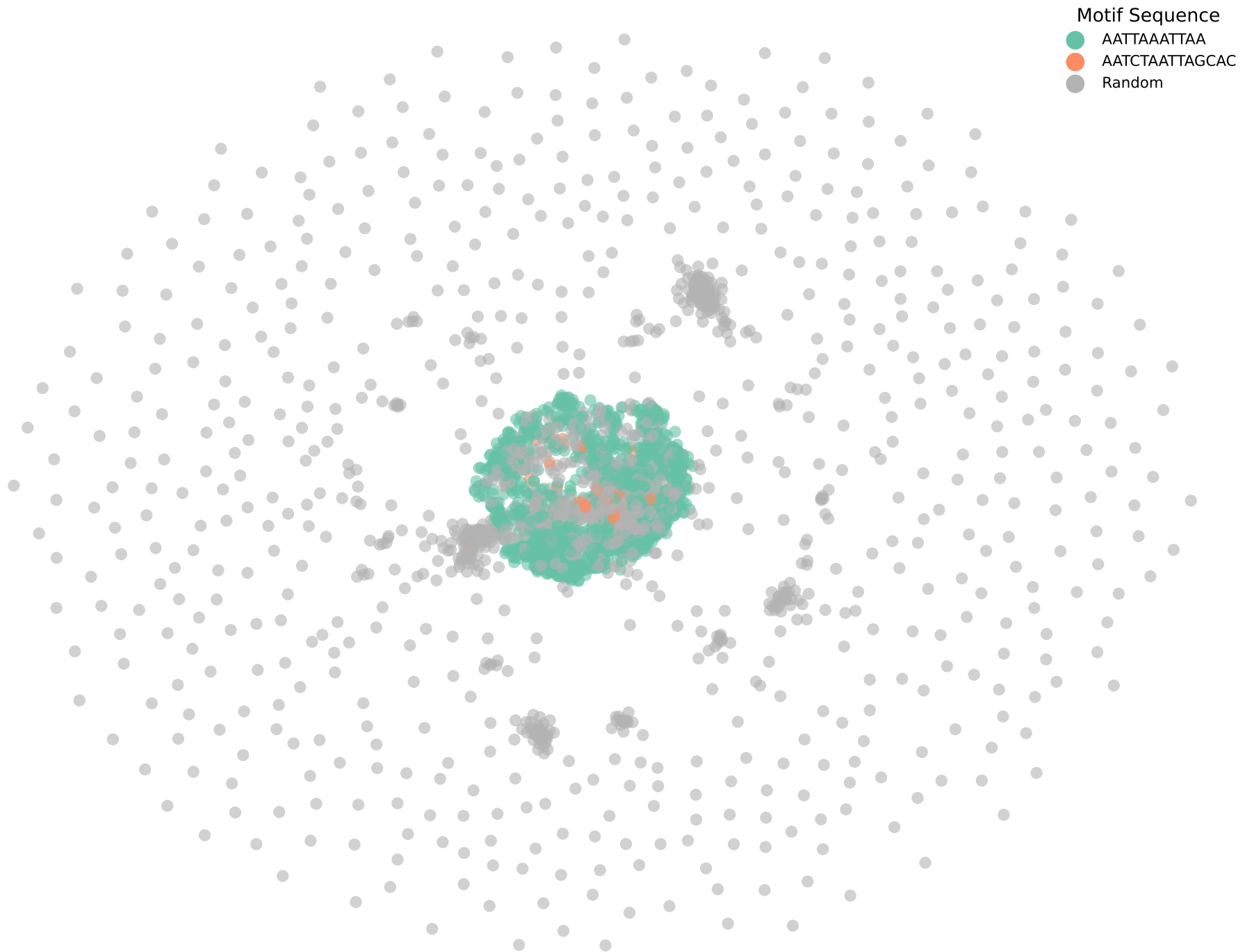

Supplement: Supplement 8 [file Supplemental_Data_1.zip › Supplemental_Data_1/ARX_TGCGTT20NTGC_Z_4/ARX_TGCGTT20NTGC_Z_4_KMAP.pdf]

MDS Plot - ARX\_TGCGTT20NTGC\_Z\_4

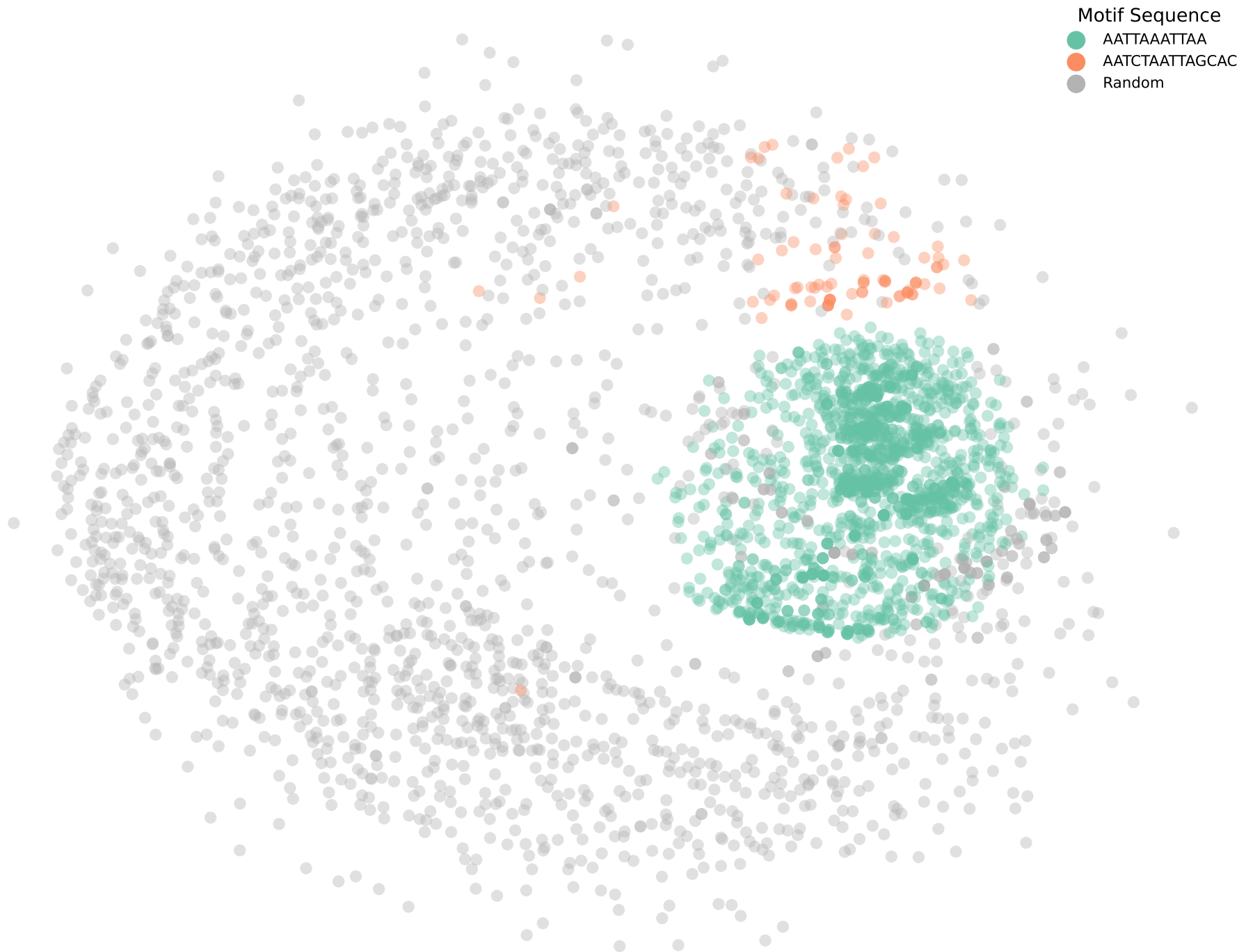

Supplement: Supplement 8 [file Supplemental_Data_1.zip › Supplemental_Data_1/ARX_TGCGTT20NTGC_Z_4/ARX_TGCGTT20NTGC_Z_4_MDS.pdf]

PCA Plot - ARX\_TGCGTT20NTGC\_Z\_4

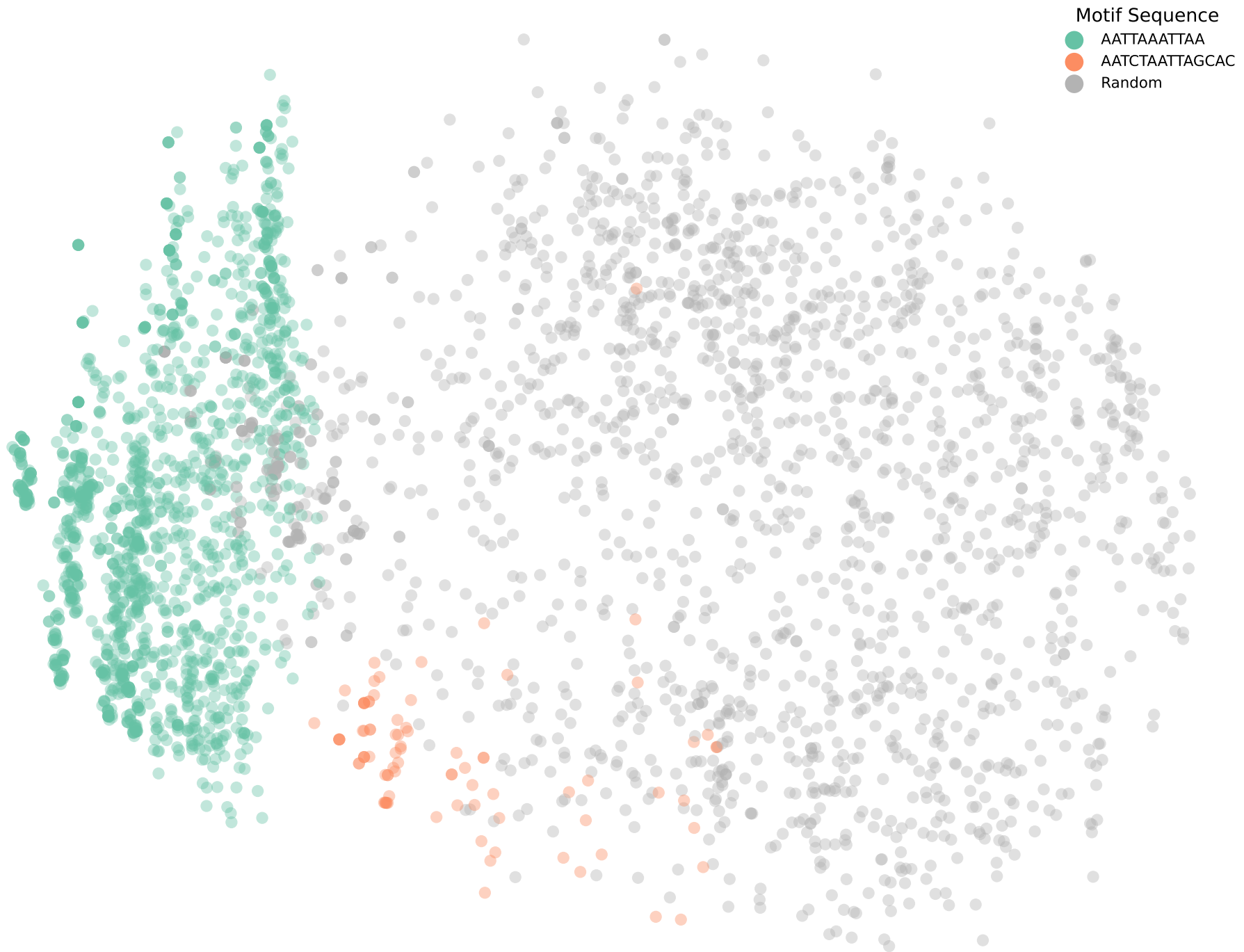

Supplement: Supplement 8 [file Supplemental_Data_1.zip › Supplemental_Data_1/ARX_TGCGTT20NTGC_Z_4/ARX_TGCGTT20NTGC_Z_4_PCA.pdf]

tSNE Plot - ARX\_TGCGTT20NTGC\_Z\_4

Motif Sequence

- AATTAAATTAA
- AATCTAATTAGCAC
- Random

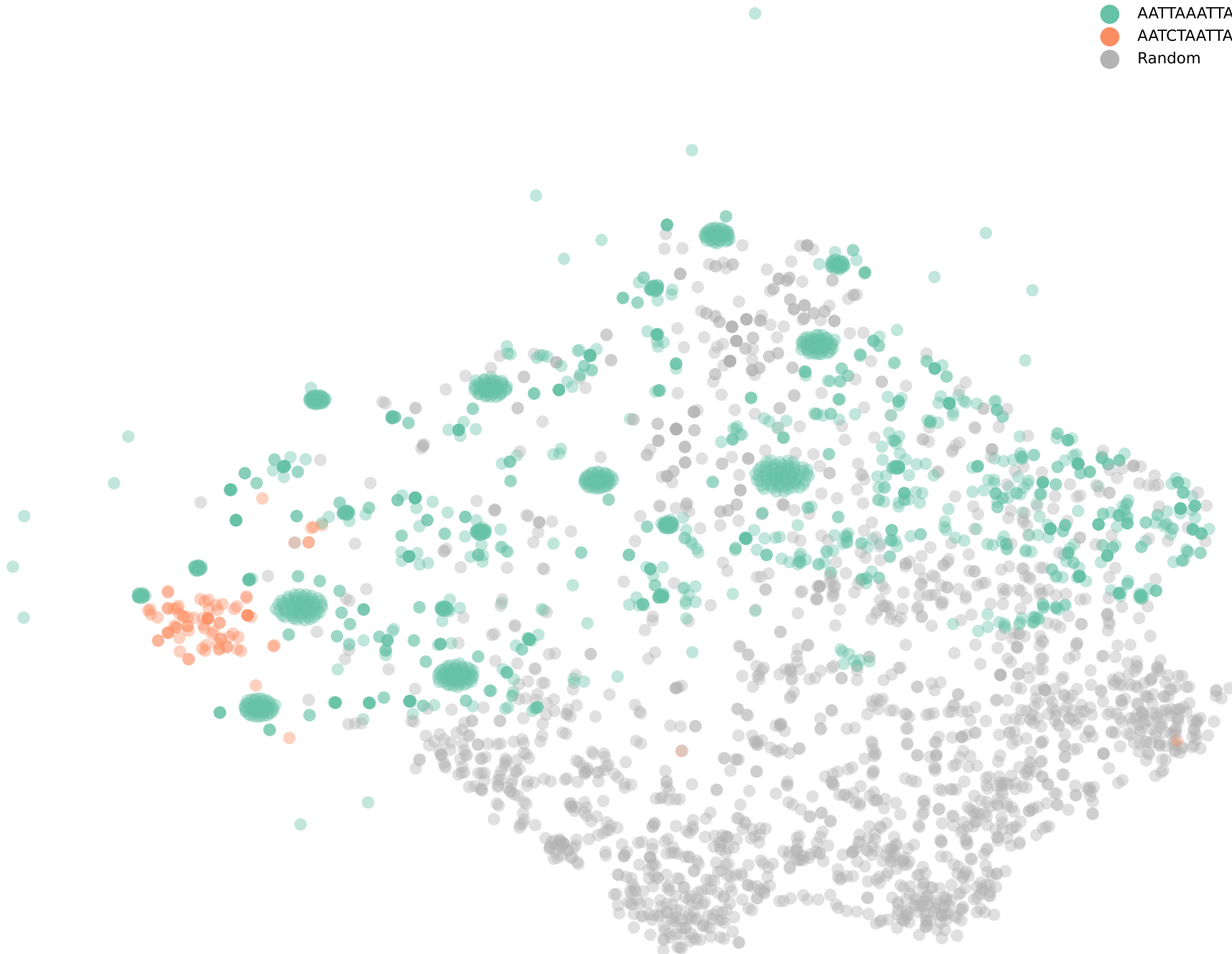

Supplement: Supplement 8 [file Supplemental_Data_1.zip › Supplemental_Data_1/ARX_TGCGTT20NTGC_Z_4/ARX_TGCGTT20NTGC_Z_4_tSNE.pdf]

UMAP Plot - ARX\_TGCGTT20NTGC\_Z\_4

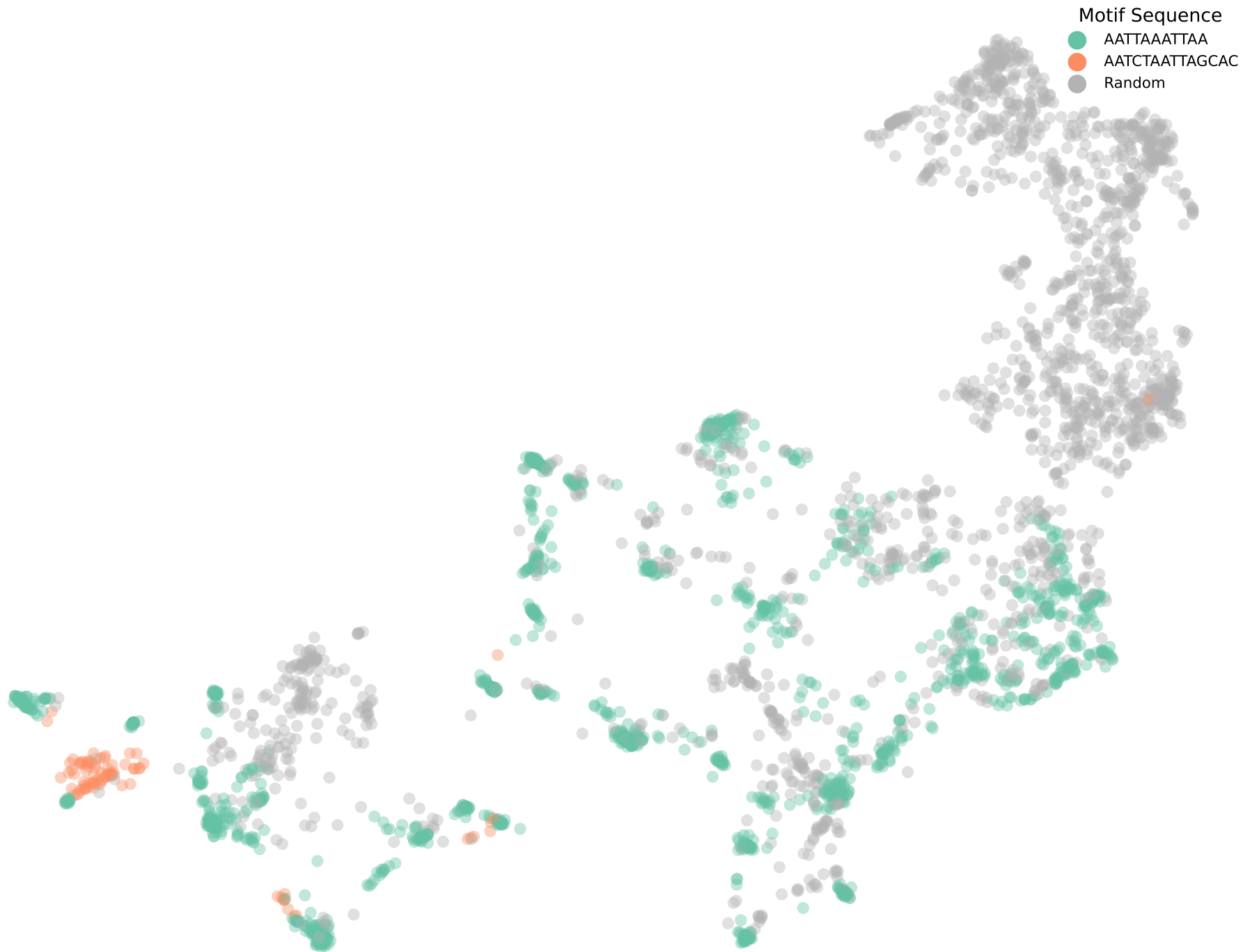

Supplement: Supplement 8 [file Supplemental_Data_1.zip › Supplemental_Data_1/ARX_TGCGTT20NTGC_Z_4/ARX_TGCGTT20NTGC_Z_4_UMAP.pdf]

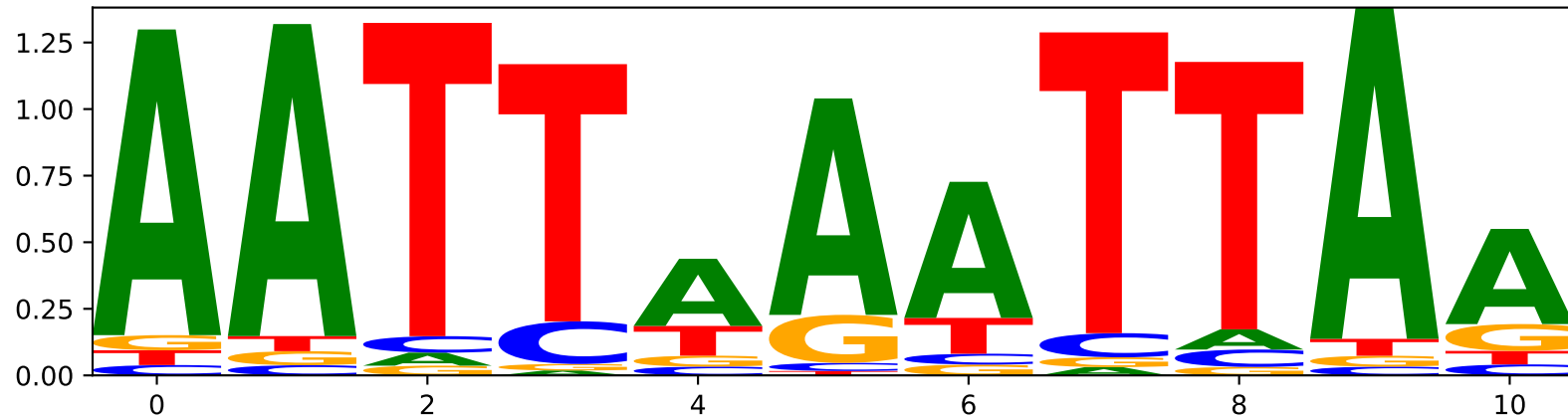

Supplement: Supplement 8 [file Supplemental_Data_1.zip › Supplemental_Data_1/ARX_TGCGTT20NTGC_Z_4/kmap_logo.pdf]

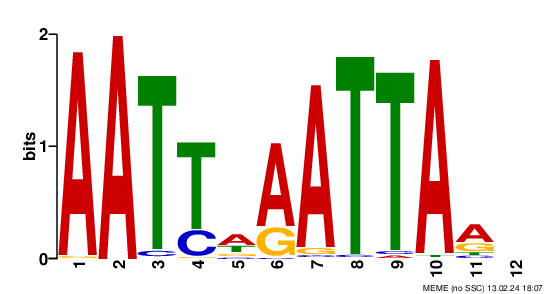

Supplement: Supplement 8 [file Supplemental_Data_1.zip › Supplemental_Data_1/ARX_TGCGTT20NTGC_Z_4/meme_logo.png]

KMAP LD Plot - Ar\_TCTAAT20NCG\_P\_3

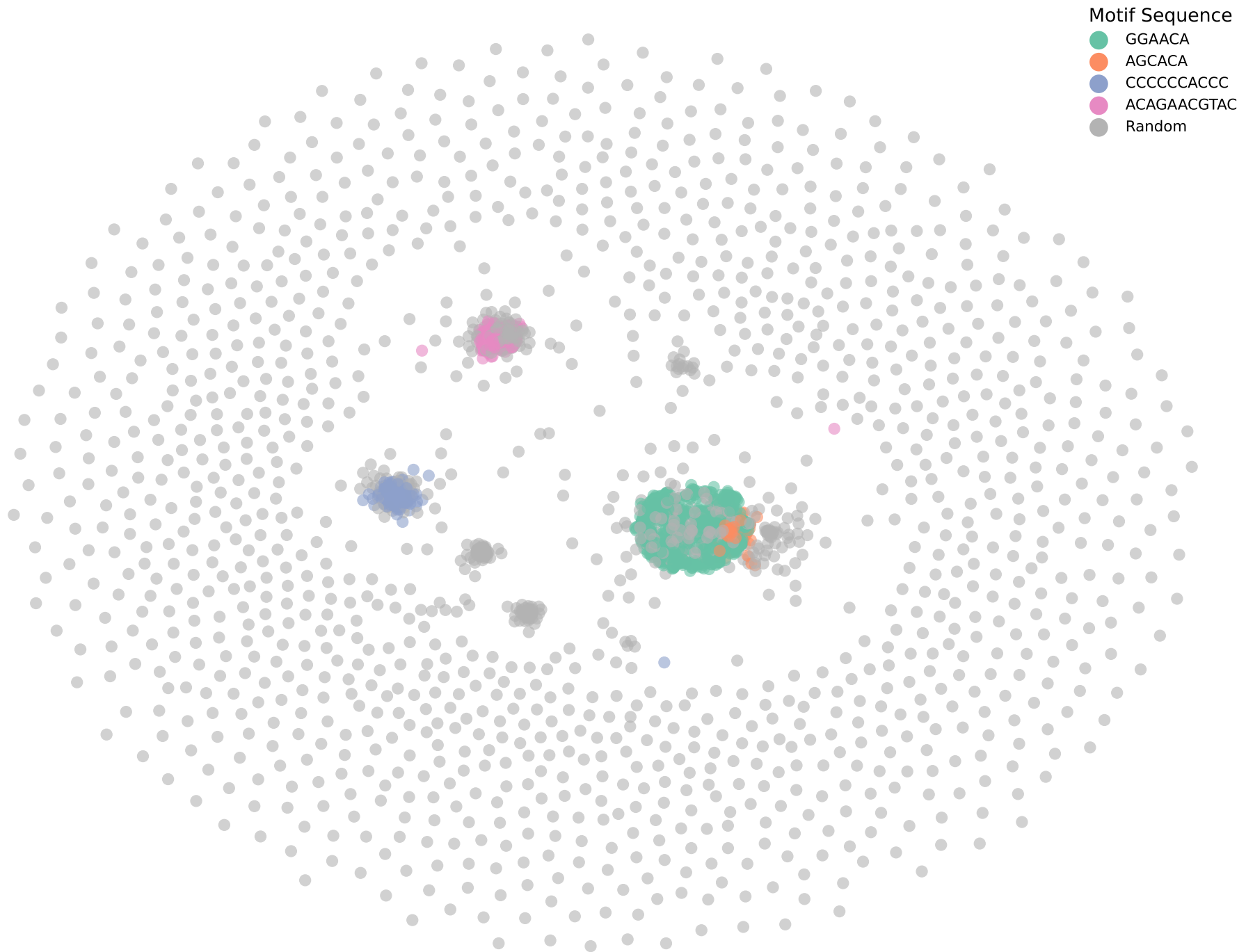

Supplement: Supplement 8 [file Supplemental_Data_1.zip › Supplemental_Data_1/Ar_TCTAAT20NCG_P_3/Ar_TCTAAT20NCG_P_3_KMAP.pdf]

MDS Plot - Ar\_TCTAAT20NCG\_P\_3

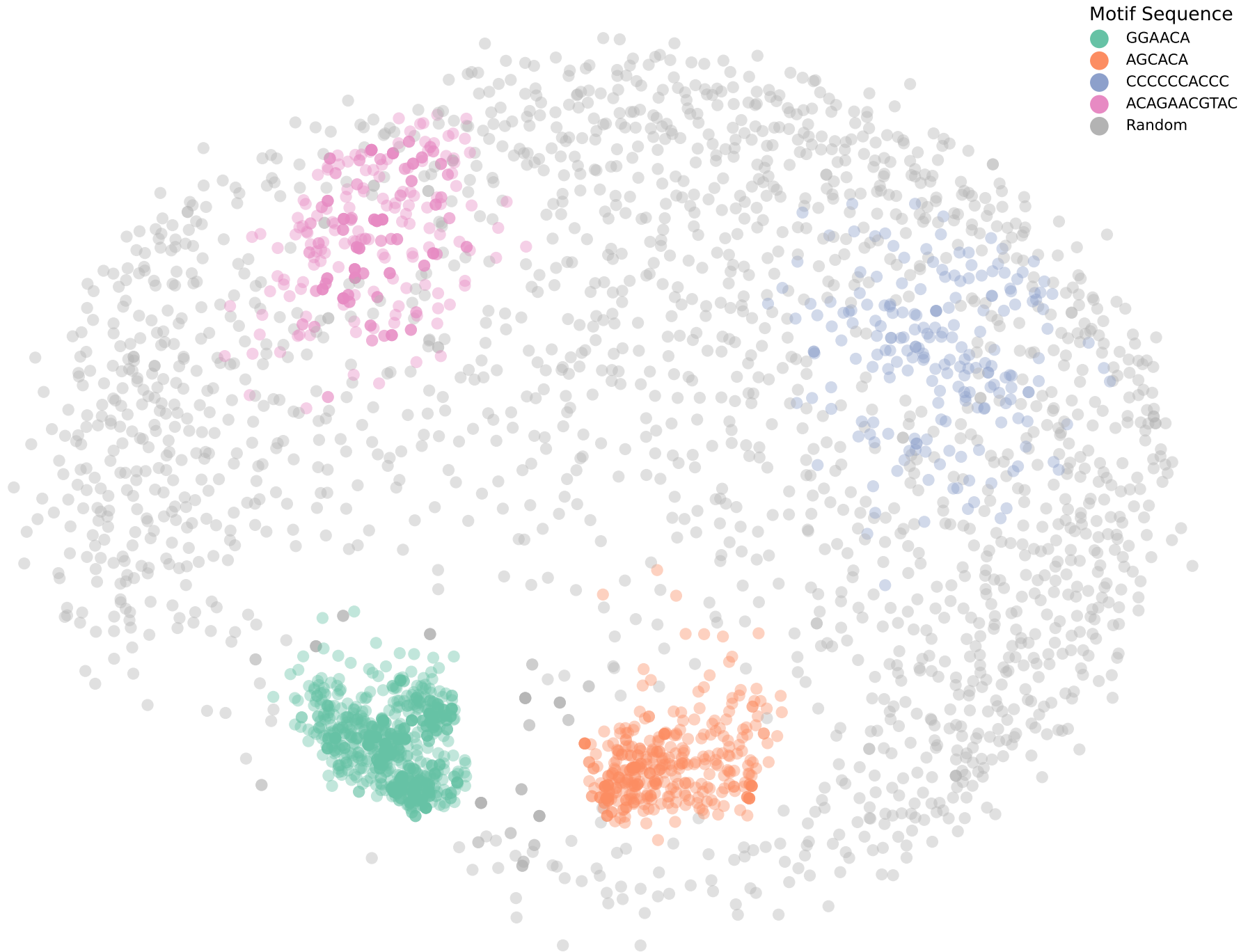

Supplement: Supplement 8 [file Supplemental_Data_1.zip › Supplemental_Data_1/Ar_TCTAAT20NCG_P_3/Ar_TCTAAT20NCG_P_3_MDS.pdf]

PCA Plot - Ar\_TCTAAT20NCG\_P\_3

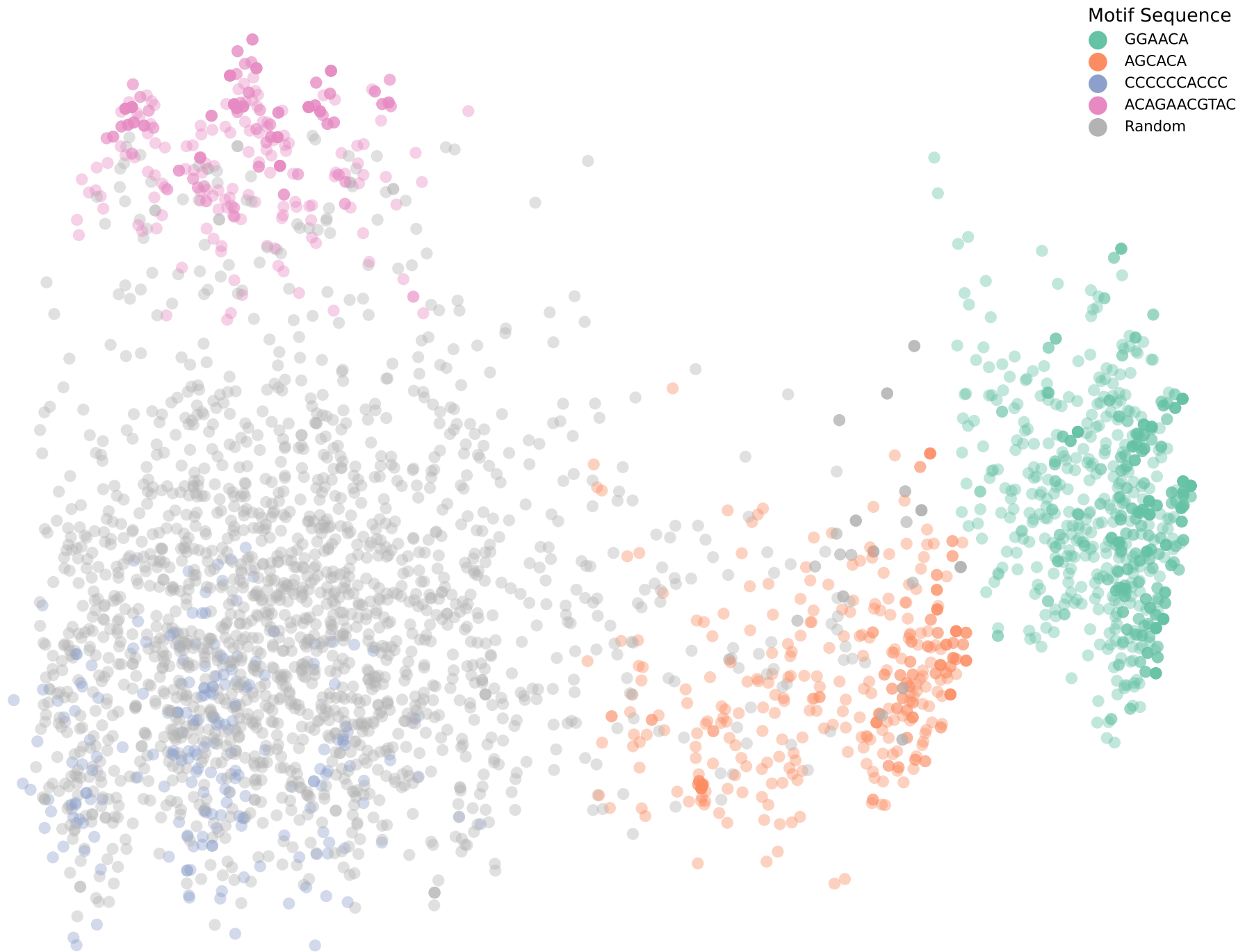

Supplement: Supplement 8 [file Supplemental_Data_1.zip › Supplemental_Data_1/Ar_TCTAAT20NCG_P_3/Ar_TCTAAT20NCG_P_3_PCA.pdf]

tSNE Plot - Ar\_TCTAAT20NCG\_P\_3

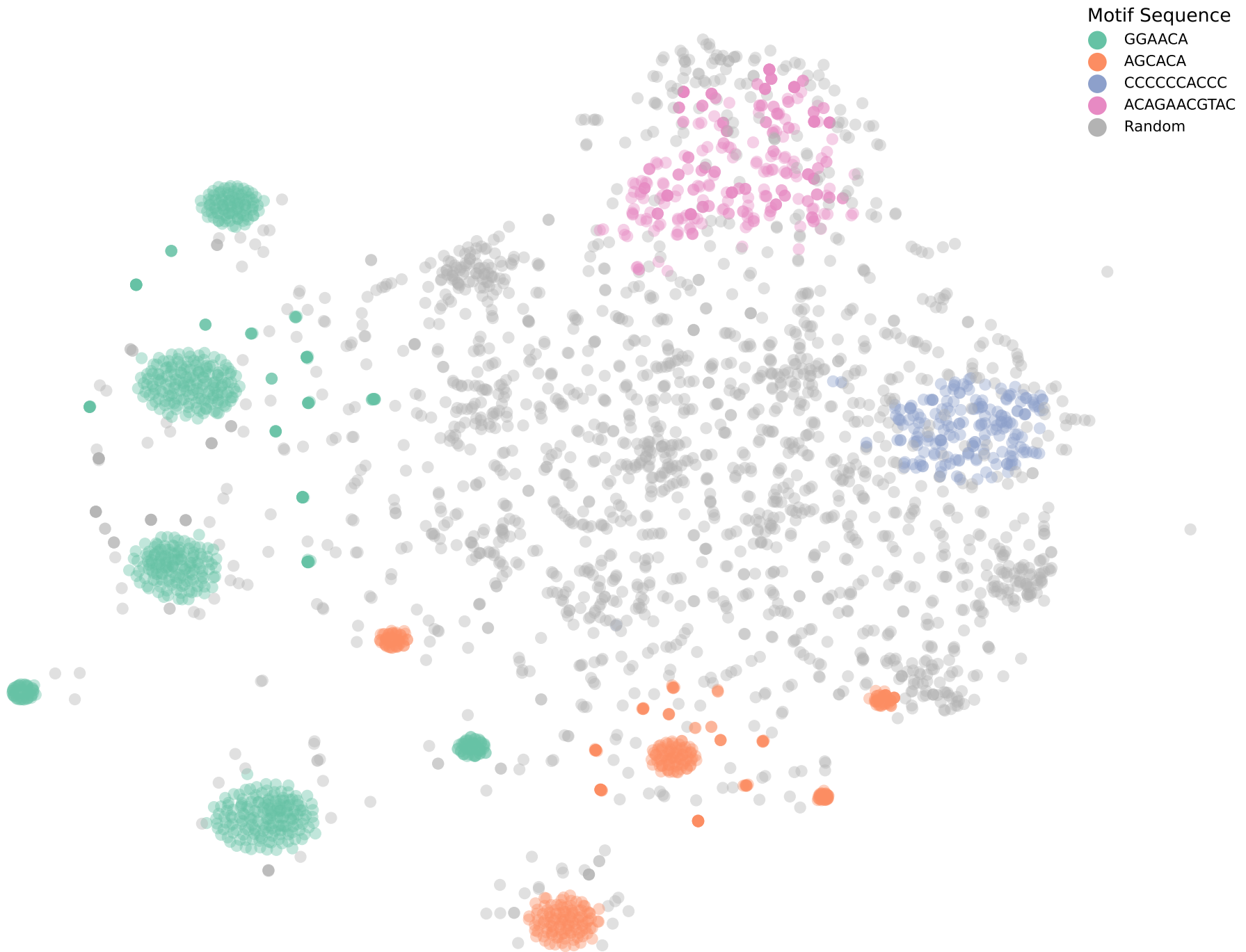

Supplement: Supplement 8 [file Supplemental_Data_1.zip › Supplemental_Data_1/Ar_TCTAAT20NCG_P_3/Ar_TCTAAT20NCG_P_3_tSNE.pdf]

UMAP Plot - Ar\_TCTAAT20NCG\_P\_3

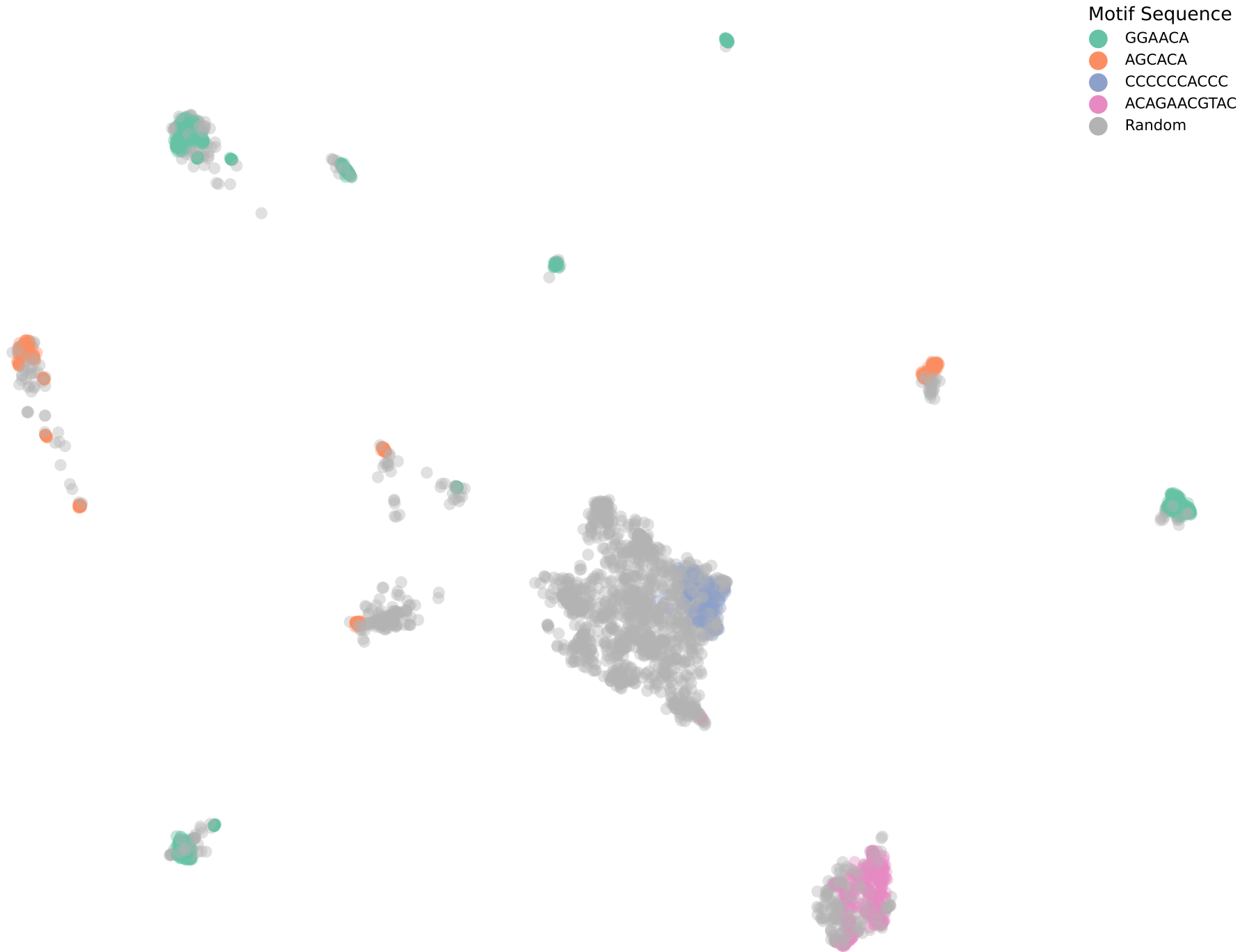

Supplement: Supplement 8 [file Supplemental_Data_1.zip › Supplemental_Data_1/Ar_TCTAAT20NCG_P_3/Ar_TCTAAT20NCG_P_3_UMAP.pdf]

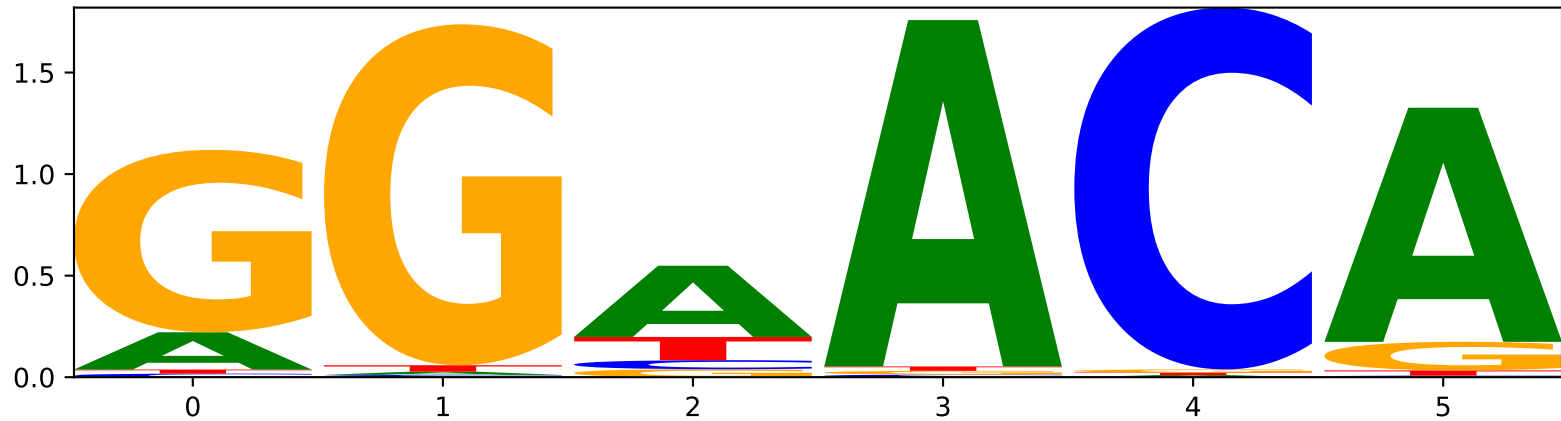

Supplement: Supplement 8 [file Supplemental_Data_1.zip › Supplemental_Data_1/Ar_TCTAAT20NCG_P_3/kmap_logo.pdf]

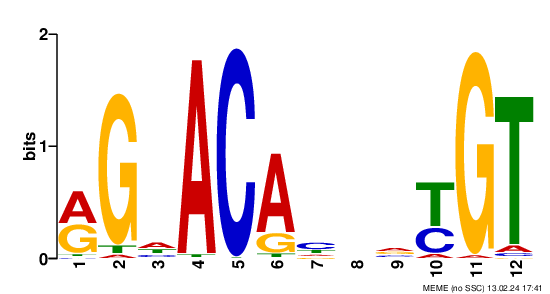

Supplement: Supplement 8 [file Supplemental_Data_1.zip › Supplemental_Data_1/Ar_TCTAAT20NCG_P_3/meme_logo.png]

KMAP LD Plot - Ar\_TCTAAT20NCG\_P\_4

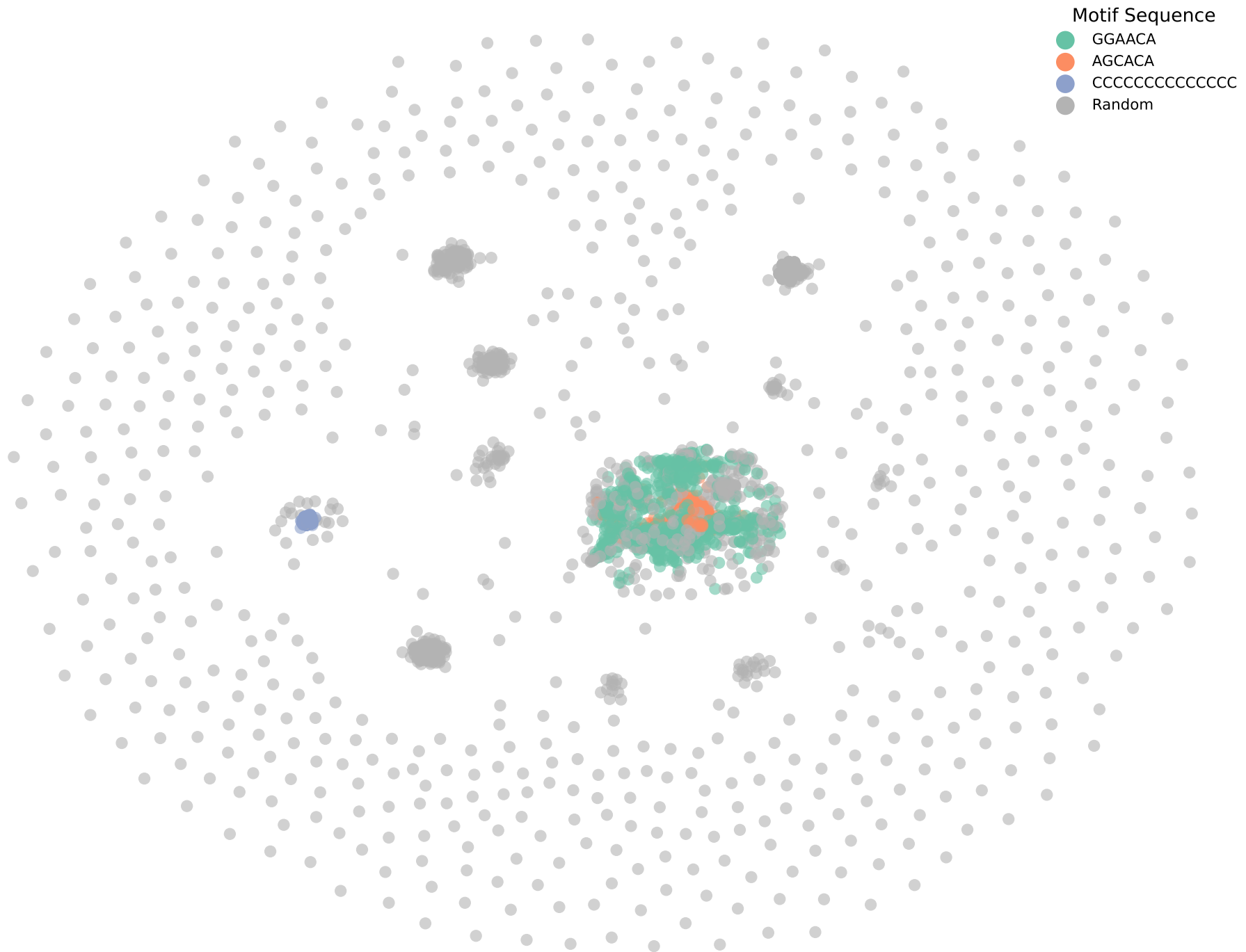

Supplement: Supplement 8 [file Supplemental_Data_1.zip › Supplemental_Data_1/Ar_TCTAAT20NCG_P_4/Ar_TCTAAT20NCG_P_4_KMAP.pdf]

MDS Plot - Ar\_TCTAAT20NCG\_P\_4

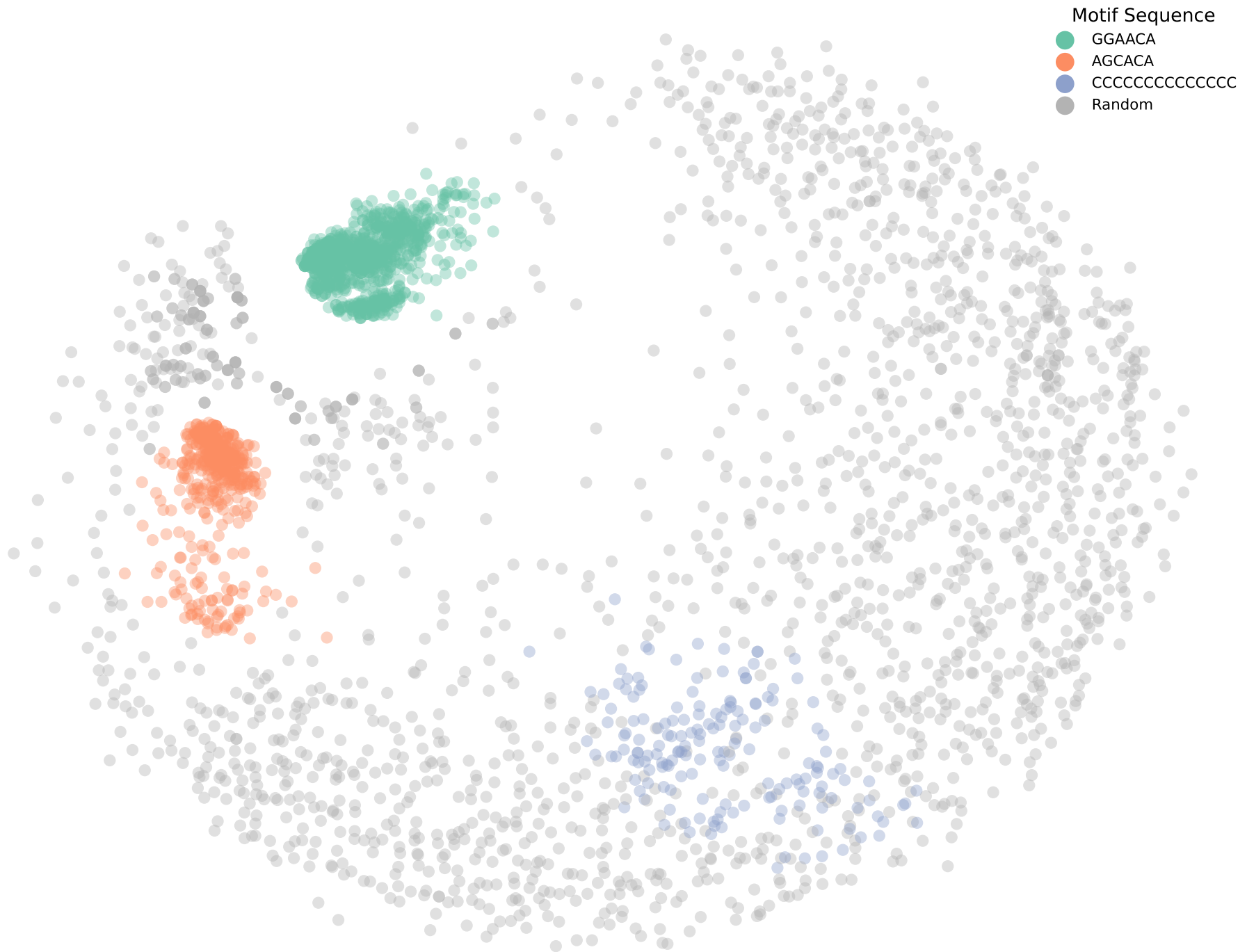

Supplement: Supplement 8 [file Supplemental_Data_1.zip › Supplemental_Data_1/Ar_TCTAAT20NCG_P_4/Ar_TCTAAT20NCG_P_4_MDS.pdf]

PCA Plot - Ar\_TCTAAT20NCG\_P\_4

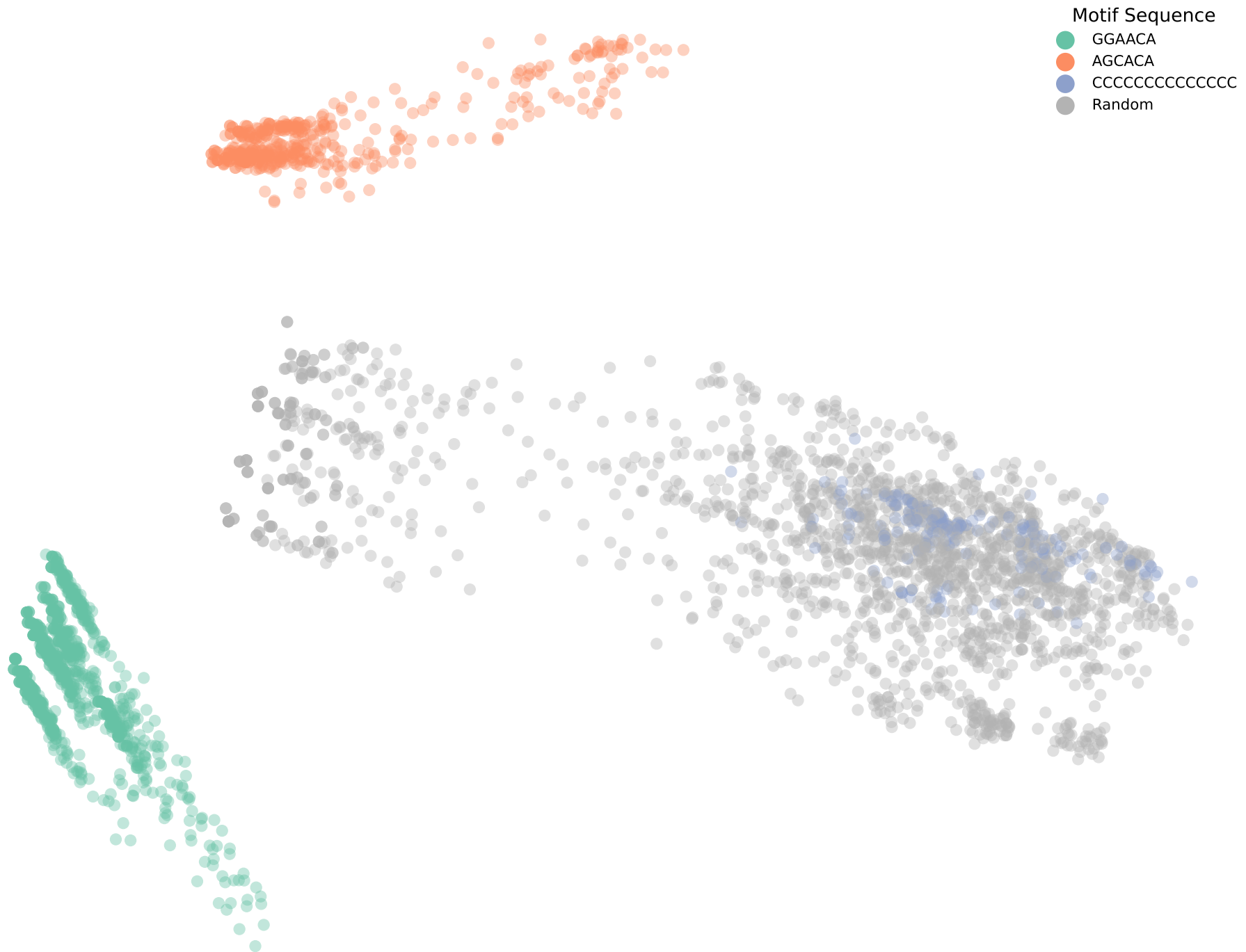

Supplement: Supplement 8 [file Supplemental_Data_1.zip › Supplemental_Data_1/Ar_TCTAAT20NCG_P_4/Ar_TCTAAT20NCG_P_4_PCA.pdf]

tSNE Plot - Ar\_TCTAAT20NCG\_P\_4

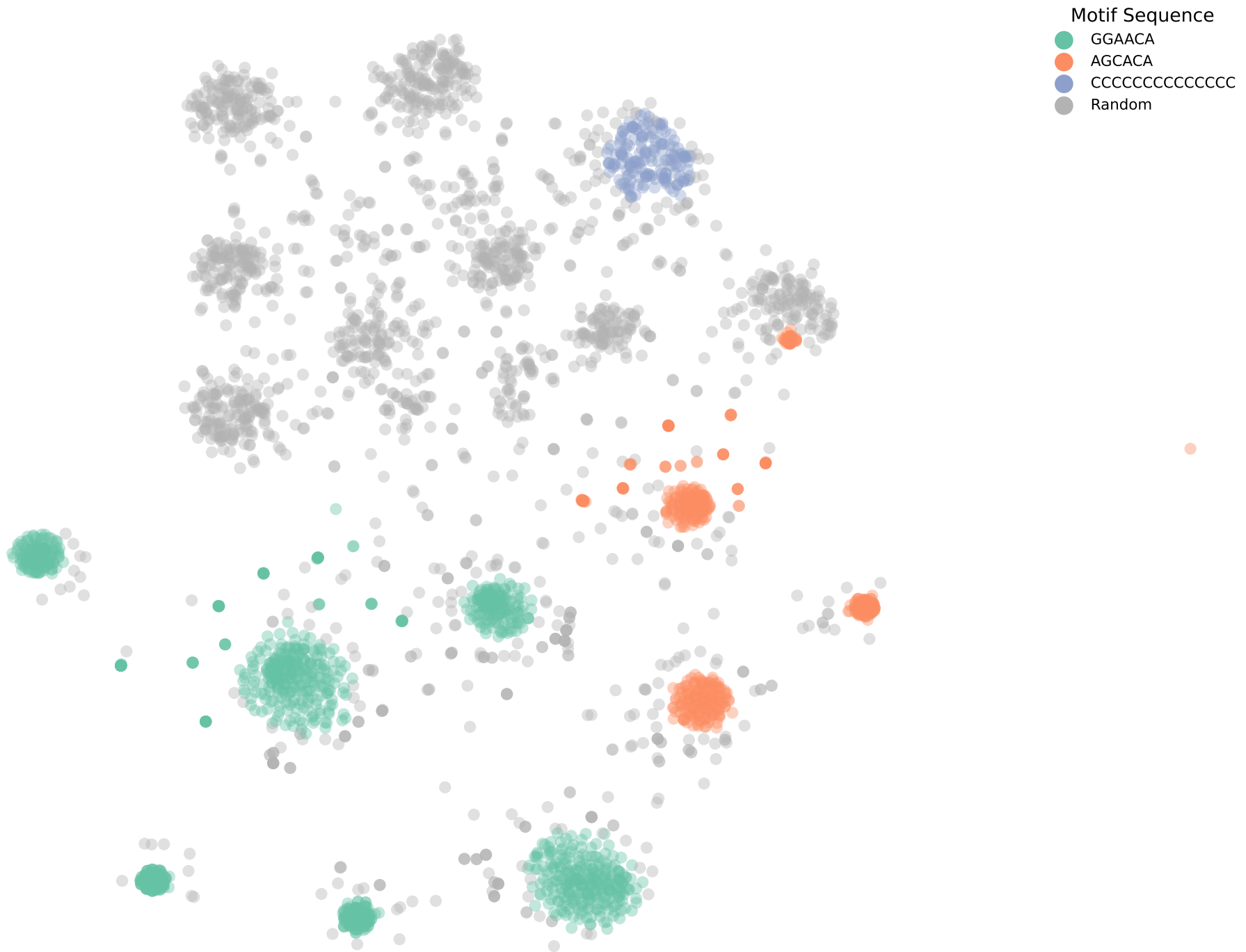

Supplement: Supplement 8 [file Supplemental_Data_1.zip › Supplemental_Data_1/Ar_TCTAAT20NCG_P_4/Ar_TCTAAT20NCG_P_4_tSNE.pdf]

UMAP Plot - Ar\_TCTAAT20NCG\_P\_4

Motif Sequence

- GGAACA
- AGCACA
- CCCCCCCCCCCCC
- Random

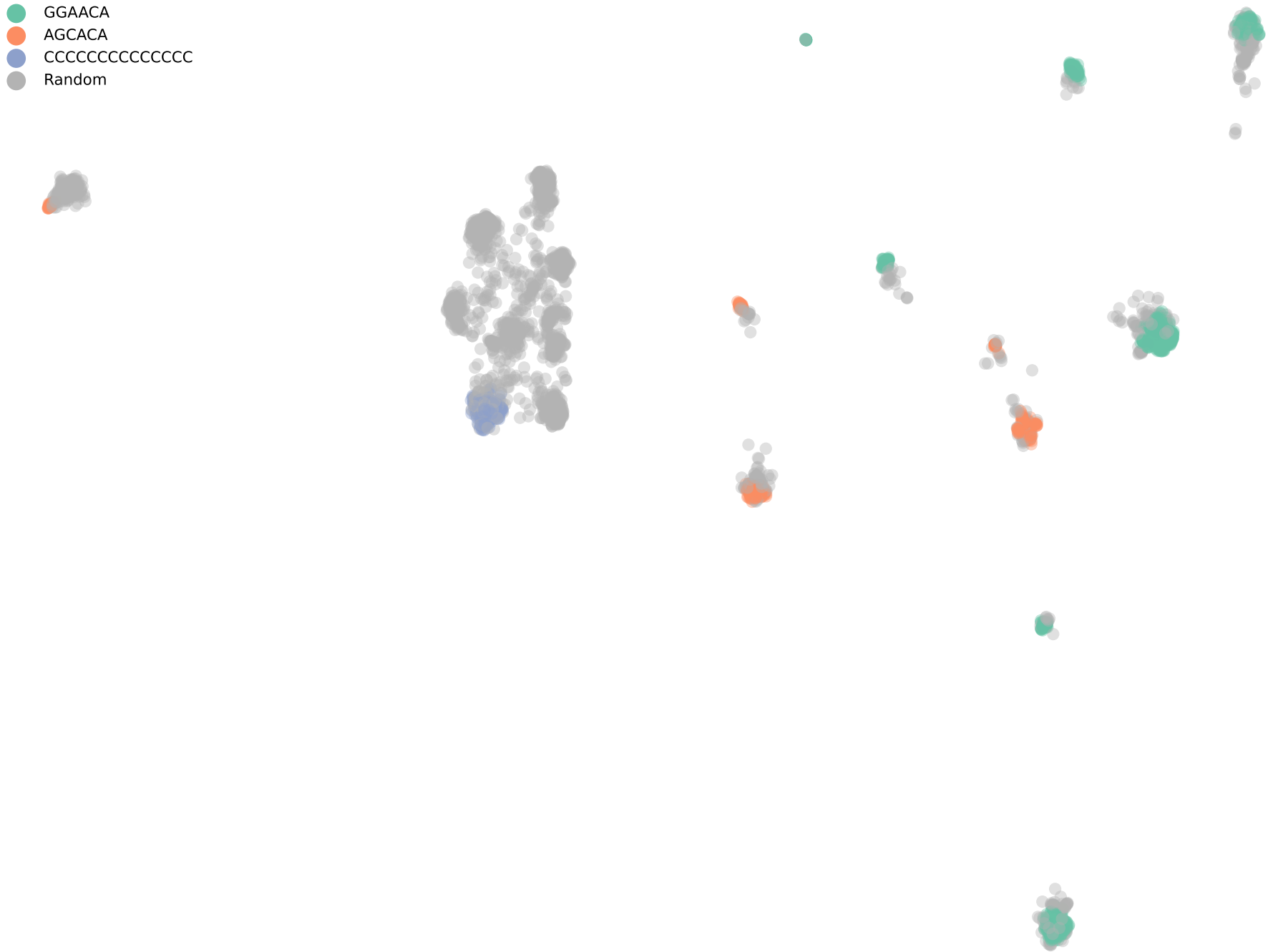

Supplement: Supplement 8 [file Supplemental_Data_1.zip › Supplemental_Data_1/Ar_TCTAAT20NCG_P_4/Ar_TCTAAT20NCG_P_4_UMAP.pdf]

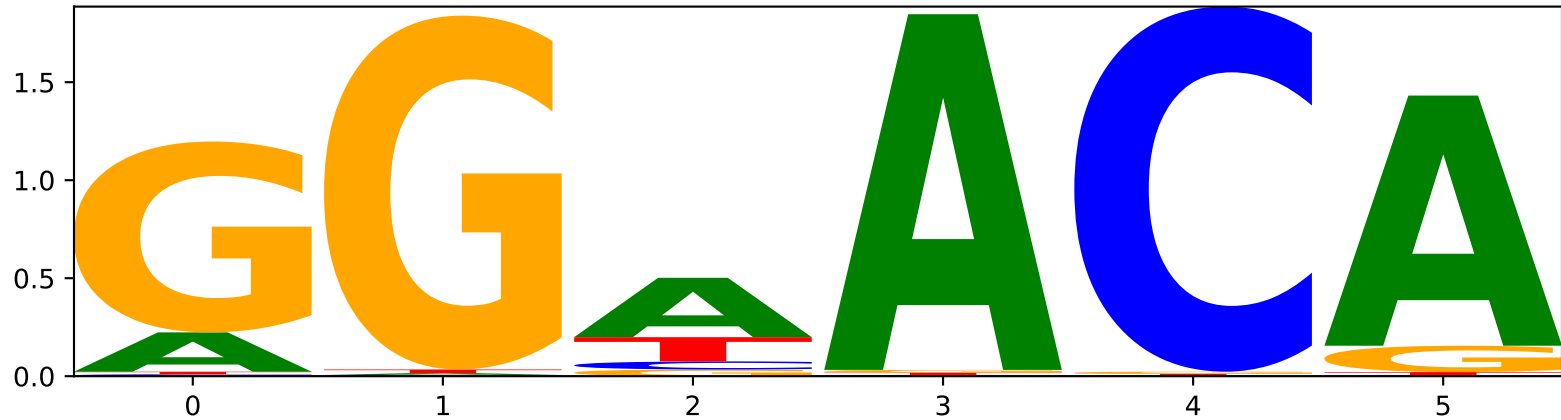

Supplement: Supplement 8 [file Supplemental_Data_1.zip › Supplemental_Data_1/Ar_TCTAAT20NCG_P_4/kmap_logo.pdf]

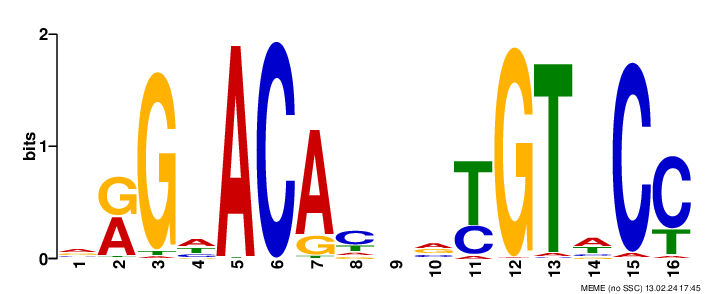

Supplement: Supplement 8 [file Supplemental_Data_1.zip › Supplemental_Data_1/Ar_TCTAAT20NCG_P_4/meme_logo.png]

KMAP LD Plot - Ar\_TCTAAT20NCG\_P\_5

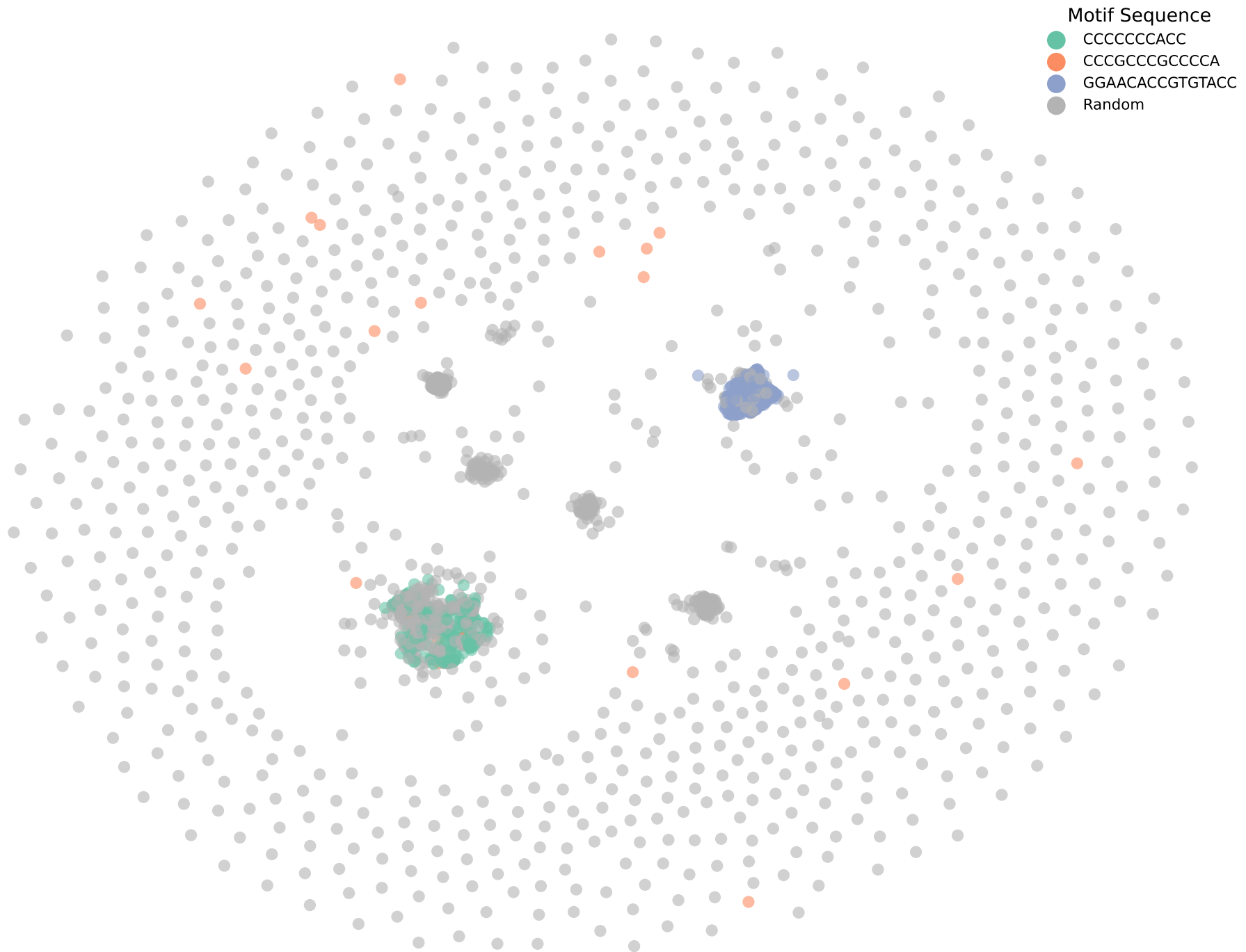

Supplement: Supplement 8 [file Supplemental_Data_1.zip › Supplemental_Data_1/Ar_TCTAAT20NCG_P_5/Ar_TCTAAT20NCG_P_5_KMAP.pdf]

MDS Plot - Ar\_TCTAAT20NCG\_P\_5

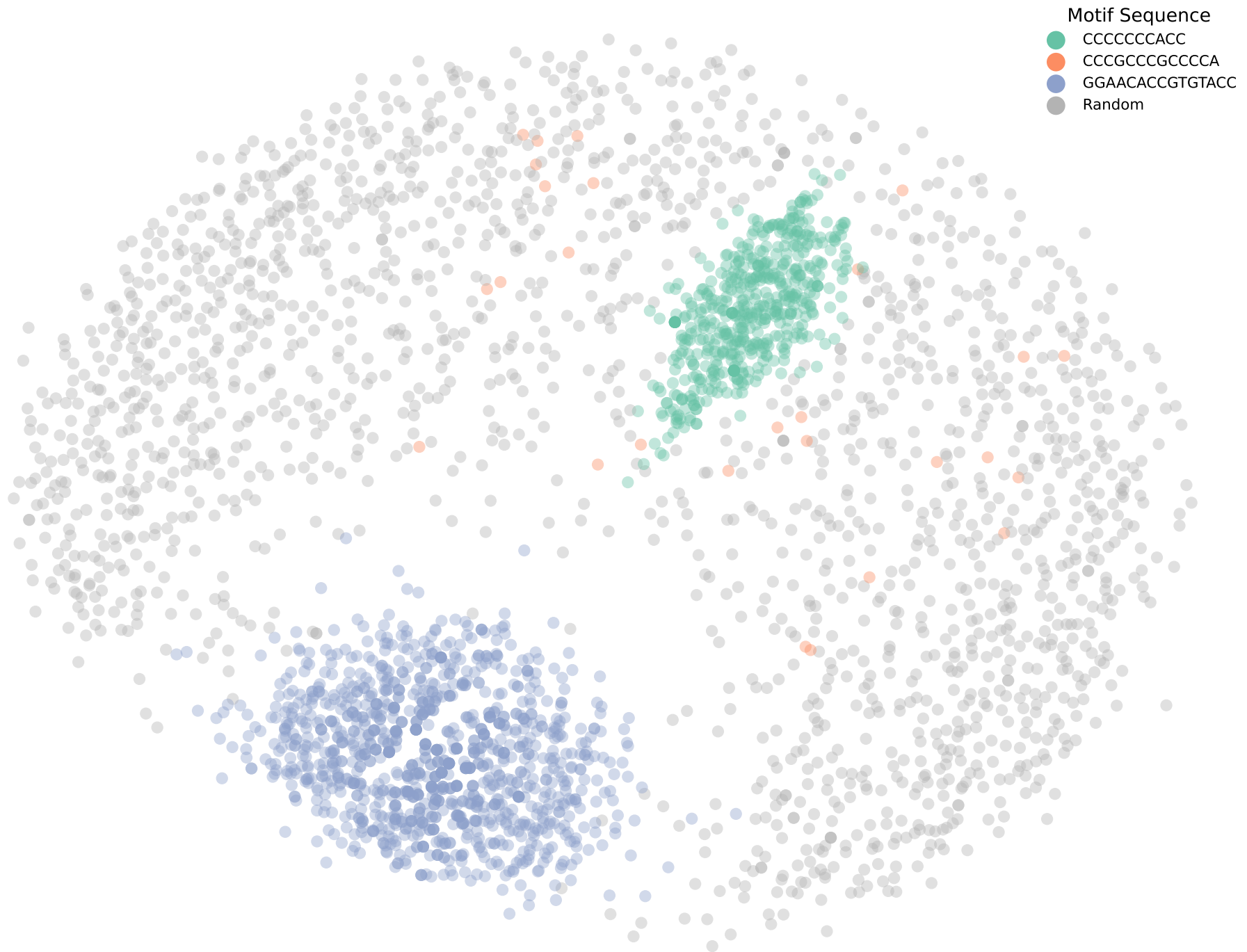

Supplement: Supplement 8 [file Supplemental_Data_1.zip › Supplemental_Data_1/Ar_TCTAAT20NCG_P_5/Ar_TCTAAT20NCG_P_5_MDS.pdf]

PCA Plot - Ar\_TCTAAT20NCG\_P\_5

Motif Sequence

- CCCCCCCACC
- CCCGCCCGCCCCA
- GGAACACCGTGTACC
- Random

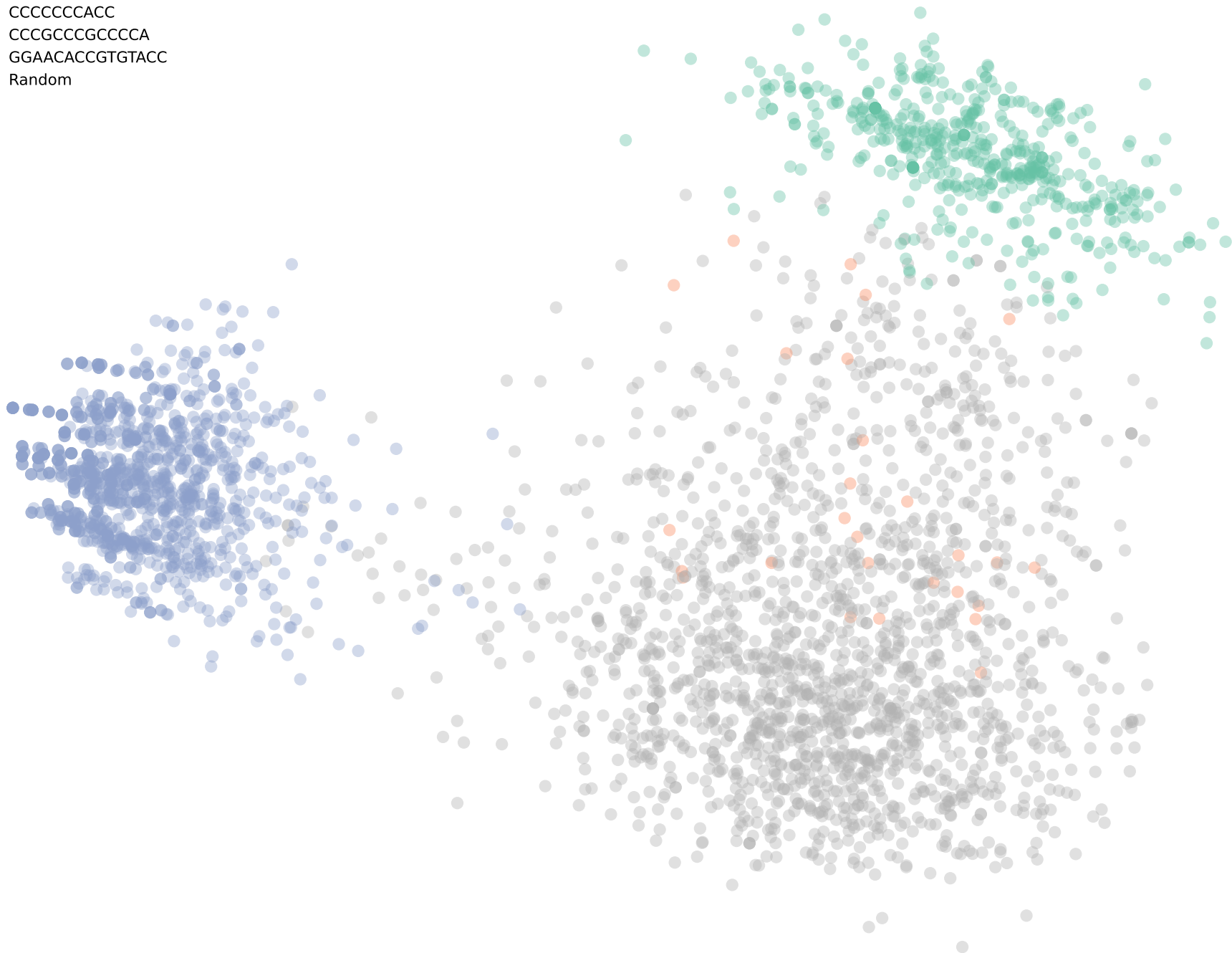

Supplement: Supplement 8 [file Supplemental_Data_1.zip › Supplemental_Data_1/Ar_TCTAAT20NCG_P_5/Ar_TCTAAT20NCG_P_5_PCA.pdf]

tSNE Plot - Ar\_TCTAAT20NCG\_P\_5

Motif Sequence

- CCCCCCCACC
- CCCGCCCGCCCCA
- GGAACACCGTGTACC
- Random

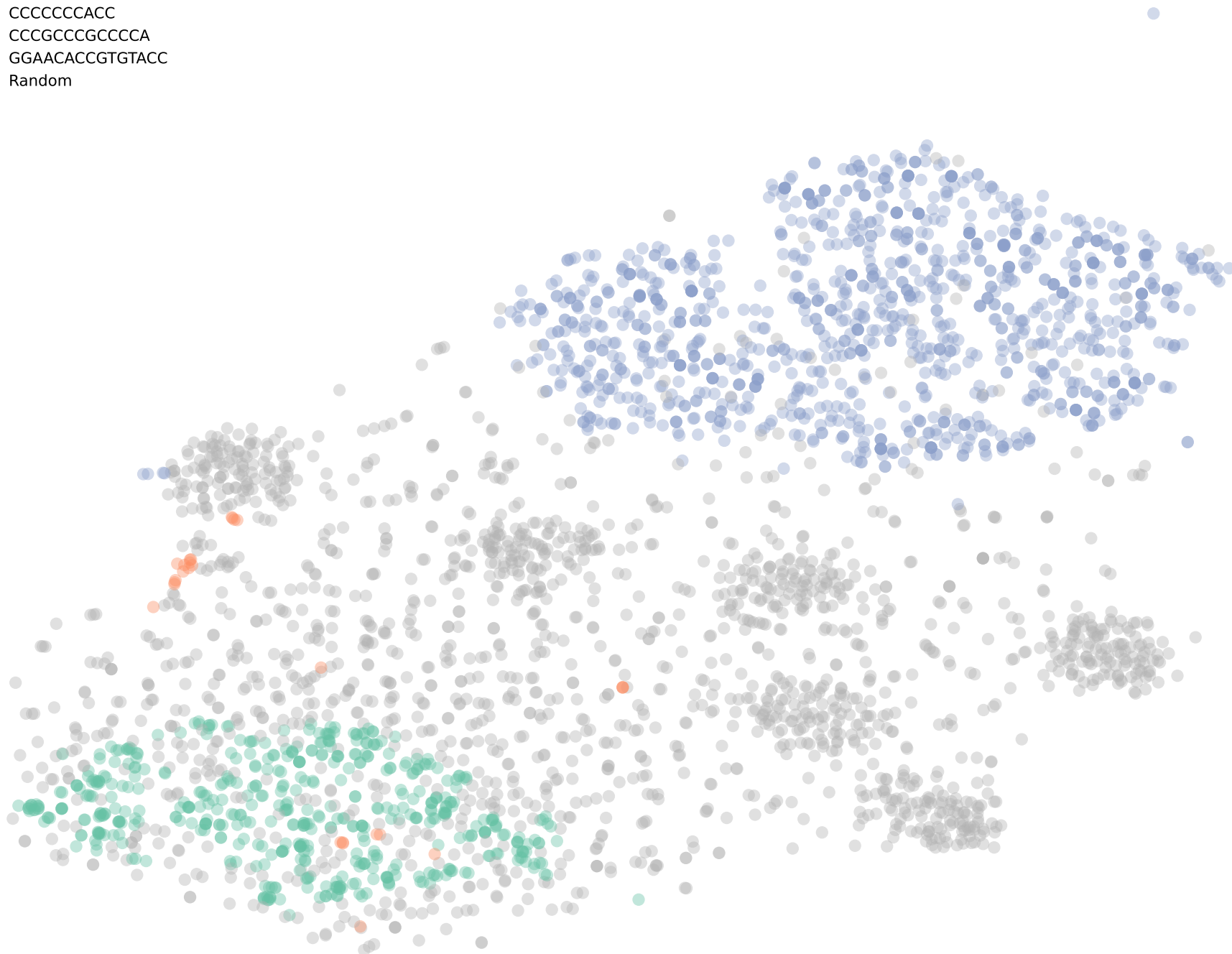

Supplement: Supplement 8 [file Supplemental_Data_1.zip › Supplemental_Data_1/Ar_TCTAAT20NCG_P_5/Ar_TCTAAT20NCG_P_5_tSNE.pdf]

UMAP Plot - Ar\_TCTAAT20NCG\_P\_5

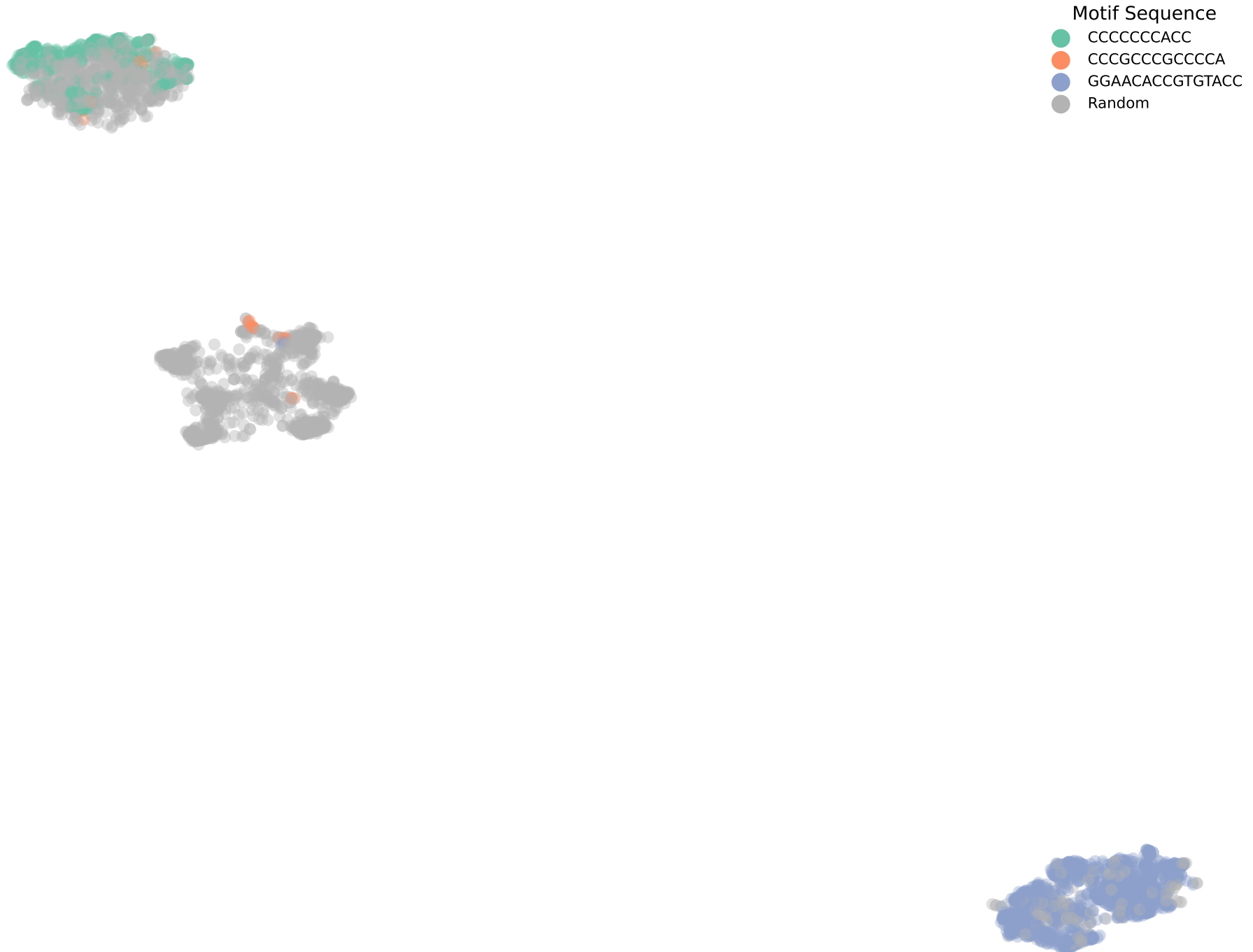

Supplement: Supplement 8 [file Supplemental_Data_1.zip › Supplemental_Data_1/Ar_TCTAAT20NCG_P_5/Ar_TCTAAT20NCG_P_5_UMAP.pdf]

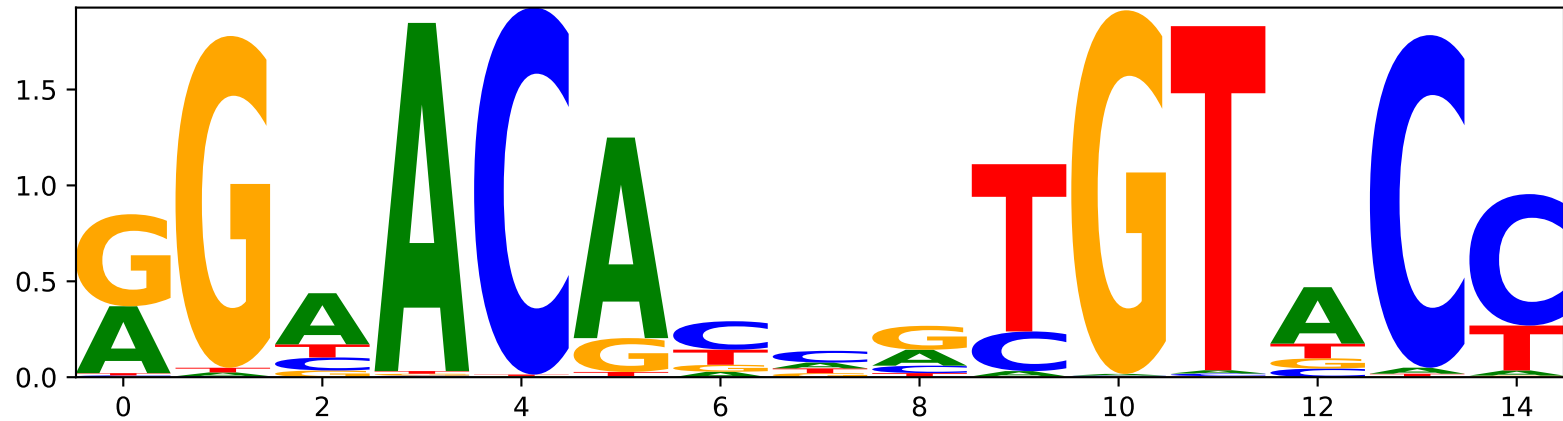

Supplement: Supplement 8 [file Supplemental_Data_1.zip › Supplemental_Data_1/Ar_TCTAAT20NCG_P_5/kmap_logo.pdf]

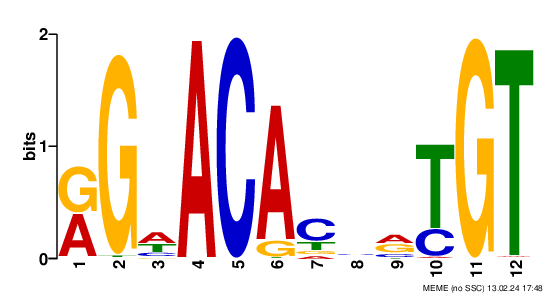

Supplement: Supplement 8 [file Supplemental_Data_1.zip › Supplemental_Data_1/Ar_TCTAAT20NCG_P_5/meme_logo.png]

KMAP LD Plot - AR\_TCTTCT20NCTG\_AD\_3

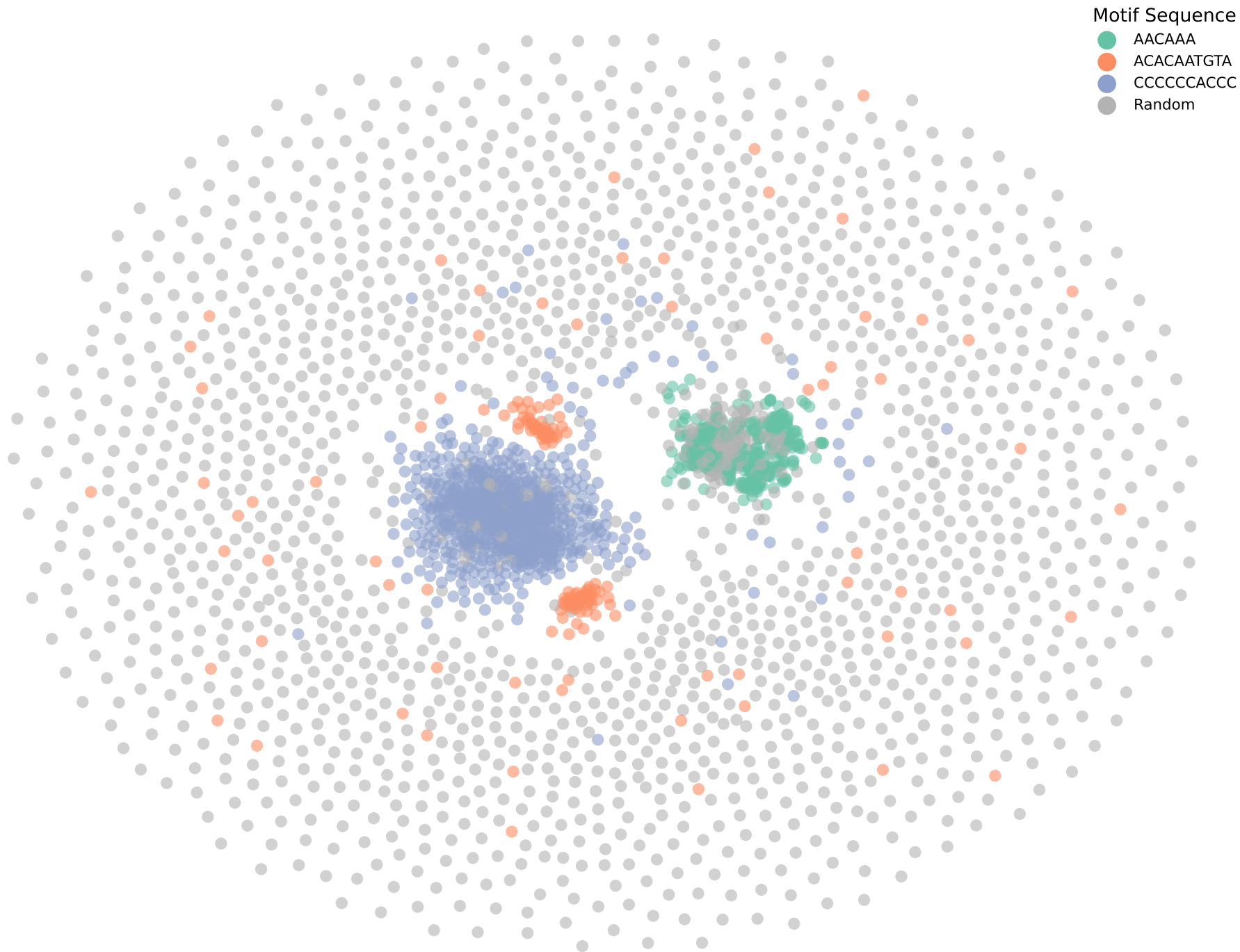

Supplement: Supplement 8 [file Supplemental_Data_1.zip › Supplemental_Data_1/AR_TCTTCT20NCTG_AD_3/AR_TCTTCT20NCTG_AD_3_KMAP.pdf]

MDS Plot - AR\_TCTTCT20NCTG\_AD\_3

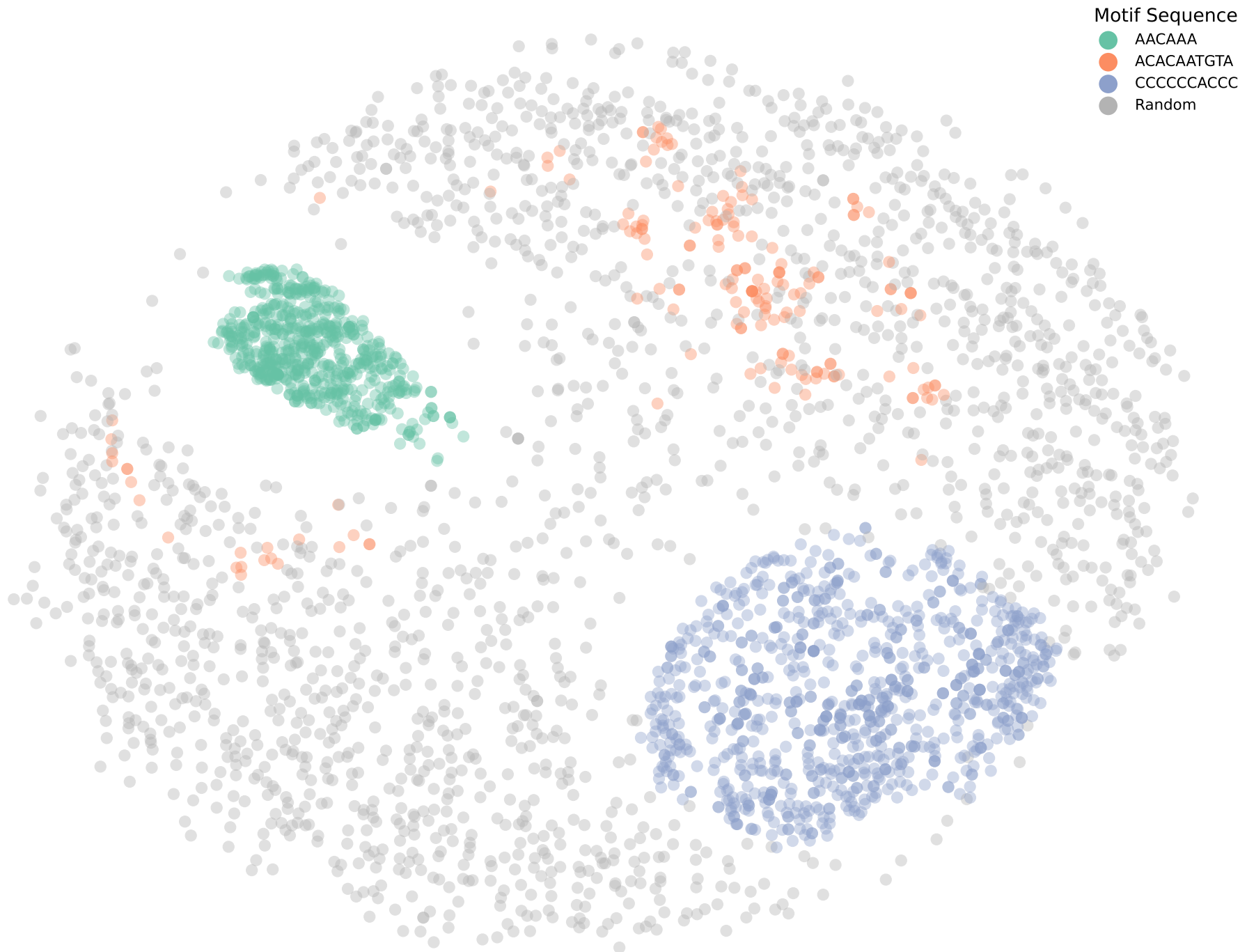

Supplement: Supplement 8 [file Supplemental_Data_1.zip › Supplemental_Data_1/AR_TCTTCT20NCTG_AD_3/AR_TCTTCT20NCTG_AD_3_MDS.pdf]

PCA Plot - AR\_TCTTCT20NCTG\_AD\_3

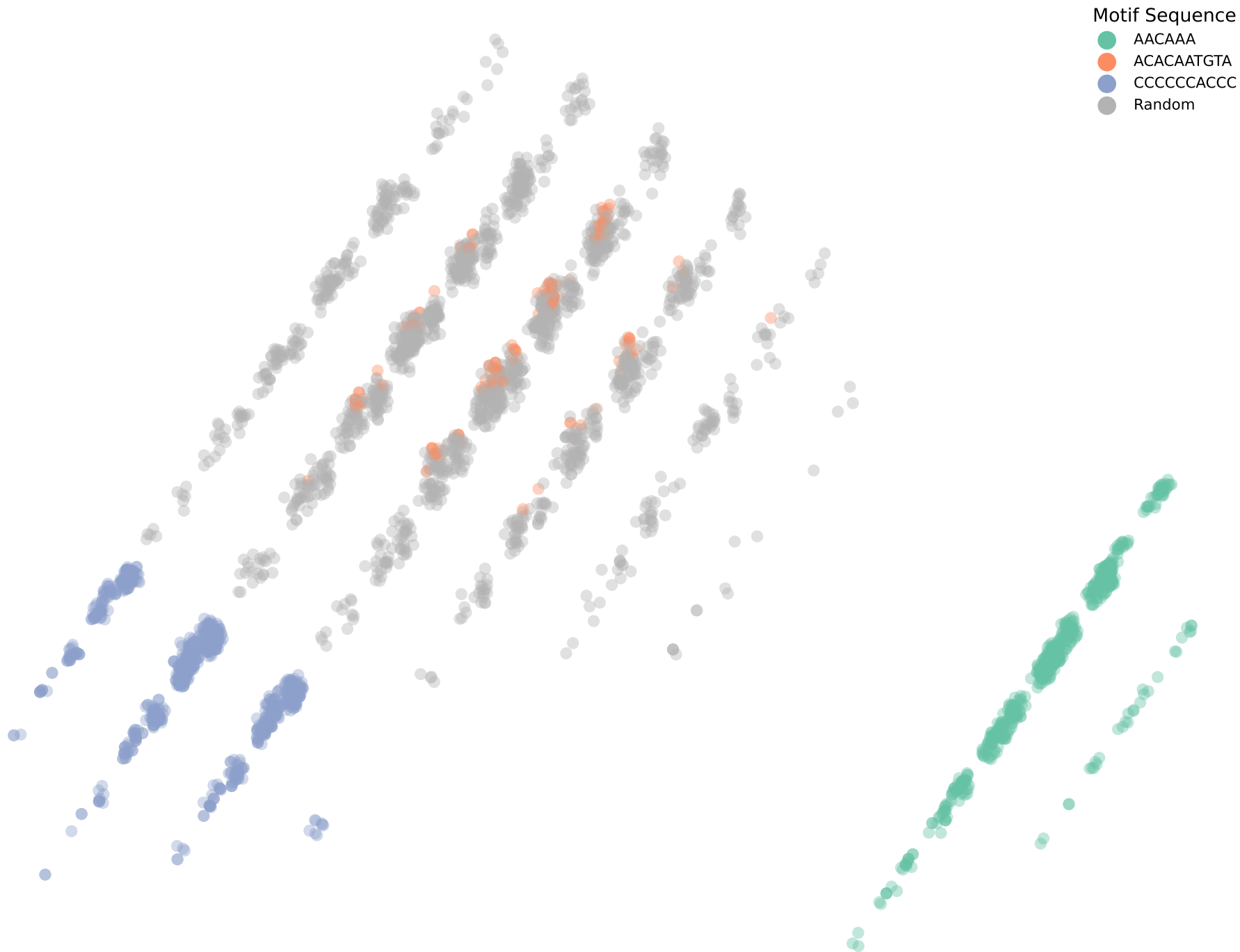

Supplement: Supplement 8 [file Supplemental_Data_1.zip › Supplemental_Data_1/AR_TCTTCT20NCTG_AD_3/AR_TCTTCT20NCTG_AD_3_PCA.pdf]

tSNE Plot - AR\_TCTTCT20NCTG\_AD\_3

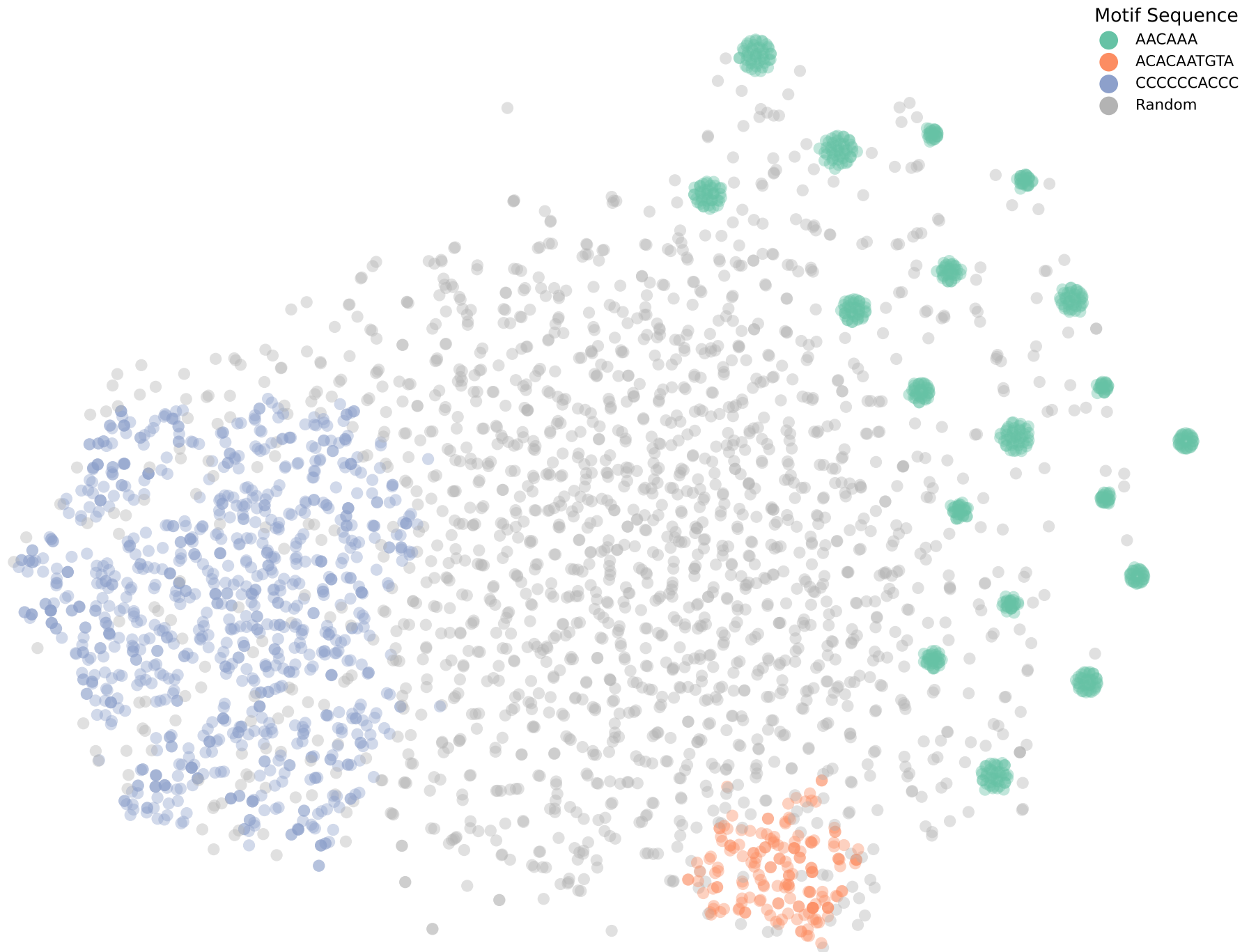

Supplement: Supplement 8 [file Supplemental_Data_1.zip › Supplemental_Data_1/AR_TCTTCT20NCTG_AD_3/AR_TCTTCT20NCTG_AD_3_tSNE.pdf]

UMAP Plot - AR\_TCTTCT20NCTG\_AD\_3

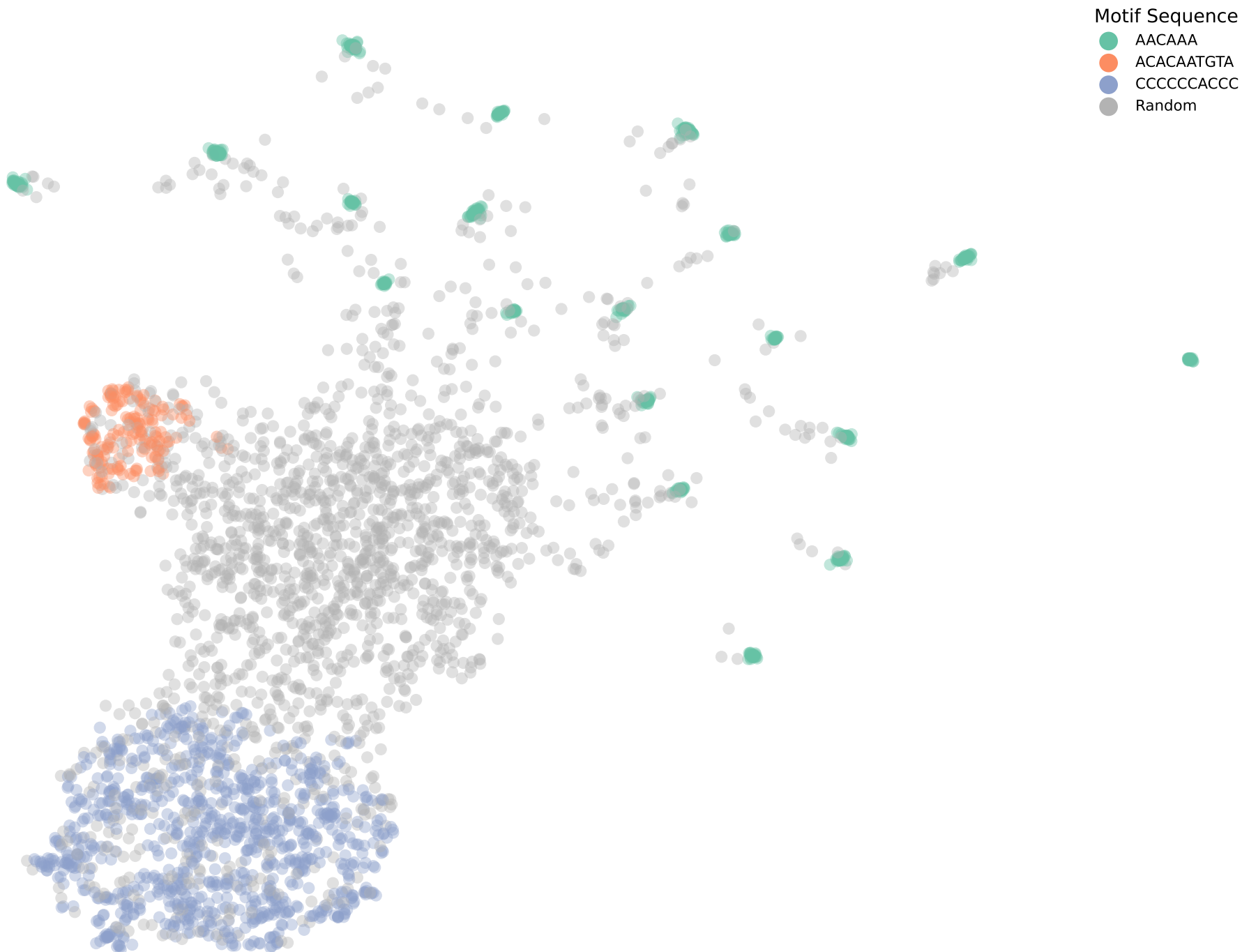

Supplement: Supplement 8 [file Supplemental_Data_1.zip › Supplemental_Data_1/AR_TCTTCT20NCTG_AD_3/AR_TCTTCT20NCTG_AD_3_UMAP.pdf]

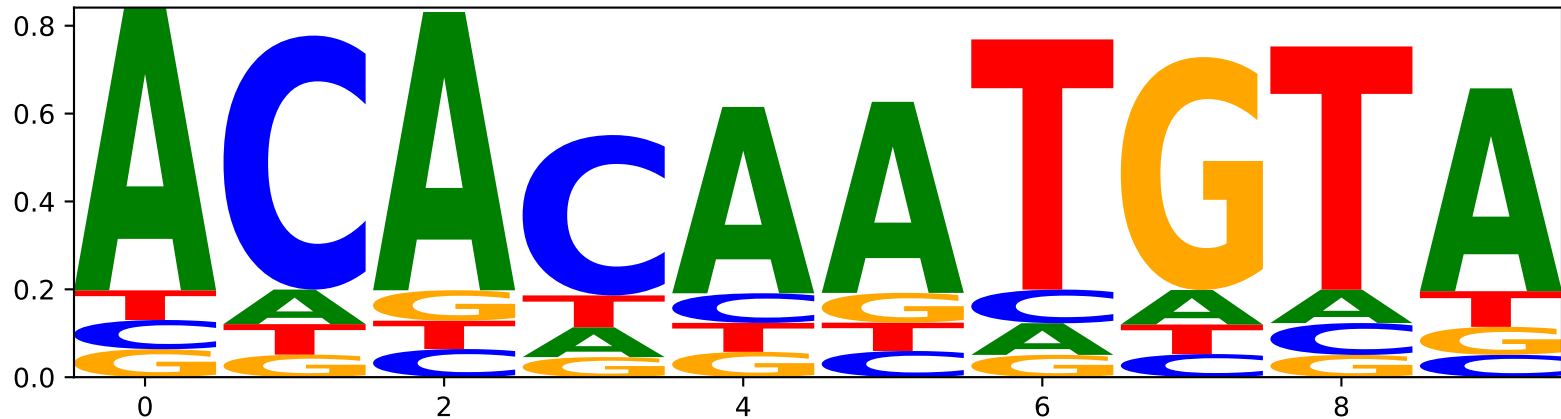

Supplement: Supplement 8 [file Supplemental_Data_1.zip › Supplemental_Data_1/AR_TCTTCT20NCTG_AD_3/kmap_logo.pdf]

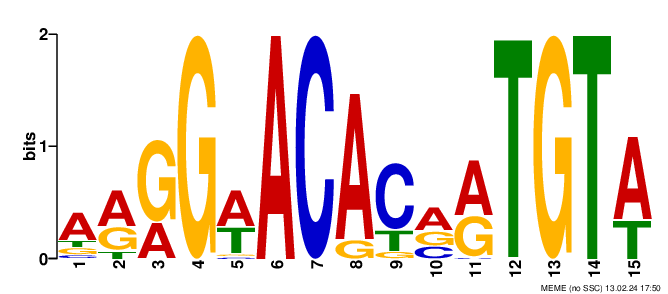

Supplement: Supplement 8 [file Supplemental_Data_1.zip › Supplemental_Data_1/AR_TCTTCT20NCTG_AD_3/meme_logo.png]

KMAP LD Plot - AR\_TCTTCT20NCTG\_AD\_4

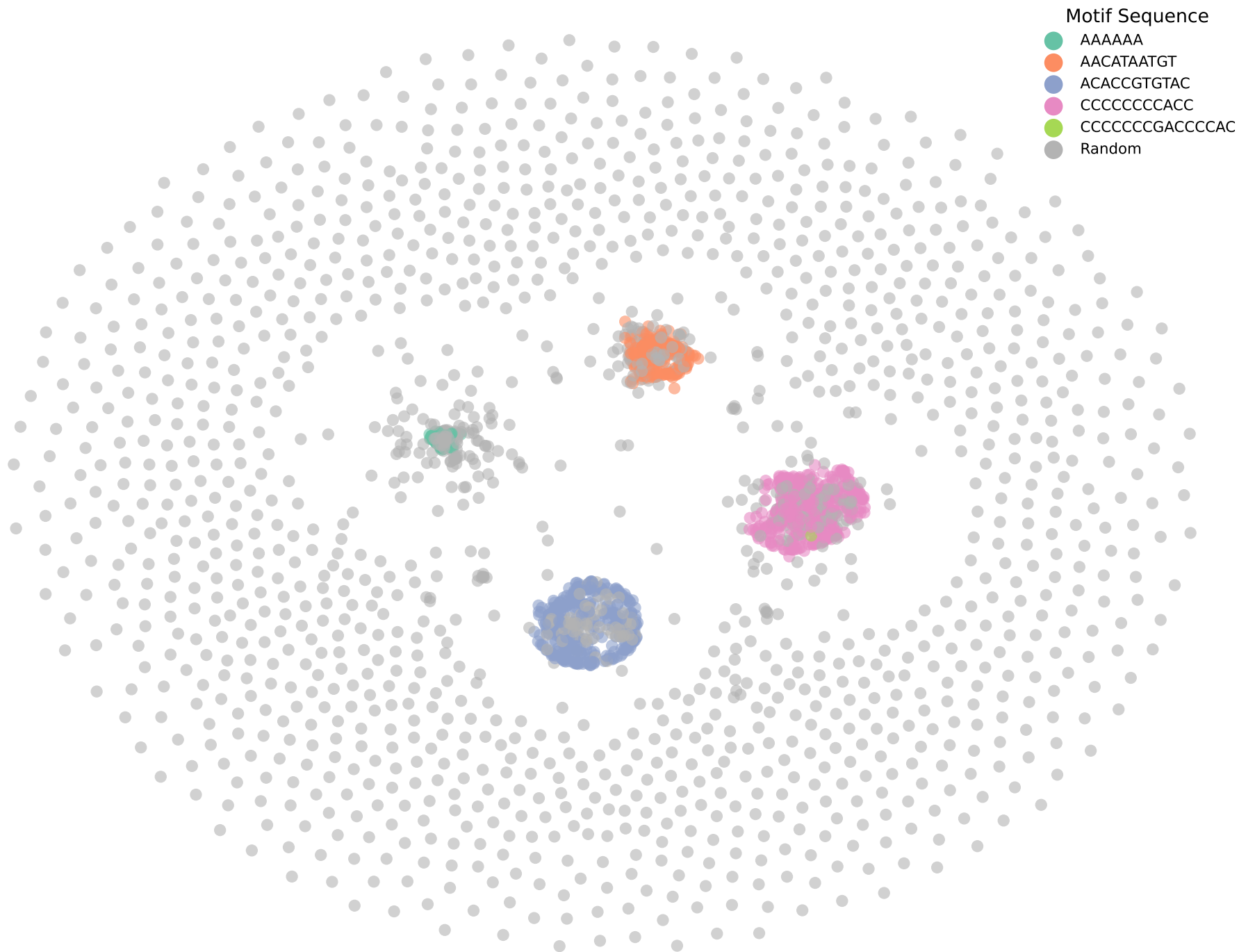

Supplement: Supplement 8 [file Supplemental_Data_1.zip › Supplemental_Data_1/AR_TCTTCT20NCTG_AD_4/AR_TCTTCT20NCTG_AD_4_KMAP.pdf]

MDS Plot - AR\_TCTTCT20NCTG\_AD\_4

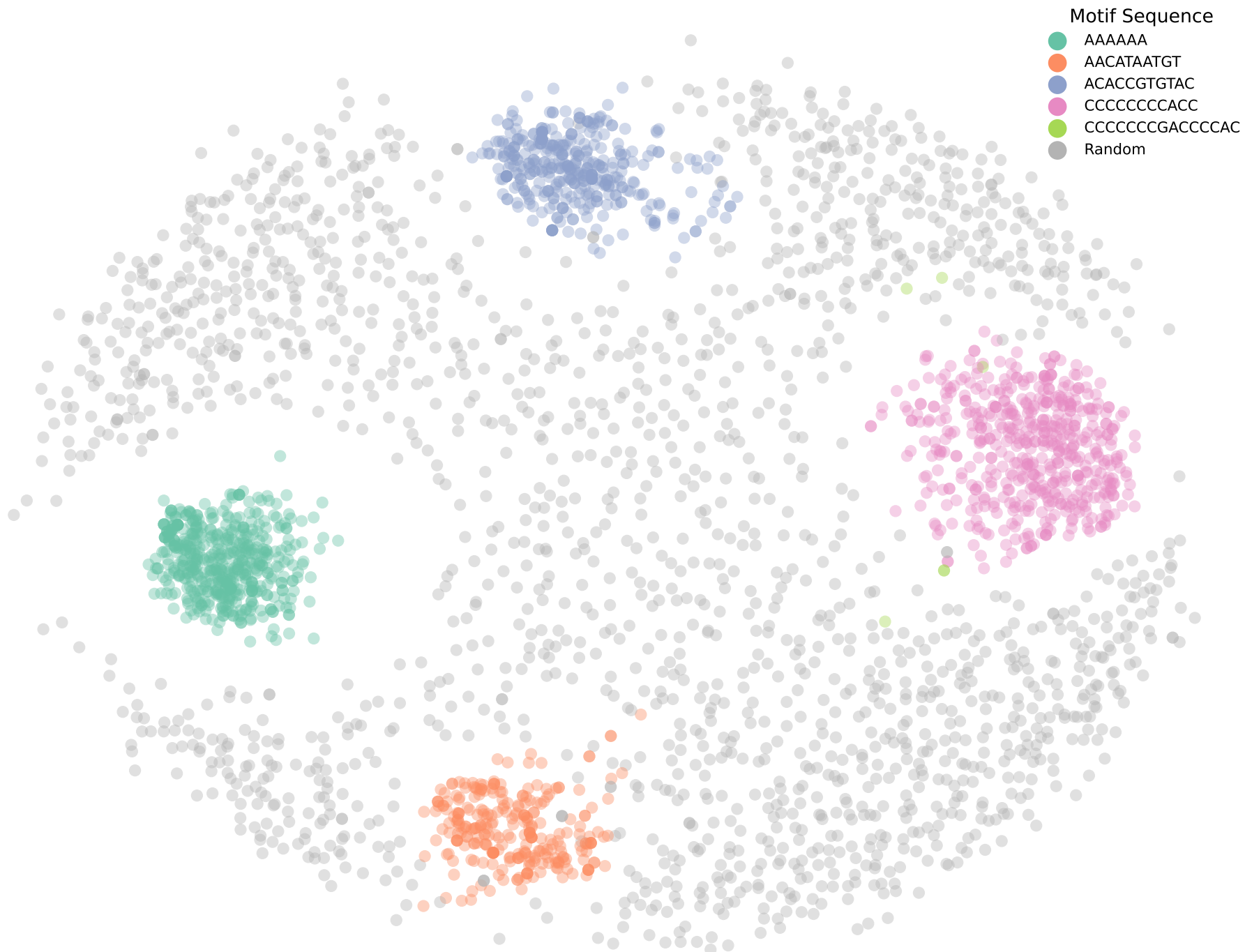

Supplement: Supplement 8 [file Supplemental_Data_1.zip › Supplemental_Data_1/AR_TCTTCT20NCTG_AD_4/AR_TCTTCT20NCTG_AD_4_MDS.pdf]

PCA Plot - AR\_TCTTCT20NCTG\_AD\_4

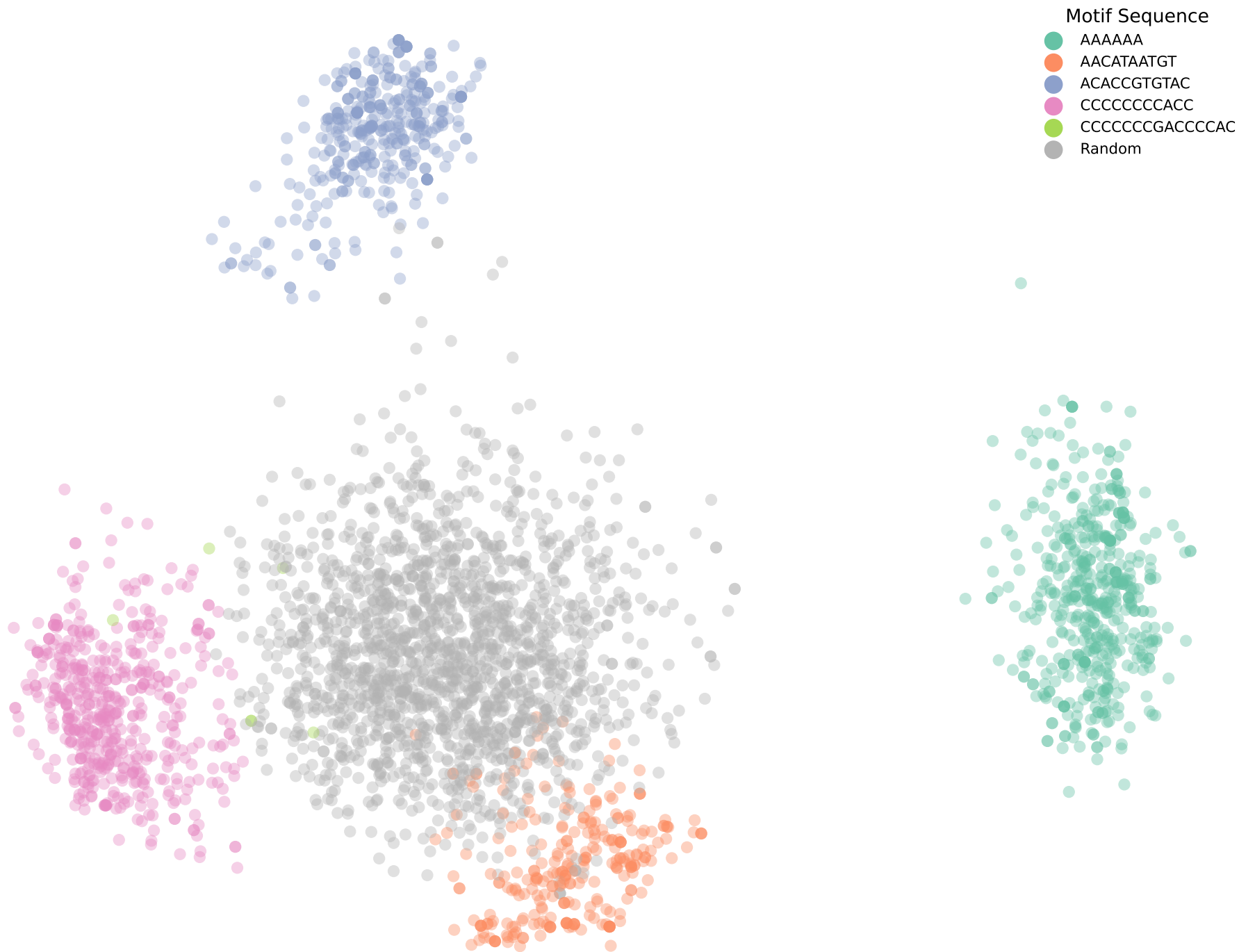

Supplement: Supplement 8 [file Supplemental_Data_1.zip › Supplemental_Data_1/AR_TCTTCT20NCTG_AD_4/AR_TCTTCT20NCTG_AD_4_PCA.pdf]

tSNE Plot - AR\_TCTTCT20NCTG\_AD\_4

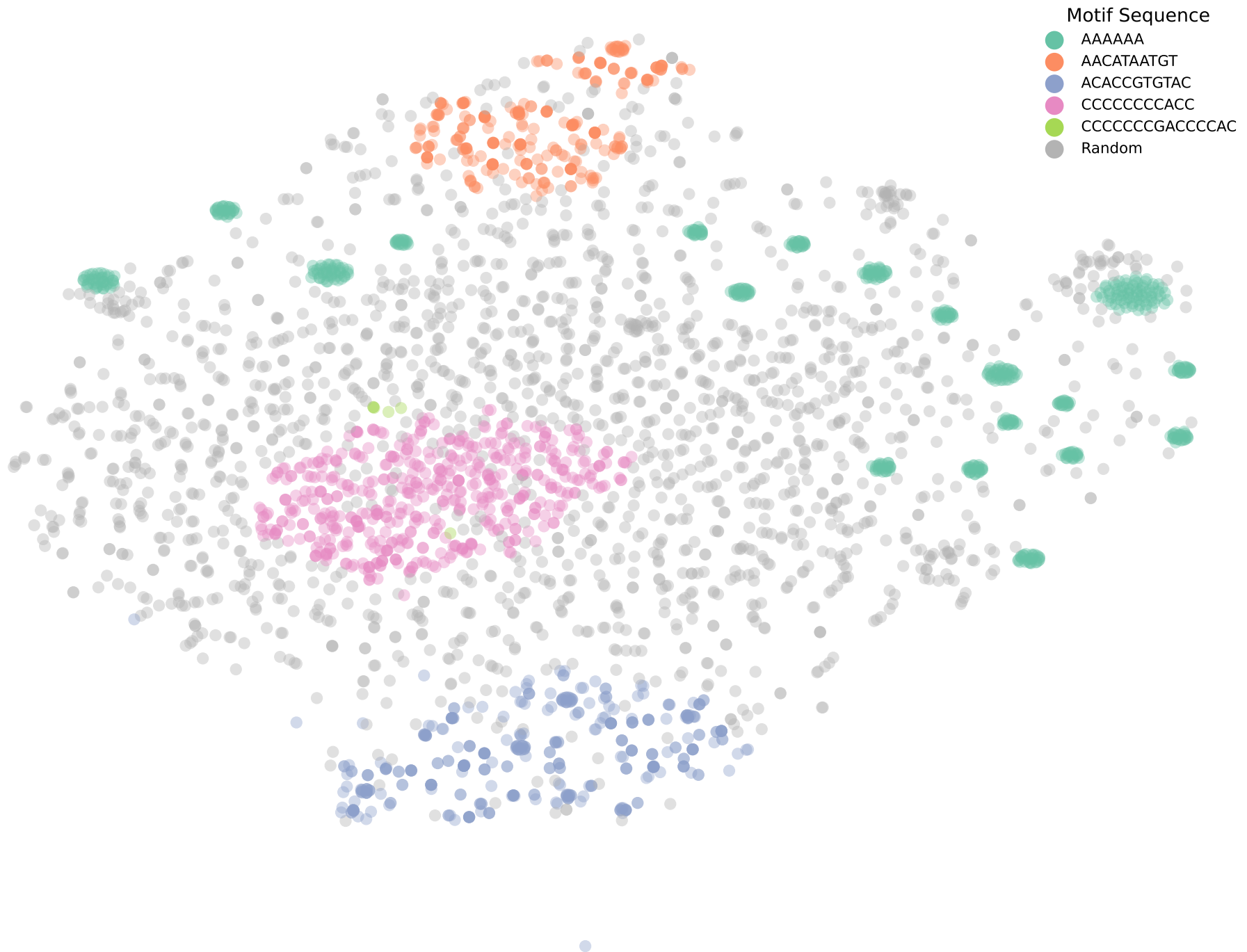

Supplement: Supplement 8 [file Supplemental_Data_1.zip › Supplemental_Data_1/AR_TCTTCT20NCTG_AD_4/AR_TCTTCT20NCTG_AD_4_tSNE.pdf]

UMAP Plot - AR\_TCTTCT20NCTG\_AD\_4

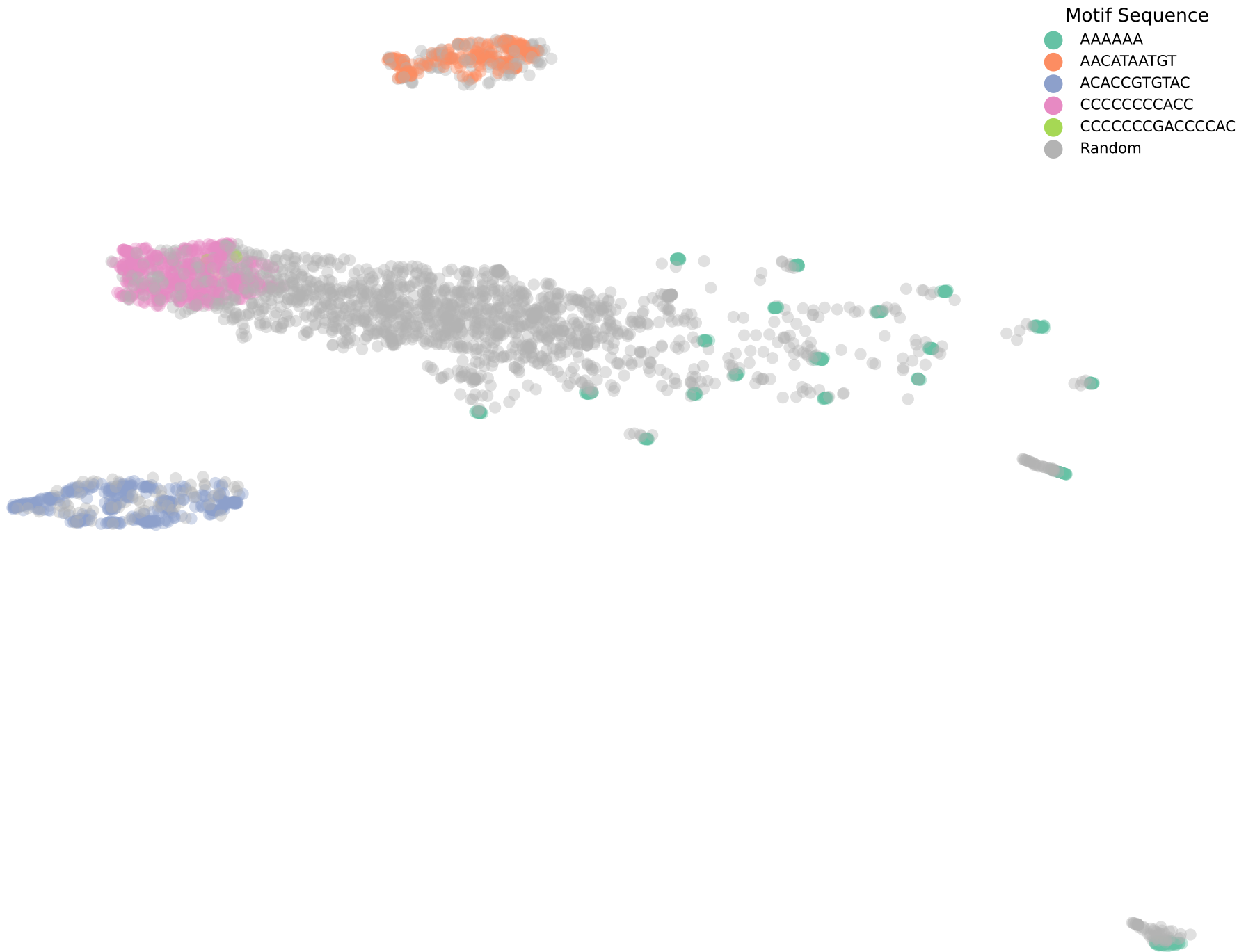

Supplement: Supplement 8 [file Supplemental_Data_1.zip › Supplemental_Data_1/AR_TCTTCT20NCTG_AD_4/AR_TCTTCT20NCTG_AD_4_UMAP.pdf]

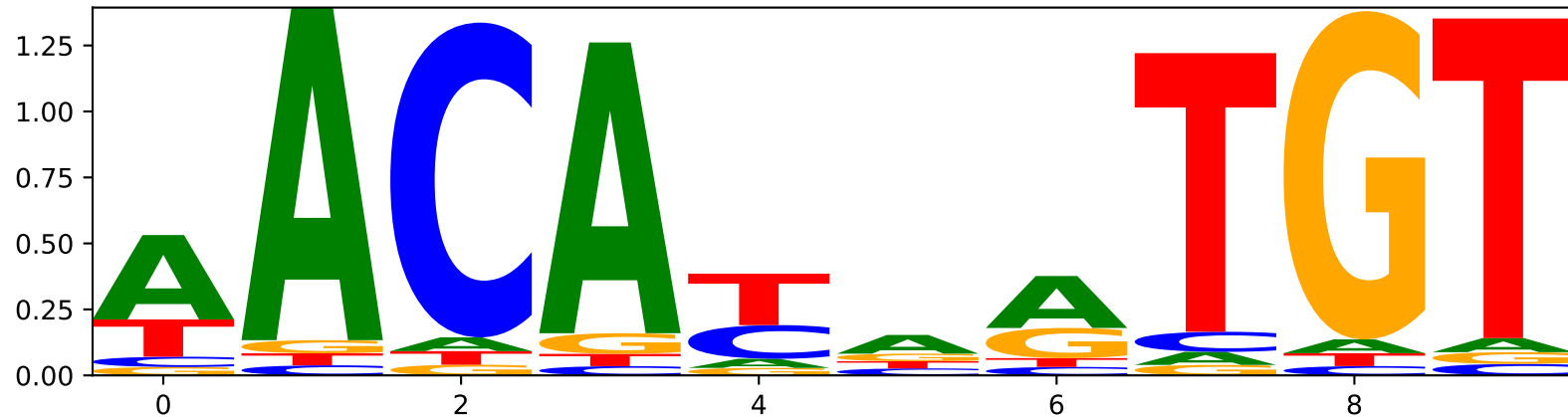

Supplement: Supplement 8 [file Supplemental_Data_1.zip › Supplemental_Data_1/AR_TCTTCT20NCTG_AD_4/kmap_logo.pdf]

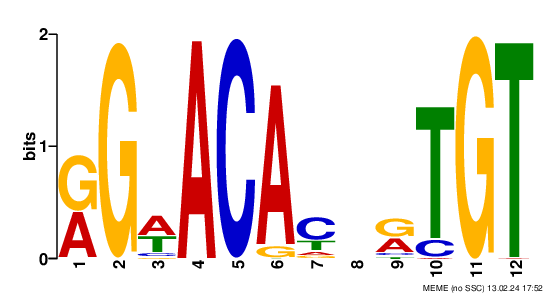

Supplement: Supplement 8 [file Supplemental_Data_1.zip › Supplemental_Data_1/AR_TCTTCT20NCTG_AD_4/meme_logo.png]

KMAP LD Plot - AR\_TGCTCG20NGA\_AF\_3

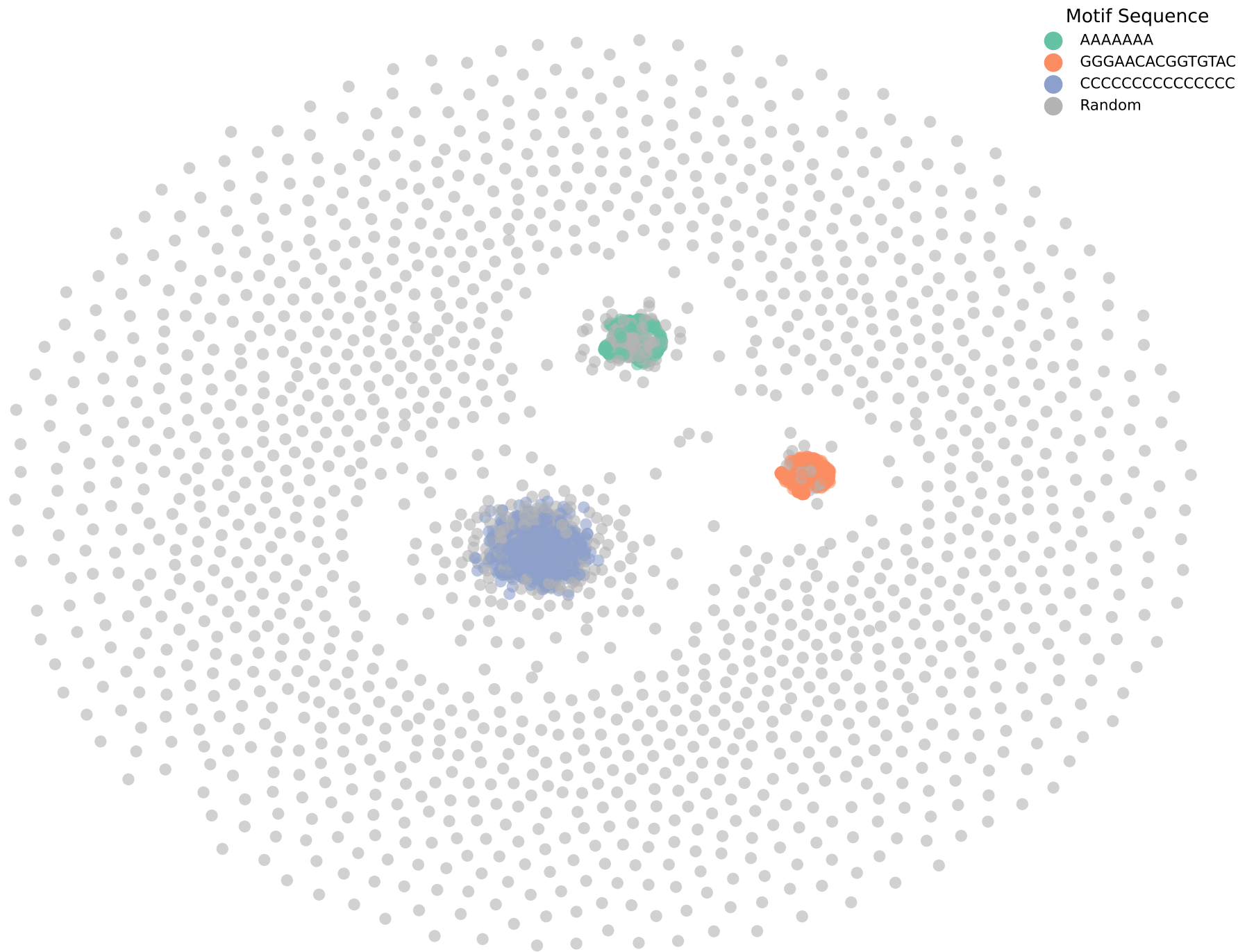

Supplement: Supplement 8 [file Supplemental_Data_1.zip › Supplemental_Data_1/AR_TGCTCG20NGA_AF_3/AR_TGCTCG20NGA_AF_3_KMAP.pdf]

MDS Plot - AR\_TGCTCG20NGA\_AF\_3

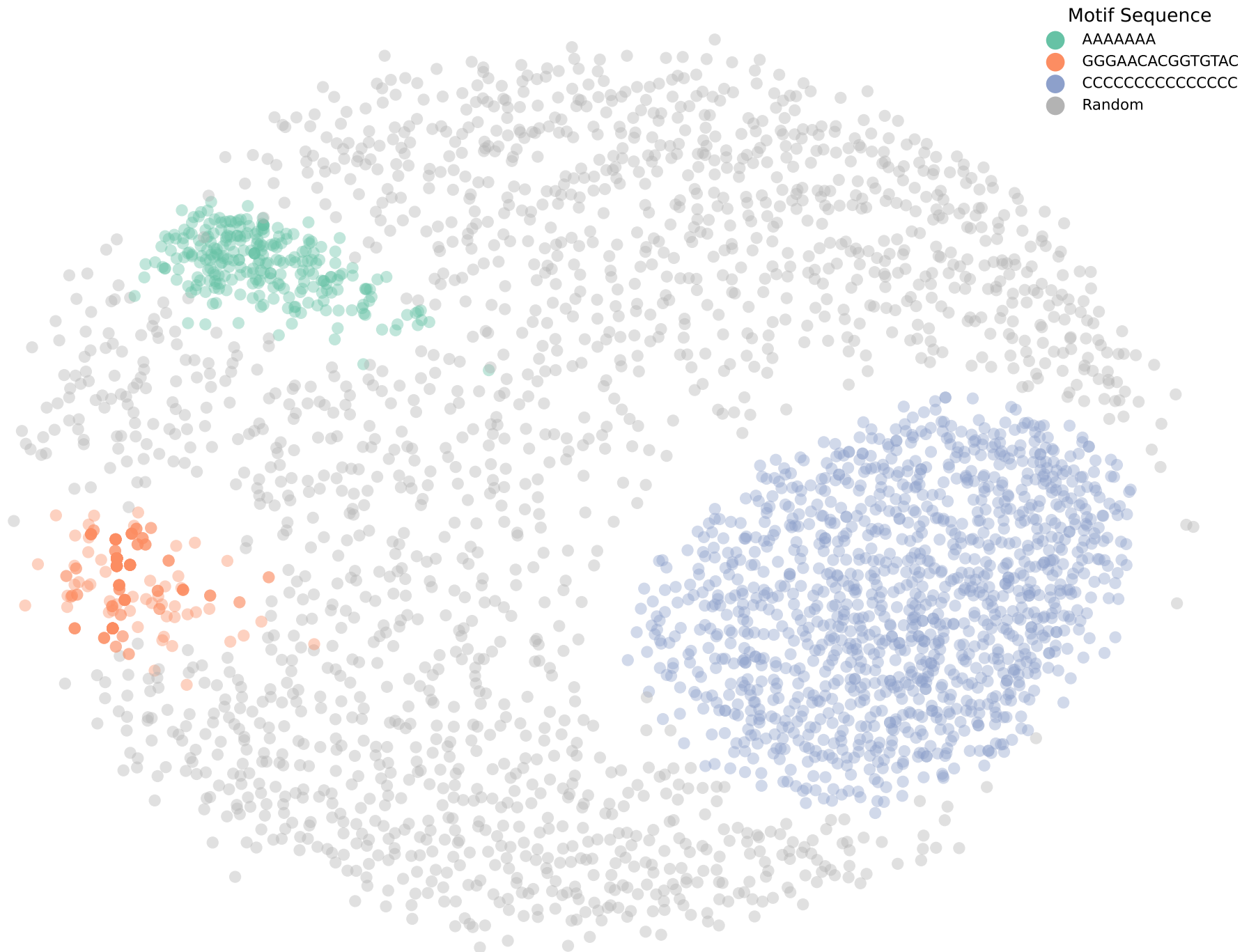

Supplement: Supplement 8 [file Supplemental_Data_1.zip › Supplemental_Data_1/AR_TGCTCG20NGA_AF_3/AR_TGCTCG20NGA_AF_3_MDS.pdf]

PCA Plot - AR\_TGCTCG20NGA\_AF\_3

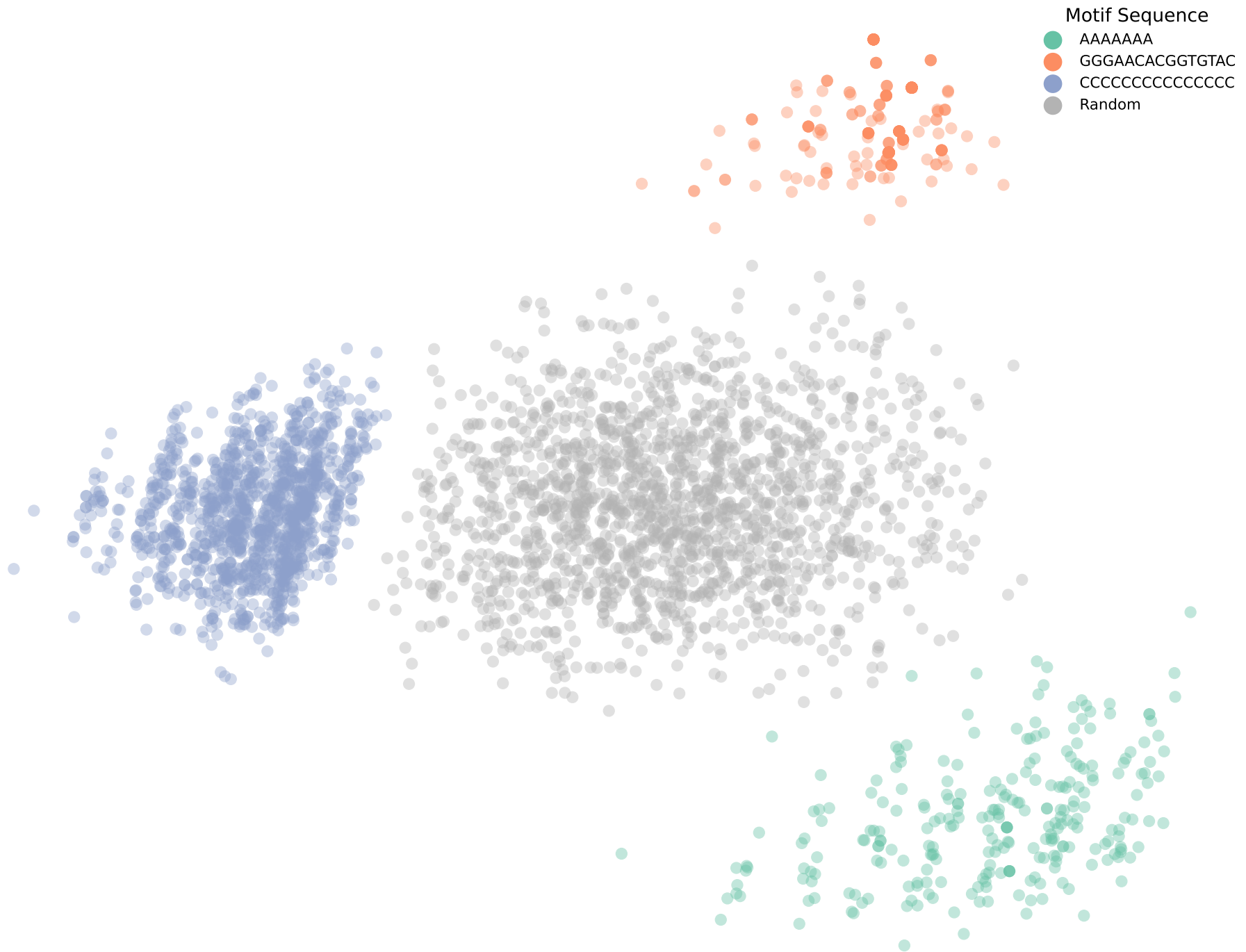

Supplement: Supplement 8 [file Supplemental_Data_1.zip › Supplemental_Data_1/AR_TGCTCG20NGA_AF_3/AR_TGCTCG20NGA_AF_3_PCA.pdf]

tSNE Plot - AR\_TGCTCG20NGA\_AF\_3

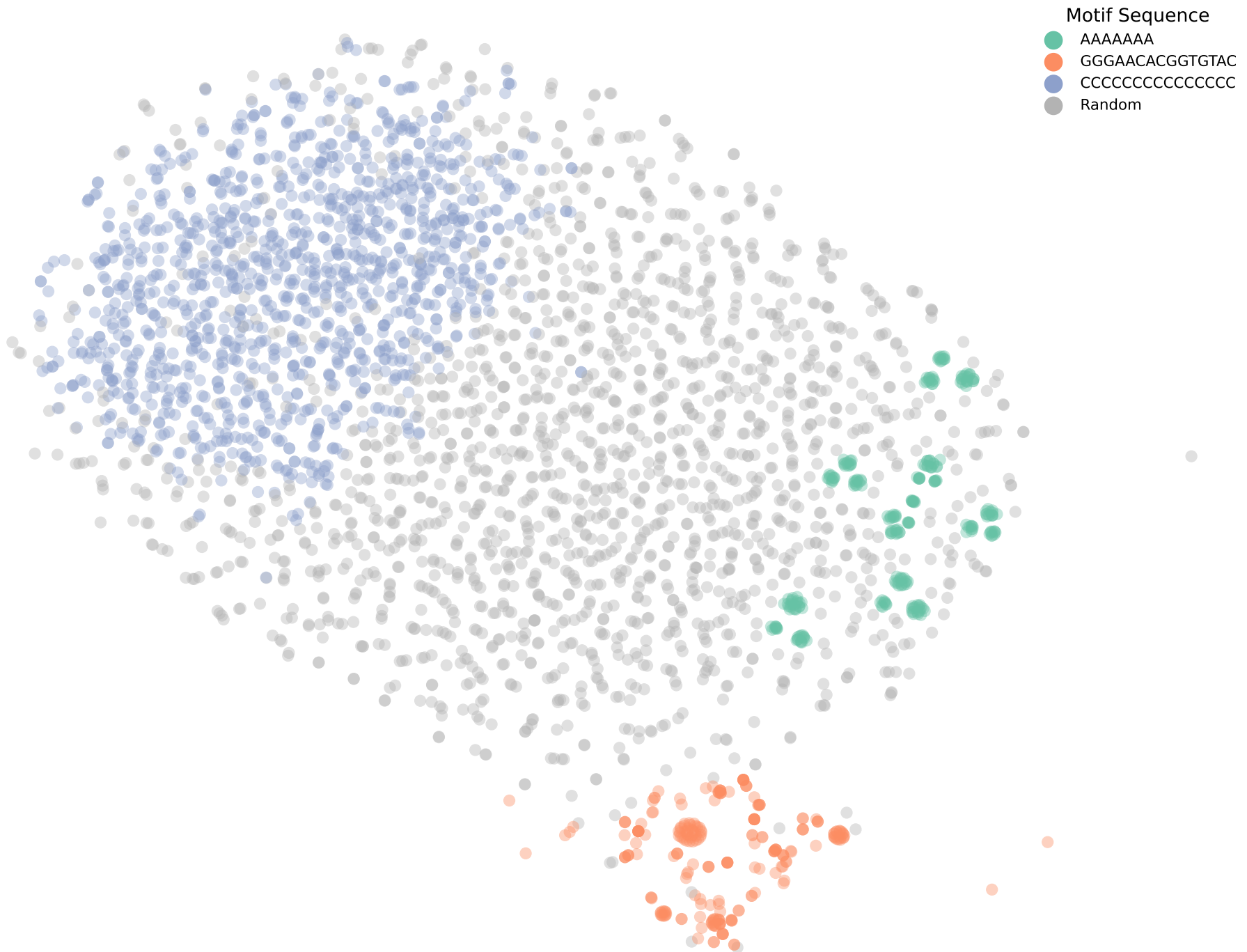

Supplement: Supplement 8 [file Supplemental_Data_1.zip › Supplemental_Data_1/AR_TGCTCG20NGA_AF_3/AR_TGCTCG20NGA_AF_3_tSNE.pdf]

UMAP Plot - AR\_TGCTCG20NGA\_AF\_3

Motif Sequence

- AAAAAAA
- GGGAACACGGTGTAC
- CCCCCCCCCCCCCCC
- Random

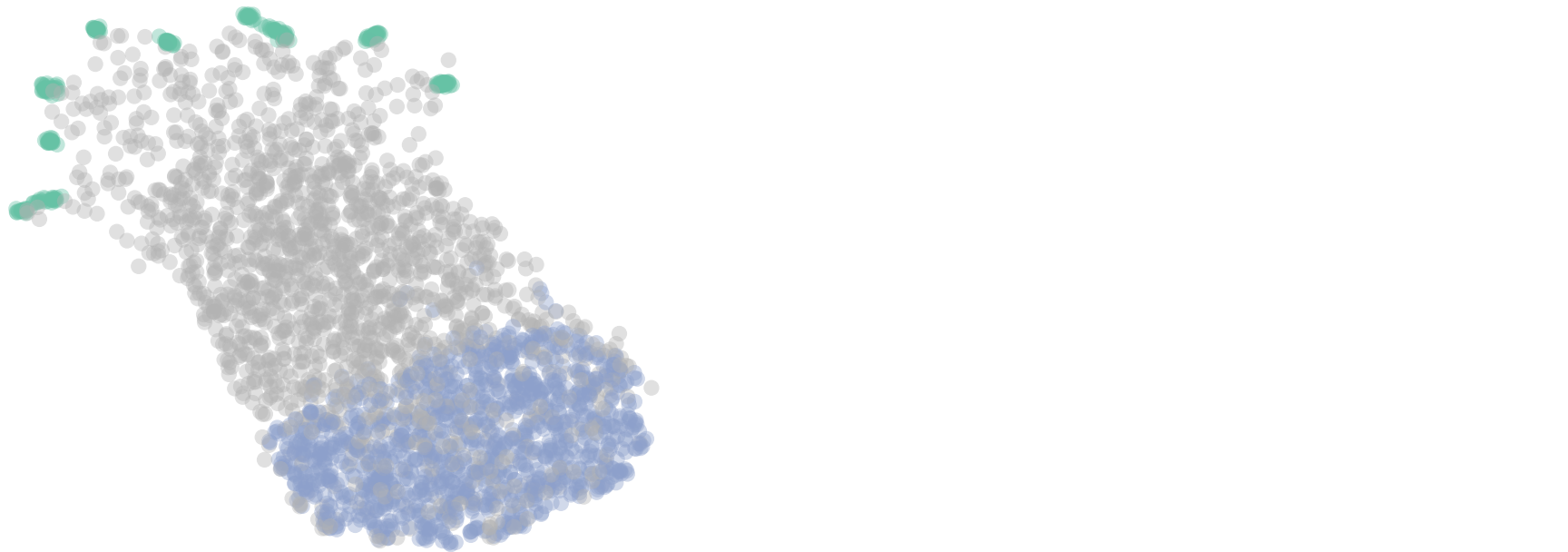

Supplement: Supplement 8 [file Supplemental_Data_1.zip › Supplemental_Data_1/AR_TGCTCG20NGA_AF_3/AR_TGCTCG20NGA_AF_3_UMAP.pdf]

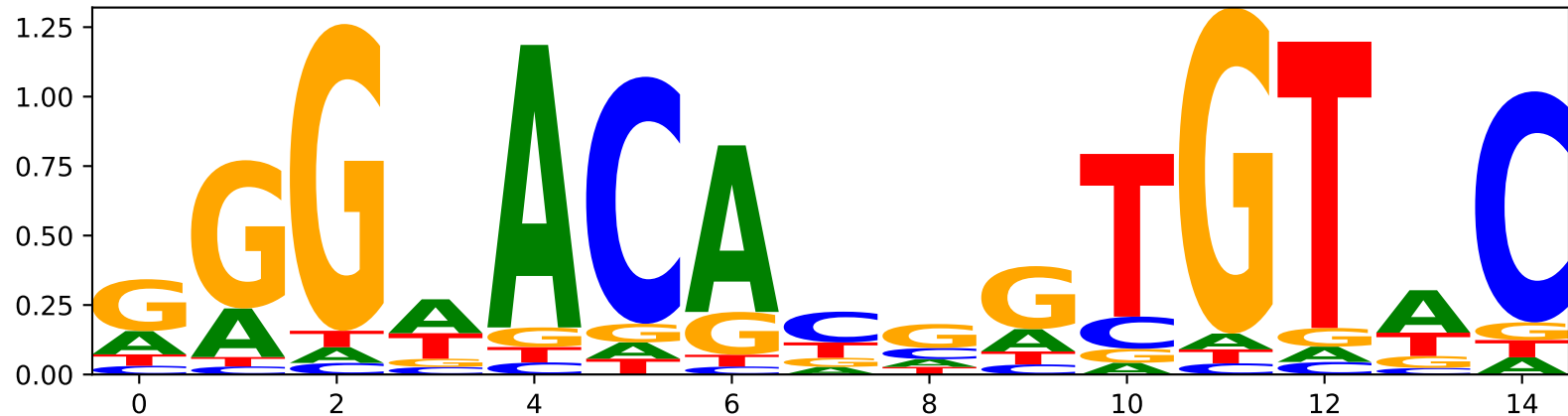

Supplement: Supplement 8 [file Supplemental_Data_1.zip › Supplemental_Data_1/AR_TGCTCG20NGA_AF_3/kmap_logo.pdf]

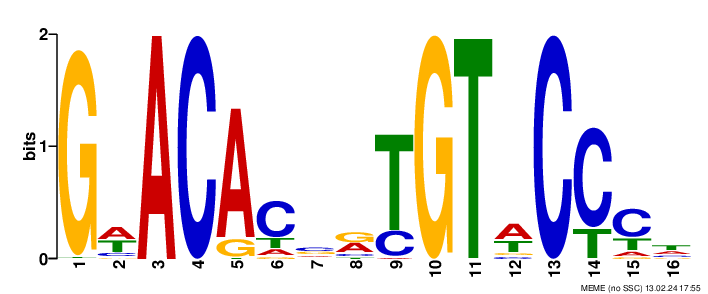

Supplement: Supplement 8 [file Supplemental_Data_1.zip › Supplemental_Data_1/AR_TGCTCG20NGA_AF_3/meme_logo.png]

KMAP LD Plot - AR\_TGCTCG20NGA\_AF\_4

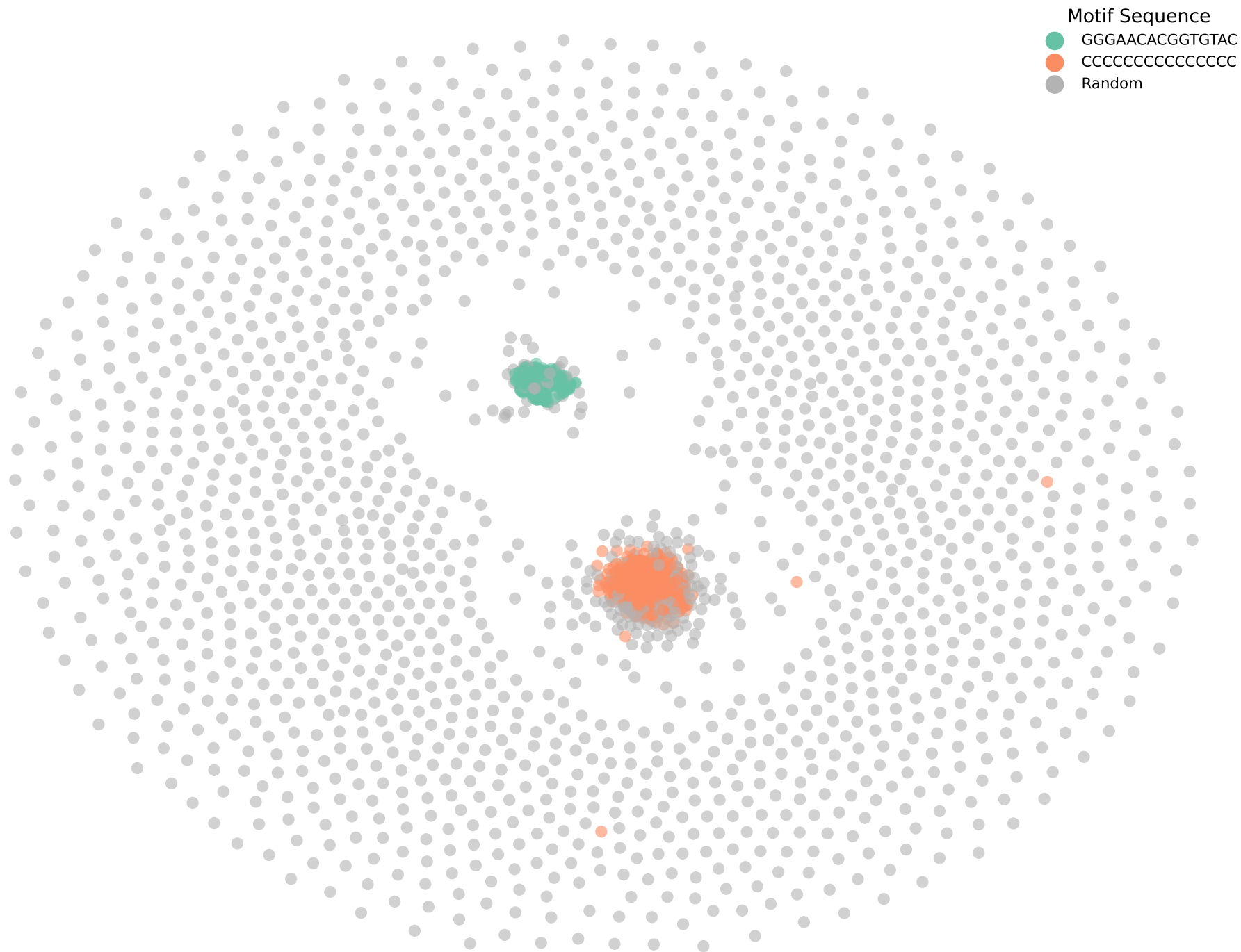

Supplement: Supplement 8 [file Supplemental_Data_1.zip › Supplemental_Data_1/AR_TGCTCG20NGA_AF_4/AR_TGCTCG20NGA_AF_4_KMAP.pdf]

MDS Plot - AR\_TGCTCG20NGA\_AF\_4

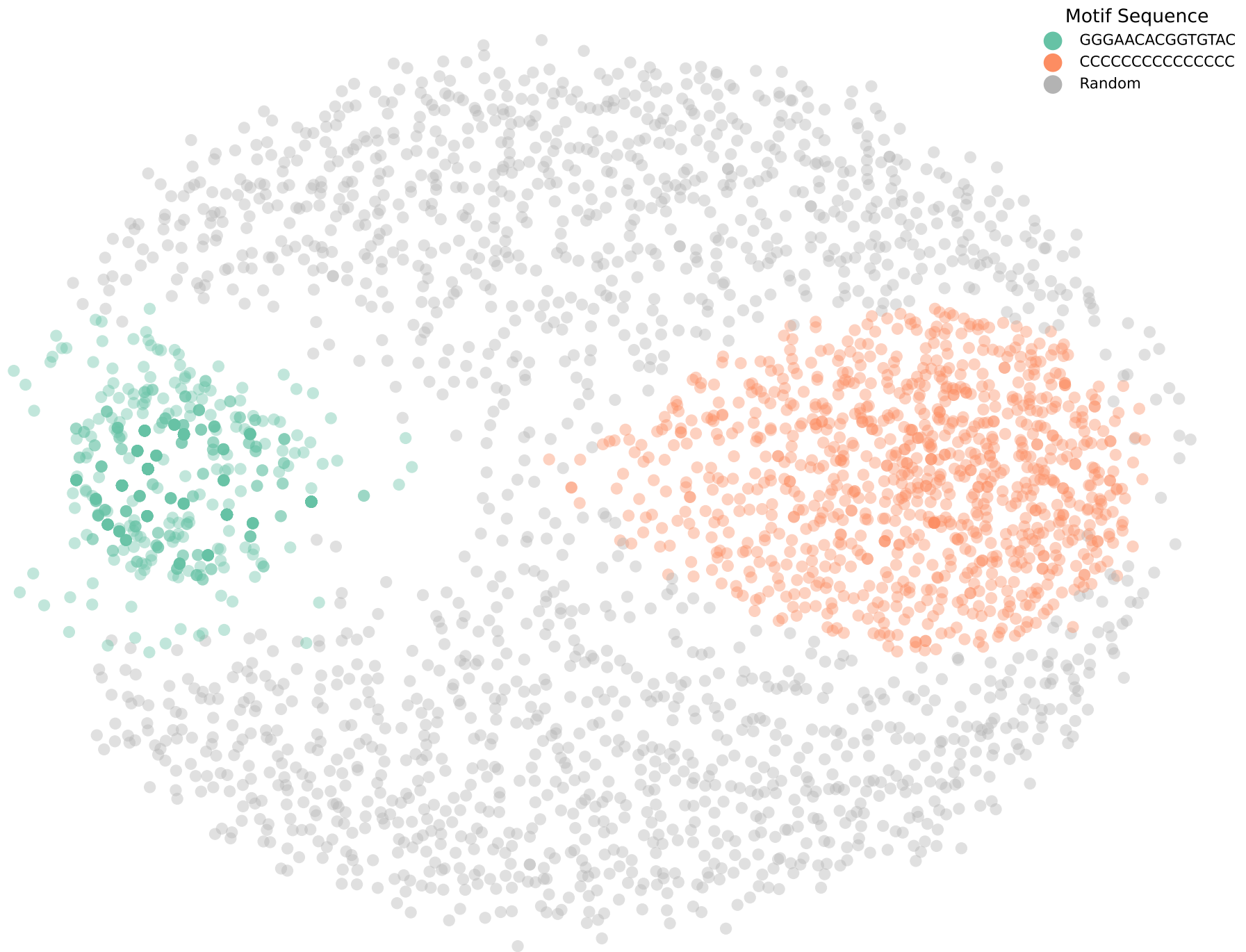

Supplement: Supplement 8 [file Supplemental_Data_1.zip › Supplemental_Data_1/AR_TGCTCG20NGA_AF_4/AR_TGCTCG20NGA_AF_4_MDS.pdf]

PCA Plot - AR\_TGCTCG20NGA\_AF\_4

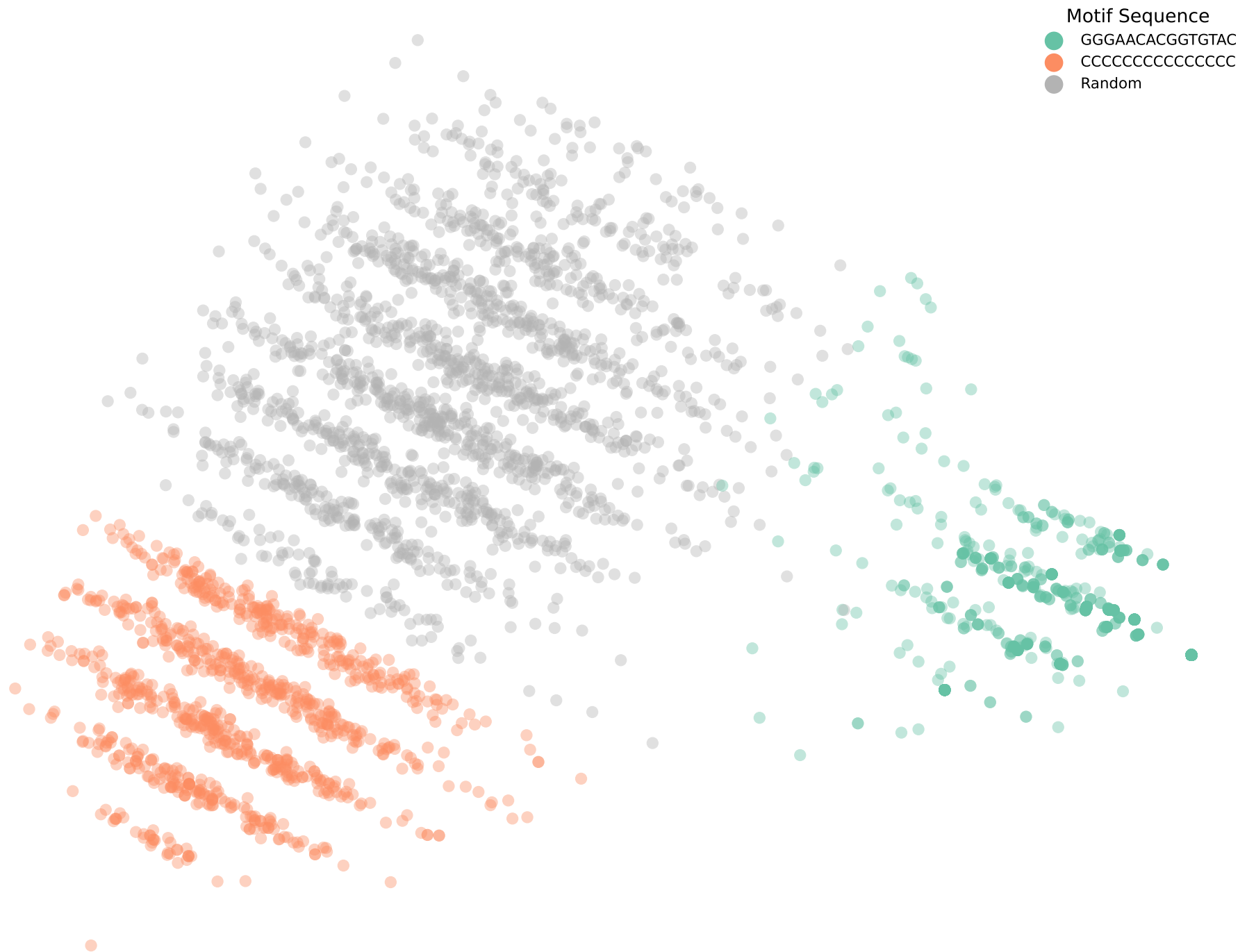

Supplement: Supplement 8 [file Supplemental_Data_1.zip › Supplemental_Data_1/AR_TGCTCG20NGA_AF_4/AR_TGCTCG20NGA_AF_4_PCA.pdf]

tSNE Plot - AR\_TGCTCG20NGA\_AF\_4

Motif Sequence

- GGGAACACGGTGTAC
- CCCCCCCCCCCCCCC
- Random

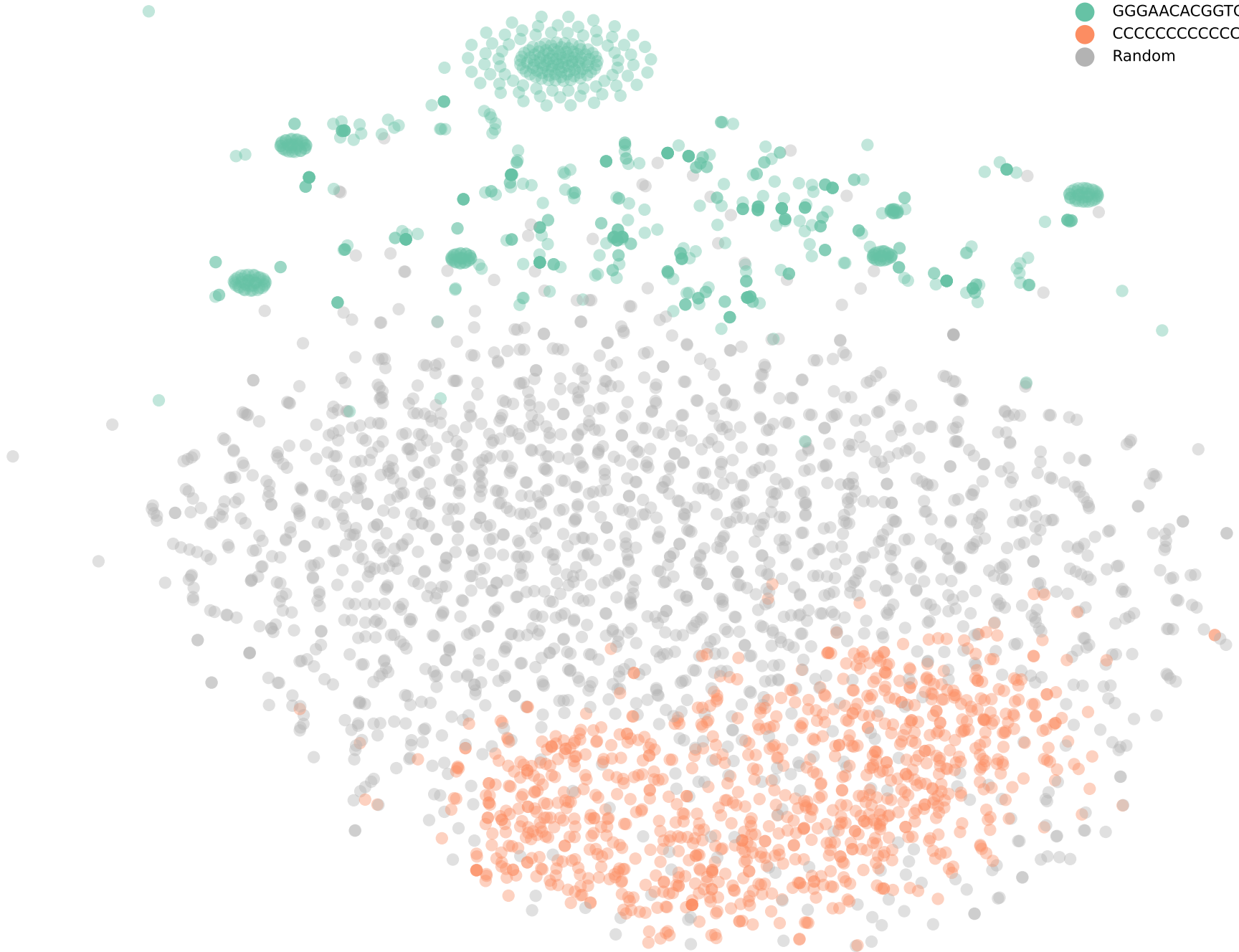

Supplement: Supplement 8 [file Supplemental_Data_1.zip › Supplemental_Data_1/AR_TGCTCG20NGA_AF_4/AR_TGCTCG20NGA_AF_4_tSNE.pdf]

UMAP Plot - AR\_TGCTCG20NGA\_AF\_4

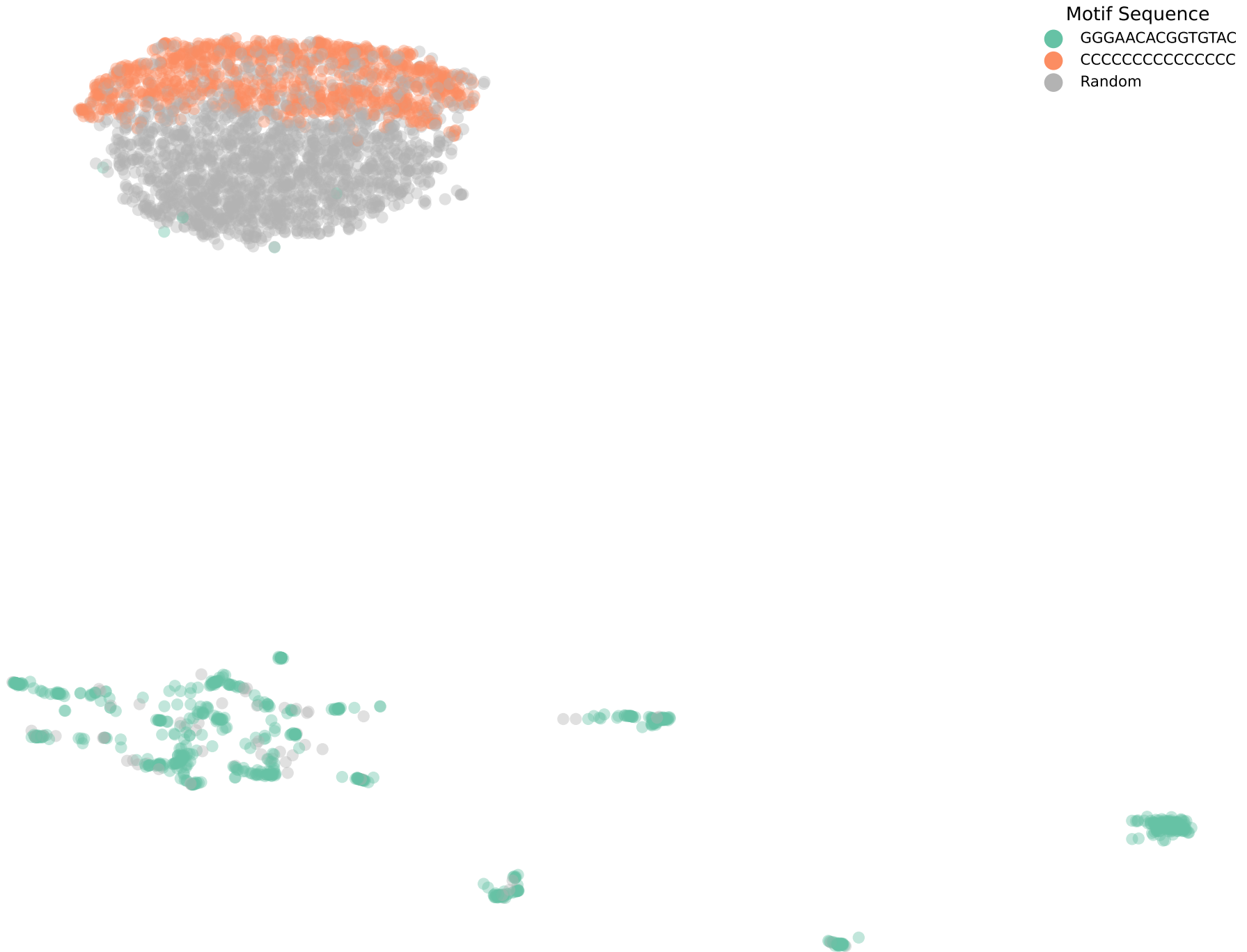

Supplement: Supplement 8 [file Supplemental_Data_1.zip › Supplemental_Data_1/AR_TGCTCG20NGA_AF_4/AR_TGCTCG20NGA_AF_4_UMAP.pdf]

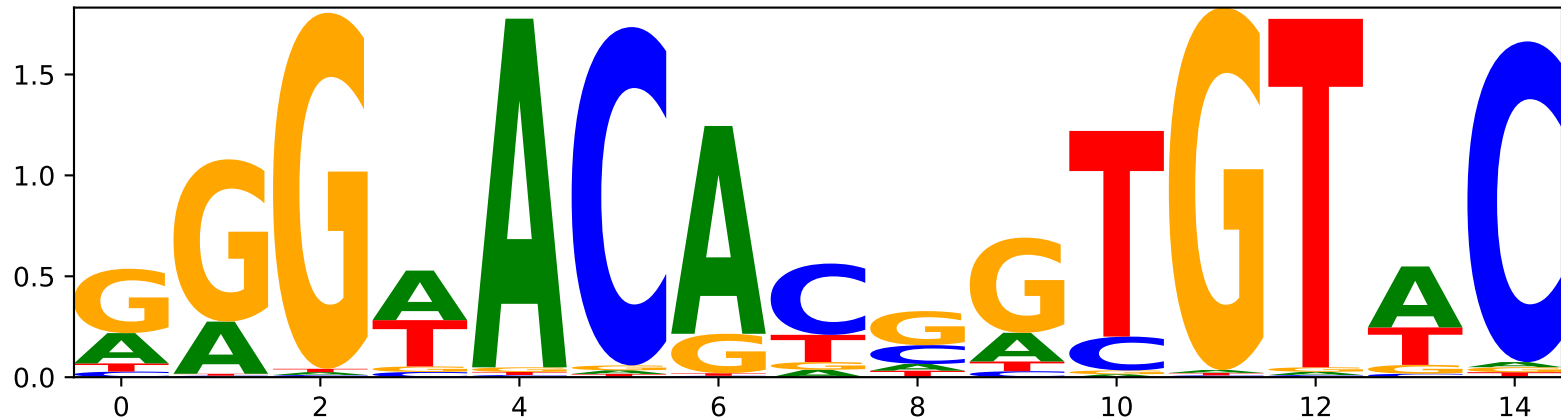

Supplement: Supplement 8 [file Supplemental_Data_1.zip › Supplemental_Data_1/AR_TGCTCG20NGA_AF_4/kmap_logo.pdf]

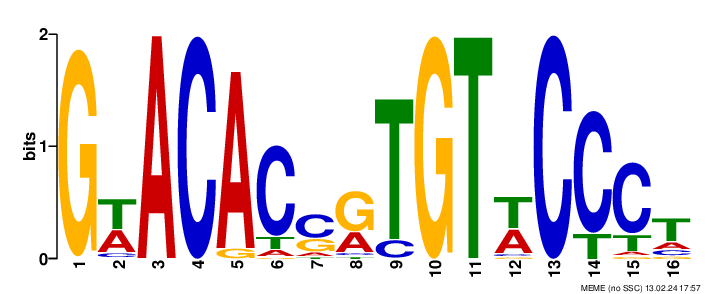

Supplement: Supplement 8 [file Supplemental_Data_1.zip › Supplemental_Data_1/AR_TGCTCG20NGA_AF_4/meme_logo.png]

KMAP LD Plot - AscI2\_TAGGGC20NCG\_Z\_3

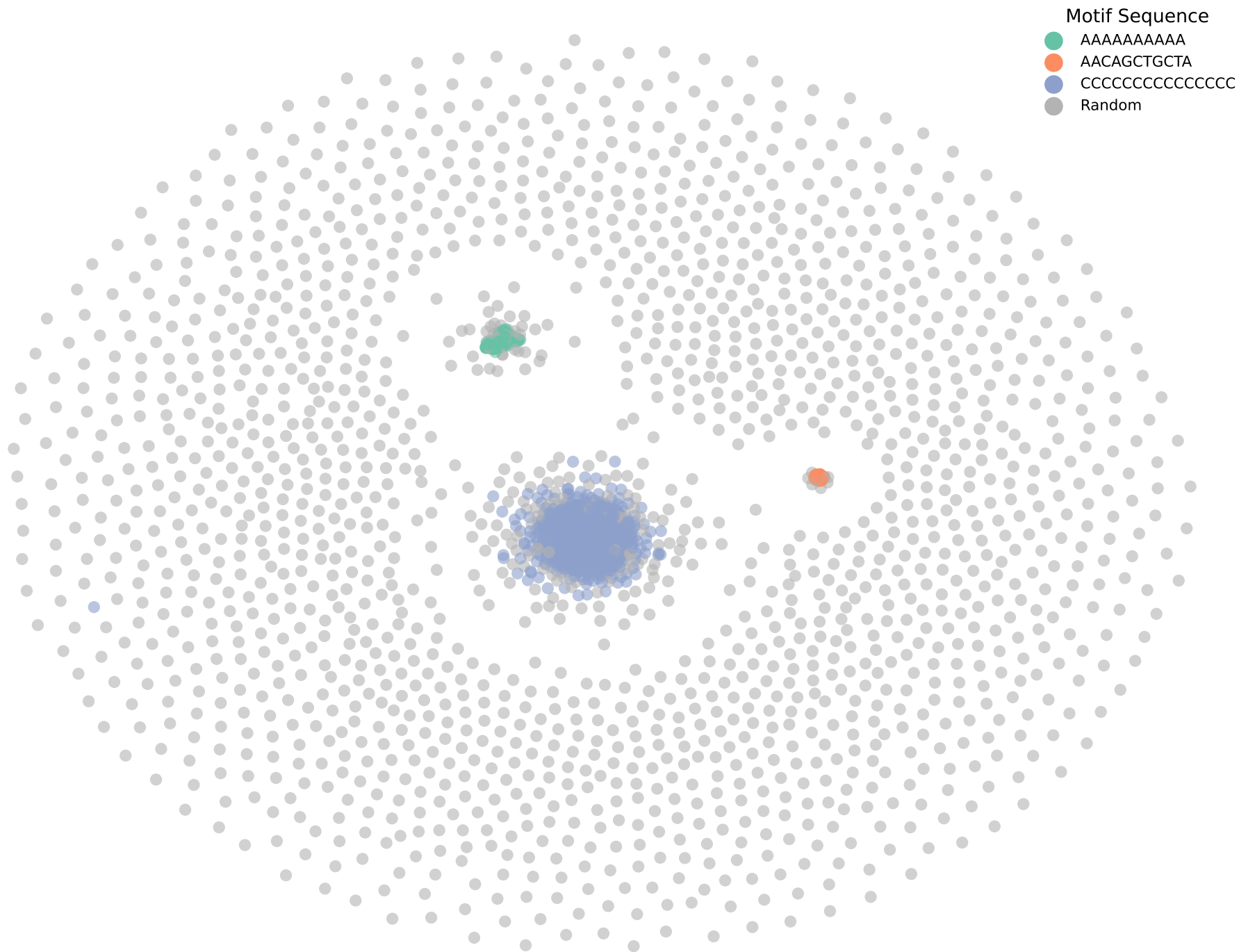

Supplement: Supplement 8 [file Supplemental_Data_1.zip › Supplemental_Data_1/Ascl2_TAGGGC20NCG_Z_3/Ascl2_TAGGGC20NCG_Z_3_KMAP.pdf]

MDS Plot - AscI2\_TAGGGC20NCG\_Z\_3

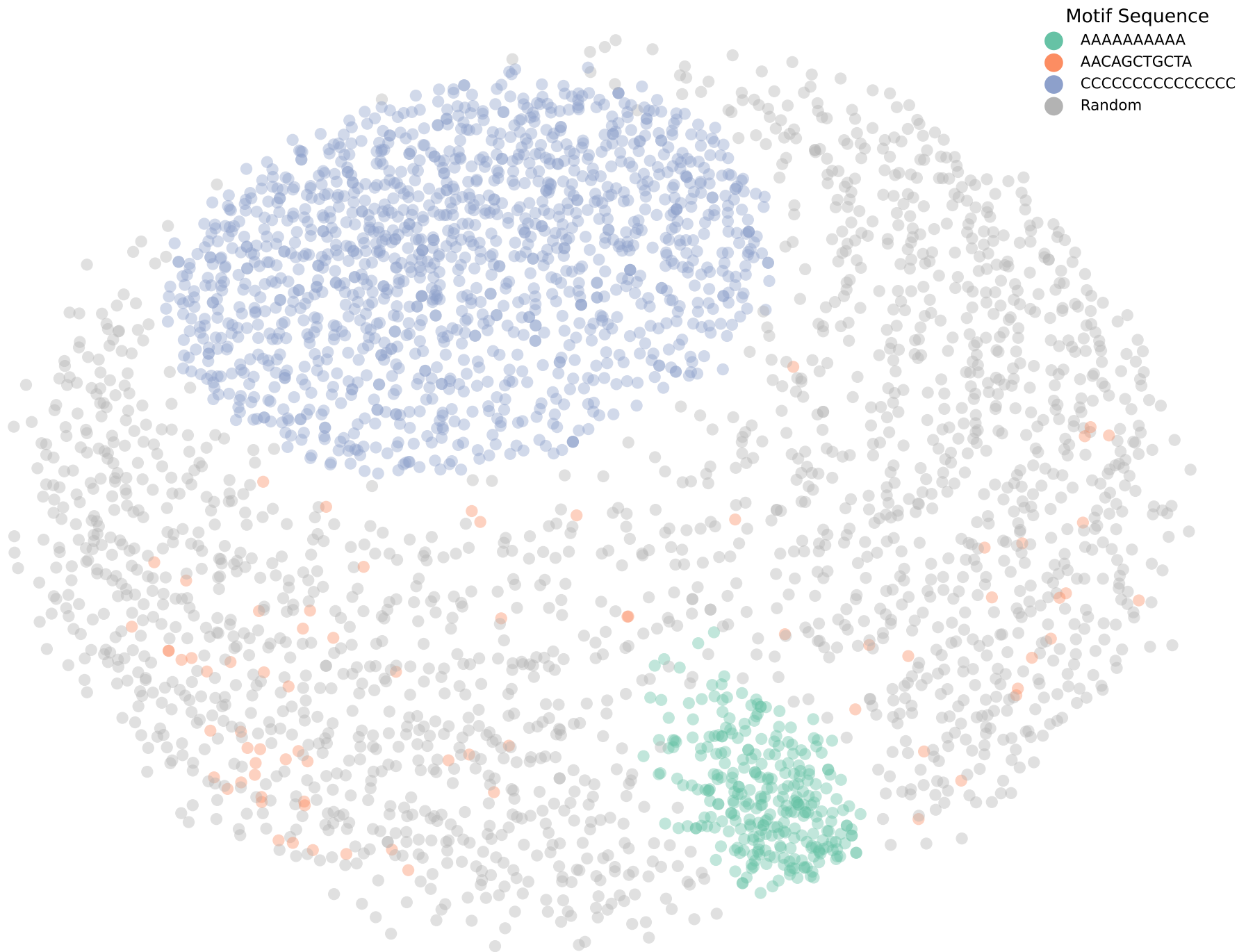

Supplement: Supplement 8 [file Supplemental_Data_1.zip › Supplemental_Data_1/Ascl2_TAGGGC20NCG_Z_3/Ascl2_TAGGGC20NCG_Z_3_MDS.pdf]

PCA Plot - Ascl2\_TAGGGC20NCG\_Z\_3

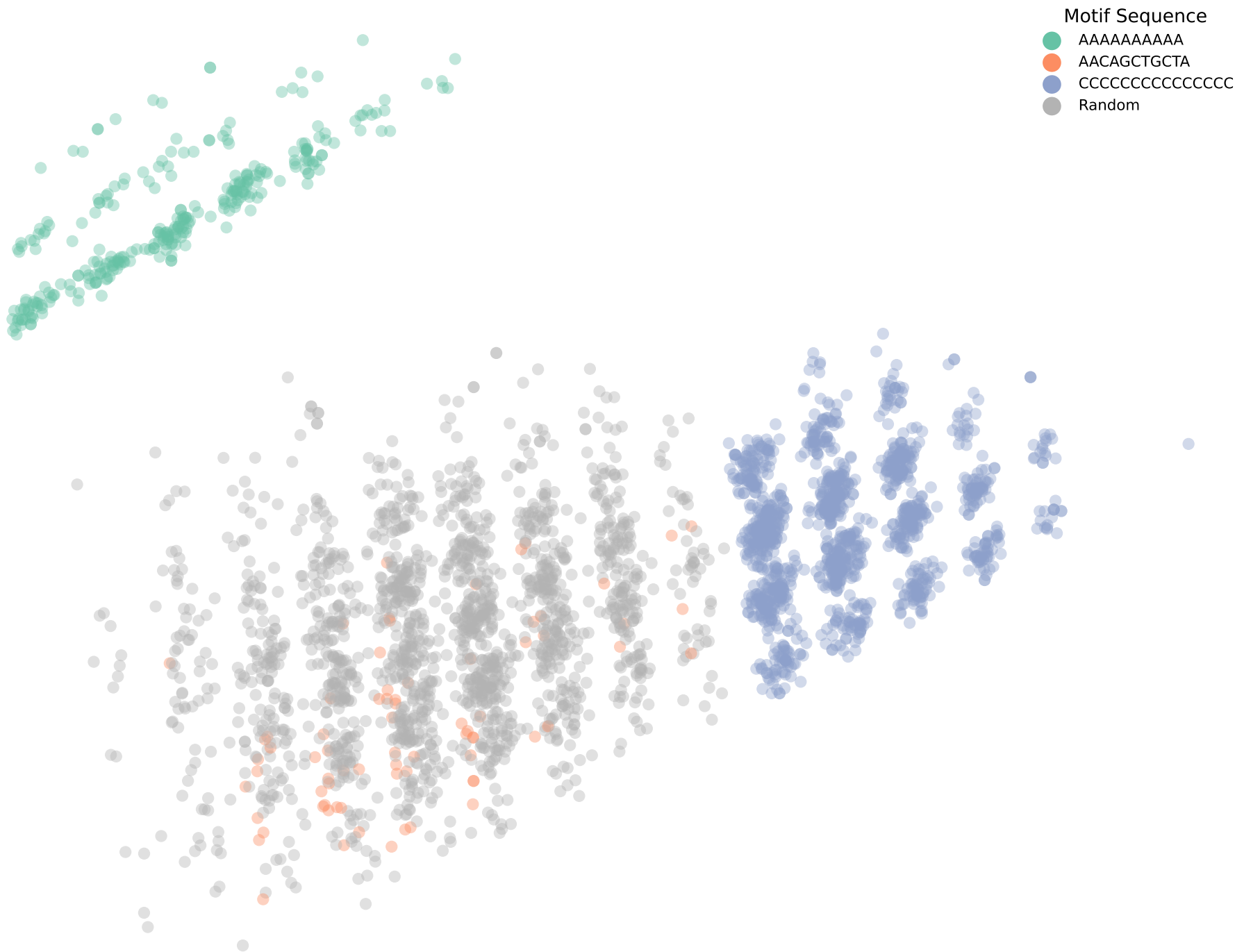

Supplement: Supplement 8 [file Supplemental_Data_1.zip › Supplemental_Data_1/Ascl2_TAGGGC20NCG_Z_3/Ascl2_TAGGGC20NCG_Z_3_PCA.pdf]

tSNE Plot - AscI2\_TAGGGC20NCG\_Z\_3

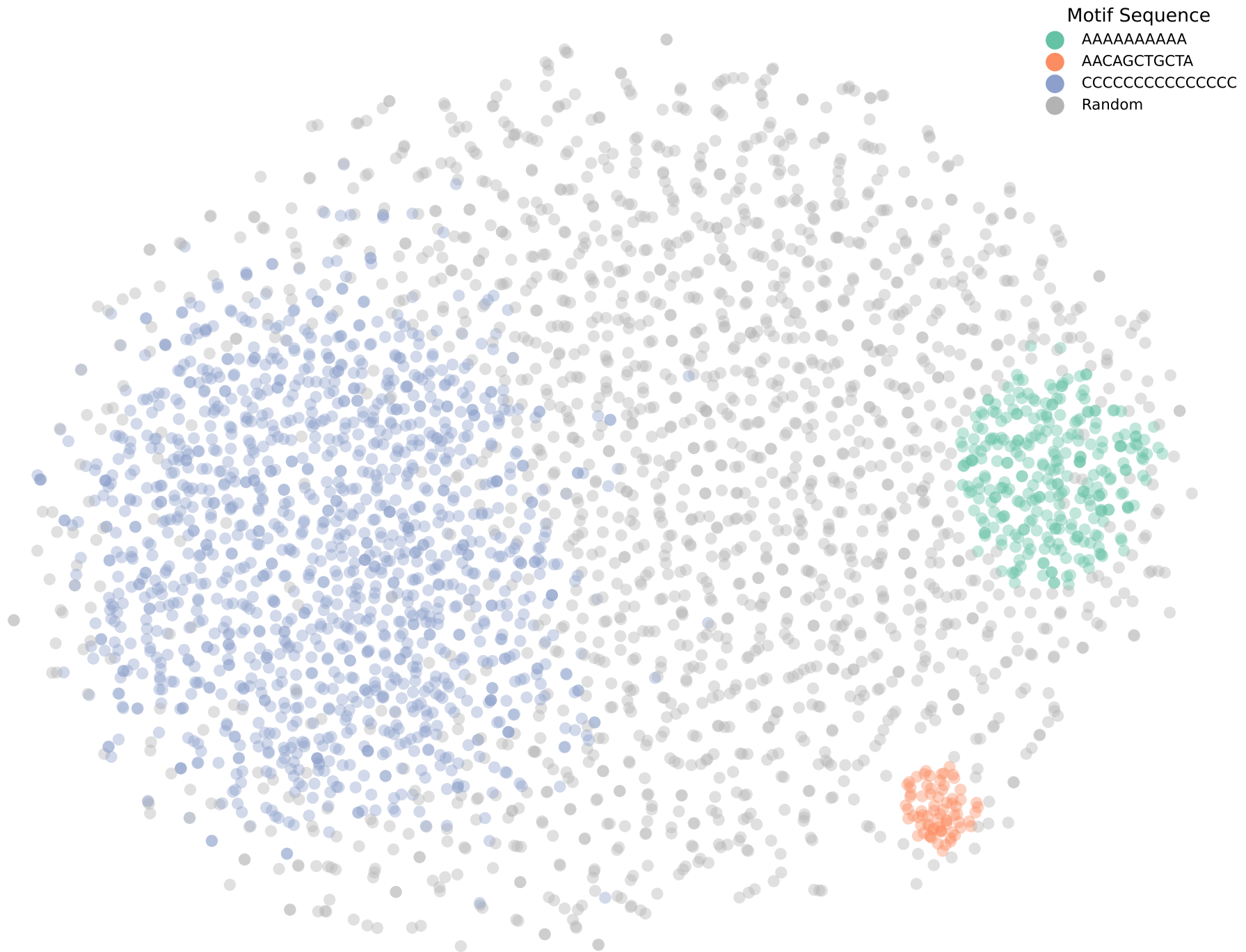

Supplement: Supplement 8 [file Supplemental_Data_1.zip › Supplemental_Data_1/Ascl2_TAGGGC20NCG_Z_3/Ascl2_TAGGGC20NCG_Z_3_tSNE.pdf]

UMAP Plot - AscI2\_TAGGGC20NCG\_Z\_3

Motif Sequence

- AAAAAAAAAA
- AACAGCTGCTA
- CCCCCCCCCCCC
- Random

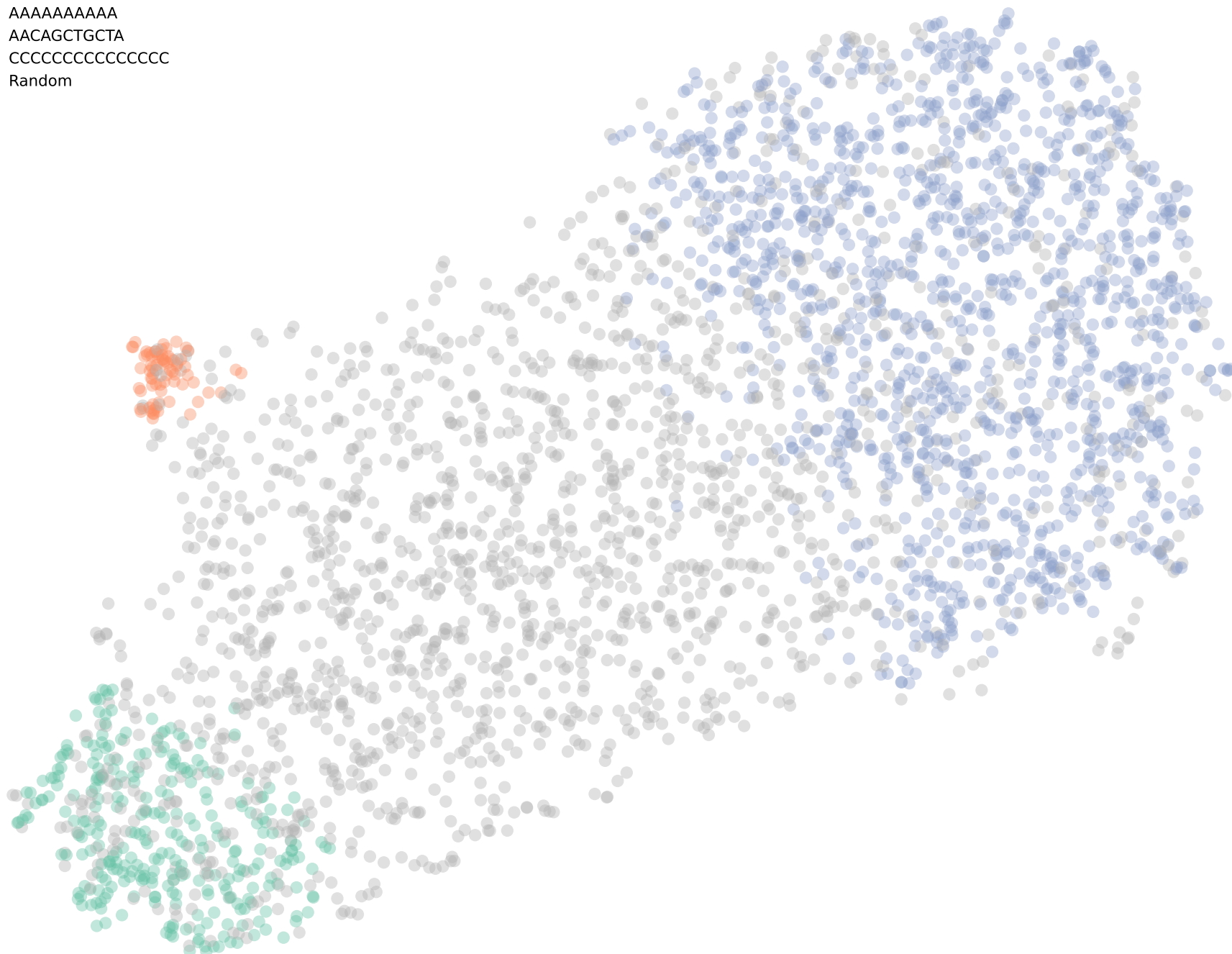

Supplement: Supplement 8 [file Supplemental_Data_1.zip › Supplemental_Data_1/Ascl2_TAGGGC20NCG_Z_3/Ascl2_TAGGGC20NCG_Z_3_UMAP.pdf]

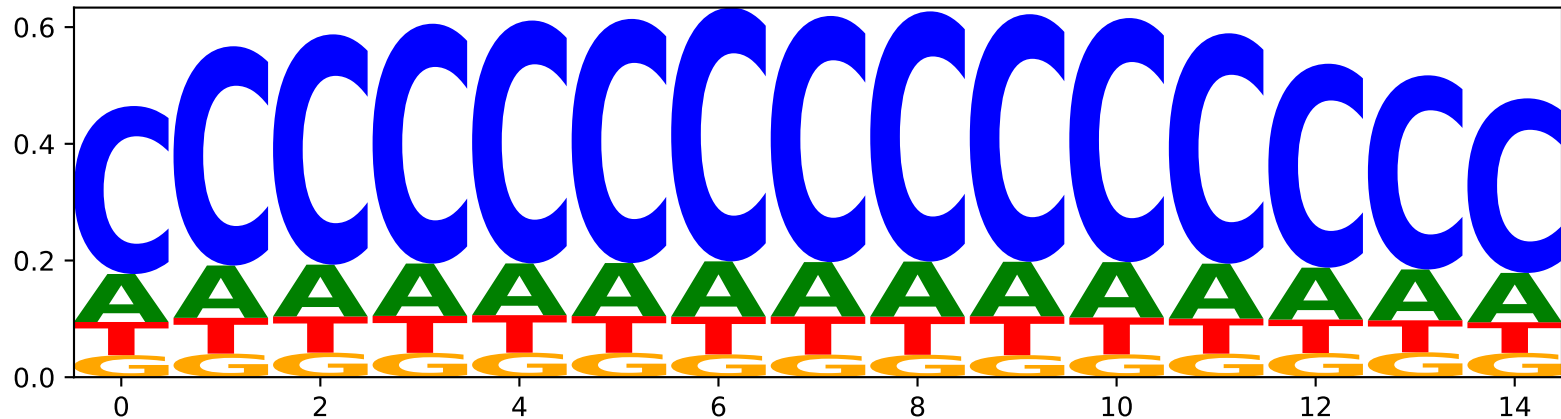

Supplement: Supplement 8 [file Supplemental_Data_1.zip › Supplemental_Data_1/Ascl2_TAGGGC20NCG_Z_3/kmap_logo.pdf]

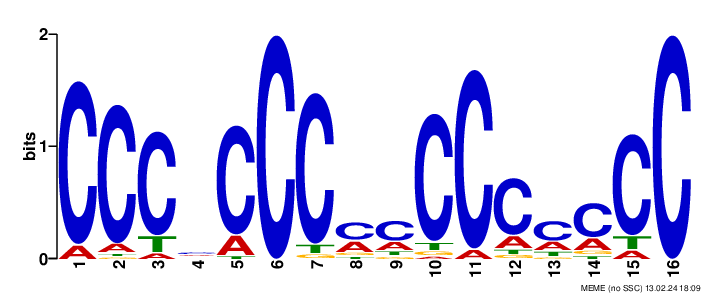

Supplement: Supplement 8 [file Supplemental_Data_1.zip › Supplemental_Data_1/Ascl2_TAGGGC20NCG_Z_3/meme_logo.png]

KMAP LD Plot - AscI2\_TAGGGC20NCG\_Z\_4

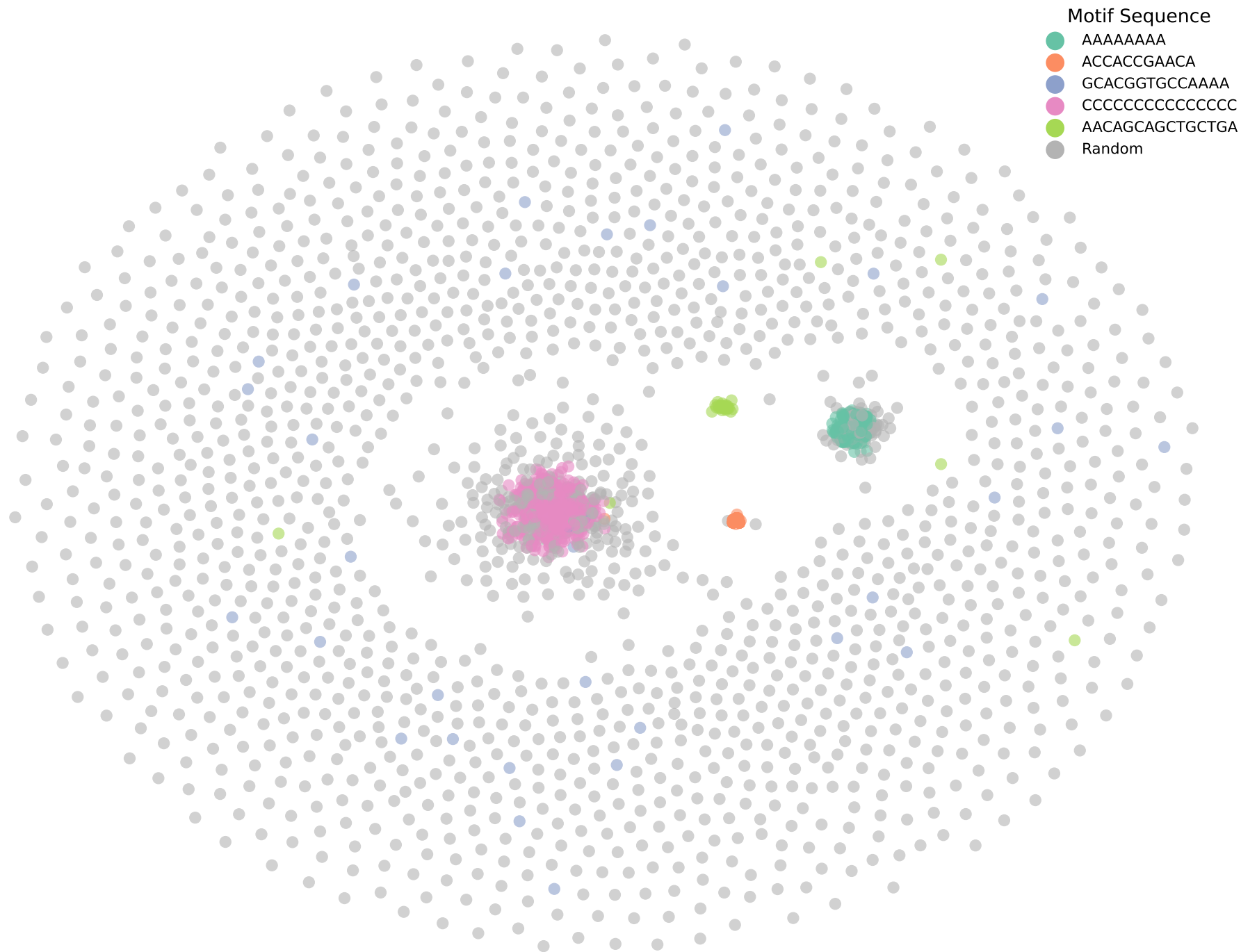

Supplement: Supplement 8 [file Supplemental_Data_1.zip › Supplemental_Data_1/Ascl2_TAGGGC20NCG_Z_4/Ascl2_TAGGGC20NCG_Z_4_KMAP.pdf]

MDS Plot - AscI2\_TAGGGC20NCG\_Z\_4

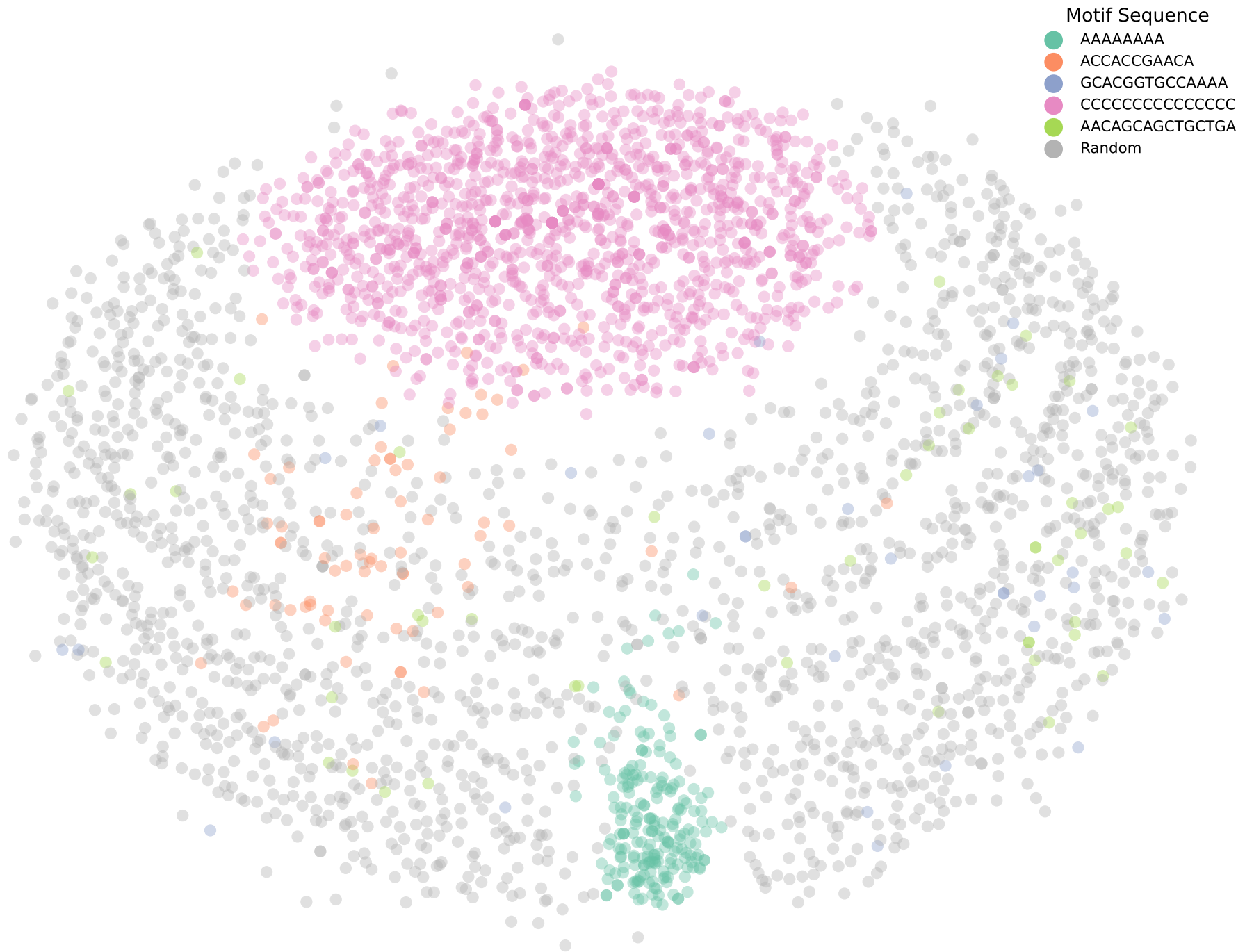

Supplement: Supplement 8 [file Supplemental_Data_1.zip › Supplemental_Data_1/Ascl2_TAGGGC20NCG_Z_4/Ascl2_TAGGGC20NCG_Z_4_MDS.pdf]

PCA Plot - Ascl2\_TAGGGC20NCG\_Z\_4

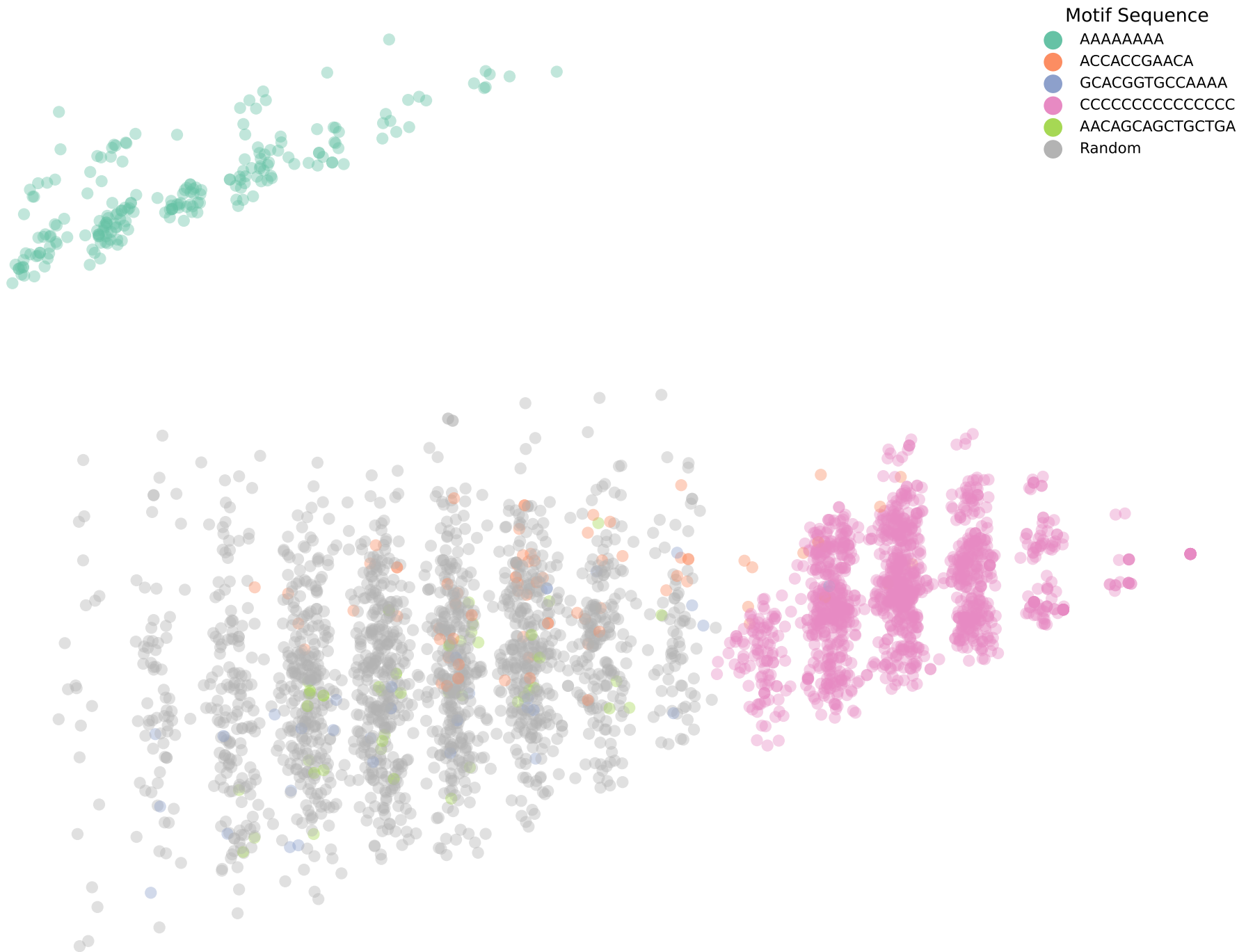

Supplement: Supplement 8 [file Supplemental_Data_1.zip › Supplemental_Data_1/Ascl2_TAGGGC20NCG_Z_4/Ascl2_TAGGGC20NCG_Z_4_PCA.pdf]

UMAP Plot - AscI2\_TAGGGC20NCG\_Z\_4

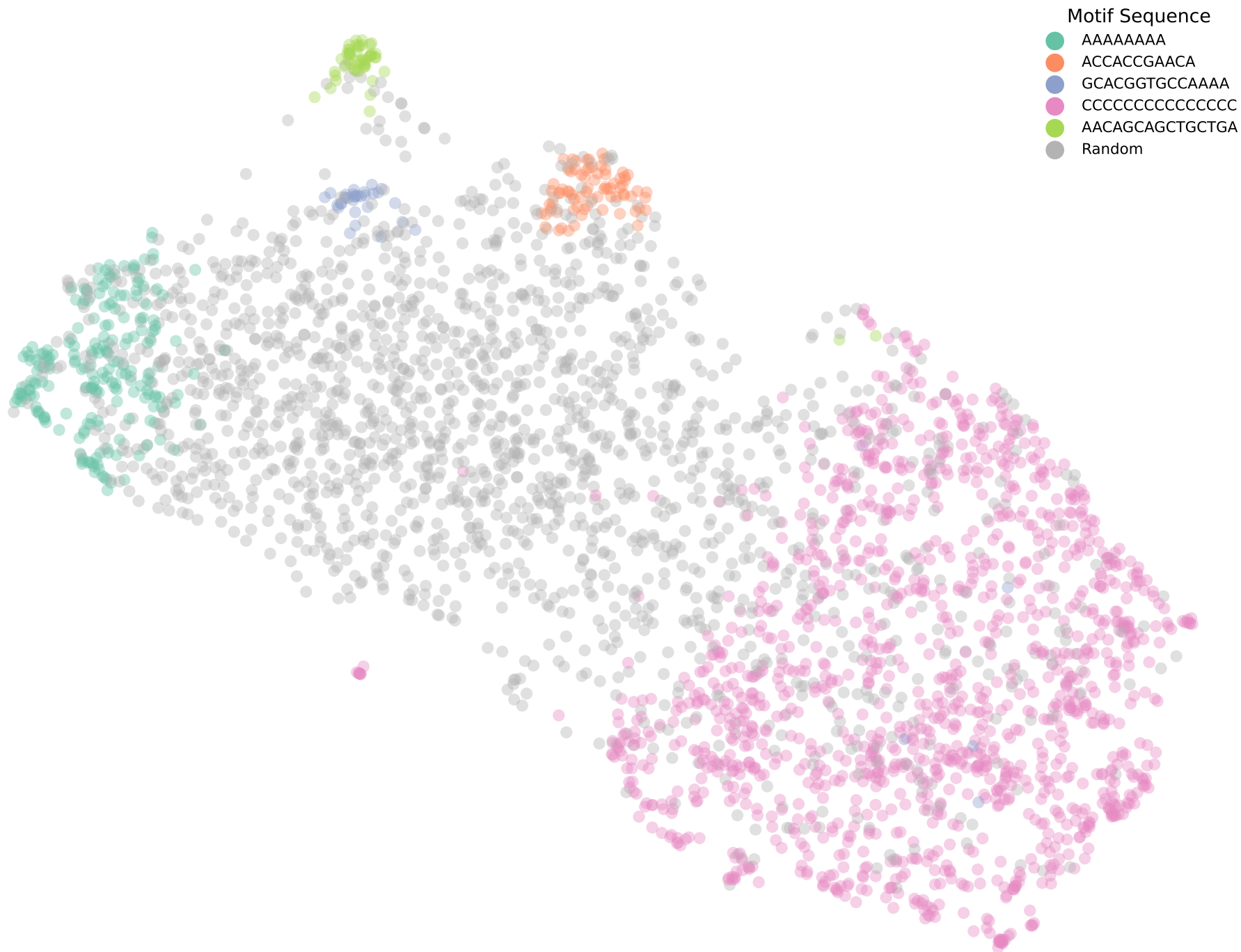

Supplement: Supplement 8 [file Supplemental_Data_1.zip › Supplemental_Data_1/Ascl2_TAGGGC20NCG_Z_4/Ascl2_TAGGGC20NCG_Z_4_UMAP.pdf]

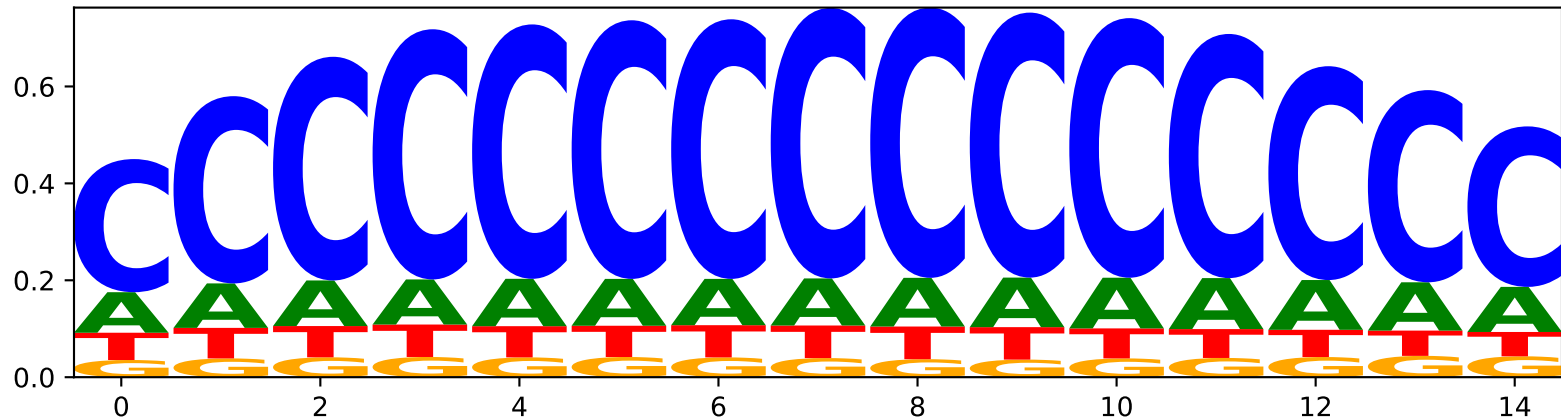

Supplement: Supplement 8 [file Supplemental_Data_1.zip › Supplemental_Data_1/Ascl2_TAGGGC20NCG_Z_4/kmap_logo.pdf]

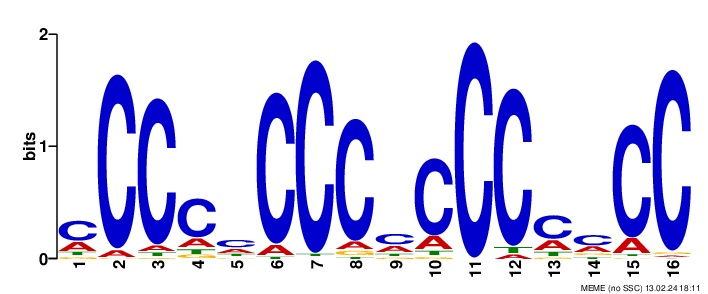

Supplement: Supplement 8 [file Supplemental_Data_1.zip › Supplemental_Data_1/Ascl2_TAGGGC20NCG_Z_4/meme_logo.png]

KMAP LD Plot - Atf4\_TGCCGC20NGA\_Z\_3

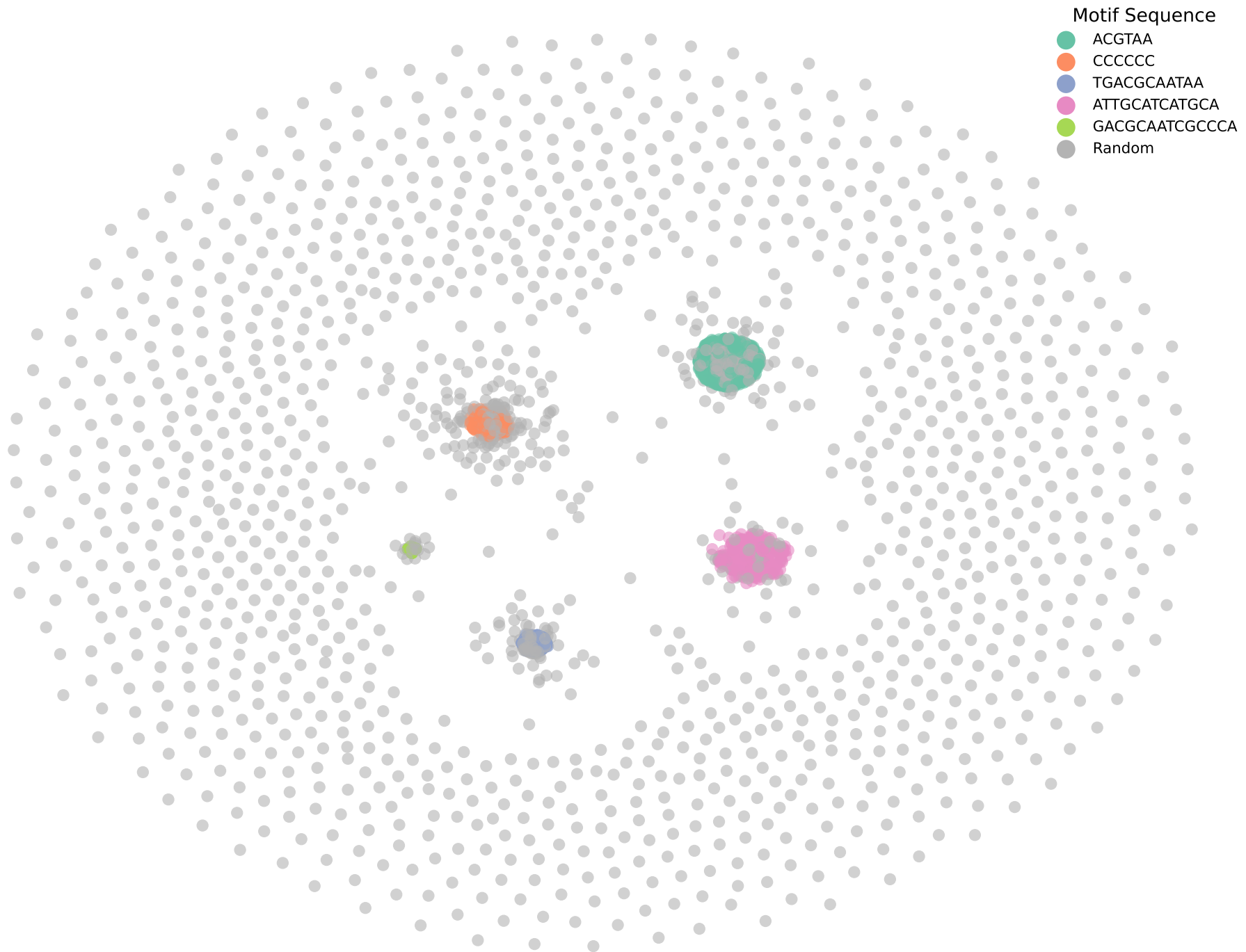

Supplement: Supplement 8 [file Supplemental_Data_1.zip › Supplemental_Data_1/Atf4_TGCCGC20NGA_Z_3/Atf4_TGCCGC20NGA_Z_3_KMAP.pdf]

MDS Plot - Atf4\_TGCCGC20NGA\_Z\_3

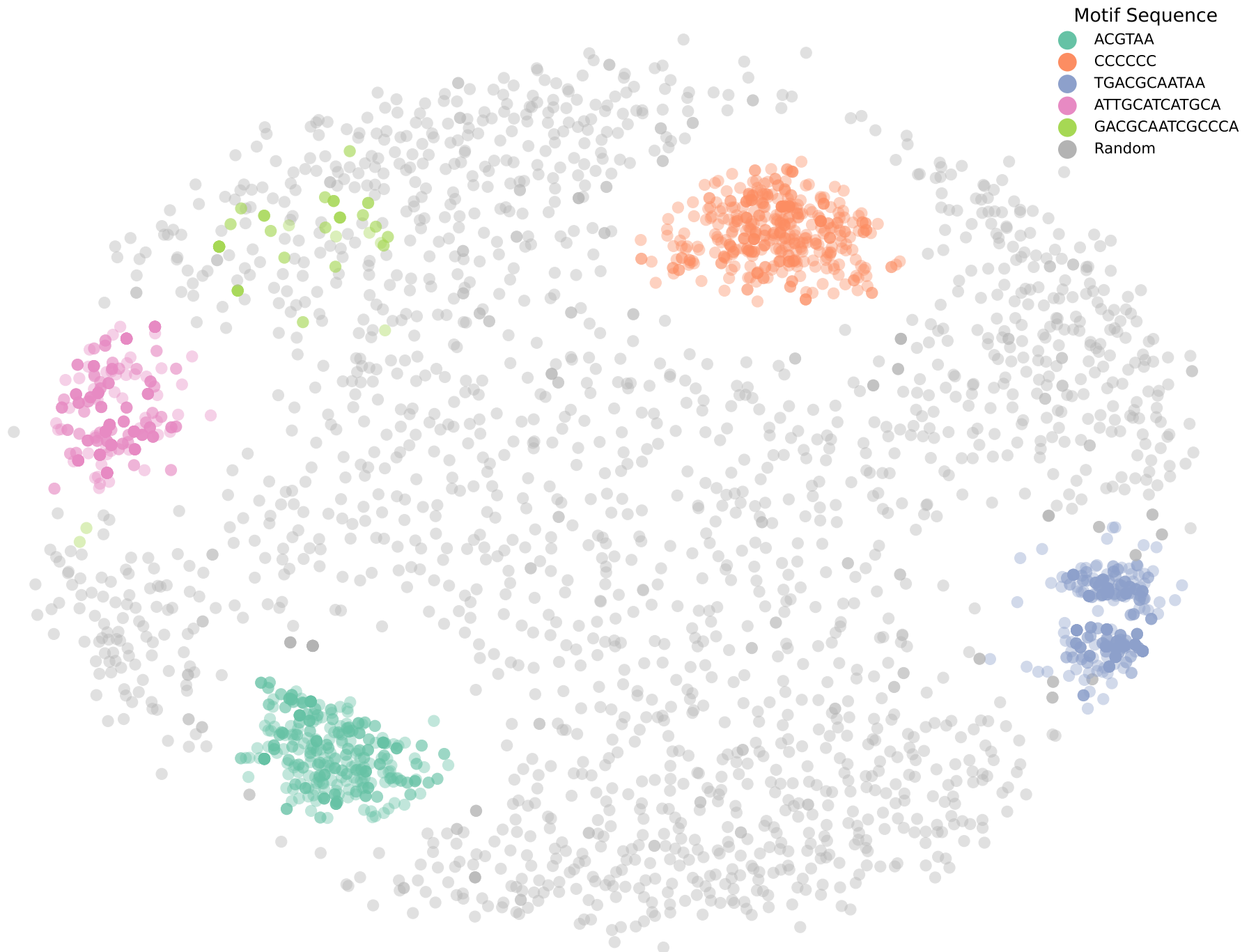

Supplement: Supplement 8 [file Supplemental_Data_1.zip › Supplemental_Data_1/Atf4_TGCCGC20NGA_Z_3/Atf4_TGCCGC20NGA_Z_3_MDS.pdf]

PCA Plot - Atf4\_TGCCGC20NGA\_Z\_3

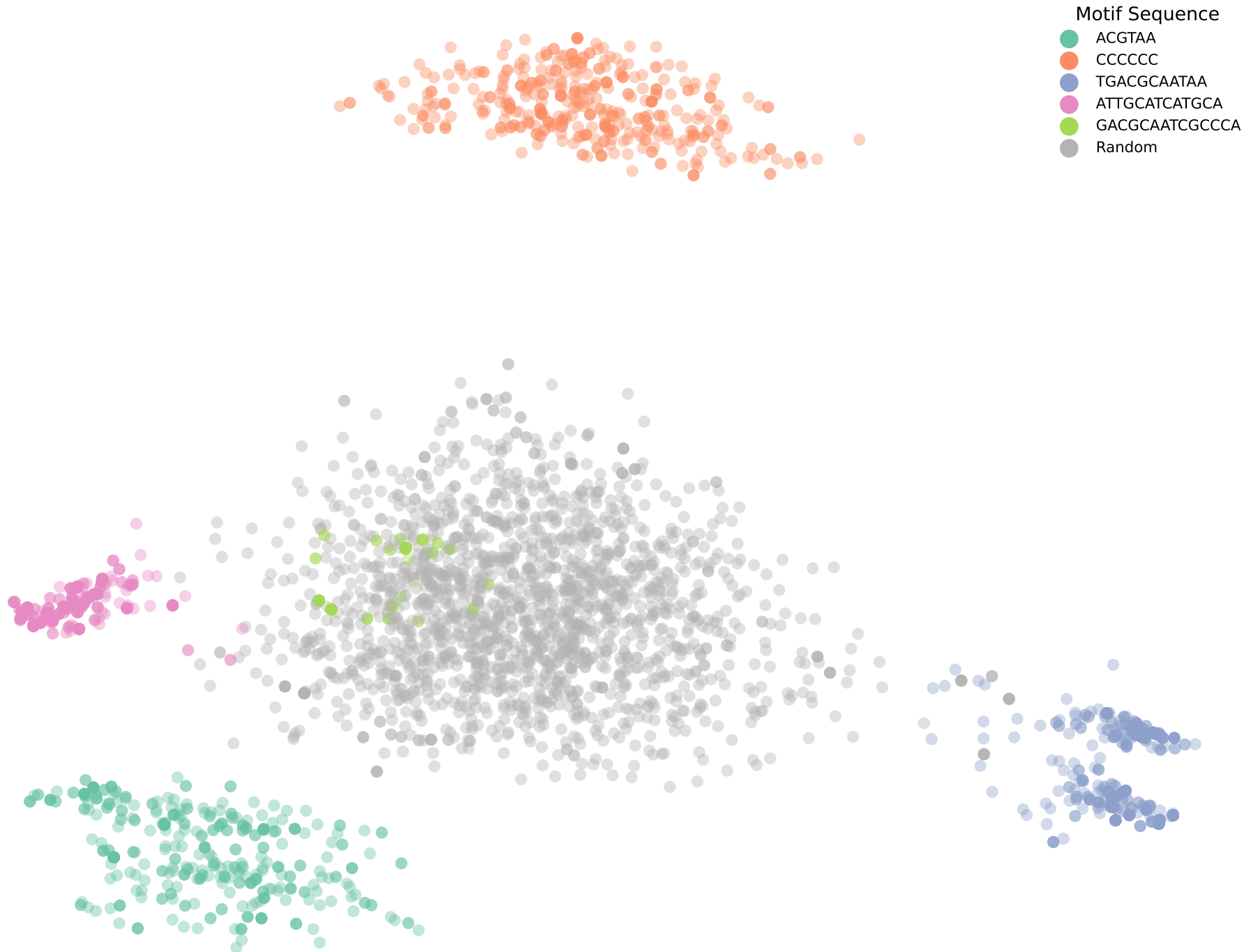

Supplement: Supplement 8 [file Supplemental_Data_1.zip › Supplemental_Data_1/Atf4_TGCCGC20NGA_Z_3/Atf4_TGCCGC20NGA_Z_3_PCA.pdf]

tSNE Plot - Atf4\_TGCCGC20NGA\_Z\_3

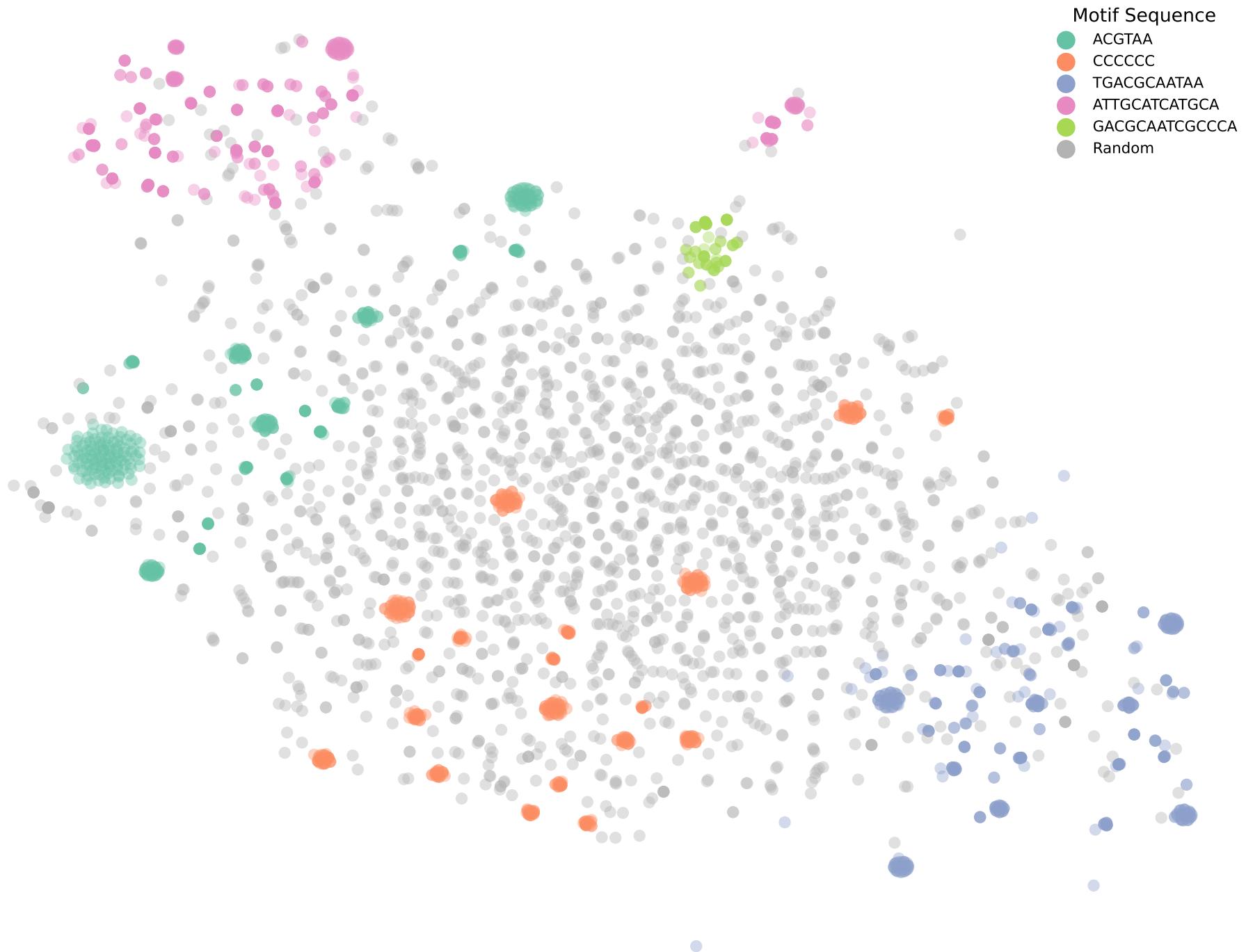

Supplement: Supplement 8 [file Supplemental_Data_1.zip › Supplemental_Data_1/Atf4_TGCCGC20NGA_Z_3/Atf4_TGCCGC20NGA_Z_3_tSNE.pdf]

UMAP Plot - Atf4\_TGCCGC20NGA\_Z\_3

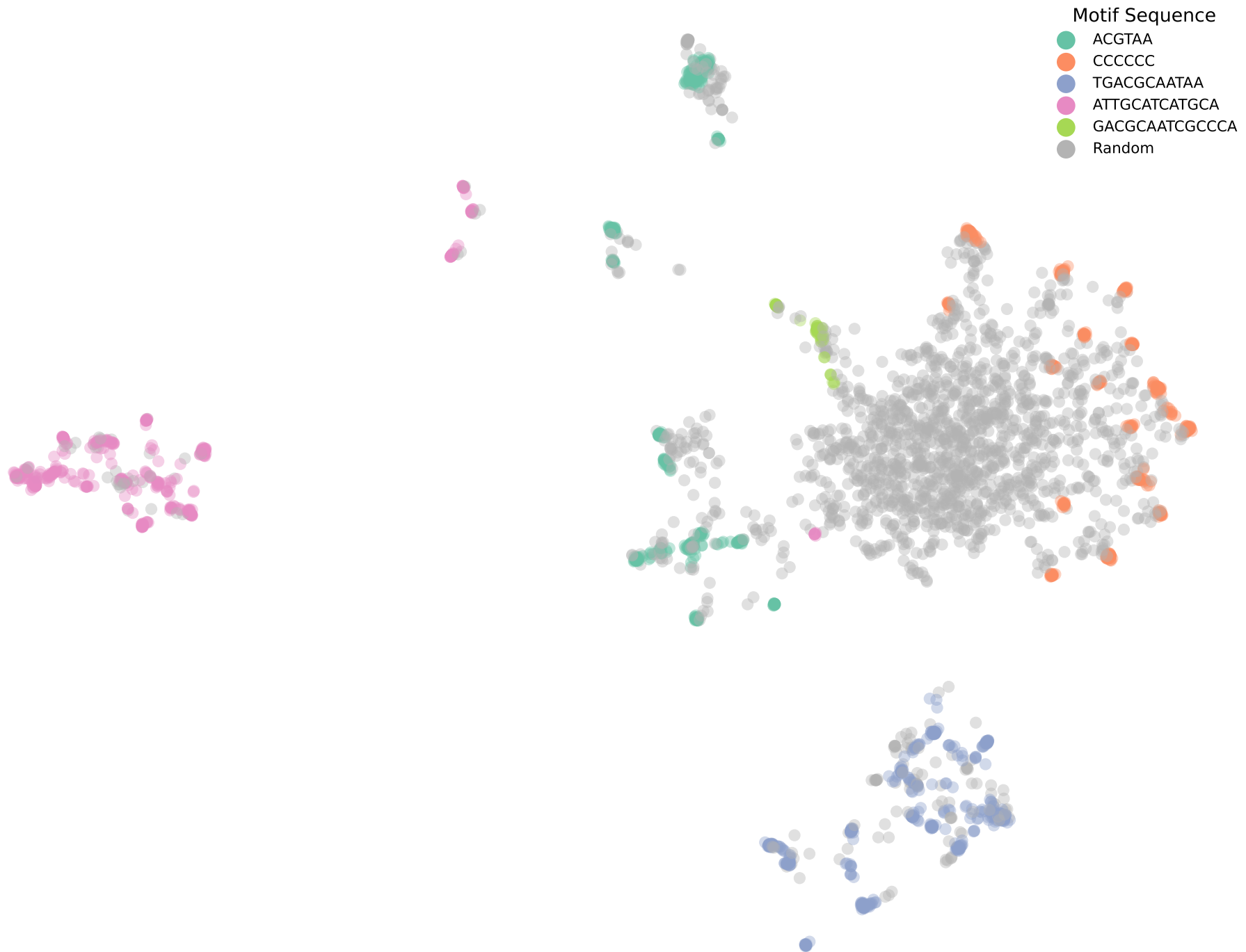

Supplement: Supplement 8 [file Supplemental_Data_1.zip › Supplemental_Data_1/Atf4_TGCCGC20NGA_Z_3/Atf4_TGCCGC20NGA_Z_3_UMAP.pdf]

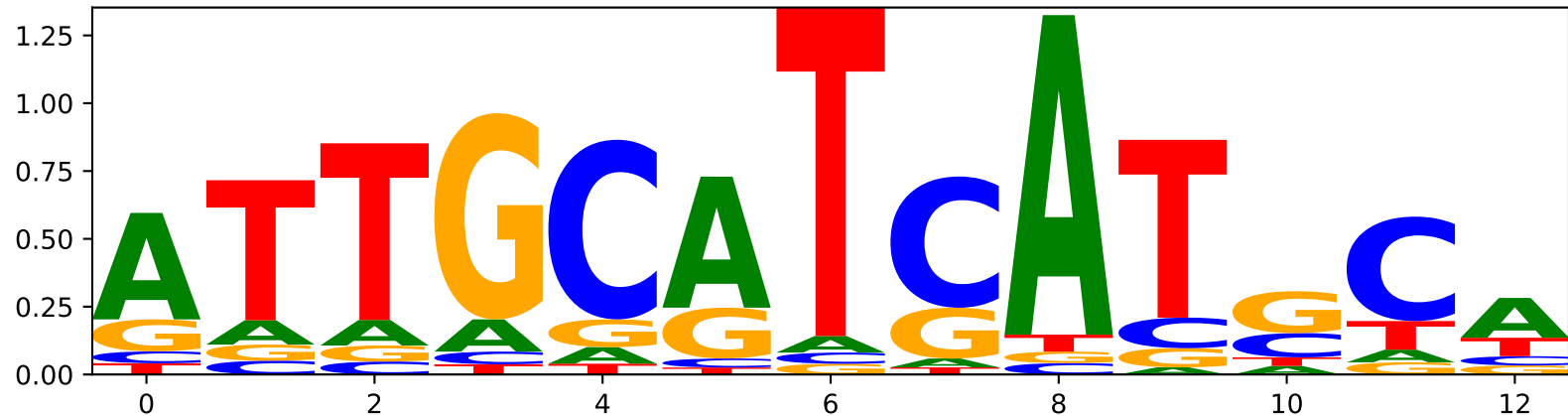

Supplement: Supplement 8 [file Supplemental_Data_1.zip › Supplemental_Data_1/Atf4_TGCCGC20NGA_Z_3/kmap_logo.pdf]

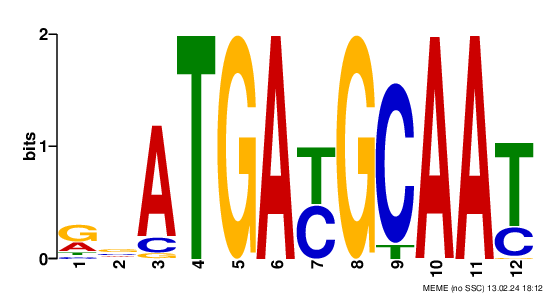

Supplement: Supplement 8 [file Supplemental_Data_1.zip › Supplemental_Data_1/Atf4_TGCCGC20NGA_Z_3/meme_logo.png]

KMAP LD Plot - Atf4\_TGCCGC20NGA\_Z\_4

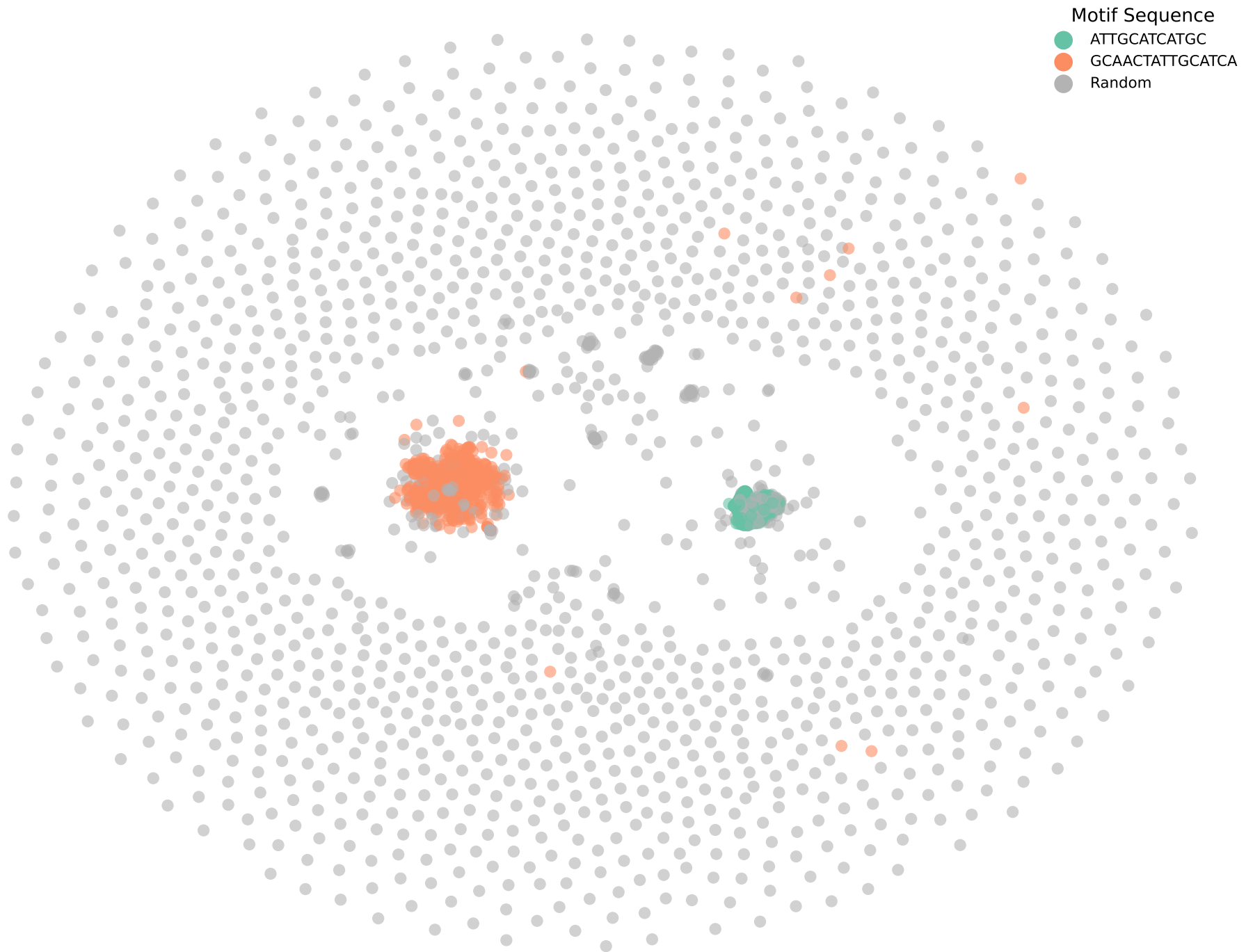

Supplement: Supplement 8 [file Supplemental_Data_1.zip › Supplemental_Data_1/Atf4_TGCCGC20NGA_Z_4/Atf4_TGCCGC20NGA_Z_4_KMAP.pdf]

MDS Plot - Atf4\_TGCCGC20NGA\_Z\_4

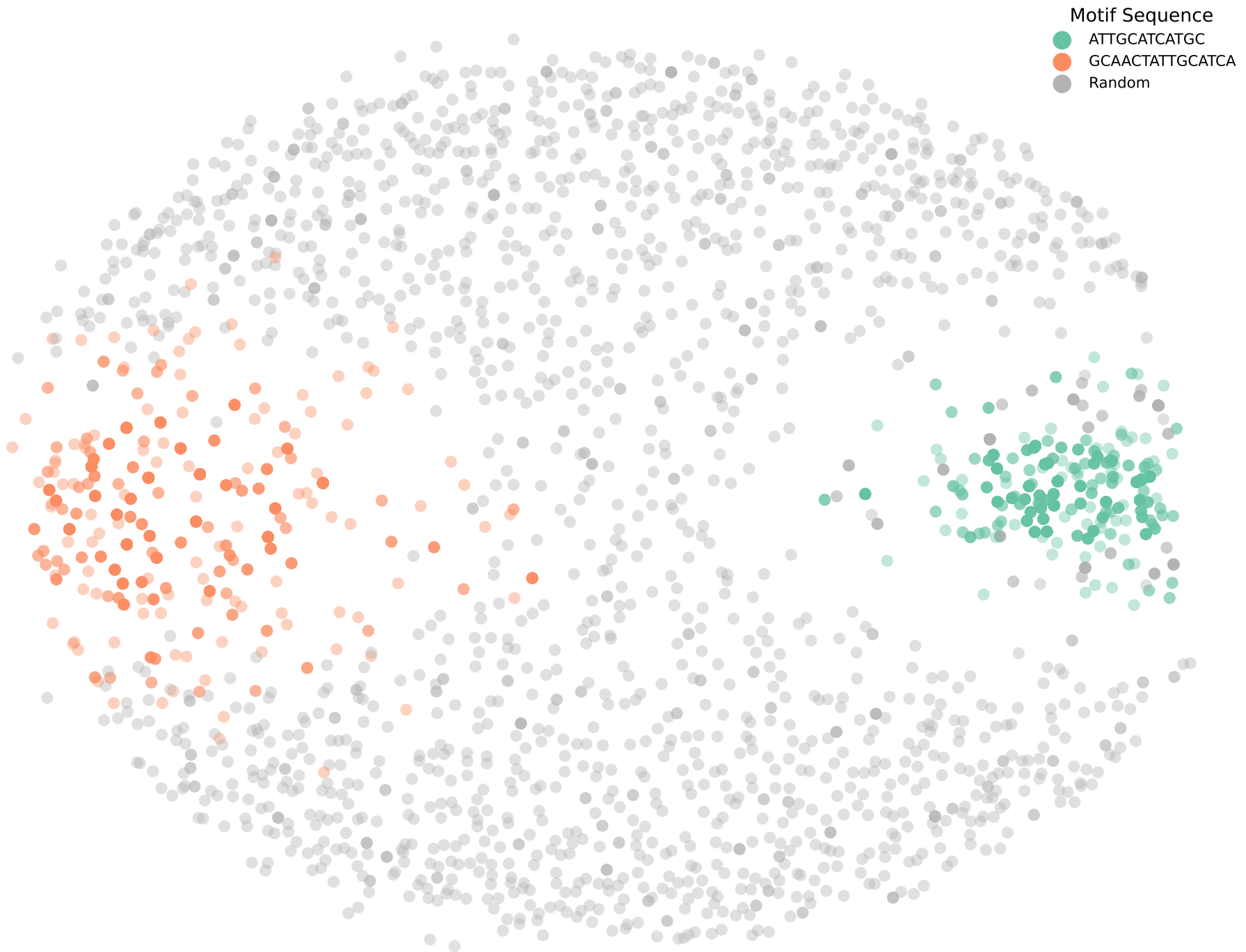

Supplement: Supplement 8 [file Supplemental_Data_1.zip › Supplemental_Data_1/Atf4_TGCCGC20NGA_Z_4/Atf4_TGCCGC20NGA_Z_4_MDS.pdf]

PCA Plot - Atf4\_TGCCGC20NGA\_Z\_4

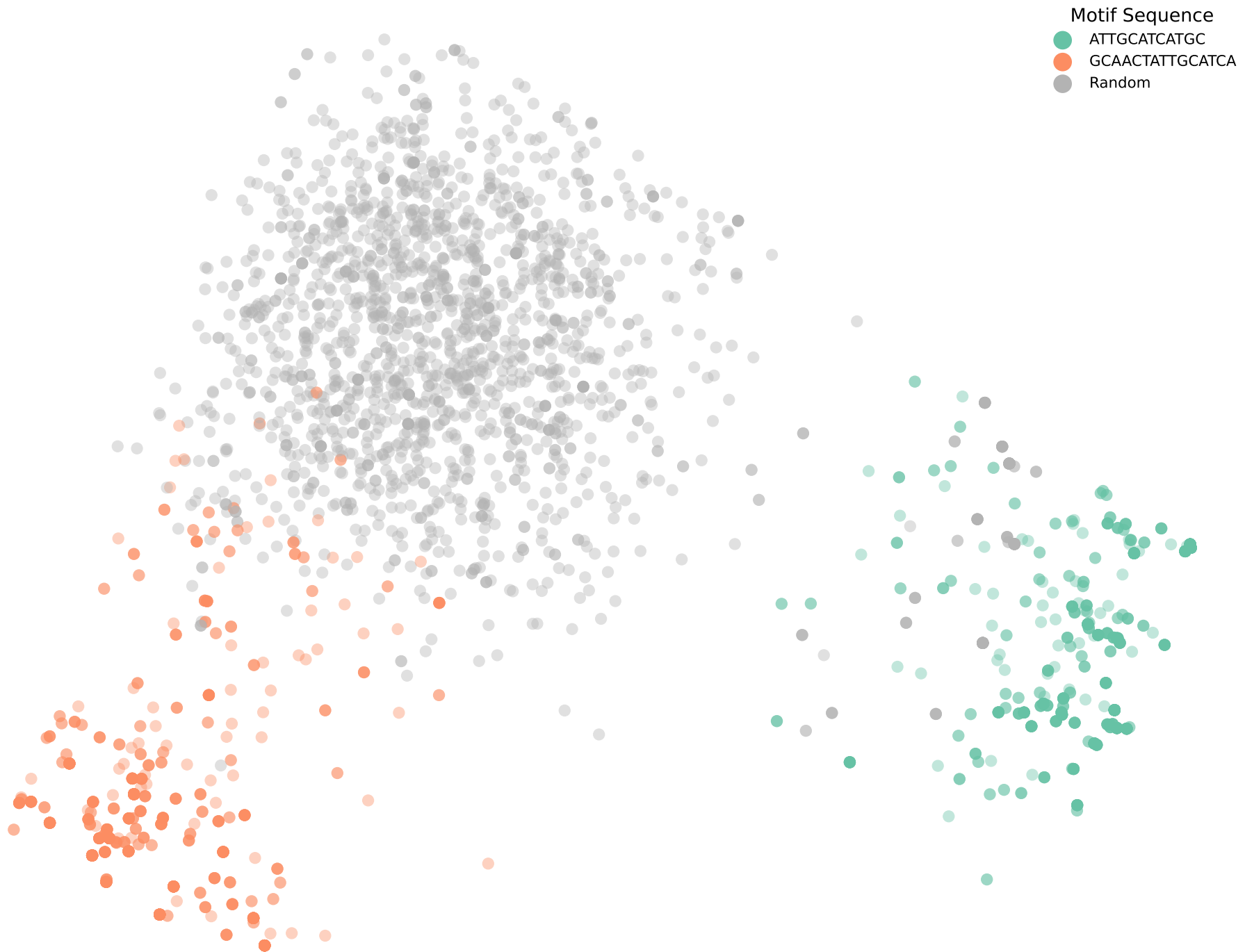

Supplement: Supplement 8 [file Supplemental_Data_1.zip › Supplemental_Data_1/Atf4_TGCCGC20NGA_Z_4/Atf4_TGCCGC20NGA_Z_4_PCA.pdf]

tSNE Plot - Atf4\_TGCCGC20NGA\_Z\_4

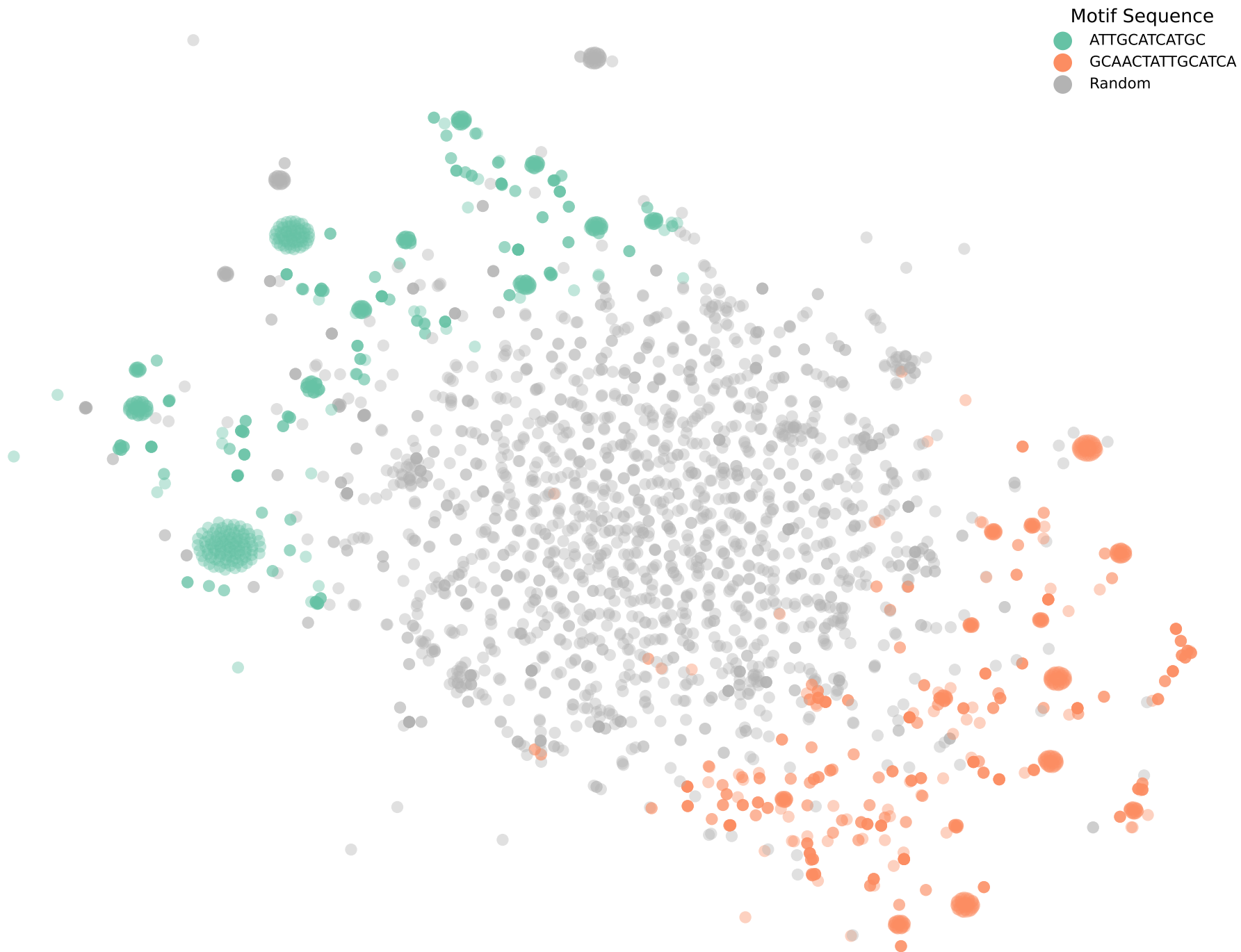

Supplement: Supplement 8 [file Supplemental_Data_1.zip › Supplemental_Data_1/Atf4_TGCCGC20NGA_Z_4/Atf4_TGCCGC20NGA_Z_4_tSNE.pdf]

UMAP Plot - Atf4\_TGCCGC20NGA\_Z\_4

Motif Sequence

- ATTGCATCATGC
- GCAACTATTGCATCA
- Random

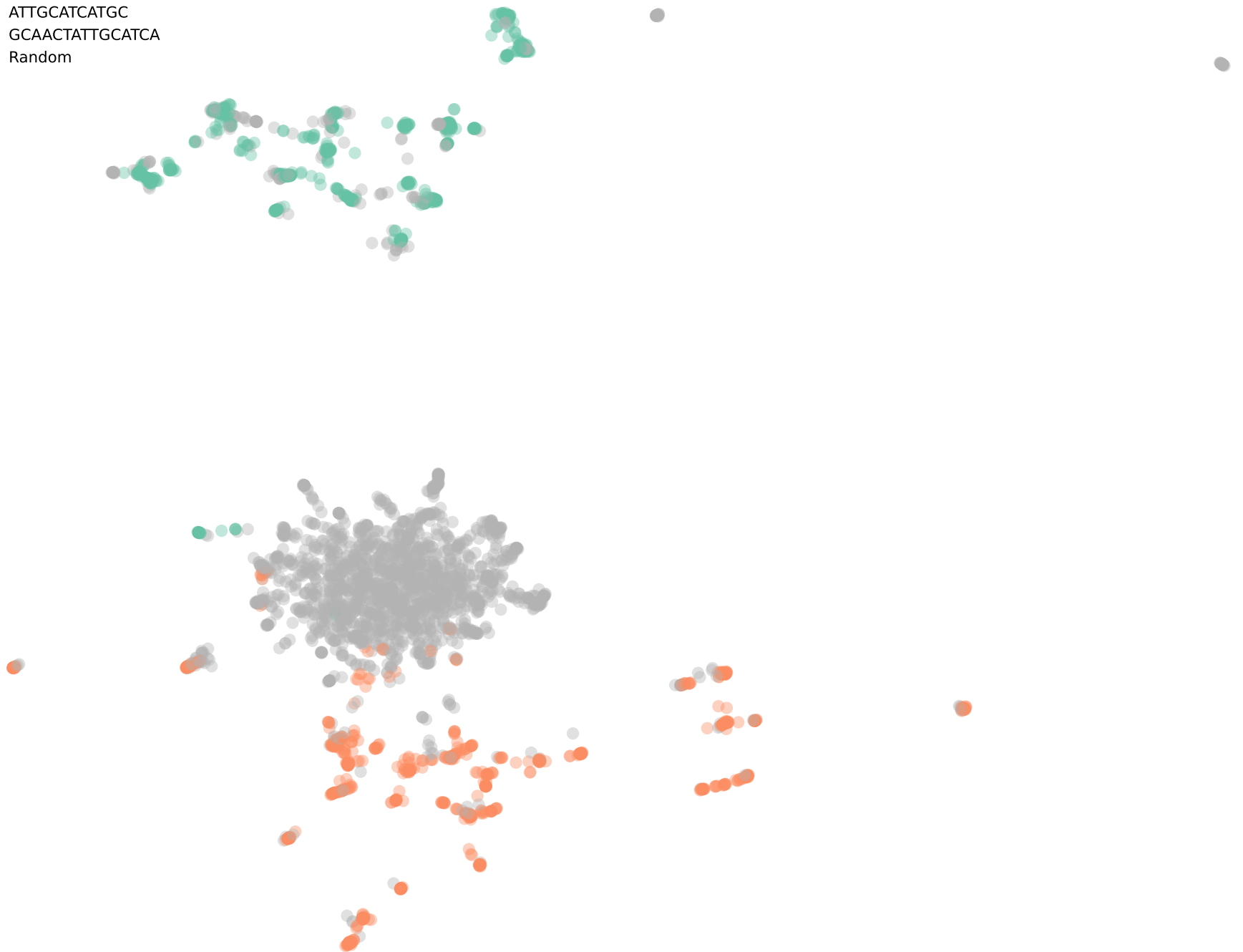

Supplement: Supplement 8 [file Supplemental_Data_1.zip › Supplemental_Data_1/Atf4_TGCCGC20NGA_Z_4/Atf4_TGCCGC20NGA_Z_4_UMAP.pdf]

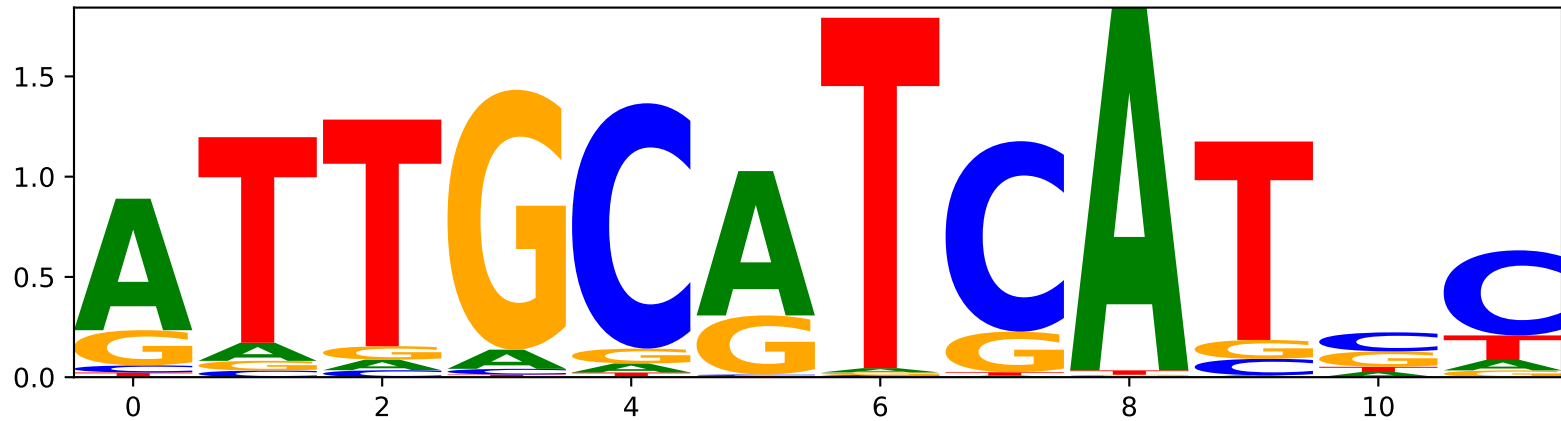

Supplement: Supplement 8 [file Supplemental_Data_1.zip › Supplemental_Data_1/Atf4_TGCCGC20NGA_Z_4/kmap_logo.pdf]

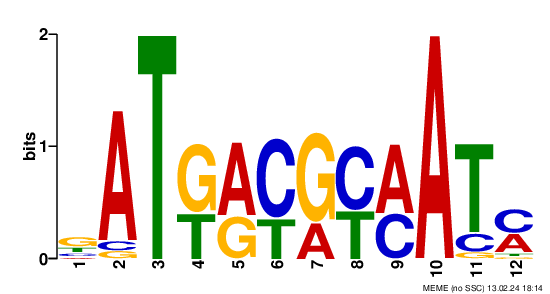

Supplement: Supplement 8 [file Supplemental_Data_1.zip › Supplemental_Data_1/Atf4_TGCCGC20NGA_Z_4/meme_logo.png]

KMAP LD Plot - ATF4\_TGGAAT20NGA\_W\_3

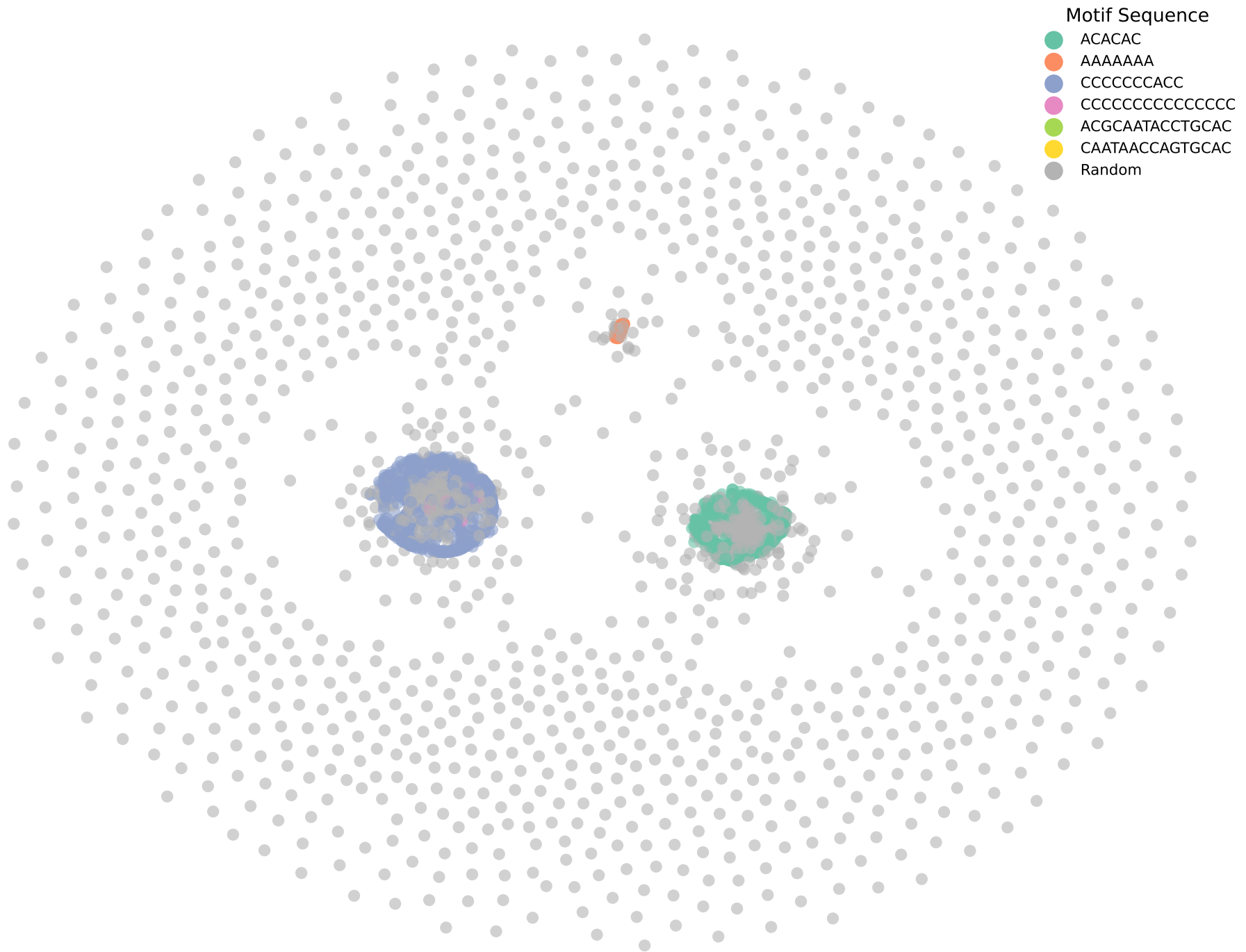

Supplement: Supplement 8 [file Supplemental_Data_1.zip › Supplemental_Data_1/ATF4_TGGAAT20NGA_W_3/ATF4_TGGAAT20NGA_W_3_KMAP.pdf]

MDS Plot - ATF4\_TGGAAT20NGA\_W\_3

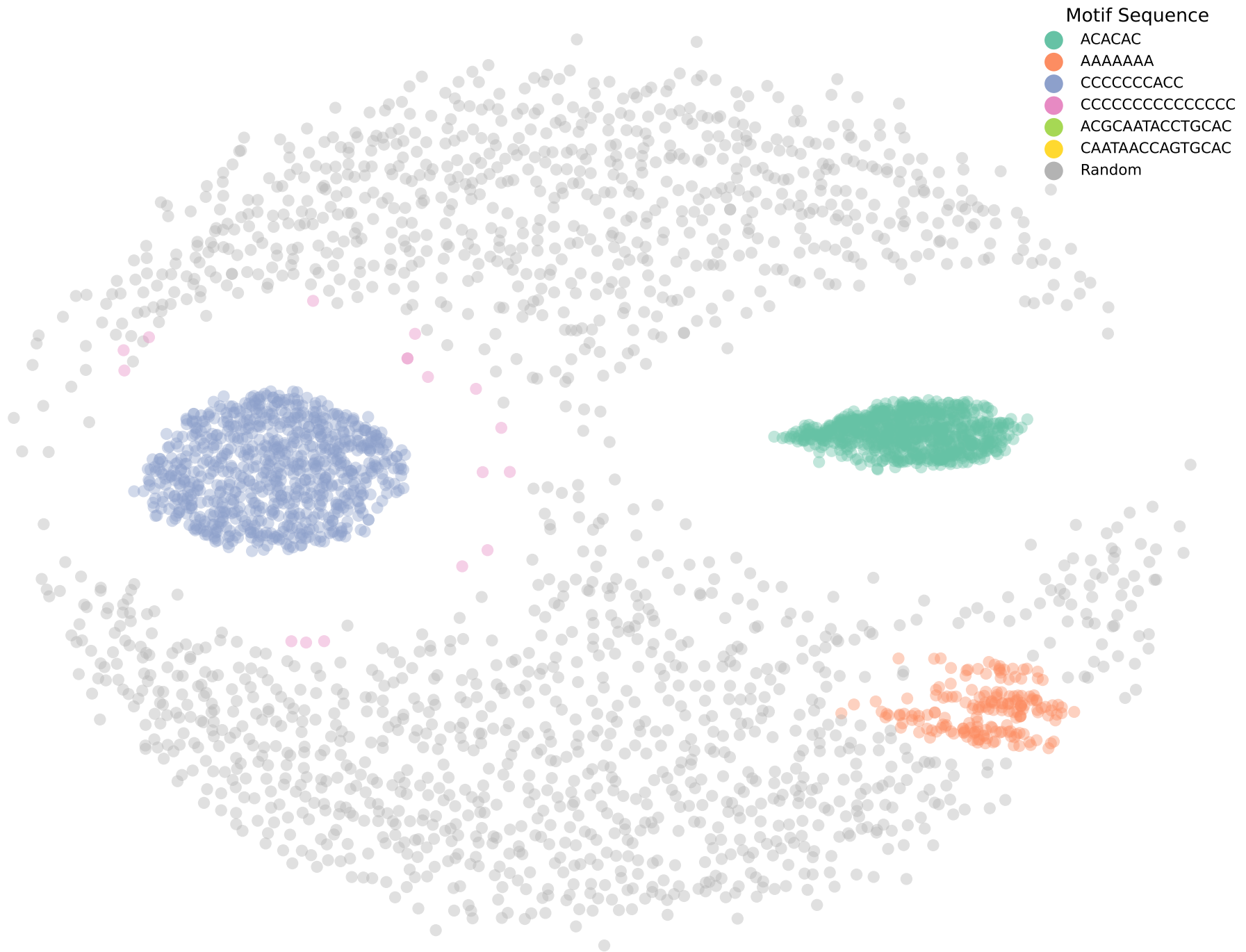

Supplement: Supplement 8 [file Supplemental_Data_1.zip › Supplemental_Data_1/ATF4_TGGAAT20NGA_W_3/ATF4_TGGAAT20NGA_W_3_MDS.pdf]

PCA Plot - ATF4\_TGGAAT20NGA\_W\_3

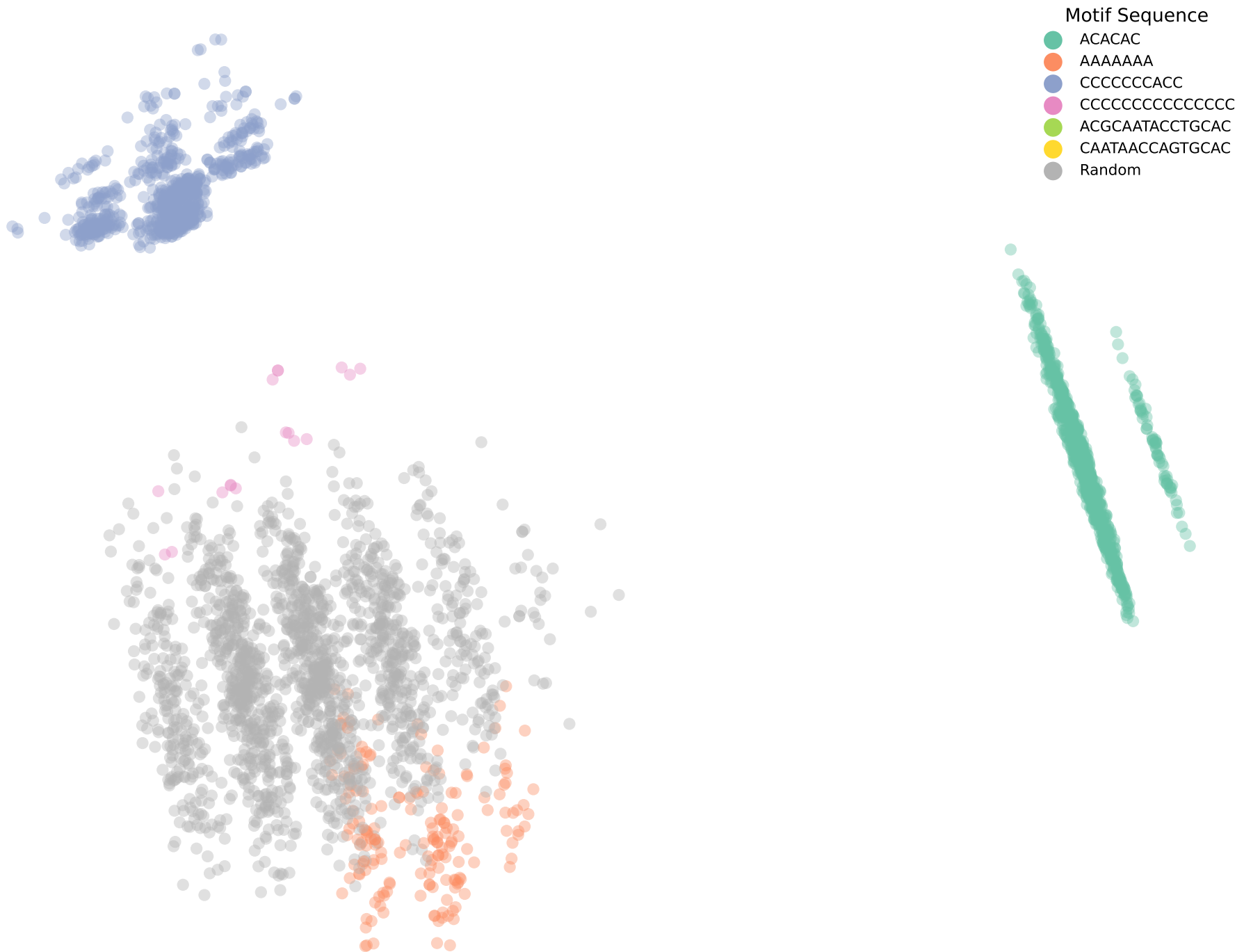

Supplement: Supplement 8 [file Supplemental_Data_1.zip › Supplemental_Data_1/ATF4_TGGAAT20NGA_W_3/ATF4_TGGAAT20NGA_W_3_PCA.pdf]

tSNE Plot - ATF4\_TGGAAT20NGA\_W\_3

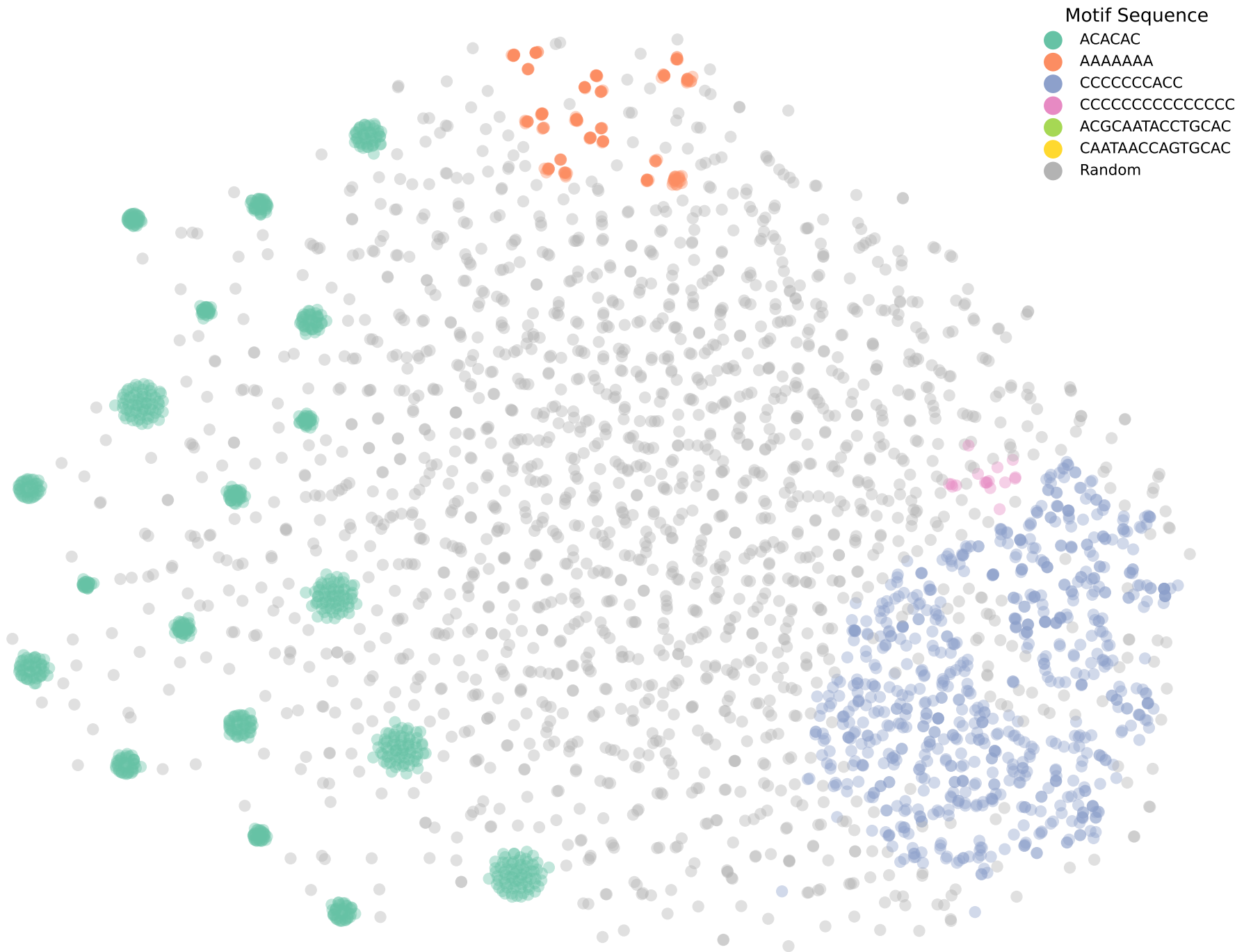

Supplement: Supplement 8 [file Supplemental_Data_1.zip › Supplemental_Data_1/ATF4_TGGAAT20NGA_W_3/ATF4_TGGAAT20NGA_W_3_tSNE.pdf]
